# Supplementary material for: Comprehensive Analysis of Transcriptome and Metabolome Reveals the Flavonoid Metabolic Pathway Is Associated with Fruit Peel Coloration of Melon
Source: Molecules. 2021 May 10;26(9):2830. doi: 10.3390/molecules26092830 (PMC8126211; doi:10.3390/molecules26092830)
Supplement: Supplementary file 1 [file molecules-26-02830-s001.zip › molecules-1183709-supplementary/Table S1 Statistics of the readsí» quality in the RNA-Seq study.docx]

| **Table S1 Statistics of the reads quality in the RNA-Seq study** | | | | | |  |
| --- | --- | --- | --- | --- | --- | --- |
| **Sample name** | **Clean reads** | **Clean bases** | **Mapped reads** | **Q30 (%)** | **GC content (%)** | |
| W | 50909692 | 7636453800 | 46171136（89.41） | 92.5 | 43.67 | |
| B | 46869088 | 7030363200 | 42491215（89.36） | 92.08 | 44.09 | |
| H | 55205315 | 8280797300 | 48892767（87.26） | 92.28 | 43.48 | |
