# Supplementary material for: Comprehensive Analysis of Transcriptome and Metabolome Reveals the Flavonoid Metabolic Pathway Is Associated with Fruit Peel Coloration of Melon
Source: Molecules. 2021 May 10;26(9):2830. doi: 10.3390/molecules26092830 (PMC8126211; doi:10.3390/molecules26092830)
Supplement: Supplementary file 1 [file molecules-26-02830-s001.zip › molecules-1183709-supplementary/Table S10 differentially expressed metabolites in B vs H.docx]

| **Table S10 differentially expressed metabolites in B vs H** | | | | |
| --- | --- | --- | --- | --- |
| **Index** | **Compounds** | **Class** | **LogFC** | **type** |
| pma0214 | methylQuercetin O-hexoside | Flavonol | 12.8182 | up |
| pma0249 | Selgin 5-O-hexoside | Flavone | 14.6118 | up |
| pma0253 | O-methylChrysoeriol 5-O-hexoside | Flavone | 5.5101 | up |
| pma0760 | Selgin O-malonylhexoside | Flavone | 6.4622 | up |
| pma0791 | Naringenin O-malonylhexoside | Flavanone | 18.4246 | up |
| pma0795 | Tricetin O-malonylhexoside | Flavone | 20.8991 | up |
| pma1108 | Apigenin C-glucoside | Flavone | 17.9208 | up |
| pma1116 | Kaempferide | Flavonol | 21.9607 | up |
| pma6218 | O-methylnaringenin C-pentoside | Flavone | 16.9208 | up |
| pma6353 | Chrysin C-hexoside | Flavone | 18.0045 | up |
| pma6516 | C-hexosyl-apigenin O-hexosyl-O-hexoside | Flavone | 21.3566 | up |
| pma6518 | C-pentosyl-chrysoeriol 7-O-feruloylhexoside | Flavone | 16.5118 | up |
| pma6639 | Isorhamnetin O-hexoside | Flavonol | 4.8316 | up |
| pmb0541 | Cyanidin 3-O-glucosyl-malonylglucoside | Anthocyanins | -12.2748 | down |
| pmb0563 | Peonidin | Anthocyanins | 16.8168 | up |
| pmb0566 | Luteolin O-hexosyl-O-pentoside | Flavone | 14.3657 | up |
| pmb0576 | Apigenin O-malonylhexoside | Flavone | 13.7068 | up |
| pmb0578 | Luteolin O-sinapoylhexoside | Flavone | 17.8182 | up |
| pmb0592 | Chrysoeriol O-hexosyl-O-rutinoside | Flavone | 21.4658 | up |
| pmb0595 | Isorhamnetin 5-O-hexoside | Flavonol | 4.0265 | up |
| pmb0604 | Kaempferol 3-O-glucoside (Astragalin) | Flavonol | 17.1475 | up |
| pmb0607 | Chrysoeriol 7-O-hexoside | Flavone | 19.7813 | up |
| pmb0608 | Chrysoeriol O-malonylhexoside | Flavone | 4.1356 | up |
| pmb0618 | 8-C-hexosyl-hesperetin O-hexoside | Flavone | -16.1982 | down |
| pmb0624 | 6-C-hexosyl-luteolin O-hexoside | Flavone | 18.1046 | up |
| pmb0628 | Eriodictiol C-hexosyl-O-hexoside | Flavone | 15.5739 | up |
| pmb0645 | 6-C-hexosyl-hesperetin O-hexoside | Flavone | -3.8612 | down |
| pmb0649 | C-hexosyl-luteolin O-hexosyl-O-salicylic acid | Flavone | 18.1420 | up |
| pmb0653 | di-C,C-hexosyl-apigenin | Flavone | 13.2999 | up |
| pmb0657 | C-rhamnosyl-acacetin O-p-coumaroylhexoside | Flavone | 10.0367 | up |
| pmb0660 | C-hexosyl-luteolin O-p-coumaroylhexoside | Flavone | 9.3452 | up |
| pmb0661 | Chrysoeriol C-hexosyl-O-rhamnoside | Flavone | 15.0874 | up |
| pmb0668 | Apigenin C-hexosyl-O-hexosyl-O-Salicylic acid | Flavone | 9.2864 | up |
| pmb0672 | 6-C-hexosyl-apigenin O-feruloylhexoside | Flavone | 10.5641 | up |
| pmb0674 | C-pentosyl apigenin O-salicyloyl hexoside | Flavone | -13.6971 | down |
| pmb0678 | 8-C-hexosyl-apigenin O-feruloylhexoside | Flavone | 16.4730 | up |
| pmb0679 | C-rhamnosyl-apigenin O-feruloylhexoside | Flavone | 12.8048 | up |
| pmb0680 | C-hexosyl-apigenin O-p-coumaroylhexoside | Flavone | 10.6551 | up |
| pmb0682 | 8-C-hexosyl-apigenin O-sinapoylhexoside | Flavone | 19.3869 | up |
| pmb0684 | C-hexosyl-luteolin O-sinapic acid | Flavone | 17.5728 | up |
| pmb0686 | Eriodictyol O-malonylhexoside | Flavanone | 19.0485 | up |
| pmb0696 | 8-C-hexosyl chrysoeriol O-hexoside | Flavone | -14.8529 | down |
| pmb0703 | 6-C-hexosyl-chrysoeriol O-feruloylhexoside | Flavone | 19.3877 | up |
| pmb0711 | Quercetin 7-O-rutinoside | Flavonol | -13.8573 | down |
| pmb0712 | Tricin 5-O-hexosyl-O-hexoside | Flavone | 16.6744 | up |
| pmb0713 | Tricin 7-O-hexosyl-O-hexoside | Flavone | 16.4899 | up |
| pmb0717 | Tricin 5-O-β-guaiacylglycerol | Flavone | -13.8584 | down |
| pmb0725 | Tricin 7-O-feruloylhexoside | Flavone | -11.7670 | down |
| pmb0732 | Tricin 5-O-feruloylhexoside | Flavone | 15.1680 | up |
| pmb0736 | Tricin 7-O-hexoside | Flavone | 16.3889 | up |
| pmb0739 | Tricin O-hexosyl-O-syringin alcohol | Flavone | -11.2221 | down |
| pmb0743 | Tricin 7-O-β-guaiacylglycerol | Flavone | 13.2199 | up |
| pmb0744 | Tricin O-phenylformic acid | Flavone | -12.1310 | down |
| pmb0835 | Gallocatechin-gallocatechin | Polyphenol | -12.9711 | down |
| pmb1108 | Luteolin 6-C-hexoside 8-C-hexosyl-O-hexoside | Flavone | -15.1416 | down |
| pmb2831 | Protocatechuic acid O-glucoside | Polyphenol | -16.0279 | down |
| pmb2961 | Peonidin O-malonylhexoside | Anthocyanins | -13.9131 | down |
| pmb2970 | Hesperetin O-hexosyl-O-hexoside | Flavanone | 11.6455 | up |
| pmb2975 | Hesperetin O-Glucuronic acid | Flavanone | -11.7670 | down |
| pmb2976 | Chrysoeriol C-pentosyl-O-hexosyl-O-hexoside | Flavone | 17.6621 | up |
| pmb2977 | Chrysoeriol 8-C-pentosyl-O-rutinoside | Flavone | -11.1927 | down |
| pmb2983 | Chrysoeriol C-pentosyl-O-rhamnosyl-rhamnoside | Flavone | 10.5507 | up |
| pmb2986 | Chrysoeriol O-hexosyl-O-malonylhexoside | Flavone | -9.8963 | down |
| pmb2991 | Apigenin O-hexosyl-O-rutinoside | Flavone | 18.1622 | up |
| pmb2997 | Chrysoeriol O-hexosyl-O-hexosyl-O-Glucuronic acid | Flavone | 15.7845 | up |
| pmb3006 | Apigenin 7-O-glucoside (Cosmosiin) | Flavone | 9.4721 | up |
| pmb3007 | Chrysoeriol O-glucuronic acid | Flavone | 10.7345 | up |
| pmb3013 | Isorhamnetin O-acetyl-hexoside | Flavonol | 6.6902 | up |
| pmb3019 | Chrysoeriol O-homovanillic acid | Flavone | 13.0027 | up |
| pmb3026 | Quercetin O-acetylhexoside | Flavonol | 12.8388 | up |
| pmb3041 | Tricin O-saccharic acid | Flavone | -14.4445 | down |
| pme0088 | Luteolin | Flavone | 16.9882 | up |
| pme0200 | Kaempferol | Flavonol | 14.6011 | up |
| pme0330 | Naringenin 7-O-neohesperidoside (Naringin) | Flavanone | 11.4777 | up |
| pme0332 | Apigenin 7-O-neohesperidoside (Rhoifolin) | Flavone | 12.1950 | up |
| pme0359 | Apigenin 5-O-glucoside | Flavone | 20.7586 | up |
| pme0363 | Chrysoeriol | Flavone | 8.2503 | up |
| pme0368 | Apigenin 7-rutinoside (Isorhoifolin) | Flavone | 13.0014 | up |
| pme0371 | Naringenin 7-O-glucoside (Prunin) | Flavanone | 19.0014 | up |
| pme0376 | Naringenin | Flavanone | 11.7842 | up |
| pme0379 | Apigenin | Flavone | 7.8856 | up |
| pme1510 | Baicalein (5,6,7-Trihydroxyflavone) | Flavone | 11.0514 | up |
| pme1521 | Dihydroquercetin (Taxifolin) | Flavonol | 13.5684 | up |
| pme1572 | Orobol (5,7,3',4'-tetrahydroxyisoflavone) | Isoflavone | 19.2607 | up |
| pme1578 | Genistein (4',5,7-Trihydroxyisoflavone) | Isoflavone | 17.5935 | up |
| pme1583 | Eriodictyol | Flavanone | 10.3606 | up |
| pme1588 | Isorhamnetin | Flavonol | 15.1767 | up |
| pme1598 | Hesperetin 5-O-glucoside | Flavanone | 5.4662 | up |
| pme1599 | 7-O-Methyleriodictyol | Flavanone | 16.8615 | up |
| pme1611 | Isohemiphloin | Flavone | 20.1622 | up |
| pme1665 | Isovitexin 7-O-glucoside (Saponarin) | Flavonoid | 20.0401 | up |
| pme1786 | Malvidin 3,5-diglucoside (Malvin) | Anthocyanins | -13.1256 | down |
| pme2296 | Curcumin | Polyphenol | 10.3144 | up |
| pme2319 | Hesperetin | Flavanone | 14.1655 | up |
| pme2459 | Luteolin 7-O-glucoside (Cynaroside) | Flavone | 20.1839 | up |
| pme2957 | Naringenin chalcone | Flavanone | 11.6853 | up |
| pme2963 | Aromadedrin (Dihydrokaempferol) | Flavonol | 20.1572 | up |
| pme2973 | Icariin (kaempferol 3,7-O-diglucoside 8-prenyl derivative) | Flavonol | -10.9443 | down |
| pme3129 | Quercetin 4'-O-glucoside (Spiraeoside) | Flavonol | 19.6216 | up |
| pme3209 | Genistein 7-O-Glucoside (Genistin) | Isoflavone | 16.5881 | up |
| pme3215 | Isoliquiritigenin | Flavanone | 13.6580 | up |
| pme3230 | Calycosin | Isoflavone | 16.0525 | up |
| pme3250 | Biochanin A | Isoflavone | 9.7275 | up |
| pme3256 | Delphinidin 3-O-rutinoside (Tulipanin) | Anthocyanins | -11.9644 | down |
| pme3261 | 6-Hydroxydaidzein | Isoflavone | 16.9337 | up |
| pme3267 | Kaempferol 3-O-galactoside (Trifolin) | Flavonol | 17.9695 | up |
| pme3276 | 2'-Hydroxygenistein | Isoflavone | 15.9053 | up |
| pme3300 | Tricetin | Flavone | 9.5390 | up |
| pme3369 | Rhamnetin (7-O-methxyl quercetin) | Flavonol | 9.7771 | up |
| pme3391 | Petunidin 3-O-glucoside | Anthocyanins | -16.7501 | down |
| pme3392 | Pelargonidin 3-O-beta-D-glucoside（Callistephin chloride) | Anthocyanins | 16.1178 | up |
| pme3401 | Syringetin | Flavonol | 12.4944 | up |
| pme3407 | Laricitrin | Flavonol | 11.4870 | up |
| pme3440 | Butein | Flavanone | 16.4313 | up |
| pme3461 | Homoeriodictyol | Flavanone | 21.1332 | up |
| pme3473 | Butin | Flavone | 11.1986 | up |
| pme3502 | Formononetin 7-O-glucoside (Ononin) | Isoflavone | -13.4569 | down |
| pmf0005 | Narirutin | Flavone | 11.5183 | up |
| pmf0057 | 4,2',4',6'-Tetrahydroxychalcone | Flavone | 11.4338 | up |
| pmf0058 | 4',5,7-Trihydroxyflavanone | Flavanone | 10.9884 | up |
| pmf0108 | Garbanzol | Flavanone | 11.5364 | up |
| pmf0279 | Gossypitrin | Flavonoid | -14.7655 | down |
| pmf0301 | Engeletin | Flavonoid | 14.1709 | up |
| pmf0361 | Astilbin | Flavonoid | -16.2179 | down |
| pmf0369 | Persicoside | Flavonoid | -11.5614 | down |
| pmf0393 | Persicogenin | Flavonoid | 10.7284 | up |
| pmf0568 | Tectorigenin | Flavonoid | 11.6304 | up |
| pmf0582 | Glabridin | Flavonoid | -10.2570 | down |
| pmf0618 | Cyanidin 3-p-hydroxybenzoylsophoroside-5-glucoside | Anthocyanins | -10.6670 | down |
