# Supplementary material for: Comprehensive Analysis of Transcriptome and Metabolome Reveals the Flavonoid Metabolic Pathway Is Associated with Fruit Peel Coloration of Melon
Source: Molecules. 2021 May 10;26(9):2830. doi: 10.3390/molecules26092830 (PMC8126211; doi:10.3390/molecules26092830)
Supplement: Supplementary file 1 [file molecules-26-02830-s001.zip › molecules-1183709-supplementary/Table S11-Pearson correlation (r2) between metabolites and genes.docx]

| **Table S11-Pearson correlation (r**2) between metabolites and genes | | | | |
| --- | --- | --- | --- | --- |
| **ID** | **Compounds** | **Class** | **PCC** | R2 |
| MELO3C035535.2 | Apigenin 7-O-glucoside (Cosmosiin) | Flavone | 0.991 | 0.982 |
| MELO3C035535.2 | Luteolin | Flavone | 0.992 | 0.984 |
| MELO3C035535.2 | Apigenin 7-O-neohesperidoside (Rhoifolin) | Flavone | 0.992 | 0.984 |
| MELO3C035535.2 | Isovitexin | Flavone | 0.993 | 0.986 |
| MELO3C035535.2 | Tricetin | Flavone | 0.987 | 0.974 |
| MELO3C035535.2 | Kaempferide | Flavonol | 0.992 | 0.984 |
| MELO3C035535.2 | Kaempferol | Flavonol | 0.992 | 0.984 |
| MELO3C035535.2 | Dihydroquercetin (Taxifolin) | Flavonol | 0.992 | 0.984 |
| MELO3C035535.2 | Aromadedrin (Dihydrokaempferol) | Flavonol | 0.992 | 0.984 |
| MELO3C035535.2 | Kaempferol 3-O-galactoside (Trifolin) | Flavonol | 0.992 | 0.984 |
| MELO3C035535.2 | Laricitrin | Flavonol | 0.992 | 0.984 |
| MELO3C035535.2 | Naringenin 7-O-neohesperidoside (Naringin) | Flavanone | 0.991 | 0.982 |
| MELO3C035535.2 | Naringenin 7-O-glucoside (Prunin) | Flavanone | 0.992 | 0.984 |
| MELO3C035535.2 | Hesperetin | Flavanone | 0.959 | 0.920 |
| MELO3C035535.2 | Isoliquiritigenin | Flavanone | 0.992 | 0.984 |
| MELO3C035535.2 | Butein | Flavanone | 0.992 | 0.984 |
| MELO3C035535.2 | 4',5,7-Trihydroxyflavanone | Flavanone | 0.98 | 0.960 |
| MELO3C035535.2 | Genistein (4',5,7-Trihydroxyisoflavone) | Isoflavone | 0.992 | 0.984 |
| MELO3C035535.2 | Genistein 7-O-Glucoside (Genistin) | Isoflavone | 0.992 | 0.984 |
| MELO3C035535.2 | Calycosin | Isoflavone | 0.992 | 0.984 |
| MELO3C035535.2 | 6-Hydroxydaidzein | Isoflavone | 0.992 | 0.984 |
| MELO3C035535.2 | 2'-Hydroxygenistein | Isoflavone | 0.992 | 0.984 |
| MELO3C035535.2 | Formononetin 7-O-glucoside (Ononin) | Isoflavone | -0.988 | 0.976 |
| MELO3C035535.2 | Delphinidin 3-O-rutinoside (Tulipanin) | Anthocyanins | -0.994 | 0.988 |
| MELO3C035535.2 | Petunidin 3-O-glucoside | Anthocyanins | -0.989 | 0.978 |
| MELO3C017219.2 | Apigenin 7-O-glucoside (Cosmosiin) | Flavone | 0.967 | 0.935 |
| MELO3C017219.2 | Luteolin | Flavone | 0.959 | 0.920 |
| MELO3C017219.2 | Apigenin 7-O-neohesperidoside (Rhoifolin) | Flavone | 0.958 | 0.918 |
| MELO3C017219.2 | Isovitexin | Flavone | 0.975 | 0.951 |
| MELO3C017219.2 | Tricetin | Flavone | 0.961 | 0.924 |
| MELO3C017219.2 | Kaempferide | Flavonol | 0.959 | 0.920 |
| MELO3C017219.2 | Kaempferol | Flavonol | 0.962 | 0.925 |
| MELO3C017219.2 | Dihydroquercetin (Taxifolin) | Flavonol | 0.958 | 0.918 |
| MELO3C017219.2 | Aromadedrin (Dihydrokaempferol) | Flavonol | 0.96 | 0.922 |
| MELO3C017219.2 | Kaempferol 3-O-galactoside (Trifolin) | Flavonol | 0.956 | 0.914 |
| MELO3C017219.2 | Laricitrin | Flavonol | 0.958 | 0.918 |
| MELO3C017219.2 | Naringenin 7-O-neohesperidoside (Naringin) | Flavanone | 0.956 | 0.914 |
| MELO3C017219.2 | Naringenin 7-O-glucoside (Prunin) | Flavanone | 0.959 | 0.920 |
| MELO3C017219.2 | Isoliquiritigenin | Flavanone | 0.96 | 0.922 |
| MELO3C017219.2 | Butein | Flavanone | 0.959 | 0.920 |
| MELO3C017219.2 | 4',5,7-Trihydroxyflavanone | Flavanone | 0.95 | 0.903 |
| MELO3C017219.2 | Genistein (4',5,7-Trihydroxyisoflavone) | Isoflavone | 0.959 | 0.920 |
| MELO3C017219.2 | Genistein 7-O-Glucoside (Genistin) | Isoflavone | 0.958 | 0.918 |
| MELO3C017219.2 | Calycosin | Isoflavone | 0.96 | 0.922 |
| MELO3C017219.2 | 6-Hydroxydaidzein | Isoflavone | 0.959 | 0.920 |
| MELO3C017219.2 | 2'-Hydroxygenistein | Isoflavone | 0.958 | 0.918 |
| MELO3C017219.2 | Formononetin 7-O-glucoside (Ononin) | Isoflavone | -0.957 | 0.916 |
| MELO3C017219.2 | Delphinidin 3-O-rutinoside (Tulipanin) | Anthocyanins | -0.955 | 0.912 |
| MELO3C017219.2 | Petunidin 3-O-glucoside | Anthocyanins | -0.969 | 0.939 |
| MELO3C005571.2 | Apigenin 7-O-glucoside (Cosmosiin) | Flavone | 0.977 | 0.955 |
| MELO3C005571.2 | Luteolin | Flavone | 0.968 | 0.937 |
| MELO3C005571.2 | Apigenin 7-O-neohesperidoside (Rhoifolin) | Flavone | 0.967 | 0.935 |
| MELO3C005571.2 | Isovitexin | Flavone | 0.958 | 0.918 |
| MELO3C005571.2 | Tricetin | Flavone | 0.976 | 0.953 |
| MELO3C005571.2 | Kaempferide | Flavonol | 0.968 | 0.937 |
| MELO3C005571.2 | Kaempferol | Flavonol | 0.969 | 0.939 |
| MELO3C005571.2 | Dihydroquercetin (Taxifolin) | Flavonol | 0.968 | 0.937 |
| MELO3C005571.2 | Aromadedrin (Dihydrokaempferol) | Flavonol | 0.968 | 0.937 |
| MELO3C005571.2 | Kaempferol 3-O-galactoside (Trifolin) | Flavonol | 0.967 | 0.935 |
| MELO3C005571.2 | Laricitrin | Flavonol | 0.968 | 0.937 |
| MELO3C005571.2 | Naringenin 7-O-neohesperidoside (Naringin) | Flavanone | 0.967 | 0.935 |
| MELO3C005571.2 | Naringenin 7-O-glucoside (Prunin) | Flavanone | 0.968 | 0.937 |
| MELO3C005571.2 | Isoliquiritigenin | Flavanone | 0.968 | 0.937 |
| MELO3C005571.2 | Butein | Flavanone | 0.968 | 0.937 |
| MELO3C005571.2 | Homoeriodictyol | Flavanone | 0.967 | 0.935 |
| MELO3C005571.2 | 4',5,7-Trihydroxyflavanone | Flavanone | 0.958 | 0.918 |
| MELO3C005571.2 | Genistein (4',5,7-Trihydroxyisoflavone) | Isoflavone | 0.968 | 0.937 |
| MELO3C005571.2 | Genistein 7-O-Glucoside (Genistin) | Isoflavone | 0.968 | 0.937 |
| MELO3C005571.2 | Calycosin | Isoflavone | 0.968 | 0.937 |
| MELO3C005571.2 | 6-Hydroxydaidzein | Isoflavone | 0.968 | 0.937 |
| MELO3C005571.2 | 2'-Hydroxygenistein | Isoflavone | 0.968 | 0.937 |
| MELO3C005571.2 | Formononetin 7-O-glucoside (Ononin) | Isoflavone | -0.961 | 0.924 |
| MELO3C005571.2 | Delphinidin 3-O-rutinoside (Tulipanin) | Anthocyanins | -0.966 | 0.933 |
| MELO3C005571.2 | Petunidin 3-O-glucoside | Anthocyanins | -0.964 | 0.929 |
| MELO3C009387.2 | Apigenin 7-O-glucoside (Cosmosiin) | Flavone | -0.977 | 0.955 |
| MELO3C009387.2 | Luteolin | Flavone | -0.964 | 0.929 |
| MELO3C009387.2 | Apigenin 7-O-neohesperidoside (Rhoifolin) | Flavone | -0.965 | 0.931 |
| MELO3C009387.2 | Isovitexin | Flavone | -0.96 | 0.922 |
| MELO3C009387.2 | Tricetin | Flavone | -0.966 | 0.933 |
| MELO3C009387.2 | Kaempferide | Flavonol | -0.964 | 0.929 |
| MELO3C009387.2 | Kaempferol | Flavonol | -0.959 | 0.920 |
| MELO3C009387.2 | Dihydroquercetin (Taxifolin) | Flavonol | -0.965 | 0.931 |
| MELO3C009387.2 | Aromadedrin (Dihydrokaempferol) | Flavonol | -0.963 | 0.927 |
| MELO3C009387.2 | Kaempferol 3-O-galactoside (Trifolin) | Flavonol | -0.967 | 0.935 |
| MELO3C009387.2 | Laricitrin | Flavonol | -0.964 | 0.929 |
| MELO3C009387.2 | Naringenin 7-O-neohesperidoside (Naringin) | Flavanone | -0.966 | 0.933 |
| MELO3C009387.2 | Naringenin 7-O-glucoside (Prunin) | Flavanone | -0.964 | 0.929 |
| MELO3C009387.2 | Isoliquiritigenin | Flavanone | -0.962 | 0.925 |
| MELO3C009387.2 | Butein | Flavanone | -0.963 | 0.927 |
| MELO3C009387.2 | 4',5,7-Trihydroxyflavanone | Flavanone | -0.952 | 0.906 |
| MELO3C009387.2 | Genistein (4',5,7-Trihydroxyisoflavone) | Isoflavone | -0.963 | 0.927 |
| MELO3C009387.2 | Genistein 7-O-Glucoside (Genistin) | Isoflavone | -0.965 | 0.931 |
| MELO3C009387.2 | Calycosin | Isoflavone | -0.962 | 0.925 |
| MELO3C009387.2 | 6-Hydroxydaidzein | Isoflavone | -0.963 | 0.927 |
| MELO3C009387.2 | 2'-Hydroxygenistein | Isoflavone | -0.964 | 0.929 |
| MELO3C009387.2 | Formononetin 7-O-glucoside (Ononin) | Isoflavone | 0.954 | 0.910 |
| MELO3C009387.2 | Delphinidin 3-O-rutinoside (Tulipanin) | Anthocyanins | 0.965 | 0.931 |
| MELO3C009387.2 | Petunidin 3-O-glucoside | Anthocyanins | 0.956 | 0.914 |
| MELO3C014584.2 | Apigenin 7-O-glucoside (Cosmosiin) | Flavone | -0.96 | 0.922 |
| MELO3C014584.2 | Luteolin | Flavone | -0.964 | 0.929 |
| MELO3C014584.2 | Apigenin 7-O-neohesperidoside (Rhoifolin) | Flavone | -0.962 | 0.925 |
| MELO3C014584.2 | Isovitexin | Flavone | -0.962 | 0.925 |
| MELO3C014584.2 | Tricetin | Flavone | -0.967 | 0.935 |
| MELO3C014584.2 | Kaempferide | Flavonol | -0.963 | 0.927 |
| MELO3C014584.2 | Kaempferol | Flavonol | -0.969 | 0.939 |
| MELO3C014584.2 | Dihydroquercetin (Taxifolin) | Flavonol | -0.963 | 0.927 |
| MELO3C014584.2 | Aromadedrin (Dihydrokaempferol) | Flavonol | -0.964 | 0.929 |
| MELO3C014584.2 | Kaempferol 3-O-galactoside (Trifolin) | Flavonol | -0.96 | 0.922 |
| MELO3C014584.2 | Laricitrin | Flavonol | -0.963 | 0.927 |
| MELO3C014584.2 | Naringenin 7-O-neohesperidoside (Naringin) | Flavanone | -0.96 | 0.922 |
| MELO3C014584.2 | Naringenin 7-O-glucoside (Prunin) | Flavanone | -0.964 | 0.929 |
| MELO3C014584.2 | Isoliquiritigenin | Flavanone | -0.966 | 0.933 |
| MELO3C014584.2 | Butein | Flavanone | -0.964 | 0.929 |
| MELO3C014584.2 | Homoeriodictyol | Flavanone | -0.953 | 0.908 |
| MELO3C014584.2 | Genistein (4',5,7-Trihydroxyisoflavone) | Isoflavone | -0.964 | 0.929 |
| MELO3C014584.2 | Genistein 7-O-Glucoside (Genistin) | Isoflavone | -0.962 | 0.925 |
| MELO3C014584.2 | Calycosin | Isoflavone | -0.965 | 0.931 |
| MELO3C014584.2 | 6-Hydroxydaidzein | Isoflavone | -0.964 | 0.929 |
| MELO3C014584.2 | 2'-Hydroxygenistein | Isoflavone | -0.963 | 0.927 |
| MELO3C014584.2 | Formononetin 7-O-glucoside (Ononin) | Isoflavone | 0.965 | 0.931 |
| MELO3C014584.2 | Delphinidin 3-O-rutinoside (Tulipanin) | Anthocyanins | 0.961 | 0.924 |
| MELO3C014584.2 | Petunidin 3-O-glucoside | Anthocyanins | 0.969 | 0.939 |
| MELO3C035771.2 | Apigenin 7-O-glucoside (Cosmosiin) | Flavone | 0.951 | 0.904 |
| MELO3C035771.2 | Luteolin | Flavone | 0.959 | 0.920 |
| MELO3C035771.2 | Apigenin 7-O-neohesperidoside (Rhoifolin) | Flavone | 0.959 | 0.920 |
| MELO3C035771.2 | Tricetin | Flavone | 0.964 | 0.929 |
| MELO3C035771.2 | Kaempferide | Flavonol | 0.959 | 0.920 |
| MELO3C035771.2 | Kaempferol | Flavonol | 0.959 | 0.920 |
| MELO3C035771.2 | Dihydroquercetin (Taxifolin) | Flavonol | 0.959 | 0.920 |
| MELO3C035771.2 | Aromadedrin (Dihydrokaempferol) | Flavonol | 0.959 | 0.920 |
| MELO3C035771.2 | Kaempferol 3-O-galactoside (Trifolin) | Flavonol | 0.959 | 0.920 |
| MELO3C035771.2 | Laricitrin | Flavonol | 0.959 | 0.920 |
| MELO3C035771.2 | Naringenin 7-O-neohesperidoside (Naringin) | Flavanone | 0.959 | 0.920 |
| MELO3C035771.2 | Naringenin 7-O-glucoside (Prunin) | Flavanone | 0.959 | 0.920 |
| MELO3C035771.2 | Isoliquiritigenin | Flavanone | 0.959 | 0.920 |
| MELO3C035771.2 | Butein | Flavanone | 0.959 | 0.920 |
| MELO3C035771.2 | Homoeriodictyol | Flavanone | 1 | 1.000 |
| MELO3C035771.2 | Genistein (4',5,7-Trihydroxyisoflavone) | Isoflavone | 0.959 | 0.920 |
| MELO3C035771.2 | Genistein 7-O-Glucoside (Genistin) | Isoflavone | 0.959 | 0.920 |
| MELO3C035771.2 | Calycosin | Isoflavone | 0.959 | 0.920 |
| MELO3C035771.2 | 6-Hydroxydaidzein | Isoflavone | 0.959 | 0.920 |
| MELO3C035771.2 | 2'-Hydroxygenistein | Isoflavone | 0.959 | 0.920 |
| MELO3C035771.2 | Formononetin 7-O-glucoside (Ononin) | Isoflavone | -0.967 | 0.935 |
| MELO3C035771.2 | Delphinidin 3-O-rutinoside (Tulipanin) | Anthocyanins | -0.955 | 0.912 |
| MELO3C035771.2 | Petunidin 3-O-glucoside | Anthocyanins | -0.963 | 0.927 |
