# Supplementary material for: Comprehensive Analysis of Transcriptome and Metabolome Reveals the Flavonoid Metabolic Pathway Is Associated with Fruit Peel Coloration of Melon
Source: Molecules. 2021 May 10;26(9):2830. doi: 10.3390/molecules26092830 (PMC8126211; doi:10.3390/molecules26092830)
Supplement: Supplementary file 1 [file molecules-26-02830-s001.zip › molecules-1183709-supplementary/Table S2 FPKMs and function annotation of genes in W vs B .docx]

| **Table S2 FPKMs and function annotation of genes in W vs B** | | | | | |
| --- | --- | --- | --- | --- | --- |
| **ID** | **W** | **B** | **log2FoldChange** | **pvalue** | **regulated** |
| MELO3C000062.2 | 171 | 397 | 1.213 | 2.29903E-06 | up |
| MELO3C000064.2 | 219 | 85 | -1.364 | 3.78318E-05 | down |
| MELO3C000147.2 | 163 | 32 | -2.376 | 7.61242E-14 | down |
| MELO3C000204.2 | 225 | 85 | -1.404 | 2.715E-11 | down |
| MELO3C000298.2 | 697 | 312 | -1.16 | 1.81085E-11 | down |
| MELO3C000391.2 | 137 | 27 | -2.343 | 1.03034E-07 | down |
| MELO3C000398.2 | 67 | 24 | -1.471 | 0.000481986 | down |
| MELO3C000487.2 | 17 | 92 | 2.424 | 4.49699E-06 | up |
| MELO3C000631.2 | 9 | 49 | 2.469 | 0.000185438 | up |
| MELO3C000781.2 | 7 | 30 | 2.023 | 0.001185082 | up |
| MELO3C000794.2 | 8 | 68 | 3.147 | 3.86299E-10 | up |
| MELO3C001419.2 | 109 | 25 | -2.148 | 4.78642E-10 | down |
| MELO3C001656.2 | 467 | 1768 | 1.922 | 4.86611E-37 | up |
| MELO3C002049.2 | 46 | 14 | -1.746 | 0.004097079 | down |
| MELO3C002084.2 | 244 | 542 | 1.151 | 4.06375E-08 | up |
| MELO3C002122.2 | 47 | 152 | 1.71 | 8.43319E-08 | up |
| MELO3C002140.2 | 83 | 348 | 2.066 | 1.46634E-18 | up |
| MELO3C002185.2 | 21 | 49 | 1.205 | 0.006162647 | up |
| MELO3C002210.2 | 33 | 95 | 1.519 | 0.00097976 | up |
| MELO3C002220.2 | 295 | 798 | 1.437 | 2.0596E-15 | up |
| MELO3C002244.2 | 70 | 34 | -1.034 | 0.00226342 | down |
| MELO3C002309.2 | 18 | 42 | 1.192 | 0.007570325 | up |
| MELO3C002310.2 | 33690 | 14056 | -1.261 | 1.42821E-05 | down |
| MELO3C002318.2 | 809 | 271 | -1.58 | 1.43892E-14 | down |
| MELO3C002385.2 | 89 | 246 | 1.458 | 1.75999E-11 | up |
| MELO3C002393.2 | 30 | 79 | 1.382 | 0.000248967 | up |
| MELO3C002483.2 | 171 | 76 | -1.17 | 0.00035 | down |
| MELO3C002511.2 | 174 | 383 | 1.138 | 9.90946E-05 | up |
| MELO3C002546.2 | 56 | 14 | -1.984 | 1.59337E-05 | down |
| MELO3C002572.2 | 64 | 328 | 2.368 | 1.26533E-12 | up |
| MELO3C002598.2 | 10982 | 5232 | -1.07 | 5.67431E-14 | down |
| MELO3C002617.2 | 77 | 161 | 1.062 | 0.003737431 | up |
| MELO3C002637.2 | 23 | 75 | 1.679 | 2.56878E-06 | up |
| MELO3C002674.2 | 634 | 284 | -1.158 | 6.5559E-16 | down |
| MELO3C002689.2 | 245 | 536 | 1.131 | 1.62511E-09 | up |
| MELO3C002726.2 | 832 | 1779 | 1.097 | 4.91305E-09 | up |
| MELO3C002777.2 | 1 | 54 | 6.415 | 9.5794E-09 | up |
| MELO3C003002.2 | 46 | 106 | 1.194 | 0.00065378 | up |
| MELO3C003148.2 | 220 | 77 | -1.511 | 1.67916E-08 | down |
| MELO3C003177.2 | 40 | 15 | -1.377 | 0.011042518 | down |
| MELO3C003188.2 | 3 | 34 | 3.426 | 0.002786878 | up |
| MELO3C003371.2 | 74 | 332 | 2.156 | 2.36023E-20 | up |
| MELO3C003374.2 | 304 | 1061 | 1.803 | 5.73553E-36 | up |
| MELO3C003375.2 | 41549 | 111297 | 1.422 | 3.06265E-69 | up |
| MELO3C003384.2 | 724 | 349 | -1.052 | 9.10538E-08 | down |
| MELO3C003385.2 | 41 | 13 | -1.7 | 0.000917914 | down |
| MELO3C003390.2 | 150 | 68 | -1.135 | 1.31341E-05 | down |
| MELO3C003403.2 | 158 | 62 | -1.354 | 2.46185E-07 | down |
| MELO3C003405.2 | 228 | 108 | -1.077 | 1.41207E-07 | down |
| MELO3C003469.2 | 170 | 345 | 1.025 | 1.26714E-05 | up |
| MELO3C003526.2 | 23 | 70 | 1.609 | 0.000516993 | up |
| MELO3C003559.2 | 374 | 1073 | 1.521 | 0.001033592 | up |
| MELO3C003585.2 | 60 | 233 | 1.958 | 1.58823E-13 | up |
| MELO3C003644.2 | 1621 | 789 | -1.039 | 0.005618273 | down |
| MELO3C003676.2 | 104 | 216 | 1.056 | 2.01963E-07 | up |
| MELO3C003686.2 | 1742 | 534 | -1.705 | 8.3038E-07 | down |
| MELO3C003698.2 | 109 | 41 | -1.394 | 5.57141E-05 | down |
| MELO3C003726.2 | 45 | 110 | 1.293 | 5.92237E-06 | up |
| MELO3C003753.2 | 69 | 33 | -1.086 | 0.001905803 | down |
| MELO3C003792.2 | 77 | 169 | 1.137 | 0.007971606 | up |
| MELO3C003813.2 | 305 | 884 | 1.536 | 2.24558E-12 | up |
| MELO3C003879.2 | 32 | 66 | 1.079 | 0.00217246 | up |
| MELO3C003990.2 | 1930 | 7807 | 2.016 | 2.31801E-30 | up |
| MELO3C003992.2 | 45 | 181 | 1.991 | 6.01755E-09 | up |
| MELO3C004054.2 | 12 | 60 | 2.346 | 0.003263914 | up |
| MELO3C004155.2 | 39 | 15 | -1.41 | 0.002307108 | down |
| MELO3C004195.2 | 131 | 64 | -1.03 | 0.001551116 | down |
| MELO3C004244.2 | 566 | 2029 | 1.843 | 0.000687176 | up |
| MELO3C004247.2 | 9 | 62 | 2.746 | 8.56032E-06 | up |
| MELO3C004262.2 | 162 | 77 | -1.062 | 9.55149E-06 | down |
| MELO3C004272.2 | 12 | 34 | 1.542 | 0.001452812 | up |
| MELO3C004283.2 | 66 | 32 | -1.026 | 0.008059646 | down |
| MELO3C004314.2 | 136 | 62 | -1.13 | 8.05642E-06 | down |
| MELO3C004315.2 | 46 | 16 | -1.528 | 0.001600432 | down |
| MELO3C004338.2 | 1135 | 541 | -1.068 | 7.10448E-12 | down |
| MELO3C004382.2 | 373 | 773 | 1.052 | 9.31181E-13 | up |
| MELO3C004404.2 | 26 | 98 | 1.941 | 7.14476E-09 | up |
| MELO3C004520.2 | 285 | 107 | -1.419 | 2.82316E-07 | down |
| MELO3C004536.2 | 78 | 184 | 1.237 | 9.57979E-08 | up |
| MELO3C004554.2 | 70 | 32 | -1.137 | 0.010034633 | down |
| MELO3C004586.2 | 70 | 22 | -1.651 | 7.99188E-06 | down |
| MELO3C004602.2 | 92 | 43 | -1.089 | 0.001565967 | down |
| MELO3C004624.2 | 131 | 291 | 1.156 | 1.67694E-06 | up |
| MELO3C004633.2 | 257 | 855 | 1.733 | 0.000103261 | up |
| MELO3C004647.2 | 72 | 148 | 1.032 | 0.000393789 | up |
| MELO3C004719.2 | 41 | 18 | -1.189 | 0.010913324 | down |
| MELO3C004974.2 | 9 | 32 | 1.847 | 0.008374246 | up |
| MELO3C005001.2 | 77 | 33 | -1.234 | 0.000258491 | down |
| MELO3C005173.2 | 1127 | 552 | -1.028 | 1.07012E-09 | down |
| MELO3C005258.2 | 93 | 240 | 1.362 | 4.01043E-07 | up |
| MELO3C005403.2 | 36 | 99 | 1.451 | 5.01472E-07 | up |
| MELO3C005435.2 | 24 | 201 | 3.076 | 3.20224E-23 | up |
| MELO3C005492.2 | 73 | 148 | 1.021 | 6.98868E-05 | up |
| MELO3C005574.2 | 33 | 69 | 1.052 | 0.001736429 | up |
| MELO3C005583.2 | 389 | 181 | -1.103 | 1.79445E-07 | down |
| MELO3C005601.2 | 76 | 194 | 1.35 | 1.12774E-06 | up |
| MELO3C005695.2 | 51 | 10 | -2.327 | 2.9141E-05 | down |
| MELO3C005748.2 | 49 | 120 | 1.286 | 0.001426003 | up |
| MELO3C005752.2 | 57 | 13 | -2.168 | 0.00299649 | down |
| MELO3C005787.2 | 1086 | 386 | -1.489 | 3.99601E-30 | down |
| MELO3C005801.2 | 385 | 856 | 1.152 | 0.003953006 | up |
| MELO3C005832.2 | 47 | 148 | 1.642 | 1.88719E-05 | up |
| MELO3C005885.2 | 32 | 74 | 1.227 | 0.006842196 | up |
| MELO3C005947.2 | 51 | 15 | -1.727 | 0.000110873 | down |
| MELO3C005978.2 | 20 | 66 | 1.735 | 0.001450247 | up |
| MELO3C006253.2 | 47478 | 21025 | -1.175 | 6.37025E-23 | down |
| MELO3C006254.2 | 24295 | 6780 | -1.841 | 6.21778E-08 | down |
| MELO3C006335.2 | 472 | 944 | 1 | 1.32213E-19 | up |
| MELO3C006482.2 | 211 | 793 | 1.91 | 2.14262E-15 | up |
| MELO3C006503.2 | 48 | 124 | 1.372 | 0.000221706 | up |
| MELO3C006561.2 | 107 | 51 | -1.052 | 0.000219809 | down |
| MELO3C006583.2 | 1364 | 397 | -1.78 | 2.15262E-05 | down |
| MELO3C006621.2 | 196 | 74 | -1.403 | 5.99274E-07 | down |
| MELO3C006678.2 | 457 | 84 | -2.442 | 4.37222E-24 | down |
| MELO3C006700.2 | 100 | 16 | -2.67 | 0.000105861 | down |
| MELO3C006716.2 | 143 | 345 | 1.271 | 1.30159E-15 | up |
| MELO3C006717.2 | 3961 | 8269 | 1.062 | 2.50534E-31 | up |
| MELO3C006810.2 | 131 | 300 | 1.197 | 8.15478E-07 | up |
| MELO3C006944.2 | 39 | 255 | 2.706 | 3.575E-22 | up |
| MELO3C006965.2 | 96 | 30 | -1.683 | 2.34019E-05 | down |
| MELO3C006981.2 | 20 | 47 | 1.224 | 0.004323751 | up |
| MELO3C007102.2 | 26 | 63 | 1.291 | 0.000605044 | up |
| MELO3C007132.2 | 34 | 14 | -1.295 | 0.008085275 | down |
| MELO3C007253.2 | 48 | 20 | -1.254 | 0.008047339 | down |
| MELO3C007425.2 | 36 | 4 | -3.022 | 0.000175862 | down |
| MELO3C007531.2 | 183 | 71 | -1.365 | 5.5723E-09 | down |
| MELO3C007545.2 | 37 | 115 | 1.631 | 1.11027E-06 | up |
| MELO3C007562.2 | 3550 | 845 | -2.07 | 1.24064E-34 | down |
| MELO3C007563.2 | 351 | 100 | -1.802 | 8.13511E-18 | down |
| MELO3C007687.2 | 55 | 9 | -2.624 | 7.09989E-07 | down |
| MELO3C007702.2 | 124 | 258 | 1.055 | 0.000339971 | up |
| MELO3C007731.2 | 2 | 41 | 4.401 | 6.3865E-08 | up |
| MELO3C007774.2 | 13 | 78 | 2.573 | 0.00033502 | up |
| MELO3C007793.2 | 98 | 710 | 2.861 | 2.81284E-22 | up |
| MELO3C007794.2 | 391 | 133 | -1.554 | 3.12856E-07 | down |
| MELO3C007798.2 | 741 | 308 | -1.265 | 8.30517E-17 | down |
| MELO3C007845.2 | 200 | 87 | -1.209 | 4.26566E-07 | down |
| MELO3C007862.2 | 61 | 136 | 1.151 | 0.000717655 | up |
| MELO3C007872.2 | 70 | 169 | 1.279 | 1.81499E-05 | up |
| MELO3C007878.2 | 277 | 110 | -1.336 | 7.10552E-12 | down |
| MELO3C007884.2 | 2416 | 763 | -1.664 | 0.002144472 | down |
| MELO3C007901.2 | 61 | 23 | -1.43 | 0.000229173 | down |
| MELO3C007928.2 | 147 | 432 | 1.557 | 9.03277E-09 | up |
| MELO3C007967.2 | 76 | 168 | 1.14 | 5.5033E-06 | up |
| MELO3C007976.2 | 42 | 86 | 1.041 | 0.004994941 | up |
| MELO3C007979.2 | 215 | 522 | 1.275 | 1.0092E-19 | up |
| MELO3C008006.2 | 62 | 30 | -1.085 | 0.00162069 | down |
| MELO3C008152.2 | 285 | 53 | -2.413 | 8.10077E-30 | down |
| MELO3C008203.2 | 112 | 39 | -1.537 | 1.57778E-06 | down |
| MELO3C008263.2 | 46 | 8 | -2.439 | 5.67599E-05 | down |
| MELO3C008322.2 | 318 | 138 | -1.198 | 1.02558E-05 | down |
| MELO3C008386.2 | 23 | 48 | 1.08 | 0.007274698 | up |
| MELO3C008466.2 | 27 | 7 | -2.034 | 0.002033245 | down |
| MELO3C008581.2 | 34 | 76 | 1.186 | 0.000579206 | up |
| MELO3C008657.2 | 161 | 415 | 1.365 | 4.2303E-05 | up |
| MELO3C009097.2 | 1274 | 3005 | 1.238 | 1.58013E-10 | up |
| MELO3C009182.2 | 42 | 150 | 1.826 | 4.08674E-06 | up |
| MELO3C009212.2 | 67 | 27 | -1.317 | 0.000211008 | down |
| MELO3C009288.2 | 1028 | 445 | -1.206 | 1.59877E-11 | down |
| MELO3C009339.2 | 2175 | 999 | -1.121 | 9.44649E-24 | down |
| MELO3C009422.2 | 1515 | 704 | -1.106 | 1.92761E-10 | down |
| MELO3C009444.2 | 31 | 86 | 1.504 | 1.21663E-05 | up |
| MELO3C009445.2 | 4308 | 11294 | 1.391 | 5.87509E-57 | up |
| MELO3C009454.2 | 1066 | 2189 | 1.037 | 7.3891E-18 | up |
| MELO3C009461.2 | 12 | 44 | 1.868 | 0.000113802 | up |
| MELO3C009506.2 | 557 | 152 | -1.875 | 6.18089E-18 | down |
| MELO3C009637.2 | 33 | 121 | 1.886 | 2.16955E-06 | up |
| MELO3C009641.2 | 10 | 38 | 1.978 | 0.000812065 | up |
| MELO3C009720.2 | 24 | 6 | -2.021 | 0.005045761 | down |
| MELO3C009739.2 | 123 | 45 | -1.44 | 0.000277462 | down |
| MELO3C009755.2 | 553 | 1125 | 1.025 | 1.63436E-05 | up |
| MELO3C009758.2 | 116 | 237 | 1.031 | 0.000262085 | up |
| MELO3C009849.2 | 31 | 81 | 1.384 | 0.000402186 | up |
| MELO3C009856.2 | 56 | 7 | -3.099 | 7.57784E-07 | down |
| MELO3C009872.2 | 28 | 80 | 1.52 | 0.000531078 | up |
| MELO3C009900.2 | 80 | 27 | -1.589 | 0.000272216 | down |
| MELO3C009918.2 | 73 | 213 | 1.546 | 1.92202E-12 | up |
| MELO3C010162.2 | 1171 | 302 | -1.956 | 2.16203E-37 | down |
| MELO3C010170.2 | 74 | 398 | 2.437 | 9.18665E-17 | up |
| MELO3C010197.2 | 2456 | 6888 | 1.488 | 2.23833E-19 | up |
| MELO3C010233.2 | 19 | 50 | 1.386 | 0.000682028 | up |
| MELO3C010272.2 | 298 | 2162 | 2.859 | 1.23053E-59 | up |
| MELO3C010323.2 | 1370 | 633 | -1.113 | 1.78464E-16 | down |
| MELO3C010458.2 | 30 | 62 | 1.035 | 0.002575646 | up |
| MELO3C010493.2 | 246 | 493 | 1.002 | 5.19597E-07 | up |
| MELO3C010506.2 | 114 | 787 | 2.787 | 1.70189E-39 | up |
| MELO3C010540.2 | 10 | 75 | 2.883 | 5.18231E-07 | up |
| MELO3C010714.2 | 45 | 98 | 1.124 | 5.35041E-05 | up |
| MELO3C010773.2 | 71 | 27 | -1.407 | 7.32215E-05 | down |
| MELO3C010779.2 | 2124 | 1039 | -1.032 | 0.001599793 | down |
| MELO3C010786.2 | 78 | 156 | 1.005 | 0.003294099 | up |
| MELO3C010801.2 | 155 | 71 | -1.131 | 0.001541821 | down |
| MELO3C010825.2 | 141 | 65 | -1.123 | 0.000198263 | down |
| MELO3C010841.2 | 19 | 50 | 1.366 | 0.005611721 | up |
| MELO3C010859.2 | 59 | 153 | 1.383 | 1.73498E-08 | up |
| MELO3C010864.2 | 25 | 8 | -1.634 | 0.008544303 | down |
| MELO3C010910.2 | 615 | 1516 | 1.302 | 3.14512E-17 | up |
| MELO3C010951.2 | 99 | 34 | -1.545 | 0.000246533 | down |
| MELO3C010954.2 | 5 | 32 | 2.858 | 2.61402E-05 | up |
| MELO3C010988.2 | 229 | 65 | -1.823 | 1.34654E-15 | down |
| MELO3C011110.2 | 121 | 248 | 1.026 | 5.55127E-07 | up |
| MELO3C011113.2 | 3863 | 8816 | 1.19 | 1.04448E-25 | up |
| MELO3C011116.2 | 54 | 10 | -2.408 | 0.005899802 | down |
| MELO3C011226.2 | 845 | 417 | -1.018 | 1.05406E-09 | down |
| MELO3C011240.2 | 23 | 82 | 1.847 | 1.61824E-05 | up |
| MELO3C011242.2 | 245 | 628 | 1.359 | 3.65561E-10 | up |
| MELO3C011250.2 | 2843 | 1214 | -1.227 | 2.93458E-18 | down |
| MELO3C011254.2 | 3 | 41 | 3.988 | 1.09761E-07 | up |
| MELO3C011360.2 | 33 | 9 | -1.905 | 0.000416237 | down |
| MELO3C011553.2 | 733 | 359 | -1.031 | 1.94156E-11 | down |
| MELO3C011576.2 | 3676 | 7966 | 1.116 | 2.53029E-37 | up |
| MELO3C011770.2 | 141 | 322 | 1.194 | 2.48172E-05 | up |
| MELO3C011930.2 | 63 | 157 | 1.321 | 0.002564905 | up |
| MELO3C011931.2 | 471 | 211 | -1.161 | 1.33521E-14 | down |
| MELO3C011988.2 | 460 | 1054 | 1.197 | 2.64175E-08 | up |
| MELO3C012111.2 | 996 | 395 | -1.332 | 1.18149E-22 | down |
| MELO3C012162.2 | 29 | 71 | 1.301 | 0.004856556 | up |
| MELO3C012176.2 | 29 | 62 | 1.088 | 0.004394504 | up |
| MELO3C012186.2 | 123 | 321 | 1.386 | 5.32125E-07 | up |
| MELO3C012218.2 | 151 | 554 | 1.872 | 1.37481E-09 | up |
| MELO3C012247.2 | 282 | 68 | -2.051 | 2.0402E-21 | down |
| MELO3C012324.2 | 10522 | 3379 | -1.639 | 5.47363E-08 | down |
| MELO3C012339.2 | 1722 | 794 | -1.117 | 2.97636E-07 | down |
| MELO3C012340.2 | 196 | 79 | -1.308 | 1.94315E-05 | down |
| MELO3C012429.2 | 8 | 51 | 2.617 | 1.79863E-05 | up |
| MELO3C012454.2 | 115 | 273 | 1.241 | 0.000159483 | up |
| MELO3C012479.2 | 10424 | 4070 | -1.357 | 1.22469E-27 | down |
| MELO3C012556.2 | 838 | 366 | -1.195 | 8.27257E-08 | down |
| MELO3C012753.2 | 26 | 69 | 1.415 | 0.002324783 | up |
| MELO3C012944.2 | 35 | 73 | 1.086 | 0.000668165 | up |
| MELO3C012962.2 | 77 | 193 | 1.337 | 5.08697E-05 | up |
| MELO3C012966.2 | 303 | 131 | -1.214 | 3.64981E-05 | down |
| MELO3C012992.2 | 208 | 90 | -1.212 | 8.81141E-08 | down |
| MELO3C013264.2 | 135 | 272 | 1.01 | 0.003640968 | up |
| MELO3C013360.2 | 128 | 32 | -2.011 | 8.43629E-07 | down |
| MELO3C013361.2 | 67 | 160 | 1.264 | 2.04903E-06 | up |
| MELO3C013366.2 | 12140 | 24616 | 1.02 | 2.28258E-26 | up |
| MELO3C013449.2 | 2297 | 350 | -2.717 | 9.23827E-57 | down |
| MELO3C013566.2 | 10446 | 3856 | -1.437 | 2.85003E-30 | down |
| MELO3C013710.2 | 1011 | 2408 | 1.253 | 1.03469E-13 | up |
| MELO3C013790.2 | 499 | 174 | -1.516 | 1.79156E-11 | down |
| MELO3C013845.2 | 22 | 64 | 1.5 | 0.001040955 | up |
| MELO3C013919.2 | 62 | 187 | 1.586 | 5.25876E-10 | up |
| MELO3C013923.2 | 489 | 2716 | 2.474 | 3.77407E-37 | up |
| MELO3C013926.2 | 34 | 92 | 1.42 | 0.000242625 | up |
| MELO3C013946.2 | 332 | 159 | -1.064 | 1.46153E-07 | down |
| MELO3C014045.2 | 5960 | 104 | -5.843 | 2.54476E-56 | down |
| MELO3C014051.2 | 131 | 2 | -5.988 | 1.20772E-18 | down |
| MELO3C014178.2 | 863 | 1828 | 1.082 | 5.42291E-14 | up |
| MELO3C014224.2 | 485 | 235 | -1.048 | 0.000418752 | down |
| MELO3C014236.2 | 21 | 48 | 1.174 | 0.004991278 | up |
| MELO3C014245.2 | 14 | 34 | 1.264 | 0.011032638 | up |
| MELO3C014319.2 | 32 | 106 | 1.719 | 1.43435E-07 | up |
| MELO3C014337.2 | 758 | 1999 | 1.4 | 1.87006E-18 | up |
| MELO3C014400.2 | 930 | 2126 | 1.192 | 1.89716E-22 | up |
| MELO3C014412.2 | 57 | 127 | 1.168 | 5.98432E-05 | up |
| MELO3C014430.2 | 61 | 218 | 1.829 | 6.82429E-15 | up |
| MELO3C014462.2 | 13 | 34 | 1.355 | 0.005550476 | up |
| MELO3C014475.2 | 41 | 121 | 1.573 | 0.00107058 | up |
| MELO3C014568.2 | 316 | 3571 | 3.501 | 1.36549E-10 | up |
| MELO3C014594.2 | 22 | 59 | 1.412 | 0.001518955 | up |
| MELO3C014630.2 | 28 | 341 | 3.615 | 0.000405425 | up |
| MELO3C014672.2 | 4104 | 1948 | -1.075 | 6.12235E-09 | down |
| MELO3C014683.2 | 558 | 210 | -1.407 | 3.11427E-07 | down |
| MELO3C014724.2 | 143 | 325 | 1.183 | 2.01103E-05 | up |
| MELO3C014762.2 | 18 | 61 | 1.762 | 0.008804804 | up |
| MELO3C014821.2 | 35 | 72 | 1.023 | 0.002941107 | up |
| MELO3C014890.2 | 287 | 113 | -1.343 | 3.06447E-06 | down |
| MELO3C014937.2 | 1007 | 2181 | 1.114 | 1.29841E-25 | up |
| MELO3C014959.2 | 53 | 23 | -1.234 | 0.002044312 | down |
| MELO3C014965.2 | 39 | 13 | -1.566 | 0.002818581 | down |
| MELO3C015005.2 | 385 | 124 | -1.628 | 3.05175E-07 | down |
| MELO3C015024.2 | 109 | 252 | 1.215 | 1.00109E-07 | up |
| MELO3C015183.2 | 4 | 33 | 2.97 | 9.24474E-06 | up |
| MELO3C015207.2 | 297 | 137 | -1.112 | 4.92498E-09 | down |
| MELO3C015216.2 | 24 | 461 | 4.273 | 1.99584E-64 | up |
| MELO3C015256.2 | 102 | 219 | 1.099 | 5.50314E-07 | up |
| MELO3C015300.2 | 59 | 4 | -3.83 | 1.9897E-11 | down |
| MELO3C015340.2 | 26 | 64 | 1.271 | 0.002915491 | up |
| MELO3C015469.2 | 5047 | 1873 | -1.43 | 1.01123E-55 | down |
| MELO3C015514.2 | 32 | 89 | 1.477 | 0.002411537 | up |
| MELO3C015593.2 | 388 | 824 | 1.088 | 1.63205E-15 | up |
| MELO3C015691.2 | 2 | 32 | 4.053 | 0.001333525 | up |
| MELO3C015744.2 | 64725 | 23180 | -1.481 | 5.88248E-20 | down |
| MELO3C015745.2 | 112 | 0 | -9.194 | 1.15021E-12 | down |
| MELO3C015852.2 | 1244 | 411 | -1.598 | 4.33187E-21 | down |
| MELO3C015894.2 | 24 | 64 | 1.393 | 0.000706869 | up |
| MELO3C016168.2 | 26 | 75 | 1.534 | 0.00010286 | up |
| MELO3C016287.2 | 70 | 155 | 1.134 | 0.00846266 | up |
| MELO3C016360.2 | 28 | 5 | -2.306 | 0.000312381 | down |
| MELO3C016539.2 | 184 | 20 | -3.213 | 2.9045E-17 | down |
| MELO3C016557.2 | 23 | 48 | 1.022 | 0.011007718 | up |
| MELO3C016569.2 | 645 | 303 | -1.093 | 6.64392E-07 | down |
| MELO3C016588.2 | 36 | 9 | -1.955 | 0.000349788 | down |
| MELO3C016590.2 | 69 | 13 | -2.439 | 2.26811E-06 | down |
| MELO3C016593.2 | 1571 | 407 | -1.95 | 9.72004E-28 | down |
| MELO3C016595.2 | 13329 | 4882 | -1.449 | 1.54263E-20 | down |
| MELO3C016685.2 | 558 | 1436 | 1.362 | 9.47464E-22 | up |
| MELO3C016769.2 | 220 | 102 | -1.114 | 0.00014784 | down |
| MELO3C016773.2 | 509 | 157 | -1.696 | 6.61941E-11 | down |
| MELO3C016794.2 | 236 | 1006 | 2.093 | 0.004644304 | up |
| MELO3C016836.2 | 85 | 34 | -1.339 | 0.005534537 | down |
| MELO3C016857.2 | 8 | 38 | 2.261 | 9.46624E-05 | up |
| MELO3C016961.2 | 21 | 47 | 1.15 | 0.005889667 | up |
| MELO3C017055.2 | 13 | 52 | 1.958 | 0.000215406 | up |
| MELO3C017146.2 | 96 | 43 | -1.174 | 0.003326487 | down |
| MELO3C017168.2 | 15 | 72 | 2.299 | 2.28037E-07 | up |
| MELO3C017210.2 | 144 | 54 | -1.415 | 0.000141884 | down |
| MELO3C017232.2 | 11 | 35 | 1.68 | 0.000826716 | up |
| MELO3C017356.2 | 22 | 67 | 1.595 | 0.00024124 | up |
| MELO3C017450.2 | 12 | 39 | 1.675 | 0.00042175 | up |
| MELO3C017478.2 | 477 | 123 | -1.959 | 0.003348936 | down |
| MELO3C017580.2 | 53 | 13 | -1.971 | 7.55001E-06 | down |
| MELO3C017582.2 | 69 | 9 | -2.885 | 2.91664E-10 | down |
| MELO3C017655.2 | 357 | 804 | 1.17 | 6.85388E-10 | up |
| MELO3C017673.2 | 1733 | 855 | -1.02 | 1.14568E-06 | down |
| MELO3C017745.2 | 40 | 89 | 1.149 | 0.000297154 | up |
| MELO3C017776.2 | 151 | 381 | 1.333 | 2.71996E-09 | up |
| MELO3C017794.2 | 147 | 19 | -2.998 | 2.67728E-10 | down |
| MELO3C017906.2 | 419 | 176 | -1.251 | 4.904E-08 | down |
| MELO3C017946.2 | 22 | 64 | 1.571 | 3.72707E-05 | up |
| MELO3C018131.2 | 181 | 405 | 1.165 | 1.53152E-06 | up |
| MELO3C018316.2 | 32 | 83 | 1.372 | 0.003248975 | up |
| MELO3C018347.2 | 278 | 93 | -1.576 | 4.34602E-06 | down |
| MELO3C018349.2 | 184 | 76 | -1.285 | 9.94074E-06 | down |
| MELO3C018385.2 | 12 | 56 | 2.217 | 6.57353E-06 | up |
| MELO3C018397.2 | 65 | 147 | 1.185 | 0.000354395 | up |
| MELO3C018450.2 | 2275 | 1024 | -1.152 | 3.02841E-15 | down |
| MELO3C018454.2 | 68 | 150 | 1.126 | 0.000528841 | up |
| MELO3C018490.2 | 14427 | 6647 | -1.118 | 0.004046806 | down |
| MELO3C018601.2 | 372 | 181 | -1.033 | 4.56153E-07 | down |
| MELO3C018603.2 | 64 | 22 | -1.588 | 8.80603E-05 | down |
| MELO3C018664.2 | 888 | 134 | -2.725 | 2.82448E-41 | down |
| MELO3C018700.2 | 2868 | 631 | -2.184 | 1.20067E-08 | down |
| MELO3C018856.2 | 3822 | 1009 | -1.921 | 4.66158E-08 | down |
| MELO3C018858.2 | 6 | 31 | 2.517 | 0.000176033 | up |
| MELO3C018859.2 | 759 | 241 | -1.656 | 3.75342E-12 | down |
| MELO3C018867.2 | 49 | 154 | 1.659 | 0.000456355 | up |
| MELO3C019135.2 | 2449 | 4900 | 1 | 1.16863E-28 | up |
| MELO3C019206.2 | 2155 | 4332 | 1.007 | 2.16719E-17 | up |
| MELO3C019221.2 | 45 | 1240 | 4.781 | 2.18222E-85 | up |
| MELO3C019254.2 | 349 | 157 | -1.158 | 3.63999E-07 | down |
| MELO3C019265.2 | 46 | 13 | -1.852 | 0.000120516 | down |
| MELO3C019297.2 | 37 | 204 | 2.444 | 1.19483E-12 | up |
| MELO3C019337.2 | 7 | 41 | 2.659 | 2.00598E-05 | up |
| MELO3C019391.2 | 269 | 126 | -1.102 | 0.000204075 | down |
| MELO3C019403.2 | 94 | 196 | 1.065 | 0.000453902 | up |
| MELO3C019537.2 | 43 | 116 | 1.41 | 0.001089626 | up |
| MELO3C019906.2 | 397 | 1026 | 1.371 | 5.81897E-23 | up |
| MELO3C019948.2 | 1452 | 701 | -1.05 | 1.61323E-13 | down |
| MELO3C020005.2 | 445 | 947 | 1.091 | 7.80653E-11 | up |
| MELO3C020038.2 | 37 | 89 | 1.298 | 0.000202743 | up |
| MELO3C020052.2 | 97 | 47 | -1.056 | 0.000383328 | down |
| MELO3C020132.2 | 1096 | 2304 | 1.071 | 2.45043E-25 | up |
| MELO3C020261.2 | 216 | 480 | 1.152 | 1.58384E-07 | up |
| MELO3C020428.2 | 268 | 620 | 1.214 | 1.12924E-05 | up |
| MELO3C020535.2 | 1109 | 459 | -1.272 | 3.21263E-19 | down |
| MELO3C020592.2 | 258 | 696 | 1.432 | 0.002106352 | up |
| MELO3C020676.2 | 133 | 308 | 1.216 | 9.98347E-07 | up |
| MELO3C020688.2 | 374 | 812 | 1.118 | 1.43056E-15 | up |
| MELO3C020767.2 | 1 | 37 | 4.875 | 1.32737E-07 | up |
| MELO3C020772.2 | 123 | 378 | 1.62 | 1.85559E-12 | up |
| MELO3C020799.2 | 177 | 86 | -1.042 | 0.000323009 | down |
| MELO3C020817.2 | 46 | 107 | 1.21 | 1.76191E-05 | up |
| MELO3C020830.2 | 41 | 18 | -1.175 | 0.007777434 | down |
| MELO3C020861.2 | 358 | 852 | 1.252 | 2.87315E-18 | up |
| MELO3C021143.2 | 3671 | 1607 | -1.192 | 0.005147436 | down |
| MELO3C021151.2 | 257 | 94 | -1.448 | 2.33989E-13 | down |
| MELO3C021155.2 | 174 | 1078 | 2.629 | 7.69342E-08 | up |
| MELO3C021300.2 | 13593 | 5852 | -1.216 | 7.02975E-34 | down |
| MELO3C021322.2 | 58 | 29 | -1.015 | 0.006227252 | down |
| MELO3C021350.2 | 407 | 171 | -1.251 | 8.91581E-16 | down |
| MELO3C021351.2 | 124 | 56 | -1.14 | 0.000135992 | down |
| MELO3C021421.2 | 105 | 48 | -1.128 | 0.00012839 | down |
| MELO3C021426.2 | 176 | 29 | -2.612 | 5.77601E-12 | down |
| MELO3C021458.2 | 97 | 367 | 1.922 | 0.000724686 | up |
| MELO3C021566.2 | 118 | 246 | 1.062 | 1.39449E-07 | up |
| MELO3C021607.2 | 5 | 29 | 2.718 | 0.005601032 | up |
| MELO3C021608.2 | 93 | 230 | 1.304 | 6.59779E-05 | up |
| MELO3C021776.2 | 51 | 104 | 1.036 | 0.00103702 | up |
| MELO3C021793.2 | 124 | 288 | 1.221 | 8.40121E-12 | up |
| MELO3C021912.2 | 57 | 135 | 1.241 | 5.83659E-05 | up |
| MELO3C021918.2 | 18 | 51 | 1.473 | 0.000826202 | up |
| MELO3C021941.2 | 4850 | 1871 | -1.374 | 2.32165E-18 | down |
| MELO3C022040.2 | 446 | 925 | 1.052 | 2.97151E-08 | up |
| MELO3C022146.2 | 78 | 208 | 1.412 | 0.000137558 | up |
| MELO3C022202.2 | 545 | 1216 | 1.159 | 1.84657E-25 | up |
| MELO3C022233.2 | 1624 | 764 | -1.09 | 4.0209E-08 | down |
| MELO3C022291.2 | 540 | 1162 | 1.106 | 2.10911E-11 | up |
| MELO3C022319.2 | 55 | 24 | -1.219 | 0.006427608 | down |
| MELO3C022410.2 | 288 | 1376 | 2.256 | 1.05583E-22 | up |
| MELO3C022436.2 | 777 | 96 | -3.012 | 7.8361E-37 | down |
| MELO3C022447.2 | 27 | 59 | 1.136 | 0.00158517 | up |
| MELO3C022449.2 | 252 | 570 | 1.176 | 2.95486E-12 | up |
| MELO3C022456.2 | 400 | 159 | -1.331 | 1.3952E-06 | down |
| MELO3C022457.2 | 2773 | 1151 | -1.268 | 2.52768E-24 | down |
| MELO3C022791.2 | 67 | 148 | 1.13 | 1.23532E-05 | up |
| MELO3C022955.2 | 44 | 15 | -1.563 | 0.001797698 | down |
| MELO3C022978.2 | 194 | 864 | 2.16 | 3.24763E-33 | up |
| MELO3C023011.2 | 93 | 41 | -1.179 | 0.003698268 | down |
| MELO3C023036.2 | 272 | 131 | -1.054 | 0.000199671 | down |
| MELO3C023045.2 | 181 | 90 | -1.016 | 0.000476087 | down |
| MELO3C023131.2 | 26313 | 54709 | 1.056 | 1.03078E-10 | up |
| MELO3C023204.2 | 14 | 48 | 1.718 | 4.41031E-05 | up |
| MELO3C023216.2 | 74 | 149 | 1.015 | 0.000538931 | up |
| MELO3C023270.2 | 110 | 228 | 1.055 | 2.17714E-05 | up |
| MELO3C023272.2 | 904 | 2834 | 1.648 | 0.004146644 | up |
| MELO3C023311.2 | 358 | 65 | -2.456 | 6.97894E-16 | down |
| MELO3C023394.2 | 1001 | 389 | -1.362 | 0.001885114 | down |
| MELO3C023441.2 | 6 | 156 | 4.687 | 4.34437E-26 | up |
| MELO3C023484.2 | 1214 | 439 | -1.466 | 2.30876E-36 | down |
| MELO3C023578.2 | 40 | 97 | 1.287 | 0.000371506 | up |
| MELO3C023709.2 | 40 | 142 | 1.84 | 3.34026E-11 | up |
| MELO3C023723.2 | 44 | 12 | -1.881 | 0.000351476 | down |
| MELO3C023770.2 | 37 | 100 | 1.464 | 9.17659E-05 | up |
| MELO3C023819.2 | 169 | 76 | -1.162 | 7.19269E-06 | down |
| MELO3C023822.2 | 143 | 293 | 1.032 | 1.50393E-06 | up |
| MELO3C023827.2 | 101 | 244 | 1.28 | 5.55644E-08 | up |
| MELO3C024263.2 | 26 | 68 | 1.361 | 0.01065783 | up |
| MELO3C024312.2 | 135 | 296 | 1.138 | 5.95738E-11 | up |
| MELO3C024396.2 | 18 | 65 | 1.849 | 1.23409E-06 | up |
| MELO3C024412.2 | 28 | 125 | 2.156 | 5.12297E-06 | up |
| MELO3C024441.2 | 49 | 102 | 1.061 | 0.002320502 | up |
| MELO3C024471.2 | 96 | 293 | 1.603 | 1.38209E-07 | up |
| MELO3C024523.2 | 13 | 39 | 1.623 | 0.006223551 | up |
| MELO3C024530.2 | 1393 | 567 | -1.297 | 2.45654E-16 | down |
| MELO3C024531.2 | 6662 | 833 | -3 | 6.99751E-20 | down |
| MELO3C024539.2 | 82 | 191 | 1.228 | 1.53399E-06 | up |
| MELO3C024545.2 | 1296 | 430 | -1.591 | 0.002011266 | down |
| MELO3C024557.2 | 142 | 300 | 1.078 | 6.24428E-05 | up |
| MELO3C024816.2 | 6 | 46 | 2.905 | 0.002171567 | up |
| MELO3C024877.2 | 25 | 66 | 1.392 | 0.000714573 | up |
| MELO3C024920.2 | 2636 | 5387 | 1.031 | 3.28589E-14 | up |
| MELO3C025025.2 | 6916 | 2878 | -1.265 | 8.41724E-14 | down |
| MELO3C025110.2 | 34 | 81 | 1.226 | 0.009296648 | up |
| MELO3C025206.2 | 50 | 183 | 1.875 | 3.65394E-12 | up |
| MELO3C025408.2 | 5274 | 2294 | -1.201 | 3.6292E-15 | down |
| MELO3C025477.2 | 94 | 356 | 1.923 | 6.93266E-25 | up |
| MELO3C025484.2 | 78 | 14 | -2.463 | 2.51323E-07 | down |
| MELO3C025485.2 | 29 | 5 | -2.652 | 0.000161954 | down |
| MELO3C025579.2 | 6 | 32 | 2.411 | 0.000255022 | up |
| MELO3C025651.2 | 175 | 388 | 1.15 | 6.29491E-14 | up |
| MELO3C025689.2 | 100 | 267 | 1.422 | 1.58788E-06 | up |
| MELO3C025712.2 | 135 | 313 | 1.21 | 1.78081E-05 | up |
| MELO3C025720.2 | 1757 | 5287 | 1.589 | 2.10401E-22 | up |
| MELO3C025907.2 | 10 | 58 | 2.538 | 0.000153571 | up |
| MELO3C025984.2 | 139 | 46 | -1.599 | 8.53932E-07 | down |
| MELO3C026008.2 | 100 | 14 | -2.833 | 6.23702E-07 | down |
| MELO3C026115.2 | 1 | 27 | 4.839 | 4.18039E-06 | up |
| MELO3C026134.2 | 97 | 208 | 1.104 | 3.82808E-05 | up |
| MELO3C026178.2 | 74 | 156 | 1.069 | 0.000967542 | up |
| MELO3C026227.2 | 161 | 70 | -1.207 | 4.11002E-07 | down |
| MELO3C026260.2 | 9 | 51 | 2.519 | 2.57029E-06 | up |
| MELO3C026289.2 | 66 | 263 | 1.985 | 2.13763E-20 | up |
| MELO3C026432.2 | 27 | 90 | 1.73 | 3.39052E-06 | up |
| MELO3C026489.2 | 68 | 334 | 2.29 | 1.35359E-18 | up |
| MELO3C026535.2 | 815 | 227 | -1.842 | 2.95181E-12 | down |
| MELO3C026558.2 | 258 | 869 | 1.754 | 8.47455E-22 | up |
| MELO3C026689.2 | 1367 | 2806 | 1.038 | 5.76308E-17 | up |
| MELO3C026889.2 | 310 | 982 | 1.665 | 0.000294546 | up |
| MELO3C026907.2 | 239 | 535 | 1.162 | 2.27705E-12 | up |
| MELO3C026947.2 | 112 | 52 | -1.092 | 0.002733006 | down |
| MELO3C027057.2 | 424 | 962 | 1.183 | 9.08634E-08 | up |
| MELO3C027075.2 | 44 | 20 | -1.149 | 0.009492124 | down |
| MELO3C027082.2 | 53 | 23 | -1.238 | 0.007288156 | down |
| MELO3C027137.2 | 33 | 9 | -1.818 | 0.000789957 | down |
| MELO3C027169.2 | 5 | 62 | 3.601 | 5.82768E-11 | up |
| MELO3C027175.2 | 66 | 27 | -1.282 | 0.005708436 | down |
| MELO3C027185.2 | 57 | 168 | 1.562 | 1.3357E-05 | up |
| MELO3C027257.2 | 42 | 150 | 1.838 | 9.70284E-05 | up |
| MELO3C027325.2 | 34 | 98 | 1.521 | 0.000168034 | up |
| MELO3C027421.2 | 234 | 533 | 1.187 | 1.06324E-16 | up |
| MELO3C027537.2 | 56 | 166 | 1.571 | 6.00044E-05 | up |
| MELO3C027914.2 | 71 | 199 | 1.476 | 8.98892E-08 | up |
| MELO3C027930.2 | 421 | 1945 | 2.207 | 1.18011E-52 | up |
| MELO3C028176.2 | 29 | 8 | -1.835 | 0.00168902 | down |
| MELO3C028402.2 | 137 | 53 | -1.351 | 1.48054E-05 | down |
| MELO3C028461.2 | 9 | 27 | 1.656 | 0.004270439 | up |
| MELO3C028965.2 | 122 | 47 | -1.362 | 1.67319E-05 | down |
| MELO3C029049.2 | 384 | 51 | -2.887 | 2.83346E-36 | down |
| MELO3C029197.2 | 1107 | 453 | -1.287 | 9.28486E-15 | down |
| MELO3C029198.2 | 204 | 76 | -1.422 | 2.23184E-06 | down |
| MELO3C029304.2 | 23 | 49 | 1.102 | 0.005721635 | up |
| MELO3C029317.2 | 209 | 846 | 2.018 | 0.004148027 | up |
| MELO3C029970.2 | 94 | 249 | 1.397 | 3.73194E-05 | up |
| MELO3C030349.2 | 61 | 14 | -2.061 | 0.000152848 | down |
| MELO3C030351.2 | 85 | 29 | -1.541 | 0.000494288 | down |
| MELO3C030358.2 | 46 | 272 | 2.579 | 7.5009E-24 | up |
| MELO3C030606.2 | 243 | 77 | -1.658 | 1.19139E-11 | down |
| MELO3C030688.2 | 28 | 57 | 1.024 | 0.00777576 | up |
| MELO3C030722.2 | 1519 | 657 | -1.209 | 6.90505E-08 | down |
| MELO3C030978.2 | 10 | 37 | 1.799 | 0.000470592 | up |
| MELO3C031023.2 | 535 | 256 | -1.068 | 1.89756E-05 | down |
| MELO3C031333.2 | 20 | 59 | 1.559 | 0.004062334 | up |
| MELO3C031342.2 | 14 | 41 | 1.603 | 0.001042239 | up |
| MELO3C031424.2 | 14 | 36 | 1.294 | 0.007057566 | up |
| MELO3C031534.2 | 40 | 18 | -1.158 | 0.008705845 | down |
| MELO3C031605.2 | 7 | 33 | 2.173 | 0.005713572 | up |
| MELO3C031876.2 | 484 | 148 | -1.709 | 4.08226E-27 | down |
| MELO3C032112.2 | 96 | 47 | -1.022 | 0.001936262 | down |
| MELO3C032309.2 | 80 | 34 | -1.256 | 0.000162096 | down |
| MELO3C032420.2 | 1722 | 861 | -1 | 9.36795E-22 | down |
| MELO3C032523.2 | 19 | 67 | 1.776 | 0.001219526 | up |
| MELO3C032593.2 | 50 | 130 | 1.392 | 6.06074E-05 | up |
| MELO3C032635.2 | 81 | 39 | -1.051 | 0.009415668 | down |
| MELO3C032794.2 | 68 | 21 | -1.712 | 7.5621E-06 | down |
| MELO3C032917.2 | 123 | 39 | -1.653 | 2.41579E-05 | down |
| MELO3C033095.2 | 35 | 0 | -7.495 | 7.28316E-08 | down |
| MELO3C033314.2 | 42 | 11 | -1.991 | 0.00025815 | down |
| MELO3C033316.2 | 168 | 39 | -2.089 | 5.45244E-09 | down |
| MELO3C033317.2 | 247 | 56 | -2.152 | 1.89219E-13 | down |
| MELO3C033320.2 | 40 | 9 | -2.152 | 0.000598533 | down |
| MELO3C033764.2 | 23 | 86 | 1.904 | 1.60655E-05 | up |
| MELO3C033882.2 | 8 | 34 | 2.238 | 0.000215693 | up |
| MELO3C033888.2 | 40 | 11 | -1.846 | 0.000206536 | down |
| MELO3C034543.2 | 12 | 40 | 1.772 | 0.000976177 | up |
| MELO3C034651.2 | 77 | 28 | -1.487 | 0.000214588 | down |
| MELO3C034872.2 | 162 | 74 | -1.121 | 9.20073E-07 | down |
| MELO3C034965.2 | 25 | 4 | -2.848 | 0.000663278 | down |
| MELO3C035199.2 | 41 | 115 | 1.502 | 2.74588E-05 | up |
| MELO3C035200.2 | 154 | 472 | 1.617 | 2.37383E-09 | up |
| MELO3C035201.2 | 10396 | 24068 | 1.211 | 7.24418E-22 | up |
| MELO3C035679.2 | 39 | 130 | 1.752 | 3.14854E-07 | up |
| MELO3C035729.2 | 41 | 17 | -1.23 | 0.004734805 | down |
