# Supplementary material for: Comprehensive Analysis of Transcriptome and Metabolome Reveals the Flavonoid Metabolic Pathway Is Associated with Fruit Peel Coloration of Melon
Source: Molecules. 2021 May 10;26(9):2830. doi: 10.3390/molecules26092830 (PMC8126211; doi:10.3390/molecules26092830)
Supplement: Supplementary file 1 [file molecules-26-02830-s001.zip › molecules-1183709-supplementary/Table S3 FPKMs and function annotation of genes in W vs H.docx]

| **Table S3 FPKMs and function annotation of genes in W vs H** | | | | | |
| --- | --- | --- | --- | --- | --- |
| **ID** | **W** | **H** | **log2FoldChange** | **pvalue** | **regulated** |
| MELO3C000026.2 | 10 | 1 | -3.965 | 0.009969911 | down |
| MELO3C000027.2 | 32 | 12 | -1.338 | 0.02658032 | down |
| MELO3C000030.2 | 163 | 46 | -1.81 | 1.91395E-06 | down |
| MELO3C000061.2 | 237 | 564 | 1.248 | 2.05119E-14 | up |
| MELO3C000062.2 | 193 | 1515 | 2.975 | 7.93985E-51 | up |
| MELO3C000064.2 | 246 | 0 | -10.496 | 1.4565E-17 | down |
| MELO3C000067.2 | 260 | 11 | -4.512 | 5.5666E-41 | down |
| MELO3C000076.2 | 0 | 73 | 8.529 | 1.04595E-11 | up |
| MELO3C000083.2 | 0 | 5 | 4.638 | 0.020079744 | up |
| MELO3C000093.2 | 148 | 0 | -9.765 | 6.35315E-15 | down |
| MELO3C000100.2 | 18 | 6 | -1.628 | 0.017659894 | down |
| MELO3C000105.2 | 14 | 1 | -3.857 | 0.002013531 | down |
| MELO3C000109.2 | 4 | 0 | -4.655 | 0.020298081 | down |
| MELO3C000111.2 | 178 | 40 | -2.152 | 8.41085E-16 | down |
| MELO3C000113.2 | 589 | 1527 | 1.375 | 1.84334E-14 | up |
| MELO3C000117.2 | 10281 | 3896 | -1.4 | 4.38032E-28 | down |
| MELO3C000123.2 | 0 | 3088 | 12.971 | 3.44386E-21 | up |
| MELO3C000130.2 | 106 | 31 | -1.781 | 5.09808E-07 | down |
| MELO3C000131.2 | 31 | 7 | -2.072 | 0.001540396 | down |
| MELO3C000147.2 | 183 | 18 | -3.294 | 1.50431E-26 | down |
| MELO3C000150.2 | 6 | 0 | -5.07 | 0.006817143 | down |
| MELO3C000161.2 | 4 | 80 | 4.334 | 4.954E-11 | up |
| MELO3C000169.2 | 174 | 355 | 1.027 | 1.12286E-05 | up |
| MELO3C000173.2 | 69 | 11 | -2.603 | 6.01361E-07 | down |
| MELO3C000196.2 | 155 | 58 | -1.42 | 2.56771E-07 | down |
| MELO3C000199.2 | 219 | 467 | 1.09 | 8.88525E-15 | up |
| MELO3C000213.2 | 84 | 451 | 2.422 | 1.0875E-33 | up |
| MELO3C000225.2 | 0 | 14 | 6.199 | 3.60877E-05 | up |
| MELO3C000228.2 | 1958 | 168 | -3.545 | 4.0094E-63 | down |
| MELO3C000237.2 | 447 | 100 | -2.158 | 3.5061E-34 | down |
| MELO3C000251.2 | 285 | 616 | 1.112 | 3.80964E-14 | up |
| MELO3C000256.2 | 150 | 0 | -9.783 | 1.95898E-15 | down |
| MELO3C000296.2 | 1 | 31 | 4.847 | 1.00987E-06 | up |
| MELO3C000311.2 | 0 | 137 | 9.441 | 2.46283E-14 | up |
| MELO3C000329.2 | 0 | 8 | 4.442 | 0.00610358 | up |
| MELO3C000333.2 | 387 | 110 | -1.823 | 3.48186E-25 | down |
| MELO3C000334.2 | 41 | 12 | -1.704 | 0.00150095 | down |
| MELO3C000335.2 | 0 | 39 | 7.623 | 8.14077E-09 | up |
| MELO3C000336.2 | 40 | 3 | -3.599 | 0.000583771 | down |
| MELO3C000366.2 | 0 | 92 | 8.855 | 2.41612E-12 | up |
| MELO3C000376.2 | 686 | 14 | -5.66 | 9.24163E-98 | down |
| MELO3C000378.2 | 127 | 665 | 2.385 | 9.58735E-28 | up |
| MELO3C000391.2 | 154 | 8 | -4.233 | 1.76487E-15 | down |
| MELO3C000392.2 | 117 | 1 | -6.571 | 1.36125E-16 | down |
| MELO3C000449.2 | 88 | 19 | -2.238 | 2.00128E-08 | down |
| MELO3C000450.2 | 0 | 110 | 9.114 | 1.26782E-13 | up |
| MELO3C000458.2 | 0 | 6 | 4.072 | 0.027728606 | up |
| MELO3C000487.2 | 20 | 0 | -6.832 | 1.17143E-05 | down |
| MELO3C000514.2 | 35 | 6 | -2.568 | 4.70854E-05 | down |
| MELO3C000515.2 | 13 | 1645 | 6.995 | 4.06867E-122 | up |
| MELO3C000526.2 | 51 | 114 | 1.177 | 8.81398E-05 | up |
| MELO3C000527.2 | 6 | 436 | 6.163 | 2.83503E-52 | up |
| MELO3C000537.2 | 198 | 71 | -1.489 | 3.29641E-08 | down |
| MELO3C000538.2 | 589 | 1638 | 1.476 | 8.88515E-22 | up |
| MELO3C000583.2 | 53 | 285 | 2.429 | 9.35086E-13 | up |
| MELO3C000592.2 | 3216 | 1357 | -1.245 | 6.86125E-13 | down |
| MELO3C000606.2 | 559 | 92 | -2.609 | 4.36466E-51 | down |
| MELO3C000609.2 | 31 | 103 | 1.751 | 0.000139136 | up |
| MELO3C000668.2 | 106 | 0 | -9.287 | 4.47514E-14 | down |
| MELO3C000776.2 | 269 | 7 | -5.208 | 5.12394E-05 | down |
| MELO3C000787.2 | 0 | 29 | 6.231 | 2.52809E-06 | up |
| MELO3C000794.2 | 8 | 0 | -5.654 | 0.001188771 | down |
| MELO3C000795.2 | 1 | 8 | 3.479 | 0.020730144 | up |
| MELO3C000818.2 | 0 | 9 | 5.461 | 0.001147895 | up |
| MELO3C000822.2 | 15 | 35 | 1.222 | 0.028710254 | up |
| MELO3C000830.2 | 0 | 27 | 7.113 | 1.54244E-07 | up |
| MELO3C000849.2 | 149 | 54 | -1.452 | 0.003608774 | down |
| MELO3C000881.2 | 447 | 5 | -6.338 | 1.38747E-58 | down |
| MELO3C000920.2 | 6 | 0 | -5.161 | 0.005331937 | down |
| MELO3C000923.2 | 0 | 5 | 4.769 | 0.015218235 | up |
| MELO3C000966.2 | 79 | 254 | 1.681 | 2.23335E-09 | up |
| MELO3C000979.2 | 17 | 1 | -4.741 | 0.000385293 | down |
| MELO3C000994.2 | 26 | 8 | -1.815 | 0.002073312 | down |
| MELO3C001068.2 | 181 | 30 | -2.587 | 2.41094E-18 | down |
| MELO3C001078.2 | 222 | 93 | -1.246 | 1.03949E-07 | down |
| MELO3C001150.2 | 61 | 272 | 2.16 | 8.39314E-21 | up |
| MELO3C001160.2 | 94 | 0 | -9.104 | 1.27183E-13 | down |
| MELO3C001165.2 | 196 | 0 | -10.165 | 9.61121E-17 | down |
| MELO3C001225.2 | 5 | 0 | -4.013 | 0.026500713 | down |
| MELO3C001235.2 | 67 | 15 | -2.145 | 3.34701E-07 | down |
| MELO3C001238.2 | 134 | 49 | -1.441 | 3.8711E-08 | down |
| MELO3C001253.2 | 63 | 0 | -8.546 | 8.44449E-12 | down |
| MELO3C001266.2 | 1 | 45 | 5.973 | 1.3481E-07 | up |
| MELO3C001321.2 | 0 | 6 | 3.885 | 0.026799962 | up |
| MELO3C001323.2 | 59 | 15 | -1.932 | 0.000544048 | down |
| MELO3C001354.2 | 16 | 202 | 3.65 | 1.10809E-26 | up |
| MELO3C001368.2 | 136 | 33 | -2.048 | 8.83266E-10 | down |
| MELO3C001388.2 | 187 | 57 | -1.696 | 1.99552E-11 | down |
| MELO3C001419.2 | 123 | 15 | -3.03 | 5.25663E-18 | down |
| MELO3C001462.2 | 367 | 173 | -1.089 | 7.86231E-08 | down |
| MELO3C001489.2 | 0 | 5 | 4.708 | 0.017839139 | up |
| MELO3C001500.2 | 0 | 28 | 6.194 | 6.02507E-06 | up |
| MELO3C001510.2 | 54 | 24 | -1.162 | 0.00817301 | down |
| MELO3C001553.2 | 23 | 6 | -1.906 | 0.00413173 | down |
| MELO3C001593.2 | 24 | 0 | -6.17 | 8.30255E-06 | down |
| MELO3C001595.2 | 15 | 2 | -2.581 | 0.014722064 | down |
| MELO3C001609.2 | 4 | 39 | 3.066 | 1.81683E-06 | up |
| MELO3C001614.2 | 141 | 16 | -3.078 | 1.12319E-21 | down |
| MELO3C001621.2 | 0 | 26 | 7.037 | 4.73006E-07 | up |
| MELO3C001651.2 | 0 | 35 | 7.451 | 1.55509E-08 | up |
| MELO3C001657.2 | 148 | 2 | -6.589 | 1.17386E-20 | down |
| MELO3C001806.2 | 12 | 0 | -6.16 | 6.92257E-05 | down |
| MELO3C001844.2 | 17 | 1 | -4.205 | 0.000876713 | down |
| MELO3C001880.2 | 5 | 0 | -5.068 | 0.00625487 | down |
| MELO3C001902.2 | 0 | 163 | 9.691 | 1.66746E-15 | up |
| MELO3C001915.2 | 0 | 59 | 8.215 | 5.75587E-11 | up |
| MELO3C001924.2 | 665 | 1984 | 1.576 | 1.18957E-61 | up |
| MELO3C001940.2 | 4 | 21 | 2.263 | 0.006513147 | up |
| MELO3C001944.2 | 1417 | 177 | -2.992 | 1.39296E-31 | down |
| MELO3C001954.2 | 88 | 358 | 2.025 | 5.71542E-17 | up |
| MELO3C001956.2 | 8 | 86 | 3.391 | 6.13115E-14 | up |
| MELO3C001964.2 | 54 | 166 | 1.607 | 2.70047E-09 | up |
| MELO3C001970.2 | 28 | 250 | 3.162 | 2.64128E-29 | up |
| MELO3C001971.2 | 110 | 12 | -3.138 | 2.78737E-14 | down |
| MELO3C001983.2 | 9 | 154 | 4.109 | 5.56011E-17 | up |
| MELO3C001992.2 | 469 | 1 | -8.582 | 5.42639E-23 | down |
| MELO3C001994.2 | 1311 | 462 | -1.504 | 5.43175E-26 | down |
| MELO3C001995.2 | 500 | 102 | -2.29 | 2.63208E-24 | down |
| MELO3C001996.2 | 972 | 246 | -1.984 | 1.32096E-59 | down |
| MELO3C001997.2 | 53 | 243 | 2.185 | 3.13299E-16 | up |
| MELO3C001998.2 | 6633 | 2849 | -1.219 | 5.24033E-21 | down |
| MELO3C002003.2 | 34 | 92 | 1.461 | 0.000249812 | up |
| MELO3C002004.2 | 17 | 58 | 1.816 | 4.089E-05 | up |
| MELO3C002009.2 | 0 | 8 | 5.284 | 0.002038641 | up |
| MELO3C002013.2 | 40 | 14 | -1.504 | 0.008415512 | down |
| MELO3C002024.2 | 88 | 39 | -1.157 | 0.001148698 | down |
| MELO3C002028.2 | 61 | 13 | -2.234 | 1.41068E-06 | down |
| MELO3C002029.2 | 321 | 63 | -2.341 | 2.28079E-30 | down |
| MELO3C002030.2 | 3069 | 6924 | 1.174 | 1.13143E-26 | up |
| MELO3C002033.2 | 3081 | 1536 | -1.004 | 8.87571E-32 | down |
| MELO3C002036.2 | 1179 | 366 | -1.691 | 1.25391E-19 | down |
| MELO3C002043.2 | 3 | 17 | 2.266 | 0.006047041 | up |
| MELO3C002044.2 | 80 | 304 | 1.933 | 4.44959E-18 | up |
| MELO3C002049.2 | 52 | 2 | -5.106 | 8.31334E-08 | down |
| MELO3C002056.2 | 722 | 2037 | 1.497 | 4.05649E-53 | up |
| MELO3C002059.2 | 103 | 5 | -4.491 | 1.59458E-18 | down |
| MELO3C002063.2 | 2970 | 791 | -1.908 | 1.89022E-21 | down |
| MELO3C002066.2 | 20 | 119 | 2.6 | 1.52565E-11 | up |
| MELO3C002070.2 | 233 | 15 | -3.923 | 6.47232E-22 | down |
| MELO3C002072.2 | 56 | 23 | -1.327 | 0.000520366 | down |
| MELO3C002073.2 | 1123 | 88 | -3.667 | 3.21853E-85 | down |
| MELO3C002078.2 | 5898 | 2446 | -1.27 | 5.77702E-28 | down |
| MELO3C002079.2 | 131 | 850 | 2.703 | 3.13077E-24 | up |
| MELO3C002083.2 | 106 | 51 | -1.043 | 0.020604145 | down |
| MELO3C002090.2 | 65 | 171 | 1.409 | 0.002482233 | up |
| MELO3C002100.2 | 5 | 25 | 2.325 | 0.000889444 | up |
| MELO3C002105.2 | 240 | 845 | 1.813 | 1.32141E-28 | up |
| MELO3C002106.2 | 11 | 66 | 2.666 | 3.03908E-07 | up |
| MELO3C002112.2 | 57 | 389 | 2.753 | 1.8983E-44 | up |
| MELO3C002114.2 | 5 | 48 | 3.246 | 2.91725E-05 | up |
| MELO3C002119.2 | 5395 | 2305 | -1.227 | 3.28009E-20 | down |
| MELO3C002122.2 | 52 | 769 | 3.878 | 4.90165E-38 | up |
| MELO3C002126.2 | 1617 | 258 | -2.651 | 3.75784E-44 | down |
| MELO3C002128.2 | 5 | 210 | 5.498 | 1.98004E-24 | up |
| MELO3C002131.2 | 110 | 561 | 2.347 | 1.51346E-42 | up |
| MELO3C002138.2 | 198 | 672 | 1.762 | 4.32037E-42 | up |
| MELO3C002140.2 | 94 | 7 | -3.663 | 5.57463E-15 | down |
| MELO3C002143.2 | 71 | 349 | 2.298 | 1.68531E-18 | up |
| MELO3C002144.2 | 368 | 2972 | 3.013 | 3.46021E-122 | up |
| MELO3C002145.2 | 7 | 0 | -5.39 | 0.001851316 | down |
| MELO3C002147.2 | 996 | 369 | -1.43 | 4.66985E-19 | down |
| MELO3C002154.2 | 761 | 78 | -3.293 | 4.35086E-33 | down |
| MELO3C002161.2 | 190 | 2 | -6.677 | 3.37941E-25 | down |
| MELO3C002172.2 | 262 | 595 | 1.183 | 1.6863E-08 | up |
| MELO3C002185.2 | 24 | 72 | 1.614 | 1.37612E-05 | up |
| MELO3C002187.2 | 3595 | 1598 | -1.169 | 3.26398E-13 | down |
| MELO3C002199.2 | 56 | 21 | -1.393 | 0.000577276 | down |
| MELO3C002202.2 | 1 | 17 | 3.624 | 0.000406626 | up |
| MELO3C002206.2 | 973 | 415 | -1.229 | 2.08029E-29 | down |
| MELO3C002207.2 | 150 | 7 | -4.428 | 7.25662E-20 | down |
| MELO3C002208.2 | 6591 | 525 | -3.651 | 4.22465E-72 | down |
| MELO3C002209.2 | 21703 | 5596 | -1.955 | 2.10039E-42 | down |
| MELO3C002210.2 | 37 | 2 | -4.603 | 5.06767E-07 | down |
| MELO3C002212.2 | 6 | 28 | 2.085 | 0.008803183 | up |
| MELO3C002216.2 | 53 | 17 | -1.623 | 0.000378442 | down |
| MELO3C002224.2 | 174 | 56 | -1.649 | 1.5109E-08 | down |
| MELO3C002227.2 | 1001 | 372 | -1.424 | 9.63565E-17 | down |
| MELO3C002228.2 | 9312 | 4433 | -1.071 | 3.21398E-55 | down |
| MELO3C002244.2 | 78 | 20 | -1.984 | 4.57407E-07 | down |
| MELO3C002247.2 | 91 | 31 | -1.551 | 0.000196989 | down |
| MELO3C002248.2 | 11 | 219 | 4.255 | 7.52412E-37 | up |
| MELO3C002253.2 | 35 | 9 | -1.917 | 0.014259909 | down |
| MELO3C002262.2 | 257 | 101 | -1.346 | 7.58755E-10 | down |
| MELO3C002269.2 | 9789 | 28682 | 1.551 | 1.54847E-25 | up |
| MELO3C002271.2 | 915 | 302 | -1.599 | 4.14412E-38 | down |
| MELO3C002272.2 | 93 | 44 | -1.082 | 0.017185754 | down |
| MELO3C002276.2 | 2012 | 768 | -1.391 | 1.07923E-46 | down |
| MELO3C002277.2 | 776 | 145 | -2.418 | 2.87635E-20 | down |
| MELO3C002280.2 | 56 | 492 | 3.139 | 6.40751E-31 | up |
| MELO3C002284.2 | 999 | 182 | -2.45 | 9.77705E-49 | down |
| MELO3C002285.2 | 433 | 187 | -1.21 | 2.74113E-07 | down |
| MELO3C002286.2 | 23 | 60 | 1.427 | 0.000192753 | up |
| MELO3C002292.2 | 0 | 153 | 9.594 | 1.32791E-14 | up |
| MELO3C002298.2 | 569 | 222 | -1.357 | 2.45837E-15 | down |
| MELO3C002305.2 | 43 | 12 | -1.816 | 0.001019115 | down |
| MELO3C002309.2 | 21 | 88 | 2.082 | 3.9561E-08 | up |
| MELO3C002316.2 | 1798 | 154 | -3.547 | 2.71388E-176 | down |
| MELO3C002319.2 | 45 | 11860 | 8.052 | 5.24575E-17 | up |
| MELO3C002337.2 | 6 | 42 | 2.684 | 1.90174E-06 | up |
| MELO3C002346.2 | 1048 | 2494 | 1.251 | 2.42959E-35 | up |
| MELO3C002347.2 | 942 | 2039 | 1.114 | 7.28757E-37 | up |
| MELO3C002350.2 | 1622 | 480 | -1.756 | 3.37657E-41 | down |
| MELO3C002351.2 | 431 | 1052 | 1.287 | 1.63036E-13 | up |
| MELO3C002360.2 | 5249 | 1168 | -2.168 | 3.42806E-85 | down |
| MELO3C002369.2 | 344 | 14 | -4.629 | 1.0944E-53 | down |
| MELO3C002374.2 | 39 | 0 | -7.837 | 1.53522E-09 | down |
| MELO3C002381.2 | 74 | 3 | -4.609 | 8.31391E-11 | down |
| MELO3C002383.2 | 2 | 38 | 4.137 | 0.000243437 | up |
| MELO3C002387.2 | 1700 | 819 | -1.054 | 3.03189E-22 | down |
| MELO3C002391.2 | 247 | 0 | -10.504 | 2.13509E-18 | down |
| MELO3C002392.2 | 280 | 1 | -8.834 | 3.70937E-17 | down |
| MELO3C002393.2 | 34 | 530 | 3.962 | 1.89274E-39 | up |
| MELO3C002394.2 | 35 | 372 | 3.405 | 2.40541E-23 | up |
| MELO3C002396.2 | 1070 | 508 | -1.077 | 3.29723E-21 | down |
| MELO3C002414.2 | 34 | 171 | 2.322 | 2.01116E-09 | up |
| MELO3C002416.2 | 660 | 2699 | 2.031 | 3.78885E-90 | up |
| MELO3C002426.2 | 61 | 28 | -1.138 | 0.008175708 | down |
| MELO3C002435.2 | 28 | 212 | 2.943 | 4.14288E-21 | up |
| MELO3C002436.2 | 3 | 82 | 4.835 | 7.29769E-15 | up |
| MELO3C002437.2 | 90 | 4635 | 5.677 | 9.71958E-22 | up |
| MELO3C002441.2 | 0 | 205 | 10.022 | 1.37688E-15 | up |
| MELO3C002454.2 | 6 | 40 | 2.801 | 2.7223E-05 | up |
| MELO3C002456.2 | 6856 | 1869 | -1.876 | 1.21746E-37 | down |
| MELO3C002457.2 | 1207 | 10769 | 3.157 | 6.34878E-09 | up |
| MELO3C002458.2 | 6 | 26 | 2.117 | 0.008094468 | up |
| MELO3C002459.2 | 869 | 390 | -1.155 | 2.49017E-07 | down |
| MELO3C002468.2 | 8206 | 3742 | -1.132 | 3.62929E-34 | down |
| MELO3C002470.2 | 310 | 650 | 1.07 | 1.28916E-12 | up |
| MELO3C002479.2 | 290 | 62 | -2.222 | 7.20589E-12 | down |
| MELO3C002480.2 | 3 | 22 | 2.93 | 0.000987866 | up |
| MELO3C002483.2 | 192 | 12 | -4.025 | 2.45996E-21 | down |
| MELO3C002485.2 | 801 | 74 | -3.426 | 1.64498E-63 | down |
| MELO3C002493.2 | 28 | 7 | -2.013 | 0.004603443 | down |
| MELO3C002495.2 | 25 | 62 | 1.309 | 0.00051479 | up |
| MELO3C002500.2 | 12 | 1 | -4.385 | 0.003734521 | down |
| MELO3C002501.2 | 64 | 1 | -6.101 | 2.30332E-09 | down |
| MELO3C002504.2 | 26133 | 7158 | -1.868 | 2.25521E-38 | down |
| MELO3C002507.2 | 571 | 265 | -1.105 | 4.96744E-12 | down |
| MELO3C002508.2 | 10270 | 1951 | -2.395 | 1.89452E-72 | down |
| MELO3C002510.2 | 24036 | 2941 | -3.031 | 3.37624E-93 | down |
| MELO3C002511.2 | 196 | 9 | -4.444 | 4.38347E-24 | down |
| MELO3C002521.2 | 854 | 228 | -1.901 | 2.84874E-16 | down |
| MELO3C002529.2 | 0 | 11 | 5.756 | 0.000361131 | up |
| MELO3C002535.2 | 32 | 96 | 1.587 | 0.000277234 | up |
| MELO3C002536.2 | 52 | 24 | -1.144 | 0.012750292 | down |
| MELO3C002538.2 | 4 | 14 | 2.013 | 0.017748089 | up |
| MELO3C002546.2 | 64 | 189 | 1.571 | 1.89994E-08 | up |
| MELO3C002551.2 | 34 | 3 | -3.493 | 2.55711E-05 | down |
| MELO3C002553.2 | 121 | 440 | 1.864 | 1.42318E-16 | up |
| MELO3C002555.2 | 30 | 273 | 3.181 | 1.21875E-31 | up |
| MELO3C002560.2 | 160 | 23 | -2.822 | 3.34716E-09 | down |
| MELO3C002562.2 | 131 | 43 | -1.609 | 2.66562E-08 | down |
| MELO3C002563.2 | 2020 | 897 | -1.17 | 2.01751E-31 | down |
| MELO3C002564.2 | 2342 | 889 | -1.398 | 1.06336E-37 | down |
| MELO3C002572.2 | 72 | 5 | -3.914 | 6.14352E-07 | down |
| MELO3C002590.2 | 33 | 8 | -2.016 | 0.002042616 | down |
| MELO3C002599.2 | 14 | 33 | 1.267 | 0.019040536 | up |
| MELO3C002603.2 | 1661 | 3707 | 1.158 | 4.18189E-45 | up |
| MELO3C002605.2 | 488 | 989 | 1.02 | 7.73705E-20 | up |
| MELO3C002606.2 | 238 | 85 | -1.497 | 8.73466E-07 | down |
| MELO3C002607.2 | 49 | 7 | -2.889 | 1.72694E-05 | down |
| MELO3C002609.2 | 87 | 245 | 1.502 | 2.01084E-15 | up |
| MELO3C002615.2 | 32 | 2 | -4.164 | 3.48873E-05 | down |
| MELO3C002616.2 | 199 | 44 | -2.169 | 5.25167E-13 | down |
| MELO3C002617.2 | 87 | 282 | 1.705 | 1.80976E-05 | up |
| MELO3C002618.2 | 22 | 53 | 1.252 | 0.002896758 | up |
| MELO3C002624.2 | 202 | 31 | -2.718 | 0.002870863 | down |
| MELO3C002628.2 | 1847 | 339 | -2.447 | 5.37031E-91 | down |
| MELO3C002637.2 | 27 | 5 | -2.537 | 0.000224691 | down |
| MELO3C002641.2 | 1483 | 3447 | 1.217 | 5.82232E-35 | up |
| MELO3C002647.2 | 30 | 1 | -4.622 | 1.07266E-06 | down |
| MELO3C002661.2 | 156 | 2 | -6.397 | 7.75655E-23 | down |
| MELO3C002664.2 | 607 | 1964 | 1.694 | 6.16711E-75 | up |
| MELO3C002669.2 | 16 | 4 | -2.153 | 0.008187928 | down |
| MELO3C002674.2 | 713 | 20 | -5.157 | 1.22559E-113 | down |
| MELO3C002676.2 | 283 | 110 | -1.357 | 1.17876E-06 | down |
| MELO3C002677.2 | 5173 | 1913 | -1.435 | 8.13276E-78 | down |
| MELO3C002678.2 | 2414 | 667 | -1.856 | 2.50902E-57 | down |
| MELO3C002679.2 | 1358 | 493 | -1.463 | 5.95395E-40 | down |
| MELO3C002685.2 | 251 | 753 | 1.585 | 5.30822E-27 | up |
| MELO3C002691.2 | 5821 | 185 | -4.973 | 1.26616E-174 | down |
| MELO3C002695.2 | 135 | 25 | -2.414 | 9.24997E-14 | down |
| MELO3C002697.2 | 2 | 40 | 3.996 | 1.79009E-05 | up |
| MELO3C002700.2 | 232 | 1844 | 2.989 | 9.80522E-131 | up |
| MELO3C002709.2 | 8 | 34 | 2.047 | 0.00072151 | up |
| MELO3C002716.2 | 2247 | 5101 | 1.183 | 4.44333E-30 | up |
| MELO3C002718.2 | 12 | 2 | -2.672 | 0.005477572 | down |
| MELO3C002723.2 | 0 | 21 | 6.719 | 2.3262E-06 | up |
| MELO3C002726.2 | 936 | 132 | -2.828 | 3.52739E-34 | down |
| MELO3C002727.2 | 42376 | 19 | -11.104 | 1.40161E-85 | down |
| MELO3C002729.2 | 792 | 1791 | 1.177 | 1.36905E-06 | up |
| MELO3C002730.2 | 110 | 256 | 1.214 | 2.86442E-07 | up |
| MELO3C002731.2 | 334 | 135 | -1.312 | 7.62904E-12 | down |
| MELO3C002732.2 | 438 | 1147 | 1.388 | 4.92513E-20 | up |
| MELO3C002736.2 | 1108 | 497 | -1.158 | 2.30597E-25 | down |
| MELO3C002747.2 | 51 | 174 | 1.766 | 4.27918E-08 | up |
| MELO3C002750.2 | 563 | 150 | -1.904 | 1.41236E-34 | down |
| MELO3C002755.2 | 1247 | 78 | -3.979 | 1.72063E-66 | down |
| MELO3C002756.2 | 139 | 62 | -1.163 | 8.00345E-06 | down |
| MELO3C002763.2 | 719 | 32 | -4.485 | 6.25711E-83 | down |
| MELO3C002768.2 | 2582 | 12 | -7.7 | 1.7732E-125 | down |
| MELO3C002770.2 | 237 | 492 | 1.053 | 2.66491E-14 | up |
| MELO3C002771.2 | 551 | 1152 | 1.065 | 4.33794E-07 | up |
| MELO3C002778.2 | 4 | 289 | 6.201 | 1.64995E-28 | up |
| MELO3C002779.2 | 1 | 49 | 5.5 | 2.65954E-08 | up |
| MELO3C002781.2 | 108 | 336 | 1.641 | 2.58178E-17 | up |
| MELO3C002807.2 | 20 | 53 | 1.409 | 0.010051164 | up |
| MELO3C002809.2 | 13 | 95 | 2.807 | 4.88514E-06 | up |
| MELO3C002811.2 | 111 | 303 | 1.455 | 2.32085E-12 | up |
| MELO3C002812.2 | 39 | 191 | 2.304 | 3.38652E-17 | up |
| MELO3C002829.2 | 75 | 153 | 1.025 | 7.61279E-05 | up |
| MELO3C002830.2 | 6040 | 2548 | -1.245 | 4.71315E-15 | down |
| MELO3C002832.2 | 209 | 805 | 1.948 | 7.03067E-52 | up |
| MELO3C002839.2 | 1133 | 45 | -4.653 | 1.04625E-102 | down |
| MELO3C002853.2 | 76 | 42590 | 9.128 | 5.97925E-15 | up |
| MELO3C002855.2 | 25 | 88 | 1.825 | 7.23013E-06 | up |
| MELO3C002874.2 | 33667 | 2304 | -3.869 | 1.80292E-108 | down |
| MELO3C002875.2 | 1 | 145 | 6.661 | 5.04301E-15 | up |
| MELO3C002877.2 | 270 | 844 | 1.644 | 1.98621E-30 | up |
| MELO3C002892.2 | 0 | 17 | 6.461 | 6.82077E-06 | up |
| MELO3C002917.2 | 17 | 37 | 1.139 | 0.018663916 | up |
| MELO3C002921.2 | 929 | 84 | -3.454 | 4.3591E-50 | down |
| MELO3C002925.2 | 33 | 0 | -7.57 | 1.11589E-06 | down |
| MELO3C002934.2 | 1618 | 697 | -1.216 | 1.62254E-21 | down |
| MELO3C002941.2 | 29 | 1395 | 5.568 | 4.35676E-111 | up |
| MELO3C002962.2 | 440 | 13 | -5.129 | 2.7737E-53 | down |
| MELO3C002976.2 | 28 | 74 | 1.429 | 0.00068933 | up |
| MELO3C002978.2 | 684 | 39 | -4.141 | 2.96996E-11 | down |
| MELO3C003002.2 | 52 | 2 | -4.427 | 3.40668E-09 | down |
| MELO3C003023.2 | 1630 | 790 | -1.044 | 5.26715E-21 | down |
| MELO3C003032.2 | 228 | 11 | -4.295 | 1.51437E-34 | down |
| MELO3C003043.2 | 178 | 368 | 1.046 | 1.81948E-09 | up |
| MELO3C003061.2 | 2649 | 1017 | -1.382 | 1.20319E-23 | down |
| MELO3C003066.2 | 5 | 82 | 4.036 | 2.05458E-08 | up |
| MELO3C003075.2 | 20546 | 9712 | -1.081 | 1.63533E-36 | down |
| MELO3C003088.2 | 1509 | 592 | -1.35 | 1.47342E-34 | down |
| MELO3C003090.2 | 96 | 10 | -3.302 | 1.24132E-12 | down |
| MELO3C003092.2 | 256 | 525 | 1.037 | 5.07205E-10 | up |
| MELO3C003093.2 | 3290 | 1334 | -1.303 | 1.10239E-26 | down |
| MELO3C003097.2 | 3314 | 1614 | -1.037 | 1.17367E-22 | down |
| MELO3C003099.2 | 36 | 97 | 1.431 | 3.51946E-06 | up |
| MELO3C003107.2 | 809 | 1963 | 1.28 | 2.3945E-15 | up |
| MELO3C003112.2 | 182 | 51 | -1.825 | 3.03988E-11 | down |
| MELO3C003121.2 | 17 | 47 | 1.497 | 0.004867193 | up |
| MELO3C003131.2 | 217 | 29 | -2.909 | 8.22965E-21 | down |
| MELO3C003132.2 | 17667 | 6460 | -1.451 | 6.06016E-18 | down |
| MELO3C003134.2 | 504 | 1061 | 1.074 | 4.02786E-07 | up |
| MELO3C003137.2 | 3128 | 1277 | -1.292 | 2.90844E-14 | down |
| MELO3C003143.2 | 3322 | 1561 | -1.089 | 1.44114E-39 | down |
| MELO3C003146.2 | 112 | 1 | -6.518 | 7.07222E-16 | down |
| MELO3C003147.2 | 3415 | 699 | -2.287 | 1.23808E-72 | down |
| MELO3C003148.2 | 248 | 1 | -7.64 | 3.27229E-22 | down |
| MELO3C003150.2 | 13017 | 5135 | -1.342 | 1.80217E-26 | down |
| MELO3C003157.2 | 544 | 166 | -1.714 | 3.07904E-15 | down |
| MELO3C003167.2 | 0 | 5 | 4.62 | 0.021407198 | up |
| MELO3C003175.2 | 17 | 2 | -2.964 | 0.001253847 | down |
| MELO3C003177.2 | 45 | 1 | -6.199 | 2.97719E-07 | down |
| MELO3C003183.2 | 26 | 0 | -7.241 | 8.73561E-08 | down |
| MELO3C003187.2 | 76 | 499 | 2.72 | 2.84279E-30 | up |
| MELO3C003188.2 | 4 | 33 | 3.214 | 0.000211861 | up |
| MELO3C003192.2 | 12511 | 5776 | -1.115 | 8.18761E-11 | down |
| MELO3C003204.2 | 54 | 2 | -4.494 | 3.36009E-09 | down |
| MELO3C003205.2 | 4801 | 893 | -2.427 | 5.76782E-174 | down |
| MELO3C003206.2 | 155 | 344 | 1.156 | 4.56231E-09 | up |
| MELO3C003214.2 | 5665 | 1557 | -1.864 | 1.33833E-31 | down |
| MELO3C003224.2 | 37 | 129 | 1.822 | 7.90954E-09 | up |
| MELO3C003227.2 | 805 | 284 | -1.504 | 2.73817E-18 | down |
| MELO3C003228.2 | 30 | 72 | 1.265 | 0.000503349 | up |
| MELO3C003229.2 | 859 | 1796 | 1.064 | 1.57224E-30 | up |
| MELO3C003238.2 | 33887 | 8991 | -1.914 | 1.10136E-28 | down |
| MELO3C003239.2 | 90 | 192 | 1.09 | 1.02284E-07 | up |
| MELO3C003241.2 | 29 | 88 | 1.588 | 4.56512E-05 | up |
| MELO3C003245.2 | 2682 | 998 | -1.426 | 2.57959E-21 | down |
| MELO3C003254.2 | 44 | 0 | -8.013 | 3.93279E-10 | down |
| MELO3C003255.2 | 249 | 0 | -10.51 | 1.92477E-18 | down |
| MELO3C003259.2 | 2 | 12 | 2.287 | 0.027870346 | up |
| MELO3C003271.2 | 5 | 19 | 1.94 | 0.006947217 | up |
| MELO3C003275.2 | 18 | 3 | -2.723 | 0.004883648 | down |
| MELO3C003284.2 | 312 | 63 | -2.297 | 1.7187E-17 | down |
| MELO3C003295.2 | 304 | 128 | -1.25 | 1.70931E-08 | down |
| MELO3C003299.2 | 31 | 464 | 3.881 | 7.74068E-56 | up |
| MELO3C003305.2 | 157 | 720 | 2.202 | 1.03249E-25 | up |
| MELO3C003308.2 | 1890 | 762 | -1.311 | 2.79204E-29 | down |
| MELO3C003316.2 | 334 | 144 | -1.215 | 3.70506E-06 | down |
| MELO3C003318.2 | 23 | 0 | -7.055 | 4.09363E-06 | down |
| MELO3C003321.2 | 130 | 55 | -1.225 | 7.78109E-05 | down |
| MELO3C003324.2 | 16317 | 4885 | -1.74 | 3.10225E-29 | down |
| MELO3C003325.2 | 11 | 0 | -6.024 | 0.000103079 | down |
| MELO3C003328.2 | 759 | 144 | -2.397 | 2.11777E-31 | down |
| MELO3C003331.2 | 8126 | 893 | -3.187 | 2.62962E-92 | down |
| MELO3C003337.2 | 10 | 0 | -5.826 | 0.000291081 | down |
| MELO3C003361.2 | 18 | 2 | -2.918 | 0.016099338 | down |
| MELO3C003362.2 | 1357 | 3197 | 1.236 | 2.12195E-26 | up |
| MELO3C003366.2 | 0 | 27 | 7.104 | 1.2323E-07 | up |
| MELO3C003372.2 | 791 | 311 | -1.349 | 3.02795E-22 | down |
| MELO3C003373.2 | 1578 | 607 | -1.379 | 3.37897E-24 | down |
| MELO3C003375.2 | 46766 | 18918 | -1.306 | 1.81697E-34 | down |
| MELO3C003379.2 | 393 | 171 | -1.194 | 3.19847E-13 | down |
| MELO3C003380.2 | 17 | 39 | 1.222 | 0.024695089 | up |
| MELO3C003381.2 | 696 | 1505 | 1.112 | 1.20017E-27 | up |
| MELO3C003384.2 | 815 | 0 | -12.224 | 1.23638E-24 | down |
| MELO3C003385.2 | 47 | 0 | -8.099 | 6.73676E-10 | down |
| MELO3C003386.2 | 1886 | 496 | -1.929 | 1.65827E-12 | down |
| MELO3C003387.2 | 13 | 1 | -4.366 | 0.00920322 | down |
| MELO3C003388.2 | 51 | 122 | 1.24 | 5.92761E-05 | up |
| MELO3C003390.2 | 168 | 52 | -1.701 | 9.86543E-08 | down |
| MELO3C003393.2 | 1287 | 193 | -2.737 | 8.92258E-97 | down |
| MELO3C003394.2 | 1739 | 188 | -3.208 | 9.42531E-33 | down |
| MELO3C003395.2 | 1915 | 559 | -1.778 | 3.41936E-34 | down |
| MELO3C003413.2 | 171 | 392 | 1.194 | 1.1797E-08 | up |
| MELO3C003425.2 | 706 | 306 | -1.205 | 5.59493E-16 | down |
| MELO3C003426.2 | 489 | 132 | -1.892 | 4.4077E-28 | down |
| MELO3C003431.2 | 186 | 396 | 1.095 | 1.21852E-10 | up |
| MELO3C003433.2 | 525 | 121 | -2.114 | 1.01504E-22 | down |
| MELO3C003439.2 | 3878 | 1905 | -1.026 | 4.11759E-22 | down |
| MELO3C003441.2 | 5731 | 2045 | -1.487 | 1.55313E-20 | down |
| MELO3C003451.2 | 14 | 56 | 2.017 | 1.01776E-06 | up |
| MELO3C003452.2 | 161 | 391 | 1.278 | 5.27403E-13 | up |
| MELO3C003460.2 | 18 | 44 | 1.308 | 0.006584577 | up |
| MELO3C003468.2 | 126 | 377 | 1.576 | 1.12528E-05 | up |
| MELO3C003469.2 | 191 | 28 | -2.722 | 1.25886E-07 | down |
| MELO3C003473.2 | 62 | 16 | -1.918 | 6.52804E-06 | down |
| MELO3C003479.2 | 1353 | 501 | -1.432 | 5.16624E-23 | down |
| MELO3C003483.2 | 696 | 243 | -1.519 | 2.77543E-12 | down |
| MELO3C003485.2 | 82 | 21 | -1.952 | 1.85147E-05 | down |
| MELO3C003494.2 | 12 | 86 | 2.877 | 9.84989E-09 | up |
| MELO3C003496.2 | 366 | 881 | 1.27 | 1.44408E-24 | up |
| MELO3C003502.2 | 3 | 11 | 2.004 | 0.028029144 | up |
| MELO3C003506.2 | 551 | 127 | -2.113 | 3.41262E-48 | down |
| MELO3C003507.2 | 381 | 72 | -2.396 | 1.51575E-30 | down |
| MELO3C003508.2 | 561 | 267 | -1.073 | 2.92649E-12 | down |
| MELO3C003519.2 | 22 | 328 | 3.908 | 5.55194E-41 | up |
| MELO3C003520.2 | 8370 | 905 | -3.21 | 3.62049E-81 | down |
| MELO3C003522.2 | 40 | 89 | 1.14 | 0.000940648 | up |
| MELO3C003526.2 | 26 | 2 | -3.584 | 6.18548E-05 | down |
| MELO3C003532.2 | 612 | 1489 | 1.283 | 6.34349E-38 | up |
| MELO3C003540.2 | 1026 | 4260 | 2.054 | 2.2452E-44 | up |
| MELO3C003546.2 | 2685 | 777 | -1.791 | 5.99485E-38 | down |
| MELO3C003554.2 | 440 | 2690 | 2.612 | 9.20056E-09 | up |
| MELO3C003558.2 | 1 | 23 | 4.443 | 3.36402E-05 | up |
| MELO3C003559.2 | 421 | 134 | -1.653 | 4.86081E-07 | down |
| MELO3C003561.2 | 1616 | 655 | -1.302 | 2.73071E-46 | down |
| MELO3C003565.2 | 2 | 55 | 4.666 | 1.7062E-09 | up |
| MELO3C003569.2 | 6482 | 2974 | -1.124 | 1.52426E-33 | down |
| MELO3C003576.2 | 13 | 1 | -3.131 | 0.013531444 | down |
| MELO3C003585.2 | 67 | 30 | -1.178 | 0.003810611 | down |
| MELO3C003598.2 | 27 | 7 | -1.97 | 0.006232204 | down |
| MELO3C003604.2 | 14 | 48 | 1.743 | 8.42716E-05 | up |
| MELO3C003605.2 | 9 | 26 | 1.533 | 0.024438162 | up |
| MELO3C003616.2 | 46 | 438 | 3.259 | 1.51629E-44 | up |
| MELO3C003617.2 | 4 | 116 | 4.882 | 6.72908E-18 | up |
| MELO3C003620.2 | 444 | 41 | -3.426 | 7.27887E-63 | down |
| MELO3C003622.2 | 3186 | 865 | -1.881 | 6.71248E-90 | down |
| MELO3C003623.2 | 1694 | 362 | -2.229 | 7.63992E-64 | down |
| MELO3C003628.2 | 103 | 366 | 1.826 | 7.29524E-11 | up |
| MELO3C003629.2 | 3 | 206 | 6 | 2.40877E-28 | up |
| MELO3C003633.2 | 53 | 0 | -8.29 | 5.42522E-11 | down |
| MELO3C003634.2 | 69 | 2 | -5.494 | 4.08928E-11 | down |
| MELO3C003635.2 | 11 | 33 | 1.58 | 0.002461677 | up |
| MELO3C003638.2 | 28 | 170 | 2.59 | 5.73789E-19 | up |
| MELO3C003642.2 | 346 | 109 | -1.675 | 2.80898E-17 | down |
| MELO3C003644.2 | 1824 | 518 | -1.817 | 4.80399E-18 | down |
| MELO3C003652.2 | 1 | 59 | 5.375 | 1.94094E-09 | up |
| MELO3C003656.2 | 341 | 48 | -2.83 | 4.11721E-27 | down |
| MELO3C003657.2 | 271 | 56 | -2.258 | 4.0256E-21 | down |
| MELO3C003659.2 | 535 | 1306 | 1.288 | 4.36816E-30 | up |
| MELO3C003662.2 | 295 | 93 | -1.667 | 1.64206E-12 | down |
| MELO3C003669.2 | 1792 | 3787 | 1.08 | 9.16359E-35 | up |
| MELO3C003672.2 | 8 | 43 | 2.311 | 0.000664657 | up |
| MELO3C003674.2 | 149 | 393 | 1.395 | 1.99063E-15 | up |
| MELO3C003676.2 | 117 | 15 | -2.927 | 2.67667E-12 | down |
| MELO3C003680.2 | 78 | 8 | -3.204 | 2.44209E-09 | down |
| MELO3C003686.2 | 1960 | 145 | -3.756 | 2.83355E-54 | down |
| MELO3C003689.2 | 2627 | 12473 | 2.248 | 1.46346E-82 | up |
| MELO3C003692.2 | 206 | 655 | 1.671 | 1.9747E-19 | up |
| MELO3C003697.2 | 7425 | 16006 | 1.108 | 1.04469E-42 | up |
| MELO3C003698.2 | 123 | 700 | 2.509 | 4.53684E-50 | up |
| MELO3C003699.2 | 2 | 11 | 2.112 | 0.028021211 | up |
| MELO3C003716.2 | 1 | 46 | 4.991 | 4.47141E-07 | up |
| MELO3C003720.2 | 99 | 0 | -8.217 | 1.91194E-11 | down |
| MELO3C003721.2 | 2050 | 661 | -1.633 | 1.90124E-77 | down |
| MELO3C003725.2 | 14086 | 266 | -5.729 | 1.18219E-170 | down |
| MELO3C003729.2 | 541 | 1123 | 1.053 | 1.98731E-27 | up |
| MELO3C003731.2 | 1357 | 5476 | 2.013 | 9.94441E-41 | up |
| MELO3C003737.2 | 112 | 25 | -2.17 | 0.00078797 | down |
| MELO3C003743.2 | 588 | 167 | -1.818 | 5.44123E-27 | down |
| MELO3C003753.2 | 77 | 31 | -1.315 | 4.56233E-05 | down |
| MELO3C003757.2 | 2 | 33 | 3.959 | 3.12586E-05 | up |
| MELO3C003759.2 | 712 | 351 | -1.02 | 3.68373E-06 | down |
| MELO3C003760.2 | 110 | 564 | 2.36 | 3.31513E-49 | up |
| MELO3C003761.2 | 71 | 5 | -3.766 | 2.32669E-11 | down |
| MELO3C003762.2 | 596 | 261 | -1.19 | 6.14674E-19 | down |
| MELO3C003764.2 | 449 | 2195 | 2.291 | 2.07457E-120 | up |
| MELO3C003778.2 | 1 | 8 | 3.449 | 0.024153936 | up |
| MELO3C003783.2 | 0 | 11 | 5.747 | 0.001038482 | up |
| MELO3C003788.2 | 4523 | 1327 | -1.769 | 2.11643E-44 | down |
| MELO3C003790.2 | 0 | 11 | 5.834 | 0.00043219 | up |
| MELO3C003791.2 | 370 | 146 | -1.339 | 1.14207E-09 | down |
| MELO3C003792.2 | 87 | 271 | 1.646 | 5.6348E-05 | up |
| MELO3C003793.2 | 3549 | 1543 | -1.202 | 1.03828E-48 | down |
| MELO3C003803.2 | 171 | 39 | -2.145 | 9.67627E-14 | down |
| MELO3C003811.2 | 2256 | 899 | -1.328 | 1.88613E-16 | down |
| MELO3C003817.2 | 6185 | 2997 | -1.045 | 2.42805E-08 | down |
| MELO3C003820.2 | 223 | 998 | 2.159 | 7.57277E-64 | up |
| MELO3C003821.2 | 46 | 312 | 2.769 | 5.37611E-30 | up |
| MELO3C003823.2 | 1957 | 94 | -4.388 | 5.60299E-25 | down |
| MELO3C003827.2 | 156 | 4 | -5.29 | 1.29169E-24 | down |
| MELO3C003838.2 | 27 | 12 | -1.167 | 0.024552141 | down |
| MELO3C003847.2 | 287 | 134 | -1.096 | 1.96268E-06 | down |
| MELO3C003852.2 | 46 | 279 | 2.596 | 2.99317E-14 | up |
| MELO3C003861.2 | 963 | 2371 | 1.3 | 2.74234E-25 | up |
| MELO3C003874.2 | 967 | 246 | -1.973 | 4.71838E-52 | down |
| MELO3C003878.2 | 1350 | 325 | -2.052 | 3.07555E-19 | down |
| MELO3C003879.2 | 35 | 90 | 1.343 | 9.93929E-05 | up |
| MELO3C003881.2 | 76 | 11 | -2.716 | 4.47112E-09 | down |
| MELO3C003884.2 | 57 | 29 | -1.006 | 0.022836084 | down |
| MELO3C003886.2 | 26 | 151 | 2.573 | 1.08438E-07 | up |
| MELO3C003889.2 | 3 | 33 | 3.335 | 9.26573E-05 | up |
| MELO3C003890.2 | 2634 | 857 | -1.62 | 2.86626E-26 | down |
| MELO3C003902.2 | 1 | 44 | 5.94 | 1.66766E-06 | up |
| MELO3C003911.2 | 56 | 461 | 3.044 | 5.87188E-36 | up |
| MELO3C003916.2 | 55 | 232 | 2.094 | 3.12615E-18 | up |
| MELO3C003917.2 | 193 | 52 | -1.874 | 1.58229E-09 | down |
| MELO3C003918.2 | 10 | 55 | 2.408 | 6.89632E-07 | up |
| MELO3C003919.2 | 19 | 4 | -2.148 | 0.002492328 | down |
| MELO3C003929.2 | 640 | 1378 | 1.104 | 7.151E-26 | up |
| MELO3C003930.2 | 0 | 14 | 6.195 | 3.7512E-05 | up |
| MELO3C003932.2 | 0 | 4 | 4.422 | 0.029266551 | up |
| MELO3C003933.2 | 2 | 34 | 3.773 | 6.55049E-06 | up |
| MELO3C003934.2 | 136 | 44 | -1.632 | 4.99339E-05 | down |
| MELO3C003942.2 | 1318 | 577 | -1.193 | 5.68354E-37 | down |
| MELO3C003945.2 | 305 | 620 | 1.024 | 1.78492E-14 | up |
| MELO3C003975.2 | 2000 | 681 | -1.557 | 2.00252E-32 | down |
| MELO3C003980.2 | 268 | 3927 | 3.873 | 2.22566E-100 | up |
| MELO3C003990.2 | 2172 | 660 | -1.72 | 4.16355E-22 | down |
| MELO3C003992.2 | 51 | 150 | 1.55 | 6.44326E-05 | up |
| MELO3C004003.2 | 3207 | 336 | -3.256 | 1.9282E-64 | down |
| MELO3C004039.2 | 168 | 39 | -2.108 | 1.60155E-11 | down |
| MELO3C004040.2 | 4146 | 1308 | -1.665 | 3.83518E-48 | down |
| MELO3C004059.2 | 11 | 88 | 2.943 | 3.09458E-08 | up |
| MELO3C004065.2 | 913 | 2491 | 1.449 | 1.75158E-27 | up |
| MELO3C004075.2 | 1524 | 3232 | 1.084 | 1.28445E-28 | up |
| MELO3C004078.2 | 5 | 164 | 5.032 | 2.83769E-16 | up |
| MELO3C004086.2 | 220 | 103 | -1.087 | 1.02E-07 | down |
| MELO3C004090.2 | 1000 | 301 | -1.734 | 5.65606E-25 | down |
| MELO3C004096.2 | 3548 | 618 | -2.52 | 1.76704E-66 | down |
| MELO3C004107.2 | 1683 | 840 | -1.004 | 3.11761E-27 | down |
| MELO3C004116.2 | 3881 | 1503 | -1.369 | 1.28847E-37 | down |
| MELO3C004125.2 | 2135 | 901 | -1.245 | 2.83407E-26 | down |
| MELO3C004135.2 | 1 | 85 | 6.884 | 3.09084E-10 | up |
| MELO3C004137.2 | 2894 | 1243 | -1.219 | 1.04101E-20 | down |
| MELO3C004141.2 | 218 | 24 | -3.198 | 2.44193E-22 | down |
| MELO3C004142.2 | 223 | 20 | -3.519 | 1.55141E-36 | down |
| MELO3C004145.2 | 13 | 173 | 3.785 | 5.37864E-24 | up |
| MELO3C004155.2 | 44 | 953 | 4.448 | 4.53783E-63 | up |
| MELO3C004161.2 | 20 | 70 | 1.824 | 9.17933E-05 | up |
| MELO3C004172.2 | 730 | 1551 | 1.087 | 1.2829E-28 | up |
| MELO3C004175.2 | 47 | 107 | 1.177 | 6.56645E-05 | up |
| MELO3C004182.2 | 1629 | 625 | -1.381 | 2.78007E-17 | down |
| MELO3C004183.2 | 104 | 10 | -3.446 | 4.5606E-18 | down |
| MELO3C004189.2 | 284 | 910 | 1.682 | 8.64769E-27 | up |
| MELO3C004199.2 | 27 | 78 | 1.553 | 0.000160769 | up |
| MELO3C004207.2 | 4 | 47 | 3.588 | 2.88297E-06 | up |
| MELO3C004214.2 | 40950 | 55 | -9.521 | 0 | down |
| MELO3C004216.2 | 451 | 1689 | 1.904 | 2.43421E-68 | up |
| MELO3C004225.2 | 812 | 320 | -1.344 | 5.1809E-11 | down |
| MELO3C004226.2 | 111 | 51 | -1.097 | 0.000392525 | down |
| MELO3C004227.2 | 166 | 1072 | 2.695 | 2.17418E-30 | up |
| MELO3C004234.2 | 31 | 63 | 1.028 | 0.00539173 | up |
| MELO3C004244.2 | 637 | 17 | -5.22 | 2.20123E-34 | down |
| MELO3C004247.2 | 11 | 0 | -5.93 | 0.001268771 | down |
| MELO3C004261.2 | 1 | 254 | 7.886 | 1.91938E-19 | up |
| MELO3C004262.2 | 182 | 25 | -2.845 | 3.92114E-20 | down |
| MELO3C004265.2 | 0 | 68 | 8.435 | 2.69455E-11 | up |
| MELO3C004269.2 | 5 | 40 | 2.887 | 4.93909E-07 | up |
| MELO3C004278.2 | 691 | 1431 | 1.05 | 5.72364E-26 | up |
| MELO3C004281.2 | 111 | 846 | 2.934 | 2.72664E-74 | up |
| MELO3C004289.2 | 1984 | 0 | -13.506 | 6.02701E-30 | down |
| MELO3C004291.2 | 32 | 0 | -7.547 | 1.30921E-08 | down |
| MELO3C004296.2 | 5804 | 88 | -6.044 | 3.81759E-248 | down |
| MELO3C004301.2 | 569 | 2362 | 2.054 | 5.29167E-22 | up |
| MELO3C004303.2 | 359 | 1104 | 1.62 | 9.60824E-42 | up |
| MELO3C004307.2 | 6018 | 1668 | -1.851 | 8.53897E-46 | down |
| MELO3C004309.2 | 24 | 130 | 2.432 | 5.87476E-13 | up |
| MELO3C004313.2 | 195 | 16 | -3.531 | 9.79864E-20 | down |
| MELO3C004315.2 | 51 | 5 | -3.373 | 3.12894E-09 | down |
| MELO3C004316.2 | 2247 | 261 | -3.109 | 1.29727E-104 | down |
| MELO3C004317.2 | 114 | 835 | 2.876 | 1.78677E-68 | up |
| MELO3C004320.2 | 0 | 24 | 5.959 | 1.66458E-05 | up |
| MELO3C004321.2 | 280 | 1400 | 2.322 | 5.8322E-64 | up |
| MELO3C004342.2 | 261 | 540 | 1.045 | 1.67412E-16 | up |
| MELO3C004346.2 | 470 | 136 | -1.792 | 2.68951E-24 | down |
| MELO3C004351.2 | 1467 | 15 | -6.528 | 6.89809E-149 | down |
| MELO3C004358.2 | 1690 | 393 | -2.105 | 1.17032E-34 | down |
| MELO3C004359.2 | 7 | 27 | 1.934 | 0.002250479 | up |
| MELO3C004362.2 | 8 | 60 | 2.814 | 4.10123E-05 | up |
| MELO3C004364.2 | 2 | 14 | 2.739 | 0.003481542 | up |
| MELO3C004365.2 | 31 | 123 | 2.018 | 3.18749E-10 | up |
| MELO3C004367.2 | 120 | 396 | 1.72 | 2.75363E-24 | up |
| MELO3C004373.2 | 399 | 837 | 1.067 | 9.26454E-05 | up |
| MELO3C004378.2 | 564 | 150 | -1.911 | 4.67968E-26 | down |
| MELO3C004379.2 | 53 | 177 | 1.742 | 2.96098E-13 | up |
| MELO3C004382.2 | 420 | 1061 | 1.338 | 3.3157E-12 | up |
| MELO3C004383.2 | 3637 | 1324 | -1.458 | 3.82354E-38 | down |
| MELO3C004388.2 | 2491 | 134 | -4.219 | 5.27988E-114 | down |
| MELO3C004396.2 | 302 | 1190 | 1.978 | 1.21856E-15 | up |
| MELO3C004407.2 | 90 | 206 | 1.194 | 5.65641E-06 | up |
| MELO3C004425.2 | 116 | 279 | 1.278 | 2.9302E-10 | up |
| MELO3C004426.2 | 79 | 223 | 1.497 | 7.14349E-08 | up |
| MELO3C004428.2 | 6324 | 2495 | -1.342 | 7.97071E-05 | down |
| MELO3C004430.2 | 4742 | 2333 | -1.023 | 7.45347E-08 | down |
| MELO3C004435.2 | 8579 | 1658 | -2.37 | 1.05644E-63 | down |
| MELO3C004437.2 | 1191 | 517 | -1.203 | 2.65408E-13 | down |
| MELO3C004442.2 | 1 | 8 | 3.573 | 0.016705372 | up |
| MELO3C004445.2 | 504 | 1155 | 1.196 | 1.14892E-17 | up |
| MELO3C004448.2 | 93 | 493 | 2.418 | 5.67571E-17 | up |
| MELO3C004449.2 | 146 | 31 | -2.204 | 7.31742E-13 | down |
| MELO3C004455.2 | 19 | 3 | -2.581 | 0.005929788 | down |
| MELO3C004456.2 | 16 | 3 | -2.655 | 0.004912649 | down |
| MELO3C004457.2 | 376 | 177 | -1.088 | 1.43424E-08 | down |
| MELO3C004465.2 | 190 | 86 | -1.137 | 4.26971E-06 | down |
| MELO3C004466.2 | 432 | 1746 | 2.017 | 1.80533E-80 | up |
| MELO3C004467.2 | 385 | 1316 | 1.776 | 2.63728E-63 | up |
| MELO3C004474.2 | 124 | 674 | 2.449 | 7.07565E-32 | up |
| MELO3C004478.2 | 178 | 671 | 1.913 | 4.99773E-33 | up |
| MELO3C004489.2 | 70 | 30 | -1.226 | 0.00177258 | down |
| MELO3C004495.2 | 116 | 3 | -5.23 | 5.87527E-21 | down |
| MELO3C004502.2 | 18 | 4 | -2.284 | 0.003442976 | down |
| MELO3C004504.2 | 1834 | 503 | -1.867 | 6.09633E-56 | down |
| MELO3C004506.2 | 2313 | 568 | -2.025 | 2.18137E-30 | down |
| MELO3C004507.2 | 1920 | 960 | -1.001 | 1.63131E-18 | down |
| MELO3C004511.2 | 14 | 41 | 1.572 | 0.00080936 | up |
| MELO3C004513.2 | 13 | 58 | 2.135 | 0.001677299 | up |
| MELO3C004515.2 | 10 | 71 | 2.777 | 7.01385E-08 | up |
| MELO3C004520.2 | 321 | 56 | -2.532 | 1.9566E-15 | down |
| MELO3C004522.2 | 20 | 131 | 2.685 | 6.29099E-15 | up |
| MELO3C004523.2 | 399 | 148 | -1.426 | 2.49629E-10 | down |
| MELO3C004524.2 | 130 | 537 | 2.054 | 1.9338E-38 | up |
| MELO3C004527.2 | 38 | 336 | 3.152 | 6.18084E-17 | up |
| MELO3C004529.2 | 651 | 111 | -2.563 | 2.81655E-28 | down |
| MELO3C004533.2 | 366 | 837 | 1.196 | 4.72343E-16 | up |
| MELO3C004535.2 | 364 | 74 | -2.29 | 1.36858E-21 | down |
| MELO3C004536.2 | 88 | 597 | 2.761 | 6.64327E-52 | up |
| MELO3C004538.2 | 1138 | 2688 | 1.24 | 1.52582E-19 | up |
| MELO3C004539.2 | 262 | 939 | 1.841 | 1.25273E-41 | up |
| MELO3C004543.2 | 262 | 81 | -1.693 | 2.79229E-10 | down |
| MELO3C004544.2 | 386 | 105 | -1.876 | 6.02902E-20 | down |
| MELO3C004549.2 | 212 | 682 | 1.683 | 1.56233E-29 | up |
| MELO3C004550.2 | 812 | 3061 | 1.915 | 3.91212E-40 | up |
| MELO3C004551.2 | 3344 | 298 | -3.49 | 2.20567E-170 | down |
| MELO3C004552.2 | 415 | 163 | -1.345 | 3.34408E-11 | down |
| MELO3C004553.2 | 1179 | 24 | -5.567 | 4.62636E-155 | down |
| MELO3C004554.2 | 79 | 11 | -2.849 | 9.70067E-08 | down |
| MELO3C004555.2 | 1769 | 677 | -1.384 | 2.08821E-16 | down |
| MELO3C004556.2 | 2627 | 538 | -2.286 | 1.70778E-38 | down |
| MELO3C004562.2 | 9 | 22 | 1.455 | 0.017857535 | up |
| MELO3C004574.2 | 3466 | 1442 | -1.265 | 6.09642E-24 | down |
| MELO3C004578.2 | 940 | 464 | -1.017 | 1.11297E-19 | down |
| MELO3C004579.2 | 13 | 458 | 5.184 | 1.77703E-06 | up |
| MELO3C004582.2 | 8 | 41 | 2.369 | 7.94706E-06 | up |
| MELO3C004586.2 | 78 | 1 | -6.992 | 1.73667E-10 | down |
| MELO3C004593.2 | 35 | 3 | -3.709 | 5.90299E-06 | down |
| MELO3C004597.2 | 175 | 579 | 1.725 | 4.57811E-15 | up |
| MELO3C004604.2 | 70 | 19 | -1.881 | 8.25516E-06 | down |
| MELO3C004609.2 | 258 | 91 | -1.498 | 2.2562E-05 | down |
| MELO3C004620.2 | 1 | 14 | 4.295 | 0.001348801 | up |
| MELO3C004624.2 | 147 | 1188 | 3.015 | 4.77771E-61 | up |
| MELO3C004633.2 | 289 | 4 | -6.085 | 3.4467E-29 | down |
| MELO3C004637.2 | 1634 | 765 | -1.094 | 2.35589E-34 | down |
| MELO3C004640.2 | 2269 | 608 | -1.901 | 2.40819E-72 | down |
| MELO3C004641.2 | 46 | 288 | 2.63 | 4.90845E-31 | up |
| MELO3C004642.2 | 4328 | 130 | -5.055 | 1.70175E-21 | down |
| MELO3C004647.2 | 82 | 170 | 1.056 | 0.001191707 | up |
| MELO3C004655.2 | 303 | 622 | 1.04 | 1.00934E-09 | up |
| MELO3C004657.2 | 33 | 140 | 2.09 | 5.31137E-13 | up |
| MELO3C004659.2 | 1802 | 3846 | 1.093 | 1.22488E-33 | up |
| MELO3C004661.2 | 35 | 73 | 1.073 | 0.005371689 | up |
| MELO3C004670.2 | 1 | 8 | 2.587 | 0.024733168 | up |
| MELO3C004685.2 | 107 | 25 | -2.125 | 1.33365E-06 | down |
| MELO3C004704.2 | 900 | 1950 | 1.115 | 7.00401E-17 | up |
| MELO3C004719.2 | 46 | 13 | -1.817 | 0.000311075 | down |
| MELO3C004728.2 | 92 | 19 | -2.245 | 5.75825E-08 | down |
| MELO3C004732.2 | 1020 | 225 | -2.181 | 8.6717E-52 | down |
| MELO3C004742.2 | 165 | 20 | -3.062 | 9.75834E-20 | down |
| MELO3C004761.2 | 4614 | 1358 | -1.765 | 1.95187E-35 | down |
| MELO3C004787.2 | 265 | 810 | 1.611 | 8.54938E-40 | up |
| MELO3C004795.2 | 688 | 320 | -1.102 | 3.5183E-08 | down |
| MELO3C004801.2 | 1651 | 46 | -5.157 | 6.68189E-123 | down |
| MELO3C004818.2 | 322 | 645 | 1.004 | 4.43969E-12 | up |
| MELO3C004850.2 | 0 | 782 | 11.952 | 4.76899E-17 | up |
| MELO3C004859.2 | 0 | 6 | 4.857 | 0.009498402 | up |
| MELO3C004866.2 | 1287 | 2853 | 1.149 | 2.97967E-16 | up |
| MELO3C004871.2 | 2057 | 359 | -2.521 | 9.78152E-103 | down |
| MELO3C004903.2 | 911 | 387 | -1.235 | 8.58767E-18 | down |
| MELO3C004914.2 | 63 | 0 | -8.542 | 4.21344E-11 | down |
| MELO3C004936.2 | 99 | 341 | 1.789 | 1.24893E-15 | up |
| MELO3C004941.2 | 1 | 8 | 3.466 | 0.021899729 | up |
| MELO3C004946.2 | 135 | 65 | -1.049 | 0.000484557 | down |
| MELO3C004957.2 | 43 | 119 | 1.481 | 1.16286E-05 | up |
| MELO3C004974.2 | 10 | 0 | -5.872 | 0.000785722 | down |
| MELO3C004975.2 | 6 | 104 | 4.014 | 5.5015E-16 | up |
| MELO3C004981.2 | 1 | 42 | 5.276 | 1.44748E-07 | up |
| MELO3C004982.2 | 14 | 3 | -2.177 | 0.022511182 | down |
| MELO3C004995.2 | 1818 | 687 | -1.404 | 4.36339E-15 | down |
| MELO3C004998.2 | 2462 | 497 | -2.309 | 7.28884E-29 | down |
| MELO3C004999.2 | 0 | 2596 | 13.682 | 6.7848E-31 | up |
| MELO3C005002.2 | 316 | 127 | -1.314 | 5.15682E-13 | down |
| MELO3C005006.2 | 1387 | 338 | -2.038 | 1.27732E-45 | down |
| MELO3C005009.2 | 15 | 58 | 1.951 | 3.85147E-06 | up |
| MELO3C005013.2 | 81 | 11 | -2.885 | 1.95417E-12 | down |
| MELO3C005044.2 | 23 | 1 | -5.236 | 2.38072E-05 | down |
| MELO3C005054.2 | 534 | 1174 | 1.136 | 1.30884E-17 | up |
| MELO3C005065.2 | 30 | 10 | -1.574 | 0.005398217 | down |
| MELO3C005070.2 | 22 | 84 | 1.93 | 8.21662E-08 | up |
| MELO3C005084.2 | 8014 | 3166 | -1.34 | 8.31854E-57 | down |
| MELO3C005085.2 | 42 | 118 | 1.5 | 2.11297E-05 | up |
| MELO3C005088.2 | 14 | 0 | -6.388 | 3.60273E-05 | down |
| MELO3C005095.2 | 107 | 572 | 2.423 | 3.64105E-37 | up |
| MELO3C005101.2 | 1925 | 543 | -1.825 | 2.3567E-26 | down |
| MELO3C005129.2 | 889 | 344 | -1.369 | 9.27944E-17 | down |
| MELO3C005130.2 | 2023 | 663 | -1.608 | 3.29023E-30 | down |
| MELO3C005131.2 | 6703 | 3156 | -1.086 | 1.76283E-30 | down |
| MELO3C005132.2 | 0 | 8 | 5.333 | 0.002628693 | up |
| MELO3C005137.2 | 4 | 108 | 4.657 | 2.47551E-16 | up |
| MELO3C005145.2 | 0 | 40 | 7.645 | 4.04371E-09 | up |
| MELO3C005147.2 | 20 | 259 | 3.663 | 1.2365E-14 | up |
| MELO3C005148.2 | 8 | 135 | 4.104 | 9.16021E-21 | up |
| MELO3C005149.2 | 972 | 459 | -1.082 | 1.60618E-09 | down |
| MELO3C005156.2 | 66 | 1136 | 4.113 | 1.61602E-47 | up |
| MELO3C005173.2 | 1269 | 581 | -1.127 | 1.89142E-07 | down |
| MELO3C005190.2 | 18 | 1 | -3.829 | 0.000264668 | down |
| MELO3C005204.2 | 45 | 2 | -4.212 | 1.19782E-08 | down |
| MELO3C005206.2 | 605 | 1469 | 1.279 | 5.98314E-10 | up |
| MELO3C005212.2 | 0 | 56 | 8.142 | 9.75433E-11 | up |
| MELO3C005214.2 | 5409 | 441 | -3.615 | 1.09676E-16 | down |
| MELO3C005215.2 | 68 | 2892 | 5.396 | 2.21311E-11 | up |
| MELO3C005226.2 | 841 | 4674 | 2.475 | 2.22193E-112 | up |
| MELO3C005227.2 | 29 | 7 | -2.128 | 0.010123338 | down |
| MELO3C005229.2 | 459 | 123 | -1.904 | 1.47022E-22 | down |
| MELO3C005231.2 | 3026 | 509 | -2.571 | 2.49701E-37 | down |
| MELO3C005241.2 | 4229 | 8545 | 1.015 | 1.31839E-39 | up |
| MELO3C005245.2 | 8649 | 4077 | -1.085 | 4.0006E-15 | down |
| MELO3C005250.2 | 413 | 3738 | 3.18 | 3.19774E-163 | up |
| MELO3C005256.2 | 4 | 30 | 2.916 | 3.57025E-05 | up |
| MELO3C005258.2 | 105 | 12 | -3.143 | 1.82516E-13 | down |
| MELO3C005262.2 | 78 | 249 | 1.676 | 4.84159E-13 | up |
| MELO3C005278.2 | 0 | 7 | 5.205 | 0.002702976 | up |
| MELO3C005288.2 | 1415 | 686 | -1.042 | 3.8023E-20 | down |
| MELO3C005291.2 | 73 | 15 | -2.27 | 1.01375E-08 | down |
| MELO3C005297.2 | 1053 | 180 | -2.549 | 1.90348E-25 | down |
| MELO3C005298.2 | 1758 | 3664 | 1.059 | 3.33336E-25 | up |
| MELO3C005310.2 | 2516 | 146 | -4.108 | 2.16032E-151 | down |
| MELO3C005321.2 | 285 | 58 | -2.306 | 8.3804E-20 | down |
| MELO3C005330.2 | 13 | 84 | 2.669 | 1.27989E-07 | up |
| MELO3C005332.2 | 1664 | 189 | -3.133 | 2.25477E-129 | down |
| MELO3C005355.2 | 332 | 4 | -6.483 | 8.0141E-39 | down |
| MELO3C005359.2 | 250 | 751 | 1.585 | 9.97106E-27 | up |
| MELO3C005361.2 | 36 | 118 | 1.711 | 6.24349E-08 | up |
| MELO3C005363.2 | 682 | 97 | -2.803 | 8.99675E-46 | down |
| MELO3C005368.2 | 7858 | 19092 | 1.281 | 2.16165E-24 | up |
| MELO3C005369.2 | 0 | 5 | 4.657 | 0.014839997 | up |
| MELO3C005373.2 | 22 | 5 | -2.136 | 0.010785272 | down |
| MELO3C005375.2 | 6 | 58 | 3.262 | 5.33141E-10 | up |
| MELO3C005381.2 | 38 | 113 | 1.596 | 1.51328E-07 | up |
| MELO3C005397.2 | 127 | 20 | -2.658 | 2.33649E-11 | down |
| MELO3C005401.2 | 1519 | 3522 | 1.213 | 5.36276E-34 | up |
| MELO3C005403.2 | 41 | 3 | -3.724 | 9.47784E-09 | down |
| MELO3C005404.2 | 187 | 1564 | 3.06 | 1.58077E-92 | up |
| MELO3C005406.2 | 9 | 50 | 2.416 | 0.000474637 | up |
| MELO3C005408.2 | 3 | 33 | 3.504 | 1.24402E-06 | up |
| MELO3C005410.2 | 5015 | 1229 | -2.03 | 7.37423E-55 | down |
| MELO3C005414.2 | 60 | 356 | 2.557 | 1.63441E-24 | up |
| MELO3C005415.2 | 639 | 3593 | 2.49 | 5.70385E-108 | up |
| MELO3C005426.2 | 461 | 1511 | 1.712 | 2.23772E-35 | up |
| MELO3C005435.2 | 27 | 0 | -7.302 | 7.93045E-08 | down |
| MELO3C005436.2 | 699 | 6 | -6.823 | 6.97309E-82 | down |
| MELO3C005437.2 | 5459 | 3 | -10.931 | 3.28277E-49 | down |
| MELO3C005442.2 | 1409 | 192 | -2.87 | 7.2092E-47 | down |
| MELO3C005449.2 | 4869 | 1907 | -1.352 | 1.4672E-58 | down |
| MELO3C005451.2 | 0 | 5 | 4.604 | 0.027782272 | up |
| MELO3C005455.2 | 55 | 149 | 1.428 | 3.72583E-07 | up |
| MELO3C005458.2 | 258 | 1102 | 2.097 | 1.5746E-34 | up |
| MELO3C005460.2 | 148 | 401 | 1.44 | 2.11338E-19 | up |
| MELO3C005462.2 | 4129 | 364 | -3.507 | 2.86694E-88 | down |
| MELO3C005463.2 | 25 | 2 | -3.498 | 0.007549507 | down |
| MELO3C005464.2 | 509 | 1801 | 1.823 | 2.31085E-39 | up |
| MELO3C005476.2 | 0 | 15 | 6.263 | 2.54082E-05 | up |
| MELO3C005484.2 | 133 | 407 | 1.609 | 9.31536E-25 | up |
| MELO3C005485.2 | 524 | 2497 | 2.253 | 3.77076E-69 | up |
| MELO3C005487.2 | 5 | 62 | 3.548 | 1.91058E-09 | up |
| MELO3C005488.2 | 2239 | 633 | -1.823 | 2.49577E-51 | down |
| MELO3C005492.2 | 82 | 24 | -1.787 | 3.67705E-07 | down |
| MELO3C005495.2 | 219 | 81 | -1.43 | 2.01395E-10 | down |
| MELO3C005498.2 | 0 | 9 | 5.507 | 0.002628383 | up |
| MELO3C005506.2 | 193 | 74 | -1.391 | 2.2004E-09 | down |
| MELO3C005510.2 | 21 | 213 | 3.384 | 3.68792E-31 | up |
| MELO3C005521.2 | 94 | 198 | 1.073 | 5.37811E-07 | up |
| MELO3C005524.2 | 140 | 387 | 1.467 | 3.12963E-18 | up |
| MELO3C005526.2 | 1213 | 388 | -1.644 | 0.008103875 | down |
| MELO3C005527.2 | 143 | 311 | 1.122 | 9.87008E-07 | up |
| MELO3C005528.2 | 141 | 60 | -1.241 | 0.000136091 | down |
| MELO3C005530.2 | 315 | 106 | -1.575 | 5.464E-16 | down |
| MELO3C005539.2 | 103 | 737 | 2.843 | 8.08018E-65 | up |
| MELO3C005540.2 | 89 | 3837 | 5.429 | 1.06733E-31 | up |
| MELO3C005556.2 | 927 | 13 | -6.165 | 1.97149E-104 | down |
| MELO3C005558.2 | 10605 | 2991 | -1.826 | 8.48712E-52 | down |
| MELO3C005559.2 | 4543 | 1398 | -1.701 | 1.87874E-42 | down |
| MELO3C005562.2 | 2096 | 516 | -2.021 | 4.89241E-39 | down |
| MELO3C005564.2 | 2805 | 277 | -3.339 | 3.8678E-51 | down |
| MELO3C005565.2 | 10983 | 2865 | -1.939 | 3.16242E-28 | down |
| MELO3C005566.2 | 9 | 69 | 2.832 | 2.72844E-08 | up |
| MELO3C005568.2 | 2408 | 662 | -1.864 | 6.12966E-25 | down |
| MELO3C005571.2 | 699 | 4949 | 2.825 | 1.78759E-37 | up |
| MELO3C005574.2 | 37 | 11 | -1.767 | 0.002830052 | down |
| MELO3C005576.2 | 0 | 23 | 6.845 | 1.81666E-06 | up |
| MELO3C005577.2 | 10337 | 4146 | -1.318 | 3.95122E-19 | down |
| MELO3C005581.2 | 684 | 175 | -1.961 | 1.54402E-19 | down |
| MELO3C005583.2 | 438 | 206 | -1.089 | 2.72349E-05 | down |
| MELO3C005585.2 | 226 | 654 | 1.532 | 8.05048E-16 | up |
| MELO3C005592.2 | 243 | 43 | -2.47 | 4.98281E-10 | down |
| MELO3C005600.2 | 869 | 1978 | 1.186 | 1.24919E-26 | up |
| MELO3C005601.2 | 86 | 38 | -1.151 | 0.006908332 | down |
| MELO3C005602.2 | 91 | 1634 | 4.161 | 7.7498E-20 | up |
| MELO3C005607.2 | 0 | 450 | 11.153 | 1.44165E-20 | up |
| MELO3C005608.2 | 152 | 499 | 1.714 | 4.57017E-26 | up |
| MELO3C005609.2 | 53 | 650 | 3.619 | 2.02058E-90 | up |
| MELO3C005611.2 | 0 | 6 | 5 | 0.008124363 | up |
| MELO3C005616.2 | 9365 | 3523 | -1.41 | 9.75713E-20 | down |
| MELO3C005623.2 | 4 | 32 | 2.885 | 5.66589E-05 | up |
| MELO3C005632.2 | 61 | 20 | -1.579 | 0.006212989 | down |
| MELO3C005635.2 | 4 | 29 | 2.909 | 0.004096665 | up |
| MELO3C005636.2 | 0 | 10 | 4.762 | 0.001681824 | up |
| MELO3C005640.2 | 1720 | 844 | -1.027 | 6.09757E-17 | down |
| MELO3C005652.2 | 679 | 7 | -6.526 | 1.27607E-81 | down |
| MELO3C005653.2 | 36 | 88 | 1.294 | 5.08255E-05 | up |
| MELO3C005654.2 | 0 | 10 | 5.696 | 0.000589159 | up |
| MELO3C005657.2 | 59 | 12 | -2.307 | 3.72179E-07 | down |
| MELO3C005658.2 | 13 | 1 | -4.389 | 0.001370428 | down |
| MELO3C005660.2 | 722 | 169 | -2.094 | 1.17988E-38 | down |
| MELO3C005663.2 | 4533 | 2047 | -1.147 | 3.0065E-33 | down |
| MELO3C005665.2 | 7 | 63 | 3.2 | 5.98199E-08 | up |
| MELO3C005667.2 | 548 | 1489 | 1.441 | 4.80329E-33 | up |
| MELO3C005672.2 | 1145 | 2858 | 1.32 | 3.61222E-55 | up |
| MELO3C005685.2 | 14585 | 3778 | -1.949 | 1.95351E-131 | down |
| MELO3C005686.2 | 32 | 182 | 2.516 | 0.024476561 | up |
| MELO3C005688.2 | 21352 | 9625 | -1.149 | 3.25847E-45 | down |
| MELO3C005696.2 | 530 | 1247 | 1.235 | 6.68018E-23 | up |
| MELO3C005701.2 | 4 | 832 | 7.598 | 3.42745E-59 | up |
| MELO3C005711.2 | 869 | 2192 | 1.336 | 1.61895E-20 | up |
| MELO3C005714.2 | 68 | 357 | 2.391 | 6.10368E-33 | up |
| MELO3C005716.2 | 215 | 0 | -10.303 | 9.49775E-18 | down |
| MELO3C005717.2 | 1369 | 549 | -1.32 | 2.31813E-21 | down |
| MELO3C005729.2 | 544 | 243 | -1.164 | 1.31263E-12 | down |
| MELO3C005732.2 | 56 | 395 | 2.821 | 1.46822E-34 | up |
| MELO3C005734.2 | 22 | 3 | -3.06 | 0.001005463 | down |
| MELO3C005736.2 | 9507 | 3364 | -1.499 | 2.01919E-17 | down |
| MELO3C005737.2 | 388 | 103 | -1.921 | 2.8619E-27 | down |
| MELO3C005739.2 | 387 | 1148 | 1.57 | 1.22957E-31 | up |
| MELO3C005741.2 | 147 | 57 | -1.368 | 2.99006E-08 | down |
| MELO3C005746.2 | 274 | 815 | 1.573 | 3.7887E-22 | up |
| MELO3C005748.2 | 56 | 818 | 3.888 | 3.21111E-38 | up |
| MELO3C005751.2 | 733 | 202 | -1.86 | 1.07374E-29 | down |
| MELO3C005752.2 | 65 | 2 | -5.117 | 1.84874E-06 | down |
| MELO3C005759.2 | 2765 | 799 | -1.791 | 3.50622E-30 | down |
| MELO3C005763.2 | 12953 | 1143 | -3.503 | 1.27044E-96 | down |
| MELO3C005764.2 | 661 | 130 | -2.348 | 7.58848E-24 | down |
| MELO3C005767.2 | 209 | 1066 | 2.354 | 3.19512E-69 | up |
| MELO3C005769.2 | 285 | 1397 | 2.293 | 9.05952E-73 | up |
| MELO3C005783.2 | 117 | 26 | -2.161 | 1.80121E-07 | down |
| MELO3C005789.2 | 8 | 212 | 4.821 | 6.32306E-32 | up |
| MELO3C005792.2 | 317 | 682 | 1.103 | 1.86319E-05 | up |
| MELO3C005796.2 | 1582 | 292 | -2.44 | 1.75238E-38 | down |
| MELO3C005799.2 | 596 | 1223 | 1.038 | 4.34955E-21 | up |
| MELO3C005800.2 | 2584 | 703 | -1.878 | 1.98393E-31 | down |
| MELO3C005801.2 | 433 | 201 | -1.109 | 1.72746E-06 | down |
| MELO3C005803.2 | 85 | 214 | 1.336 | 4.91895E-09 | up |
| MELO3C005805.2 | 118 | 22 | -2.403 | 6.25353E-08 | down |
| MELO3C005806.2 | 1 | 67 | 6.544 | 3.98287E-09 | up |
| MELO3C005811.2 | 2918 | 441 | -2.728 | 5.74864E-73 | down |
| MELO3C005813.2 | 1061 | 4880 | 2.201 | 4.79584E-67 | up |
| MELO3C005816.2 | 72 | 617 | 3.104 | 1.26927E-42 | up |
| MELO3C005818.2 | 134 | 359 | 1.421 | 0.000208178 | up |
| MELO3C005819.2 | 86 | 425 | 2.302 | 2.95921E-30 | up |
| MELO3C005821.2 | 541 | 185 | -1.555 | 1.11016E-20 | down |
| MELO3C005822.2 | 5 | 32 | 2.483 | 4.31031E-05 | up |
| MELO3C005829.2 | 3721 | 683 | -2.447 | 1.23098E-42 | down |
| MELO3C005831.2 | 0 | 10 | 5.694 | 0.000817193 | up |
| MELO3C005832.2 | 53 | 22 | -1.274 | 0.004515443 | down |
| MELO3C005834.2 | 6913 | 2416 | -1.517 | 3.42337E-31 | down |
| MELO3C005847.2 | 231 | 68 | -1.765 | 1.20128E-10 | down |
| MELO3C005849.2 | 101 | 220 | 1.124 | 6.57465E-06 | up |
| MELO3C005850.2 | 38 | 13 | -1.611 | 0.02178198 | down |
| MELO3C005853.2 | 39 | 99 | 1.323 | 0.000397057 | up |
| MELO3C005855.2 | 70 | 394 | 2.491 | 3.62531E-31 | up |
| MELO3C005858.2 | 29 | 204 | 2.825 | 3.86877E-07 | up |
| MELO3C005869.2 | 9 | 33 | 1.881 | 0.014015152 | up |
| MELO3C005874.2 | 914 | 4470 | 2.29 | 1.01036E-106 | up |
| MELO3C005878.2 | 27 | 66 | 1.274 | 0.001546352 | up |
| MELO3C005885.2 | 36 | 471 | 3.718 | 5.72535E-33 | up |
| MELO3C005888.2 | 9 | 110 | 3.621 | 2.09699E-11 | up |
| MELO3C005898.2 | 632 | 3099 | 2.295 | 2.14128E-77 | up |
| MELO3C005899.2 | 461 | 72 | -2.681 | 5.95613E-53 | down |
| MELO3C005902.2 | 310 | 47 | -2.719 | 1.07343E-13 | down |
| MELO3C005903.2 | 19 | 64 | 1.751 | 9.07011E-06 | up |
| MELO3C005911.2 | 1005 | 301 | -1.74 | 2.74408E-23 | down |
| MELO3C005912.2 | 258 | 563 | 1.13 | 2.09568E-13 | up |
| MELO3C005913.2 | 533 | 217 | -1.295 | 1.09794E-13 | down |
| MELO3C005914.2 | 602 | 100 | -2.6 | 1.28779E-35 | down |
| MELO3C005915.2 | 8481 | 2711 | -1.645 | 2.06075E-116 | down |
| MELO3C005921.2 | 2188 | 6260 | 1.517 | 1.3463E-47 | up |
| MELO3C005928.2 | 158 | 12 | -3.736 | 2.4401E-12 | down |
| MELO3C005937.2 | 36 | 113 | 1.662 | 2.04496E-08 | up |
| MELO3C005947.2 | 58 | 0 | -8.403 | 3.64644E-11 | down |
| MELO3C005949.2 | 908 | 242 | -1.903 | 1.82561E-35 | down |
| MELO3C005951.2 | 14 | 34 | 1.307 | 0.027834286 | up |
| MELO3C005960.2 | 150 | 376 | 1.327 | 3.62638E-07 | up |
| MELO3C005968.2 | 11 | 138 | 3.739 | 2.01199E-23 | up |
| MELO3C005972.2 | 6 | 103 | 4.083 | 1.98768E-11 | up |
| MELO3C005975.2 | 446 | 210 | -1.083 | 2.17305E-11 | down |
| MELO3C005979.2 | 688 | 253 | -1.443 | 1.10773E-14 | down |
| MELO3C005983.2 | 432 | 899 | 1.056 | 1.29293E-13 | up |
| MELO3C005987.2 | 6077 | 2397 | -1.342 | 5.32651E-64 | down |
| MELO3C005988.2 | 53 | 110 | 1.037 | 0.000148562 | up |
| MELO3C005991.2 | 1945 | 528 | -1.88 | 1.64032E-41 | down |
| MELO3C005992.2 | 6976 | 1133 | -2.622 | 2.27212E-154 | down |
| MELO3C005994.2 | 242 | 499 | 1.05 | 4.46351E-06 | up |
| MELO3C005995.2 | 29 | 2 | -3.966 | 6.91047E-06 | down |
| MELO3C005999.2 | 2397 | 1189 | -1.011 | 3.93236E-07 | down |
| MELO3C006000.2 | 1490 | 701 | -1.088 | 3.54118E-25 | down |
| MELO3C006001.2 | 987 | 142 | -2.799 | 1.80828E-33 | down |
| MELO3C006011.2 | 213 | 450 | 1.085 | 4.97477E-11 | up |
| MELO3C006014.2 | 50 | 22 | -1.238 | 0.002672628 | down |
| MELO3C006019.2 | 1664 | 4307 | 1.372 | 1.33674E-37 | up |
| MELO3C006020.2 | 305 | 1426 | 2.228 | 5.17822E-23 | up |
| MELO3C006025.2 | 334 | 1115 | 1.741 | 2.24095E-11 | up |
| MELO3C006028.2 | 12 | 46 | 1.9 | 0.004612043 | up |
| MELO3C006032.2 | 192 | 8 | -4.63 | 2.38654E-29 | down |
| MELO3C006035.2 | 12260 | 5777 | -1.086 | 5.9454E-17 | down |
| MELO3C006037.2 | 72 | 8 | -3.243 | 0.000734897 | down |
| MELO3C006039.2 | 59 | 19 | -1.591 | 0.002928192 | down |
| MELO3C006042.2 | 5205 | 1099 | -2.243 | 3.88299E-47 | down |
| MELO3C006043.2 | 116 | 32 | -1.865 | 4.28141E-05 | down |
| MELO3C006047.2 | 79 | 23 | -1.838 | 5.96549E-05 | down |
| MELO3C006053.2 | 7202 | 1311 | -2.458 | 6.88275E-41 | down |
| MELO3C006055.2 | 379 | 867 | 1.197 | 4.50479E-09 | up |
| MELO3C006056.2 | 226 | 1429 | 2.662 | 6.11609E-73 | up |
| MELO3C006057.2 | 7 | 27 | 2.052 | 0.001820241 | up |
| MELO3C006059.2 | 28 | 72 | 1.349 | 0.001094602 | up |
| MELO3C006062.2 | 7 | 56 | 3.036 | 5.38495E-06 | up |
| MELO3C006064.2 | 66 | 165 | 1.323 | 4.39742E-07 | up |
| MELO3C006066.2 | 8 | 37 | 2.165 | 0.000114209 | up |
| MELO3C006067.2 | 1 | 9 | 3.687 | 0.011835108 | up |
| MELO3C006072.2 | 13 | 51 | 1.974 | 2.3547E-05 | up |
| MELO3C006080.2 | 15 | 41 | 1.442 | 0.002041386 | up |
| MELO3C006088.2 | 2207 | 1033 | -1.096 | 4.75145E-17 | down |
| MELO3C006090.2 | 362 | 1010 | 1.483 | 1.39965E-15 | up |
| MELO3C006094.2 | 357 | 1213 | 1.766 | 2.4455E-37 | up |
| MELO3C006098.2 | 114 | 365 | 1.682 | 5.89819E-15 | up |
| MELO3C006099.2 | 22 | 191 | 3.134 | 4.44713E-28 | up |
| MELO3C006100.2 | 1335 | 644 | -1.051 | 1.00978E-18 | down |
| MELO3C006109.2 | 1278 | 2593 | 1.021 | 1.05243E-20 | up |
| MELO3C006117.2 | 208 | 529 | 1.343 | 2.89855E-15 | up |
| MELO3C006123.2 | 8816 | 2753 | -1.679 | 5.55723E-110 | down |
| MELO3C006139.2 | 12 | 0 | -6.084 | 0.021861953 | down |
| MELO3C006147.2 | 4965 | 1987 | -1.322 | 4.70734E-47 | down |
| MELO3C006151.2 | 651 | 299 | -1.121 | 1.34214E-13 | down |
| MELO3C006152.2 | 9 | 227 | 4.665 | 6.3467E-32 | up |
| MELO3C006155.2 | 1900 | 512 | -1.893 | 1.73597E-30 | down |
| MELO3C006159.2 | 296 | 41 | -2.846 | 1.1515E-30 | down |
| MELO3C006165.2 | 197 | 723 | 1.877 | 2.67214E-36 | up |
| MELO3C006166.2 | 388 | 1826 | 2.234 | 5.29746E-72 | up |
| MELO3C006169.2 | 2308 | 4921 | 1.092 | 2.41032E-18 | up |
| MELO3C006172.2 | 3264 | 472 | -2.791 | 1.26102E-152 | down |
| MELO3C006174.2 | 82 | 241 | 1.56 | 1.39689E-13 | up |
| MELO3C006180.2 | 281 | 1123 | 2.004 | 5.02443E-33 | up |
| MELO3C006182.2 | 320 | 146 | -1.125 | 2.29283E-12 | down |
| MELO3C006193.2 | 200 | 12 | -4.06 | 7.22383E-36 | down |
| MELO3C006195.2 | 1025 | 367 | -1.48 | 3.35019E-15 | down |
| MELO3C006200.2 | 10698 | 3927 | -1.446 | 3.37533E-44 | down |
| MELO3C006201.2 | 161 | 326 | 1.013 | 3.74759E-08 | up |
| MELO3C006202.2 | 1023 | 481 | -1.088 | 8.01266E-21 | down |
| MELO3C006220.2 | 68 | 168 | 1.304 | 0.002433397 | up |
| MELO3C006227.2 | 1238 | 560 | -1.144 | 1.68105E-27 | down |
| MELO3C006228.2 | 23 | 64 | 1.472 | 0.001023725 | up |
| MELO3C006246.2 | 2207 | 6459 | 1.549 | 2.16462E-84 | up |
| MELO3C006248.2 | 117 | 357 | 1.615 | 1.28264E-12 | up |
| MELO3C006252.2 | 120 | 342 | 1.51 | 1.77863E-05 | up |
| MELO3C006254.2 | 27349 | 2435 | -3.49 | 1.32431E-19 | down |
| MELO3C006256.2 | 184 | 466 | 1.338 | 5.18112E-18 | up |
| MELO3C006274.2 | 20 | 51 | 1.317 | 0.001845377 | up |
| MELO3C006285.2 | 841 | 403 | -1.061 | 2.25489E-20 | down |
| MELO3C006289.2 | 100 | 580 | 2.534 | 4.73548E-35 | up |
| MELO3C006291.2 | 4 | 0 | -4.655 | 0.020298081 | down |
| MELO3C006313.2 | 346 | 119 | -1.547 | 2.56668E-17 | down |
| MELO3C006321.2 | 1266 | 516 | -1.296 | 4.09341E-11 | down |
| MELO3C006326.2 | 24 | 49 | 1.028 | 0.012119525 | up |
| MELO3C006328.2 | 2028 | 4510 | 1.153 | 8.10318E-34 | up |
| MELO3C006334.2 | 8199 | 3612 | -1.182 | 4.62378E-60 | down |
| MELO3C006335.2 | 532 | 7 | -6.224 | 4.36287E-73 | down |
| MELO3C006341.2 | 8 | 50 | 2.716 | 2.94817E-07 | up |
| MELO3C006351.2 | 381 | 1108 | 1.541 | 1.02029E-31 | up |
| MELO3C006353.2 | 12 | 56 | 2.255 | 2.34465E-06 | up |
| MELO3C006358.2 | 3618 | 1441 | -1.328 | 4.50494E-21 | down |
| MELO3C006359.2 | 88 | 28 | -1.666 | 4.17522E-05 | down |
| MELO3C006373.2 | 3891 | 1281 | -1.603 | 2.44187E-39 | down |
| MELO3C006379.2 | 15 | 39 | 1.332 | 0.008349361 | up |
| MELO3C006380.2 | 128 | 271 | 1.081 | 1.30708E-08 | up |
| MELO3C006382.2 | 854 | 399 | -1.097 | 1.77568E-17 | down |
| MELO3C006392.2 | 159 | 378 | 1.245 | 3.93668E-07 | up |
| MELO3C006400.2 | 1742 | 784 | -1.151 | 2.18725E-12 | down |
| MELO3C006404.2 | 728 | 252 | -1.531 | 8.22678E-20 | down |
| MELO3C006405.2 | 1337 | 534 | -1.324 | 5.7274E-12 | down |
| MELO3C006406.2 | 91 | 192 | 1.071 | 0.000575285 | up |
| MELO3C006407.2 | 6654 | 2002 | -1.733 | 6.46275E-46 | down |
| MELO3C006410.2 | 453 | 119 | -1.929 | 7.7557E-22 | down |
| MELO3C006433.2 | 59 | 27 | -1.126 | 0.010483174 | down |
| MELO3C006436.2 | 233 | 82 | -1.504 | 1.57271E-13 | down |
| MELO3C006439.2 | 390 | 31 | -3.649 | 5.06146E-44 | down |
| MELO3C006443.2 | 2249 | 668 | -1.751 | 1.93396E-38 | down |
| MELO3C006444.2 | 1012 | 283 | -1.835 | 6.29059E-56 | down |
| MELO3C006446.2 | 26 | 56 | 1.095 | 0.004357558 | up |
| MELO3C006456.2 | 113 | 55 | -1.046 | 0.000574713 | down |
| MELO3C006464.2 | 157 | 15 | -3.379 | 0.004610122 | down |
| MELO3C006465.2 | 103 | 15 | -2.755 | 2.87799E-10 | down |
| MELO3C006467.2 | 97 | 7 | -3.768 | 3.74499E-15 | down |
| MELO3C006470.2 | 45 | 14 | -1.687 | 0.000222619 | down |
| MELO3C006473.2 | 1049 | 317 | -1.731 | 2.13527E-34 | down |
| MELO3C006475.2 | 4 | 22 | 2.342 | 0.001272595 | up |
| MELO3C006476.2 | 4025 | 10543 | 1.389 | 4.11429E-30 | up |
| MELO3C006477.2 | 1082 | 407 | -1.412 | 2.3259E-15 | down |
| MELO3C006482.2 | 238 | 73 | -1.714 | 1.08581E-08 | down |
| MELO3C006483.2 | 32 | 94 | 1.566 | 4.42449E-05 | up |
| MELO3C006489.2 | 280 | 133 | -1.073 | 6.34468E-06 | down |
| MELO3C006492.2 | 7 | 28 | 2.054 | 0.002216958 | up |
| MELO3C006501.2 | 1 | 10 | 3.153 | 0.013679222 | up |
| MELO3C006503.2 | 54 | 0 | -8.305 | 4.48059E-10 | down |
| MELO3C006506.2 | 89 | 0 | -9.035 | 2.16236E-13 | down |
| MELO3C006510.2 | 815 | 2311 | 1.504 | 5.31149E-39 | up |
| MELO3C006511.2 | 50 | 20 | -1.297 | 0.011185909 | down |
| MELO3C006519.2 | 221 | 85 | -1.371 | 2.97349E-06 | down |
| MELO3C006527.2 | 1993 | 751 | -1.409 | 4.47721E-48 | down |
| MELO3C006532.2 | 262 | 115 | -1.196 | 1.13029E-06 | down |
| MELO3C006533.2 | 669 | 255 | -1.393 | 1.72784E-12 | down |
| MELO3C006538.2 | 2 | 38 | 4.163 | 3.68831E-07 | up |
| MELO3C006539.2 | 25891 | 1293 | -4.324 | 1.249E-171 | down |
| MELO3C006546.2 | 236 | 1128 | 2.256 | 8.66348E-80 | up |
| MELO3C006553.2 | 213 | 729 | 1.773 | 3.5099E-38 | up |
| MELO3C006558.2 | 16784 | 35682 | 1.088 | 5.43757E-22 | up |
| MELO3C006561.2 | 120 | 0 | -9.464 | 1.19217E-14 | down |
| MELO3C006562.2 | 3566 | 1251 | -1.511 | 0.001613587 | down |
| MELO3C006564.2 | 131 | 1611 | 3.624 | 4.00664E-81 | up |
| MELO3C006569.2 | 1781 | 4046 | 1.184 | 2.73785E-42 | up |
| MELO3C006572.2 | 2622 | 81 | -5.025 | 7.4377E-17 | down |
| MELO3C006574.2 | 360 | 741 | 1.042 | 1.45012E-14 | up |
| MELO3C006577.2 | 7959 | 2975 | -1.42 | 2.31415E-24 | down |
| MELO3C006581.2 | 14 | 39 | 1.426 | 0.005654374 | up |
| MELO3C006585.2 | 13971 | 3497 | -1.998 | 3.26701E-44 | down |
| MELO3C006593.2 | 68 | 31 | -1.124 | 0.002163059 | down |
| MELO3C006602.2 | 124 | 935 | 2.921 | 3.99705E-57 | up |
| MELO3C006605.2 | 454 | 1163 | 1.358 | 6.28734E-33 | up |
| MELO3C006610.2 | 30 | 162 | 2.425 | 1.97083E-13 | up |
| MELO3C006611.2 | 81 | 272 | 1.748 | 1.26546E-11 | up |
| MELO3C006616.2 | 2367 | 1048 | -1.177 | 2.39125E-13 | down |
| MELO3C006620.2 | 30 | 0 | -7.447 | 3.48772E-08 | down |
| MELO3C006621.2 | 221 | 70 | -1.655 | 3.7118E-06 | down |
| MELO3C006644.2 | 3 | 19 | 2.55 | 0.004551274 | up |
| MELO3C006671.2 | 24 | 137 | 2.517 | 1.16618E-15 | up |
| MELO3C006674.2 | 1258 | 2638 | 1.068 | 2.62389E-10 | up |
| MELO3C006676.2 | 234 | 1194 | 2.351 | 3.67022E-34 | up |
| MELO3C006678.2 | 514 | 1 | -9.71 | 2.70839E-20 | down |
| MELO3C006679.2 | 1052 | 403 | -1.383 | 1.99988E-17 | down |
| MELO3C006682.2 | 245 | 81 | -1.594 | 5.89138E-07 | down |
| MELO3C006685.2 | 863 | 4 | -7.87 | 5.92336E-72 | down |
| MELO3C006688.2 | 32 | 1 | -4.71 | 2.15767E-06 | down |
| MELO3C006691.2 | 894 | 184 | -2.277 | 1.12446E-59 | down |
| MELO3C006695.2 | 16 | 285 | 4.148 | 3.74665E-38 | up |
| MELO3C006698.2 | 111 | 8 | -3.715 | 1.02015E-13 | down |
| MELO3C006700.2 | 112 | 0 | -9.364 | 1.0646E-06 | down |
| MELO3C006704.2 | 386 | 131 | -1.558 | 2.59052E-16 | down |
| MELO3C006715.2 | 4076 | 1920 | -1.086 | 8.85543E-24 | down |
| MELO3C006716.2 | 161 | 50 | -1.676 | 8.59087E-10 | down |
| MELO3C006721.2 | 720 | 143 | -2.332 | 1.99092E-59 | down |
| MELO3C006722.2 | 129 | 385 | 1.578 | 5.76742E-11 | up |
| MELO3C006733.2 | 4032 | 1091 | -1.886 | 1.03575E-50 | down |
| MELO3C006743.2 | 106 | 304 | 1.518 | 3.3968E-14 | up |
| MELO3C006744.2 | 61 | 146 | 1.246 | 3.84709E-06 | up |
| MELO3C006751.2 | 18 | 62 | 1.799 | 8.74478E-05 | up |
| MELO3C006755.2 | 3353 | 1296 | -1.371 | 1.24106E-48 | down |
| MELO3C006757.2 | 24 | 1 | -4.263 | 1.73198E-05 | down |
| MELO3C006763.2 | 875 | 86 | -3.346 | 2.5101E-101 | down |
| MELO3C006765.2 | 1715 | 608 | -1.496 | 5.89219E-47 | down |
| MELO3C006766.2 | 1493 | 339 | -2.142 | 1.43481E-31 | down |
| MELO3C006777.2 | 72 | 18 | -1.995 | 6.14011E-07 | down |
| MELO3C006779.2 | 13 | 3 | -2.219 | 0.026641439 | down |
| MELO3C006783.2 | 11208 | 5471 | -1.035 | 2.1679E-34 | down |
| MELO3C006789.2 | 676 | 2479 | 1.875 | 1.4191E-42 | up |
| MELO3C006800.2 | 4 | 173 | 5.459 | 3.78875E-27 | up |
| MELO3C006801.2 | 690 | 2198 | 1.671 | 1.90062E-35 | up |
| MELO3C006802.2 | 1355 | 529 | -1.36 | 9.38122E-26 | down |
| MELO3C006805.2 | 220 | 1962 | 3.159 | 2.75258E-125 | up |
| MELO3C006807.2 | 51 | 180 | 1.824 | 1.89738E-10 | up |
| MELO3C006808.2 | 196 | 408 | 1.058 | 3.08914E-10 | up |
| MELO3C006810.2 | 147 | 46 | -1.664 | 3.35793E-07 | down |
| MELO3C006811.2 | 910 | 2078 | 1.191 | 9.79822E-36 | up |
| MELO3C006813.2 | 24 | 59 | 1.267 | 0.001192715 | up |
| MELO3C006823.2 | 12 | 51 | 2.074 | 1.44859E-05 | up |
| MELO3C006827.2 | 120 | 1567 | 3.701 | 5.03526E-117 | up |
| MELO3C006833.2 | 57 | 171 | 1.594 | 2.71265E-07 | up |
| MELO3C006836.2 | 1 | 18 | 4.655 | 0.002666823 | up |
| MELO3C006843.2 | 2298 | 5209 | 1.18 | 0.005731643 | up |
| MELO3C006847.2 | 64 | 7 | -3.317 | 3.99803E-06 | down |
| MELO3C006851.2 | 17 | 276 | 4.001 | 4.76602E-13 | up |
| MELO3C006852.2 | 58 | 10 | -2.507 | 0.005231955 | down |
| MELO3C006854.2 | 13 | 39 | 1.513 | 0.022270149 | up |
| MELO3C006863.2 | 329 | 141 | -1.222 | 3.88751E-08 | down |
| MELO3C006865.2 | 53 | 306 | 2.529 | 2.15928E-32 | up |
| MELO3C006866.2 | 2335 | 609 | -1.939 | 7.55817E-77 | down |
| MELO3C006873.2 | 151 | 54 | -1.503 | 2.58544E-06 | down |
| MELO3C006885.2 | 1022 | 2085 | 1.029 | 6.46082E-13 | up |
| MELO3C006887.2 | 52 | 224 | 2.112 | 6.48717E-15 | up |
| MELO3C006888.2 | 409 | 93 | -2.124 | 6.76443E-14 | down |
| MELO3C006891.2 | 2107 | 842 | -1.324 | 3.90573E-49 | down |
| MELO3C006895.2 | 1792 | 771 | -1.217 | 7.13083E-39 | down |
| MELO3C006903.2 | 326 | 157 | -1.059 | 6.24312E-05 | down |
| MELO3C006909.2 | 41 | 1916 | 5.565 | 2.2249E-92 | up |
| MELO3C006917.2 | 90 | 748 | 3.048 | 1.62002E-31 | up |
| MELO3C006929.2 | 571 | 1372 | 1.264 | 1.4305E-24 | up |
| MELO3C006930.2 | 34 | 194 | 2.529 | 3.67997E-18 | up |
| MELO3C006933.2 | 14 | 117 | 3.061 | 1.51584E-17 | up |
| MELO3C006942.2 | 104 | 365 | 1.812 | 1.01768E-15 | up |
| MELO3C006944.2 | 44 | 8 | -2.565 | 4.44342E-06 | down |
| MELO3C006946.2 | 0 | 13 | 6.084 | 6.0759E-05 | up |
| MELO3C006956.2 | 10 | 31 | 1.695 | 0.001939203 | up |
| MELO3C006957.2 | 35 | 8 | -2.099 | 0.000276775 | down |
| MELO3C006958.2 | 488 | 28 | -4.123 | 2.0638E-38 | down |
| MELO3C006959.2 | 664 | 305 | -1.124 | 2.28789E-12 | down |
| MELO3C006965.2 | 108 | 49 | -1.144 | 0.005154886 | down |
| MELO3C006971.2 | 92 | 557 | 2.611 | 3.5134E-41 | up |
| MELO3C006973.2 | 27 | 200 | 2.901 | 2.51502E-25 | up |
| MELO3C006980.2 | 386 | 1179 | 1.609 | 5.84081E-31 | up |
| MELO3C006981.2 | 23 | 591 | 4.69 | 1.52128E-70 | up |
| MELO3C006993.2 | 909 | 2092 | 1.203 | 4.99526E-08 | up |
| MELO3C006995.2 | 136 | 820 | 2.593 | 9.68794E-35 | up |
| MELO3C006997.2 | 445 | 3615 | 3.024 | 4.39594E-101 | up |
| MELO3C007001.2 | 785 | 1742 | 1.15 | 3.72592E-10 | up |
| MELO3C007012.2 | 971 | 418 | -1.216 | 3.13938E-22 | down |
| MELO3C007014.2 | 824 | 315 | -1.388 | 4.0922E-16 | down |
| MELO3C007028.2 | 98 | 31 | -1.647 | 8.16693E-08 | down |
| MELO3C007037.2 | 7 | 1269 | 7.54 | 3.80832E-36 | up |
| MELO3C007038.2 | 27 | 370 | 3.78 | 1.3086E-45 | up |
| MELO3C007044.2 | 42 | 9 | -2.223 | 3.21348E-05 | down |
| MELO3C007046.2 | 72 | 7 | -3.283 | 2.41319E-11 | down |
| MELO3C007047.2 | 2116 | 958 | -1.142 | 7.95156E-16 | down |
| MELO3C007057.2 | 6 | 0 | -5.242 | 0.00546495 | down |
| MELO3C007058.2 | 14 | 78 | 2.483 | 2.44621E-10 | up |
| MELO3C007060.2 | 38 | 1 | -4.661 | 3.89568E-07 | down |
| MELO3C007069.2 | 12 | 36 | 1.563 | 0.002063928 | up |
| MELO3C007070.2 | 412 | 977 | 1.244 | 1.74467E-15 | up |
| MELO3C007072.2 | 10 | 1 | -3.018 | 0.010135243 | down |
| MELO3C007083.2 | 378 | 1222 | 1.693 | 3.44402E-25 | up |
| MELO3C007091.2 | 12799 | 1169 | -3.453 | 1.18388E-78 | down |
| MELO3C007093.2 | 11 | 124 | 3.433 | 5.61491E-17 | up |
| MELO3C007100.2 | 4366 | 2052 | -1.089 | 9.2198E-13 | down |
| MELO3C007102.2 | 29 | 60 | 1.062 | 0.008805859 | up |
| MELO3C007103.2 | 3487 | 1151 | -1.599 | 1.0605E-110 | down |
| MELO3C007120.2 | 992 | 2085 | 1.073 | 1.62276E-17 | up |
| MELO3C007124.2 | 15857 | 4963 | -1.676 | 1.82444E-50 | down |
| MELO3C007126.2 | 1136 | 562 | -1.015 | 1.00821E-18 | down |
| MELO3C007127.2 | 584 | 11 | -5.665 | 1.84459E-39 | down |
| MELO3C007132.2 | 38 | 0 | -7.809 | 2.41767E-09 | down |
| MELO3C007138.2 | 385 | 2921 | 2.925 | 1.07137E-62 | up |
| MELO3C007140.2 | 336 | 51 | -2.735 | 8.47662E-18 | down |
| MELO3C007144.2 | 127 | 657 | 2.368 | 1.82665E-16 | up |
| MELO3C007146.2 | 464 | 84 | -2.463 | 9.32663E-18 | down |
| MELO3C007147.2 | 419 | 127 | -1.722 | 2.36823E-22 | down |
| MELO3C007148.2 | 19 | 48 | 1.362 | 0.001156614 | up |
| MELO3C007152.2 | 59 | 3 | -4.116 | 4.6794E-12 | down |
| MELO3C007154.2 | 77645 | 365 | -7.736 | 0 | down |
| MELO3C007174.2 | 214 | 782 | 1.872 | 8.58508E-37 | up |
| MELO3C007180.2 | 1 | 11 | 2.886 | 0.020803676 | up |
| MELO3C007185.2 | 144 | 67 | -1.095 | 9.23534E-05 | down |
| MELO3C007203.2 | 4 | 27 | 2.893 | 0.000684078 | up |
| MELO3C007207.2 | 1267 | 4256 | 1.748 | 2.45927E-48 | up |
| MELO3C007215.2 | 30 | 135 | 2.186 | 5.4532E-10 | up |
| MELO3C007216.2 | 1 | 10 | 3.895 | 0.007389498 | up |
| MELO3C007217.2 | 2 | 13 | 2.662 | 0.015983186 | up |
| MELO3C007218.2 | 454 | 210 | -1.11 | 7.94914E-09 | down |
| MELO3C007219.2 | 94 | 356 | 1.922 | 8.88366E-15 | up |
| MELO3C007226.2 | 519 | 161 | -1.685 | 1.47158E-29 | down |
| MELO3C007230.2 | 10 | 41 | 1.984 | 0.000246266 | up |
| MELO3C007232.2 | 306 | 135 | -1.177 | 0.000120799 | down |
| MELO3C007233.2 | 8960 | 1630 | -2.458 | 1.198E-52 | down |
| MELO3C007236.2 | 371 | 751 | 1.018 | 1.35753E-08 | up |
| MELO3C007252.2 | 10 | 88 | 3.083 | 6.79367E-11 | up |
| MELO3C007257.2 | 37 | 1 | -5.903 | 3.18524E-07 | down |
| MELO3C007258.2 | 10 | 0 | -5.817 | 0.001016774 | down |
| MELO3C007259.2 | 17110 | 4348 | -1.976 | 2.62173E-42 | down |
| MELO3C007269.2 | 45680 | 14678 | -1.638 | 4.222E-128 | down |
| MELO3C007275.2 | 147 | 17 | -3.106 | 4.70217E-19 | down |
| MELO3C007280.2 | 6977 | 1691 | -2.045 | 3.65517E-127 | down |
| MELO3C007281.2 | 1970 | 3964 | 1.009 | 1.46984E-23 | up |
| MELO3C007285.2 | 1822 | 800 | -1.187 | 1.28556E-18 | down |
| MELO3C007296.2 | 46 | 102 | 1.14 | 0.000747685 | up |
| MELO3C007297.2 | 1022 | 41 | -4.628 | 3.10436E-59 | down |
| MELO3C007306.2 | 223 | 105 | -1.096 | 1.27162E-07 | down |
| MELO3C007307.2 | 31 | 66 | 1.075 | 0.016012549 | up |
| MELO3C007314.2 | 577 | 106 | -2.442 | 1.95123E-54 | down |
| MELO3C007317.2 | 847 | 1965 | 1.215 | 7.40961E-08 | up |
| MELO3C007318.2 | 41 | 107 | 1.384 | 0.014823448 | up |
| MELO3C007323.2 | 2255 | 393 | -2.522 | 1.13637E-115 | down |
| MELO3C007324.2 | 25 | 4 | -2.734 | 0.001143965 | down |
| MELO3C007325.2 | 371 | 54 | -2.759 | 1.23514E-09 | down |
| MELO3C007327.2 | 330 | 136 | -1.28 | 7.47812E-12 | down |
| MELO3C007328.2 | 261 | 1 | -8.145 | 7.52796E-21 | down |
| MELO3C007340.2 | 406 | 2525 | 2.635 | 2.43734E-62 | up |
| MELO3C007341.2 | 56 | 8 | -2.699 | 9.93367E-09 | down |
| MELO3C007350.2 | 264 | 120 | -1.14 | 1.67652E-10 | down |
| MELO3C007358.2 | 169 | 34 | -2.33 | 6.72609E-14 | down |
| MELO3C007367.2 | 557 | 1382 | 1.311 | 1.37394E-08 | up |
| MELO3C007378.2 | 0 | 12 | 5.964 | 0.000107188 | up |
| MELO3C007386.2 | 89 | 26 | -1.765 | 6.88612E-05 | down |
| MELO3C007387.2 | 224 | 72 | -1.633 | 1.03376E-07 | down |
| MELO3C007391.2 | 74 | 19 | -1.925 | 1.3294E-06 | down |
| MELO3C007396.2 | 340 | 700 | 1.042 | 1.91353E-12 | up |
| MELO3C007402.2 | 2582 | 945 | -1.451 | 1.21332E-20 | down |
| MELO3C007403.2 | 19 | 0 | -6.794 | 2.64634E-06 | down |
| MELO3C007405.2 | 2559 | 1253 | -1.03 | 3.51781E-13 | down |
| MELO3C007406.2 | 457 | 925 | 1.017 | 2.71243E-19 | up |
| MELO3C007412.2 | 2118 | 4382 | 1.049 | 3.23165E-22 | up |
| MELO3C007418.2 | 1486 | 419 | -1.825 | 6.02577E-63 | down |
| MELO3C007423.2 | 18510 | 7747 | -1.257 | 3.25755E-32 | down |
| MELO3C007425.2 | 41 | 1 | -6.045 | 5.00409E-07 | down |
| MELO3C007426.2 | 2151 | 4596 | 1.095 | 4.93726E-29 | up |
| MELO3C007428.2 | 357 | 92 | -1.952 | 1.24904E-14 | down |
| MELO3C007438.2 | 6732 | 1969 | -1.773 | 4.79661E-52 | down |
| MELO3C007440.2 | 432 | 1045 | 1.274 | 4.02043E-19 | up |
| MELO3C007442.2 | 37 | 9 | -2.045 | 0.001506225 | down |
| MELO3C007443.2 | 40 | 192 | 2.249 | 6.58558E-19 | up |
| MELO3C007446.2 | 702 | 231 | -1.602 | 7.52012E-24 | down |
| MELO3C007455.2 | 13 | 114 | 3.142 | 1.35746E-12 | up |
| MELO3C007469.2 | 2900 | 1234 | -1.234 | 2.02324E-17 | down |
| MELO3C007471.2 | 1 | 11 | 4.007 | 0.012443682 | up |
| MELO3C007481.2 | 14388 | 3616 | -1.993 | 1.53379E-25 | down |
| MELO3C007482.2 | 1564 | 38 | -5.366 | 2.4501E-155 | down |
| MELO3C007483.2 | 39110 | 15135 | -1.37 | 1.41871E-72 | down |
| MELO3C007484.2 | 280 | 66 | -2.09 | 3.82724E-19 | down |
| MELO3C007486.2 | 107 | 33 | -1.722 | 1.66402E-06 | down |
| MELO3C007487.2 | 148 | 65 | -1.202 | 0.000568046 | down |
| MELO3C007491.2 | 417 | 69 | -2.596 | 8.1896E-36 | down |
| MELO3C007494.2 | 18 | 355 | 4.306 | 1.08851E-06 | up |
| MELO3C007499.2 | 1573 | 3489 | 1.15 | 6.04022E-14 | up |
| MELO3C007507.2 | 47724 | 4028 | -3.567 | 2.97352E-138 | down |
| MELO3C007509.2 | 156 | 2881 | 4.211 | 6.28709E-172 | up |
| MELO3C007512.2 | 14 | 38 | 1.445 | 0.002288695 | up |
| MELO3C007530.2 | 2 | 54 | 4.659 | 5.33192E-10 | up |
| MELO3C007531.2 | 206 | 34 | -2.584 | 5.7593E-22 | down |
| MELO3C007533.2 | 1848 | 5101 | 1.465 | 1.13042E-17 | up |
| MELO3C007535.2 | 24 | 0 | -7.122 | 2.189E-06 | down |
| MELO3C007536.2 | 7 | 31 | 2.209 | 0.006801745 | up |
| MELO3C007537.2 | 0 | 72 | 7.545 | 2.53197E-09 | up |
| MELO3C007538.2 | 0 | 12 | 5.953 | 0.000100286 | up |
| MELO3C007540.2 | 11210 | 5278 | -1.087 | 8.46933E-39 | down |
| MELO3C007545.2 | 42 | 14 | -1.556 | 0.001777496 | down |
| MELO3C007547.2 | 10 | 47 | 2.268 | 1.11368E-05 | up |
| MELO3C007549.2 | 34 | 433 | 3.659 | 3.64861E-48 | up |
| MELO3C007550.2 | 469 | 229 | -1.03 | 1.97646E-10 | down |
| MELO3C007551.2 | 4 | 0 | -4.654 | 0.023302791 | down |
| MELO3C007554.2 | 35 | 121 | 1.801 | 2.02972E-08 | up |
| MELO3C007560.2 | 2010 | 861 | -1.224 | 2.57543E-13 | down |
| MELO3C007563.2 | 395 | 182 | -1.121 | 2.64078E-09 | down |
| MELO3C007566.2 | 1 | 156 | 6.775 | 3.54557E-15 | up |
| MELO3C007574.2 | 271 | 20 | -3.754 | 1.59356E-36 | down |
| MELO3C007575.2 | 188 | 501 | 1.411 | 7.47251E-13 | up |
| MELO3C007578.2 | 15 | 1 | -3.664 | 0.000531116 | down |
| MELO3C007583.2 | 1124 | 244 | -2.197 | 3.31789E-27 | down |
| MELO3C007586.2 | 1000 | 271 | -1.881 | 7.54185E-27 | down |
| MELO3C007589.2 | 12 | 1 | -4.336 | 0.001694425 | down |
| MELO3C007592.2 | 68 | 7 | -3.287 | 2.50356E-09 | down |
| MELO3C007597.2 | 898 | 3470 | 1.951 | 2.71007E-26 | up |
| MELO3C007600.2 | 46 | 22 | -1.102 | 0.016545436 | down |
| MELO3C007601.2 | 174 | 3071 | 4.14 | 2.3087E-18 | up |
| MELO3C007602.2 | 84 | 7 | -3.645 | 1.26653E-11 | down |
| MELO3C007609.2 | 2465 | 16686 | 2.759 | 9.76445E-43 | up |
| MELO3C007613.2 | 4709 | 9487 | 1.01 | 1.39457E-37 | up |
| MELO3C007616.2 | 857 | 423 | -1.021 | 1.37707E-11 | down |
| MELO3C007617.2 | 134 | 412 | 1.623 | 2.2482E-14 | up |
| MELO3C007618.2 | 108 | 32 | -1.762 | 0.027552128 | down |
| MELO3C007627.2 | 3054 | 214 | -3.834 | 1.86485E-260 | down |
| MELO3C007634.2 | 1 | 71 | 5.622 | 9.23565E-12 | up |
| MELO3C007644.2 | 1400 | 568 | -1.3 | 3.06368E-15 | down |
| MELO3C007649.2 | 15 | 2 | -3.213 | 0.001520003 | down |
| MELO3C007652.2 | 184 | 18 | -3.284 | 6.70033E-13 | down |
| MELO3C007655.2 | 12 | 80 | 2.703 | 1.43746E-09 | up |
| MELO3C007656.2 | 10 | 27 | 1.443 | 0.007837329 | up |
| MELO3C007660.2 | 255 | 67 | -1.927 | 5.16296E-16 | down |
| MELO3C007661.2 | 870 | 2388 | 1.456 | 1.41591E-42 | up |
| MELO3C007667.2 | 1039 | 84 | -3.627 | 2.38046E-86 | down |
| MELO3C007670.2 | 2 | 38 | 4.412 | 4.77805E-07 | up |
| MELO3C007672.2 | 12 | 2 | -2.929 | 0.007210054 | down |
| MELO3C007673.2 | 818 | 4702 | 2.523 | 1.38727E-79 | up |
| MELO3C007675.2 | 257 | 107 | -1.256 | 1.75628E-05 | down |
| MELO3C007678.2 | 8 | 0 | -5.531 | 0.001161209 | down |
| MELO3C007683.2 | 796 | 1847 | 1.214 | 1.79138E-41 | up |
| MELO3C007694.2 | 144 | 1202 | 3.058 | 4.93281E-70 | up |
| MELO3C007696.2 | 3 | 23 | 2.702 | 0.000218614 | up |
| MELO3C007698.2 | 10123 | 735 | -3.785 | 1.61411E-81 | down |
| MELO3C007699.2 | 154 | 622 | 2.015 | 1.46869E-21 | up |
| MELO3C007702.2 | 140 | 3619 | 4.696 | 2.90233E-23 | up |
| MELO3C007707.2 | 17 | 119 | 2.827 | 2.95815E-13 | up |
| MELO3C007708.2 | 15 | 2 | -3.073 | 0.002128946 | down |
| MELO3C007715.2 | 1138 | 4211 | 1.888 | 1.8167E-72 | up |
| MELO3C007747.2 | 1064 | 394 | -1.435 | 2.56226E-13 | down |
| MELO3C007749.2 | 434 | 214 | -1.017 | 1.62574E-09 | down |
| MELO3C007760.2 | 22 | 136 | 2.62 | 6.03843E-12 | up |
| MELO3C007761.2 | 377 | 998 | 1.404 | 2.48485E-30 | up |
| MELO3C007762.2 | 647 | 2374 | 1.875 | 4.58664E-25 | up |
| MELO3C007767.2 | 2757 | 5544 | 1.008 | 7.65006E-07 | up |
| MELO3C007773.2 | 655 | 282 | -1.213 | 2.4068E-10 | down |
| MELO3C007781.2 | 3823 | 13976 | 1.87 | 4.4637E-58 | up |
| MELO3C007786.2 | 3035 | 1461 | -1.053 | 2.0051E-16 | down |
| MELO3C007789.2 | 308 | 815 | 1.403 | 5.89541E-06 | up |
| MELO3C007793.2 | 110 | 974 | 3.146 | 8.37189E-42 | up |
| MELO3C007794.2 | 440 | 34 | -3.713 | 5.41496E-26 | down |
| MELO3C007798.2 | 834 | 81 | -3.372 | 8.2502E-44 | down |
| MELO3C007799.2 | 5689 | 1034 | -2.461 | 1.60106E-64 | down |
| MELO3C007809.2 | 32 | 4 | -3.087 | 0.012841286 | down |
| MELO3C007820.2 | 9511 | 1205 | -2.981 | 7.1467E-53 | down |
| MELO3C007827.2 | 2 | 101 | 5.334 | 4.34666E-16 | up |
| MELO3C007837.2 | 93 | 450 | 2.273 | 1.42582E-41 | up |
| MELO3C007838.2 | 1714 | 402 | -2.091 | 2.37341E-75 | down |
| MELO3C007840.2 | 140 | 41 | -1.792 | 1.71576E-06 | down |
| MELO3C007842.2 | 343 | 933 | 1.442 | 3.1128E-26 | up |
| MELO3C007844.2 | 356 | 61 | -2.548 | 1.92197E-31 | down |
| MELO3C007846.2 | 859 | 1967 | 1.194 | 7.46684E-22 | up |
| MELO3C007849.2 | 62 | 0 | -8.515 | 3.36642E-11 | down |
| MELO3C007850.2 | 6 | 77 | 3.577 | 9.53326E-08 | up |
| MELO3C007859.2 | 667 | 1902 | 1.513 | 1.85225E-23 | up |
| MELO3C007862.2 | 69 | 235 | 1.774 | 4.27672E-10 | up |
| MELO3C007863.2 | 3 | 204 | 6.155 | 1.7936E-25 | up |
| MELO3C007871.2 | 467 | 1412 | 1.597 | 1.34011E-26 | up |
| MELO3C007872.2 | 78 | 9647 | 6.943 | 1.5853E-253 | up |
| MELO3C007874.2 | 349 | 62 | -2.492 | 1.42691E-19 | down |
| MELO3C007877.2 | 86 | 315 | 1.877 | 6.90901E-18 | up |
| MELO3C007884.2 | 2719 | 124 | -4.451 | 4.51652E-13 | down |
| MELO3C007886.2 | 8908 | 4247 | -1.069 | 1.42231E-23 | down |
| MELO3C007893.2 | 351 | 103 | -1.761 | 7.5002E-13 | down |
| MELO3C007908.2 | 8571 | 3422 | -1.325 | 4.48079E-91 | down |
| MELO3C007917.2 | 120 | 25 | -2.258 | 4.08848E-11 | down |
| MELO3C007919.2 | 195 | 566 | 1.54 | 2.80687E-19 | up |
| MELO3C007927.2 | 6 | 31 | 2.254 | 0.000190072 | up |
| MELO3C007928.2 | 165 | 736 | 2.153 | 5.16408E-14 | up |
| MELO3C007929.2 | 1 | 9 | 3.733 | 0.014008895 | up |
| MELO3C007935.2 | 168 | 77 | -1.128 | 9.89104E-07 | down |
| MELO3C007938.2 | 41 | 194 | 2.221 | 2.66836E-18 | up |
| MELO3C007949.2 | 4880 | 133 | -5.205 | 3.93564E-215 | down |
| MELO3C007956.2 | 29 | 118 | 2.04 | 3.15296E-11 | up |
| MELO3C007962.2 | 29 | 1 | -5.612 | 0.000225097 | down |
| MELO3C007967.2 | 86 | 5 | -3.979 | 1.43618E-12 | down |
| MELO3C007969.2 | 557 | 132 | -2.077 | 2.57707E-38 | down |
| MELO3C007986.2 | 964 | 422 | -1.19 | 5.3114E-25 | down |
| MELO3C007988.2 | 1873 | 926 | -1.016 | 9.42856E-21 | down |
| MELO3C007989.2 | 1347 | 2813 | 1.061 | 1.34355E-23 | up |
| MELO3C007990.2 | 62 | 311 | 2.326 | 7.43835E-17 | up |
| MELO3C007995.2 | 198 | 51 | -1.955 | 5.04694E-11 | down |
| MELO3C008001.2 | 664 | 286 | -1.21 | 6.94617E-21 | down |
| MELO3C008005.2 | 314 | 1409 | 2.165 | 5.86811E-96 | up |
| MELO3C008006.2 | 70 | 181 | 1.369 | 8.54672E-10 | up |
| MELO3C008008.2 | 1824 | 393 | -2.214 | 1.19081E-26 | down |
| MELO3C008015.2 | 355 | 757 | 1.094 | 5.43487E-20 | up |
| MELO3C008026.2 | 102 | 13 | -2.96 | 4.78341E-15 | down |
| MELO3C008028.2 | 65 | 311 | 2.257 | 4.83211E-13 | up |
| MELO3C008029.2 | 0 | 7 | 5.114 | 0.004650385 | up |
| MELO3C008033.2 | 387 | 815 | 1.073 | 3.75033E-06 | up |
| MELO3C008039.2 | 145 | 29 | -2.349 | 6.77128E-10 | down |
| MELO3C008040.2 | 172 | 421 | 1.291 | 3.38738E-17 | up |
| MELO3C008049.2 | 339 | 122 | -1.484 | 5.80534E-13 | down |
| MELO3C008050.2 | 3 | 33 | 3.183 | 3.13031E-06 | up |
| MELO3C008059.2 | 1089 | 472 | -1.205 | 1.62048E-24 | down |
| MELO3C008060.2 | 12520 | 335 | -5.222 | 1.32509E-156 | down |
| MELO3C008063.2 | 4473 | 1137 | -1.976 | 9.12968E-67 | down |
| MELO3C008064.2 | 4449 | 2225 | -1 | 9.14525E-15 | down |
| MELO3C008077.2 | 27 | 172 | 2.679 | 5.53546E-14 | up |
| MELO3C008080.2 | 646 | 317 | -1.027 | 1.99559E-10 | down |
| MELO3C008084.2 | 7 | 0 | -5.399 | 0.003374669 | down |
| MELO3C008091.2 | 128 | 12 | -3.427 | 3.43649E-18 | down |
| MELO3C008097.2 | 0 | 440 | 11.121 | 1.19613E-20 | up |
| MELO3C008100.2 | 480 | 28 | -4.117 | 1.50217E-41 | down |
| MELO3C008101.2 | 58 | 173 | 1.588 | 1.28306E-05 | up |
| MELO3C008114.2 | 4062 | 637 | -2.672 | 5.82449E-89 | down |
| MELO3C008115.2 | 1031 | 507 | -1.024 | 1.08461E-18 | down |
| MELO3C008116.2 | 386 | 172 | -1.165 | 9.93033E-08 | down |
| MELO3C008124.2 | 48 | 109 | 1.185 | 0.000206728 | up |
| MELO3C008129.2 | 593 | 257 | -1.207 | 6.01686E-20 | down |
| MELO3C008134.2 | 454 | 1545 | 1.768 | 4.08627E-50 | up |
| MELO3C008140.2 | 544 | 177 | -1.623 | 5.16575E-16 | down |
| MELO3C008143.2 | 36 | 7 | -2.341 | 0.000386504 | down |
| MELO3C008145.2 | 64 | 134 | 1.055 | 1.38936E-05 | up |
| MELO3C008149.2 | 456 | 40 | -3.525 | 9.56355E-48 | down |
| MELO3C008152.2 | 321 | 34 | -3.25 | 1.0144E-46 | down |
| MELO3C008158.2 | 1359 | 585 | -1.215 | 7.08402E-32 | down |
| MELO3C008160.2 | 209 | 90 | -1.214 | 1.90258E-08 | down |
| MELO3C008161.2 | 16 | 125 | 2.927 | 1.39393E-17 | up |
| MELO3C008163.2 | 2816 | 8197 | 1.541 | 5.47439E-74 | up |
| MELO3C008164.2 | 252 | 16 | -4.002 | 2.00882E-37 | down |
| MELO3C008171.2 | 784 | 1838 | 1.229 | 3.34404E-38 | up |
| MELO3C008175.2 | 776 | 167 | -2.219 | 9.03615E-29 | down |
| MELO3C008176.2 | 115 | 53 | -1.114 | 0.000732024 | down |
| MELO3C008195.2 | 82 | 413 | 2.333 | 5.32604E-25 | up |
| MELO3C008196.2 | 0 | 9 | 5.601 | 0.000783965 | up |
| MELO3C008201.2 | 768 | 218 | -1.816 | 1.10196E-31 | down |
| MELO3C008203.2 | 126 | 1231 | 3.283 | 3.5881E-42 | up |
| MELO3C008205.2 | 11 | 0 | -5.976 | 0.000154606 | down |
| MELO3C008209.2 | 112 | 38 | -1.543 | 1.94768E-05 | down |
| MELO3C008211.2 | 187 | 463 | 1.31 | 2.21346E-11 | up |
| MELO3C008224.2 | 1001 | 408 | -1.294 | 2.00074E-23 | down |
| MELO3C008231.2 | 17893 | 6995 | -1.355 | 5.20256E-34 | down |
| MELO3C008233.2 | 1187 | 215 | -2.462 | 3.55532E-56 | down |
| MELO3C008236.2 | 239 | 735 | 1.621 | 4.49509E-22 | up |
| MELO3C008241.2 | 4220 | 9796 | 1.215 | 1.25748E-37 | up |
| MELO3C008244.2 | 1824 | 4811 | 1.399 | 7.60347E-68 | up |
| MELO3C008250.2 | 0 | 78 | 8.618 | 4.81698E-12 | up |
| MELO3C008252.2 | 0 | 14 | 6.162 | 0.000276141 | up |
| MELO3C008255.2 | 709 | 216 | -1.717 | 1.00441E-33 | down |
| MELO3C008261.2 | 15 | 83 | 2.472 | 2.86461E-08 | up |
| MELO3C008262.2 | 228 | 28 | -3.011 | 1.51988E-19 | down |
| MELO3C008263.2 | 52 | 0 | -8.256 | 2.31625E-10 | down |
| MELO3C008269.2 | 34 | 223 | 2.719 | 6.08524E-19 | up |
| MELO3C008272.2 | 5 | 34 | 2.738 | 5.05528E-05 | up |
| MELO3C008281.2 | 50 | 1 | -5.367 | 8.09128E-09 | down |
| MELO3C008286.2 | 3049 | 574 | -2.409 | 1.44567E-56 | down |
| MELO3C008287.2 | 10 | 240 | 4.588 | 5.59112E-26 | up |
| MELO3C008288.2 | 2896 | 929 | -1.641 | 1.95699E-31 | down |
| MELO3C008313.2 | 1995 | 872 | -1.195 | 3.01924E-39 | down |
| MELO3C008314.2 | 845 | 125 | -2.75 | 1.70364E-32 | down |
| MELO3C008318.2 | 1766 | 599 | -1.56 | 4.06843E-60 | down |
| MELO3C008322.2 | 358 | 86 | -2.05 | 5.2313E-12 | down |
| MELO3C008332.2 | 508 | 127 | -1.999 | 1.50699E-25 | down |
| MELO3C008337.2 | 0 | 84 | 8.732 | 1.83128E-12 | up |
| MELO3C008342.2 | 29 | 1 | -4.534 | 1.13051E-06 | down |
| MELO3C008350.2 | 216 | 23 | -3.214 | 2.32942E-13 | down |
| MELO3C008357.2 | 992 | 2260 | 1.189 | 3.88681E-14 | up |
| MELO3C008366.2 | 1169 | 16639 | 3.831 | 0.000408299 | up |
| MELO3C008375.2 | 9 | 343 | 5.309 | 0.000273227 | up |
| MELO3C008381.2 | 0 | 143 | 9.499 | 3.36308E-14 | up |
| MELO3C008382.2 | 5 | 0 | -5.065 | 0.007156074 | down |
| MELO3C008394.2 | 0 | 13 | 6.098 | 8.04139E-05 | up |
| MELO3C008410.2 | 42 | 6 | -2.883 | 3.69861E-06 | down |
| MELO3C008415.2 | 7755 | 2381 | -1.704 | 1.89647E-59 | down |
| MELO3C008417.2 | 25805 | 7774 | -1.731 | 1.851E-20 | down |
| MELO3C008424.2 | 61 | 1141 | 4.233 | 1.09641E-42 | up |
| MELO3C008429.2 | 8396 | 3531 | -1.249 | 9.8098E-52 | down |
| MELO3C008431.2 | 6 | 0 | -5.074 | 0.009468566 | down |
| MELO3C008433.2 | 3512 | 877 | -2.003 | 1.06805E-41 | down |
| MELO3C008435.2 | 1093 | 270 | -2.02 | 1.17577E-29 | down |
| MELO3C008436.2 | 290 | 0 | -10.73 | 2.81864E-19 | down |
| MELO3C008440.2 | 1 | 40 | 5.825 | 1.41278E-06 | up |
| MELO3C008456.2 | 218 | 28 | -2.96 | 2.86057E-29 | down |
| MELO3C008461.2 | 817 | 359 | -1.19 | 2.69825E-26 | down |
| MELO3C008466.2 | 31 | 4 | -3.05 | 3.51401E-06 | down |
| MELO3C008469.2 | 2034 | 14291 | 2.813 | 2.369E-83 | up |
| MELO3C008491.2 | 123 | 56 | -1.117 | 0.000335689 | down |
| MELO3C008492.2 | 238 | 1480 | 2.635 | 2.93403E-133 | up |
| MELO3C008498.2 | 14 | 107 | 2.903 | 5.6534E-15 | up |
| MELO3C008499.2 | 743 | 206 | -1.853 | 2.50704E-20 | down |
| MELO3C008524.2 | 1543 | 380 | -2.019 | 4.24319E-48 | down |
| MELO3C008526.2 | 13 | 1 | -4.461 | 0.002348752 | down |
| MELO3C008529.2 | 56 | 338 | 2.58 | 2.92507E-29 | up |
| MELO3C008530.2 | 3 | 61 | 4.095 | 1.03875E-10 | up |
| MELO3C008553.2 | 190 | 465 | 1.291 | 1.82285E-19 | up |
| MELO3C008557.2 | 246 | 564 | 1.201 | 4.35431E-21 | up |
| MELO3C008570.2 | 211 | 6 | -5.245 | 1.0133E-35 | down |
| MELO3C008578.2 | 211 | 1213 | 2.523 | 6.0663E-67 | up |
| MELO3C008596.2 | 725 | 1687 | 1.219 | 1.00127E-22 | up |
| MELO3C008598.2 | 3167 | 1308 | -1.277 | 8.47224E-36 | down |
| MELO3C008647.2 | 1 | 122 | 7.418 | 5.30087E-12 | up |
| MELO3C008648.2 | 8 | 103 | 3.58 | 3.36542E-11 | up |
| MELO3C008657.2 | 181 | 4 | -5.473 | 8.31168E-19 | down |
| MELO3C008659.2 | 616 | 2 | -8.382 | 5.52148E-43 | down |
| MELO3C008663.2 | 13917 | 103 | -7.081 | 1.73785E-235 | down |
| MELO3C008669.2 | 43 | 165 | 1.967 | 3.12462E-12 | up |
| MELO3C008672.2 | 745 | 178 | -2.064 | 1.15376E-23 | down |
| MELO3C008677.2 | 1585 | 445 | -1.836 | 5.82439E-34 | down |
| MELO3C008679.2 | 82 | 5 | -3.969 | 7.68312E-16 | down |
| MELO3C008688.2 | 0 | 33 | 7.366 | 4.58409E-08 | up |
| MELO3C008693.2 | 1 | 50 | 6.11 | 8.19577E-08 | up |
| MELO3C008696.2 | 28 | 0 | -7.353 | 1.88095E-07 | down |
| MELO3C008699.2 | 2613 | 264 | -3.308 | 1.39646E-207 | down |
| MELO3C008731.2 | 29786 | 252 | -6.885 | 6.97512E-268 | down |
| MELO3C008738.2 | 0 | 10 | 5.68 | 0.000352586 | up |
| MELO3C008739.2 | 240 | 540 | 1.173 | 2.79925E-08 | up |
| MELO3C008742.2 | 2 | 69 | 5.262 | 3.52069E-11 | up |
| MELO3C008751.2 | 17 | 104 | 2.575 | 1.39846E-12 | up |
| MELO3C008767.2 | 218 | 522 | 1.258 | 3.92691E-09 | up |
| MELO3C008773.2 | 4665 | 1554 | -1.586 | 2.45777E-38 | down |
| MELO3C008798.2 | 157 | 7 | -4.482 | 2.16996E-19 | down |
| MELO3C008799.2 | 259 | 100 | -1.374 | 5.44109E-06 | down |
| MELO3C008801.2 | 1180 | 2716 | 1.203 | 4.30807E-16 | up |
| MELO3C008802.2 | 688 | 222 | -1.632 | 0.002652849 | down |
| MELO3C008835.2 | 602 | 282 | -1.096 | 5.2861E-19 | down |
| MELO3C008846.2 | 374 | 166 | -1.167 | 4.84419E-12 | down |
| MELO3C008855.2 | 11 | 38 | 1.781 | 0.001849627 | up |
| MELO3C008856.2 | 649 | 261 | -1.314 | 2.65165E-23 | down |
| MELO3C008866.2 | 79 | 22 | -1.843 | 1.44555E-06 | down |
| MELO3C008885.2 | 67 | 618 | 3.2 | 7.23175E-68 | up |
| MELO3C008895.2 | 356 | 28 | -3.692 | 9.72814E-49 | down |
| MELO3C008898.2 | 992 | 404 | -1.295 | 7.30042E-14 | down |
| MELO3C008899.2 | 633 | 2017 | 1.672 | 1.6973E-25 | up |
| MELO3C008910.2 | 196 | 612 | 1.642 | 2.1396E-30 | up |
| MELO3C008920.2 | 4155 | 949 | -2.132 | 2.38344E-34 | down |
| MELO3C008923.2 | 85 | 700 | 3.037 | 1.59353E-70 | up |
| MELO3C008948.2 | 0 | 9 | 5.455 | 0.00141214 | up |
| MELO3C008949.2 | 793 | 363 | -1.129 | 5.97064E-23 | down |
| MELO3C008963.2 | 125 | 14 | -3.175 | 7.03612E-13 | down |
| MELO3C008996.2 | 6045 | 2710 | -1.158 | 2.21143E-62 | down |
| MELO3C009007.2 | 165 | 526 | 1.675 | 5.19538E-11 | up |
| MELO3C009016.2 | 52 | 226 | 2.121 | 1.07944E-14 | up |
| MELO3C009019.2 | 1564 | 496 | -1.657 | 5.955E-27 | down |
| MELO3C009030.2 | 43 | 0 | -7.976 | 7.81033E-10 | down |
| MELO3C009053.2 | 521 | 1600 | 1.621 | 4.45612E-35 | up |
| MELO3C009076.2 | 497 | 5496 | 3.466 | 1.47493E-152 | up |
| MELO3C009078.2 | 113 | 38 | -1.582 | 2.87484E-06 | down |
| MELO3C009089.2 | 2796 | 6679 | 1.256 | 1.0087E-30 | up |
| MELO3C009092.2 | 33 | 6 | -2.434 | 0.000167393 | down |
| MELO3C009097.2 | 1434 | 484 | -1.565 | 0.00925379 | down |
| MELO3C009104.2 | 1626 | 692 | -1.233 | 4.43413E-18 | down |
| MELO3C009106.2 | 8 | 30 | 1.935 | 0.001221363 | up |
| MELO3C009107.2 | 14 | 110 | 3.014 | 1.16001E-15 | up |
| MELO3C009116.2 | 35 | 71 | 1.007 | 0.004965115 | up |
| MELO3C009117.2 | 0 | 6 | 3.872 | 0.026261127 | up |
| MELO3C009121.2 | 10 | 62 | 2.689 | 0.00058461 | up |
| MELO3C009122.2 | 7 | 398 | 5.87 | 3.12647E-44 | up |
| MELO3C009133.2 | 124 | 800 | 2.688 | 3.96262E-27 | up |
| MELO3C009135.2 | 133 | 329 | 1.31 | 3.85674E-11 | up |
| MELO3C009139.2 | 1509 | 738 | -1.032 | 4.27379E-23 | down |
| MELO3C009143.2 | 107 | 49 | -1.107 | 0.000106815 | down |
| MELO3C009146.2 | 1145 | 504 | -1.184 | 1.54583E-28 | down |
| MELO3C009147.2 | 964 | 303 | -1.668 | 3.27787E-37 | down |
| MELO3C009166.2 | 397 | 1172 | 1.561 | 8.72171E-29 | up |
| MELO3C009185.2 | 87 | 261 | 1.588 | 3.29645E-07 | up |
| MELO3C009187.2 | 6231 | 1457 | -2.096 | 1.39768E-52 | down |
| MELO3C009190.2 | 593 | 88 | -2.77 | 6.50605E-26 | down |
| MELO3C009192.2 | 61 | 2058 | 5.088 | 2.04565E-200 | up |
| MELO3C009197.2 | 673 | 1394 | 1.052 | 7.75923E-26 | up |
| MELO3C009203.2 | 747 | 13176 | 4.14 | 1.3353E-130 | up |
| MELO3C009204.2 | 1 | 16 | 3.543 | 0.000861743 | up |
| MELO3C009205.2 | 16 | 2 | -3.092 | 0.013636594 | down |
| MELO3C009206.2 | 8 | 24 | 1.532 | 0.016198847 | up |
| MELO3C009210.2 | 1947 | 471 | -2.047 | 7.7915E-17 | down |
| MELO3C009212.2 | 75 | 19 | -1.964 | 1.16063E-07 | down |
| MELO3C009213.2 | 476 | 1318 | 1.47 | 2.05244E-21 | up |
| MELO3C009218.2 | 89 | 44 | -1.035 | 0.001131891 | down |
| MELO3C009219.2 | 7 | 197 | 4.709 | 1.48257E-17 | up |
| MELO3C009221.2 | 299 | 102 | -1.555 | 1.38144E-09 | down |
| MELO3C009222.2 | 3435 | 1656 | -1.052 | 1.37772E-23 | down |
| MELO3C009223.2 | 410 | 178 | -1.206 | 1.35947E-09 | down |
| MELO3C009226.2 | 2822 | 1398 | -1.014 | 1.46875E-31 | down |
| MELO3C009229.2 | 29 | 7 | -2.016 | 0.01794436 | down |
| MELO3C009230.2 | 216 | 769 | 1.833 | 1.64095E-32 | up |
| MELO3C009232.2 | 16 | 3 | -2.371 | 0.003631277 | down |
| MELO3C009233.2 | 367 | 79 | -2.215 | 1.76053E-25 | down |
| MELO3C009236.2 | 4 | 0 | -4.651 | 0.023899341 | down |
| MELO3C009238.2 | 28 | 7 | -2.019 | 0.001276012 | down |
| MELO3C009241.2 | 765 | 308 | -1.316 | 8.71148E-10 | down |
| MELO3C009245.2 | 88 | 0 | -8.054 | 1.75706E-10 | down |
| MELO3C009246.2 | 2 | 385 | 7.264 | 8.4337E-35 | up |
| MELO3C009252.2 | 3174 | 398 | -2.995 | 3.04068E-43 | down |
| MELO3C009262.2 | 30 | 114 | 1.944 | 4.67416E-08 | up |
| MELO3C009263.2 | 3 | 259 | 6.326 | 7.2108E-31 | up |
| MELO3C009266.2 | 7 | 75 | 3.402 | 3.13892E-09 | up |
| MELO3C009267.2 | 33 | 9 | -1.923 | 0.000658148 | down |
| MELO3C009282.2 | 2206 | 74 | -4.91 | 7.05547E-245 | down |
| MELO3C009286.2 | 360 | 155 | -1.216 | 4.85226E-07 | down |
| MELO3C009288.2 | 1157 | 2489 | 1.105 | 7.66065E-08 | up |
| MELO3C009291.2 | 37 | 92 | 1.319 | 0.000406778 | up |
| MELO3C009292.2 | 42 | 148 | 1.82 | 3.6444E-05 | up |
| MELO3C009294.2 | 47 | 9 | -2.318 | 0.000200122 | down |
| MELO3C009307.2 | 17 | 4 | -2.178 | 0.006288521 | down |
| MELO3C009308.2 | 137 | 426 | 1.638 | 3.32246E-16 | up |
| MELO3C009313.2 | 25 | 197 | 3.017 | 4.20111E-25 | up |
| MELO3C009321.2 | 12 | 43 | 1.819 | 0.002077071 | up |
| MELO3C009322.2 | 11 | 37 | 1.782 | 0.00138926 | up |
| MELO3C009325.2 | 63 | 129 | 1.031 | 0.004504777 | up |
| MELO3C009328.2 | 131 | 446 | 1.766 | 2.23558E-16 | up |
| MELO3C009330.2 | 1244 | 143 | -3.117 | 4.40676E-47 | down |
| MELO3C009333.2 | 147 | 1 | -7.892 | 8.41174E-14 | down |
| MELO3C009334.2 | 8033 | 25576 | 1.671 | 1.00476E-24 | up |
| MELO3C009337.2 | 53 | 113 | 1.101 | 0.000300521 | up |
| MELO3C009339.2 | 2448 | 873 | -1.488 | 3.75377E-48 | down |
| MELO3C009342.2 | 44 | 172 | 1.962 | 7.99167E-12 | up |
| MELO3C009355.2 | 46386 | 13660 | -1.764 | 2.57196E-36 | down |
| MELO3C009361.2 | 846 | 382 | -1.148 | 8.88212E-19 | down |
| MELO3C009362.2 | 1880 | 813 | -1.21 | 5.50769E-14 | down |
| MELO3C009364.2 | 266 | 42 | -2.652 | 9.28362E-19 | down |
| MELO3C009368.2 | 178 | 87 | -1.04 | 0.001089712 | down |
| MELO3C009369.2 | 4 | 23 | 2.411 | 0.004275388 | up |
| MELO3C009372.2 | 3292 | 206 | -3.999 | 6.54597E-187 | down |
| MELO3C009386.2 | 363 | 111 | -1.71 | 5.27975E-17 | down |
| MELO3C009387.2 | 855 | 37 | -4.548 | 4.49158E-44 | down |
| MELO3C009390.2 | 322 | 821 | 1.352 | 3.08363E-19 | up |
| MELO3C009391.2 | 3007 | 415 | -2.856 | 5.10038E-63 | down |
| MELO3C009403.2 | 1095 | 2326 | 1.086 | 4.77301E-14 | up |
| MELO3C009406.2 | 1 | 13 | 4.206 | 0.001830026 | up |
| MELO3C009412.2 | 0 | 22 | 5.819 | 1.92619E-05 | up |
| MELO3C009415.2 | 813 | 133 | -2.615 | 1.28167E-33 | down |
| MELO3C009435.2 | 3 | 20 | 2.807 | 0.002924493 | up |
| MELO3C009438.2 | 8772 | 21722 | 1.308 | 5.62564E-42 | up |
| MELO3C009440.2 | 1529 | 4273 | 1.482 | 3.93005E-26 | up |
| MELO3C009452.2 | 1084 | 3530 | 1.703 | 3.28728E-18 | up |
| MELO3C009454.2 | 1201 | 584 | -1.039 | 2.18903E-10 | down |
| MELO3C009459.2 | 745 | 263 | -1.499 | 4.18687E-25 | down |
| MELO3C009461.2 | 14 | 1 | -3.208 | 0.008879061 | down |
| MELO3C009470.2 | 30 | 4 | -3.025 | 1.81387E-05 | down |
| MELO3C009475.2 | 1347 | 500 | -1.43 | 2.81557E-24 | down |
| MELO3C009477.2 | 186 | 1073 | 2.528 | 5.11632E-39 | up |
| MELO3C009486.2 | 7 | 149 | 4.379 | 1.08876E-16 | up |
| MELO3C009495.2 | 26 | 0 | -7.256 | 5.89389E-07 | down |
| MELO3C009497.2 | 800 | 3394 | 2.086 | 5.99848E-52 | up |
| MELO3C009501.2 | 195 | 68 | -1.503 | 7.40775E-07 | down |
| MELO3C009503.2 | 391 | 146 | -1.426 | 1.39545E-11 | down |
| MELO3C009504.2 | 0 | 214 | 10.079 | 5.37732E-17 | up |
| MELO3C009505.2 | 0 | 140 | 9.47 | 1.14644E-14 | up |
| MELO3C009506.2 | 626 | 5 | -6.91 | 3.06669E-57 | down |
| MELO3C009507.2 | 102 | 4 | -4.667 | 1.63008E-17 | down |
| MELO3C009512.2 | 1528 | 215 | -2.828 | 3.0685E-44 | down |
| MELO3C009524.2 | 831 | 95 | -3.118 | 5.45205E-41 | down |
| MELO3C009525.2 | 13 | 2 | -2.656 | 0.008318095 | down |
| MELO3C009530.2 | 124 | 3 | -5.527 | 0.000133935 | down |
| MELO3C009531.2 | 107 | 2 | -5.855 | 6.17058E-05 | down |
| MELO3C009532.2 | 4022 | 9307 | 1.211 | 2.71389E-36 | up |
| MELO3C009536.2 | 1 | 13 | 4.213 | 0.002455293 | up |
| MELO3C009541.2 | 14 | 0 | -6.347 | 0.000146813 | down |
| MELO3C009543.2 | 33 | 3 | -3.561 | 0.000356446 | down |
| MELO3C009550.2 | 1219 | 188 | -2.697 | 8.37759E-64 | down |
| MELO3C009554.2 | 44 | 6 | -2.952 | 3.78189E-06 | down |
| MELO3C009555.2 | 130 | 35 | -1.875 | 2.55462E-07 | down |
| MELO3C009564.2 | 86 | 449 | 2.391 | 9.30786E-29 | up |
| MELO3C009567.2 | 1153 | 7913 | 2.779 | 5.68659E-79 | up |
| MELO3C009569.2 | 670 | 75 | -3.146 | 2.31516E-39 | down |
| MELO3C009572.2 | 16368 | 7779 | -1.073 | 0.00068524 | down |
| MELO3C009574.2 | 416 | 905 | 1.122 | 3.84849E-19 | up |
| MELO3C009581.2 | 2325 | 825 | -1.495 | 5.4555E-57 | down |
| MELO3C009585.2 | 3 | 33 | 3.193 | 4.94312E-05 | up |
| MELO3C009587.2 | 609 | 1320 | 1.116 | 2.18394E-16 | up |
| MELO3C009595.2 | 4687 | 1881 | -1.317 | 1.06329E-72 | down |
| MELO3C009596.2 | 140 | 19 | -2.869 | 5.93009E-11 | down |
| MELO3C009597.2 | 826 | 2312 | 1.486 | 6.26127E-51 | up |
| MELO3C009598.2 | 85 | 179 | 1.074 | 1.27164E-05 | up |
| MELO3C009602.2 | 163 | 475 | 1.545 | 8.27009E-20 | up |
| MELO3C009618.2 | 0 | 49 | 7.963 | 4.93827E-10 | up |
| MELO3C009628.2 | 1445 | 696 | -1.054 | 2.71791E-17 | down |
| MELO3C009630.2 | 9817 | 2332 | -2.073 | 1.04325E-30 | down |
| MELO3C009631.2 | 10 | 1 | -3.401 | 0.007798029 | down |
| MELO3C009637.2 | 37 | 1 | -4.905 | 2.20725E-06 | down |
| MELO3C009649.2 | 1 | 47 | 5.049 | 3.98802E-09 | up |
| MELO3C009665.2 | 69 | 596 | 3.115 | 4.47591E-22 | up |
| MELO3C009674.2 | 4196 | 1007 | -2.06 | 1.4876E-37 | down |
| MELO3C009678.2 | 932 | 3201 | 1.78 | 8.9741E-19 | up |
| MELO3C009681.2 | 87 | 38 | -1.183 | 0.018426725 | down |
| MELO3C009682.2 | 400 | 870 | 1.122 | 8.44025E-19 | up |
| MELO3C009683.2 | 4267 | 10463 | 1.294 | 0.002137027 | up |
| MELO3C009706.2 | 617 | 73 | -3.077 | 4.7263E-49 | down |
| MELO3C009710.2 | 1573 | 775 | -1.019 | 2.1491E-13 | down |
| MELO3C009719.2 | 470 | 85 | -2.471 | 1.31039E-40 | down |
| MELO3C009728.2 | 613 | 98 | -2.653 | 3.50397E-30 | down |
| MELO3C009733.2 | 161 | 15 | -3.37 | 1.27541E-20 | down |
| MELO3C009739.2 | 138 | 1 | -6.803 | 1.82082E-18 | down |
| MELO3C009740.2 | 27 | 1 | -5.424 | 8.3718E-06 | down |
| MELO3C009741.2 | 3074 | 418 | -2.88 | 1.90071E-63 | down |
| MELO3C009755.2 | 622 | 241 | -1.367 | 2.08414E-05 | down |
| MELO3C009757.2 | 24 | 63 | 1.412 | 0.005834205 | up |
| MELO3C009759.2 | 13919 | 6314 | -1.14 | 2.21256E-88 | down |
| MELO3C009768.2 | 274 | 61 | -2.163 | 6.0834E-15 | down |
| MELO3C009773.2 | 14 | 41 | 1.534 | 0.005262683 | up |
| MELO3C009782.2 | 4436 | 28985 | 2.708 | 4.20049E-160 | up |
| MELO3C009784.2 | 73 | 150 | 1.039 | 0.000163909 | up |
| MELO3C009789.2 | 814 | 104 | -2.969 | 3.69959E-83 | down |
| MELO3C009790.2 | 1605 | 53 | -4.922 | 6.85973E-225 | down |
| MELO3C009798.2 | 204 | 28 | -2.864 | 6.24994E-26 | down |
| MELO3C009806.2 | 84 | 36 | -1.223 | 7.3091E-05 | down |
| MELO3C009810.2 | 192 | 648 | 1.753 | 8.39083E-21 | up |
| MELO3C009818.2 | 3486 | 1302 | -1.421 | 2.00585E-17 | down |
| MELO3C009826.2 | 2285 | 4584 | 1.005 | 5.7757E-13 | up |
| MELO3C009833.2 | 12 | 124 | 3.351 | 1.22814E-18 | up |
| MELO3C009846.2 | 3335 | 1210 | -1.463 | 1.14653E-27 | down |
| MELO3C009855.2 | 100 | 25 | -1.966 | 2.72392E-08 | down |
| MELO3C009856.2 | 63 | 0 | -8.532 | 1.31991E-10 | down |
| MELO3C009858.2 | 0 | 5 | 4.705 | 0.014110069 | up |
| MELO3C009863.2 | 517 | 246 | -1.074 | 4.44984E-08 | down |
| MELO3C009864.2 | 8078 | 154 | -5.707 | 0 | down |
| MELO3C009869.2 | 85 | 420 | 2.309 | 3.48881E-14 | up |
| MELO3C009870.2 | 70 | 24 | -1.494 | 0.012328205 | down |
| MELO3C009872.2 | 31 | 606 | 4.281 | 4.19649E-52 | up |
| MELO3C009873.2 | 728 | 70 | -3.393 | 1.06423E-58 | down |
| MELO3C009874.2 | 13497 | 4305 | -1.649 | 6.23602E-23 | down |
| MELO3C009877.2 | 4419 | 668 | -2.727 | 1.32742E-118 | down |
| MELO3C009881.2 | 385 | 1 | -9.285 | 3.46698E-19 | down |
| MELO3C009882.2 | 538 | 203 | -1.41 | 5.45568E-24 | down |
| MELO3C009885.2 | 5 | 0 | -4.977 | 0.011730902 | down |
| MELO3C009900.2 | 90 | 7 | -3.55 | 3.26159E-08 | down |
| MELO3C009910.2 | 92 | 224 | 1.281 | 6.07148E-10 | up |
| MELO3C009911.2 | 1727 | 710 | -1.282 | 7.5368E-36 | down |
| MELO3C009918.2 | 82 | 165 | 1.013 | 0.001632851 | up |
| MELO3C009927.2 | 2720 | 761 | -1.838 | 4.52823E-30 | down |
| MELO3C009934.2 | 623 | 1347 | 1.113 | 3.65744E-10 | up |
| MELO3C009937.2 | 312 | 93 | -1.751 | 5.23679E-19 | down |
| MELO3C009940.2 | 334 | 97 | -1.789 | 6.3229E-13 | down |
| MELO3C009943.2 | 256 | 871 | 1.767 | 1.68029E-27 | up |
| MELO3C009944.2 | 684 | 1376 | 1.007 | 4.66189E-14 | up |
| MELO3C009952.2 | 1319 | 351 | -1.909 | 2.49505E-46 | down |
| MELO3C009961.2 | 10837 | 3830 | -1.501 | 1.68371E-51 | down |
| MELO3C009963.2 | 1161 | 2409 | 1.053 | 8.98031E-10 | up |
| MELO3C009965.2 | 4609 | 1256 | -1.877 | 3.35874E-23 | down |
| MELO3C009966.2 | 695 | 1617 | 1.218 | 4.03781E-25 | up |
| MELO3C009968.2 | 1301 | 535 | -1.282 | 4.497E-17 | down |
| MELO3C009970.2 | 14 | 59 | 2.115 | 5.13705E-06 | up |
| MELO3C009990.2 | 29 | 1 | -4.54 | 5.8974E-06 | down |
| MELO3C009996.2 | 9816 | 3629 | -1.436 | 2.25046E-70 | down |
| MELO3C010002.2 | 38 | 171 | 2.161 | 3.53834E-11 | up |
| MELO3C010008.2 | 3076 | 1056 | -1.543 | 1.09094E-25 | down |
| MELO3C010015.2 | 122 | 418 | 1.775 | 5.53478E-09 | up |
| MELO3C010033.2 | 967 | 2007 | 1.054 | 1.15299E-05 | up |
| MELO3C010045.2 | 1764 | 456 | -1.953 | 9.08414E-32 | down |
| MELO3C010056.2 | 15 | 40 | 1.441 | 0.016908373 | up |
| MELO3C010061.2 | 165 | 82 | -1.007 | 2.10529E-06 | down |
| MELO3C010125.2 | 65 | 874 | 3.759 | 1.82519E-60 | up |
| MELO3C010132.2 | 1 | 134 | 7.548 | 5.30225E-12 | up |
| MELO3C010152.2 | 309 | 128 | -1.267 | 6.67408E-10 | down |
| MELO3C010155.2 | 0 | 121 | 9.255 | 2.80489E-14 | up |
| MELO3C010162.2 | 1318 | 45 | -4.871 | 2.48399E-110 | down |
| MELO3C010164.2 | 4820 | 1543 | -1.644 | 1.00911E-53 | down |
| MELO3C010172.2 | 3838 | 68 | -5.829 | 8.72573E-261 | down |
| MELO3C010177.2 | 45 | 0 | -8.059 | 2.71628E-10 | down |
| MELO3C010181.2 | 689 | 228 | -1.599 | 1.2454E-19 | down |
| MELO3C010182.2 | 8 | 1 | -3.679 | 0.021064405 | down |
| MELO3C010183.2 | 7622 | 663 | -3.522 | 1.94405E-11 | down |
| MELO3C010184.2 | 1752 | 26 | -6.052 | 1.78413E-13 | down |
| MELO3C010188.2 | 10357 | 3439 | -1.591 | 6.60342E-61 | down |
| MELO3C010190.2 | 632 | 38 | -4.037 | 1.52966E-36 | down |
| MELO3C010193.2 | 16 | 66 | 2.084 | 1.51718E-05 | up |
| MELO3C010196.2 | 887 | 251 | -1.821 | 1.05224E-11 | down |
| MELO3C010210.2 | 1365 | 127 | -3.426 | 8.91326E-55 | down |
| MELO3C010212.2 | 7 | 0 | -5.246 | 0.005141826 | down |
| MELO3C010215.2 | 1442 | 699 | -1.047 | 4.67312E-12 | down |
| MELO3C010222.2 | 4336 | 2146 | -1.014 | 5.88898E-19 | down |
| MELO3C010233.2 | 21 | 885 | 5.365 | 5.93673E-76 | up |
| MELO3C010234.2 | 604 | 2206 | 1.87 | 2.97368E-30 | up |
| MELO3C010236.2 | 44 | 101 | 1.193 | 1.54571E-05 | up |
| MELO3C010244.2 | 4372 | 73 | -5.894 | 2.06796E-146 | down |
| MELO3C010256.2 | 95 | 47 | -1.013 | 0.003496112 | down |
| MELO3C010259.2 | 0 | 5 | 4.696 | 0.016979065 | up |
| MELO3C010261.2 | 668 | 2313 | 1.793 | 7.01927E-49 | up |
| MELO3C010263.2 | 285 | 590 | 1.046 | 3.59797E-08 | up |
| MELO3C010270.2 | 291 | 60 | -2.296 | 7.29184E-16 | down |
| MELO3C010272.2 | 335 | 15 | -4.467 | 1.58015E-39 | down |
| MELO3C010275.2 | 21799 | 3213 | -2.762 | 2.89537E-252 | down |
| MELO3C010276.2 | 235 | 3 | -6.262 | 2.66334E-33 | down |
| MELO3C010277.2 | 2383 | 913 | -1.384 | 2.19712E-62 | down |
| MELO3C010280.2 | 10 | 39 | 1.888 | 0.000650624 | up |
| MELO3C010293.2 | 55 | 117 | 1.073 | 0.000270499 | up |
| MELO3C010297.2 | 865 | 2069 | 1.259 | 2.24268E-33 | up |
| MELO3C010305.2 | 1781 | 3565 | 1.001 | 9.61573E-31 | up |
| MELO3C010308.2 | 5 | 22 | 2.141 | 0.014660784 | up |
| MELO3C010314.2 | 3144 | 162 | -4.273 | 2.22401E-22 | down |
| MELO3C010316.2 | 581 | 1937 | 1.738 | 1.98233E-31 | up |
| MELO3C010317.2 | 1060 | 148 | -2.839 | 4.7433E-44 | down |
| MELO3C010324.2 | 34 | 6 | -2.577 | 0.004801702 | down |
| MELO3C010332.2 | 78 | 4 | -4.37 | 5.01597E-11 | down |
| MELO3C010341.2 | 82 | 13 | -2.625 | 2.59893E-08 | down |
| MELO3C010348.2 | 8 | 0 | -5.476 | 0.025158349 | down |
| MELO3C010349.2 | 5423 | 2415 | -1.167 | 2.0448E-20 | down |
| MELO3C010353.2 | 3175 | 478 | -2.73 | 2.1436E-07 | down |
| MELO3C010370.2 | 511 | 1403 | 1.458 | 1.1976E-39 | up |
| MELO3C010388.2 | 17 | 559 | 5.055 | 4.18641E-77 | up |
| MELO3C010391.2 | 1 | 104 | 7.173 | 6.87501E-11 | up |
| MELO3C010399.2 | 5 | 0 | -4.975 | 0.008165229 | down |
| MELO3C010403.2 | 533 | 1941 | 1.865 | 4.98175E-24 | up |
| MELO3C010409.2 | 698 | 84 | -3.049 | 2.59004E-18 | down |
| MELO3C010417.2 | 27 | 414 | 3.913 | 5.33632E-26 | up |
| MELO3C010419.2 | 213 | 772 | 1.853 | 1.38167E-24 | up |
| MELO3C010420.2 | 591 | 2013 | 1.768 | 9.36501E-63 | up |
| MELO3C010423.2 | 209 | 435 | 1.058 | 2.39466E-07 | up |
| MELO3C010425.2 | 502 | 143 | -1.81 | 7.97496E-22 | down |
| MELO3C010434.2 | 544 | 237 | -1.197 | 3.43947E-06 | down |
| MELO3C010452.2 | 281 | 37 | -2.904 | 6.34395E-29 | down |
| MELO3C010457.2 | 1 | 26 | 5.212 | 2.98002E-05 | up |
| MELO3C010458.2 | 34 | 6 | -2.618 | 8.3671E-06 | down |
| MELO3C010463.2 | 21 | 49 | 1.208 | 0.004786514 | up |
| MELO3C010470.2 | 1290 | 507 | -1.347 | 9.01278E-23 | down |
| MELO3C010473.2 | 22 | 168 | 2.901 | 6.80705E-16 | up |
| MELO3C010474.2 | 669 | 2376 | 1.829 | 6.42988E-71 | up |
| MELO3C010476.2 | 538 | 1151 | 1.097 | 1.45356E-24 | up |
| MELO3C010492.2 | 362 | 1499 | 2.051 | 6.19266E-51 | up |
| MELO3C010497.2 | 1 | 10 | 3.786 | 0.009984394 | up |
| MELO3C010500.2 | 1851 | 289 | -2.678 | 1.00588E-96 | down |
| MELO3C010506.2 | 128 | 39 | -1.711 | 7.84379E-08 | down |
| MELO3C010512.2 | 1267 | 356 | -1.829 | 8.41822E-58 | down |
| MELO3C010520.2 | 8 | 34 | 2.192 | 0.000301784 | up |
| MELO3C010528.2 | 3 | 72 | 4.335 | 2.0884E-13 | up |
| MELO3C010532.2 | 1995 | 864 | -1.208 | 9.90725E-17 | down |
| MELO3C010537.2 | 1594 | 3394 | 1.09 | 3.87718E-26 | up |
| MELO3C010540.2 | 11 | 253 | 4.46 | 5.19891E-22 | up |
| MELO3C010548.2 | 808 | 3409 | 2.076 | 3.31693E-140 | up |
| MELO3C010561.2 | 179 | 49 | -1.878 | 5.92964E-15 | down |
| MELO3C010588.2 | 73 | 13 | -2.484 | 1.76913E-06 | down |
| MELO3C010598.2 | 368 | 139 | -1.401 | 3.26458E-09 | down |
| MELO3C010604.2 | 42 | 290 | 2.778 | 1.98686E-18 | up |
| MELO3C010605.2 | 113 | 24 | -2.248 | 4.02476E-13 | down |
| MELO3C010608.2 | 19027 | 6645 | -1.518 | 1.65769E-13 | down |
| MELO3C010614.2 | 5619 | 1032 | -2.445 | 5.35752E-109 | down |
| MELO3C010617.2 | 15 | 2 | -3.072 | 0.002653292 | down |
| MELO3C010621.2 | 24 | 159 | 2.704 | 3.63143E-20 | up |
| MELO3C010624.2 | 241 | 4015 | 4.056 | 1.84375E-69 | up |
| MELO3C010632.2 | 1078 | 160 | -2.75 | 1.73782E-41 | down |
| MELO3C010636.2 | 176 | 687 | 1.973 | 4.62929E-31 | up |
| MELO3C010639.2 | 45 | 129 | 1.515 | 0.002953224 | up |
| MELO3C010660.2 | 369 | 1145 | 1.634 | 1.44412E-47 | up |
| MELO3C010672.2 | 890 | 380 | -1.228 | 8.47165E-20 | down |
| MELO3C010673.2 | 60 | 11 | -2.45 | 0.003379001 | down |
| MELO3C010675.2 | 8368 | 20076 | 1.262 | 3.43569E-28 | up |
| MELO3C010682.2 | 0 | 10 | 5.711 | 0.000440146 | up |
| MELO3C010683.2 | 0 | 13 | 6.016 | 7.53114E-05 | up |
| MELO3C010691.2 | 144 | 25 | -2.509 | 3.42188E-15 | down |
| MELO3C010700.2 | 13 | 2 | -2.885 | 0.008550947 | down |
| MELO3C010703.2 | 48 | 12 | -1.952 | 1.76535E-05 | down |
| MELO3C010706.2 | 0 | 20 | 6.66 | 6.4311E-06 | up |
| MELO3C010708.2 | 2861 | 21 | -7.127 | 9.09625E-137 | down |
| MELO3C010711.2 | 1222 | 470 | -1.379 | 2.63693E-18 | down |
| MELO3C010714.2 | 51 | 128 | 1.335 | 0.001424381 | up |
| MELO3C010716.2 | 6195 | 1261 | -2.297 | 4.24757E-52 | down |
| MELO3C010725.2 | 3 | 51 | 3.992 | 7.35021E-09 | up |
| MELO3C010726.2 | 94 | 311 | 1.73 | 1.15663E-12 | up |
| MELO3C010737.2 | 857 | 156 | -2.458 | 9.97075E-37 | down |
| MELO3C010738.2 | 3370 | 965 | -1.805 | 1.39701E-48 | down |
| MELO3C010743.2 | 19 | 74 | 1.96 | 1.52532E-06 | up |
| MELO3C010748.2 | 891 | 354 | -1.332 | 1.15895E-20 | down |
| MELO3C010752.2 | 28 | 11 | -1.366 | 0.013765554 | down |
| MELO3C010755.2 | 272 | 98 | -1.461 | 2.14208E-14 | down |
| MELO3C010759.2 | 1 | 12 | 4.044 | 0.003996766 | up |
| MELO3C010760.2 | 147 | 2570 | 4.126 | 4.2126E-151 | up |
| MELO3C010763.2 | 50212 | 17454 | -1.525 | 7.09469E-53 | down |
| MELO3C010771.2 | 1734 | 850 | -1.028 | 9.94018E-34 | down |
| MELO3C010773.2 | 80 | 4 | -4.219 | 2.50647E-15 | down |
| MELO3C010774.2 | 1394 | 161 | -3.106 | 1.90913E-73 | down |
| MELO3C010776.2 | 10186 | 238 | -5.42 | 1.19615E-267 | down |
| MELO3C010778.2 | 9695 | 4670 | -1.054 | 8.66832E-17 | down |
| MELO3C010779.2 | 2390 | 71 | -5.074 | 2.515E-87 | down |
| MELO3C010782.2 | 39 | 700 | 4.163 | 6.24823E-55 | up |
| MELO3C010784.2 | 5 | 43 | 2.997 | 0.001510925 | up |
| MELO3C010785.2 | 3439 | 1708 | -1.01 | 2.65293E-20 | down |
| MELO3C010786.2 | 88 | 376 | 2.103 | 1.19169E-11 | up |
| MELO3C010787.2 | 12 | 1 | -3.274 | 0.005737328 | down |
| MELO3C010792.2 | 45 | 97 | 1.109 | 0.002444406 | up |
| MELO3C010796.2 | 8 | 656 | 6.303 | 0.002763674 | up |
| MELO3C010810.2 | 7480 | 2584 | -1.533 | 9.20082E-79 | down |
| MELO3C010812.2 | 3245 | 6765 | 1.06 | 4.91042E-30 | up |
| MELO3C010813.2 | 482 | 240 | -1.005 | 4.26988E-05 | down |
| MELO3C010822.2 | 1 | 23 | 5.027 | 5.01606E-05 | up |
| MELO3C010825.2 | 159 | 3 | -5.701 | 6.97615E-23 | down |
| MELO3C010830.2 | 65 | 13 | -2.262 | 3.75746E-07 | down |
| MELO3C010833.2 | 5227 | 1393 | -1.908 | 5.3637E-63 | down |
| MELO3C010840.2 | 939 | 28 | -5.079 | 1.7506E-143 | down |
| MELO3C010843.2 | 51 | 21 | -1.292 | 0.000825732 | down |
| MELO3C010846.2 | 290 | 777 | 1.424 | 4.1709E-10 | up |
| MELO3C010857.2 | 3110 | 6820 | 1.133 | 1.30061E-54 | up |
| MELO3C010858.2 | 442 | 25 | -4.141 | 1.16524E-53 | down |
| MELO3C010860.2 | 744 | 347 | -1.103 | 7.41181E-09 | down |
| MELO3C010861.2 | 812 | 51 | -3.991 | 2.45875E-63 | down |
| MELO3C010863.2 | 1024 | 243 | -2.071 | 8.37596E-31 | down |
| MELO3C010864.2 | 28 | 2 | -3.898 | 8.77802E-06 | down |
| MELO3C010865.2 | 21 | 2 | -3.13 | 0.00019964 | down |
| MELO3C010868.2 | 9 | 87 | 3.222 | 8.02589E-14 | up |
| MELO3C010869.2 | 3380 | 26 | -7.026 | 4.18096E-57 | down |
| MELO3C010870.2 | 44 | 126 | 1.525 | 6.57084E-05 | up |
| MELO3C010872.2 | 8475 | 2327 | -1.865 | 3.98729E-06 | down |
| MELO3C010875.2 | 3200 | 1057 | -1.597 | 1.91073E-16 | down |
| MELO3C010882.2 | 23 | 324 | 3.844 | 2.45706E-38 | up |
| MELO3C010900.2 | 0 | 9 | 5.572 | 0.000736131 | up |
| MELO3C010902.2 | 24 | 51 | 1.121 | 0.006336692 | up |
| MELO3C010908.2 | 3 | 19 | 2.434 | 0.002469012 | up |
| MELO3C010909.2 | 6 | 133 | 4.372 | 3.24223E-17 | up |
| MELO3C010910.2 | 692 | 3321 | 2.263 | 9.4831E-45 | up |
| MELO3C010917.2 | 13 | 2 | -2.835 | 0.00296969 | down |
| MELO3C010918.2 | 633 | 262 | -1.272 | 4.65719E-17 | down |
| MELO3C010922.2 | 346 | 1267 | 1.871 | 2.20294E-73 | up |
| MELO3C010925.2 | 177 | 549 | 1.635 | 1.11973E-09 | up |
| MELO3C010926.2 | 270 | 67 | -2.021 | 6.71204E-14 | down |
| MELO3C010929.2 | 921 | 2031 | 1.14 | 3.463E-22 | up |
| MELO3C010932.2 | 143 | 69 | -1.057 | 4.96288E-05 | down |
| MELO3C010933.2 | 593 | 1858 | 1.649 | 5.0498E-55 | up |
| MELO3C010935.2 | 167 | 443 | 1.412 | 1.02056E-12 | up |
| MELO3C010938.2 | 12513 | 4831 | -1.373 | 1.41968E-40 | down |
| MELO3C010951.2 | 111 | 354 | 1.669 | 1.22444E-06 | up |
| MELO3C010961.2 | 3 | 24 | 2.772 | 0.000276121 | up |
| MELO3C010963.2 | 232 | 63 | -1.879 | 4.35044E-17 | down |
| MELO3C010965.2 | 29 | 96 | 1.745 | 2.74456E-05 | up |
| MELO3C010967.2 | 1200 | 3237 | 1.431 | 8.78324E-20 | up |
| MELO3C010973.2 | 3555 | 1069 | -1.734 | 3.42677E-26 | down |
| MELO3C010974.2 | 176 | 731 | 2.051 | 2.44187E-22 | up |
| MELO3C010976.2 | 4068 | 1876 | -1.116 | 3.67287E-60 | down |
| MELO3C010978.2 | 2 | 36 | 4.087 | 1.45605E-06 | up |
| MELO3C010982.2 | 30623 | 14534 | -1.075 | 1.44933E-24 | down |
| MELO3C011001.2 | 317 | 690 | 1.124 | 1.08635E-19 | up |
| MELO3C011002.2 | 16 | 3 | -2.381 | 0.005593045 | down |
| MELO3C011008.2 | 2221 | 57 | -5.301 | 4.44313E-204 | down |
| MELO3C011013.2 | 335 | 133 | -1.328 | 8.36413E-08 | down |
| MELO3C011016.2 | 148 | 1509 | 3.35 | 3.47935E-48 | up |
| MELO3C011017.2 | 1 | 14 | 4.351 | 0.002333195 | up |
| MELO3C011020.2 | 114 | 446 | 1.962 | 1.87014E-32 | up |
| MELO3C011023.2 | 5205 | 2157 | -1.272 | 1.7592E-13 | down |
| MELO3C011029.2 | 260 | 583 | 1.167 | 4.24803E-14 | up |
| MELO3C011033.2 | 72 | 1 | -6.868 | 3.67396E-10 | down |
| MELO3C011034.2 | 18 | 327 | 4.249 | 1.15085E-38 | up |
| MELO3C011035.2 | 420 | 160 | -1.396 | 0.000911736 | down |
| MELO3C011037.2 | 2263 | 13219 | 2.546 | 6.95807E-36 | up |
| MELO3C011041.2 | 759 | 2641 | 1.799 | 4.31842E-41 | up |
| MELO3C011043.2 | 12 | 1 | -3.671 | 0.004914368 | down |
| MELO3C011051.2 | 54 | 291 | 2.44 | 3.48921E-18 | up |
| MELO3C011055.2 | 66 | 7 | -3.165 | 1.99788E-09 | down |
| MELO3C011056.2 | 309 | 86 | -1.835 | 3.08565E-07 | down |
| MELO3C011064.2 | 17 | 1 | -4.796 | 0.000312306 | down |
| MELO3C011066.2 | 116 | 18 | -2.65 | 5.9467E-12 | down |
| MELO3C011078.2 | 773 | 282 | -1.457 | 4.65709E-29 | down |
| MELO3C011093.2 | 13 | 0 | -6.199 | 5.32269E-05 | down |
| MELO3C011095.2 | 238 | 117 | -1.017 | 4.5408E-06 | down |
| MELO3C011100.2 | 1 | 25 | 4.118 | 0.000263967 | up |
| MELO3C011105.2 | 1117 | 464 | -1.265 | 5.85102E-17 | down |
| MELO3C011113.2 | 4348 | 938 | -2.213 | 8.7955E-76 | down |
| MELO3C011114.2 | 2458 | 399 | -2.621 | 3.79424E-30 | down |
| MELO3C011116.2 | 60 | 13 | -2.175 | 0.021305024 | down |
| MELO3C011117.2 | 199 | 1037 | 2.381 | 8.99492E-30 | up |
| MELO3C011120.2 | 0 | 11 | 4.81 | 0.001585339 | up |
| MELO3C011123.2 | 37 | 8 | -2.21 | 0.000245506 | down |
| MELO3C011125.2 | 41 | 6 | -2.624 | 2.54477E-06 | down |
| MELO3C011129.2 | 6156 | 536 | -3.52 | 7.61352E-256 | down |
| MELO3C011134.2 | 12 | 1 | -3.598 | 0.003019972 | down |
| MELO3C011140.2 | 598 | 1304 | 1.124 | 1.46305E-25 | up |
| MELO3C011146.2 | 80 | 170 | 1.076 | 0.000159434 | up |
| MELO3C011148.2 | 6 | 0 | -5.152 | 0.005405155 | down |
| MELO3C011159.2 | 212 | 656 | 1.627 | 9.18938E-26 | up |
| MELO3C011160.2 | 471 | 1031 | 1.129 | 1.42081E-23 | up |
| MELO3C011164.2 | 354 | 141 | -1.331 | 1.14244E-12 | down |
| MELO3C011177.2 | 3784 | 1055 | -1.844 | 6.21836E-24 | down |
| MELO3C011178.2 | 619 | 133 | -2.223 | 1.01343E-25 | down |
| MELO3C011179.2 | 743 | 2223 | 1.58 | 1.02421E-69 | up |
| MELO3C011180.2 | 36 | 199 | 2.464 | 3.32275E-15 | up |
| MELO3C011181.2 | 437 | 1449 | 1.729 | 1.06189E-54 | up |
| MELO3C011187.2 | 652 | 113 | -2.534 | 1.23598E-46 | down |
| MELO3C011192.2 | 2086 | 342 | -2.606 | 5.72149E-43 | down |
| MELO3C011194.2 | 2311 | 1105 | -1.066 | 4.99398E-27 | down |
| MELO3C011196.2 | 19872 | 7378 | -1.43 | 3.7372E-69 | down |
| MELO3C011207.2 | 81 | 180 | 1.163 | 3.87328E-08 | up |
| MELO3C011209.2 | 189 | 463 | 1.296 | 3.11332E-15 | up |
| MELO3C011210.2 | 32 | 8 | -2.021 | 0.000213955 | down |
| MELO3C011214.2 | 2262 | 756 | -1.581 | 3.26949E-76 | down |
| MELO3C011215.2 | 16 | 4 | -1.811 | 0.025475663 | down |
| MELO3C011217.2 | 922 | 2000 | 1.118 | 4.6492E-20 | up |
| MELO3C011221.2 | 1 | 48 | 5.058 | 2.76897E-08 | up |
| MELO3C011224.2 | 3 | 38 | 3.74 | 5.57527E-07 | up |
| MELO3C011227.2 | 3 | 80 | 4.801 | 2.53191E-14 | up |
| MELO3C011229.2 | 6 | 0 | -5.16 | 0.004616299 | down |
| MELO3C011238.2 | 69 | 21 | -1.725 | 0.000313427 | down |
| MELO3C011240.2 | 26 | 1 | -4.315 | 0.000175327 | down |
| MELO3C011241.2 | 22 | 3 | -2.87 | 0.000383129 | down |
| MELO3C011242.2 | 275 | 42 | -2.721 | 6.26014E-23 | down |
| MELO3C011253.2 | 486 | 1074 | 1.143 | 9.45771E-09 | up |
| MELO3C011257.2 | 893 | 1966 | 1.138 | 2.81318E-45 | up |
| MELO3C011264.2 | 19 | 4 | -2.226 | 0.015159177 | down |
| MELO3C011266.2 | 27 | 6 | -2.119 | 0.000537646 | down |
| MELO3C011268.2 | 5 | 0 | -4.885 | 0.01859716 | down |
| MELO3C011269.2 | 8516 | 2844 | -1.583 | 6.23086E-25 | down |
| MELO3C011270.2 | 190 | 79 | -1.249 | 6.31737E-05 | down |
| MELO3C011271.2 | 2650 | 6774 | 1.354 | 3.04144E-90 | up |
| MELO3C011275.2 | 117 | 39 | -1.615 | 1.22176E-05 | down |
| MELO3C011283.2 | 113 | 1952 | 4.11 | 5.11165E-170 | up |
| MELO3C011289.2 | 93 | 37 | -1.343 | 3.19468E-06 | down |
| MELO3C011293.2 | 17 | 148 | 3.17 | 9.00073E-18 | up |
| MELO3C011296.2 | 90 | 29 | -1.64 | 1.54424E-05 | down |
| MELO3C011298.2 | 8 | 29 | 1.884 | 0.002753055 | up |
| MELO3C011309.2 | 350 | 74 | -2.232 | 8.12605E-33 | down |
| MELO3C011317.2 | 7046 | 2717 | -1.375 | 4.91866E-88 | down |
| MELO3C011329.2 | 9 | 454 | 5.727 | 1.55731E-49 | up |
| MELO3C011338.2 | 132 | 279 | 1.078 | 6.55226E-09 | up |
| MELO3C011340.2 | 18463 | 7128 | -1.373 | 1.54331E-22 | down |
| MELO3C011359.2 | 72 | 209 | 1.537 | 5.19133E-09 | up |
| MELO3C011366.2 | 317 | 6039 | 4.251 | 1.24461E-27 | up |
| MELO3C011368.2 | 1872 | 9286 | 2.311 | 4.15432E-134 | up |
| MELO3C011373.2 | 1006 | 390 | -1.368 | 4.38897E-19 | down |
| MELO3C011387.2 | 94 | 218 | 1.212 | 6.08074E-09 | up |
| MELO3C011389.2 | 1102 | 294 | -1.906 | 3.53034E-12 | down |
| MELO3C011392.2 | 1277 | 517 | -1.302 | 3.51466E-19 | down |
| MELO3C011393.2 | 273 | 123 | -1.154 | 6.05957E-08 | down |
| MELO3C011402.2 | 1504 | 3331 | 1.147 | 2.72812E-58 | up |
| MELO3C011405.2 | 24 | 138 | 2.478 | 1.02427E-13 | up |
| MELO3C011413.2 | 726 | 1809 | 1.317 | 5.91933E-07 | up |
| MELO3C011430.2 | 80 | 183 | 1.189 | 1.96298E-08 | up |
| MELO3C011435.2 | 39729 | 4006 | -3.31 | 9.01628E-25 | down |
| MELO3C011439.2 | 1938 | 6128 | 1.661 | 1.5372E-31 | up |
| MELO3C011441.2 | 122 | 429 | 1.814 | 1.62499E-18 | up |
| MELO3C011443.2 | 26 | 283 | 3.461 | 2.88044E-29 | up |
| MELO3C011450.2 | 1098 | 265 | -2.048 | 1.66631E-75 | down |
| MELO3C011460.2 | 338 | 7 | -5.639 | 8.38264E-50 | down |
| MELO3C011461.2 | 166 | 15 | -3.525 | 4.56284E-20 | down |
| MELO3C011466.2 | 41 | 0 | -7.92 | 3.29009E-09 | down |
| MELO3C011474.2 | 48 | 634 | 3.732 | 5.50721E-08 | up |
| MELO3C011475.2 | 3 | 265 | 6.532 | 2.82536E-23 | up |
| MELO3C011478.2 | 874 | 130 | -2.761 | 1.32901E-56 | down |
| MELO3C011484.2 | 522 | 164 | -1.665 | 3.21222E-14 | down |
| MELO3C011491.2 | 643 | 16 | -5.354 | 4.89465E-104 | down |
| MELO3C011493.2 | 17 | 111 | 2.679 | 0.000558545 | up |
| MELO3C011497.2 | 356 | 766 | 1.104 | 7.25675E-14 | up |
| MELO3C011504.2 | 522 | 174 | -1.592 | 2.42944E-16 | down |
| MELO3C011512.2 | 2427 | 6721 | 1.47 | 2.9103E-50 | up |
| MELO3C011535.2 | 774 | 112 | -2.787 | 4.97856E-74 | down |
| MELO3C011536.2 | 8621 | 3021 | -1.513 | 6.15898E-94 | down |
| MELO3C011541.2 | 29 | 270 | 3.22 | 3.81563E-24 | up |
| MELO3C011546.2 | 44 | 226 | 2.349 | 9.87883E-15 | up |
| MELO3C011550.2 | 261 | 48 | -2.456 | 5.13392E-31 | down |
| MELO3C011553.2 | 826 | 144 | -2.519 | 6.70014E-47 | down |
| MELO3C011560.2 | 47 | 173 | 1.878 | 3.7786E-10 | up |
| MELO3C011570.2 | 173 | 1206 | 2.8 | 1.67211E-67 | up |
| MELO3C011576.2 | 4138 | 24 | -7.448 | 7.62287E-280 | down |
| MELO3C011588.2 | 39 | 16 | -1.314 | 0.012819867 | down |
| MELO3C011610.2 | 14 | 309 | 4.42 | 0.000128891 | up |
| MELO3C011632.2 | 1455 | 713 | -1.028 | 2.06011E-11 | down |
| MELO3C011657.2 | 2167 | 60 | -5.175 | 3.09229E-77 | down |
| MELO3C011674.2 | 1 | 16 | 3.491 | 0.004715062 | up |
| MELO3C011698.2 | 0 | 6 | 5.004 | 0.008624948 | up |
| MELO3C011705.2 | 2769 | 507 | -2.449 | 3.7588E-08 | down |
| MELO3C011706.2 | 3 | 113 | 5.306 | 1.61496E-18 | up |
| MELO3C011720.2 | 2152 | 661 | -1.703 | 7.38603E-30 | down |
| MELO3C011724.2 | 5 | 0 | -4.776 | 0.017634197 | down |
| MELO3C011761.2 | 44 | 937 | 4.416 | 5.15048E-56 | up |
| MELO3C011771.2 | 16 | 775 | 5.621 | 2.10982E-77 | up |
| MELO3C011785.2 | 310 | 705 | 1.187 | 1.07773E-20 | up |
| MELO3C011800.2 | 32 | 147 | 2.227 | 7.99259E-15 | up |
| MELO3C011807.2 | 41 | 8 | -2.32 | 9.85853E-06 | down |
| MELO3C011809.2 | 577 | 2269 | 1.974 | 6.94366E-39 | up |
| MELO3C011810.2 | 24 | 105 | 2.13 | 1.46147E-06 | up |
| MELO3C011815.2 | 2 | 20 | 3.217 | 0.00074957 | up |
| MELO3C011836.2 | 34 | 11 | -1.623 | 0.00835401 | down |
| MELO3C011846.2 | 324 | 149 | -1.123 | 9.53984E-13 | down |
| MELO3C011848.2 | 1020 | 9 | -6.791 | 3.56187E-76 | down |
| MELO3C011849.2 | 821 | 147 | -2.476 | 2.01492E-20 | down |
| MELO3C011850.2 | 10 | 2 | -2.661 | 0.01359229 | down |
| MELO3C011852.2 | 2524 | 7577 | 1.586 | 2.81973E-38 | up |
| MELO3C011855.2 | 105 | 281 | 1.421 | 5.93224E-11 | up |
| MELO3C011861.2 | 145 | 2125 | 3.877 | 1.01445E-228 | up |
| MELO3C011864.2 | 3336 | 784 | -2.09 | 4.22825E-60 | down |
| MELO3C011865.2 | 26703 | 9 | -11.51 | 7.59698E-18 | down |
| MELO3C011868.2 | 15478 | 4425 | -1.807 | 1.34999E-40 | down |
| MELO3C011870.2 | 3 | 25 | 3.107 | 0.000918651 | up |
| MELO3C011872.2 | 8738 | 2272 | -1.943 | 2.19041E-41 | down |
| MELO3C011880.2 | 12 | 3 | -1.97 | 0.02899553 | down |
| MELO3C011883.2 | 871 | 97 | -3.169 | 1.51805E-74 | down |
| MELO3C011885.2 | 1984 | 605 | -1.715 | 2.29891E-33 | down |
| MELO3C011889.2 | 33 | 360 | 3.469 | 6.16023E-25 | up |
| MELO3C011890.2 | 509 | 236 | -1.106 | 5.28646E-14 | down |
| MELO3C011894.2 | 46 | 9 | -2.349 | 1.101E-06 | down |
| MELO3C011902.2 | 115 | 262 | 1.18 | 2.00075E-06 | up |
| MELO3C011905.2 | 2911 | 1217 | -1.257 | 4.94924E-54 | down |
| MELO3C011908.2 | 2850 | 961 | -1.568 | 4.65681E-53 | down |
| MELO3C011909.2 | 74 | 463 | 2.66 | 4.9776E-43 | up |
| MELO3C011911.2 | 12539 | 894 | -3.81 | 9.75185E-147 | down |
| MELO3C011912.2 | 14586 | 5756 | -1.342 | 4.59118E-38 | down |
| MELO3C011916.2 | 327 | 914 | 1.482 | 1.68263E-11 | up |
| MELO3C011925.2 | 188 | 651 | 1.794 | 1.55096E-38 | up |
| MELO3C011928.2 | 16207 | 2559 | -2.663 | 1.98648E-60 | down |
| MELO3C011929.2 | 2636 | 28 | -6.566 | 6.99629E-117 | down |
| MELO3C011930.2 | 70 | 1426 | 4.339 | 2.0237E-12 | up |
| MELO3C011931.2 | 531 | 0 | -11.604 | 1.79671E-22 | down |
| MELO3C011933.2 | 5 | 0 | -4.972 | 0.009769437 | down |
| MELO3C011939.2 | 465 | 211 | -1.137 | 2.73982E-10 | down |
| MELO3C011942.2 | 178 | 49 | -1.862 | 1.03194E-09 | down |
| MELO3C011943.2 | 75 | 5 | -3.959 | 1.62754E-10 | down |
| MELO3C011944.2 | 862 | 414 | -1.058 | 1.92511E-07 | down |
| MELO3C011948.2 | 10 | 125 | 3.635 | 4.1207E-15 | up |
| MELO3C011949.2 | 4700 | 2335 | -1.009 | 9.70447E-22 | down |
| MELO3C011950.2 | 2652 | 930 | -1.512 | 1.85238E-26 | down |
| MELO3C011957.2 | 1314 | 2685 | 1.031 | 1.1608E-10 | up |
| MELO3C011959.2 | 410 | 144 | -1.511 | 8.29708E-11 | down |
| MELO3C011965.2 | 5 | 29 | 2.403 | 0.000293751 | up |
| MELO3C011968.2 | 1305 | 148 | -3.146 | 3.90951E-83 | down |
| MELO3C011970.2 | 483 | 1378 | 1.513 | 7.21241E-14 | up |
| MELO3C011976.2 | 16 | 45 | 1.491 | 0.006395581 | up |
| MELO3C011978.2 | 326 | 154 | -1.085 | 1.24622E-06 | down |
| MELO3C011979.2 | 1872 | 272 | -2.775 | 1.75867E-54 | down |
| MELO3C011980.2 | 2993 | 0 | -14.1 | 1.78629E-32 | down |
| MELO3C011982.2 | 3006 | 959 | -1.649 | 3.40269E-40 | down |
| MELO3C011984.2 | 1630 | 421 | -1.954 | 7.1197E-53 | down |
| MELO3C011985.2 | 15 | 4 | -1.793 | 0.016451689 | down |
| MELO3C011986.2 | 3443 | 459 | -2.907 | 8.75083E-172 | down |
| MELO3C011987.2 | 31 | 73 | 1.221 | 0.008287654 | up |
| MELO3C011988.2 | 518 | 16 | -5.057 | 2.07996E-51 | down |
| MELO3C011993.2 | 51 | 11 | -2.211 | 0.000143547 | down |
| MELO3C011995.2 | 115 | 24 | -2.313 | 5.1118E-11 | down |
| MELO3C011996.2 | 66 | 32 | -1.033 | 0.024046149 | down |
| MELO3C012002.2 | 298 | 77 | -1.941 | 1.25455E-15 | down |
| MELO3C012004.2 | 189 | 412 | 1.126 | 1.06126E-08 | up |
| MELO3C012010.2 | 4207 | 1620 | -1.377 | 1.92838E-43 | down |
| MELO3C012013.2 | 146 | 17 | -3.076 | 8.30726E-17 | down |
| MELO3C012014.2 | 55 | 20 | -1.431 | 0.000142008 | down |
| MELO3C012015.2 | 57 | 11 | -2.367 | 1.59694E-05 | down |
| MELO3C012020.2 | 740 | 116 | -2.681 | 1.17066E-57 | down |
| MELO3C012023.2 | 2720 | 11239 | 2.047 | 1.45698E-36 | up |
| MELO3C012024.2 | 26 | 88 | 1.771 | 6.70157E-07 | up |
| MELO3C012027.2 | 24 | 539 | 4.491 | 2.15991E-56 | up |
| MELO3C012034.2 | 143 | 38 | -1.922 | 1.28204E-08 | down |
| MELO3C012039.2 | 89 | 22 | -1.987 | 8.2278E-07 | down |
| MELO3C012049.2 | 215 | 1009 | 2.227 | 5.28028E-36 | up |
| MELO3C012053.2 | 108 | 330 | 1.609 | 0.000211828 | up |
| MELO3C012055.2 | 5283 | 623 | -3.082 | 1.12832E-45 | down |
| MELO3C012064.2 | 75 | 19 | -1.973 | 0.029348007 | down |
| MELO3C012065.2 | 198 | 77 | -1.359 | 5.95682E-07 | down |
| MELO3C012075.2 | 233 | 1542 | 2.728 | 9.50943E-62 | up |
| MELO3C012078.2 | 2781 | 380 | -2.873 | 1.29091E-76 | down |
| MELO3C012079.2 | 2624 | 6687 | 1.35 | 3.49046E-81 | up |
| MELO3C012082.2 | 930 | 140 | -2.737 | 3.53811E-96 | down |
| MELO3C012093.2 | 31 | 7 | -2.21 | 0.000316355 | down |
| MELO3C012094.2 | 165 | 13 | -3.66 | 5.73836E-17 | down |
| MELO3C012095.2 | 279 | 131 | -1.092 | 1.33461E-10 | down |
| MELO3C012103.2 | 2592 | 820 | -1.66 | 5.78E-63 | down |
| MELO3C012114.2 | 30 | 2 | -4.022 | 6.8432E-06 | down |
| MELO3C012124.2 | 468 | 996 | 1.091 | 4.71716E-23 | up |
| MELO3C012127.2 | 63 | 4 | -3.864 | 1.47891E-11 | down |
| MELO3C012128.2 | 63 | 147 | 1.21 | 2.96495E-05 | up |
| MELO3C012133.2 | 32 | 144 | 2.147 | 2.56266E-10 | up |
| MELO3C012134.2 | 4421 | 2179 | -1.02 | 1.06896E-31 | down |
| MELO3C012135.2 | 1118 | 457 | -1.291 | 0.0078531 | down |
| MELO3C012137.2 | 47 | 10 | -2.306 | 1.76898E-05 | down |
| MELO3C012139.2 | 324 | 1835 | 2.499 | 2.33255E-108 | up |
| MELO3C012142.2 | 98 | 344 | 1.815 | 9.28719E-15 | up |
| MELO3C012148.2 | 186 | 42 | -2.162 | 3.57202E-13 | down |
| MELO3C012149.2 | 348 | 1222 | 1.812 | 8.78744E-30 | up |
| MELO3C012151.2 | 38 | 5 | -2.793 | 2.93115E-05 | down |
| MELO3C012155.2 | 434 | 209 | -1.059 | 3.2077E-11 | down |
| MELO3C012158.2 | 100 | 333 | 1.74 | 4.74875E-14 | up |
| MELO3C012160.2 | 171 | 381 | 1.159 | 5.41778E-07 | up |
| MELO3C012161.2 | 1 | 8 | 3.529 | 0.017986819 | up |
| MELO3C012162.2 | 32 | 411 | 3.661 | 2.15962E-06 | up |
| MELO3C012166.2 | 91 | 28 | -1.689 | 1.01606E-05 | down |
| MELO3C012167.2 | 307 | 987 | 1.686 | 1.05311E-16 | up |
| MELO3C012168.2 | 21 | 1 | -5.09 | 7.4618E-05 | down |
| MELO3C012169.2 | 173 | 500 | 1.53 | 2.85537E-21 | up |
| MELO3C012175.2 | 1260 | 347 | -1.86 | 9.2858E-62 | down |
| MELO3C012176.2 | 33 | 68 | 1.051 | 0.002305443 | up |
| MELO3C012178.2 | 1343 | 662 | -1.021 | 8.71122E-12 | down |
| MELO3C012180.2 | 1748 | 563 | -1.635 | 1.04031E-54 | down |
| MELO3C012181.2 | 262 | 23 | -3.496 | 4.25788E-37 | down |
| MELO3C012186.2 | 138 | 372 | 1.425 | 4.75081E-06 | up |
| MELO3C012190.2 | 4 | 0 | -4.654 | 0.023802888 | down |
| MELO3C012196.2 | 4092 | 9052 | 1.145 | 1.27681E-47 | up |
| MELO3C012200.2 | 461 | 51 | -3.171 | 1.27305E-34 | down |
| MELO3C012203.2 | 140 | 12 | -3.535 | 1.45723E-21 | down |
| MELO3C012204.2 | 92 | 10 | -3.215 | 9.94097E-12 | down |
| MELO3C012209.2 | 4460 | 1845 | -1.274 | 1.32612E-28 | down |
| MELO3C012218.2 | 171 | 460 | 1.433 | 5.7874E-05 | up |
| MELO3C012222.2 | 4 | 533 | 7.08 | 4.87881E-48 | up |
| MELO3C012231.2 | 29 | 111 | 1.937 | 1.04178E-07 | up |
| MELO3C012240.2 | 113 | 48 | -1.225 | 2.85657E-06 | down |
| MELO3C012241.2 | 3 | 29 | 3.343 | 1.35039E-05 | up |
| MELO3C012242.2 | 70 | 249 | 1.832 | 3.16289E-16 | up |
| MELO3C012244.2 | 131 | 286 | 1.123 | 4.15475E-08 | up |
| MELO3C012252.2 | 41226 | 13604 | -1.6 | 3.12569E-35 | down |
| MELO3C012253.2 | 2331 | 946 | -1.302 | 1.28199E-31 | down |
| MELO3C012254.2 | 3818 | 1457 | -1.39 | 1.48066E-28 | down |
| MELO3C012261.2 | 267 | 1292 | 2.275 | 2.67565E-52 | up |
| MELO3C012270.2 | 75 | 27 | -1.483 | 6.47588E-06 | down |
| MELO3C012273.2 | 27 | 1033 | 5.266 | 6.177E-149 | up |
| MELO3C012278.2 | 1934 | 4339 | 1.165 | 2.56848E-17 | up |
| MELO3C012286.2 | 362 | 766 | 1.082 | 3.19592E-11 | up |
| MELO3C012290.2 | 69 | 10 | -2.796 | 4.68078E-08 | down |
| MELO3C012307.2 | 153 | 15 | -3.353 | 8.9033E-23 | down |
| MELO3C012316.2 | 0 | 9 | 5.536 | 0.000763092 | up |
| MELO3C012322.2 | 338 | 716 | 1.081 | 7.16665E-08 | up |
| MELO3C012324.2 | 11845 | 4412 | -1.425 | 9.43657E-15 | down |
| MELO3C012334.2 | 506 | 1294 | 1.354 | 1.82096E-14 | up |
| MELO3C012336.2 | 36 | 125 | 1.793 | 1.96125E-10 | up |
| MELO3C012340.2 | 220 | 92 | -1.257 | 0.000157084 | down |
| MELO3C012344.2 | 1 | 14 | 4.326 | 0.000781956 | up |
| MELO3C012345.2 | 235 | 74 | -1.66 | 3.9028E-12 | down |
| MELO3C012346.2 | 67 | 9 | -2.855 | 1.81234E-09 | down |
| MELO3C012348.2 | 851 | 374 | -1.186 | 4.32548E-12 | down |
| MELO3C012349.2 | 38 | 164 | 2.102 | 1.12397E-12 | up |
| MELO3C012372.2 | 8 | 380 | 5.592 | 6.31801E-57 | up |
| MELO3C012382.2 | 5652 | 2269 | -1.317 | 5.19921E-12 | down |
| MELO3C012388.2 | 80 | 28 | -1.518 | 0.000823671 | down |
| MELO3C012389.2 | 16556 | 6563 | -1.335 | 2.44771E-60 | down |
| MELO3C012403.2 | 17 | 43 | 1.343 | 0.003257377 | up |
| MELO3C012404.2 | 455 | 130 | -1.809 | 5.08262E-23 | down |
| MELO3C012409.2 | 5492 | 1423 | -1.948 | 1.29192E-30 | down |
| MELO3C012412.2 | 164 | 77 | -1.095 | 0.000105795 | down |
| MELO3C012418.2 | 11 | 1 | -4.127 | 0.0041221 | down |
| MELO3C012421.2 | 2417 | 998 | -1.276 | 1.08619E-19 | down |
| MELO3C012424.2 | 3 | 17 | 2.227 | 0.007178431 | up |
| MELO3C012426.2 | 717 | 2187 | 1.608 | 6.69718E-45 | up |
| MELO3C012433.2 | 46 | 902 | 4.309 | 9.33017E-41 | up |
| MELO3C012437.2 | 996 | 205 | -2.281 | 1.77632E-57 | down |
| MELO3C012438.2 | 788 | 25 | -5.016 | 4.30712E-105 | down |
| MELO3C012439.2 | 570 | 178 | -1.68 | 1.90901E-07 | down |
| MELO3C012440.2 | 140 | 356 | 1.349 | 8.66411E-13 | up |
| MELO3C012442.2 | 748 | 225 | -1.733 | 2.33675E-41 | down |
| MELO3C012452.2 | 90 | 228 | 1.329 | 2.44021E-10 | up |
| MELO3C012453.2 | 32 | 73 | 1.183 | 0.004544838 | up |
| MELO3C012454.2 | 130 | 2802 | 4.43 | 7.26155E-21 | up |
| MELO3C012455.2 | 183 | 43 | -2.08 | 1.18359E-15 | down |
| MELO3C012456.2 | 467 | 174 | -1.421 | 1.22421E-16 | down |
| MELO3C012470.2 | 497 | 22 | -4.458 | 1.07244E-33 | down |
| MELO3C012471.2 | 509 | 1 | -9.688 | 1.21314E-20 | down |
| MELO3C012475.2 | 254 | 595 | 1.226 | 8.38671E-16 | up |
| MELO3C012476.2 | 85 | 23 | -1.885 | 7.33947E-06 | down |
| MELO3C012479.2 | 11734 | 5603 | -1.066 | 8.87181E-15 | down |
| MELO3C012484.2 | 284 | 84 | -1.751 | 7.84502E-08 | down |
| MELO3C012494.2 | 3687 | 1689 | -1.127 | 1.8733E-14 | down |
| MELO3C012498.2 | 38 | 6 | -2.674 | 1.58113E-05 | down |
| MELO3C012502.2 | 0 | 459 | 11.182 | 8.18154E-21 | up |
| MELO3C012508.2 | 7 | 56 | 3.033 | 2.00004E-06 | up |
| MELO3C012525.2 | 307 | 1919 | 2.643 | 2.52487E-64 | up |
| MELO3C012547.2 | 1839 | 21 | -6.45 | 2.15108E-179 | down |
| MELO3C012556.2 | 943 | 196 | -2.264 | 1.53538E-19 | down |
| MELO3C012559.2 | 2191 | 172 | -3.672 | 1.2952E-94 | down |
| MELO3C012570.2 | 3 | 19 | 2.712 | 0.003579814 | up |
| MELO3C012573.2 | 16 | 5 | -1.584 | 0.027978049 | down |
| MELO3C012590.2 | 1420 | 289 | -2.297 | 6.61457E-42 | down |
| MELO3C012594.2 | 1 | 21 | 4.301 | 0.000237793 | up |
| MELO3C012595.2 | 55 | 14 | -1.944 | 0.002641357 | down |
| MELO3C012601.2 | 83 | 316 | 1.93 | 1.41546E-16 | up |
| MELO3C012603.2 | 19 | 44 | 1.197 | 0.007972349 | up |
| MELO3C012614.2 | 1020 | 356 | -1.523 | 2.18226E-16 | down |
| MELO3C012619.2 | 1 | 17 | 3.563 | 0.00298012 | up |
| MELO3C012628.2 | 1774 | 4189 | 1.24 | 1.49654E-32 | up |
| MELO3C012630.2 | 169 | 35 | -2.299 | 0.000438357 | down |
| MELO3C012631.2 | 347 | 1529 | 2.138 | 3.3784E-50 | up |
| MELO3C012645.2 | 2751 | 369 | -2.897 | 1.15743E-83 | down |
| MELO3C012679.2 | 6 | 0 | -5.062 | 0.009660222 | down |
| MELO3C012686.2 | 1 | 18 | 4.655 | 0.00028556 | up |
| MELO3C012699.2 | 625 | 233 | -1.425 | 3.74577E-27 | down |
| MELO3C012701.2 | 17288 | 1874 | -3.205 | 1.8624E-17 | down |
| MELO3C012702.2 | 7947 | 487 | -4.027 | 5.41746E-18 | down |
| MELO3C012712.2 | 716 | 9 | -6.328 | 3.75142E-70 | down |
| MELO3C012714.2 | 105 | 595 | 2.5 | 1.00707E-60 | up |
| MELO3C012716.2 | 1928 | 4251 | 1.141 | 3.08923E-28 | up |
| MELO3C012717.2 | 507 | 1194 | 1.234 | 4.67032E-22 | up |
| MELO3C012724.2 | 1 | 13 | 4.211 | 0.001885944 | up |
| MELO3C012727.2 | 278 | 112 | -1.299 | 1.25364E-05 | down |
| MELO3C012728.2 | 2344 | 1126 | -1.058 | 2.53579E-25 | down |
| MELO3C012740.2 | 469 | 234 | -1.003 | 4.43015E-09 | down |
| MELO3C012746.2 | 88 | 351 | 2 | 1.96114E-20 | up |
| MELO3C012753.2 | 29 | 217 | 2.9 | 1.35769E-16 | up |
| MELO3C012791.2 | 10 | 31 | 1.637 | 0.001570744 | up |
| MELO3C012802.2 | 7 | 0 | -5.46 | 0.001693125 | down |
| MELO3C012806.2 | 0 | 3315 | 14.035 | 6.53509E-22 | up |
| MELO3C012822.2 | 197 | 889 | 2.172 | 4.20687E-62 | up |
| MELO3C012832.2 | 302 | 140 | -1.104 | 1.52163E-10 | down |
| MELO3C012838.2 | 41 | 244 | 2.582 | 1.32421E-21 | up |
| MELO3C012843.2 | 2173 | 908 | -1.26 | 1.53078E-42 | down |
| MELO3C012852.2 | 22 | 118 | 2.456 | 3.93202E-12 | up |
| MELO3C012854.2 | 1060 | 83 | -3.659 | 3.47971E-73 | down |
| MELO3C012870.2 | 1 | 218 | 8.253 | 4.75667E-15 | up |
| MELO3C012874.2 | 137 | 1102 | 3.013 | 5.66723E-60 | up |
| MELO3C012885.2 | 3221 | 961 | -1.745 | 3.03869E-55 | down |
| MELO3C012902.2 | 1904 | 744 | -1.357 | 4.14297E-31 | down |
| MELO3C012906.2 | 21 | 182 | 3.129 | 3.93104E-23 | up |
| MELO3C012911.2 | 7349 | 24149 | 1.716 | 1.9724E-56 | up |
| MELO3C012912.2 | 87568 | 65 | -10.416 | 0 | down |
| MELO3C012916.2 | 6 | 0 | -5.251 | 0.023767985 | down |
| MELO3C012917.2 | 10 | 164 | 3.982 | 1.36479E-16 | up |
| MELO3C012920.2 | 78 | 1353 | 4.115 | 2.95111E-53 | up |
| MELO3C012921.2 | 193 | 745 | 1.945 | 1.01558E-32 | up |
| MELO3C012925.2 | 2769 | 6765 | 1.289 | 1.07186E-40 | up |
| MELO3C012928.2 | 114 | 337 | 1.561 | 3.59268E-06 | up |
| MELO3C012939.2 | 5397 | 2302 | -1.23 | 5.48867E-17 | down |
| MELO3C012944.2 | 38 | 333 | 3.105 | 2.57495E-45 | up |
| MELO3C012945.2 | 406 | 28 | -3.884 | 8.22728E-44 | down |
| MELO3C012956.2 | 660 | 221 | -1.576 | 1.55811E-18 | down |
| MELO3C012958.2 | 273 | 120 | -1.19 | 3.15709E-07 | down |
| MELO3C012960.2 | 7016 | 899 | -2.964 | 1.38366E-158 | down |
| MELO3C012962.2 | 86 | 658 | 2.934 | 6.99634E-27 | up |
| MELO3C012966.2 | 341 | 129 | -1.411 | 7.39696E-06 | down |
| MELO3C012968.2 | 554 | 179 | -1.632 | 1.32413E-19 | down |
| MELO3C012969.2 | 596 | 1306 | 1.131 | 5.83272E-23 | up |
| MELO3C012970.2 | 111 | 2 | -5.5 | 5.287E-15 | down |
| MELO3C012971.2 | 24 | 3 | -3.236 | 0.000273027 | down |
| MELO3C012974.2 | 588 | 1188 | 1.014 | 1.21532E-23 | up |
| MELO3C012982.2 | 141 | 337 | 1.254 | 7.66044E-08 | up |
| MELO3C012987.2 | 71 | 12 | -2.589 | 8.17947E-05 | down |
| MELO3C012992.2 | 233 | 874 | 1.904 | 1.72467E-44 | up |
| MELO3C012994.2 | 1053 | 47 | -4.499 | 8.50338E-93 | down |
| MELO3C013000.2 | 80292 | 27176 | -1.563 | 7.71925E-79 | down |
| MELO3C013002.2 | 1301 | 551 | -1.24 | 1.76129E-18 | down |
| MELO3C013003.2 | 138 | 45 | -1.604 | 8.73811E-09 | down |
| MELO3C013005.2 | 34 | 117 | 1.782 | 1.58635E-07 | up |
| MELO3C013006.2 | 192 | 19 | -3.34 | 7.74753E-23 | down |
| MELO3C013013.2 | 953 | 5403 | 2.504 | 1.15981E-36 | up |
| MELO3C013014.2 | 381 | 22 | -4.14 | 6.36769E-51 | down |
| MELO3C013018.2 | 4 | 0 | -4.656 | 0.023712809 | down |
| MELO3C013034.2 | 4471 | 1418 | -1.657 | 2.21599E-104 | down |
| MELO3C013047.2 | 273 | 10 | -4.72 | 1.49369E-31 | down |
| MELO3C013057.2 | 57 | 4 | -3.969 | 6.91074E-10 | down |
| MELO3C013082.2 | 4447 | 2057 | -1.112 | 6.35244E-16 | down |
| MELO3C013090.2 | 0 | 67 | 8.4 | 1.74625E-11 | up |
| MELO3C013099.2 | 8 | 0 | -5.656 | 0.000900832 | down |
| MELO3C013100.2 | 14 | 122 | 3.122 | 1.73693E-08 | up |
| MELO3C013103.2 | 1 | 38 | 5.706 | 4.54667E-05 | up |
| MELO3C013111.2 | 7 | 616 | 6.429 | 8.45089E-66 | up |
| MELO3C013114.2 | 494 | 8 | -6.054 | 3.5165E-67 | down |
| MELO3C013132.2 | 65 | 177 | 1.454 | 3.49058E-08 | up |
| MELO3C013152.2 | 364 | 860 | 1.241 | 1.80365E-28 | up |
| MELO3C013157.2 | 1388 | 15 | -6.494 | 5.31758E-166 | down |
| MELO3C013158.2 | 1 | 333 | 7.861 | 7.39229E-26 | up |
| MELO3C013159.2 | 1316 | 610 | -1.109 | 8.91339E-06 | down |
| MELO3C013168.2 | 210 | 513 | 1.288 | 7.08866E-08 | up |
| MELO3C013175.2 | 1347 | 2705 | 1.006 | 1.99828E-26 | up |
| MELO3C013186.2 | 289 | 643 | 1.153 | 1.16579E-17 | up |
| MELO3C013188.2 | 489 | 1377 | 1.494 | 2.01074E-38 | up |
| MELO3C013195.2 | 34 | 161 | 2.242 | 5.49694E-11 | up |
| MELO3C013203.2 | 750 | 290 | -1.369 | 3.17754E-06 | down |
| MELO3C013225.2 | 1170 | 2414 | 1.045 | 1.85384E-24 | up |
| MELO3C013232.2 | 202 | 702 | 1.794 | 4.35734E-23 | up |
| MELO3C013246.2 | 22 | 2 | -3.62 | 9.76171E-05 | down |
| MELO3C013250.2 | 5906 | 2395 | -1.302 | 3.42285E-47 | down |
| MELO3C013254.2 | 2001 | 934 | -1.1 | 2.42684E-14 | down |
| MELO3C013264.2 | 152 | 651 | 2.1 | 2.53715E-10 | up |
| MELO3C013273.2 | 19 | 93 | 2.291 | 3.49062E-09 | up |
| MELO3C013274.2 | 36 | 78 | 1.121 | 0.000506007 | up |
| MELO3C013289.2 | 81 | 561 | 2.796 | 4.32926E-14 | up |
| MELO3C013302.2 | 32 | 6 | -2.512 | 0.000142001 | down |
| MELO3C013306.2 | 3457 | 255 | -3.762 | 1.2027E-176 | down |
| MELO3C013308.2 | 63 | 451 | 2.833 | 3.21826E-34 | up |
| MELO3C013310.2 | 17 | 0 | -6.599 | 1.49587E-05 | down |
| MELO3C013320.2 | 6210 | 2601 | -1.256 | 4.98782E-26 | down |
| MELO3C013322.2 | 515 | 219 | -1.236 | 2.37029E-13 | down |
| MELO3C013324.2 | 382 | 838 | 1.133 | 1.56148E-12 | up |
| MELO3C013347.2 | 7708 | 270 | -4.833 | 3.05478E-196 | down |
| MELO3C013348.2 | 328 | 129 | -1.35 | 1.58249E-14 | down |
| MELO3C013350.2 | 15948 | 5661 | -1.494 | 4.7133E-35 | down |
| MELO3C013360.2 | 143 | 6 | -4.61 | 8.69259E-17 | down |
| MELO3C013361.2 | 75 | 363 | 2.273 | 4.41929E-22 | up |
| MELO3C013366.2 | 13664 | 2091 | -2.707 | 1.02303E-49 | down |
| MELO3C013376.2 | 65 | 260 | 1.988 | 1.42693E-08 | up |
| MELO3C013387.2 | 15 | 102 | 2.795 | 9.28068E-12 | up |
| MELO3C013403.2 | 62 | 13 | -2.286 | 2.54282E-08 | down |
| MELO3C013411.2 | 251 | 96 | -1.38 | 3.51144E-08 | down |
| MELO3C013427.2 | 21 | 2 | -3.513 | 0.002336101 | down |
| MELO3C013428.2 | 591 | 266 | -1.15 | 2.74903E-14 | down |
| MELO3C013429.2 | 468 | 1045 | 1.159 | 1.81677E-10 | up |
| MELO3C013436.2 | 59 | 421 | 2.834 | 4.25872E-28 | up |
| MELO3C013439.2 | 1 | 88 | 6.943 | 5.58752E-10 | up |
| MELO3C013445.2 | 8196 | 3476 | -1.238 | 4.23363E-50 | down |
| MELO3C013449.2 | 2586 | 38 | -6.088 | 1.95967E-145 | down |
| MELO3C013451.2 | 0 | 8747 | 15.435 | 1.65381E-38 | up |
| MELO3C013453.2 | 147 | 53 | -1.462 | 1.4386E-08 | down |
| MELO3C013455.2 | 1297 | 3034 | 1.226 | 3.29941E-44 | up |
| MELO3C013478.2 | 37 | 7 | -2.347 | 2.75247E-05 | down |
| MELO3C013480.2 | 804 | 281 | -1.515 | 2.00097E-09 | down |
| MELO3C013487.2 | 48 | 620 | 3.683 | 6.64984E-57 | up |
| MELO3C013495.2 | 296 | 914 | 1.629 | 1.04948E-13 | up |
| MELO3C013512.2 | 1806 | 637 | -1.503 | 6.85726E-18 | down |
| MELO3C013519.2 | 726 | 302 | -1.262 | 6.92955E-18 | down |
| MELO3C013524.2 | 723 | 223 | -1.699 | 4.27879E-27 | down |
| MELO3C013538.2 | 19 | 3 | -2.585 | 0.002225643 | down |
| MELO3C013544.2 | 132 | 10 | -3.674 | 3.94766E-15 | down |
| MELO3C013552.2 | 32 | 206 | 2.698 | 6.88136E-17 | up |
| MELO3C013553.2 | 423 | 113 | -1.905 | 2.93593E-18 | down |
| MELO3C013561.2 | 19 | 57 | 1.614 | 0.000102006 | up |
| MELO3C013566.2 | 11758 | 2582 | -2.187 | 2.78676E-60 | down |
| MELO3C013570.2 | 65 | 717 | 3.469 | 6.76798E-63 | up |
| MELO3C013577.2 | 111 | 46 | -1.262 | 4.37888E-06 | down |
| MELO3C013583.2 | 2155 | 7990 | 1.89 | 5.23485E-133 | up |
| MELO3C013586.2 | 34 | 92 | 1.417 | 8.22828E-06 | up |
| MELO3C013592.2 | 89 | 34 | -1.374 | 0.002561572 | down |
| MELO3C013594.2 | 7 | 1 | -3.54 | 0.025274875 | down |
| MELO3C013603.2 | 558 | 1131 | 1.02 | 9.0895E-07 | up |
| MELO3C013606.2 | 3 | 50 | 4.116 | 5.23094E-09 | up |
| MELO3C013612.2 | 38 | 84 | 1.151 | 0.000518051 | up |
| MELO3C013615.2 | 1990 | 347 | -2.522 | 9.37986E-96 | down |
| MELO3C013618.2 | 233 | 28 | -3.083 | 3.91255E-20 | down |
| MELO3C013622.2 | 103 | 952 | 3.214 | 2.15247E-54 | up |
| MELO3C013623.2 | 539 | 144 | -1.902 | 5.39685E-21 | down |
| MELO3C013625.2 | 148 | 35 | -2.079 | 1.44117E-13 | down |
| MELO3C013632.2 | 2648 | 547 | -2.276 | 4.04504E-38 | down |
| MELO3C013641.2 | 105 | 800 | 2.937 | 1.9774E-38 | up |
| MELO3C013647.2 | 855 | 0 | -12.293 | 3.74873E-25 | down |
| MELO3C013665.2 | 209 | 1654 | 2.983 | 3.45179E-67 | up |
| MELO3C013667.2 | 499 | 1132 | 1.183 | 2.15803E-29 | up |
| MELO3C013669.2 | 797 | 25 | -4.976 | 1.44371E-96 | down |
| MELO3C013672.2 | 439 | 1154 | 1.394 | 1.73543E-34 | up |
| MELO3C013677.2 | 767 | 47 | -4.008 | 1.29084E-47 | down |
| MELO3C013679.2 | 39 | 7 | -2.535 | 0.013367219 | down |
| MELO3C013681.2 | 267 | 558 | 1.063 | 4.41924E-14 | up |
| MELO3C013682.2 | 11 | 319 | 4.89 | 2.17079E-26 | up |
| MELO3C013686.2 | 72 | 866 | 3.595 | 5.83316E-38 | up |
| MELO3C013687.2 | 519 | 1221 | 1.235 | 1.40004E-10 | up |
| MELO3C013692.2 | 836 | 372 | -1.167 | 7.15572E-23 | down |
| MELO3C013693.2 | 52 | 5 | -3.235 | 2.45404E-06 | down |
| MELO3C013699.2 | 396 | 2435 | 2.62 | 6.66346E-32 | up |
| MELO3C013708.2 | 408 | 127 | -1.68 | 3.70556E-21 | down |
| MELO3C013710.2 | 1138 | 323 | -1.815 | 5.26731E-15 | down |
| MELO3C013715.2 | 797 | 2049 | 1.363 | 8.59182E-27 | up |
| MELO3C013726.2 | 859 | 2283 | 1.411 | 9.43036E-22 | up |
| MELO3C013727.2 | 936 | 4460 | 2.252 | 4.46692E-29 | up |
| MELO3C013737.2 | 27 | 74 | 1.472 | 6.82652E-05 | up |
| MELO3C013751.2 | 175 | 709 | 2.02 | 9.87239E-27 | up |
| MELO3C013752.2 | 69 | 299 | 2.109 | 5.64295E-12 | up |
| MELO3C013761.2 | 301 | 882 | 1.552 | 4.32895E-39 | up |
| MELO3C013762.2 | 272 | 598 | 1.139 | 0.000115348 | up |
| MELO3C013771.2 | 924 | 136 | -2.759 | 1.60801E-47 | down |
| MELO3C013774.2 | 5554 | 940 | -2.562 | 1.03608E-90 | down |
| MELO3C013778.2 | 463 | 222 | -1.062 | 1.75689E-07 | down |
| MELO3C013780.2 | 143 | 298 | 1.059 | 3.40131E-08 | up |
| MELO3C013782.2 | 0 | 99 | 8.962 | 3.69665E-13 | up |
| MELO3C013800.2 | 35 | 10 | -1.732 | 0.001305371 | down |
| MELO3C013801.2 | 2727 | 706 | -1.95 | 1.70318E-55 | down |
| MELO3C013805.2 | 175 | 378 | 1.111 | 7.38079E-07 | up |
| MELO3C013807.2 | 11 | 38 | 1.831 | 0.000696724 | up |
| MELO3C013817.2 | 933 | 202 | -2.207 | 7.5982E-49 | down |
| MELO3C013821.2 | 1860 | 3972 | 1.095 | 5.47275E-34 | up |
| MELO3C013829.2 | 17 | 37 | 1.071 | 0.022523516 | up |
| MELO3C013841.2 | 3216 | 1599 | -1.008 | 1.76594E-33 | down |
| MELO3C013842.2 | 192 | 436 | 1.18 | 2.56246E-12 | up |
| MELO3C013843.2 | 489 | 230 | -1.087 | 5.84068E-13 | down |
| MELO3C013845.2 | 26 | 0 | -7.219 | 9.54297E-08 | down |
| MELO3C013852.2 | 844 | 357 | -1.238 | 7.5668E-21 | down |
| MELO3C013859.2 | 7567 | 25 | -8.239 | 1.57997E-288 | down |
| MELO3C013862.2 | 741 | 223 | -1.729 | 7.84944E-16 | down |
| MELO3C013867.2 | 141 | 577 | 2.033 | 1.17898E-18 | up |
| MELO3C013868.2 | 0 | 3213 | 13.99 | 1.22494E-31 | up |
| MELO3C013872.2 | 927 | 394 | -1.234 | 8.28171E-12 | down |
| MELO3C013875.2 | 6533 | 16556 | 1.342 | 1.95083E-29 | up |
| MELO3C013880.2 | 1350 | 111 | -3.601 | 4.17431E-55 | down |
| MELO3C013885.2 | 5679 | 2344 | -1.278 | 1.31524E-38 | down |
| MELO3C013887.2 | 788 | 316 | -1.317 | 7.49402E-13 | down |
| MELO3C013889.2 | 140 | 407 | 1.535 | 1.17522E-08 | up |
| MELO3C013893.2 | 337 | 97 | -1.791 | 1.98524E-12 | down |
| MELO3C013898.2 | 880 | 1811 | 1.041 | 2.65081E-22 | up |
| MELO3C013905.2 | 324 | 123 | -1.404 | 1.47637E-14 | down |
| MELO3C013906.2 | 2213 | 351 | -2.656 | 1.70172E-60 | down |
| MELO3C013907.2 | 392 | 92 | -2.085 | 1.60085E-14 | down |
| MELO3C013919.2 | 70 | 2 | -5.256 | 1.28589E-12 | down |
| MELO3C013922.2 | 468 | 104 | -2.163 | 1.45132E-15 | down |
| MELO3C013924.2 | 296 | 145 | -1.031 | 5.97378E-08 | down |
| MELO3C013925.2 | 14828 | 3760 | -1.98 | 2.0337E-91 | down |
| MELO3C013926.2 | 39 | 370 | 3.255 | 4.54317E-23 | up |
| MELO3C013927.2 | 1225 | 607 | -1.013 | 4.06266E-12 | down |
| MELO3C013934.2 | 36 | 3 | -3.55 | 5.27372E-07 | down |
| MELO3C013935.2 | 183 | 2 | -6.898 | 3.87828E-23 | down |
| MELO3C013941.2 | 466 | 222 | -1.064 | 4.98072E-09 | down |
| MELO3C013943.2 | 856 | 2505 | 1.548 | 5.85976E-22 | up |
| MELO3C013945.2 | 8226 | 2733 | -1.59 | 1.11668E-48 | down |
| MELO3C013946.2 | 374 | 907 | 1.278 | 8.6295E-15 | up |
| MELO3C013961.2 | 557 | 3319 | 2.576 | 1.37556E-110 | up |
| MELO3C013962.2 | 0 | 17 | 6.438 | 1.10178E-05 | up |
| MELO3C013964.2 | 3590 | 1182 | -1.604 | 7.93545E-36 | down |
| MELO3C013968.2 | 748 | 1765 | 1.238 | 4.96203E-42 | up |
| MELO3C013969.2 | 1372 | 457 | -1.588 | 4.31295E-44 | down |
| MELO3C013972.2 | 44 | 196 | 2.167 | 8.89112E-16 | up |
| MELO3C013973.2 | 262 | 549 | 1.07 | 3.78364E-11 | up |
| MELO3C013989.2 | 48 | 164 | 1.763 | 2.68381E-10 | up |
| MELO3C014000.2 | 1002 | 466 | -1.105 | 8.48677E-19 | down |
| MELO3C014002.2 | 9 | 76 | 3.144 | 2.87391E-06 | up |
| MELO3C014004.2 | 12 | 42 | 1.778 | 0.000252108 | up |
| MELO3C014005.2 | 100 | 1547 | 3.953 | 9.00538E-114 | up |
| MELO3C014007.2 | 1206 | 2670 | 1.147 | 4.89932E-19 | up |
| MELO3C014009.2 | 6743 | 2759 | -1.289 | 4.83155E-46 | down |
| MELO3C014015.2 | 8 | 43 | 2.524 | 0.000141728 | up |
| MELO3C014023.2 | 8 | 31 | 1.976 | 0.00113614 | up |
| MELO3C014026.2 | 85 | 0 | -8.97 | 1.426E-10 | down |
| MELO3C014027.2 | 195 | 392 | 1.005 | 2.14913E-10 | up |
| MELO3C014038.2 | 6 | 0 | -5.158 | 0.005340718 | down |
| MELO3C014040.2 | 210 | 0 | -10.267 | 3.39225E-16 | down |
| MELO3C014042.2 | 1 | 323 | 8.821 | 2.76589E-17 | up |
| MELO3C014045.2 | 6709 | 20608 | 1.619 | 1.43064E-17 | up |
| MELO3C014047.2 | 27 | 6 | -2.3 | 0.004844412 | down |
| MELO3C014051.2 | 148 | 0 | -9.761 | 2.09476E-15 | down |
| MELO3C014056.2 | 10 | 1639 | 7.253 | 1.72984E-116 | up |
| MELO3C014076.2 | 0 | 1123 | 12.474 | 6.86198E-26 | up |
| MELO3C014089.2 | 2832 | 224 | -3.665 | 1.65877E-174 | down |
| MELO3C014099.2 | 2175 | 4558 | 1.067 | 3.92986E-11 | up |
| MELO3C014105.2 | 504 | 46 | -3.457 | 7.84668E-46 | down |
| MELO3C014107.2 | 39 | 156 | 1.987 | 3.36407E-14 | up |
| MELO3C014121.2 | 1 | 88 | 5.945 | 1.19549E-12 | up |
| MELO3C014124.2 | 8051 | 2115 | -1.929 | 1.38434E-48 | down |
| MELO3C014128.2 | 309 | 20 | -4.002 | 1.30586E-44 | down |
| MELO3C014131.2 | 0 | 83 | 8.711 | 1.94876E-12 | up |
| MELO3C014132.2 | 474 | 180 | -1.393 | 1.09163E-11 | down |
| MELO3C014140.2 | 251 | 607 | 1.278 | 4.369E-22 | up |
| MELO3C014154.2 | 34 | 105 | 1.607 | 3.76702E-05 | up |
| MELO3C014161.2 | 23570 | 11641 | -1.018 | 6.45359E-52 | down |
| MELO3C014168.2 | 930 | 418 | -1.152 | 1.08475E-12 | down |
| MELO3C014178.2 | 971 | 165 | -2.558 | 8.62283E-53 | down |
| MELO3C014190.2 | 131 | 56 | -1.212 | 0.000134503 | down |
| MELO3C014199.2 | 0 | 189 | 9.901 | 2.01631E-16 | up |
| MELO3C014208.2 | 79 | 699 | 3.132 | 1.93675E-49 | up |
| MELO3C014209.2 | 1 | 9 | 3.624 | 0.026684409 | up |
| MELO3C014218.2 | 1 | 10 | 3.811 | 0.009443684 | up |
| MELO3C014224.2 | 546 | 27 | -4.314 | 1.78241E-33 | down |
| MELO3C014227.2 | 32 | 5 | -2.611 | 0.00012702 | down |
| MELO3C014228.2 | 555 | 2977 | 2.422 | 5.91751E-06 | up |
| MELO3C014229.2 | 57 | 13 | -2.085 | 1.7235E-05 | down |
| MELO3C014234.2 | 1 | 10 | 3.2 | 0.014924396 | up |
| MELO3C014238.2 | 786 | 4043 | 2.364 | 5.69798E-35 | up |
| MELO3C014240.2 | 189 | 24 | -2.956 | 0.007144297 | down |
| MELO3C014247.2 | 28 | 3 | -3.516 | 0.00043136 | down |
| MELO3C014256.2 | 824 | 3080 | 1.902 | 4.73795E-21 | up |
| MELO3C014257.2 | 61 | 633 | 3.369 | 1.20486E-41 | up |
| MELO3C014260.2 | 611 | 2137 | 1.806 | 3.24556E-79 | up |
| MELO3C014279.2 | 7 | 32 | 2.186 | 0.002161514 | up |
| MELO3C014281.2 | 13 | 1 | -3.418 | 0.003151405 | down |
| MELO3C014282.2 | 2994 | 1322 | -1.18 | 4.92E-15 | down |
| MELO3C014288.2 | 52 | 210 | 2.011 | 1.74367E-13 | up |
| MELO3C014289.2 | 3941 | 474 | -3.056 | 3.07838E-41 | down |
| MELO3C014293.2 | 657 | 2211 | 1.75 | 2.50273E-35 | up |
| MELO3C014294.2 | 1453 | 173 | -3.064 | 2.19921E-06 | down |
| MELO3C014299.2 | 1748 | 475 | -1.879 | 0.00029392 | down |
| MELO3C014305.2 | 307 | 10 | -4.92 | 7.89907E-48 | down |
| MELO3C014309.2 | 830 | 390 | -1.089 | 2.86564E-19 | down |
| MELO3C014314.2 | 0 | 93 | 8.879 | 2.74443E-12 | up |
| MELO3C014317.2 | 27 | 669 | 4.638 | 2.29241E-36 | up |
| MELO3C014318.2 | 545 | 207 | -1.399 | 6.68959E-20 | down |
| MELO3C014319.2 | 36 | 110 | 1.606 | 1.21181E-05 | up |
| MELO3C014321.2 | 20187 | 7609 | -1.408 | 2.38977E-33 | down |
| MELO3C014324.2 | 86 | 537 | 2.635 | 2.36797E-18 | up |
| MELO3C014337.2 | 852 | 38 | -4.49 | 7.98108E-59 | down |
| MELO3C014347.2 | 47 | 142 | 1.597 | 6.49545E-07 | up |
| MELO3C014353.2 | 202 | 664 | 1.714 | 8.51714E-26 | up |
| MELO3C014391.2 | 2283 | 628 | -1.863 | 7.81483E-40 | down |
| MELO3C014392.2 | 40 | 157 | 1.969 | 5.2249E-14 | up |
| MELO3C014393.2 | 690 | 215 | -1.683 | 2.65287E-16 | down |
| MELO3C014398.2 | 11 | 43 | 2.002 | 0.000186747 | up |
| MELO3C014400.2 | 1048 | 411 | -1.35 | 4.33911E-35 | down |
| MELO3C014401.2 | 2234 | 640 | -1.805 | 5.40955E-33 | down |
| MELO3C014405.2 | 0 | 6 | 4.106 | 0.015541235 | up |
| MELO3C014408.2 | 177 | 79 | -1.175 | 5.21707E-06 | down |
| MELO3C014409.2 | 82 | 169 | 1.033 | 5.61424E-05 | up |
| MELO3C014412.2 | 64 | 20 | -1.7 | 0.000484542 | down |
| MELO3C014420.2 | 6946 | 2674 | -1.377 | 1.48615E-36 | down |
| MELO3C014425.2 | 2 | 19 | 3.126 | 0.015990495 | up |
| MELO3C014427.2 | 413 | 184 | -1.173 | 1.87278E-07 | down |
| MELO3C014430.2 | 69 | 4286 | 5.956 | 1.24574E-228 | up |
| MELO3C014432.2 | 223 | 953 | 2.098 | 5.22687E-23 | up |
| MELO3C014433.2 | 67 | 28 | -1.255 | 0.000279093 | down |
| MELO3C014436.2 | 3286 | 765 | -2.104 | 3.89844E-86 | down |
| MELO3C014437.2 | 3927 | 148 | -4.726 | 1.36376E-06 | down |
| MELO3C014441.2 | 4074 | 1597 | -1.352 | 2.61024E-42 | down |
| MELO3C014443.2 | 442 | 990 | 1.161 | 1.2027E-08 | up |
| MELO3C014444.2 | 0 | 14 | 6.189 | 2.61874E-05 | up |
| MELO3C014445.2 | 27 | 98 | 1.861 | 1.63469E-06 | up |
| MELO3C014447.2 | 978 | 2100 | 1.101 | 2.76645E-21 | up |
| MELO3C014448.2 | 137 | 502 | 1.875 | 2.24665E-23 | up |
| MELO3C014462.2 | 15 | 63 | 2.096 | 5.52775E-07 | up |
| MELO3C014463.2 | 0 | 9 | 5.482 | 0.001015912 | up |
| MELO3C014475.2 | 46 | 833 | 4.18 | 8.09439E-38 | up |
| MELO3C014476.2 | 278 | 896 | 1.69 | 1.99919E-38 | up |
| MELO3C014478.2 | 21 | 212 | 3.353 | 1.62797E-19 | up |
| MELO3C014481.2 | 3687 | 249 | -3.889 | 1.45896E-62 | down |
| MELO3C014486.2 | 1 | 19 | 4.712 | 0.000273275 | up |
| MELO3C014488.2 | 3109 | 6607 | 1.087 | 8.90257E-13 | up |
| MELO3C014489.2 | 140 | 405 | 1.533 | 9.15546E-10 | up |
| MELO3C014498.2 | 106 | 467 | 2.146 | 4.93285E-24 | up |
| MELO3C014507.2 | 634 | 235 | -1.427 | 4.70262E-18 | down |
| MELO3C014508.2 | 13 | 3 | -2.087 | 0.018072777 | down |
| MELO3C014511.2 | 8 | 2590 | 8.362 | 4.67906E-120 | up |
| MELO3C014513.2 | 3353 | 1516 | -1.145 | 1.77823E-15 | down |
| MELO3C014514.2 | 419 | 856 | 1.034 | 1.39333E-16 | up |
| MELO3C014523.2 | 19 | 62 | 1.746 | 0.000561841 | up |
| MELO3C014527.2 | 5423 | 12382 | 1.191 | 3.45036E-53 | up |
| MELO3C014530.2 | 337 | 114 | -1.563 | 8.62737E-15 | down |
| MELO3C014535.2 | 61 | 282 | 2.215 | 4.04054E-28 | up |
| MELO3C014551.2 | 7557 | 3423 | -1.142 | 2.03467E-21 | down |
| MELO3C014559.2 | 169 | 419 | 1.314 | 5.30119E-18 | up |
| MELO3C014564.2 | 352 | 1008 | 1.517 | 4.58744E-17 | up |
| MELO3C014568.2 | 355 | 9 | -5.241 | 3.94445E-25 | down |
| MELO3C014571.2 | 3122 | 1009 | -1.63 | 6.47986E-59 | down |
| MELO3C014579.2 | 54 | 377 | 2.81 | 1.17895E-36 | up |
| MELO3C014587.2 | 7285 | 462 | -3.979 | 3.12724E-81 | down |
| MELO3C014588.2 | 313 | 840 | 1.423 | 1.13801E-25 | up |
| MELO3C014589.2 | 440 | 39 | -3.506 | 3.69185E-57 | down |
| MELO3C014591.2 | 14 | 4 | -1.898 | 0.026954602 | down |
| MELO3C014596.2 | 5405 | 2030 | -1.413 | 7.15112E-29 | down |
| MELO3C014599.2 | 1316 | 464 | -1.505 | 5.40695E-26 | down |
| MELO3C014602.2 | 21 | 54 | 1.367 | 0.003430403 | up |
| MELO3C014607.2 | 1404 | 654 | -1.104 | 2.28668E-24 | down |
| MELO3C014622.2 | 492 | 166 | -1.568 | 2.17612E-27 | down |
| MELO3C014624.2 | 88 | 30 | -1.564 | 7.81154E-06 | down |
| MELO3C014625.2 | 212 | 3 | -5.973 | 3.86527E-33 | down |
| MELO3C014626.2 | 29 | 8 | -1.957 | 0.00673824 | down |
| MELO3C014632.2 | 5 | 315 | 5.783 | 2.14783E-45 | up |
| MELO3C014638.2 | 38 | 116 | 1.604 | 0.0092243 | up |
| MELO3C014648.2 | 1310 | 280 | -2.227 | 3.17751E-27 | down |
| MELO3C014661.2 | 1984 | 662 | -1.585 | 2.68608E-27 | down |
| MELO3C014666.2 | 2619 | 194 | -3.756 | 2.08173E-21 | down |
| MELO3C014669.2 | 942 | 464 | -1.024 | 0.001008067 | down |
| MELO3C014688.2 | 356 | 139 | -1.357 | 6.25864E-09 | down |
| MELO3C014692.2 | 715 | 122 | -2.55 | 4.84002E-48 | down |
| MELO3C014696.2 | 56 | 9 | -2.546 | 4.75069E-08 | down |
| MELO3C014697.2 | 343 | 155 | -1.147 | 6.2878E-12 | down |
| MELO3C014698.2 | 548 | 1344 | 1.295 | 2.26483E-16 | up |
| MELO3C014701.2 | 255 | 47 | -2.435 | 2.74842E-21 | down |
| MELO3C014709.2 | 328 | 54 | -2.622 | 7.77508E-26 | down |
| MELO3C014712.2 | 122 | 271 | 1.144 | 7.73146E-06 | up |
| MELO3C014714.2 | 819 | 382 | -1.102 | 1.14312E-07 | down |
| MELO3C014717.2 | 153 | 345 | 1.168 | 3.1277E-09 | up |
| MELO3C014722.2 | 141 | 48 | -1.54 | 1.91244E-06 | down |
| MELO3C014724.2 | 161 | 80 | -1.009 | 0.002248974 | down |
| MELO3C014728.2 | 2495 | 1075 | -1.216 | 3.68408E-18 | down |
| MELO3C014752.2 | 22174 | 9434 | -1.233 | 4.55224E-21 | down |
| MELO3C014774.2 | 1111 | 3309 | 1.575 | 7.57277E-39 | up |
| MELO3C014795.2 | 615 | 1786 | 1.539 | 1.99773E-18 | up |
| MELO3C014803.2 | 74 | 246 | 1.73 | 8.4854E-13 | up |
| MELO3C014810.2 | 6 | 31 | 2.44 | 0.002530334 | up |
| MELO3C014812.2 | 55 | 209 | 1.933 | 2.455E-10 | up |
| MELO3C014815.2 | 70 | 17 | -2.046 | 3.79524E-07 | down |
| MELO3C014821.2 | 40 | 421 | 3.403 | 6.75535E-47 | up |
| MELO3C014826.2 | 1708 | 624 | -1.455 | 3.51875E-43 | down |
| MELO3C014827.2 | 34 | 1 | -5.802 | 7.90411E-07 | down |
| MELO3C014853.2 | 91 | 3 | -4.898 | 1.20179E-15 | down |
| MELO3C014857.2 | 27 | 67 | 1.293 | 0.011924288 | up |
| MELO3C014870.2 | 7469 | 2415 | -1.629 | 5.60665E-41 | down |
| MELO3C014872.2 | 26 | 9 | -1.56 | 0.007323825 | down |
| MELO3C014875.2 | 2604 | 634 | -2.039 | 1.05491E-33 | down |
| MELO3C014888.2 | 3798 | 1353 | -1.489 | 1.48321E-49 | down |
| MELO3C014890.2 | 323 | 72 | -2.157 | 3.51498E-09 | down |
| MELO3C014892.2 | 88 | 34 | -1.365 | 0.001780595 | down |
| MELO3C014896.2 | 581 | 83 | -2.801 | 1.55226E-58 | down |
| MELO3C014897.2 | 40953 | 15502 | -1.402 | 3.97908E-22 | down |
| MELO3C014905.2 | 3 | 27 | 3.224 | 2.3058E-05 | up |
| MELO3C014907.2 | 59 | 178 | 1.587 | 4.01058E-09 | up |
| MELO3C014909.2 | 193 | 6 | -5.037 | 7.56427E-30 | down |
| MELO3C014912.2 | 0 | 9 | 5.552 | 0.000569689 | up |
| MELO3C014935.2 | 70 | 246 | 1.802 | 1.25312E-13 | up |
| MELO3C014936.2 | 94 | 259 | 1.458 | 2.47631E-08 | up |
| MELO3C014944.2 | 31 | 1 | -4.643 | 2.20726E-06 | down |
| MELO3C014949.2 | 0 | 179 | 9.821 | 3.58672E-16 | up |
| MELO3C014965.2 | 44 | 7 | -2.631 | 1.15635E-05 | down |
| MELO3C014984.2 | 65 | 475 | 2.878 | 1.81847E-17 | up |
| MELO3C014986.2 | 3 | 335 | 6.701 | 3.90856E-37 | up |
| MELO3C014990.2 | 1 | 33 | 4.922 | 1.09865E-06 | up |
| MELO3C014991.2 | 14463 | 901 | -4.004 | 5.19329E-18 | down |
| MELO3C015002.2 | 5 | 190 | 5.35 | 3.80944E-27 | up |
| MELO3C015005.2 | 433 | 0 | -11.311 | 2.0256E-20 | down |
| MELO3C015011.2 | 3025 | 106 | -4.833 | 5.26E-122 | down |
| MELO3C015021.2 | 122 | 8 | -3.884 | 3.88494E-17 | down |
| MELO3C015029.2 | 0 | 7 | 5.183 | 0.002694107 | up |
| MELO3C015055.2 | 65 | 317 | 2.3 | 3.41092E-13 | up |
| MELO3C015067.2 | 914 | 449 | -1.027 | 2.3694E-05 | down |
| MELO3C015076.2 | 31107 | 1949 | -3.996 | 8.05516E-176 | down |
| MELO3C015079.2 | 117 | 289 | 1.314 | 2.18491E-08 | up |
| MELO3C015093.2 | 77 | 4554 | 5.881 | 3.27443E-263 | up |
| MELO3C015101.2 | 238 | 33 | -2.872 | 5.95685E-22 | down |
| MELO3C015118.2 | 637 | 67 | -3.244 | 3.36641E-36 | down |
| MELO3C015119.2 | 146 | 55 | -1.409 | 3.80874E-08 | down |
| MELO3C015123.2 | 11 | 40 | 1.856 | 0.001366235 | up |
| MELO3C015128.2 | 78 | 2 | -5.404 | 2.00907E-13 | down |
| MELO3C015129.2 | 23 | 5 | -2.297 | 0.001932969 | down |
| MELO3C015140.2 | 265 | 2 | -7.025 | 1.124E-07 | down |
| MELO3C015149.2 | 67 | 159 | 1.243 | 1.04903E-06 | up |
| MELO3C015151.2 | 11783 | 3351 | -1.815 | 1.5771E-80 | down |
| MELO3C015152.2 | 1275 | 326 | -1.968 | 1.62034E-33 | down |
| MELO3C015155.2 | 497 | 2096 | 2.076 | 1.63656E-88 | up |
| MELO3C015184.2 | 21 | 178 | 3.056 | 1.61778E-06 | up |
| MELO3C015185.2 | 167 | 2977 | 4.158 | 3.70727E-08 | up |
| MELO3C015189.2 | 5976 | 1 | -13.245 | 2.88342E-38 | down |
| MELO3C015193.2 | 2074 | 704 | -1.56 | 1.17535E-35 | down |
| MELO3C015208.2 | 38 | 99 | 1.391 | 1.73497E-05 | up |
| MELO3C015209.2 | 12 | 87 | 2.917 | 5.83599E-12 | up |
| MELO3C015216.2 | 27 | 145 | 2.434 | 1.70138E-11 | up |
| MELO3C015228.2 | 85 | 549 | 2.694 | 5.12372E-51 | up |
| MELO3C015230.2 | 115 | 1997 | 4.122 | 1.56956E-54 | up |
| MELO3C015231.2 | 15480 | 6231 | -1.313 | 5.18261E-25 | down |
| MELO3C015243.2 | 67 | 500 | 2.904 | 3.37256E-24 | up |
| MELO3C015253.2 | 330 | 42 | -2.966 | 4.18533E-35 | down |
| MELO3C015255.2 | 5510 | 2570 | -1.101 | 9.40477E-19 | down |
| MELO3C015257.2 | 246 | 29 | -3.084 | 2.90301E-15 | down |
| MELO3C015259.2 | 1 | 80 | 6.807 | 5.98066E-10 | up |
| MELO3C015260.2 | 471 | 1196 | 1.344 | 1.15625E-26 | up |
| MELO3C015261.2 | 0 | 150 | 9.567 | 3.47943E-15 | up |
| MELO3C015263.2 | 407 | 873 | 1.102 | 3.37952E-22 | up |
| MELO3C015277.2 | 464 | 143 | -1.699 | 1.38621E-13 | down |
| MELO3C015282.2 | 184 | 83 | -1.146 | 5.48555E-06 | down |
| MELO3C015287.2 | 156 | 335 | 1.098 | 1.44431E-08 | up |
| MELO3C015291.2 | 211 | 707 | 1.74 | 1.59675E-39 | up |
| MELO3C015294.2 | 5566 | 1729 | -1.687 | 1.8902E-42 | down |
| MELO3C015297.2 | 165 | 75 | -1.125 | 0.000239791 | down |
| MELO3C015300.2 | 67 | 3 | -4.773 | 1.60076E-12 | down |
| MELO3C015306.2 | 1918 | 55 | -5.113 | 1.38483E-18 | down |
| MELO3C015312.2 | 15 | 43 | 1.522 | 0.002310938 | up |
| MELO3C015313.2 | 23 | 192 | 3.041 | 4.02007E-24 | up |
| MELO3C015329.2 | 46 | 22 | -1.119 | 0.013485544 | down |
| MELO3C015330.2 | 9 | 0 | -4.694 | 0.002986406 | down |
| MELO3C015331.2 | 123 | 51 | -1.281 | 1.05879E-06 | down |
| MELO3C015337.2 | 14969 | 42971 | 1.521 | 1.72192E-59 | up |
| MELO3C015342.2 | 602 | 1219 | 1.017 | 1.24638E-25 | up |
| MELO3C015351.2 | 681 | 335 | -1.022 | 1.37757E-11 | down |
| MELO3C015353.2 | 307 | 73 | -2.084 | 3.96775E-25 | down |
| MELO3C015354.2 | 18 | 126 | 2.787 | 2.12936E-13 | up |
| MELO3C015357.2 | 1141 | 556 | -1.038 | 1.72656E-20 | down |
| MELO3C015360.2 | 3138 | 1462 | -1.102 | 1.09622E-45 | down |
| MELO3C015374.2 | 4636 | 9735 | 1.07 | 1.18858E-31 | up |
| MELO3C015378.2 | 1902 | 796 | -1.257 | 7.24801E-27 | down |
| MELO3C015382.2 | 126 | 56 | -1.156 | 3.28932E-05 | down |
| MELO3C015387.2 | 72 | 767 | 3.413 | 6.35016E-57 | up |
| MELO3C015396.2 | 2662 | 809 | -1.719 | 1.38967E-32 | down |
| MELO3C015398.2 | 1357 | 236 | -2.522 | 1.24312E-61 | down |
| MELO3C015399.2 | 6958 | 3027 | -1.201 | 2.87823E-33 | down |
| MELO3C015415.2 | 319 | 139 | -1.198 | 2.19327E-09 | down |
| MELO3C015418.2 | 1 | 171 | 6.905 | 3.48437E-19 | up |
| MELO3C015419.2 | 0 | 7 | 5.141 | 0.004038636 | up |
| MELO3C015421.2 | 7085 | 1790 | -1.984 | 7.99547E-121 | down |
| MELO3C015423.2 | 645 | 26 | -4.66 | 5.33877E-103 | down |
| MELO3C015427.2 | 35 | 4 | -3.257 | 1.59229E-06 | down |
| MELO3C015430.2 | 41 | 5 | -3.146 | 1.84538E-07 | down |
| MELO3C015434.2 | 12 | 30 | 1.323 | 0.020248931 | up |
| MELO3C015446.2 | 4253 | 1561 | -1.446 | 1.29564E-26 | down |
| MELO3C015450.2 | 897 | 1986 | 1.148 | 1.58594E-29 | up |
| MELO3C015469.2 | 5682 | 476 | -3.575 | 0 | down |
| MELO3C015470.2 | 16293 | 193 | -6.397 | 1.21657E-249 | down |
| MELO3C015475.2 | 206 | 618 | 1.586 | 3.40444E-13 | up |
| MELO3C015480.2 | 158 | 1012 | 2.678 | 9.81572E-34 | up |
| MELO3C015494.2 | 4237 | 1789 | -1.244 | 7.51778E-20 | down |
| MELO3C015495.2 | 14 | 42 | 1.624 | 0.0003588 | up |
| MELO3C015506.2 | 260 | 47 | -2.48 | 3.30471E-27 | down |
| MELO3C015507.2 | 1 | 14 | 4.303 | 0.00126562 | up |
| MELO3C015509.2 | 485 | 18 | -4.785 | 1.37464E-53 | down |
| MELO3C015511.2 | 14 | 37 | 1.407 | 0.011432321 | up |
| MELO3C015513.2 | 74 | 319 | 2.099 | 3.07174E-18 | up |
| MELO3C015514.2 | 36 | 122 | 1.763 | 0.000577041 | up |
| MELO3C015515.2 | 2 | 20 | 3.209 | 0.001277503 | up |
| MELO3C015519.2 | 314 | 156 | -1.003 | 1.17218E-09 | down |
| MELO3C015526.2 | 137 | 2963 | 4.437 | 2.09452E-28 | up |
| MELO3C015527.2 | 145 | 668 | 2.209 | 4.82473E-18 | up |
| MELO3C015533.2 | 38 | 3 | -3.613 | 7.05877E-08 | down |
| MELO3C015536.2 | 2520 | 240 | -3.393 | 3.4574E-84 | down |
| MELO3C015538.2 | 727 | 107 | -2.765 | 1.35453E-05 | down |
| MELO3C015544.2 | 16673 | 6616 | -1.334 | 2.91933E-32 | down |
| MELO3C015549.2 | 42 | 1 | -5.501 | 3.07866E-08 | down |
| MELO3C015551.2 | 0 | 9 | 5.523 | 0.000770732 | up |
| MELO3C015552.2 | 4603 | 1040 | -2.146 | 3.63557E-46 | down |
| MELO3C015556.2 | 3584 | 1399 | -1.358 | 2.14907E-30 | down |
| MELO3C015567.2 | 1577 | 399 | -1.981 | 1.02903E-66 | down |
| MELO3C015568.2 | 28126 | 11597 | -1.278 | 1.74502E-42 | down |
| MELO3C015569.2 | 9 | 32 | 1.796 | 0.003498346 | up |
| MELO3C015570.2 | 33 | 353 | 3.425 | 1.66698E-44 | up |
| MELO3C015575.2 | 397 | 1358 | 1.777 | 7.47249E-32 | up |
| MELO3C015577.2 | 2698 | 5622 | 1.059 | 2.12307E-17 | up |
| MELO3C015587.2 | 16 | 2 | -2.894 | 0.002877122 | down |
| MELO3C015590.2 | 70 | 248 | 1.825 | 6.03091E-18 | up |
| MELO3C015593.2 | 436 | 5396 | 3.629 | 1.42861E-153 | up |
| MELO3C015598.2 | 180 | 370 | 1.038 | 1.86605E-10 | up |
| MELO3C015618.2 | 78 | 196 | 1.324 | 2.0862E-07 | up |
| MELO3C015622.2 | 139 | 383 | 1.466 | 1.90756E-10 | up |
| MELO3C015630.2 | 92 | 27 | -1.756 | 0.000161989 | down |
| MELO3C015640.2 | 7 | 55 | 2.948 | 2.54223E-06 | up |
| MELO3C015648.2 | 1627 | 772 | -1.077 | 1.34844E-16 | down |
| MELO3C015651.2 | 21 | 3 | -2.925 | 0.000347272 | down |
| MELO3C015653.2 | 7119 | 3452 | -1.044 | 1.13986E-30 | down |
| MELO3C015661.2 | 2743 | 7665 | 1.482 | 8.55226E-24 | up |
| MELO3C015677.2 | 369 | 134 | -1.466 | 1.54762E-15 | down |
| MELO3C015678.2 | 677 | 213 | -1.673 | 2.66691E-17 | down |
| MELO3C015686.2 | 233 | 501 | 1.103 | 1.22407E-07 | up |
| MELO3C015687.2 | 6 | 0 | -5.16 | 0.004849017 | down |
| MELO3C015689.2 | 0 | 7 | 5.114 | 0.004650385 | up |
| MELO3C015706.2 | 3869 | 1886 | -1.036 | 1.21912E-31 | down |
| MELO3C015711.2 | 382 | 775 | 1.017 | 6.7214E-08 | up |
| MELO3C015718.2 | 2413 | 1134 | -1.09 | 1.00593E-29 | down |
| MELO3C015722.2 | 2519 | 7283 | 1.531 | 6.3657E-87 | up |
| MELO3C015726.2 | 43 | 155 | 1.848 | 3.39393E-07 | up |
| MELO3C015727.2 | 163 | 388 | 1.252 | 2.27673E-14 | up |
| MELO3C015738.2 | 89 | 15 | -2.628 | 2.51343E-08 | down |
| MELO3C015740.2 | 226 | 495 | 1.131 | 5.64674E-09 | up |
| MELO3C015743.2 | 246 | 3785 | 3.944 | 2.74054E-67 | up |
| MELO3C015744.2 | 72856 | 3 | -14.528 | 1.96317E-204 | down |
| MELO3C015745.2 | 126 | 0 | -9.532 | 4.0836E-13 | down |
| MELO3C015746.2 | 0 | 11 | 4.875 | 0.00250177 | up |
| MELO3C015762.2 | 28 | 5 | -2.385 | 0.01794487 | down |
| MELO3C015764.2 | 28 | 180 | 2.687 | 6.40586E-21 | up |
| MELO3C015768.2 | 18 | 42 | 1.195 | 0.009612321 | up |
| MELO3C015772.2 | 14598 | 1210 | -3.593 | 5.02813E-115 | down |
| MELO3C015789.2 | 4051 | 574 | -2.818 | 4.52891E-108 | down |
| MELO3C015791.2 | 188 | 79 | -1.254 | 8.07625E-08 | down |
| MELO3C015792.2 | 856 | 56 | -3.948 | 1.71445E-60 | down |
| MELO3C015795.2 | 1532 | 265 | -2.531 | 3.03231E-68 | down |
| MELO3C015796.2 | 2081 | 517 | -2.01 | 1.45614E-55 | down |
| MELO3C015799.2 | 298 | 928 | 1.64 | 6.603E-35 | up |
| MELO3C015801.2 | 520 | 1367 | 1.392 | 3.99842E-33 | up |
| MELO3C015804.2 | 2513 | 1161 | -1.115 | 1.33347E-13 | down |
| MELO3C015806.2 | 140 | 50 | -1.468 | 5.22714E-07 | down |
| MELO3C015811.2 | 1792 | 780 | -1.201 | 2.83877E-28 | down |
| MELO3C015813.2 | 14 | 71 | 2.35 | 1.94361E-08 | up |
| MELO3C015818.2 | 0 | 9 | 5.488 | 0.000938199 | up |
| MELO3C015828.2 | 740 | 1566 | 1.081 | 8.76119E-21 | up |
| MELO3C015831.2 | 14 | 57 | 1.977 | 0.000926665 | up |
| MELO3C015834.2 | 1 | 15 | 3.786 | 0.001331645 | up |
| MELO3C015848.2 | 32 | 3 | -3.261 | 2.69031E-05 | down |
| MELO3C015850.2 | 623 | 182 | -1.778 | 2.66755E-39 | down |
| MELO3C015863.2 | 8 | 0 | -5.591 | 0.001908504 | down |
| MELO3C015865.2 | 0 | 20 | 6.664 | 4.03897E-06 | up |
| MELO3C015868.2 | 1775 | 314 | -2.496 | 4.524E-27 | down |
| MELO3C015872.2 | 1543 | 692 | -1.156 | 7.20777E-15 | down |
| MELO3C015875.2 | 170 | 376 | 1.152 | 4.43245E-10 | up |
| MELO3C015881.2 | 2 | 17 | 2.97 | 0.001435539 | up |
| MELO3C015882.2 | 7 | 359 | 5.574 | 8.71223E-36 | up |
| MELO3C015908.2 | 9 | 1 | -3.927 | 0.014803204 | down |
| MELO3C015909.2 | 650 | 2533 | 1.962 | 5.27504E-108 | up |
| MELO3C015910.2 | 182 | 39 | -2.231 | 4.83536E-15 | down |
| MELO3C015921.2 | 63 | 26 | -1.268 | 0.000336287 | down |
| MELO3C015924.2 | 13975 | 6551 | -1.093 | 5.23073E-38 | down |
| MELO3C015926.2 | 43 | 414 | 3.257 | 3.54486E-30 | up |
| MELO3C015930.2 | 76 | 18 | -2.099 | 0.001059015 | down |
| MELO3C015932.2 | 0 | 7 | 5.234 | 0.002104632 | up |
| MELO3C015934.2 | 23 | 118 | 2.369 | 3.76674E-14 | up |
| MELO3C015942.2 | 2 | 15 | 2.813 | 0.021082302 | up |
| MELO3C015947.2 | 174 | 74 | -1.234 | 1.30099E-06 | down |
| MELO3C015949.2 | 232 | 618 | 1.416 | 3.45221E-15 | up |
| MELO3C015957.2 | 1171 | 582 | -1.01 | 2.12418E-20 | down |
| MELO3C015963.2 | 0 | 8 | 5.297 | 0.00454854 | up |
| MELO3C015965.2 | 163 | 29 | -2.479 | 1.46819E-13 | down |
| MELO3C015984.2 | 209 | 77 | -1.437 | 3.97621E-11 | down |
| MELO3C015995.2 | 29220 | 6282 | -2.218 | 3.32852E-33 | down |
| MELO3C015998.2 | 3117 | 1420 | -1.133 | 3.72262E-29 | down |
| MELO3C016006.2 | 50 | 191 | 1.928 | 1.56593E-05 | up |
| MELO3C016007.2 | 6729 | 891 | -2.915 | 2.15235E-169 | down |
| MELO3C016008.2 | 132 | 332 | 1.34 | 8.14216E-09 | up |
| MELO3C016017.2 | 267 | 36 | -2.878 | 1.06129E-26 | down |
| MELO3C016022.2 | 0 | 24 | 6.914 | 4.13991E-07 | up |
| MELO3C016031.2 | 2110 | 298 | -2.825 | 2.45503E-11 | down |
| MELO3C016032.2 | 50 | 116 | 1.227 | 0.000106569 | up |
| MELO3C016033.2 | 1084 | 453 | -1.257 | 5.97838E-11 | down |
| MELO3C016049.2 | 1546 | 481 | -1.686 | 2.86263E-46 | down |
| MELO3C016051.2 | 5 | 16 | 1.685 | 0.02041894 | up |
| MELO3C016055.2 | 178 | 2 | -6.382 | 2.48816E-20 | down |
| MELO3C016080.2 | 26 | 99 | 1.918 | 2.83585E-09 | up |
| MELO3C016082.2 | 486 | 1319 | 1.44 | 3.21143E-08 | up |
| MELO3C016099.2 | 30 | 62 | 1.055 | 0.023645844 | up |
| MELO3C016109.2 | 16 | 2 | -2.944 | 0.0010417 | down |
| MELO3C016110.2 | 12 | 2 | -2.697 | 0.012457162 | down |
| MELO3C016114.2 | 121 | 342 | 1.499 | 3.92086E-15 | up |
| MELO3C016116.2 | 29 | 64 | 1.142 | 0.001873899 | up |
| MELO3C016119.2 | 801 | 1808 | 1.175 | 2.83051E-33 | up |
| MELO3C016131.2 | 3087 | 1357 | -1.186 | 8.11212E-47 | down |
| MELO3C016139.2 | 3858 | 1846 | -1.064 | 1.42385E-22 | down |
| MELO3C016140.2 | 5003 | 2227 | -1.168 | 1.25704E-16 | down |
| MELO3C016141.2 | 5 | 55 | 3.373 | 1.65209E-07 | up |
| MELO3C016164.2 | 449 | 1386 | 1.626 | 5.09643E-44 | up |
| MELO3C016167.2 | 27655 | 840 | -5.041 | 0 | down |
| MELO3C016168.2 | 29 | 222 | 2.92 | 6.23898E-25 | up |
| MELO3C016172.2 | 37 | 210 | 2.506 | 1.51971E-15 | up |
| MELO3C016176.2 | 220 | 14 | -3.927 | 9.86284E-39 | down |
| MELO3C016177.2 | 1799 | 544 | -1.727 | 1.81021E-46 | down |
| MELO3C016181.2 | 24828 | 4670 | -2.41 | 1.85904E-64 | down |
| MELO3C016188.2 | 4 | 17 | 1.846 | 0.017964646 | up |
| MELO3C016190.2 | 26953 | 3985 | -2.758 | 1.09984E-77 | down |
| MELO3C016197.2 | 40 | 173 | 2.107 | 5.32468E-13 | up |
| MELO3C016208.2 | 110 | 17 | -2.644 | 1.1417E-06 | down |
| MELO3C016210.2 | 947 | 223 | -2.082 | 1.30883E-30 | down |
| MELO3C016214.2 | 1 | 8 | 3.47 | 0.022022625 | up |
| MELO3C016218.2 | 161 | 395 | 1.298 | 1.35438E-10 | up |
| MELO3C016221.2 | 708 | 316 | -1.163 | 0.021431961 | down |
| MELO3C016224.2 | 63566 | 16574 | -1.939 | 7.26339E-93 | down |
| MELO3C016226.2 | 5999 | 2377 | -1.336 | 3.96494E-58 | down |
| MELO3C016232.2 | 9351 | 3873 | -1.272 | 7.8281E-20 | down |
| MELO3C016236.2 | 495 | 140 | -1.821 | 3.48251E-27 | down |
| MELO3C016252.2 | 1042 | 2160 | 1.052 | 1.29882E-41 | up |
| MELO3C016259.2 | 5662 | 188 | -4.906 | 1.48228E-153 | down |
| MELO3C016260.2 | 6122 | 2795 | -1.131 | 5.49216E-46 | down |
| MELO3C016263.2 | 52 | 9 | -2.518 | 1.12257E-06 | down |
| MELO3C016267.2 | 14 | 64 | 2.241 | 3.74798E-06 | up |
| MELO3C016268.2 | 52 | 6 | -3.014 | 8.3757E-06 | down |
| MELO3C016270.2 | 342 | 3 | -7.049 | 1.16699E-05 | down |
| MELO3C016271.2 | 119 | 8 | -3.858 | 1.34877E-14 | down |
| MELO3C016276.2 | 3269 | 1207 | -1.438 | 2.23256E-22 | down |
| MELO3C016287.2 | 79 | 2409 | 4.923 | 2.54749E-107 | up |
| MELO3C016288.2 | 473 | 1158 | 1.293 | 8.23564E-23 | up |
| MELO3C016290.2 | 6954 | 2918 | -1.253 | 2.02212E-19 | down |
| MELO3C016294.2 | 6 | 159 | 4.694 | 0.000269285 | up |
| MELO3C016296.2 | 6838 | 992 | -2.785 | 6.60704E-98 | down |
| MELO3C016297.2 | 1180 | 419 | -1.495 | 5.1003E-30 | down |
| MELO3C016300.2 | 7293 | 2769 | -1.397 | 1.35029E-19 | down |
| MELO3C016304.2 | 30 | 6 | -2.333 | 0.000226618 | down |
| MELO3C016308.2 | 7034 | 2805 | -1.327 | 4.4724E-65 | down |
| MELO3C016322.2 | 11 | 40 | 1.818 | 0.003706163 | up |
| MELO3C016331.2 | 2 | 128 | 5.893 | 8.23885E-19 | up |
| MELO3C016332.2 | 3 | 335 | 6.87 | 3.15569E-34 | up |
| MELO3C016334.2 | 1812 | 741 | -1.289 | 5.22465E-13 | down |
| MELO3C016340.2 | 264 | 5 | -5.837 | 1.62768E-09 | down |
| MELO3C016342.2 | 274 | 1195 | 2.125 | 1.10208E-57 | up |
| MELO3C016346.2 | 161 | 1843 | 3.519 | 1.3345E-49 | up |
| MELO3C016348.2 | 2586 | 5810 | 1.167 | 7.50605E-24 | up |
| MELO3C016350.2 | 816 | 1724 | 1.08 | 2.38507E-16 | up |
| MELO3C016351.2 | 4920 | 1112 | -2.146 | 3.69136E-148 | down |
| MELO3C016354.2 | 2643 | 384 | -2.779 | 1.10113E-151 | down |
| MELO3C016356.2 | 7 | 0 | -5.321 | 0.00273494 | down |
| MELO3C016358.2 | 11 | 1 | -4.212 | 0.004050216 | down |
| MELO3C016366.2 | 27411 | 7755 | -1.822 | 2.59318E-129 | down |
| MELO3C016368.2 | 2813 | 1307 | -1.105 | 3.82022E-34 | down |
| MELO3C016375.2 | 3951 | 1938 | -1.028 | 4.07478E-07 | down |
| MELO3C016383.2 | 1235 | 547 | -1.174 | 8.72455E-33 | down |
| MELO3C016384.2 | 283 | 14 | -4.359 | 1.81901E-47 | down |
| MELO3C016385.2 | 61 | 0 | -8.478 | 1.56869E-11 | down |
| MELO3C016396.2 | 62 | 16 | -1.927 | 4.07497E-05 | down |
| MELO3C016398.2 | 235 | 865 | 1.877 | 0.000147606 | up |
| MELO3C016402.2 | 84 | 1 | -7.065 | 1.14267E-08 | down |
| MELO3C016410.2 | 335 | 130 | -1.37 | 6.65222E-11 | down |
| MELO3C016420.2 | 923 | 457 | -1.014 | 1.15627E-23 | down |
| MELO3C016421.2 | 2463 | 5686 | 1.207 | 7.51742E-24 | up |
| MELO3C016434.2 | 130 | 526 | 2.023 | 1.12656E-09 | up |
| MELO3C016445.2 | 0 | 69 | 8.449 | 1.1875E-11 | up |
| MELO3C016448.2 | 386 | 783 | 1.02 | 2.48956E-19 | up |
| MELO3C016451.2 | 29 | 97 | 1.755 | 1.7454E-07 | up |
| MELO3C016459.2 | 125 | 922 | 2.889 | 7.19221E-105 | up |
| MELO3C016465.2 | 64 | 1 | -6.669 | 4.37808E-09 | down |
| MELO3C016476.2 | 12 | 61 | 2.334 | 5.5786E-06 | up |
| MELO3C016482.2 | 133 | 317 | 1.254 | 1.12714E-11 | up |
| MELO3C016491.2 | 604 | 1336 | 1.146 | 1.54953E-19 | up |
| MELO3C016494.2 | 2542 | 3 | -10.009 | 3.06888E-78 | down |
| MELO3C016502.2 | 357 | 946 | 1.407 | 1.19644E-28 | up |
| MELO3C016505.2 | 2732 | 1283 | -1.09 | 5.15359E-35 | down |
| MELO3C016513.2 | 17730 | 8159 | -1.12 | 1.21674E-12 | down |
| MELO3C016522.2 | 117 | 3 | -5.113 | 4.46306E-22 | down |
| MELO3C016525.2 | 1008 | 3435 | 1.769 | 5.90054E-95 | up |
| MELO3C016527.2 | 326 | 139 | -1.232 | 7.51672E-06 | down |
| MELO3C016536.2 | 5318 | 37 | -7.16 | 3.07353E-218 | down |
| MELO3C016539.2 | 207 | 0 | -10.244 | 2.05006E-17 | down |
| MELO3C016541.2 | 34925 | 16044 | -1.122 | 4.52565E-34 | down |
| MELO3C016552.2 | 59 | 15 | -1.965 | 2.44065E-06 | down |
| MELO3C016556.2 | 50157 | 680 | -6.205 | 6.05271E-196 | down |
| MELO3C016562.2 | 6018 | 2628 | -1.196 | 7.12987E-55 | down |
| MELO3C016566.2 | 392 | 163 | -1.265 | 1.04899E-16 | down |
| MELO3C016569.2 | 726 | 307 | -1.245 | 3.88454E-07 | down |
| MELO3C016579.2 | 203 | 517 | 1.346 | 7.42721E-23 | up |
| MELO3C016586.2 | 23 | 0 | -7.113 | 2.71569E-07 | down |
| MELO3C016588.2 | 41 | 1 | -5.486 | 6.84991E-08 | down |
| MELO3C016590.2 | 77 | 1 | -6.968 | 6.2094E-10 | down |
| MELO3C016593.2 | 1769 | 18 | -6.607 | 5.81342E-135 | down |
| MELO3C016595.2 | 15004 | 1 | -14.574 | 3.30579E-45 | down |
| MELO3C016602.2 | 327 | 148 | -1.141 | 4.11537E-08 | down |
| MELO3C016608.2 | 567 | 161 | -1.817 | 5.27237E-18 | down |
| MELO3C016619.2 | 111 | 35 | -1.657 | 0.002668652 | down |
| MELO3C016623.2 | 511 | 1323 | 1.375 | 2.47128E-18 | up |
| MELO3C016627.2 | 354 | 77 | -2.197 | 3.24292E-14 | down |
| MELO3C016628.2 | 237 | 10 | -4.578 | 9.33244E-23 | down |
| MELO3C016650.2 | 39 | 386 | 3.309 | 2.70995E-39 | up |
| MELO3C016656.2 | 832 | 351 | -1.246 | 4.96538E-21 | down |
| MELO3C016660.2 | 1370 | 5012 | 1.871 | 5.1217E-111 | up |
| MELO3C016661.2 | 167 | 25 | -2.757 | 4.94871E-23 | down |
| MELO3C016674.2 | 483 | 85 | -2.518 | 1.02365E-32 | down |
| MELO3C016676.2 | 3635 | 1330 | -1.451 | 2.63984E-16 | down |
| MELO3C016677.2 | 5 | 41 | 3.033 | 1.52913E-06 | up |
| MELO3C016683.2 | 52 | 12 | -2.151 | 5.34908E-05 | down |
| MELO3C016684.2 | 21 | 5 | -2.084 | 0.005621443 | down |
| MELO3C016686.2 | 264 | 1052 | 1.997 | 1.75524E-22 | up |
| MELO3C016695.2 | 140 | 63 | -1.144 | 0.000137774 | down |
| MELO3C016703.2 | 243 | 612 | 1.332 | 3.24971E-24 | up |
| MELO3C016704.2 | 90 | 12 | -2.87 | 7.17332E-14 | down |
| MELO3C016707.2 | 395 | 801 | 1.02 | 2.28215E-13 | up |
| MELO3C016709.2 | 16 | 47 | 1.553 | 0.000459882 | up |
| MELO3C016712.2 | 6309 | 471 | -3.747 | 5.50607E-211 | down |
| MELO3C016714.2 | 31292 | 400 | -6.29 | 5.50413E-246 | down |
| MELO3C016717.2 | 2008 | 6008 | 1.581 | 1.88224E-20 | up |
| MELO3C016718.2 | 3222 | 1205 | -1.418 | 1.04137E-72 | down |
| MELO3C016719.2 | 5402 | 239 | -4.495 | 1.32312E-225 | down |
| MELO3C016729.2 | 540 | 1185 | 1.135 | 1.63857E-13 | up |
| MELO3C016732.2 | 328 | 981 | 1.583 | 4.64619E-35 | up |
| MELO3C016733.2 | 2034 | 10714 | 2.397 | 1.70704E-65 | up |
| MELO3C016739.2 | 12 | 0 | -6.158 | 5.38717E-05 | down |
| MELO3C016745.2 | 2131 | 97 | -4.449 | 3.925E-156 | down |
| MELO3C016746.2 | 4598 | 822 | -2.482 | 2.38617E-36 | down |
| MELO3C016747.2 | 2043 | 967 | -1.079 | 2.63944E-08 | down |
| MELO3C016754.2 | 223 | 616 | 1.466 | 3.73359E-17 | up |
| MELO3C016759.2 | 33 | 177 | 2.412 | 2.49606E-12 | up |
| MELO3C016765.2 | 18 | 52 | 1.54 | 0.003737247 | up |
| MELO3C016768.2 | 1 | 26 | 4.157 | 6.6945E-05 | up |
| MELO3C016769.2 | 247 | 4 | -6.072 | 4.11878E-28 | down |
| MELO3C016770.2 | 11 | 2 | -2.581 | 0.018882416 | down |
| MELO3C016771.2 | 9049 | 2083 | -2.119 | 3.80395E-05 | down |
| MELO3C016772.2 | 30 | 8 | -1.916 | 0.003549412 | down |
| MELO3C016773.2 | 573 | 63 | -3.188 | 3.81042E-23 | down |
| MELO3C016777.2 | 1407 | 8126 | 2.529 | 6.04423E-54 | up |
| MELO3C016779.2 | 1005 | 352 | -1.512 | 3.23802E-27 | down |
| MELO3C016780.2 | 664 | 228 | -1.55 | 1.17949E-26 | down |
| MELO3C016783.2 | 9 | 30 | 1.722 | 0.002346064 | up |
| MELO3C016787.2 | 22 | 247 | 3.5 | 5.62068E-20 | up |
| MELO3C016798.2 | 3 | 23 | 2.84 | 0.003144463 | up |
| MELO3C016806.2 | 1 | 12 | 3.141 | 0.005914908 | up |
| MELO3C016807.2 | 79 | 22 | -1.823 | 0.000111328 | down |
| MELO3C016808.2 | 148 | 57 | -1.381 | 3.47476E-05 | down |
| MELO3C016809.2 | 2074 | 429 | -2.275 | 3.63669E-08 | down |
| MELO3C016821.2 | 248 | 100 | -1.309 | 9.59819E-11 | down |
| MELO3C016824.2 | 4 | 32 | 3.001 | 1.25927E-05 | up |
| MELO3C016826.2 | 5 | 19 | 1.958 | 0.02851203 | up |
| MELO3C016828.2 | 2880 | 400 | -2.852 | 2.89007E-50 | down |
| MELO3C016829.2 | 4453 | 609 | -2.87 | 5.50948E-06 | down |
| MELO3C016835.2 | 12 | 1 | -4.296 | 0.004175782 | down |
| MELO3C016836.2 | 96 | 1 | -7.282 | 5.4481E-10 | down |
| MELO3C016841.2 | 123 | 27 | -2.176 | 3.28754E-06 | down |
| MELO3C016842.2 | 544 | 1907 | 1.809 | 6.48285E-13 | up |
| MELO3C016848.2 | 47 | 835 | 4.156 | 2.24776E-76 | up |
| MELO3C016849.2 | 32 | 503 | 3.983 | 1.69761E-61 | up |
| MELO3C016850.2 | 176 | 2 | -6.839 | 4.55714E-23 | down |
| MELO3C016855.2 | 345 | 933 | 1.437 | 1.7247E-14 | up |
| MELO3C016857.2 | 9 | 1 | -2.895 | 0.021073716 | down |
| MELO3C016860.2 | 92 | 409 | 2.16 | 2.14463E-27 | up |
| MELO3C016864.2 | 80 | 13 | -2.61 | 1.12952E-09 | down |
| MELO3C016867.2 | 975 | 354 | -1.465 | 4.29205E-25 | down |
| MELO3C016871.2 | 850 | 288 | -1.563 | 3.46996E-31 | down |
| MELO3C016872.2 | 4 | 422 | 6.619 | 2.60215E-47 | up |
| MELO3C016878.2 | 2084 | 4317 | 1.05 | 2.2693E-23 | up |
| MELO3C016879.2 | 12 | 37 | 1.646 | 0.002559873 | up |
| MELO3C016881.2 | 359 | 899 | 1.328 | 2.22965E-26 | up |
| MELO3C016882.2 | 505 | 1413 | 1.484 | 8.66579E-29 | up |
| MELO3C016883.2 | 114 | 755 | 2.733 | 6.68904E-28 | up |
| MELO3C016892.2 | 2805 | 1323 | -1.085 | 6.05135E-34 | down |
| MELO3C016897.2 | 5829 | 1511 | -1.949 | 1.26491E-69 | down |
| MELO3C016901.2 | 188 | 32 | -2.536 | 3.93727E-17 | down |
| MELO3C016905.2 | 86 | 15 | -2.55 | 1.84059E-11 | down |
| MELO3C016910.2 | 896 | 319 | -1.492 | 1.72182E-12 | down |
| MELO3C016922.2 | 50 | 4 | -3.645 | 2.3577E-09 | down |
| MELO3C016926.2 | 36 | 112 | 1.643 | 3.28325E-08 | up |
| MELO3C016932.2 | 2815 | 210 | -3.745 | 4.65185E-62 | down |
| MELO3C016935.2 | 1105 | 535 | -1.047 | 6.15234E-16 | down |
| MELO3C016938.2 | 67 | 441 | 2.72 | 1.21401E-41 | up |
| MELO3C016941.2 | 10 | 2 | -2.726 | 0.018112152 | down |
| MELO3C016951.2 | 9004 | 3451 | -1.384 | 1.83043E-26 | down |
| MELO3C016956.2 | 2024 | 4265 | 1.075 | 5.27653E-32 | up |
| MELO3C016957.2 | 159 | 401 | 1.336 | 1.17675E-14 | up |
| MELO3C016962.2 | 190 | 19 | -3.275 | 9.80372E-28 | down |
| MELO3C016964.2 | 27 | 5 | -2.497 | 0.000122599 | down |
| MELO3C016966.2 | 578 | 212 | -1.445 | 7.94963E-21 | down |
| MELO3C016977.2 | 64 | 188 | 1.566 | 2.16876E-10 | up |
| MELO3C016978.2 | 988 | 355 | -1.478 | 1.83952E-25 | down |
| MELO3C016989.2 | 158 | 73 | -1.122 | 0.001151829 | down |
| MELO3C016999.2 | 1 | 9 | 3.723 | 0.011894853 | up |
| MELO3C017005.2 | 45 | 167 | 1.904 | 1.23597E-11 | up |
| MELO3C017009.2 | 468 | 1481 | 1.662 | 0.000198216 | up |
| MELO3C017011.2 | 24474 | 8345 | -1.552 | 7.06814E-24 | down |
| MELO3C017023.2 | 2074 | 260 | -2.996 | 1.27055E-65 | down |
| MELO3C017027.2 | 1139 | 3659 | 1.683 | 9.25586E-33 | up |
| MELO3C017032.2 | 65 | 144 | 1.151 | 4.78301E-06 | up |
| MELO3C017034.2 | 22 | 171 | 2.968 | 9.57246E-25 | up |
| MELO3C017044.2 | 31152 | 14947 | -1.06 | 6.81225E-17 | down |
| MELO3C017047.2 | 3015 | 883 | -1.772 | 1.21811E-87 | down |
| MELO3C017052.2 | 17 | 60 | 1.829 | 7.49314E-06 | up |
| MELO3C017053.2 | 0 | 8 | 4.426 | 0.005614338 | up |
| MELO3C017057.2 | 373 | 774 | 1.053 | 6.23135E-10 | up |
| MELO3C017059.2 | 11 | 69 | 2.739 | 0.00020623 | up |
| MELO3C017060.2 | 580 | 1161 | 1.001 | 3.26054E-07 | up |
| MELO3C017068.2 | 818 | 7271 | 3.152 | 6.48076E-158 | up |
| MELO3C017072.2 | 20 | 3 | -2.772 | 0.008055007 | down |
| MELO3C017087.2 | 4089 | 1886 | -1.117 | 3.20158E-29 | down |
| MELO3C017090.2 | 67 | 179 | 1.419 | 7.41188E-08 | up |
| MELO3C017100.2 | 4034 | 12264 | 1.604 | 5.65448E-36 | up |
| MELO3C017104.2 | 5011 | 1139 | -2.138 | 2.48168E-47 | down |
| MELO3C017109.2 | 4076 | 1394 | -1.548 | 2.63292E-21 | down |
| MELO3C017113.2 | 838 | 293 | -1.515 | 1.53592E-12 | down |
| MELO3C017116.2 | 2986 | 1131 | -1.4 | 3.04289E-12 | down |
| MELO3C017121.2 | 963 | 445 | -1.111 | 2.88776E-17 | down |
| MELO3C017124.2 | 70 | 11 | -2.598 | 2.34897E-06 | down |
| MELO3C017126.2 | 1555 | 3785 | 1.283 | 5.77782E-36 | up |
| MELO3C017130.2 | 608 | 232 | -1.388 | 1.17587E-21 | down |
| MELO3C017131.2 | 3454 | 1468 | -1.234 | 4.39484E-47 | down |
| MELO3C017132.2 | 88 | 194 | 1.15 | 0.000113836 | up |
| MELO3C017141.2 | 90 | 39 | -1.227 | 0.000181211 | down |
| MELO3C017146.2 | 108 | 25 | -2.117 | 1.28263E-06 | down |
| MELO3C017147.2 | 23 | 164 | 2.869 | 2.11173E-14 | up |
| MELO3C017151.2 | 83 | 168 | 1.014 | 0.015816562 | up |
| MELO3C017152.2 | 0 | 4 | 4.461 | 0.029785725 | up |
| MELO3C017154.2 | 583 | 36 | -3.98 | 1.69277E-25 | down |
| MELO3C017156.2 | 1312 | 523 | -1.329 | 1.66482E-21 | down |
| MELO3C017158.2 | 16 | 175 | 3.437 | 1.47718E-16 | up |
| MELO3C017161.2 | 0 | 20 | 6.673 | 8.03697E-05 | up |
| MELO3C017166.2 | 1690 | 833 | -1.02 | 1.15716E-19 | down |
| MELO3C017168.2 | 17 | 1 | -4.772 | 0.000349149 | down |
| MELO3C017170.2 | 0 | 4 | 4.474 | 0.025224498 | up |
| MELO3C017175.2 | 767 | 2185 | 1.511 | 1.6854E-72 | up |
| MELO3C017176.2 | 13130 | 5665 | -1.213 | 7.76214E-21 | down |
| MELO3C017180.2 | 36760 | 182 | -7.652 | 0 | down |
| MELO3C017182.2 | 569 | 223 | -1.354 | 1.67506E-11 | down |
| MELO3C017185.2 | 461 | 943 | 1.034 | 9.85406E-11 | up |
| MELO3C017197.2 | 351 | 1241 | 1.823 | 2.51406E-48 | up |
| MELO3C017198.2 | 51 | 138 | 1.421 | 1.63872E-08 | up |
| MELO3C017201.2 | 8 | 41 | 2.324 | 9.54532E-06 | up |
| MELO3C017206.2 | 638 | 2272 | 1.832 | 1.00972E-29 | up |
| MELO3C017210.2 | 162 | 31 | -2.396 | 9.29186E-08 | down |
| MELO3C017218.2 | 23 | 55 | 1.273 | 0.015424895 | up |
| MELO3C017219.2 | 1983 | 13210 | 2.736 | 1.04992E-16 | up |
| MELO3C017220.2 | 152 | 360 | 1.242 | 1.46119E-07 | up |
| MELO3C017228.2 | 10 | 283 | 4.767 | 1.47445E-30 | up |
| MELO3C017231.2 | 752 | 2338 | 1.636 | 1.22536E-51 | up |
| MELO3C017232.2 | 12 | 149 | 3.613 | 1.21014E-19 | up |
| MELO3C017242.2 | 1422 | 9539 | 2.746 | 3.45924E-79 | up |
| MELO3C017243.2 | 8 | 24 | 1.58 | 0.013714416 | up |
| MELO3C017244.2 | 2 | 51 | 4.575 | 1.81562E-09 | up |
| MELO3C017245.2 | 268 | 1176 | 2.135 | 3.77968E-20 | up |
| MELO3C017253.2 | 2792 | 5886 | 1.076 | 9.08226E-34 | up |
| MELO3C017254.2 | 1532 | 743 | -1.044 | 1.92336E-29 | down |
| MELO3C017256.2 | 263 | 1699 | 2.692 | 5.49159E-101 | up |
| MELO3C017258.2 | 53 | 186 | 1.802 | 5.09237E-11 | up |
| MELO3C017261.2 | 1756 | 534 | -1.718 | 3.39837E-18 | down |
| MELO3C017263.2 | 325 | 794 | 1.29 | 4.93107E-20 | up |
| MELO3C017267.2 | 1584 | 580 | -1.449 | 3.17568E-47 | down |
| MELO3C017272.2 | 0 | 119 | 8.277 | 1.04212E-11 | up |
| MELO3C017291.2 | 1655 | 3528 | 1.092 | 1.089E-21 | up |
| MELO3C017294.2 | 890 | 2 | -9.178 | 2.40095E-43 | down |
| MELO3C017298.2 | 557 | 1465 | 1.395 | 3.14882E-19 | up |
| MELO3C017299.2 | 22 | 98 | 2.164 | 1.38492E-08 | up |
| MELO3C017302.2 | 619 | 5331 | 3.107 | 4.68296E-120 | up |
| MELO3C017304.2 | 0 | 7 | 5.116 | 0.005488691 | up |
| MELO3C017314.2 | 89 | 3 | -4.614 | 2.96218E-14 | down |
| MELO3C017317.2 | 288 | 875 | 1.605 | 2.24252E-28 | up |
| MELO3C017318.2 | 380 | 906 | 1.254 | 5.40662E-19 | up |
| MELO3C017322.2 | 219 | 64 | -1.763 | 6.87438E-10 | down |
| MELO3C017328.2 | 0 | 18 | 5.575 | 8.68674E-05 | up |
| MELO3C017332.2 | 2999 | 11636 | 1.956 | 1.02779E-48 | up |
| MELO3C017333.2 | 181 | 81 | -1.143 | 1.11385E-06 | down |
| MELO3C017341.2 | 424 | 1110 | 1.389 | 7.08975E-10 | up |
| MELO3C017345.2 | 2 | 136 | 5.76 | 1.00523E-17 | up |
| MELO3C017348.2 | 206 | 91 | -1.176 | 7.78194E-07 | down |
| MELO3C017356.2 | 25 | 109 | 2.124 | 2.3695E-06 | up |
| MELO3C017357.2 | 32 | 272 | 3.098 | 4.13387E-13 | up |
| MELO3C017358.2 | 12463 | 5086 | -1.293 | 4.59118E-13 | down |
| MELO3C017374.2 | 1840 | 684 | -1.427 | 3.93253E-17 | down |
| MELO3C017385.2 | 438 | 81 | -2.432 | 1.80155E-27 | down |
| MELO3C017390.2 | 2960 | 1475 | -1.005 | 5.10596E-36 | down |
| MELO3C017391.2 | 121 | 528 | 2.126 | 8.90067E-32 | up |
| MELO3C017398.2 | 170 | 341 | 1.007 | 5.79125E-08 | up |
| MELO3C017410.2 | 134 | 410 | 1.617 | 1.89112E-14 | up |
| MELO3C017414.2 | 9 | 0 | -5.764 | 0.001248869 | down |
| MELO3C017415.2 | 510 | 116 | -2.129 | 3.05845E-37 | down |
| MELO3C017420.2 | 13 | 38 | 1.574 | 0.004997196 | up |
| MELO3C017424.2 | 348 | 112 | -1.638 | 7.95974E-10 | down |
| MELO3C017426.2 | 2320 | 916 | -1.341 | 3.01561E-17 | down |
| MELO3C017437.2 | 289 | 82 | -1.814 | 9.13049E-14 | down |
| MELO3C017439.2 | 1207 | 2738 | 1.182 | 2.4855E-43 | up |
| MELO3C017453.2 | 16 | 126 | 2.998 | 1.78572E-10 | up |
| MELO3C017481.2 | 28 | 1188 | 5.412 | 6.45168E-24 | up |
| MELO3C017482.2 | 12 | 36 | 1.577 | 0.006027378 | up |
| MELO3C017485.2 | 1487 | 442 | -1.751 | 2.02705E-49 | down |
| MELO3C017490.2 | 254 | 791 | 1.639 | 1.36314E-14 | up |
| MELO3C017499.2 | 0 | 30 | 7.237 | 4.51459E-08 | up |
| MELO3C017500.2 | 8624 | 3604 | -1.259 | 4.58782E-39 | down |
| MELO3C017503.2 | 2447 | 201 | -3.597 | 4.64042E-61 | down |
| MELO3C017507.2 | 32 | 5 | -2.691 | 5.85479E-05 | down |
| MELO3C017520.2 | 1395 | 31 | -5.478 | 6.85208E-145 | down |
| MELO3C017542.2 | 9034 | 4212 | -1.101 | 6.51683E-12 | down |
| MELO3C017554.2 | 71 | 10 | -2.825 | 1.04314E-08 | down |
| MELO3C017558.2 | 418 | 1075 | 1.362 | 3.49947E-24 | up |
| MELO3C017566.2 | 1682 | 7356 | 2.129 | 3.04737E-05 | up |
| MELO3C017568.2 | 160 | 38 | -2.071 | 8.09701E-13 | down |
| MELO3C017569.2 | 7 | 33 | 2.226 | 0.000163062 | up |
| MELO3C017571.2 | 3225 | 1333 | -1.274 | 3.98672E-23 | down |
| MELO3C017574.2 | 304 | 27 | -3.523 | 8.54236E-30 | down |
| MELO3C017579.2 | 396 | 924 | 1.222 | 1.06164E-29 | up |
| MELO3C017581.2 | 18045 | 1298 | -3.797 | 6.27172E-18 | down |
| MELO3C017588.2 | 215 | 32 | -2.745 | 7.14777E-11 | down |
| MELO3C017590.2 | 2202 | 719 | -1.615 | 2.19678E-34 | down |
| MELO3C017597.2 | 580 | 1601 | 1.464 | 1.74833E-23 | up |
| MELO3C017600.2 | 4812 | 2402 | -1.002 | 5.76197E-25 | down |
| MELO3C017603.2 | 994 | 5246 | 2.399 | 6.48091E-57 | up |
| MELO3C017606.2 | 391 | 801 | 1.035 | 5.88366E-17 | up |
| MELO3C017618.2 | 201 | 63 | -1.67 | 1.00799E-13 | down |
| MELO3C017620.2 | 20482 | 4032 | -2.345 | 8.76834E-143 | down |
| MELO3C017624.2 | 2901 | 1392 | -1.059 | 5.02837E-41 | down |
| MELO3C017630.2 | 80 | 879 | 3.464 | 1.928E-29 | up |
| MELO3C017632.2 | 5936 | 2483 | -1.257 | 1.76458E-37 | down |
| MELO3C017644.2 | 4 | 0 | -4.656 | 0.023712809 | down |
| MELO3C017651.2 | 31 | 75 | 1.31 | 0.000166229 | up |
| MELO3C017656.2 | 48 | 279 | 2.527 | 1.56073E-21 | up |
| MELO3C017662.2 | 9 | 40 | 2.09 | 0.001252379 | up |
| MELO3C017669.2 | 1207 | 595 | -1.021 | 5.76071E-21 | down |
| MELO3C017671.2 | 10 | 48 | 2.248 | 0.009229078 | up |
| MELO3C017674.2 | 7 | 106 | 3.951 | 0.003774952 | up |
| MELO3C017675.2 | 400 | 912 | 1.188 | 1.27314E-19 | up |
| MELO3C017677.2 | 4468 | 17030 | 1.93 | 2.11741E-210 | up |
| MELO3C017684.2 | 48 | 20 | -1.299 | 0.003684788 | down |
| MELO3C017687.2 | 24 | 87 | 1.867 | 2.25808E-05 | up |
| MELO3C017691.2 | 271 | 104 | -1.381 | 9.06003E-07 | down |
| MELO3C017701.2 | 140 | 318 | 1.18 | 4.0882E-09 | up |
| MELO3C017708.2 | 117 | 302 | 1.373 | 2.59772E-14 | up |
| MELO3C017721.2 | 1400 | 570 | -1.298 | 8.6292E-18 | down |
| MELO3C017723.2 | 120 | 60 | -1.005 | 0.000262416 | down |
| MELO3C017737.2 | 2998 | 1480 | -1.018 | 5.3728E-35 | down |
| MELO3C017740.2 | 852 | 1847 | 1.116 | 4.27398E-15 | up |
| MELO3C017743.2 | 339 | 51 | -2.733 | 9.34682E-15 | down |
| MELO3C017750.2 | 681 | 1383 | 1.023 | 3.57047E-16 | up |
| MELO3C017752.2 | 1275 | 483 | -1.397 | 3.49694E-22 | down |
| MELO3C017753.2 | 4743 | 1866 | -1.346 | 2.25012E-55 | down |
| MELO3C017754.2 | 15717 | 2643 | -2.573 | 1.65944E-94 | down |
| MELO3C017762.2 | 457 | 2385 | 2.384 | 3.50278E-99 | up |
| MELO3C017772.2 | 8514 | 2887 | -1.56 | 2.3817E-81 | down |
| MELO3C017776.2 | 170 | 35 | -2.308 | 1.28534E-12 | down |
| MELO3C017780.2 | 6 | 22 | 1.859 | 0.018500255 | up |
| MELO3C017782.2 | 432 | 3 | -7.137 | 2.83259E-48 | down |
| MELO3C017784.2 | 438 | 1047 | 1.257 | 9.62766E-26 | up |
| MELO3C017794.2 | 166 | 10 | -4.008 | 1.68811E-13 | down |
| MELO3C017795.2 | 443 | 191 | -1.213 | 1.3362E-15 | down |
| MELO3C017796.2 | 143 | 1016 | 2.828 | 3.73609E-41 | up |
| MELO3C017797.2 | 25 | 4 | -2.562 | 0.000855799 | down |
| MELO3C017801.2 | 3841 | 1902 | -1.014 | 6.84096E-34 | down |
| MELO3C017803.2 | 12 | 1 | -3.33 | 0.004117833 | down |
| MELO3C017807.2 | 17 | 73 | 2.093 | 7.93435E-06 | up |
| MELO3C017808.2 | 608 | 111 | -2.453 | 1.57557E-28 | down |
| MELO3C017812.2 | 318 | 968 | 1.608 | 9.51215E-39 | up |
| MELO3C017816.2 | 3521 | 709 | -2.313 | 2.09315E-48 | down |
| MELO3C017831.2 | 512 | 113 | -2.172 | 9.59707E-33 | down |
| MELO3C017834.2 | 598 | 219 | -1.447 | 3.084E-15 | down |
| MELO3C017836.2 | 77 | 319 | 2.053 | 4.84453E-17 | up |
| MELO3C017846.2 | 323 | 106 | -1.604 | 1.87144E-16 | down |
| MELO3C017847.2 | 3115 | 1116 | -1.482 | 2.18808E-29 | down |
| MELO3C017855.2 | 2290 | 863 | -1.408 | 1.61433E-29 | down |
| MELO3C017856.2 | 413 | 87 | -2.26 | 6.36132E-20 | down |
| MELO3C017857.2 | 40 | 0 | -6.929 | 7.03323E-08 | down |
| MELO3C017858.2 | 842 | 281 | -1.583 | 3.57404E-33 | down |
| MELO3C017861.2 | 1352 | 610 | -1.147 | 1.28814E-16 | down |
| MELO3C017864.2 | 2907 | 7308 | 1.33 | 4.64943E-21 | up |
| MELO3C017876.2 | 1149 | 35 | -5.031 | 8.21317E-24 | down |
| MELO3C017878.2 | 297 | 1391 | 2.226 | 1.27872E-24 | up |
| MELO3C017880.2 | 2114 | 596 | -1.828 | 1.67748E-57 | down |
| MELO3C017883.2 | 151 | 53 | -1.508 | 1.65568E-10 | down |
| MELO3C017886.2 | 734 | 347 | -1.081 | 1.14154E-08 | down |
| MELO3C017910.2 | 26 | 69 | 1.413 | 0.00011509 | up |
| MELO3C017915.2 | 4359 | 85 | -5.682 | 0 | down |
| MELO3C017917.2 | 7779 | 716 | -3.441 | 4.72591E-85 | down |
| MELO3C017920.2 | 397 | 112 | -1.821 | 1.91136E-25 | down |
| MELO3C017923.2 | 1 | 8 | 3.517 | 0.0247196 | up |
| MELO3C017929.2 | 20 | 44 | 1.146 | 0.009377861 | up |
| MELO3C017930.2 | 723 | 1831 | 1.339 | 2.57997E-48 | up |
| MELO3C017931.2 | 1149 | 135 | -3.089 | 2.00853E-107 | down |
| MELO3C017933.2 | 642 | 7 | -6.502 | 2.09463E-81 | down |
| MELO3C017935.2 | 72 | 5 | -3.833 | 2.20721E-09 | down |
| MELO3C017940.2 | 3194 | 464 | -2.782 | 1.72946E-79 | down |
| MELO3C017946.2 | 24 | 426 | 4.127 | 1.00018E-31 | up |
| MELO3C017947.2 | 5 | 0 | -5.066 | 0.006786812 | down |
| MELO3C017948.2 | 357 | 53 | -2.753 | 2.28363E-29 | down |
| MELO3C017953.2 | 1213 | 231 | -2.391 | 2.5051E-45 | down |
| MELO3C017954.2 | 8 | 0 | -4.634 | 0.003769837 | down |
| MELO3C017959.2 | 580 | 1165 | 1.006 | 1.82054E-13 | up |
| MELO3C017981.2 | 77 | 13 | -2.499 | 8.21417E-07 | down |
| MELO3C017989.2 | 426 | 1156 | 1.442 | 3.86271E-36 | up |
| MELO3C018004.2 | 19 | 2 | -3.39 | 0.002128143 | down |
| MELO3C018005.2 | 244 | 5478 | 4.491 | 0.000355826 | up |
| MELO3C018008.2 | 77 | 0 | -8.817 | 0.000105393 | down |
| MELO3C018009.2 | 5501 | 11 | -8.936 | 2.37289E-36 | down |
| MELO3C018017.2 | 4 | 26 | 2.683 | 0.028083385 | up |
| MELO3C018020.2 | 74 | 150 | 1.014 | 3.79289E-05 | up |
| MELO3C018023.2 | 1988 | 888 | -1.162 | 5.91772E-33 | down |
| MELO3C018025.2 | 12933 | 49091 | 1.924 | 1.55712E-129 | up |
| MELO3C018026.2 | 2685 | 1335 | -1.009 | 3.29086E-19 | down |
| MELO3C018048.2 | 83 | 323 | 1.957 | 5.6922E-13 | up |
| MELO3C018049.2 | 19 | 98 | 2.401 | 2.98686E-11 | up |
| MELO3C018058.2 | 163 | 508 | 1.641 | 5.38722E-28 | up |
| MELO3C018060.2 | 2140 | 4357 | 1.026 | 6.33765E-14 | up |
| MELO3C018066.2 | 2350 | 1128 | -1.058 | 4.10242E-24 | down |
| MELO3C018082.2 | 1678 | 4280 | 1.351 | 2.85846E-51 | up |
| MELO3C018088.2 | 30 | 12 | -1.291 | 0.017290586 | down |
| MELO3C018090.2 | 67 | 5 | -3.668 | 3.18311E-13 | down |
| MELO3C018106.2 | 266 | 610 | 1.2 | 5.18247E-13 | up |
| MELO3C018128.2 | 1612 | 3403 | 1.078 | 1.29949E-36 | up |
| MELO3C018132.2 | 78 | 436 | 2.484 | 1.96215E-32 | up |
| MELO3C018149.2 | 2681 | 437 | -2.618 | 3.70689E-132 | down |
| MELO3C018158.2 | 0 | 11 | 5.836 | 0.000281771 | up |
| MELO3C018161.2 | 840 | 1724 | 1.039 | 4.41867E-15 | up |
| MELO3C018185.2 | 525 | 4 | -6.933 | 6.81364E-49 | down |
| MELO3C018201.2 | 718 | 306 | -1.229 | 1.17856E-24 | down |
| MELO3C018202.2 | 20 | 2 | -3.021 | 0.000813377 | down |
| MELO3C018223.2 | 610 | 1254 | 1.04 | 1.00927E-22 | up |
| MELO3C018228.2 | 29 | 3 | -3.308 | 0.000131127 | down |
| MELO3C018242.2 | 679 | 11 | -5.898 | 2.3284E-18 | down |
| MELO3C018247.2 | 119 | 19 | -2.651 | 1.5238E-14 | down |
| MELO3C018248.2 | 6 | 145 | 4.497 | 3.1468E-20 | up |
| MELO3C018292.2 | 342 | 150 | -1.191 | 1.83334E-10 | down |
| MELO3C018303.2 | 74 | 26 | -1.479 | 0.001435618 | down |
| MELO3C018309.2 | 531 | 239 | -1.15 | 2.78025E-12 | down |
| MELO3C018311.2 | 19 | 0 | -6.8 | 1.5529E-06 | down |
| MELO3C018312.2 | 64 | 6 | -3.428 | 4.44012E-07 | down |
| MELO3C018317.2 | 60 | 20 | -1.586 | 0.007754466 | down |
| MELO3C018347.2 | 313 | 22 | -3.805 | 9.19744E-14 | down |
| MELO3C018349.2 | 206 | 31 | -2.699 | 1.40227E-10 | down |
| MELO3C018362.2 | 84 | 17 | -2.297 | 4.20391E-09 | down |
| MELO3C018364.2 | 4159 | 1189 | -1.807 | 3.09732E-70 | down |
| MELO3C018366.2 | 41 | 16 | -1.337 | 0.002287414 | down |
| MELO3C018372.2 | 2818 | 514 | -2.456 | 1.46963E-48 | down |
| MELO3C018374.2 | 472 | 984 | 1.061 | 4.60162E-16 | up |
| MELO3C018385.2 | 14 | 247 | 4.181 | 1.70418E-31 | up |
| MELO3C018386.2 | 114 | 466 | 2.037 | 1.85862E-33 | up |
| MELO3C018397.2 | 73 | 175 | 1.264 | 0.002463192 | up |
| MELO3C018409.2 | 3794 | 1217 | -1.641 | 2.82876E-35 | down |
| MELO3C018412.2 | 3196 | 3 | -10.173 | 1.32143E-93 | down |
| MELO3C018413.2 | 12649 | 2 | -12.326 | 1.84563E-125 | down |
| MELO3C018414.2 | 373 | 1096 | 1.555 | 3.16493E-40 | up |
| MELO3C018417.2 | 808 | 396 | -1.03 | 3.02854E-22 | down |
| MELO3C018419.2 | 4436 | 850 | -2.383 | 6.55778E-41 | down |
| MELO3C018421.2 | 902 | 1945 | 1.109 | 4.37603E-22 | up |
| MELO3C018422.2 | 61 | 307 | 2.326 | 5.16386E-24 | up |
| MELO3C018424.2 | 65 | 406 | 2.65 | 3.01831E-21 | up |
| MELO3C018434.2 | 369 | 744 | 1.015 | 9.98211E-17 | up |
| MELO3C018439.2 | 547 | 197 | -1.471 | 2.05028E-10 | down |
| MELO3C018442.2 | 151 | 41 | -1.867 | 7.22E-07 | down |
| MELO3C018445.2 | 2683 | 450 | -2.573 | 1.98589E-33 | down |
| MELO3C018448.2 | 916 | 295 | -1.633 | 4.60268E-46 | down |
| MELO3C018450.2 | 2561 | 355 | -2.85 | 6.9931E-104 | down |
| MELO3C018453.2 | 3813 | 1069 | -1.836 | 1.51357E-87 | down |
| MELO3C018454.2 | 77 | 293 | 1.928 | 1.74893E-17 | up |
| MELO3C018456.2 | 3730 | 1271 | -1.554 | 5.42015E-60 | down |
| MELO3C018463.2 | 22 | 3 | -2.78 | 0.019154321 | down |
| MELO3C018467.2 | 173 | 23 | -2.938 | 7.69108E-23 | down |
| MELO3C018468.2 | 40 | 6 | -2.749 | 9.54077E-06 | down |
| MELO3C018469.2 | 8 | 50 | 2.542 | 7.18605E-05 | up |
| MELO3C018470.2 | 4 | 4313 | 10.235 | 5.93835E-102 | up |
| MELO3C018482.2 | 2016 | 624 | -1.693 | 4.03652E-46 | down |
| MELO3C018484.2 | 116 | 50 | -1.201 | 6.69721E-06 | down |
| MELO3C018489.2 | 4186 | 894 | -2.228 | 7.48672E-69 | down |
| MELO3C018490.2 | 16240 | 2238 | -2.859 | 1.10331E-08 | down |
| MELO3C018492.2 | 1914 | 135 | -3.824 | 6.14758E-60 | down |
| MELO3C018495.2 | 76 | 773 | 3.343 | 2.71081E-81 | up |
| MELO3C018496.2 | 16 | 3 | -2.615 | 0.018733682 | down |
| MELO3C018503.2 | 25 | 1193 | 5.574 | 3.98467E-97 | up |
| MELO3C018505.2 | 2441 | 993 | -1.297 | 7.43749E-17 | down |
| MELO3C018508.2 | 550 | 266 | -1.047 | 1.29101E-13 | down |
| MELO3C018509.2 | 583 | 110 | -2.398 | 2.87061E-16 | down |
| MELO3C018522.2 | 0 | 62 | 8.305 | 5.89635E-11 | up |
| MELO3C018528.2 | 653 | 6 | -6.708 | 7.52798E-50 | down |
| MELO3C018532.2 | 33 | 694 | 4.398 | 5.0295E-62 | up |
| MELO3C018539.2 | 4 | 50 | 3.53 | 2.48386E-07 | up |
| MELO3C018551.2 | 26 | 93 | 1.85 | 5.146E-06 | up |
| MELO3C018552.2 | 1792 | 4357 | 1.282 | 4.33932E-20 | up |
| MELO3C018553.2 | 175 | 86 | -1.018 | 0.00164737 | down |
| MELO3C018569.2 | 3605 | 1448 | -1.316 | 8.41461E-32 | down |
| MELO3C018570.2 | 5245 | 2413 | -1.121 | 2.51776E-22 | down |
| MELO3C018571.2 | 291 | 71 | -2.032 | 1.8481E-24 | down |
| MELO3C018579.2 | 12542 | 960 | -3.708 | 1.22024E-26 | down |
| MELO3C018582.2 | 105 | 440 | 2.067 | 5.69793E-25 | up |
| MELO3C018593.2 | 631 | 2 | -8.413 | 9.08418E-42 | down |
| MELO3C018594.2 | 353 | 2 | -7.168 | 6.63625E-35 | down |
| MELO3C018598.2 | 33 | 2 | -4.114 | 0.003517725 | down |
| MELO3C018603.2 | 72 | 0 | -8.728 | 4.18422E-12 | down |
| MELO3C018607.2 | 3182 | 968 | -1.717 | 5.6446E-92 | down |
| MELO3C018618.2 | 172 | 38 | -2.169 | 2.77039E-09 | down |
| MELO3C018625.2 | 19 | 47 | 1.356 | 0.006088356 | up |
| MELO3C018627.2 | 414 | 78 | -2.395 | 5.54029E-24 | down |
| MELO3C018631.2 | 774 | 93 | -3.056 | 2.39418E-28 | down |
| MELO3C018632.2 | 218 | 9 | -4.578 | 1.85859E-33 | down |
| MELO3C018636.2 | 499 | 106 | -2.232 | 1.1193E-10 | down |
| MELO3C018643.2 | 1178 | 520 | -1.178 | 2.63427E-28 | down |
| MELO3C018645.2 | 0 | 24 | 6.921 | 5.98279E-07 | up |
| MELO3C018658.2 | 942 | 3037 | 1.689 | 1.72565E-22 | up |
| MELO3C018663.2 | 109 | 256 | 1.242 | 6.1937E-06 | up |
| MELO3C018664.2 | 999 | 14 | -6.145 | 1.73984E-116 | down |
| MELO3C018667.2 | 156 | 523 | 1.745 | 2.04386E-13 | up |
| MELO3C018669.2 | 1012 | 245 | -2.048 | 1.66323E-51 | down |
| MELO3C018677.2 | 416 | 204 | -1.025 | 1.41428E-07 | down |
| MELO3C018681.2 | 5483 | 741 | -2.887 | 7.46592E-05 | down |
| MELO3C018692.2 | 940 | 0 | -12.43 | 1.98109E-25 | down |
| MELO3C018698.2 | 818 | 241 | -1.761 | 2.79921E-27 | down |
| MELO3C018700.2 | 3228 | 248 | -3.699 | 3.83728E-17 | down |
| MELO3C018701.2 | 0 | 6 | 4.786 | 0.010902052 | up |
| MELO3C018713.2 | 27662 | 8066 | -1.778 | 9.66946E-49 | down |
| MELO3C018715.2 | 7083 | 2850 | -1.314 | 1.63638E-53 | down |
| MELO3C018718.2 | 2254 | 309 | -2.866 | 2.66911E-131 | down |
| MELO3C018720.2 | 12326 | 2968 | -2.054 | 5.85505E-85 | down |
| MELO3C018721.2 | 723 | 1557 | 1.108 | 1.71014E-14 | up |
| MELO3C018723.2 | 2670 | 5921 | 1.149 | 6.36704E-22 | up |
| MELO3C018724.2 | 14337 | 6175 | -1.215 | 7.84727E-46 | down |
| MELO3C018729.2 | 1716 | 85 | -4.33 | 1.33792E-20 | down |
| MELO3C018732.2 | 60 | 284 | 2.245 | 1.64252E-13 | up |
| MELO3C018733.2 | 1083 | 220 | -2.298 | 1.07577E-29 | down |
| MELO3C018738.2 | 716 | 48 | -3.892 | 2.95071E-80 | down |
| MELO3C018739.2 | 41 | 1 | -6.078 | 1.64719E-07 | down |
| MELO3C018740.2 | 148 | 362 | 1.29 | 1.04728E-08 | up |
| MELO3C018742.2 | 269 | 106 | -1.335 | 2.56507E-10 | down |
| MELO3C018744.2 | 51 | 4 | -3.556 | 8.71331E-07 | down |
| MELO3C018747.2 | 46 | 160 | 1.804 | 5.5847E-10 | up |
| MELO3C018748.2 | 2133 | 161 | -3.725 | 1.87911E-64 | down |
| MELO3C018749.2 | 988 | 254 | -1.959 | 5.88268E-63 | down |
| MELO3C018758.2 | 76 | 281 | 1.884 | 2.16235E-11 | up |
| MELO3C018762.2 | 2664 | 1060 | -1.331 | 7.25214E-28 | down |
| MELO3C018767.2 | 418 | 196 | -1.09 | 3.56293E-07 | down |
| MELO3C018770.2 | 970 | 450 | -1.11 | 6.3784E-13 | down |
| MELO3C018774.2 | 1 | 102 | 6.573 | 4.4503E-13 | up |
| MELO3C018775.2 | 8653 | 2250 | -1.943 | 6.12011E-28 | down |
| MELO3C018778.2 | 115 | 42 | -1.459 | 2.2744E-07 | down |
| MELO3C018782.2 | 350 | 859 | 1.297 | 1.27083E-16 | up |
| MELO3C018784.2 | 6 | 18 | 1.559 | 0.029803581 | up |
| MELO3C018785.2 | 58 | 2 | -5.241 | 2.37477E-09 | down |
| MELO3C018798.2 | 35 | 4 | -3.125 | 0.002423714 | down |
| MELO3C018799.2 | 39 | 2 | -4.364 | 3.16771E-05 | down |
| MELO3C018800.2 | 239 | 507 | 1.087 | 1.74927E-12 | up |
| MELO3C018813.2 | 3132 | 1190 | -1.396 | 2.19505E-23 | down |
| MELO3C018817.2 | 54 | 191 | 1.819 | 6.51321E-10 | up |
| MELO3C018819.2 | 1342 | 146 | -3.202 | 8.50341E-84 | down |
| MELO3C018823.2 | 0 | 16 | 6.357 | 1.18335E-05 | up |
| MELO3C018824.2 | 29 | 10 | -1.6 | 0.005275737 | down |
| MELO3C018828.2 | 126 | 273 | 1.114 | 2.31869E-08 | up |
| MELO3C018830.2 | 133 | 424 | 1.67 | 3.8889E-20 | up |
| MELO3C018831.2 | 86 | 246 | 1.509 | 1.82475E-10 | up |
| MELO3C018839.2 | 930 | 338 | -1.462 | 5.02906E-14 | down |
| MELO3C018844.2 | 397 | 88 | -2.176 | 1.22306E-21 | down |
| MELO3C018850.2 | 19 | 0 | -6.805 | 8.27025E-06 | down |
| MELO3C018851.2 | 143 | 3 | -5.695 | 1.59869E-21 | down |
| MELO3C018853.2 | 36 | 80 | 1.171 | 0.000378036 | up |
| MELO3C018856.2 | 4302 | 628 | -2.778 | 1.00546E-39 | down |
| MELO3C018858.2 | 6 | 20 | 1.692 | 0.029166107 | up |
| MELO3C018859.2 | 854 | 123 | -2.802 | 1.19831E-61 | down |
| MELO3C018873.2 | 34 | 178 | 2.385 | 1.71462E-17 | up |
| MELO3C018878.2 | 156 | 9476 | 5.92 | 8.20598E-16 | up |
| MELO3C018879.2 | 8 | 0 | -5.524 | 0.001565962 | down |
| MELO3C018880.2 | 40202 | 18968 | -1.084 | 1.77629E-10 | down |
| MELO3C018881.2 | 0 | 359 | 10.829 | 1.06825E-19 | up |
| MELO3C018886.2 | 18 | 4 | -2.397 | 0.013223778 | down |
| MELO3C018888.2 | 16 | 1567 | 6.636 | 8.84693E-152 | up |
| MELO3C018892.2 | 8086 | 989 | -3.031 | 2.50139E-239 | down |
| MELO3C018897.2 | 213 | 473 | 1.15 | 4.08985E-11 | up |
| MELO3C018925.2 | 9 | 0 | -5.771 | 0.000486251 | down |
| MELO3C018937.2 | 16641 | 31 | -9.046 | 0 | down |
| MELO3C018942.2 | 92 | 269 | 1.556 | 6.23675E-10 | up |
| MELO3C018959.2 | 32 | 7 | -2.251 | 0.000378516 | down |
| MELO3C018967.2 | 516 | 116 | -2.156 | 0.020356742 | down |
| MELO3C018993.2 | 155 | 316 | 1.023 | 1.63891E-08 | up |
| MELO3C018994.2 | 8763 | 2562 | -1.774 | 2.01871E-59 | down |
| MELO3C019002.2 | 1 | 172 | 6.907 | 1.76227E-17 | up |
| MELO3C019017.2 | 69 | 0 | -8.658 | 1.23205E-11 | down |
| MELO3C019025.2 | 0 | 39 | 7.615 | 2.54452E-08 | up |
| MELO3C019065.2 | 13 | 0 | -6.198 | 4.31612E-05 | down |
| MELO3C019076.2 | 1 | 11 | 2.983 | 0.009496698 | up |
| MELO3C019110.2 | 188 | 31 | -2.614 | 3.89243E-18 | down |
| MELO3C019112.2 | 491 | 1362 | 1.474 | 6.99853E-32 | up |
| MELO3C019125.2 | 282 | 57 | -2.312 | 4.87896E-19 | down |
| MELO3C019136.2 | 877 | 2018 | 1.203 | 8.63482E-24 | up |
| MELO3C019148.2 | 2135 | 364 | -2.553 | 1.31929E-42 | down |
| MELO3C019150.2 | 0 | 215 | 10.086 | 2.69175E-16 | up |
| MELO3C019161.2 | 34 | 250 | 2.891 | 6.0974E-13 | up |
| MELO3C019177.2 | 205 | 25 | -3.026 | 1.13133E-15 | down |
| MELO3C019186.2 | 10 | 0 | -5.937 | 0.00098894 | down |
| MELO3C019188.2 | 50 | 10 | -2.285 | 0.00011761 | down |
| MELO3C019192.2 | 10 | 42 | 2.056 | 0.000294985 | up |
| MELO3C019202.2 | 1 | 13 | 3.156 | 0.009358525 | up |
| MELO3C019203.2 | 382 | 28 | -3.772 | 1.44406E-43 | down |
| MELO3C019205.2 | 83 | 773 | 3.23 | 9.17557E-58 | up |
| MELO3C019210.2 | 24 | 0 | -7.132 | 7.50558E-07 | down |
| MELO3C019215.2 | 12688 | 3861 | -1.716 | 1.69641E-49 | down |
| MELO3C019221.2 | 51 | 0 | -8.218 | 3.66564E-10 | down |
| MELO3C019224.2 | 4 | 0 | -4.654 | 0.023802888 | down |
| MELO3C019227.2 | 4 | 17 | 2.034 | 0.018121545 | up |
| MELO3C019231.2 | 1268 | 222 | -2.517 | 2.0489E-44 | down |
| MELO3C019232.2 | 70 | 6 | -3.497 | 1.02431E-11 | down |
| MELO3C019247.2 | 30 | 1 | -5.037 | 8.08583E-06 | down |
| MELO3C019254.2 | 393 | 846 | 1.107 | 1.22058E-07 | up |
| MELO3C019257.2 | 13 | 64 | 2.361 | 5.67186E-07 | up |
| MELO3C019266.2 | 99 | 1087 | 3.461 | 1.49514E-114 | up |
| MELO3C019267.2 | 236 | 633 | 1.425 | 3.85359E-10 | up |
| MELO3C019273.2 | 1140 | 395 | -1.529 | 7.15994E-22 | down |
| MELO3C019291.2 | 3 | 19 | 2.739 | 0.000687704 | up |
| MELO3C019297.2 | 42 | 3 | -3.776 | 9.96231E-08 | down |
| MELO3C019303.2 | 95 | 382 | 2.002 | 7.69753E-23 | up |
| MELO3C019310.2 | 3846 | 438 | -3.131 | 1.20684E-140 | down |
| MELO3C019320.2 | 1180 | 484 | -1.288 | 1.97569E-26 | down |
| MELO3C019335.2 | 150 | 24 | -2.651 | 1.92309E-14 | down |
| MELO3C019348.2 | 1154 | 523 | -1.142 | 1.10479E-28 | down |
| MELO3C019360.2 | 270 | 547 | 1.015 | 1.80688E-12 | up |
| MELO3C019361.2 | 8 | 64 | 3.099 | 1.27622E-09 | up |
| MELO3C019363.2 | 34609 | 72641 | 1.07 | 1.62798E-29 | up |
| MELO3C019366.2 | 1084 | 330 | -1.718 | 1.28692E-39 | down |
| MELO3C019368.2 | 21 | 6 | -1.787 | 0.006742053 | down |
| MELO3C019370.2 | 50 | 103 | 1.038 | 0.002783811 | up |
| MELO3C019373.2 | 3353 | 962 | -1.803 | 4.97789E-47 | down |
| MELO3C019377.2 | 964 | 67 | -3.85 | 9.33179E-93 | down |
| MELO3C019379.2 | 43 | 130 | 1.6 | 1.53144E-06 | up |
| MELO3C019382.2 | 2551 | 438 | -2.543 | 3.30715E-29 | down |
| MELO3C019393.2 | 293 | 68 | -2.111 | 1.43088E-23 | down |
| MELO3C019394.2 | 19 | 62 | 1.762 | 3.67777E-05 | up |
| MELO3C019410.2 | 12902 | 905 | -3.834 | 0 | down |
| MELO3C019418.2 | 455 | 106 | -2.105 | 3.10186E-34 | down |
| MELO3C019435.2 | 7 | 929 | 6.95 | 1.89091E-87 | up |
| MELO3C019437.2 | 0 | 9 | 5.596 | 0.000472943 | up |
| MELO3C019440.2 | 11500 | 1440 | -2.998 | 8.04593E-52 | down |
| MELO3C019441.2 | 548 | 207 | -1.404 | 3.85682E-05 | down |
| MELO3C019445.2 | 54 | 270 | 2.312 | 7.39791E-24 | up |
| MELO3C019447.2 | 158 | 1118 | 2.821 | 1.20644E-92 | up |
| MELO3C019451.2 | 30 | 154 | 2.34 | 2.49351E-12 | up |
| MELO3C019453.2 | 210 | 638 | 1.607 | 3.50427E-19 | up |
| MELO3C019454.2 | 3042 | 8733 | 1.522 | 4.59958E-43 | up |
| MELO3C019470.2 | 5589 | 1 | -12.148 | 9.74908E-63 | down |
| MELO3C019474.2 | 8 | 0 | -5.532 | 0.001159456 | down |
| MELO3C019475.2 | 1424 | 651 | -1.13 | 1.22886E-36 | down |
| MELO3C019497.2 | 5816 | 2377 | -1.291 | 3.87654E-39 | down |
| MELO3C019506.2 | 103 | 1 | -6.438 | 1.6317E-05 | down |
| MELO3C019517.2 | 87 | 192 | 1.136 | 0.000121069 | up |
| MELO3C019529.2 | 204 | 66 | -1.618 | 4.64091E-07 | down |
| MELO3C019537.2 | 49 | 228 | 2.212 | 1.74649E-09 | up |
| MELO3C019544.2 | 31 | 142 | 2.202 | 2.03617E-11 | up |
| MELO3C019548.2 | 473 | 187 | -1.338 | 9.27735E-13 | down |
| MELO3C019552.2 | 374 | 32 | -3.544 | 2.60625E-38 | down |
| MELO3C019561.2 | 423 | 133 | -1.66 | 3.01315E-13 | down |
| MELO3C019563.2 | 203 | 415 | 1.029 | 1.82847E-07 | up |
| MELO3C019567.2 | 11 | 1 | -4.207 | 0.005141565 | down |
| MELO3C019572.2 | 1697 | 327 | -2.377 | 1.32723E-50 | down |
| MELO3C019573.2 | 4556 | 12699 | 1.479 | 3.43023E-37 | up |
| MELO3C019588.2 | 304 | 774 | 1.346 | 1.22535E-30 | up |
| MELO3C019598.2 | 2867 | 734 | -1.966 | 1.79912E-37 | down |
| MELO3C019599.2 | 868 | 308 | -1.499 | 1.93294E-12 | down |
| MELO3C019602.2 | 5238 | 2317 | -1.177 | 1.23254E-26 | down |
| MELO3C019603.2 | 1728 | 630 | -1.457 | 2.01546E-18 | down |
| MELO3C019621.2 | 792 | 234 | -1.758 | 8.68067E-16 | down |
| MELO3C019634.2 | 14976 | 5036 | -1.572 | 6.81439E-32 | down |
| MELO3C019639.2 | 589 | 86 | -2.79 | 7.94734E-26 | down |
| MELO3C019649.2 | 222 | 44 | -2.334 | 1.91386E-14 | down |
| MELO3C019654.2 | 54 | 2 | -4.846 | 1.20278E-10 | down |
| MELO3C019677.2 | 78883 | 667 | -6.886 | 0 | down |
| MELO3C019680.2 | 22 | 1 | -5.17 | 3.08078E-05 | down |
| MELO3C019689.2 | 110 | 54 | -1.036 | 9.22623E-05 | down |
| MELO3C019691.2 | 228 | 1455 | 2.673 | 5.01217E-93 | up |
| MELO3C019723.2 | 396 | 958 | 1.274 | 1.01322E-18 | up |
| MELO3C019734.2 | 1356 | 2903 | 1.099 | 5.9393E-09 | up |
| MELO3C019735.2 | 4488 | 938 | -2.258 | 1.69267E-27 | down |
| MELO3C019745.2 | 386 | 0 | -11.144 | 4.45933E-20 | down |
| MELO3C019759.2 | 2020 | 238 | -3.084 | 2.58394E-51 | down |
| MELO3C019772.2 | 5 | 0 | -4.872 | 0.014761363 | down |
| MELO3C019783.2 | 154 | 310 | 1.006 | 9.63632E-10 | up |
| MELO3C019791.2 | 337 | 1459 | 2.117 | 1.94513E-28 | up |
| MELO3C019796.2 | 12 | 1 | -3.723 | 0.004015126 | down |
| MELO3C019808.2 | 179 | 5 | -5.091 | 3.7449E-27 | down |
| MELO3C019810.2 | 68 | 280 | 2.052 | 5.55598E-19 | up |
| MELO3C019818.2 | 85 | 418 | 2.301 | 6.86605E-41 | up |
| MELO3C019832.2 | 4456 | 2074 | -1.104 | 4.22088E-16 | down |
| MELO3C019833.2 | 0 | 825 | 12.028 | 4.20389E-24 | up |
| MELO3C019840.2 | 375 | 3069 | 3.031 | 1.61814E-193 | up |
| MELO3C019842.2 | 12 | 50 | 2.087 | 1.74888E-05 | up |
| MELO3C019843.2 | 1 | 20 | 3.801 | 0.00022757 | up |
| MELO3C019846.2 | 752 | 1601 | 1.09 | 6.62784E-17 | up |
| MELO3C019849.2 | 2 | 126 | 5.652 | 7.89555E-20 | up |
| MELO3C019852.2 | 687 | 316 | -1.121 | 1.53267E-15 | down |
| MELO3C019853.2 | 534 | 1070 | 1.001 | 1.2446E-11 | up |
| MELO3C019871.2 | 915 | 3498 | 1.934 | 5.0753E-29 | up |
| MELO3C019897.2 | 76 | 24 | -1.682 | 4.84757E-07 | down |
| MELO3C019904.2 | 1674 | 673 | -1.316 | 1.16238E-36 | down |
| MELO3C019906.2 | 446 | 206 | -1.11 | 0.001070402 | down |
| MELO3C019912.2 | 1802 | 625 | -1.526 | 2.68327E-30 | down |
| MELO3C019917.2 | 47 | 1 | -6.272 | 4.30212E-08 | down |
| MELO3C019919.2 | 25 | 55 | 1.131 | 0.007119903 | up |
| MELO3C019929.2 | 567 | 279 | -1.027 | 5.04536E-08 | down |
| MELO3C019930.2 | 60 | 440 | 2.872 | 9.08926E-36 | up |
| MELO3C019932.2 | 223 | 99 | -1.172 | 1.11226E-08 | down |
| MELO3C019934.2 | 3 | 12 | 2.048 | 0.025566255 | up |
| MELO3C019939.2 | 768 | 2386 | 1.636 | 4.11442E-30 | up |
| MELO3C019941.2 | 8583 | 2881 | -1.575 | 3.69514E-83 | down |
| MELO3C019948.2 | 1634 | 76 | -4.425 | 7.67986E-119 | down |
| MELO3C019962.2 | 172 | 419 | 1.282 | 3.46927E-16 | up |
| MELO3C019964.2 | 59 | 147 | 1.311 | 2.54858E-06 | up |
| MELO3C019965.2 | 4 | 35 | 3.043 | 3.28057E-05 | up |
| MELO3C019970.2 | 14 | 69 | 2.266 | 8.89384E-08 | up |
| MELO3C019981.2 | 4898 | 1593 | -1.62 | 9.37821E-52 | down |
| MELO3C019982.2 | 312 | 98 | -1.666 | 1.07703E-17 | down |
| MELO3C019983.2 | 9297 | 3188 | -1.544 | 3.53646E-60 | down |
| MELO3C019997.2 | 276 | 1448 | 2.389 | 1.36612E-48 | up |
| MELO3C019998.2 | 25 | 173 | 2.789 | 1.43444E-13 | up |
| MELO3C019999.2 | 70 | 160 | 1.186 | 9.40196E-05 | up |
| MELO3C020000.2 | 93 | 22 | -2.1 | 1.75781E-08 | down |
| MELO3C020001.2 | 309 | 743 | 1.264 | 1.95357E-14 | up |
| MELO3C020005.2 | 500 | 9849 | 4.299 | 6.50608E-87 | up |
| MELO3C020007.2 | 4114 | 84 | -5.61 | 1.16149E-164 | down |
| MELO3C020010.2 | 299 | 716 | 1.261 | 2.87475E-13 | up |
| MELO3C020011.2 | 71 | 279 | 1.971 | 4.31225E-17 | up |
| MELO3C020032.2 | 36 | 167 | 2.205 | 2.78337E-10 | up |
| MELO3C020052.2 | 109 | 14 | -2.97 | 1.40261E-11 | down |
| MELO3C020056.2 | 21 | 5 | -2 | 0.007353656 | down |
| MELO3C020061.2 | 32 | 0 | -7.53 | 2.26131E-08 | down |
| MELO3C020063.2 | 0 | 25 | 7.017 | 1.62315E-06 | up |
| MELO3C020091.2 | 28 | 5 | -2.585 | 0.000757922 | down |
| MELO3C020093.2 | 1513 | 3193 | 1.078 | 1.82398E-29 | up |
| MELO3C020095.2 | 196 | 46 | -2.098 | 1.38302E-19 | down |
| MELO3C020096.2 | 157 | 39 | -2.003 | 6.39415E-12 | down |
| MELO3C020099.2 | 262 | 62 | -2.088 | 2.17761E-10 | down |
| MELO3C020101.2 | 574 | 1168 | 1.024 | 2.89305E-17 | up |
| MELO3C020105.2 | 5 | 57 | 3.499 | 1.42001E-05 | up |
| MELO3C020115.2 | 3669 | 1165 | -1.654 | 5.14449E-25 | down |
| MELO3C020120.2 | 664 | 0 | -11.928 | 1.48309E-23 | down |
| MELO3C020121.2 | 0 | 91 | 8.85 | 8.46533E-13 | up |
| MELO3C020131.2 | 2642 | 119 | -4.472 | 4.28473E-99 | down |
| MELO3C020132.2 | 1234 | 448 | -1.46 | 7.45616E-12 | down |
| MELO3C020138.2 | 4016 | 1469 | -1.45 | 8.28933E-25 | down |
| MELO3C020146.2 | 7189 | 16470 | 1.196 | 0.001137872 | up |
| MELO3C020156.2 | 0 | 296 | 10.548 | 1.65075E-18 | up |
| MELO3C020166.2 | 22 | 3 | -2.855 | 0.000972805 | down |
| MELO3C020181.2 | 1325 | 2991 | 1.175 | 3.95587E-38 | up |
| MELO3C020192.2 | 7996 | 3594 | -1.154 | 5.16832E-20 | down |
| MELO3C020257.2 | 0 | 27 | 7.094 | 1.29551E-07 | up |
| MELO3C020268.2 | 207 | 918 | 2.147 | 7.79163E-14 | up |
| MELO3C020278.2 | 583 | 287 | -1.021 | 3.46022E-13 | down |
| MELO3C020303.2 | 206 | 449 | 1.126 | 9.81354E-13 | up |
| MELO3C020312.2 | 4616 | 2101 | -1.136 | 9.80458E-20 | down |
| MELO3C020317.2 | 158 | 1461 | 3.21 | 2.98568E-49 | up |
| MELO3C020318.2 | 377 | 171 | -1.138 | 1.85428E-09 | down |
| MELO3C020321.2 | 1008 | 2588 | 1.36 | 2.6186E-44 | up |
| MELO3C020328.2 | 130 | 62 | -1.076 | 0.000487581 | down |
| MELO3C020363.2 | 914 | 448 | -1.026 | 1.22281E-15 | down |
| MELO3C020370.2 | 284 | 2 | -7.523 | 1.58274E-28 | down |
| MELO3C020373.2 | 9 | 100 | 3.545 | 3.10039E-12 | up |
| MELO3C020375.2 | 142 | 504 | 1.822 | 5.22669E-19 | up |
| MELO3C020377.2 | 14 | 199 | 3.796 | 2.66961E-26 | up |
| MELO3C020393.2 | 57 | 21 | -1.406 | 0.001743268 | down |
| MELO3C020398.2 | 3047 | 566 | -2.428 | 4.63771E-08 | down |
| MELO3C020400.2 | 69 | 26 | -1.402 | 0.000381978 | down |
| MELO3C020408.2 | 971 | 2339 | 1.269 | 4.87994E-18 | up |
| MELO3C020418.2 | 73 | 0 | -8.749 | 1.79031E-12 | down |
| MELO3C020419.2 | 78 | 1 | -6.985 | 1.94344E-10 | down |
| MELO3C020421.2 | 0 | 604 | 11.579 | 2.2146E-22 | up |
| MELO3C020426.2 | 5287 | 17567 | 1.732 | 1.48584E-22 | up |
| MELO3C020428.2 | 301 | 6414 | 4.414 | 3.12541E-29 | up |
| MELO3C020432.2 | 27808 | 70566 | 1.343 | 1.2682E-70 | up |
| MELO3C020434.2 | 2 | 115 | 5.749 | 3.6562E-17 | up |
| MELO3C020446.2 | 236 | 50 | -2.235 | 9.2668E-21 | down |
| MELO3C020457.2 | 2 | 281 | 7.021 | 1.17534E-07 | up |
| MELO3C020464.2 | 37 | 6 | -2.516 | 9.82746E-06 | down |
| MELO3C020485.2 | 444 | 209 | -1.083 | 1.67758E-12 | down |
| MELO3C020486.2 | 2005 | 4359 | 1.12 | 2.25932E-60 | up |
| MELO3C020496.2 | 2688 | 55 | -5.617 | 2.55177E-24 | down |
| MELO3C020508.2 | 510 | 1294 | 1.345 | 3.64603E-22 | up |
| MELO3C020510.2 | 2607 | 772 | -1.753 | 1.23556E-78 | down |
| MELO3C020515.2 | 3506 | 816 | -2.104 | 6.41134E-61 | down |
| MELO3C020521.2 | 2379 | 6190 | 1.379 | 3.01315E-37 | up |
| MELO3C020532.2 | 988 | 460 | -1.104 | 7.67967E-08 | down |
| MELO3C020535.2 | 1248 | 12 | -6.707 | 1.73761E-21 | down |
| MELO3C020536.2 | 495 | 169 | -1.548 | 5.29416E-06 | down |
| MELO3C020537.2 | 61 | 290 | 2.245 | 7.78347E-11 | up |
| MELO3C020541.2 | 33 | 813 | 4.641 | 1.78133E-85 | up |
| MELO3C020542.2 | 12 | 48 | 2.067 | 8.31835E-06 | up |
| MELO3C020543.2 | 5797 | 2814 | -1.043 | 9.05203E-31 | down |
| MELO3C020547.2 | 347 | 154 | -1.178 | 2.12518E-08 | down |
| MELO3C020554.2 | 2593 | 938 | -1.468 | 8.46578E-48 | down |
| MELO3C020560.2 | 1740 | 800 | -1.122 | 1.27172E-16 | down |
| MELO3C020570.2 | 525 | 9837 | 4.228 | 5.45323E-152 | up |
| MELO3C020588.2 | 2915 | 301 | -3.275 | 9.32992E-58 | down |
| MELO3C020589.2 | 9104 | 2410 | -1.918 | 8.59131E-59 | down |
| MELO3C020591.2 | 32 | 4765 | 7.193 | 0 | up |
| MELO3C020594.2 | 3924 | 7 | -9.112 | 1.81059E-175 | down |
| MELO3C020595.2 | 360 | 893 | 1.312 | 1.01099E-16 | up |
| MELO3C020596.2 | 22 | 64 | 1.575 | 4.15499E-05 | up |
| MELO3C020597.2 | 238 | 541 | 1.184 | 1.03876E-11 | up |
| MELO3C020600.2 | 148 | 589 | 1.994 | 1.32937E-16 | up |
| MELO3C020605.2 | 178 | 467 | 1.389 | 2.38386E-19 | up |
| MELO3C020609.2 | 240 | 793 | 1.725 | 1.20643E-33 | up |
| MELO3C020611.2 | 191 | 404 | 1.079 | 4.99087E-11 | up |
| MELO3C020630.2 | 195 | 1459 | 2.907 | 9.2627E-09 | up |
| MELO3C020631.2 | 33 | 10 | -1.704 | 0.009248638 | down |
| MELO3C020637.2 | 872 | 2299 | 1.398 | 1.01082E-17 | up |
| MELO3C020644.2 | 661 | 238 | -1.476 | 7.7115E-16 | down |
| MELO3C020649.2 | 1530 | 749 | -1.031 | 4.38367E-12 | down |
| MELO3C020652.2 | 3931 | 1702 | -1.208 | 1.75408E-21 | down |
| MELO3C020653.2 | 14 | 3 | -2.181 | 0.021460608 | down |
| MELO3C020655.2 | 8 | 0 | -5.598 | 0.001102873 | down |
| MELO3C020660.2 | 10 | 0 | -5.931 | 0.000260293 | down |
| MELO3C020675.2 | 5947 | 1686 | -1.82 | 8.52285E-53 | down |
| MELO3C020676.2 | 150 | 400 | 1.419 | 5.73355E-08 | up |
| MELO3C020681.2 | 856 | 29 | -4.891 | 1.60008E-75 | down |
| MELO3C020688.2 | 421 | 48 | -3.125 | 1.07805E-53 | down |
| MELO3C020692.2 | 0 | 5 | 4.672 | 0.020214643 | up |
| MELO3C020695.2 | 48 | 108 | 1.151 | 2.82616E-05 | up |
| MELO3C020699.2 | 11 | 2 | -2.499 | 0.029361159 | down |
| MELO3C020725.2 | 1668 | 695 | -1.262 | 1.40434E-15 | down |
| MELO3C020732.2 | 469 | 1038 | 1.147 | 1.67441E-19 | up |
| MELO3C020742.2 | 11 | 125 | 3.535 | 5.58522E-19 | up |
| MELO3C020749.2 | 2253 | 1036 | -1.121 | 1.43041E-35 | down |
| MELO3C020751.2 | 2 | 14 | 2.736 | 0.006659807 | up |
| MELO3C020752.2 | 12 | 303 | 4.685 | 3.26083E-30 | up |
| MELO3C020761.2 | 45 | 8 | -2.482 | 0.001013085 | down |
| MELO3C020765.2 | 77 | 5 | -3.956 | 2.05355E-10 | down |
| MELO3C020767.2 | 1 | 39 | 4.752 | 1.9223E-07 | up |
| MELO3C020771.2 | 54 | 14 | -1.918 | 2.51621E-05 | down |
| MELO3C020772.2 | 138 | 51 | -1.45 | 7.86584E-06 | down |
| MELO3C020782.2 | 37 | 0 | -6.811 | 1.90857E-07 | down |
| MELO3C020791.2 | 32 | 129 | 2.008 | 0.003777556 | up |
| MELO3C020792.2 | 14 | 91 | 2.707 | 3.01086E-12 | up |
| MELO3C020793.2 | 16 | 213 | 3.754 | 1.02992E-17 | up |
| MELO3C020796.2 | 11 | 1 | -4.272 | 0.006913677 | down |
| MELO3C020798.2 | 2227 | 245 | -3.183 | 2.4543E-38 | down |
| MELO3C020799.2 | 199 | 3 | -6.031 | 1.42102E-28 | down |
| MELO3C020802.2 | 388 | 847 | 1.126 | 2.50363E-23 | up |
| MELO3C020803.2 | 2 | 29 | 3.547 | 5.5772E-06 | up |
| MELO3C020805.2 | 25 | 0 | -7.166 | 1.22243E-05 | down |
| MELO3C020809.2 | 44 | 89 | 1.033 | 0.001678384 | up |
| MELO3C020810.2 | 7026 | 20159 | 1.521 | 4.8578E-41 | up |
| MELO3C020811.2 | 1990 | 604 | -1.719 | 1.82035E-22 | down |
| MELO3C020812.2 | 435 | 137 | -1.662 | 1.28292E-22 | down |
| MELO3C020817.2 | 52 | 1819 | 5.131 | 6.67711E-135 | up |
| MELO3C020822.2 | 2922 | 836 | -1.806 | 1.12903E-52 | down |
| MELO3C020823.2 | 1189 | 516 | -1.204 | 5.35596E-26 | down |
| MELO3C020830.2 | 47 | 155 | 1.734 | 7.90371E-10 | up |
| MELO3C020835.2 | 26 | 94 | 1.878 | 4.08547E-07 | up |
| MELO3C020844.2 | 671 | 110 | -2.603 | 1.2767E-45 | down |
| MELO3C020845.2 | 1020 | 277 | -1.882 | 1.76398E-37 | down |
| MELO3C020848.2 | 285 | 840 | 1.56 | 9.33131E-26 | up |
| MELO3C020855.2 | 796 | 1699 | 1.093 | 6.00224E-24 | up |
| MELO3C020857.2 | 3 | 34 | 3.242 | 0.000207901 | up |
| MELO3C020860.2 | 125 | 656 | 2.393 | 1.71042E-22 | up |
| MELO3C020867.2 | 53 | 913 | 4.112 | 3.5399E-52 | up |
| MELO3C020872.2 | 10315 | 4500 | -1.197 | 5.14826E-47 | down |
| MELO3C020877.2 | 972 | 2760 | 1.507 | 7.34157E-19 | up |
| MELO3C020886.2 | 2 | 26 | 3.584 | 0.000543797 | up |
| MELO3C020900.2 | 330 | 64 | -2.361 | 1.12377E-31 | down |
| MELO3C020901.2 | 18 | 60 | 1.729 | 5.66975E-05 | up |
| MELO3C020912.2 | 7659 | 3638 | -1.074 | 2.16935E-20 | down |
| MELO3C020913.2 | 12955 | 4629 | -1.485 | 1.96642E-77 | down |
| MELO3C020914.2 | 19 | 3 | -2.628 | 0.001039713 | down |
| MELO3C020915.2 | 492 | 94 | -2.397 | 2.39846E-41 | down |
| MELO3C020917.2 | 1739 | 370 | -2.234 | 2.53785E-23 | down |
| MELO3C020919.2 | 1665 | 603 | -1.467 | 4.7395E-48 | down |
| MELO3C020927.2 | 223 | 466 | 1.066 | 2.87311E-11 | up |
| MELO3C020931.2 | 465 | 207 | -1.169 | 8.82462E-15 | down |
| MELO3C020932.2 | 268 | 788 | 1.555 | 1.02696E-23 | up |
| MELO3C020934.2 | 26 | 0 | -6.289 | 0.000140757 | down |
| MELO3C020941.2 | 4655 | 1651 | -1.496 | 4.26748E-49 | down |
| MELO3C020942.2 | 2950 | 1143 | -1.368 | 4.6352E-11 | down |
| MELO3C020956.2 | 1 | 13 | 4.198 | 0.003596745 | up |
| MELO3C020963.2 | 80 | 19 | -2.038 | 9.59711E-05 | down |
| MELO3C020965.2 | 247 | 87 | -1.503 | 4.63983E-13 | down |
| MELO3C020967.2 | 30 | 10 | -1.591 | 0.002114153 | down |
| MELO3C020975.2 | 78 | 27 | -1.531 | 0.003392769 | down |
| MELO3C020979.2 | 804 | 179 | -2.158 | 3.14026E-47 | down |
| MELO3C020980.2 | 1351 | 3250 | 1.266 | 2.02101E-33 | up |
| MELO3C020982.2 | 205 | 5 | -5.487 | 5.94805E-33 | down |
| MELO3C020984.2 | 371 | 10 | -5.091 | 8.54978E-42 | down |
| MELO3C020989.2 | 338 | 691 | 1.027 | 7.8851E-08 | up |
| MELO3C020997.2 | 132 | 535 | 2.02 | 9.6477E-24 | up |
| MELO3C020998.2 | 710 | 137 | -2.371 | 4.08577E-55 | down |
| MELO3C020999.2 | 89 | 220 | 1.306 | 1.00245E-10 | up |
| MELO3C021000.2 | 3 | 55 | 4.271 | 6.21809E-09 | up |
| MELO3C021005.2 | 0 | 1612 | 12.994 | 5.09107E-28 | up |
| MELO3C021012.2 | 317 | 48 | -2.735 | 3.13485E-39 | down |
| MELO3C021014.2 | 7 | 63 | 3.206 | 1.10311E-08 | up |
| MELO3C021015.2 | 0 | 20 | 6.636 | 8.42338E-06 | up |
| MELO3C021031.2 | 216 | 1707 | 2.982 | 8.37205E-63 | up |
| MELO3C021033.2 | 284 | 631 | 1.151 | 1.05603E-14 | up |
| MELO3C021034.2 | 1490 | 659 | -1.176 | 3.75371E-19 | down |
| MELO3C021047.2 | 740 | 244 | -1.599 | 2.03376E-26 | down |
| MELO3C021050.2 | 0 | 130 | 9.369 | 1.28931E-14 | up |
| MELO3C021053.2 | 915 | 410 | -1.16 | 8.90967E-17 | down |
| MELO3C021058.2 | 12 | 142 | 3.587 | 9.31305E-12 | up |
| MELO3C021060.2 | 411 | 28 | -3.889 | 2.85027E-54 | down |
| MELO3C021064.2 | 2942 | 822 | -1.837 | 6.18168E-27 | down |
| MELO3C021069.2 | 0 | 88 | 8.802 | 3.26429E-12 | up |
| MELO3C021071.2 | 129 | 325 | 1.332 | 2.01479E-10 | up |
| MELO3C021074.2 | 12274 | 3460 | -1.827 | 7.42765E-142 | down |
| MELO3C021075.2 | 8279 | 2905 | -1.511 | 3.70029E-48 | down |
| MELO3C021077.2 | 651 | 2792 | 2.101 | 7.37845E-41 | up |
| MELO3C021079.2 | 166 | 2032 | 3.61 | 2.47781E-93 | up |
| MELO3C021083.2 | 258 | 794 | 1.618 | 2.22223E-24 | up |
| MELO3C021091.2 | 994 | 363 | -1.455 | 1.60737E-09 | down |
| MELO3C021100.2 | 1 | 1523 | 10.055 | 4.30167E-43 | up |
| MELO3C021106.2 | 109 | 379 | 1.795 | 3.30322E-16 | up |
| MELO3C021111.2 | 32 | 11 | -1.543 | 0.004020358 | down |
| MELO3C021113.2 | 2018 | 996 | -1.02 | 9.19837E-21 | down |
| MELO3C021118.2 | 625 | 2363 | 1.918 | 7.08368E-81 | up |
| MELO3C021125.2 | 1116 | 3205 | 1.522 | 1.20288E-67 | up |
| MELO3C021132.2 | 4 | 19 | 2.253 | 0.004087787 | up |
| MELO3C021137.2 | 17 | 901 | 5.705 | 2.98692E-11 | up |
| MELO3C021142.2 | 4 | 0 | -4.655 | 0.020298081 | down |
| MELO3C021143.2 | 4132 | 23 | -7.447 | 2.03373E-31 | down |
| MELO3C021144.2 | 134 | 409 | 1.607 | 7.8788E-07 | up |
| MELO3C021148.2 | 2151 | 1017 | -1.081 | 2.16028E-37 | down |
| MELO3C021153.2 | 6002 | 1406 | -2.094 | 4.38905E-142 | down |
| MELO3C021156.2 | 5 | 119 | 4.572 | 1.87436E-18 | up |
| MELO3C021167.2 | 500 | 156 | -1.678 | 2.14439E-13 | down |
| MELO3C021170.2 | 1733 | 700 | -1.309 | 3.60488E-40 | down |
| MELO3C021171.2 | 70350 | 29916 | -1.234 | 7.44142E-55 | down |
| MELO3C021176.2 | 6856 | 1713 | -2 | 2.3096E-46 | down |
| MELO3C021178.2 | 4 | 35 | 3.133 | 8.44819E-06 | up |
| MELO3C021180.2 | 17 | 79 | 2.181 | 1.75385E-08 | up |
| MELO3C021182.2 | 320 | 3 | -7.008 | 6.87626E-34 | down |
| MELO3C021185.2 | 1383 | 352 | -1.974 | 1.76178E-44 | down |
| MELO3C021190.2 | 85 | 203 | 1.25 | 8.68637E-08 | up |
| MELO3C021193.2 | 9 | 160 | 4.154 | 4.02039E-24 | up |
| MELO3C021195.2 | 0 | 5 | 4.687 | 0.016436045 | up |
| MELO3C021196.2 | 6 | 31 | 2.405 | 0.000668804 | up |
| MELO3C021197.2 | 117 | 235 | 1.009 | 8.217E-07 | up |
| MELO3C021205.2 | 10 | 50 | 2.255 | 0.000160597 | up |
| MELO3C021206.2 | 526 | 6057 | 3.525 | 1.44453E-19 | up |
| MELO3C021211.2 | 10 | 85 | 3.048 | 5.31179E-08 | up |
| MELO3C021216.2 | 692 | 1849 | 1.419 | 1.67477E-40 | up |
| MELO3C021218.2 | 3473 | 1642 | -1.08 | 5.33793E-31 | down |
| MELO3C021230.2 | 19 | 4 | -2.308 | 0.028251975 | down |
| MELO3C021231.2 | 13828 | 4925 | -1.489 | 1.20445E-61 | down |
| MELO3C021233.2 | 2 | 46 | 4.675 | 4.68814E-09 | up |
| MELO3C021238.2 | 8 | 34 | 2.09 | 0.000665004 | up |
| MELO3C021240.2 | 690 | 1908 | 1.469 | 1.61118E-24 | up |
| MELO3C021245.2 | 257 | 60 | -2.111 | 3.94324E-18 | down |
| MELO3C021249.2 | 22936 | 6164 | -1.896 | 5.2701E-09 | down |
| MELO3C021253.2 | 8574 | 4052 | -1.081 | 8.29904E-44 | down |
| MELO3C021255.2 | 1036 | 484 | -1.098 | 3.29277E-16 | down |
| MELO3C021265.2 | 5586 | 2759 | -1.017 | 6.29841E-35 | down |
| MELO3C021281.2 | 10591 | 2502 | -2.082 | 1.349E-50 | down |
| MELO3C021282.2 | 19 | 1445 | 6.251 | 2.00546E-157 | up |
| MELO3C021303.2 | 304 | 655 | 1.107 | 4.34166E-18 | up |
| MELO3C021309.2 | 34 | 0 | -7.636 | 1.34671E-08 | down |
| MELO3C021310.2 | 359 | 33 | -3.458 | 4.03888E-43 | down |
| MELO3C021319.2 | 1 | 10 | 3.823 | 0.007227812 | up |
| MELO3C021322.2 | 65 | 589 | 3.175 | 1.84987E-50 | up |
| MELO3C021323.2 | 51 | 126 | 1.307 | 0.000535329 | up |
| MELO3C021326.2 | 608 | 181 | -1.747 | 1.05323E-27 | down |
| MELO3C021333.2 | 1474 | 3842 | 1.382 | 4.69109E-47 | up |
| MELO3C021334.2 | 156 | 0 | -9.837 | 5.48599E-16 | down |
| MELO3C021350.2 | 458 | 8 | -5.741 | 8.25982E-65 | down |
| MELO3C021351.2 | 140 | 2 | -6.487 | 1.60774E-18 | down |
| MELO3C021354.2 | 5 | 0 | -4.879 | 0.011179436 | down |
| MELO3C021358.2 | 42 | 10 | -2.063 | 1.14745E-05 | down |
| MELO3C021370.2 | 271 | 106 | -1.358 | 7.16285E-13 | down |
| MELO3C021375.2 | 40412 | 8270 | -2.289 | 3.41155E-39 | down |
| MELO3C021377.2 | 90 | 42 | -1.125 | 0.000895354 | down |
| MELO3C021379.2 | 989 | 236 | -2.06 | 2.58855E-22 | down |
| MELO3C021404.2 | 4299 | 11745 | 1.45 | 1.91683E-44 | up |
| MELO3C021406.2 | 3765 | 1070 | -1.816 | 2.90874E-42 | down |
| MELO3C021407.2 | 28074 | 6984 | -2.007 | 1.83204E-95 | down |
| MELO3C021412.2 | 3110 | 18 | -7.441 | 4.28949E-272 | down |
| MELO3C021416.2 | 1948 | 725 | -1.427 | 2.6273E-24 | down |
| MELO3C021421.2 | 119 | 29 | -2.05 | 3.97394E-10 | down |
| MELO3C021426.2 | 198 | 43 | -2.196 | 1.68995E-09 | down |
| MELO3C021427.2 | 112 | 26 | -2.093 | 7.7839E-09 | down |
| MELO3C021428.2 | 160 | 37 | -2.128 | 5.62109E-06 | down |
| MELO3C021433.2 | 6379 | 2682 | -1.25 | 1.17666E-15 | down |
| MELO3C021434.2 | 233 | 1124 | 2.268 | 4.21215E-31 | up |
| MELO3C021437.2 | 81 | 785 | 3.265 | 9.75249E-48 | up |
| MELO3C021447.2 | 26 | 431 | 4.065 | 5.10245E-31 | up |
| MELO3C021449.2 | 1659 | 522 | -1.67 | 2.5988E-35 | down |
| MELO3C021451.2 | 146 | 32 | -2.189 | 4.87208E-16 | down |
| MELO3C021455.2 | 16462 | 6640 | -1.31 | 9.40369E-40 | down |
| MELO3C021457.2 | 722 | 1458 | 1.014 | 1.16141E-11 | up |
| MELO3C021458.2 | 109 | 17478 | 7.327 | 1.65768E-37 | up |
| MELO3C021465.2 | 1741 | 5318 | 1.611 | 1.16238E-95 | up |
| MELO3C021466.2 | 2 | 18 | 3.057 | 0.001998498 | up |
| MELO3C021473.2 | 147 | 43 | -1.752 | 0.00069844 | down |
| MELO3C021483.2 | 320 | 904 | 1.5 | 2.0887E-40 | up |
| MELO3C021486.2 | 13 | 46 | 1.797 | 0.000308453 | up |
| MELO3C021488.2 | 242 | 112 | -1.117 | 3.36911E-08 | down |
| MELO3C021491.2 | 529 | 26 | -4.313 | 1.43581E-65 | down |
| MELO3C021499.2 | 47 | 14 | -1.782 | 0.000155946 | down |
| MELO3C021510.2 | 5117 | 2251 | -1.185 | 9.01964E-21 | down |
| MELO3C021528.2 | 1080 | 153 | -2.82 | 1.82644E-117 | down |
| MELO3C021532.2 | 375 | 805 | 1.102 | 1.38943E-17 | up |
| MELO3C021535.2 | 1437 | 623 | -1.205 | 5.16438E-40 | down |
| MELO3C021542.2 | 148 | 718 | 2.28 | 1.9847E-41 | up |
| MELO3C021544.2 | 27 | 83 | 1.62 | 3.67377E-06 | up |
| MELO3C021551.2 | 9 | 1 | -3.963 | 0.014005699 | down |
| MELO3C021553.2 | 1725 | 109 | -3.993 | 2.90339E-48 | down |
| MELO3C021558.2 | 23 | 6 | -1.769 | 0.0160661 | down |
| MELO3C021559.2 | 5 | 0 | -4.776 | 0.017634197 | down |
| MELO3C021566.2 | 133 | 394 | 1.568 | 6.62768E-11 | up |
| MELO3C021569.2 | 1048 | 444 | -1.239 | 4.87564E-35 | down |
| MELO3C021570.2 | 100 | 34 | -1.555 | 2.96887E-07 | down |
| MELO3C021579.2 | 13 | 95 | 2.925 | 1.48823E-11 | up |
| MELO3C021586.2 | 1156 | 544 | -1.087 | 1.43499E-16 | down |
| MELO3C021597.2 | 121 | 450 | 1.896 | 5.17755E-15 | up |
| MELO3C021603.2 | 1226 | 560 | -1.128 | 4.81452E-30 | down |
| MELO3C021604.2 | 171 | 511 | 1.582 | 2.8997E-13 | up |
| MELO3C021609.2 | 15 | 185 | 3.621 | 1.77594E-29 | up |
| MELO3C021613.2 | 367 | 170 | -1.106 | 2.77913E-09 | down |
| MELO3C021616.2 | 5253 | 1456 | -1.851 | 2.18898E-39 | down |
| MELO3C021629.2 | 1231 | 610 | -1.013 | 1.23829E-13 | down |
| MELO3C021641.2 | 134 | 60 | -1.165 | 3.26177E-06 | down |
| MELO3C021648.2 | 21925 | 6497 | -1.755 | 4.04254E-87 | down |
| MELO3C021658.2 | 25370 | 6280 | -2.014 | 2.98531E-58 | down |
| MELO3C021663.2 | 183 | 417 | 1.187 | 2.91935E-10 | up |
| MELO3C021681.2 | 1550 | 683 | -1.182 | 1.73027E-12 | down |
| MELO3C021682.2 | 468 | 1222 | 1.384 | 6.20017E-18 | up |
| MELO3C021688.2 | 4660 | 1791 | -1.379 | 3.86044E-14 | down |
| MELO3C021694.2 | 121 | 354 | 1.545 | 2.58262E-16 | up |
| MELO3C021703.2 | 60 | 436 | 2.858 | 1.39586E-24 | up |
| MELO3C021708.2 | 5281 | 504 | -3.389 | 3.45076E-231 | down |
| MELO3C021727.2 | 696 | 4285 | 2.624 | 1.98167E-79 | up |
| MELO3C021744.2 | 528 | 168 | -1.655 | 3.14424E-12 | down |
| MELO3C021748.2 | 0 | 7 | 5.211 | 0.003279508 | up |
| MELO3C021760.2 | 2386 | 70 | -5.085 | 5.44037E-18 | down |
| MELO3C021765.2 | 235 | 543 | 1.208 | 6.27238E-19 | up |
| MELO3C021766.2 | 6111 | 551 | -3.473 | 1.55882E-13 | down |
| MELO3C021767.2 | 3803 | 706 | -2.43 | 1.53574E-27 | down |
| MELO3C021768.2 | 182 | 31 | -2.534 | 3.56687E-17 | down |
| MELO3C021788.2 | 21403 | 1106 | -4.274 | 1.22599E-121 | down |
| MELO3C021789.2 | 21 | 7 | -1.665 | 0.028012369 | down |
| MELO3C021806.2 | 3218 | 1591 | -1.016 | 1.42906E-31 | down |
| MELO3C021807.2 | 893 | 216 | -2.049 | 2.67298E-28 | down |
| MELO3C021811.2 | 690 | 264 | -1.386 | 3.41585E-11 | down |
| MELO3C021818.2 | 6732 | 2397 | -1.49 | 5.14526E-40 | down |
| MELO3C021820.2 | 42 | 885 | 4.389 | 3.50846E-110 | up |
| MELO3C021823.2 | 130 | 7785 | 5.908 | 1.82393E-152 | up |
| MELO3C021843.2 | 223 | 56 | -2.012 | 3.50164E-08 | down |
| MELO3C021846.2 | 227 | 817 | 1.847 | 4.84803E-33 | up |
| MELO3C021851.2 | 961 | 2 | -8.608 | 7.00354E-60 | down |
| MELO3C021853.2 | 392 | 75 | -2.384 | 1.83491E-17 | down |
| MELO3C021857.2 | 1212 | 576 | -1.073 | 9.79948E-12 | down |
| MELO3C021871.2 | 15 | 1 | -3.607 | 0.001666587 | down |
| MELO3C021877.2 | 159 | 0 | -9.863 | 4.48856E-16 | down |
| MELO3C021880.2 | 1893 | 918 | -1.044 | 2.00907E-24 | down |
| MELO3C021884.2 | 537 | 2995 | 2.481 | 7.59444E-62 | up |
| MELO3C021886.2 | 1545 | 3888 | 1.331 | 2.07764E-20 | up |
| MELO3C021896.2 | 22 | 0 | -7.003 | 5.19957E-07 | down |
| MELO3C021901.2 | 12 | 2 | -2.675 | 0.007148736 | down |
| MELO3C021904.2 | 5 | 50 | 3.21 | 4.0825E-06 | up |
| MELO3C021912.2 | 64 | 239 | 1.898 | 3.69929E-09 | up |
| MELO3C021914.2 | 201 | 9 | -4.391 | 5.11434E-16 | down |
| MELO3C021919.2 | 843 | 2809 | 1.737 | 1.94227E-87 | up |
| MELO3C021920.2 | 99 | 42 | -1.235 | 0.000136245 | down |
| MELO3C021922.2 | 53 | 25 | -1.122 | 0.002858273 | down |
| MELO3C021923.2 | 4021 | 442 | -3.183 | 1.31872E-80 | down |
| MELO3C021934.2 | 696 | 2688 | 1.95 | 4.9324E-18 | up |
| MELO3C021940.2 | 4353 | 2127 | -1.033 | 1.19285E-31 | down |
| MELO3C021941.2 | 5460 | 14156 | 1.375 | 1.63444E-14 | up |
| MELO3C021948.2 | 1 | 22 | 3.919 | 0.000154435 | up |
| MELO3C021949.2 | 0 | 5 | 4.678 | 0.018659052 | up |
| MELO3C021951.2 | 70 | 0 | -8.68 | 1.96831E-11 | down |
| MELO3C021952.2 | 745 | 2582 | 1.793 | 3.71119E-32 | up |
| MELO3C021955.2 | 115 | 4 | -4.945 | 3.76282E-20 | down |
| MELO3C021959.2 | 638 | 239 | -1.413 | 2.05108E-15 | down |
| MELO3C021971.2 | 1427 | 394 | -1.856 | 1.27775E-67 | down |
| MELO3C021972.2 | 4539 | 1949 | -1.219 | 1.10478E-24 | down |
| MELO3C021980.2 | 1019 | 0 | -11.584 | 1.82704E-22 | down |
| MELO3C021987.2 | 3822 | 10257 | 1.424 | 1.2202E-59 | up |
| MELO3C021988.2 | 5867 | 1401 | -2.068 | 3.53751E-87 | down |
| MELO3C021993.2 | 335 | 28 | -3.585 | 9.42147E-06 | down |
| MELO3C021998.2 | 785 | 173 | -2.182 | 8.24159E-11 | down |
| MELO3C021999.2 | 5867 | 35391 | 2.593 | 5.84781E-229 | up |
| MELO3C022002.2 | 30 | 3 | -3.446 | 1.37823E-06 | down |
| MELO3C022005.2 | 1074 | 312 | -1.782 | 1.52622E-22 | down |
| MELO3C022007.2 | 18 | 100 | 2.48 | 2.50441E-12 | up |
| MELO3C022015.2 | 954 | 438 | -1.123 | 4.78446E-14 | down |
| MELO3C022018.2 | 2298 | 1002 | -1.198 | 3.65489E-09 | down |
| MELO3C022020.2 | 7523 | 17950 | 1.255 | 2.03483E-29 | up |
| MELO3C022021.2 | 3476 | 1721 | -1.015 | 2.78957E-17 | down |
| MELO3C022023.2 | 2285 | 1138 | -1.007 | 1.86617E-29 | down |
| MELO3C022027.2 | 1250 | 2843 | 1.185 | 2.73992E-25 | up |
| MELO3C022028.2 | 0 | 18 | 6.51 | 9.16245E-06 | up |
| MELO3C022029.2 | 51 | 128 | 1.326 | 3.11063E-06 | up |
| MELO3C022034.2 | 46 | 104 | 1.198 | 4.71281E-05 | up |
| MELO3C022037.2 | 196 | 1403 | 2.837 | 9.10729E-100 | up |
| MELO3C022039.2 | 0 | 48 | 7.925 | 1.97339E-09 | up |
| MELO3C022040.2 | 502 | 220 | -1.19 | 7.15943E-07 | down |
| MELO3C022041.2 | 229 | 9 | -4.587 | 8.17359E-39 | down |
| MELO3C022044.2 | 1656 | 674 | -1.299 | 2.13899E-14 | down |
| MELO3C022045.2 | 2559 | 7671 | 1.584 | 5.72384E-05 | up |
| MELO3C022057.2 | 52 | 107 | 1.04 | 0.002825878 | up |
| MELO3C022064.2 | 57 | 9 | -2.693 | 8.41279E-08 | down |
| MELO3C022068.2 | 475 | 953 | 1.005 | 2.33597E-18 | up |
| MELO3C022070.2 | 67 | 24 | -1.48 | 0.00038209 | down |
| MELO3C022075.2 | 315 | 643 | 1.029 | 6.00882E-14 | up |
| MELO3C022076.2 | 321 | 56 | -2.519 | 2.18011E-35 | down |
| MELO3C022077.2 | 1325 | 622 | -1.092 | 4.48296E-23 | down |
| MELO3C022087.2 | 0 | 5 | 4.509 | 0.029327769 | up |
| MELO3C022088.2 | 2596 | 1008 | -1.366 | 3.28463E-17 | down |
| MELO3C022090.2 | 1971 | 641 | -1.619 | 9.30714E-25 | down |
| MELO3C022095.2 | 1778 | 5559 | 1.645 | 1.76541E-57 | up |
| MELO3C022099.2 | 1184 | 549 | -1.109 | 1.438E-24 | down |
| MELO3C022108.2 | 8 | 138 | 4.14 | 9.31706E-19 | up |
| MELO3C022113.2 | 26009 | 266 | -6.611 | 0 | down |
| MELO3C022116.2 | 252 | 96 | -1.391 | 6.99937E-09 | down |
| MELO3C022120.2 | 114 | 841 | 2.884 | 1.2948E-32 | up |
| MELO3C022123.2 | 35 | 390 | 3.488 | 4.27592E-26 | up |
| MELO3C022126.2 | 587 | 226 | -1.38 | 3.45001E-21 | down |
| MELO3C022140.2 | 1921 | 530 | -1.86 | 2.10975E-33 | down |
| MELO3C022141.2 | 163 | 52 | -1.655 | 7.10864E-08 | down |
| MELO3C022146.2 | 88 | 21 | -2.077 | 8.10881E-06 | down |
| MELO3C022148.2 | 116 | 475 | 2.028 | 6.80888E-16 | up |
| MELO3C022150.2 | 1 | 72 | 6.656 | 1.18989E-09 | up |
| MELO3C022152.2 | 0 | 382 | 10.918 | 4.99099E-20 | up |
| MELO3C022157.2 | 0 | 5 | 4.533 | 0.021389783 | up |
| MELO3C022162.2 | 19066 | 433 | -5.459 | 6.98078E-42 | down |
| MELO3C022176.2 | 1007 | 26681 | 4.728 | 0 | up |
| MELO3C022180.2 | 839 | 368 | -1.187 | 1.51651E-06 | down |
| MELO3C022189.2 | 7 | 29 | 1.957 | 0.003604402 | up |
| MELO3C022193.2 | 2155 | 485 | -2.152 | 3.30831E-27 | down |
| MELO3C022202.2 | 613 | 18 | -5.054 | 7.57555E-99 | down |
| MELO3C022206.2 | 23 | 248 | 3.435 | 7.65357E-21 | up |
| MELO3C022207.2 | 6 | 89 | 3.794 | 4.42933E-16 | up |
| MELO3C022214.2 | 459 | 1002 | 1.127 | 1.05032E-25 | up |
| MELO3C022227.2 | 5032 | 2293 | -1.134 | 1.54414E-39 | down |
| MELO3C022228.2 | 3983 | 744 | -2.421 | 5.43417E-89 | down |
| MELO3C022231.2 | 2957 | 1450 | -1.028 | 2.59408E-29 | down |
| MELO3C022232.2 | 2472 | 1168 | -1.081 | 1.36164E-27 | down |
| MELO3C022233.2 | 1829 | 741 | -1.303 | 2.27021E-08 | down |
| MELO3C022236.2 | 2250 | 883 | -1.349 | 1.80601E-38 | down |
| MELO3C022240.2 | 876 | 0 | -12.328 | 2.5059E-25 | down |
| MELO3C022242.2 | 3807 | 1811 | -1.072 | 2.40983E-25 | down |
| MELO3C022246.2 | 12293 | 151 | -6.34 | 4.12368E-204 | down |
| MELO3C022249.2 | 898 | 193 | -2.213 | 4.22496E-25 | down |
| MELO3C022251.2 | 25 | 6 | -1.981 | 0.00342545 | down |
| MELO3C022252.2 | 501 | 171 | -1.554 | 2.04702E-12 | down |
| MELO3C022253.2 | 665 | 256 | -1.38 | 2.23556E-07 | down |
| MELO3C022259.2 | 523 | 129 | -2.018 | 2.17518E-08 | down |
| MELO3C022264.2 | 2 | 154 | 6.415 | 5.73961E-06 | up |
| MELO3C022268.2 | 24 | 90 | 1.915 | 5.68728E-08 | up |
| MELO3C022278.2 | 2047 | 5559 | 1.442 | 1.933E-61 | up |
| MELO3C022279.2 | 327 | 681 | 1.057 | 2.54384E-16 | up |
| MELO3C022281.2 | 0 | 9 | 5.441 | 0.001497204 | up |
| MELO3C022287.2 | 6 | 87 | 3.758 | 1.01233E-13 | up |
| MELO3C022291.2 | 608 | 0 | -11.8 | 8.09131E-23 | down |
| MELO3C022295.2 | 189 | 1064 | 2.495 | 1.68475E-26 | up |
| MELO3C022299.2 | 848 | 264 | -1.684 | 2.58696E-37 | down |
| MELO3C022300.2 | 86 | 14 | -2.603 | 0.000567067 | down |
| MELO3C022303.2 | 6017 | 2061 | -1.546 | 6.46394E-29 | down |
| MELO3C022310.2 | 6359 | 2417 | -1.396 | 1.74205E-36 | down |
| MELO3C022315.2 | 263 | 851 | 1.697 | 2.22854E-09 | up |
| MELO3C022316.2 | 64 | 3 | -4.485 | 1.19002E-09 | down |
| MELO3C022319.2 | 62 | 305 | 2.293 | 2.88923E-18 | up |
| MELO3C022321.2 | 3225 | 1592 | -1.018 | 7.59696E-39 | down |
| MELO3C022327.2 | 18 | 75 | 2 | 0.004115183 | up |
| MELO3C022334.2 | 1216 | 528 | -1.206 | 1.80238E-17 | down |
| MELO3C022335.2 | 3643 | 1752 | -1.056 | 3.7135E-18 | down |
| MELO3C022338.2 | 24 | 66 | 1.422 | 0.000689726 | up |
| MELO3C022342.2 | 7353 | 2329 | -1.659 | 7.44075E-44 | down |
| MELO3C022345.2 | 943 | 175 | -2.429 | 4.64857E-50 | down |
| MELO3C022348.2 | 7 | 31 | 2.134 | 0.006326307 | up |
| MELO3C022352.2 | 2459 | 1136 | -1.113 | 6.08436E-10 | down |
| MELO3C022354.2 | 4475 | 213 | -4.39 | 0 | down |
| MELO3C022355.2 | 1193 | 287 | -2.06 | 6.48359E-84 | down |
| MELO3C022356.2 | 5831 | 2406 | -1.278 | 2.49966E-33 | down |
| MELO3C022362.2 | 7 | 39 | 2.444 | 6.95274E-06 | up |
| MELO3C022371.2 | 49 | 102 | 1.045 | 0.004955258 | up |
| MELO3C022391.2 | 73 | 320 | 2.138 | 6.68371E-28 | up |
| MELO3C022393.2 | 1968 | 909 | -1.114 | 4.17247E-07 | down |
| MELO3C022397.2 | 12 | 1261 | 6.784 | 1.68736E-121 | up |
| MELO3C022404.2 | 38 | 78 | 1.026 | 0.001711531 | up |
| MELO3C022409.2 | 169 | 357 | 1.08 | 4.50274E-12 | up |
| MELO3C022410.2 | 324 | 148 | -1.136 | 7.11649E-08 | down |
| MELO3C022412.2 | 162 | 433 | 1.416 | 1.19332E-18 | up |
| MELO3C022417.2 | 307 | 65 | -2.23 | 2.46721E-16 | down |
| MELO3C022429.2 | 24 | 344 | 3.857 | 9.86314E-07 | up |
| MELO3C022430.2 | 140 | 2773 | 4.306 | 0.006662862 | up |
| MELO3C022445.2 | 9717 | 4754 | -1.032 | 9.20395E-28 | down |
| MELO3C022447.2 | 31 | 269 | 3.14 | 7.13709E-29 | up |
| MELO3C022449.2 | 284 | 969 | 1.772 | 4.98276E-16 | up |
| MELO3C022458.2 | 0 | 7 | 4.169 | 0.012350528 | up |
| MELO3C022459.2 | 35 | 160 | 2.193 | 2.1934E-13 | up |
| MELO3C022464.2 | 48 | 11 | -2.15 | 0.010862362 | down |
| MELO3C022468.2 | 6 | 27 | 2.255 | 0.006490024 | up |
| MELO3C022470.2 | 0 | 67 | 8.405 | 7.14101E-11 | up |
| MELO3C022473.2 | 6 | 19 | 1.671 | 0.016877379 | up |
| MELO3C022483.2 | 1070 | 251 | -2.091 | 4.0809E-36 | down |
| MELO3C022485.2 | 3801 | 1275 | -1.576 | 4.79843E-26 | down |
| MELO3C022487.2 | 35 | 12 | -1.504 | 0.001952339 | down |
| MELO3C022499.2 | 2313 | 709 | -1.706 | 1.44147E-46 | down |
| MELO3C022501.2 | 0 | 121 | 9.255 | 4.33367E-14 | up |
| MELO3C022507.2 | 163 | 1291 | 2.991 | 2.38641E-71 | up |
| MELO3C022513.2 | 103 | 32 | -1.699 | 3.74925E-06 | down |
| MELO3C022516.2 | 22 | 4 | -2.569 | 0.000878062 | down |
| MELO3C022517.2 | 1651 | 4655 | 1.495 | 1.34377E-57 | up |
| MELO3C022518.2 | 483 | 36 | -3.713 | 4.96277E-29 | down |
| MELO3C022520.2 | 328 | 164 | -1.001 | 9.0762E-11 | down |
| MELO3C022524.2 | 165 | 347 | 1.068 | 2.72545E-08 | up |
| MELO3C022542.2 | 188 | 10 | -4.176 | 5.6579E-31 | down |
| MELO3C022568.2 | 671 | 56 | -3.566 | 1.028E-74 | down |
| MELO3C022590.2 | 3 | 61 | 4.407 | 4.7025E-10 | up |
| MELO3C022596.2 | 27 | 86 | 1.704 | 2.8955E-05 | up |
| MELO3C022601.2 | 80 | 8 | -3.386 | 1.34301E-11 | down |
| MELO3C022602.2 | 262 | 100 | -1.381 | 3.68506E-11 | down |
| MELO3C022620.2 | 87 | 28 | -1.626 | 7.49614E-06 | down |
| MELO3C022631.2 | 557 | 0 | -11.675 | 1.29198E-22 | down |
| MELO3C022641.2 | 50 | 25 | -1.051 | 0.025685804 | down |
| MELO3C022646.2 | 142 | 0 | -9.709 | 1.44791E-15 | down |
| MELO3C022669.2 | 26 | 8 | -1.772 | 0.003852959 | down |
| MELO3C022671.2 | 8 | 152 | 4.147 | 3.39625E-18 | up |
| MELO3C022688.2 | 124 | 32 | -1.966 | 3.70571E-11 | down |
| MELO3C022727.2 | 30 | 170 | 2.488 | 6.83696E-11 | up |
| MELO3C022731.2 | 2 | 55 | 4.67 | 8.90609E-10 | up |
| MELO3C022734.2 | 1045 | 90 | -3.523 | 6.98074E-37 | down |
| MELO3C022736.2 | 56 | 250 | 2.152 | 5.84301E-23 | up |
| MELO3C022738.2 | 282 | 2076 | 2.882 | 1.54543E-64 | up |
| MELO3C022746.2 | 5819 | 1269 | -2.197 | 1.12231E-96 | down |
| MELO3C022748.2 | 30 | 6 | -2.18 | 0.002325439 | down |
| MELO3C022753.2 | 2769 | 9332 | 1.753 | 2.33417E-35 | up |
| MELO3C022765.2 | 27 | 57 | 1.102 | 0.003247227 | up |
| MELO3C022773.2 | 298 | 29 | -3.346 | 9.79875E-25 | down |
| MELO3C022802.2 | 205 | 56 | -1.875 | 1.83635E-07 | down |
| MELO3C022804.2 | 177 | 77 | -1.188 | 0.000923492 | down |
| MELO3C022817.2 | 96 | 37 | -1.374 | 0.000350357 | down |
| MELO3C022819.2 | 786 | 1851 | 1.237 | 2.61498E-19 | up |
| MELO3C022820.2 | 295 | 68 | -2.126 | 8.92615E-17 | down |
| MELO3C022828.2 | 7558 | 181 | -5.378 | 4.40611E-212 | down |
| MELO3C022834.2 | 980 | 270 | -1.862 | 1.15055E-62 | down |
| MELO3C022835.2 | 0 | 6 | 4.805 | 0.009350471 | up |
| MELO3C022837.2 | 8 | 45 | 2.472 | 0.001006042 | up |
| MELO3C022843.2 | 0 | 17 | 6.429 | 1.08695E-05 | up |
| MELO3C022844.2 | 0 | 92 | 8.863 | 6.76515E-13 | up |
| MELO3C022852.2 | 158 | 386 | 1.286 | 6.89747E-12 | up |
| MELO3C022910.2 | 117 | 285 | 1.288 | 5.31545E-09 | up |
| MELO3C022917.2 | 268 | 1376 | 2.36 | 1.06294E-30 | up |
| MELO3C022935.2 | 54 | 3 | -4.315 | 6.10341E-09 | down |
| MELO3C022936.2 | 1359 | 622 | -1.129 | 3.02632E-24 | down |
| MELO3C022954.2 | 18 | 61 | 1.773 | 0.000496581 | up |
| MELO3C022955.2 | 49 | 1 | -6.3 | 2.17358E-08 | down |
| MELO3C022961.2 | 2994 | 1436 | -1.061 | 8.75602E-15 | down |
| MELO3C022970.2 | 2958 | 1264 | -1.227 | 3.71376E-18 | down |
| MELO3C022978.2 | 218 | 1778 | 3.031 | 7.3411E-30 | up |
| MELO3C022979.2 | 118 | 293 | 1.315 | 1.12583E-11 | up |
| MELO3C022991.2 | 4 | 2108 | 9.199 | 1.10686E-31 | up |
| MELO3C022997.2 | 61 | 137 | 1.171 | 0.004568498 | up |
| MELO3C023001.2 | 3565 | 9154 | 1.361 | 4.79205E-50 | up |
| MELO3C023002.2 | 0 | 6 | 4.957 | 0.009506846 | up |
| MELO3C023008.2 | 27 | 6 | -2.173 | 0.004297097 | down |
| MELO3C023009.2 | 1736 | 3559 | 1.036 | 2.46381E-33 | up |
| MELO3C023011.2 | 105 | 238 | 1.187 | 1.85656E-09 | up |
| MELO3C023017.2 | 188 | 575 | 1.609 | 1.21383E-19 | up |
| MELO3C023022.2 | 717 | 1785 | 1.317 | 1.12209E-18 | up |
| MELO3C023027.2 | 7 | 357 | 5.564 | 1.28081E-05 | up |
| MELO3C023031.2 | 0 | 5 | 4.699 | 0.014441696 | up |
| MELO3C023032.2 | 536 | 48 | -3.498 | 1.62993E-45 | down |
| MELO3C023041.2 | 596 | 32 | -4.231 | 1.5219E-72 | down |
| MELO3C023043.2 | 3 | 29 | 3.338 | 0.000589867 | up |
| MELO3C023045.2 | 204 | 36 | -2.489 | 6.74343E-12 | down |
| MELO3C023047.2 | 414 | 963 | 1.218 | 1.67739E-15 | up |
| MELO3C023065.2 | 33 | 490 | 3.879 | 2.6419E-58 | up |
| MELO3C023067.2 | 2706 | 286 | -3.242 | 3.65946E-118 | down |
| MELO3C023072.2 | 1290 | 539 | -1.259 | 3.70599E-13 | down |
| MELO3C023086.2 | 8997 | 1084 | -3.053 | 6.04622E-93 | down |
| MELO3C023087.2 | 52 | 6 | -3.026 | 5.26853E-07 | down |
| MELO3C023093.2 | 0 | 28 | 7.141 | 3.63315E-07 | up |
| MELO3C023114.2 | 1949 | 7484 | 1.941 | 5.81501E-24 | up |
| MELO3C023118.2 | 0 | 18 | 6.539 | 3.98318E-06 | up |
| MELO3C023128.2 | 70 | 259 | 1.876 | 5.52294E-13 | up |
| MELO3C023131.2 | 29619 | 6103 | -2.279 | 5.09929E-44 | down |
| MELO3C023156.2 | 1 | 11 | 3.414 | 0.004654828 | up |
| MELO3C023166.2 | 443 | 58 | -2.931 | 2.81636E-22 | down |
| MELO3C023173.2 | 19 | 89 | 2.198 | 7.78284E-10 | up |
| MELO3C023180.2 | 5 | 176 | 5.034 | 1.74154E-22 | up |
| MELO3C023188.2 | 604 | 2608 | 2.111 | 2.64839E-97 | up |
| MELO3C023190.2 | 6716 | 2496 | -1.428 | 8.13758E-26 | down |
| MELO3C023192.2 | 22 | 64 | 1.567 | 7.17685E-05 | up |
| MELO3C023197.2 | 1390 | 590 | -1.238 | 5.91392E-20 | down |
| MELO3C023200.2 | 745 | 190 | -1.965 | 4.23595E-28 | down |
| MELO3C023203.2 | 77 | 454 | 2.56 | 0.001950997 | up |
| MELO3C023204.2 | 16 | 83 | 2.337 | 1.72864E-07 | up |
| MELO3C023220.2 | 338 | 3247 | 3.265 | 3.19829E-39 | up |
| MELO3C023230.2 | 32 | 2 | -4.089 | 1.03479E-06 | down |
| MELO3C023232.2 | 564 | 225 | -1.33 | 8.52779E-16 | down |
| MELO3C023234.2 | 274 | 60 | -2.196 | 6.25169E-12 | down |
| MELO3C023240.2 | 19 | 44 | 1.197 | 0.010055325 | up |
| MELO3C023241.2 | 15 | 167 | 3.505 | 2.46491E-24 | up |
| MELO3C023245.2 | 23 | 2 | -3.646 | 6.96726E-05 | down |
| MELO3C023251.2 | 449 | 1038 | 1.211 | 2.40073E-21 | up |
| MELO3C023252.2 | 98 | 329 | 1.759 | 3.28264E-20 | up |
| MELO3C023253.2 | 43 | 673 | 3.977 | 2.16239E-27 | up |
| MELO3C023255.2 | 1573 | 141 | -3.476 | 1.87966E-42 | down |
| MELO3C023263.2 | 41 | 12 | -1.747 | 0.006883171 | down |
| MELO3C023268.2 | 338 | 166 | -1.027 | 4.46856E-07 | down |
| MELO3C023270.2 | 123 | 50 | -1.295 | 0.000464163 | down |
| MELO3C023274.2 | 287 | 119 | -1.268 | 5.13569E-11 | down |
| MELO3C023285.2 | 0 | 7 | 5.107 | 0.003391356 | up |
| MELO3C023286.2 | 1 | 25 | 5.125 | 3.63724E-05 | up |
| MELO3C023304.2 | 258 | 715 | 1.472 | 3.77699E-10 | up |
| MELO3C023308.2 | 3584 | 1545 | -1.213 | 7.619E-34 | down |
| MELO3C023311.2 | 403 | 38 | -3.411 | 7.15864E-25 | down |
| MELO3C023313.2 | 378 | 889 | 1.234 | 1.99931E-06 | up |
| MELO3C023316.2 | 0 | 31 | 7.293 | 4.21378E-08 | up |
| MELO3C023318.2 | 3034 | 1385 | -1.131 | 9.25186E-34 | down |
| MELO3C023320.2 | 0 | 89 | 8.825 | 1.44844E-11 | up |
| MELO3C023335.2 | 7 | 51 | 2.823 | 3.91923E-08 | up |
| MELO3C023342.2 | 343 | 1630 | 2.251 | 4.73593E-23 | up |
| MELO3C023349.2 | 19 | 43 | 1.209 | 0.003762635 | up |
| MELO3C023354.2 | 67514 | 18851 | -1.841 | 8.75337E-74 | down |
| MELO3C023361.2 | 8 | 72 | 3.187 | 1.93736E-05 | up |
| MELO3C023377.2 | 0 | 16 | 6.377 | 1.08501E-05 | up |
| MELO3C023385.2 | 567 | 1648 | 1.54 | 9.97482E-23 | up |
| MELO3C023391.2 | 962 | 2007 | 1.061 | 7.67896E-13 | up |
| MELO3C023394.2 | 1127 | 227 | -2.314 | 4.7188E-21 | down |
| MELO3C023407.2 | 3 | 126 | 5.649 | 2.14388E-18 | up |
| MELO3C023408.2 | 2915 | 373 | -2.967 | 6.54065E-96 | down |
| MELO3C023420.2 | 321 | 105 | -1.614 | 0.000113941 | down |
| MELO3C023425.2 | 933 | 4555 | 2.288 | 1.42047E-70 | up |
| MELO3C023426.2 | 923 | 2231 | 1.274 | 2.95105E-23 | up |
| MELO3C023431.2 | 3261 | 324 | -3.332 | 9.4155E-96 | down |
| MELO3C023440.2 | 78 | 0 | -8.832 | 1.09205E-12 | down |
| MELO3C023442.2 | 1999 | 646 | -1.631 | 2.04365E-49 | down |
| MELO3C023447.2 | 14 | 3 | -2.507 | 0.003740022 | down |
| MELO3C023448.2 | 28 | 58 | 1.051 | 0.011057134 | up |
| MELO3C023460.2 | 23 | 63 | 1.451 | 0.000129293 | up |
| MELO3C023461.2 | 435 | 133 | -1.707 | 3.56115E-23 | down |
| MELO3C023465.2 | 26415 | 2990 | -3.143 | 8.22106E-16 | down |
| MELO3C023468.2 | 3829 | 1149 | -1.737 | 2.59472E-59 | down |
| MELO3C023470.2 | 183 | 41 | -2.148 | 2.07526E-13 | down |
| MELO3C023473.2 | 4475 | 1661 | -1.43 | 1.9148E-46 | down |
| MELO3C023478.2 | 89 | 238 | 1.42 | 4.7552E-11 | up |
| MELO3C023479.2 | 798 | 342 | -1.223 | 7.83301E-27 | down |
| MELO3C023483.2 | 9 | 0 | -5.718 | 0.000761827 | down |
| MELO3C023485.2 | 9 | 68 | 2.983 | 8.23778E-10 | up |
| MELO3C023490.2 | 370 | 3268 | 3.142 | 5.46484E-160 | up |
| MELO3C023495.2 | 92 | 224 | 1.277 | 6.09668E-06 | up |
| MELO3C023496.2 | 71 | 610 | 3.099 | 5.75253E-73 | up |
| MELO3C023500.2 | 0 | 7 | 5.184 | 0.025733441 | up |
| MELO3C023503.2 | 47 | 8 | -2.601 | 9.32863E-06 | down |
| MELO3C023505.2 | 519 | 1135 | 1.129 | 9.60101E-31 | up |
| MELO3C023521.2 | 106 | 263 | 1.306 | 1.45286E-08 | up |
| MELO3C023522.2 | 52 | 5 | -3.593 | 1.54667E-09 | down |
| MELO3C023523.2 | 1318 | 579 | -1.188 | 1.30682E-11 | down |
| MELO3C023527.2 | 133 | 1335 | 3.325 | 1.89194E-36 | up |
| MELO3C023539.2 | 123 | 19 | -2.724 | 4.40613E-12 | down |
| MELO3C023540.2 | 29 | 5 | -2.561 | 0.009749586 | down |
| MELO3C023542.2 | 536 | 114 | -2.232 | 1.85946E-29 | down |
| MELO3C023545.2 | 275 | 575 | 1.061 | 3.77955E-11 | up |
| MELO3C023546.2 | 3 | 18 | 2.668 | 0.001938114 | up |
| MELO3C023550.2 | 132 | 287 | 1.126 | 9.01497E-09 | up |
| MELO3C023555.2 | 17153 | 7164 | -1.26 | 4.82592E-23 | down |
| MELO3C023563.2 | 288 | 61 | -2.238 | 2.09567E-21 | down |
| MELO3C023566.2 | 1 | 267 | 8.541 | 3.31978E-16 | up |
| MELO3C023567.2 | 0 | 24 | 6.905 | 5.60807E-07 | up |
| MELO3C023568.2 | 242 | 84 | -1.529 | 1.97145E-10 | down |
| MELO3C023570.2 | 247 | 1279 | 2.374 | 6.2926E-38 | up |
| MELO3C023571.2 | 344 | 745 | 1.115 | 1.34094E-18 | up |
| MELO3C023578.2 | 45 | 274 | 2.611 | 5.86416E-22 | up |
| MELO3C023579.2 | 35 | 0 | -7.667 | 8.1633E-09 | down |
| MELO3C023581.2 | 7050 | 15663 | 1.152 | 1.47001E-43 | up |
| MELO3C023586.2 | 870 | 3 | -8.296 | 2.5421E-61 | down |
| MELO3C023587.2 | 86 | 19 | -2.137 | 4.7622E-06 | down |
| MELO3C023589.2 | 11 | 26 | 1.273 | 0.018973732 | up |
| MELO3C023591.2 | 1 | 71 | 6.626 | 1.57075E-09 | up |
| MELO3C023593.2 | 6 | 69 | 3.503 | 4.67647E-11 | up |
| MELO3C023596.2 | 3588 | 800 | -2.165 | 3.01072E-35 | down |
| MELO3C023599.2 | 2070 | 993 | -1.061 | 1.14923E-10 | down |
| MELO3C023604.2 | 988 | 466 | -1.087 | 2.14848E-14 | down |
| MELO3C023605.2 | 2745 | 401 | -2.774 | 3.53885E-124 | down |
| MELO3C023606.2 | 16892 | 34683 | 1.038 | 1.10832E-31 | up |
| MELO3C023609.2 | 2972 | 739 | -2.006 | 7.41857E-100 | down |
| MELO3C023618.2 | 7 | 41 | 2.65 | 4.22418E-05 | up |
| MELO3C023620.2 | 16 | 102 | 2.695 | 1.17709E-10 | up |
| MELO3C023621.2 | 84 | 0 | -8.957 | 4.68899E-13 | down |
| MELO3C023630.2 | 5012 | 10457 | 1.061 | 2.21461E-35 | up |
| MELO3C023633.2 | 427 | 6 | -5.987 | 6.25588E-49 | down |
| MELO3C023654.2 | 20 | 164 | 3.088 | 7.24183E-23 | up |
| MELO3C023673.2 | 320 | 656 | 1.037 | 1.01234E-13 | up |
| MELO3C023678.2 | 753 | 1986 | 1.399 | 2.10963E-08 | up |
| MELO3C023682.2 | 2452 | 1089 | -1.171 | 1.32761E-17 | down |
| MELO3C023684.2 | 2752 | 1083 | -1.346 | 1.68569E-48 | down |
| MELO3C023694.2 | 9 | 47 | 2.397 | 0.00087652 | up |
| MELO3C023702.2 | 1164 | 496 | -1.228 | 3.62151E-10 | down |
| MELO3C023714.2 | 192 | 54 | -1.823 | 3.41015E-07 | down |
| MELO3C023723.2 | 50 | 3 | -4.02 | 1.24595E-08 | down |
| MELO3C023724.2 | 4151 | 2017 | -1.041 | 1.80201E-20 | down |
| MELO3C023727.2 | 37 | 126 | 1.765 | 5.98457E-08 | up |
| MELO3C023770.2 | 41 | 8 | -2.269 | 0.000250986 | down |
| MELO3C023777.2 | 280 | 773 | 1.467 | 1.31005E-19 | up |
| MELO3C023781.2 | 3048 | 177 | -4.114 | 1.33259E-79 | down |
| MELO3C023788.2 | 2277 | 822 | -1.471 | 3.04509E-27 | down |
| MELO3C023790.2 | 1133 | 141 | -3.009 | 6.8853E-105 | down |
| MELO3C023794.2 | 24 | 71 | 1.592 | 8.28144E-05 | up |
| MELO3C023799.2 | 0 | 29 | 7.187 | 6.84616E-08 | up |
| MELO3C023804.2 | 2356 | 4943 | 1.069 | 2.93787E-41 | up |
| MELO3C023810.2 | 44 | 17 | -1.392 | 0.000830878 | down |
| MELO3C023814.2 | 3 | 46 | 3.691 | 2.72193E-08 | up |
| MELO3C023822.2 | 161 | 802 | 2.314 | 4.52989E-50 | up |
| MELO3C023827.2 | 113 | 0 | -9.377 | 2.13586E-14 | down |
| MELO3C023833.2 | 68 | 8 | -3.012 | 8.24184E-08 | down |
| MELO3C023840.2 | 46 | 9 | -2.351 | 0.002399171 | down |
| MELO3C023842.2 | 252 | 607 | 1.272 | 1.9687E-06 | up |
| MELO3C023843.2 | 308 | 37 | -3.054 | 2.3395E-14 | down |
| MELO3C023847.2 | 129 | 32 | -2.034 | 0.002189544 | down |
| MELO3C023848.2 | 40 | 216 | 2.429 | 1.34317E-16 | up |
| MELO3C023849.2 | 0 | 119 | 9.236 | 3.86697E-14 | up |
| MELO3C023851.2 | 42 | 85 | 1.027 | 0.002174002 | up |
| MELO3C023852.2 | 369 | 14 | -4.668 | 9.70461E-39 | down |
| MELO3C023853.2 | 397 | 158 | -1.329 | 1.03079E-06 | down |
| MELO3C023855.2 | 61 | 156 | 1.354 | 7.23202E-07 | up |
| MELO3C023876.2 | 40 | 4 | -3.311 | 2.21234E-06 | down |
| MELO3C023879.2 | 54031 | 19687 | -1.457 | 5.65981E-38 | down |
| MELO3C023881.2 | 80 | 346 | 2.103 | 7.22329E-27 | up |
| MELO3C023896.2 | 496 | 112 | -2.146 | 3.81801E-16 | down |
| MELO3C023900.2 | 0 | 88 | 8.798 | 9.43029E-13 | up |
| MELO3C023917.2 | 0 | 13 | 6.107 | 0.000106609 | up |
| MELO3C023918.2 | 1 | 27 | 5.22 | 1.70976E-05 | up |
| MELO3C023928.2 | 73 | 12 | -2.629 | 1.27144E-08 | down |
| MELO3C023930.2 | 356 | 21 | -4.075 | 1.24698E-47 | down |
| MELO3C023931.2 | 0 | 845 | 12.063 | 2.49774E-24 | up |
| MELO3C023944.2 | 28 | 114 | 2.027 | 1.4163E-09 | up |
| MELO3C023956.2 | 13 | 33 | 1.361 | 0.006292571 | up |
| MELO3C023972.2 | 38 | 240 | 2.662 | 2.38226E-24 | up |
| MELO3C023976.2 | 37 | 3 | -3.916 | 4.37587E-08 | down |
| MELO3C023981.2 | 62 | 19 | -1.672 | 1.39223E-05 | down |
| MELO3C023982.2 | 1376 | 499 | -1.464 | 6.31186E-43 | down |
| MELO3C023986.2 | 370 | 2 | -7.644 | 2.74114E-35 | down |
| MELO3C023987.2 | 134 | 437 | 1.706 | 0.014407506 | up |
| MELO3C023997.2 | 9609 | 4163 | -1.207 | 1.48062E-30 | down |
| MELO3C024010.2 | 3787 | 848 | -2.159 | 8.11166E-33 | down |
| MELO3C024012.2 | 6 | 22 | 1.954 | 0.018936002 | up |
| MELO3C024016.2 | 1755 | 746 | -1.234 | 1.39006E-34 | down |
| MELO3C024030.2 | 2657 | 8440 | 1.668 | 8.16857E-34 | up |
| MELO3C024033.2 | 128 | 54 | -1.252 | 1.54557E-05 | down |
| MELO3C024035.2 | 11 | 26 | 1.276 | 0.024668819 | up |
| MELO3C024045.2 | 0 | 169 | 8.778 | 3.82096E-13 | up |
| MELO3C024061.2 | 4843 | 1506 | -1.686 | 8.46947E-21 | down |
| MELO3C024063.2 | 474 | 44 | -3.425 | 1.87546E-35 | down |
| MELO3C024066.2 | 0 | 5 | 4.728 | 0.011758431 | up |
| MELO3C024075.2 | 882 | 439 | -1.007 | 9.89963E-09 | down |
| MELO3C024080.2 | 355 | 3370 | 3.246 | 2.98454E-79 | up |
| MELO3C024086.2 | 6354 | 1647 | -1.949 | 2.62413E-68 | down |
| MELO3C024092.2 | 217 | 8 | -4.742 | 1.57098E-31 | down |
| MELO3C024100.2 | 12 | 0 | -6.114 | 7.56185E-05 | down |
| MELO3C024102.2 | 198 | 1147 | 2.531 | 4.84259E-29 | up |
| MELO3C024107.2 | 988 | 71 | -3.785 | 7.68984E-133 | down |
| MELO3C024108.2 | 1112 | 6683 | 2.587 | 1.16129E-191 | up |
| MELO3C024121.2 | 2820 | 562 | -2.327 | 1.34686E-47 | down |
| MELO3C024127.2 | 129 | 518 | 2.007 | 1.61931E-25 | up |
| MELO3C024143.2 | 8 | 44 | 2.487 | 0.00070556 | up |
| MELO3C024146.2 | 121 | 682 | 2.495 | 6.97918E-59 | up |
| MELO3C024152.2 | 5 | 21 | 1.977 | 0.007483273 | up |
| MELO3C024155.2 | 372 | 124 | -1.582 | 5.45105E-20 | down |
| MELO3C024167.2 | 662 | 1326 | 1.002 | 4.65196E-11 | up |
| MELO3C024168.2 | 57 | 149 | 1.388 | 3.56939E-07 | up |
| MELO3C024175.2 | 5130 | 1965 | -1.384 | 1.03478E-77 | down |
| MELO3C024176.2 | 24 | 90 | 1.915 | 4.67588E-07 | up |
| MELO3C024188.2 | 1853 | 862 | -1.105 | 4.81286E-17 | down |
| MELO3C024191.2 | 525 | 248 | -1.085 | 4.56177E-12 | down |
| MELO3C024192.2 | 2181 | 12430 | 2.511 | 7.94863E-39 | up |
| MELO3C024198.2 | 35 | 83 | 1.224 | 0.000134515 | up |
| MELO3C024199.2 | 14 | 44 | 1.617 | 0.000587905 | up |
| MELO3C024206.2 | 52061 | 5161 | -3.334 | 3.29898E-92 | down |
| MELO3C024213.2 | 250 | 781 | 1.643 | 5.18109E-34 | up |
| MELO3C024225.2 | 1294 | 3575 | 1.466 | 5.21487E-44 | up |
| MELO3C024228.2 | 1663 | 646 | -1.365 | 2.2491E-41 | down |
| MELO3C024231.2 | 66 | 342 | 2.369 | 4.91593E-21 | up |
| MELO3C024234.2 | 399 | 165 | -1.276 | 4.29749E-15 | down |
| MELO3C024235.2 | 51 | 7 | -2.775 | 6.0287E-08 | down |
| MELO3C024236.2 | 84 | 616 | 2.873 | 3.64676E-63 | up |
| MELO3C024238.2 | 2 | 23 | 3.439 | 0.000125148 | up |
| MELO3C024239.2 | 137 | 6 | -4.458 | 6.65261E-23 | down |
| MELO3C024241.2 | 54 | 965 | 4.158 | 6.08858E-76 | up |
| MELO3C024247.2 | 24 | 1584 | 6.067 | 7.55469E-167 | up |
| MELO3C024252.2 | 61 | 209 | 1.765 | 1.31887E-12 | up |
| MELO3C024255.2 | 2204 | 724 | -1.606 | 4.06193E-29 | down |
| MELO3C024256.2 | 67 | 245 | 1.875 | 0.000105903 | up |
| MELO3C024263.2 | 30 | 2 | -3.595 | 5.55688E-05 | down |
| MELO3C024264.2 | 242 | 83 | -1.55 | 0.000291194 | down |
| MELO3C024268.2 | 12 | 36 | 1.568 | 0.004582575 | up |
| MELO3C024278.2 | 285 | 730 | 1.356 | 1.83856E-27 | up |
| MELO3C024292.2 | 2121 | 6191 | 1.545 | 2.07949E-38 | up |
| MELO3C024303.2 | 375 | 1143 | 1.609 | 4.63638E-47 | up |
| MELO3C024304.2 | 1 | 9 | 3.66 | 0.016668968 | up |
| MELO3C024306.2 | 308 | 1351 | 2.133 | 0.000157728 | up |
| MELO3C024311.2 | 13 | 3 | -2.088 | 0.020603645 | down |
| MELO3C024312.2 | 152 | 60 | -1.325 | 4.88321E-08 | down |
| MELO3C024314.2 | 1123 | 2993 | 1.414 | 2.10681E-18 | up |
| MELO3C024317.2 | 3368 | 1291 | -1.382 | 7.46425E-19 | down |
| MELO3C024318.2 | 115 | 13 | -3.161 | 2.10092E-14 | down |
| MELO3C024323.2 | 133 | 52 | -1.347 | 0.002844595 | down |
| MELO3C024324.2 | 2227 | 344 | -2.695 | 4.82967E-85 | down |
| MELO3C024326.2 | 25202 | 10281 | -1.294 | 8.35126E-23 | down |
| MELO3C024337.2 | 766 | 4216 | 2.46 | 1.88731E-49 | up |
| MELO3C024339.2 | 14 | 2 | -2.474 | 0.018254012 | down |
| MELO3C024341.2 | 427 | 1458 | 1.772 | 5.04869E-18 | up |
| MELO3C024344.2 | 5868 | 1615 | -1.861 | 2.29871E-89 | down |
| MELO3C024346.2 | 4906 | 1897 | -1.371 | 3.46538E-28 | down |
| MELO3C024355.2 | 21 | 46 | 1.172 | 0.014477188 | up |
| MELO3C024371.2 | 46 | 144 | 1.649 | 5.47095E-10 | up |
| MELO3C024377.2 | 14 | 201 | 3.885 | 6.15349E-21 | up |
| MELO3C024378.2 | 2858 | 5777 | 1.015 | 1.10362E-10 | up |
| MELO3C024387.2 | 10 | 134 | 3.798 | 2.24298E-21 | up |
| MELO3C024388.2 | 464 | 1717 | 1.888 | 4.58077E-47 | up |
| MELO3C024390.2 | 2287 | 5674 | 1.311 | 1.50486E-47 | up |
| MELO3C024398.2 | 1115 | 263 | -2.089 | 1.34446E-29 | down |
| MELO3C024400.2 | 71 | 4 | -4.355 | 7.7178E-12 | down |
| MELO3C024412.2 | 32 | 296 | 3.229 | 4.78033E-28 | up |
| MELO3C024417.2 | 43 | 132 | 1.613 | 5.3679E-09 | up |
| MELO3C024420.2 | 5 | 1265 | 7.88 | 1.16971E-90 | up |
| MELO3C024422.2 | 14 | 44 | 1.613 | 0.001258449 | up |
| MELO3C024427.2 | 0 | 15 | 6.298 | 2.41295E-05 | up |
| MELO3C024430.2 | 2655 | 1225 | -1.116 | 4.3036E-18 | down |
| MELO3C024431.2 | 2820 | 729 | -1.952 | 5.72195E-78 | down |
| MELO3C024434.2 | 141 | 16 | -3.15 | 4.54594E-19 | down |
| MELO3C024436.2 | 1701 | 613 | -1.473 | 1.49746E-40 | down |
| MELO3C024437.2 | 994 | 2455 | 1.303 | 1.13482E-37 | up |
| MELO3C024439.2 | 11 | 28 | 1.379 | 0.023840979 | up |
| MELO3C024459.2 | 111 | 302 | 1.444 | 1.51103E-15 | up |
| MELO3C024463.2 | 451 | 129 | -1.819 | 4.22309E-23 | down |
| MELO3C024465.2 | 1355 | 628 | -1.11 | 1.97552E-22 | down |
| MELO3C024466.2 | 263 | 1843 | 2.811 | 1.53205E-05 | up |
| MELO3C024471.2 | 109 | 428 | 1.979 | 2.26524E-10 | up |
| MELO3C024477.2 | 19 | 46 | 1.271 | 0.008148252 | up |
| MELO3C024482.2 | 867 | 297 | -1.547 | 2.53821E-26 | down |
| MELO3C024486.2 | 900 | 181 | -2.311 | 1.8665E-56 | down |
| MELO3C024490.2 | 52 | 315 | 2.593 | 8.93425E-17 | up |
| MELO3C024493.2 | 0 | 11 | 5.792 | 0.000215816 | up |
| MELO3C024503.2 | 80 | 35 | -1.185 | 0.002015448 | down |
| MELO3C024508.2 | 160 | 1447 | 3.177 | 4.85024E-51 | up |
| MELO3C024513.2 | 15 | 1707 | 6.827 | 1.72959E-102 | up |
| MELO3C024516.2 | 1461 | 3361 | 1.202 | 5.15698E-35 | up |
| MELO3C024520.2 | 25 | 0 | -7.194 | 4.61363E-07 | down |
| MELO3C024523.2 | 14 | 2 | -2.74 | 0.007023861 | down |
| MELO3C024525.2 | 276 | 584 | 1.079 | 2.32527E-05 | up |
| MELO3C024529.2 | 20 | 127 | 2.663 | 5.19617E-11 | up |
| MELO3C024531.2 | 7499 | 2243 | -1.741 | 1.97422E-07 | down |
| MELO3C024532.2 | 810 | 150 | -2.432 | 5.15352E-17 | down |
| MELO3C024535.2 | 1 | 13 | 4.195 | 0.003948148 | up |
| MELO3C024539.2 | 92 | 1 | -6.238 | 3.34176E-14 | down |
| MELO3C024541.2 | 0 | 5 | 4.708 | 0.013245491 | up |
| MELO3C024549.2 | 428 | 1434 | 1.744 | 5.95281E-29 | up |
| MELO3C024550.2 | 4563 | 691 | -2.724 | 4.09091E-195 | down |
| MELO3C024552.2 | 1452 | 2958 | 1.027 | 1.57817E-20 | up |
| MELO3C024557.2 | 160 | 62 | -1.369 | 0.000173058 | down |
| MELO3C024559.2 | 117 | 258 | 1.143 | 5.72424E-09 | up |
| MELO3C024560.2 | 1 | 20 | 4.82 | 0.000160835 | up |
| MELO3C024569.2 | 101 | 258 | 1.359 | 9.94198E-10 | up |
| MELO3C024571.2 | 24 | 165 | 2.797 | 3.84E-08 | up |
| MELO3C024572.2 | 198 | 412 | 1.053 | 1.09254E-06 | up |
| MELO3C024574.2 | 8 | 45 | 2.501 | 0.000158698 | up |
| MELO3C024575.2 | 278 | 563 | 1.02 | 2.56155E-15 | up |
| MELO3C024579.2 | 724 | 229 | -1.66 | 2.16363E-20 | down |
| MELO3C024582.2 | 1204 | 113 | -3.404 | 3.51676E-100 | down |
| MELO3C024585.2 | 20 | 2 | -3.453 | 0.000872469 | down |
| MELO3C024591.2 | 1431 | 597 | -1.263 | 1.54273E-22 | down |
| MELO3C024603.2 | 2465 | 5999 | 1.283 | 1.79682E-19 | up |
| MELO3C024605.2 | 6517 | 517 | -3.655 | 2.6563E-92 | down |
| MELO3C024610.2 | 19613 | 1733 | -3.5 | 1.87873E-118 | down |
| MELO3C024627.2 | 6 | 24 | 1.919 | 0.012352417 | up |
| MELO3C024628.2 | 10 | 87 | 3.056 | 1.24931E-07 | up |
| MELO3C024629.2 | 0 | 61 | 8.266 | 4.37382E-11 | up |
| MELO3C024638.2 | 5 | 33 | 2.618 | 5.23944E-05 | up |
| MELO3C024660.2 | 292 | 1279 | 2.132 | 2.74499E-14 | up |
| MELO3C024664.2 | 2242 | 603 | -1.894 | 4.84803E-22 | down |
| MELO3C024669.2 | 1016 | 2055 | 1.015 | 2.41102E-32 | up |
| MELO3C024673.2 | 921 | 5385 | 2.548 | 2.75701E-114 | up |
| MELO3C024674.2 | 7763 | 3121 | -1.315 | 8.38251E-37 | down |
| MELO3C024679.2 | 1838 | 551 | -1.739 | 1.57701E-33 | down |
| MELO3C024693.2 | 122 | 292 | 1.256 | 2.13682E-08 | up |
| MELO3C024699.2 | 717 | 3449 | 2.267 | 3.47956E-69 | up |
| MELO3C024701.2 | 6579 | 2698 | -1.286 | 2.02826E-30 | down |
| MELO3C024704.2 | 193 | 484 | 1.325 | 6.68908E-20 | up |
| MELO3C024715.2 | 572 | 1223 | 1.096 | 1.41987E-24 | up |
| MELO3C024718.2 | 32 | 9 | -1.745 | 0.013856737 | down |
| MELO3C024728.2 | 501 | 1004 | 1.002 | 1.56567E-14 | up |
| MELO3C024731.2 | 0 | 79 | 8.646 | 1.62016E-11 | up |
| MELO3C024736.2 | 55 | 685 | 3.634 | 2.11167E-51 | up |
| MELO3C024745.2 | 47 | 13 | -1.818 | 0.001035872 | down |
| MELO3C024760.2 | 5891 | 1083 | -2.444 | 1.3605E-69 | down |
| MELO3C024766.2 | 64839 | 19176 | -1.758 | 5.52476E-26 | down |
| MELO3C024771.2 | 73177 | 3662 | -4.321 | 2.16409E-90 | down |
| MELO3C024777.2 | 583 | 1376 | 1.241 | 2.57211E-25 | up |
| MELO3C024781.2 | 304 | 629 | 1.05 | 5.29637E-10 | up |
| MELO3C024783.2 | 975 | 3060 | 1.65 | 7.40501E-56 | up |
| MELO3C024794.2 | 0 | 8 | 5.289 | 0.003685145 | up |
| MELO3C024796.2 | 113 | 1046 | 3.206 | 1.87059E-27 | up |
| MELO3C024799.2 | 4967 | 2123 | -1.226 | 9.46097E-20 | down |
| MELO3C024809.2 | 88 | 20 | -2.115 | 2.62897E-07 | down |
| MELO3C024820.2 | 3028 | 369 | -3.036 | 6.53855E-14 | down |
| MELO3C024832.2 | 0 | 13 | 6.076 | 6.41749E-05 | up |
| MELO3C024834.2 | 9 | 0 | -5.778 | 0.000925812 | down |
| MELO3C024842.2 | 17 | 0 | -6.636 | 0.00025368 | down |
| MELO3C024857.2 | 272 | 40 | -2.758 | 1.63953E-13 | down |
| MELO3C024871.2 | 1314 | 2666 | 1.021 | 4.31356E-32 | up |
| MELO3C024872.2 | 27 | 449 | 4.066 | 5.22349E-65 | up |
| MELO3C024877.2 | 28 | 1 | -4.535 | 2.90959E-06 | down |
| MELO3C024882.2 | 17 | 38 | 1.17 | 0.02010029 | up |
| MELO3C024885.2 | 1061 | 323 | -1.718 | 8.76493E-34 | down |
| MELO3C024886.2 | 404 | 30 | -3.749 | 1.83264E-19 | down |
| MELO3C024887.2 | 3579 | 1522 | -1.234 | 5.82319E-12 | down |
| MELO3C024898.2 | 5 | 36 | 2.756 | 2.66154E-06 | up |
| MELO3C024899.2 | 2113 | 725 | -1.544 | 1.43152E-33 | down |
| MELO3C024900.2 | 447 | 126 | -1.828 | 2.66571E-20 | down |
| MELO3C024915.2 | 111 | 231 | 1.06 | 1.00684E-06 | up |
| MELO3C024920.2 | 2967 | 574 | -2.371 | 1.13856E-43 | down |
| MELO3C024925.2 | 561 | 167 | -1.743 | 1.52414E-16 | down |
| MELO3C024938.2 | 148 | 850 | 2.524 | 6.69464E-55 | up |
| MELO3C024964.2 | 4927 | 1135 | -2.119 | 1.10454E-125 | down |
| MELO3C024972.2 | 105 | 311 | 1.566 | 2.35794E-16 | up |
| MELO3C024975.2 | 382 | 2828 | 2.889 | 7.89374E-131 | up |
| MELO3C024982.2 | 292 | 940 | 1.685 | 1.95547E-19 | up |
| MELO3C024990.2 | 371 | 814 | 1.133 | 2.28828E-23 | up |
| MELO3C025005.2 | 4373 | 762 | -2.522 | 2.06417E-46 | down |
| MELO3C025012.2 | 74 | 28 | -1.405 | 0.000106445 | down |
| MELO3C025019.2 | 1 | 17 | 4.543 | 0.000424233 | up |
| MELO3C025023.2 | 2749 | 1137 | -1.274 | 7.93137E-16 | down |
| MELO3C025026.2 | 1446 | 29 | -5.615 | 1.57504E-128 | down |
| MELO3C025027.2 | 38532 | 18285 | -1.075 | 1.92455E-49 | down |
| MELO3C025029.2 | 3134 | 25926 | 3.049 | 1.20455E-118 | up |
| MELO3C025034.2 | 1079 | 501 | -1.108 | 1.68909E-09 | down |
| MELO3C025049.2 | 110 | 326 | 1.565 | 2.16979E-16 | up |
| MELO3C025056.2 | 562 | 2529 | 2.169 | 1.40805E-32 | up |
| MELO3C025066.2 | 3 | 17 | 2.377 | 0.012227688 | up |
| MELO3C025078.2 | 5564 | 12275 | 1.142 | 2.24246E-49 | up |
| MELO3C025079.2 | 697 | 8 | -6.454 | 3.15331E-83 | down |
| MELO3C025087.2 | 242 | 555 | 1.195 | 1.42545E-15 | up |
| MELO3C025095.2 | 291 | 0 | -10.734 | 4.16203E-19 | down |
| MELO3C025101.2 | 21177 | 1114 | -4.248 | 1.31422E-143 | down |
| MELO3C025102.2 | 2649 | 556 | -2.251 | 2.22187E-73 | down |
| MELO3C025110.2 | 39 | 11 | -1.835 | 0.012084312 | down |
| MELO3C025111.2 | 9724 | 1358 | -2.842 | 3.94352E-152 | down |
| MELO3C025138.2 | 521 | 79 | -2.731 | 7.71243E-35 | down |
| MELO3C025142.2 | 225 | 100 | -1.166 | 5.79515E-09 | down |
| MELO3C025143.2 | 40 | 98 | 1.288 | 0.007230276 | up |
| MELO3C025149.2 | 16978 | 2667 | -2.671 | 2.60549E-69 | down |
| MELO3C025165.2 | 66 | 0 | -8.606 | 0.00024684 | down |
| MELO3C025166.2 | 129 | 11 | -3.618 | 0.003537335 | down |
| MELO3C025174.2 | 162 | 65 | -1.315 | 0.000593049 | down |
| MELO3C025183.2 | 864 | 203 | -2.084 | 2.40305E-15 | down |
| MELO3C025192.2 | 168 | 474 | 1.495 | 9.51532E-24 | up |
| MELO3C025194.2 | 1812 | 4294 | 1.245 | 4.34079E-64 | up |
| MELO3C025196.2 | 3494 | 1141 | -1.615 | 1.51802E-63 | down |
| MELO3C025199.2 | 57 | 19 | -1.601 | 0.007682471 | down |
| MELO3C025205.2 | 214 | 60 | -1.833 | 3.20941E-11 | down |
| MELO3C025206.2 | 56 | 437 | 2.958 | 3.44438E-19 | up |
| MELO3C025210.2 | 14 | 50 | 1.812 | 0.002045419 | up |
| MELO3C025219.2 | 1602 | 29562 | 4.205 | 6.72548E-06 | up |
| MELO3C025264.2 | 58853 | 973 | -5.918 | 1.23501E-62 | down |
| MELO3C025268.2 | 630 | 3 | -7.831 | 1.61235E-49 | down |
| MELO3C025282.2 | 491 | 1662 | 1.758 | 3.3811E-27 | up |
| MELO3C025291.2 | 8 | 25 | 1.684 | 0.013848872 | up |
| MELO3C025295.2 | 392 | 65 | -2.602 | 1.33966E-33 | down |
| MELO3C025297.2 | 64 | 212 | 1.74 | 2.01703E-16 | up |
| MELO3C025301.2 | 3 | 56 | 3.965 | 3.76895E-10 | up |
| MELO3C025307.2 | 1328 | 495 | -1.423 | 3.32928E-37 | down |
| MELO3C025316.2 | 2038 | 515 | -1.987 | 1.49396E-34 | down |
| MELO3C025332.2 | 39 | 111 | 1.497 | 0.003847708 | up |
| MELO3C025334.2 | 80 | 30 | -1.439 | 6.82649E-05 | down |
| MELO3C025338.2 | 137 | 711 | 2.376 | 2.46097E-47 | up |
| MELO3C025345.2 | 89 | 881 | 3.301 | 1.47071E-89 | up |
| MELO3C025346.2 | 6230 | 2249 | -1.47 | 5.55487E-39 | down |
| MELO3C025347.2 | 32452 | 14130 | -1.2 | 4.30845E-33 | down |
| MELO3C025352.2 | 6144 | 2113 | -1.54 | 3.25802E-36 | down |
| MELO3C025354.2 | 1021 | 3312 | 1.697 | 1.68741E-46 | up |
| MELO3C025365.2 | 121 | 1171 | 3.279 | 1.02563E-102 | up |
| MELO3C025375.2 | 3487 | 1191 | -1.549 | 2.1677E-38 | down |
| MELO3C025383.2 | 43 | 3 | -3.767 | 3.18417E-08 | down |
| MELO3C025392.2 | 178 | 84 | -1.089 | 3.53351E-05 | down |
| MELO3C025397.2 | 2054 | 848 | -1.276 | 3.2277E-15 | down |
| MELO3C025401.2 | 485 | 126 | -1.955 | 8.34366E-21 | down |
| MELO3C025405.2 | 95 | 29 | -1.72 | 9.98591E-06 | down |
| MELO3C025417.2 | 2782 | 1372 | -1.02 | 2.58661E-43 | down |
| MELO3C025423.2 | 6 | 0 | -5.16 | 0.004848621 | down |
| MELO3C025424.2 | 368 | 113 | -1.698 | 1.42555E-10 | down |
| MELO3C025433.2 | 152 | 39 | -1.947 | 2.17489E-06 | down |
| MELO3C025448.2 | 275 | 58 | -2.244 | 7.42477E-27 | down |
| MELO3C025456.2 | 356 | 87 | -2.025 | 1.89261E-22 | down |
| MELO3C025462.2 | 62 | 9 | -2.704 | 1.03066E-09 | down |
| MELO3C025463.2 | 187 | 69 | -1.427 | 7.52392E-06 | down |
| MELO3C025464.2 | 1448 | 205 | -2.817 | 5.88453E-122 | down |
| MELO3C025470.2 | 223 | 579 | 1.378 | 6.46822E-16 | up |
| MELO3C025473.2 | 747 | 1539 | 1.043 | 9.8694E-15 | up |
| MELO3C025475.2 | 619 | 284 | -1.121 | 4.89112E-12 | down |
| MELO3C025477.2 | 106 | 15 | -2.819 | 1.46893E-12 | down |
| MELO3C025484.2 | 88 | 2 | -5.575 | 8.04027E-14 | down |
| MELO3C025485.2 | 32 | 0 | -7.56 | 1.83662E-08 | down |
| MELO3C025493.2 | 1390 | 430 | -1.694 | 6.20905E-31 | down |
| MELO3C025505.2 | 41 | 228 | 2.473 | 2.27512E-16 | up |
| MELO3C025508.2 | 29 | 70 | 1.246 | 0.006671112 | up |
| MELO3C025521.2 | 36 | 6 | -2.438 | 0.005971115 | down |
| MELO3C025523.2 | 226 | 1 | -7.502 | 1.16709E-16 | down |
| MELO3C025525.2 | 83 | 312 | 1.907 | 3.53806E-13 | up |
| MELO3C025532.2 | 7 | 45 | 2.584 | 6.86254E-06 | up |
| MELO3C025554.2 | 8 | 1 | -3.674 | 0.018436987 | down |
| MELO3C025563.2 | 1020 | 148 | -2.789 | 6.38041E-37 | down |
| MELO3C025579.2 | 7 | 1055 | 7.278 | 9.88771E-90 | up |
| MELO3C025580.2 | 1163 | 581 | -1.001 | 1.34194E-06 | down |
| MELO3C025583.2 | 4410 | 2 | -11.244 | 4.09401E-45 | down |
| MELO3C025587.2 | 22109 | 478 | -5.531 | 2.32295E-292 | down |
| MELO3C025597.2 | 5 | 32 | 2.583 | 0.027397683 | up |
| MELO3C025598.2 | 332 | 771 | 1.216 | 1.25128E-16 | up |
| MELO3C025601.2 | 339 | 3108 | 3.198 | 3.37381E-75 | up |
| MELO3C025609.2 | 2406 | 432 | -2.476 | 3.05131E-43 | down |
| MELO3C025634.2 | 243 | 509 | 1.068 | 2.45524E-10 | up |
| MELO3C025641.2 | 264 | 658 | 1.319 | 1.3403E-17 | up |
| MELO3C025645.2 | 2304 | 1053 | -1.13 | 1.00559E-25 | down |
| MELO3C025651.2 | 197 | 1433 | 2.864 | 3.5672E-99 | up |
| MELO3C025655.2 | 116 | 42 | -1.467 | 0.000172814 | down |
| MELO3C025663.2 | 395 | 4615 | 3.546 | 1.67414E-64 | up |
| MELO3C025664.2 | 4 | 26 | 2.61 | 0.025981205 | up |
| MELO3C025667.2 | 50 | 346 | 2.8 | 5.45752E-33 | up |
| MELO3C025669.2 | 3684 | 848 | -2.12 | 3.92829E-57 | down |
| MELO3C025673.2 | 1427 | 460 | -1.632 | 1.04645E-30 | down |
| MELO3C025676.2 | 1 | 76 | 6.731 | 9.17406E-10 | up |
| MELO3C025677.2 | 199 | 664 | 1.736 | 6.28489E-24 | up |
| MELO3C025678.2 | 0 | 27 | 7.09 | 1.40853E-07 | up |
| MELO3C025688.2 | 2 | 26 | 3.605 | 7.64967E-05 | up |
| MELO3C025702.2 | 1478 | 625 | -1.24 | 1.15846E-12 | down |
| MELO3C025706.2 | 6 | 0 | -5.247 | 0.005811176 | down |
| MELO3C025709.2 | 13 | 2 | -3.065 | 0.004429394 | down |
| MELO3C025712.2 | 152 | 45 | -1.767 | 5.27183E-10 | down |
| MELO3C025717.2 | 19 | 2 | -3.139 | 0.000733854 | down |
| MELO3C025720.2 | 1978 | 7 | -8.136 | 6.47875E-109 | down |
| MELO3C025735.2 | 37 | 132 | 1.85 | 7.99446E-09 | up |
| MELO3C025737.2 | 16 | 60 | 1.973 | 0.000221423 | up |
| MELO3C025741.2 | 1410 | 3001 | 1.09 | 1.29787E-16 | up |
| MELO3C025742.2 | 46 | 13 | -1.868 | 0.000547476 | down |
| MELO3C025744.2 | 295 | 87 | -1.753 | 1.22921E-15 | down |
| MELO3C025754.2 | 44 | 19 | -1.222 | 0.010291553 | down |
| MELO3C025758.2 | 728 | 1995 | 1.455 | 3.36552E-33 | up |
| MELO3C025761.2 | 297 | 2775 | 3.225 | 2.04229E-50 | up |
| MELO3C025764.2 | 10 | 509 | 5.719 | 3.70408E-64 | up |
| MELO3C025770.2 | 84 | 30 | -1.501 | 2.04664E-05 | down |
| MELO3C025771.2 | 137 | 310 | 1.18 | 5.01365E-10 | up |
| MELO3C025772.2 | 14327 | 4412 | -1.699 | 9.71175E-25 | down |
| MELO3C025779.2 | 141 | 338 | 1.256 | 2.20595E-13 | up |
| MELO3C025780.2 | 893 | 1914 | 1.1 | 0.020954204 | up |
| MELO3C025783.2 | 286 | 2691 | 3.234 | 5.90148E-111 | up |
| MELO3C025784.2 | 274 | 664 | 1.275 | 9.10966E-08 | up |
| MELO3C025791.2 | 1042 | 311 | -1.747 | 1.30343E-17 | down |
| MELO3C025794.2 | 328 | 66 | -2.314 | 5.75442E-17 | down |
| MELO3C025795.2 | 942 | 356 | -1.402 | 2.77821E-24 | down |
| MELO3C025797.2 | 7 | 34 | 2.245 | 0.000614234 | up |
| MELO3C025798.2 | 15732 | 185 | -6.407 | 1.5687E-46 | down |
| MELO3C025807.2 | 5 | 511 | 6.779 | 1.19977E-52 | up |
| MELO3C025837.2 | 32 | 3 | -3.497 | 1.68833E-05 | down |
| MELO3C025851.2 | 2 | 12 | 2.234 | 0.021407633 | up |
| MELO3C025853.2 | 1449 | 462 | -1.65 | 1.26481E-23 | down |
| MELO3C025855.2 | 2597 | 475 | -2.453 | 7.7354E-97 | down |
| MELO3C025857.2 | 493 | 1191 | 1.274 | 3.30614E-19 | up |
| MELO3C025861.2 | 549 | 250 | -1.133 | 1.21046E-08 | down |
| MELO3C025862.2 | 48 | 129 | 1.43 | 5.00116E-08 | up |
| MELO3C025863.2 | 476 | 153 | -1.639 | 1.81806E-29 | down |
| MELO3C025864.2 | 73 | 422 | 2.531 | 6.54478E-25 | up |
| MELO3C025865.2 | 597 | 211 | -1.502 | 5.28483E-13 | down |
| MELO3C025866.2 | 339 | 716 | 1.076 | 2.00776E-09 | up |
| MELO3C025869.2 | 3107 | 1268 | -1.293 | 6.95768E-31 | down |
| MELO3C025877.2 | 1167 | 2486 | 1.092 | 5.99532E-06 | up |
| MELO3C025879.2 | 297 | 633 | 1.093 | 2.31199E-09 | up |
| MELO3C025882.2 | 50 | 362 | 2.865 | 5.1653E-27 | up |
| MELO3C025883.2 | 68 | 408 | 2.583 | 1.70924E-27 | up |
| MELO3C025884.2 | 8 | 71 | 3.178 | 9.29196E-10 | up |
| MELO3C025885.2 | 80 | 232 | 1.536 | 5.99436E-12 | up |
| MELO3C025888.2 | 12785 | 877 | -3.864 | 3.01946E-117 | down |
| MELO3C025892.2 | 9499 | 4068 | -1.224 | 1.85736E-33 | down |
| MELO3C025896.2 | 272 | 128 | -1.09 | 2.61361E-06 | down |
| MELO3C025900.2 | 0 | 20 | 6.627 | 2.50233E-06 | up |
| MELO3C025902.2 | 79 | 23 | -1.789 | 1.3407E-07 | down |
| MELO3C025907.2 | 11 | 114 | 3.356 | 2.93871E-11 | up |
| MELO3C025912.2 | 2739 | 110 | -4.634 | 4.4675E-178 | down |
| MELO3C025914.2 | 1075 | 489 | -1.138 | 1.10487E-16 | down |
| MELO3C025918.2 | 635 | 258 | -1.301 | 2.09691E-20 | down |
| MELO3C025924.2 | 2 | 30 | 3.561 | 5.48259E-06 | up |
| MELO3C025925.2 | 2730 | 5478 | 1.005 | 3.36341E-24 | up |
| MELO3C025940.2 | 125 | 691 | 2.475 | 1.15919E-56 | up |
| MELO3C025944.2 | 2542 | 1239 | -1.038 | 1.11545E-11 | down |
| MELO3C025951.2 | 1129 | 508 | -1.153 | 4.36369E-30 | down |
| MELO3C025953.2 | 1609 | 741 | -1.119 | 2.32517E-16 | down |
| MELO3C025955.2 | 31 | 4 | -2.746 | 7.23792E-05 | down |
| MELO3C025974.2 | 42 | 0 | -7.941 | 1.04164E-09 | down |
| MELO3C025984.2 | 156 | 73 | -1.094 | 0.0006766 | down |
| MELO3C026001.2 | 53 | 12 | -2.118 | 1.37226E-05 | down |
| MELO3C026008.2 | 113 | 11 | -3.37 | 5.19562E-08 | down |
| MELO3C026013.2 | 39 | 95 | 1.292 | 0.000177404 | up |
| MELO3C026018.2 | 871 | 3508 | 2.009 | 2.85488E-98 | up |
| MELO3C026019.2 | 293 | 1101 | 1.91 | 5.33538E-50 | up |
| MELO3C026029.2 | 102 | 969 | 3.253 | 1.80256E-39 | up |
| MELO3C026034.2 | 13 | 146 | 3.502 | 3.46601E-13 | up |
| MELO3C026045.2 | 3860 | 945 | -2.03 | 2.86814E-08 | down |
| MELO3C026046.2 | 3437 | 735 | -2.226 | 2.14029E-120 | down |
| MELO3C026050.2 | 1817 | 620 | -1.552 | 5.53401E-33 | down |
| MELO3C026051.2 | 563 | 1702 | 1.596 | 2.78395E-51 | up |
| MELO3C026054.2 | 12921 | 5703 | -1.18 | 1.87552E-49 | down |
| MELO3C026058.2 | 1737 | 137 | -3.659 | 3.27939E-119 | down |
| MELO3C026066.2 | 536 | 1 | -9.768 | 8.79576E-21 | down |
| MELO3C026077.2 | 2884 | 5877 | 1.027 | 2.28126E-23 | up |
| MELO3C026099.2 | 21078 | 3236 | -2.703 | 5.03056E-151 | down |
| MELO3C026109.2 | 1193 | 268 | -2.151 | 1.69691E-51 | down |
| MELO3C026134.2 | 109 | 0 | -9.321 | 4.88611E-14 | down |
| MELO3C026137.2 | 256 | 70 | -1.859 | 8.5615E-12 | down |
| MELO3C026142.2 | 27 | 2 | -3.477 | 2.57404E-05 | down |
| MELO3C026143.2 | 7 | 106 | 3.97 | 9.45559E-19 | up |
| MELO3C026160.2 | 4751 | 1809 | -1.394 | 6.92771E-52 | down |
| MELO3C026170.2 | 1283 | 42 | -4.936 | 1.41589E-50 | down |
| MELO3C026178.2 | 83 | 230 | 1.461 | 2.42978E-08 | up |
| MELO3C026179.2 | 14 | 3 | -2.13 | 0.021202625 | down |
| MELO3C026183.2 | 156 | 0 | -9.836 | 3.36154E-06 | down |
| MELO3C026184.2 | 1093 | 166 | -2.715 | 0.00132582 | down |
| MELO3C026194.2 | 15 | 166 | 3.472 | 2.71519E-12 | up |
| MELO3C026199.2 | 140 | 392 | 1.485 | 6.74292E-18 | up |
| MELO3C026207.2 | 3401 | 1686 | -1.013 | 2.92786E-07 | down |
| MELO3C026220.2 | 24 | 94 | 1.992 | 2.19725E-06 | up |
| MELO3C026223.2 | 780 | 377 | -1.049 | 2.03021E-11 | down |
| MELO3C026226.2 | 6 | 32 | 2.313 | 0.00124817 | up |
| MELO3C026229.2 | 13905 | 5209 | -1.416 | 3.13467E-41 | down |
| MELO3C026235.2 | 2439 | 238 | -3.358 | 2.88687E-90 | down |
| MELO3C026236.2 | 40 | 84 | 1.058 | 0.001171174 | up |
| MELO3C026238.2 | 18 | 111 | 2.632 | 2.4426E-06 | up |
| MELO3C026240.2 | 492 | 215 | -1.198 | 2.38266E-09 | down |
| MELO3C026242.2 | 7 | 34 | 2.146 | 0.000130955 | up |
| MELO3C026246.2 | 7649 | 1966 | -1.96 | 3.93786E-49 | down |
| MELO3C026247.2 | 2815 | 686 | -2.037 | 3.27791E-50 | down |
| MELO3C026250.2 | 2037 | 495 | -2.041 | 2.571E-26 | down |
| MELO3C026252.2 | 703 | 320 | -1.138 | 1.19719E-11 | down |
| MELO3C026259.2 | 18206 | 4643 | -1.971 | 1.27548E-41 | down |
| MELO3C026260.2 | 10 | 1 | -3.091 | 0.024926056 | down |
| MELO3C026261.2 | 247 | 104 | -1.249 | 2.64013E-11 | down |
| MELO3C026262.2 | 124 | 31 | -2.034 | 1.27713E-10 | down |
| MELO3C026265.2 | 1 | 57 | 6.337 | 1.22936E-07 | up |
| MELO3C026270.2 | 14 | 175 | 3.691 | 3.90992E-16 | up |
| MELO3C026272.2 | 96 | 439 | 2.192 | 1.16895E-36 | up |
| MELO3C026278.2 | 1 | 402 | 8.135 | 2.89669E-26 | up |
| MELO3C026282.2 | 179 | 416 | 1.212 | 1.7037E-10 | up |
| MELO3C026288.2 | 4461 | 1487 | -1.586 | 1.20293E-45 | down |
| MELO3C026289.2 | 75 | 10 | -2.943 | 1.7909E-09 | down |
| MELO3C026292.2 | 7499 | 1748 | -2.101 | 3.00969E-159 | down |
| MELO3C026296.2 | 718 | 1818 | 1.341 | 3.24604E-29 | up |
| MELO3C026300.2 | 24127 | 10026 | -1.267 | 5.9088E-36 | down |
| MELO3C026307.2 | 93 | 35 | -1.422 | 0.000142918 | down |
| MELO3C026308.2 | 34 | 148 | 2.142 | 2.0083E-14 | up |
| MELO3C026310.2 | 459 | 1113 | 1.276 | 4.84191E-10 | up |
| MELO3C026337.2 | 1113 | 503 | -1.149 | 5.83351E-17 | down |
| MELO3C026340.2 | 3 | 186 | 5.85 | 6.19602E-26 | up |
| MELO3C026342.2 | 457 | 974 | 1.092 | 7.93511E-06 | up |
| MELO3C026359.2 | 0 | 12 | 5.871 | 0.001006145 | up |
| MELO3C026366.2 | 165 | 46 | -1.83 | 8.63096E-11 | down |
| MELO3C026367.2 | 45 | 16 | -1.433 | 0.017065291 | down |
| MELO3C026368.2 | 1840 | 742 | -1.31 | 3.72533E-33 | down |
| MELO3C026369.2 | 43 | 120 | 1.472 | 9.34659E-07 | up |
| MELO3C026372.2 | 3495 | 646 | -2.438 | 4.35166E-56 | down |
| MELO3C026374.2 | 1214 | 412 | -1.56 | 5.56056E-26 | down |
| MELO3C026375.2 | 441 | 172 | -1.357 | 7.65777E-08 | down |
| MELO3C026378.2 | 2148 | 457 | -2.229 | 2.68188E-22 | down |
| MELO3C026380.2 | 521 | 197 | -1.406 | 7.54799E-20 | down |
| MELO3C026387.2 | 1255 | 407 | -1.623 | 3.99523E-48 | down |
| MELO3C026388.2 | 104 | 272 | 1.394 | 3.50602E-07 | up |
| MELO3C026395.2 | 4 | 70 | 4.272 | 2.08265E-12 | up |
| MELO3C026402.2 | 2090 | 140 | -3.899 | 1.23075E-140 | down |
| MELO3C026419.2 | 290 | 902 | 1.638 | 7.48725E-22 | up |
| MELO3C026420.2 | 9 | 0 | -5.759 | 0.007398117 | down |
| MELO3C026424.2 | 2314 | 598 | -1.953 | 1.7587E-113 | down |
| MELO3C026431.2 | 170 | 46 | -1.873 | 1.69268E-06 | down |
| MELO3C026432.2 | 30 | 7 | -2.053 | 0.000757845 | down |
| MELO3C026436.2 | 18378 | 7586 | -1.277 | 2.73844E-36 | down |
| MELO3C026468.2 | 990 | 142 | -2.807 | 7.71078E-28 | down |
| MELO3C026484.2 | 27 | 99 | 1.844 | 5.08408E-06 | up |
| MELO3C026485.2 | 25 | 146 | 2.525 | 1.07549E-12 | up |
| MELO3C026486.2 | 2194 | 0 | -13.652 | 2.27658E-30 | down |
| MELO3C026489.2 | 77 | 3984 | 5.694 | 3.03484E-171 | up |
| MELO3C026493.2 | 119 | 1029 | 3.113 | 1.13186E-09 | up |
| MELO3C026494.2 | 32638 | 14493 | -1.171 | 4.99578E-39 | down |
| MELO3C026498.2 | 3003 | 104 | -4.853 | 3.65317E-238 | down |
| MELO3C026500.2 | 3212 | 128 | -4.65 | 2.47538E-205 | down |
| MELO3C026507.2 | 42 | 7 | -2.531 | 7.98624E-05 | down |
| MELO3C026509.2 | 9 | 116 | 3.765 | 8.27088E-13 | up |
| MELO3C026512.2 | 24300 | 7125 | -1.77 | 2.73923E-92 | down |
| MELO3C026517.2 | 39 | 92 | 1.251 | 7.4239E-05 | up |
| MELO3C026518.2 | 300 | 132 | -1.188 | 1.53007E-07 | down |
| MELO3C026529.2 | 62 | 6 | -3.478 | 5.57316E-10 | down |
| MELO3C026535.2 | 917 | 153 | -2.58 | 7.21174E-19 | down |
| MELO3C026537.2 | 225 | 451 | 1.003 | 2.4248E-09 | up |
| MELO3C026548.2 | 7 | 0 | -5.463 | 0.003461385 | down |
| MELO3C026550.2 | 9784 | 30589 | 1.645 | 1.21912E-17 | up |
| MELO3C026552.2 | 16 | 71 | 2.148 | 0.000110357 | up |
| MELO3C026557.2 | 38 | 150 | 2.004 | 0.000145726 | up |
| MELO3C026558.2 | 290 | 1884 | 2.701 | 9.76232E-101 | up |
| MELO3C026563.2 | 46 | 123 | 1.43 | 1.18375E-06 | up |
| MELO3C026564.2 | 663 | 45 | -3.875 | 1.37025E-45 | down |
| MELO3C026565.2 | 6 | 247 | 5.43 | 1.57203E-25 | up |
| MELO3C026569.2 | 241 | 3 | -6.45 | 1.53613E-31 | down |
| MELO3C026575.2 | 478 | 59 | -3.012 | 5.7416E-16 | down |
| MELO3C026578.2 | 5 | 62 | 3.64 | 1.89675E-10 | up |
| MELO3C026581.2 | 1681 | 599 | -1.486 | 1.77532E-35 | down |
| MELO3C026585.2 | 1935 | 4832 | 1.321 | 4.53224E-47 | up |
| MELO3C026587.2 | 295 | 724 | 1.296 | 3.48933E-21 | up |
| MELO3C026590.2 | 282 | 3452 | 3.616 | 1.11779E-109 | up |
| MELO3C026594.2 | 2019 | 173 | -3.543 | 2.74132E-134 | down |
| MELO3C026599.2 | 21 | 0 | -6.93 | 6.55224E-07 | down |
| MELO3C026603.2 | 74 | 955 | 3.687 | 3.83678E-102 | up |
| MELO3C026609.2 | 9 | 84 | 3.23 | 2.23167E-12 | up |
| MELO3C026611.2 | 2081 | 385 | -2.436 | 1.47504E-89 | down |
| MELO3C026618.2 | 73 | 169 | 1.216 | 2.84472E-06 | up |
| MELO3C026620.2 | 597 | 297 | -1.006 | 3.1299E-13 | down |
| MELO3C026629.2 | 1243 | 4699 | 1.919 | 2.37238E-57 | up |
| MELO3C026630.2 | 332 | 114 | -1.547 | 2.78935E-10 | down |
| MELO3C026634.2 | 7 | 0 | -5.323 | 0.004848653 | down |
| MELO3C026637.2 | 57 | 21 | -1.425 | 0.001882987 | down |
| MELO3C026642.2 | 45 | 21 | -1.076 | 0.025872849 | down |
| MELO3C026652.2 | 13 | 0 | -6.28 | 2.7893E-05 | down |
| MELO3C026654.2 | 236 | 0 | -10.434 | 8.36911E-18 | down |
| MELO3C026657.2 | 5721 | 2631 | -1.121 | 1.91132E-38 | down |
| MELO3C026658.2 | 532 | 1577 | 1.567 | 4.12631E-37 | up |
| MELO3C026665.2 | 294 | 856 | 1.543 | 2.73677E-18 | up |
| MELO3C026677.2 | 7 | 469 | 6.191 | 1.51314E-43 | up |
| MELO3C026685.2 | 60 | 374 | 2.637 | 1.65215E-28 | up |
| MELO3C026686.2 | 342 | 1 | -8.525 | 4.19844E-23 | down |
| MELO3C026687.2 | 31 | 0 | -6.532 | 8.88814E-07 | down |
| MELO3C026689.2 | 1539 | 190 | -3.014 | 2.57075E-91 | down |
| MELO3C026703.2 | 9 | 1 | -2.939 | 0.017677492 | down |
| MELO3C026715.2 | 1266 | 579 | -1.129 | 1.17251E-18 | down |
| MELO3C026718.2 | 0 | 3494 | 14.111 | 1.61101E-22 | up |
| MELO3C026722.2 | 8545 | 1303 | -2.714 | 1.42507E-79 | down |
| MELO3C026731.2 | 2 | 491 | 8.1 | 7.20773E-34 | up |
| MELO3C026732.2 | 9281 | 4312 | -1.106 | 3.66304E-31 | down |
| MELO3C026734.2 | 61 | 578 | 3.24 | 1.20901E-57 | up |
| MELO3C026737.2 | 423 | 1537 | 1.863 | 8.14587E-53 | up |
| MELO3C026738.2 | 1232 | 38 | -5.011 | 7.3702E-107 | down |
| MELO3C026748.2 | 11 | 188 | 4.079 | 3.35799E-31 | up |
| MELO3C026749.2 | 202 | 1040 | 2.362 | 1.12206E-27 | up |
| MELO3C026754.2 | 1029 | 190 | -2.435 | 0.001157457 | down |
| MELO3C026755.2 | 101 | 728 | 2.845 | 0.000760964 | up |
| MELO3C026772.2 | 52 | 109 | 1.06 | 4.56666E-05 | up |
| MELO3C026781.2 | 4437 | 9864 | 1.153 | 6.88627E-62 | up |
| MELO3C026782.2 | 6 | 1084 | 7.563 | 1.89564E-71 | up |
| MELO3C026784.2 | 569 | 210 | -1.438 | 8.81187E-09 | down |
| MELO3C026796.2 | 115 | 40 | -1.526 | 1.62469E-06 | down |
| MELO3C026802.2 | 22551 | 1055 | -4.417 | 1.73001E-113 | down |
| MELO3C026803.2 | 392 | 1421 | 1.859 | 5.04399E-62 | up |
| MELO3C026805.2 | 16 | 530 | 5.01 | 1.18388E-70 | up |
| MELO3C026807.2 | 20 | 4 | -2.424 | 0.008997449 | down |
| MELO3C026808.2 | 10 | 1 | -3.031 | 0.027120289 | down |
| MELO3C026811.2 | 66 | 135 | 1.025 | 0.005408004 | up |
| MELO3C026818.2 | 0 | 15 | 6.224 | 3.44642E-05 | up |
| MELO3C026824.2 | 392 | 72 | -2.452 | 8.12531E-08 | down |
| MELO3C026828.2 | 126 | 23 | -2.43 | 9.91851E-05 | down |
| MELO3C026842.2 | 29 | 0 | -7.417 | 7.49731E-07 | down |
| MELO3C026843.2 | 113 | 13 | -3.078 | 2.44333E-08 | down |
| MELO3C026845.2 | 2509 | 1113 | -1.173 | 7.16437E-33 | down |
| MELO3C026846.2 | 80 | 32 | -1.317 | 0.000210789 | down |
| MELO3C026847.2 | 351 | 147 | -1.257 | 1.00878E-13 | down |
| MELO3C026848.2 | 98 | 28 | -1.827 | 3.59615E-08 | down |
| MELO3C026855.2 | 690 | 1822 | 1.402 | 9.79806E-20 | up |
| MELO3C026868.2 | 30 | 8 | -1.831 | 0.016864711 | down |
| MELO3C026870.2 | 2649 | 12943 | 2.289 | 9.69409E-47 | up |
| MELO3C026873.2 | 2228 | 820 | -1.444 | 8.74353E-40 | down |
| MELO3C026875.2 | 11134 | 3719 | -1.582 | 6.789E-70 | down |
| MELO3C026887.2 | 132 | 33 | -2.017 | 6.32366E-11 | down |
| MELO3C026889.2 | 348 | 34 | -3.386 | 6.80558E-18 | down |
| MELO3C026892.2 | 15 | 62 | 2.08 | 7.69235E-06 | up |
| MELO3C026896.2 | 1171 | 211 | -2.466 | 9.73611E-24 | down |
| MELO3C026897.2 | 6208 | 2214 | -1.487 | 4.45538E-51 | down |
| MELO3C026900.2 | 18 | 5 | -1.733 | 0.017354324 | down |
| MELO3C026901.2 | 4141 | 1822 | -1.185 | 3.13637E-40 | down |
| MELO3C026918.2 | 409 | 199 | -1.042 | 1.62502E-11 | down |
| MELO3C026919.2 | 26949 | 11840 | -1.187 | 9.20543E-43 | down |
| MELO3C026921.2 | 53 | 158 | 1.564 | 1.74132E-09 | up |
| MELO3C026941.2 | 4 | 32 | 2.783 | 7.89461E-06 | up |
| MELO3C026945.2 | 452 | 165 | -1.456 | 1.12363E-09 | down |
| MELO3C026947.2 | 126 | 1 | -7.664 | 4.95009E-12 | down |
| MELO3C026948.2 | 91 | 189 | 1.058 | 1.52532E-06 | up |
| MELO3C026950.2 | 821 | 1762 | 1.103 | 2.34363E-26 | up |
| MELO3C026951.2 | 331 | 26 | -3.648 | 1.54355E-43 | down |
| MELO3C026959.2 | 554 | 274 | -1.016 | 8.42646E-08 | down |
| MELO3C026961.2 | 40 | 5 | -3.025 | 8.81785E-07 | down |
| MELO3C026967.2 | 195 | 37 | -2.388 | 3.04647E-19 | down |
| MELO3C026968.2 | 999 | 243 | -2.041 | 6.73766E-38 | down |
| MELO3C026974.2 | 48 | 18 | -1.388 | 0.008700514 | down |
| MELO3C026991.2 | 46 | 103 | 1.174 | 0.000179848 | up |
| MELO3C026992.2 | 605 | 208 | -1.536 | 1.13244E-10 | down |
| MELO3C026997.2 | 849 | 424 | -1.005 | 4.17051E-14 | down |
| MELO3C026998.2 | 119 | 241 | 1.023 | 1.54473E-07 | up |
| MELO3C027001.2 | 72 | 643 | 3.154 | 1.29901E-26 | up |
| MELO3C027004.2 | 140 | 59 | -1.251 | 1.62666E-05 | down |
| MELO3C027005.2 | 69 | 14 | -2.268 | 2.04943E-07 | down |
| MELO3C027009.2 | 278 | 18 | -3.922 | 1.40564E-41 | down |
| MELO3C027013.2 | 8 | 1 | -3.783 | 0.014609066 | down |
| MELO3C027040.2 | 10 | 139 | 3.843 | 5.04772E-16 | up |
| MELO3C027052.2 | 8 | 1 | -3.736 | 0.017633515 | down |
| MELO3C027057.2 | 477 | 7 | -6.15 | 9.29907E-44 | down |
| MELO3C027060.2 | 146 | 377 | 1.363 | 5.29789E-05 | up |
| MELO3C027061.2 | 13215 | 4902 | -1.431 | 7.69994E-48 | down |
| MELO3C027064.2 | 1065 | 369 | -1.527 | 2.44785E-42 | down |
| MELO3C027082.2 | 60 | 14 | -2.045 | 5.99203E-05 | down |
| MELO3C027083.2 | 633 | 16 | -5.307 | 4.18842E-100 | down |
| MELO3C027089.2 | 236 | 508 | 1.106 | 3.0478E-07 | up |
| MELO3C027107.2 | 120 | 48 | -1.327 | 6.86923E-05 | down |
| MELO3C027112.2 | 3387 | 325 | -3.383 | 6.18072E-47 | down |
| MELO3C027119.2 | 4887 | 1135 | -2.106 | 5.77549E-85 | down |
| MELO3C027120.2 | 481 | 1667 | 1.794 | 2.18947E-31 | up |
| MELO3C027124.2 | 321 | 34 | -3.235 | 1.10031E-35 | down |
| MELO3C027137.2 | 37 | 2 | -4.125 | 1.65295E-07 | down |
| MELO3C027143.2 | 1171 | 546 | -1.101 | 3.09138E-27 | down |
| MELO3C027158.2 | 1777 | 4939 | 1.475 | 4.82133E-42 | up |
| MELO3C027159.2 | 133 | 11 | -3.577 | 7.93803E-19 | down |
| MELO3C027169.2 | 6 | 0 | -5.073 | 0.00670808 | down |
| MELO3C027172.2 | 1160 | 2498 | 1.106 | 0.014648834 | up |
| MELO3C027175.2 | 75 | 35 | -1.114 | 0.002267133 | down |
| MELO3C027179.2 | 134 | 440 | 1.717 | 3.58455E-15 | up |
| MELO3C027184.2 | 33 | 113 | 1.783 | 2.77967E-08 | up |
| MELO3C027185.2 | 64 | 170 | 1.401 | 1.07251E-06 | up |
| MELO3C027208.2 | 0 | 363 | 10.845 | 3.83885E-18 | up |
| MELO3C027212.2 | 667 | 299 | -1.156 | 1.58263E-10 | down |
| MELO3C027216.2 | 60 | 11 | -2.425 | 8.51467E-06 | down |
| MELO3C027220.2 | 10 | 0 | -5.833 | 0.001239629 | down |
| MELO3C027227.2 | 19 | 3 | -2.789 | 0.000842565 | down |
| MELO3C027239.2 | 92 | 206 | 1.152 | 1.29848E-05 | up |
| MELO3C027244.2 | 565 | 178 | -1.662 | 9.63196E-08 | down |
| MELO3C027253.2 | 4 | 18 | 2.199 | 0.009681275 | up |
| MELO3C027254.2 | 20 | 84 | 2.067 | 3.01674E-07 | up |
| MELO3C027257.2 | 47 | 145 | 1.619 | 0.001747666 | up |
| MELO3C027259.2 | 132 | 0 | -9.601 | 2.72621E-15 | down |
| MELO3C027276.2 | 0 | 7 | 5.127 | 0.00471373 | up |
| MELO3C027277.2 | 364 | 2 | -7.617 | 4.89843E-31 | down |
| MELO3C027278.2 | 13 | 40 | 1.677 | 0.000750883 | up |
| MELO3C027297.2 | 1034 | 263 | -1.979 | 2.01494E-36 | down |
| MELO3C027325.2 | 39 | 0 | -7.821 | 6.13189E-09 | down |
| MELO3C027337.2 | 15 | 3 | -2.198 | 0.019207501 | down |
| MELO3C027345.2 | 2712 | 1156 | -1.23 | 1.51439E-18 | down |
| MELO3C027346.2 | 22 | 283 | 3.641 | 0.000780434 | up |
| MELO3C027362.2 | 2106 | 705 | -1.58 | 1.30465E-24 | down |
| MELO3C027375.2 | 168 | 448 | 1.415 | 3.00625E-09 | up |
| MELO3C027376.2 | 56 | 0 | -8.364 | 4.68095E-11 | down |
| MELO3C027378.2 | 201 | 26 | -2.929 | 1.25331E-24 | down |
| MELO3C027379.2 | 976 | 2232 | 1.193 | 3.21691E-21 | up |
| MELO3C027385.2 | 259 | 703 | 1.44 | 5.52659E-20 | up |
| MELO3C027408.2 | 3 | 29 | 3.319 | 0.000951047 | up |
| MELO3C027413.2 | 38 | 12 | -1.608 | 0.001384432 | down |
| MELO3C027417.2 | 31 | 12 | -1.336 | 0.017774012 | down |
| MELO3C027420.2 | 236 | 1495 | 2.663 | 2.14814E-40 | up |
| MELO3C027425.2 | 508 | 250 | -1.022 | 4.86852E-06 | down |
| MELO3C027428.2 | 339 | 10 | -5.105 | 2.82515E-58 | down |
| MELO3C027441.2 | 121 | 738 | 2.609 | 2.74619E-53 | up |
| MELO3C027448.2 | 855 | 112 | -2.933 | 3.06396E-24 | down |
| MELO3C027455.2 | 0 | 6 | 3.885 | 0.028147239 | up |
| MELO3C027462.2 | 83 | 35 | -1.274 | 0.000159309 | down |
| MELO3C027466.2 | 0 | 15 | 6.227 | 4.24651E-05 | up |
| MELO3C027467.2 | 17 | 45 | 1.431 | 0.001065078 | up |
| MELO3C027479.2 | 237 | 0 | -10.44 | 1.02786E-17 | down |
| MELO3C027509.2 | 0 | 6 | 4.919 | 0.007803376 | up |
| MELO3C027538.2 | 106 | 0 | -9.278 | 4.73403E-14 | down |
| MELO3C027553.2 | 6 | 25 | 1.962 | 0.003892036 | up |
| MELO3C027577.2 | 192 | 79 | -1.289 | 1.66108E-07 | down |
| MELO3C027595.2 | 94 | 34 | -1.478 | 1.9356E-06 | down |
| MELO3C027607.2 | 56 | 0 | -8.348 | 7.83824E-11 | down |
| MELO3C027615.2 | 173 | 629 | 1.865 | 2.08512E-22 | up |
| MELO3C027618.2 | 427 | 0 | -10.328 | 4.30795E-18 | down |
| MELO3C027632.2 | 0 | 8 | 5.361 | 0.001506217 | up |
| MELO3C027633.2 | 12 | 0 | -6.068 | 0.000107059 | down |
| MELO3C027635.2 | 4 | 0 | -4.529 | 0.028513558 | down |
| MELO3C027646.2 | 0 | 732 | 10.894 | 5.08975E-20 | up |
| MELO3C027654.2 | 3 | 43 | 3.731 | 6.7935E-08 | up |
| MELO3C027671.2 | 32 | 0 | -7.564 | 1.3603E-08 | down |
| MELO3C027697.2 | 11 | 0 | -6.069 | 0.000121716 | down |
| MELO3C027699.2 | 0 | 11 | 5.879 | 0.000159368 | up |
| MELO3C027701.2 | 135 | 0 | -9.636 | 1.97334E-15 | down |
| MELO3C027702.2 | 0 | 7 | 5.216 | 0.00546774 | up |
| MELO3C027737.2 | 377 | 162 | -1.218 | 4.52174E-14 | down |
| MELO3C027740.2 | 74 | 14 | -2.369 | 1.33318E-07 | down |
| MELO3C027746.2 | 190 | 5 | -5.202 | 1.16304E-25 | down |
| MELO3C027753.2 | 33 | 5 | -2.664 | 0.000125915 | down |
| MELO3C027756.2 | 0 | 65 | 8.356 | 2.76242E-11 | up |
| MELO3C027769.2 | 702 | 0 | -12.009 | 5.6379E-24 | down |
| MELO3C027773.2 | 97 | 406 | 2.064 | 1.0078E-28 | up |
| MELO3C027785.2 | 0 | 17 | 6.417 | 8.72953E-06 | up |
| MELO3C027812.2 | 23 | 7 | -1.77 | 0.004963131 | down |
| MELO3C027813.2 | 26 | 3 | -2.933 | 9.94439E-05 | down |
| MELO3C027820.2 | 24 | 65 | 1.418 | 0.000272259 | up |
| MELO3C027823.2 | 119 | 52 | -1.189 | 0.000471128 | down |
| MELO3C027831.2 | 3 | 21 | 2.563 | 0.001653473 | up |
| MELO3C027836.2 | 44 | 10 | -2.104 | 0.000194363 | down |
| MELO3C027843.2 | 37 | 0 | -7.784 | 2.02307E-09 | down |
| MELO3C027847.2 | 0 | 20 | 6.678 | 2.70273E-06 | up |
| MELO3C027855.2 | 1621 | 461 | -1.813 | 7.19164E-53 | down |
| MELO3C027872.2 | 1053 | 0 | -12.593 | 2.37302E-26 | down |
| MELO3C027877.2 | 1 | 7 | 3.418 | 0.024252224 | up |
| MELO3C027880.2 | 0 | 5 | 4.821 | 0.010347868 | up |
| MELO3C027887.2 | 18 | 1 | -3.605 | 0.000328478 | down |
| MELO3C027893.2 | 3 | 29 | 3.163 | 1.01191E-05 | up |
| MELO3C027907.2 | 124 | 274 | 1.14 | 0.000785514 | up |
| MELO3C027912.2 | 0 | 30 | 7.254 | 1.22796E-07 | up |
| MELO3C027913.2 | 53 | 8 | -2.599 | 1.62928E-05 | down |
| MELO3C027914.2 | 81 | 173 | 1.109 | 0.000395271 | up |
| MELO3C027922.2 | 0 | 15 | 6.308 | 1.39554E-05 | up |
| MELO3C027934.2 | 0 | 51 | 8.027 | 2.34531E-10 | up |
| MELO3C027940.2 | 9 | 0 | -4.692 | 0.003810233 | down |
| MELO3C027943.2 | 10 | 2 | -2.339 | 0.026281584 | down |
| MELO3C027946.2 | 2 | 102 | 5.571 | 3.28314E-15 | up |
| MELO3C027949.2 | 0 | 13 | 5.996 | 7.12471E-05 | up |
| MELO3C027962.2 | 109 | 656 | 2.592 | 4.83763E-53 | up |
| MELO3C027967.2 | 11 | 0 | -5.118 | 0.003434431 | down |
| MELO3C027971.2 | 96 | 298 | 1.632 | 1.44613E-11 | up |
| MELO3C027992.2 | 8 | 0 | -5.595 | 0.000779781 | down |
| MELO3C027998.2 | 6 | 0 | -5.247 | 0.007406911 | down |
| MELO3C028009.2 | 3 | 23 | 2.82 | 0.000165908 | up |
| MELO3C028023.2 | 5 | 16 | 1.704 | 0.02033677 | up |
| MELO3C028032.2 | 0 | 13 | 6.057 | 6.56415E-05 | up |
| MELO3C028033.2 | 24 | 0 | -6.196 | 4.22927E-06 | down |
| MELO3C028034.2 | 0 | 5 | 4.526 | 0.023609069 | up |
| MELO3C028036.2 | 1 | 69 | 6.591 | 2.70745E-09 | up |
| MELO3C028069.2 | 27 | 1 | -5.463 | 6.5069E-06 | down |
| MELO3C028086.2 | 6 | 42 | 2.697 | 0.002128257 | up |
| MELO3C028091.2 | 5 | 0 | -4.875 | 0.011795629 | down |
| MELO3C028104.2 | 0 | 25 | 7.014 | 2.58204E-07 | up |
| MELO3C028107.2 | 4 | 29 | 2.74 | 0.000134592 | up |
| MELO3C028114.2 | 66 | 32 | -1.078 | 0.002714845 | down |
| MELO3C028117.2 | 168 | 10 | -4.129 | 3.27764E-30 | down |
| MELO3C028118.2 | 15 | 0 | -6.431 | 2.66699E-05 | down |
| MELO3C028121.2 | 186 | 30 | -2.645 | 2.25504E-09 | down |
| MELO3C028139.2 | 45 | 2 | -4.845 | 2.63649E-08 | down |
| MELO3C028153.2 | 0 | 22 | 6.814 | 6.61391E-07 | up |
| MELO3C028155.2 | 6 | 0 | -4.111 | 0.01882307 | down |
| MELO3C028176.2 | 32 | 0 | -7.559 | 1.36108E-08 | down |
| MELO3C028190.2 | 1 | 12 | 3.531 | 0.003357257 | up |
| MELO3C028211.2 | 14 | 0 | -6.424 | 3.19873E-05 | down |
| MELO3C028235.2 | 22 | 6 | -1.844 | 0.021027684 | down |
| MELO3C028251.2 | 30 | 1 | -5.635 | 1.45344E-06 | down |
| MELO3C028262.2 | 24 | 1 | -4.272 | 5.44645E-06 | down |
| MELO3C028271.2 | 0 | 28 | 6.191 | 3.24199E-05 | up |
| MELO3C028274.2 | 2 | 11 | 2.324 | 0.024025159 | up |
| MELO3C028281.2 | 829 | 0 | -11.286 | 1.78249E-21 | down |
| MELO3C028301.2 | 9 | 52 | 2.548 | 6.22649E-07 | up |
| MELO3C028402.2 | 154 | 0 | -9.82 | 1.21738E-15 | down |
| MELO3C028422.2 | 23 | 0 | -7.047 | 2.82383E-07 | down |
| MELO3C028427.2 | 0 | 15 | 5.317 | 0.000218916 | up |
| MELO3C028430.2 | 0 | 5 | 4.562 | 0.019626181 | up |
| MELO3C028431.2 | 0 | 5 | 4.588 | 0.023884103 | up |
| MELO3C028442.2 | 0 | 44 | 7.789 | 1.97243E-09 | up |
| MELO3C028443.2 | 0 | 6 | 4.88 | 0.008249967 | up |
| MELO3C028446.2 | 0 | 7 | 4.276 | 0.017880082 | up |
| MELO3C028449.2 | 850 | 324 | -1.392 | 1.10817E-16 | down |
| MELO3C028458.2 | 220 | 11 | -4.25 | 5.57496E-30 | down |
| MELO3C028469.2 | 0 | 17 | 5.481 | 9.1115E-05 | up |
| MELO3C028471.2 | 4 | 104 | 4.598 | 2.68861E-18 | up |
| MELO3C028475.2 | 0 | 7 | 4.273 | 0.012035985 | up |
| MELO3C028482.2 | 9 | 25 | 1.485 | 0.024587523 | up |
| MELO3C028484.2 | 0 | 49 | 7.952 | 5.2385E-10 | up |
| MELO3C028492.2 | 59 | 24 | -1.281 | 0.002205577 | down |
| MELO3C028498.2 | 52 | 25 | -1.029 | 0.022705238 | down |
| MELO3C028503.2 | 335 | 0 | -10.941 | 5.719E-20 | down |
| MELO3C028506.2 | 33 | 0 | -6.615 | 4.49983E-07 | down |
| MELO3C028507.2 | 343 | 1 | -9.12 | 2.54456E-18 | down |
| MELO3C028511.2 | 39 | 18 | -1.114 | 0.012040339 | down |
| MELO3C028513.2 | 45 | 7 | -2.596 | 1.88451E-06 | down |
| MELO3C028515.2 | 0 | 51 | 8.02 | 3.81209E-10 | up |
| MELO3C028519.2 | 25 | 0 | -7.209 | 2.65521E-06 | down |
| MELO3C028526.2 | 3 | 23 | 2.814 | 0.005915017 | up |
| MELO3C028530.2 | 118 | 0 | -9.436 | 1.16193E-14 | down |
| MELO3C028531.2 | 187 | 0 | -10.102 | 6.88718E-17 | down |
| MELO3C028533.2 | 0 | 12 | 4.937 | 0.001974263 | up |
| MELO3C028535.2 | 0 | 624 | 11.627 | 2.18775E-22 | up |
| MELO3C028540.2 | 38 | 1 | -4.685 | 2.77E-05 | down |
| MELO3C028543.2 | 0 | 56 | 8.136 | 9.89742E-11 | up |
| MELO3C028547.2 | 7 | 35 | 2.434 | 0.00029075 | up |
| MELO3C028550.2 | 3 | 20 | 2.654 | 0.002403213 | up |
| MELO3C028558.2 | 52 | 1 | -5.82 | 1.82635E-09 | down |
| MELO3C028562.2 | 22 | 0 | -7.001 | 3.65597E-07 | down |
| MELO3C028563.2 | 0 | 44 | 6.831 | 1.2837E-07 | up |
| MELO3C028577.2 | 104 | 40 | -1.396 | 5.71014E-06 | down |
| MELO3C028584.2 | 60 | 11 | -2.46 | 1.63602E-07 | down |
| MELO3C028593.2 | 56 | 0 | -8.365 | 3.09864E-11 | down |
| MELO3C028594.2 | 22 | 57 | 1.371 | 0.001917336 | up |
| MELO3C028595.2 | 9 | 22 | 1.34 | 0.023547726 | up |
| MELO3C028634.2 | 9 | 1 | -2.819 | 0.024473774 | down |
| MELO3C028648.2 | 24 | 127 | 2.408 | 7.43613E-15 | up |
| MELO3C028652.2 | 34 | 0 | -7.655 | 1.02852E-08 | down |
| MELO3C028661.2 | 1 | 21 | 3.883 | 8.6913E-05 | up |
| MELO3C028666.2 | 52 | 4 | -3.807 | 1.7436E-10 | down |
| MELO3C028682.2 | 0 | 24 | 6.901 | 4.26213E-07 | up |
| MELO3C028701.2 | 0 | 901 | 12.156 | 1.08801E-24 | up |
| MELO3C028713.2 | 0 | 24 | 6.946 | 6.00975E-06 | up |
| MELO3C028749.2 | 91 | 200 | 1.141 | 6.43359E-06 | up |
| MELO3C028763.2 | 0 | 10 | 5.682 | 0.000590775 | up |
| MELO3C028764.2 | 1 | 136 | 6.569 | 3.11055E-17 | up |
| MELO3C028773.2 | 2 | 29 | 3.552 | 1.8138E-05 | up |
| MELO3C028774.2 | 9 | 33 | 1.871 | 0.006896932 | up |
| MELO3C028779.2 | 137 | 0 | -9.653 | 2.88825E-15 | down |
| MELO3C028786.2 | 0 | 278 | 10.459 | 2.34597E-18 | up |
| MELO3C028788.2 | 0 | 50 | 7.992 | 1.83427E-09 | up |
| MELO3C028796.2 | 12 | 0 | -6.084 | 0.021861953 | down |
| MELO3C028797.2 | 1201 | 2852 | 1.248 | 3.07564E-29 | up |
| MELO3C028821.2 | 6 | 0 | -5.16 | 0.004619225 | down |
| MELO3C028836.2 | 23 | 47 | 1.047 | 0.020471833 | up |
| MELO3C028845.2 | 76 | 36 | -1.074 | 0.002437501 | down |
| MELO3C028848.2 | 15 | 106 | 2.811 | 9.728E-13 | up |
| MELO3C028852.2 | 233 | 63 | -1.873 | 6.15551E-11 | down |
| MELO3C028860.2 | 545 | 2810 | 2.365 | 8.8861E-55 | up |
| MELO3C028864.2 | 7 | 0 | -5.391 | 0.002574052 | down |
| MELO3C028865.2 | 0 | 19 | 6.568 | 1.27842E-05 | up |
| MELO3C028873.2 | 5 | 39 | 2.873 | 5.22725E-06 | up |
| MELO3C028881.2 | 37 | 0 | -7.772 | 3.54549E-09 | down |
| MELO3C028889.2 | 120 | 434 | 1.861 | 4.30721E-24 | up |
| MELO3C028898.2 | 17 | 42 | 1.334 | 0.02522806 | up |
| MELO3C028906.2 | 41 | 0 | -6.941 | 6.16382E-08 | down |
| MELO3C028921.2 | 1 | 29 | 5.338 | 8.05202E-06 | up |
| MELO3C028928.2 | 25 | 52 | 1.084 | 0.013758524 | up |
| MELO3C028959.2 | 191 | 13 | -3.915 | 8.21482E-32 | down |
| MELO3C028965.2 | 137 | 30 | -2.215 | 6.5288E-10 | down |
| MELO3C028967.2 | 0 | 66 | 8.381 | 7.5069E-10 | up |
| MELO3C028973.2 | 1 | 10 | 3.775 | 0.008908107 | up |
| MELO3C028983.2 | 7 | 29 | 2.078 | 0.004813017 | up |
| MELO3C029003.2 | 40 | 171 | 2.109 | 3.3353E-15 | up |
| MELO3C029009.2 | 5 | 0 | -4.974 | 0.008660429 | down |
| MELO3C029038.2 | 11 | 32 | 1.564 | 0.002903484 | up |
| MELO3C029050.2 | 12 | 2 | -2.625 | 0.008168774 | down |
| MELO3C029054.2 | 19956 | 1044 | -4.256 | 0 | down |
| MELO3C029067.2 | 26 | 0 | -6.279 | 2.63013E-06 | down |
| MELO3C029078.2 | 0 | 12 | 5.942 | 0.000100304 | up |
| MELO3C029083.2 | 545 | 209 | -1.384 | 3.40424E-23 | down |
| MELO3C029084.2 | 1 | 46 | 5.424 | 2.09899E-08 | up |
| MELO3C029093.2 | 29 | 80 | 1.462 | 0.000129562 | up |
| MELO3C029112.2 | 0 | 354 | 10.808 | 1.64202E-19 | up |
| MELO3C029121.2 | 15 | 35 | 1.19 | 0.012590215 | up |
| MELO3C029122.2 | 0 | 21 | 6.698 | 1.48368E-06 | up |
| MELO3C029124.2 | 0 | 220 | 10.122 | 3.89233E-17 | up |
| MELO3C029138.2 | 20 | 1 | -4.093 | 0.000239056 | down |
| MELO3C029140.2 | 5 | 0 | -4.976 | 0.008159099 | down |
| MELO3C029144.2 | 99 | 37 | -1.44 | 8.41244E-07 | down |
| MELO3C029147.2 | 0 | 61 | 8.261 | 6.11526E-11 | up |
| MELO3C029148.2 | 165 | 0 | -9.919 | 2.78688E-16 | down |
| MELO3C029161.2 | 288 | 0 | -10.722 | 3.77594E-19 | down |
| MELO3C029162.2 | 41 | 2 | -4.474 | 9.46673E-09 | down |
| MELO3C029163.2 | 0 | 363 | 10.846 | 9.14528E-20 | up |
| MELO3C029164.2 | 0 | 23 | 6.888 | 4.10137E-06 | up |
| MELO3C029165.2 | 4 | 0 | -4.772 | 0.014880115 | down |
| MELO3C029166.2 | 389 | 2940 | 2.918 | 4.75702E-163 | up |
| MELO3C029167.2 | 233 | 9 | -4.656 | 2.0714E-42 | down |
| MELO3C029171.2 | 3 | 73 | 4.513 | 1.46686E-11 | up |
| MELO3C029173.2 | 0 | 5 | 4.654 | 0.015637619 | up |
| MELO3C029178.2 | 22 | 178 | 2.98 | 5.67704E-22 | up |
| MELO3C029185.2 | 22 | 238 | 3.472 | 5.71803E-36 | up |
| MELO3C029189.2 | 1 | 12 | 3.078 | 0.00704064 | up |
| MELO3C029196.2 | 0 | 39 | 7.642 | 3.55447E-09 | up |
| MELO3C029197.2 | 1246 | 14 | -6.52 | 3.19956E-119 | down |
| MELO3C029198.2 | 230 | 0 | -9.436 | 1.17679E-14 | down |
| MELO3C029199.2 | 0 | 1275 | 12.656 | 1.52862E-26 | up |
| MELO3C029217.2 | 0 | 11 | 5.774 | 0.000202685 | up |
| MELO3C029218.2 | 15 | 1 | -4.004 | 0.000540757 | down |
| MELO3C029228.2 | 14 | 1 | -3.188 | 0.003347451 | down |
| MELO3C029249.2 | 380 | 161 | -1.231 | 1.12923E-11 | down |
| MELO3C029252.2 | 84 | 8 | -3.398 | 9.8463E-14 | down |
| MELO3C029257.2 | 54 | 148 | 1.463 | 7.35152E-06 | up |
| MELO3C029261.2 | 33 | 15 | -1.11 | 0.026172418 | down |
| MELO3C029265.2 | 73 | 29 | -1.34 | 0.001994248 | down |
| MELO3C029266.2 | 4 | 145 | 4.962 | 4.02778E-24 | up |
| MELO3C029267.2 | 0 | 178 | 9.819 | 4.2924E-16 | up |
| MELO3C029269.2 | 13 | 36 | 1.434 | 0.004239861 | up |
| MELO3C029276.2 | 1 | 11 | 3.321 | 0.006937739 | up |
| MELO3C029279.2 | 37 | 294 | 3.011 | 1.9746E-30 | up |
| MELO3C029287.2 | 1 | 900 | 10.297 | 1.3938E-23 | up |
| MELO3C029292.2 | 74 | 0 | -8.769 | 6.49687E-12 | down |
| MELO3C029297.2 | 1 | 40 | 5.8 | 7.79354E-07 | up |
| MELO3C029298.2 | 18 | 4 | -2.344 | 0.006331423 | down |
| MELO3C029299.2 | 300 | 669 | 1.156 | 8.50877E-05 | up |
| MELO3C029301.2 | 85 | 16 | -2.417 | 5.57996E-10 | down |
| MELO3C029304.2 | 26 | 137 | 2.416 | 1.70159E-13 | up |
| MELO3C029313.2 | 48 | 16 | -1.589 | 0.000340452 | down |
| MELO3C029317.2 | 235 | 8 | -4.97 | 7.94141E-19 | down |
| MELO3C029341.2 | 1749 | 251 | -2.798 | 3.52874E-06 | down |
| MELO3C029344.2 | 58 | 247 | 2.078 | 2.12319E-14 | up |
| MELO3C029352.2 | 301 | 984 | 1.71 | 0.003645973 | up |
| MELO3C029354.2 | 2 | 11 | 2.323 | 0.025968805 | up |
| MELO3C029371.2 | 1 | 11 | 2.944 | 0.01019191 | up |
| MELO3C029378.2 | 3 | 59 | 4.202 | 2.66783E-11 | up |
| MELO3C029383.2 | 4 | 0 | -4.531 | 0.029352181 | down |
| MELO3C029386.2 | 33 | 4 | -2.978 | 0.000210569 | down |
| MELO3C029414.2 | 1 | 15 | 4.392 | 0.000812964 | up |
| MELO3C029430.2 | 204 | 44 | -2.205 | 4.43942E-13 | down |
| MELO3C029441.2 | 11 | 947 | 6.464 | 4.92599E-102 | up |
| MELO3C029447.2 | 6 | 0 | -5.238 | 0.005493431 | down |
| MELO3C029472.2 | 1 | 21 | 3.883 | 0.000388157 | up |
| MELO3C029477.2 | 0 | 23 | 6.843 | 5.94374E-07 | up |
| MELO3C029487.2 | 0 | 33 | 6.437 | 8.98727E-07 | up |
| MELO3C029494.2 | 109 | 14 | -3.006 | 8.65123E-16 | down |
| MELO3C029503.2 | 0 | 12 | 5.917 | 0.000115529 | up |
| MELO3C029517.2 | 0 | 246 | 10.283 | 9.35398E-18 | up |
| MELO3C029522.2 | 17 | 2 | -3.204 | 0.001123478 | down |
| MELO3C029529.2 | 4447 | 1950 | -1.19 | 3.79221E-11 | down |
| MELO3C029544.2 | 0 | 127 | 9.33 | 1.88201E-14 | up |
| MELO3C029549.2 | 0 | 542 | 11.421 | 7.15523E-22 | up |
| MELO3C029550.2 | 1 | 318 | 8.794 | 5.53834E-17 | up |
| MELO3C029551.2 | 0 | 17 | 6.464 | 6.15958E-06 | up |
| MELO3C029553.2 | 11 | 77 | 2.755 | 4.37979E-09 | up |
| MELO3C029558.2 | 8 | 139 | 4.082 | 2.12256E-21 | up |
| MELO3C029559.2 | 0 | 249 | 10.301 | 8.78491E-18 | up |
| MELO3C029572.2 | 4 | 36 | 3.05 | 1.60951E-05 | up |
| MELO3C029575.2 | 365 | 70 | -2.37 | 3.05388E-28 | down |
| MELO3C029576.2 | 1 | 44 | 5.359 | 2.50641E-07 | up |
| MELO3C029580.2 | 10 | 1 | -3.398 | 0.007334033 | down |
| MELO3C029581.2 | 58 | 1 | -6.558 | 4.6332E-09 | down |
| MELO3C029585.2 | 0 | 5 | 4.805 | 0.01845043 | up |
| MELO3C029588.2 | 0 | 11 | 5.742 | 0.000331423 | up |
| MELO3C029590.2 | 464 | 0 | -11.411 | 8.8689E-22 | down |
| MELO3C029591.2 | 1296 | 3 | -8.581 | 1.58665E-80 | down |
| MELO3C029598.2 | 0 | 6 | 4.914 | 0.008730428 | up |
| MELO3C029616.2 | 1 | 9 | 3.011 | 0.02253803 | up |
| MELO3C029630.2 | 0 | 744 | 11.88 | 2.93308E-23 | up |
| MELO3C029631.2 | 174 | 431 | 1.315 | 3.23402E-13 | up |
| MELO3C029633.2 | 0 | 7 | 4.171 | 0.013322598 | up |
| MELO3C029634.2 | 60 | 28 | -1.131 | 0.001481344 | down |
| MELO3C029646.2 | 1 | 41 | 5.851 | 4.89272E-07 | up |
| MELO3C029647.2 | 0 | 32 | 7.345 | 2.22544E-08 | up |
| MELO3C029677.2 | 0 | 14 | 6.132 | 5.63615E-05 | up |
| MELO3C029689.2 | 273 | 131 | -1.068 | 3.32417E-06 | down |
| MELO3C029692.2 | 0 | 1097 | 12.439 | 9.24523E-26 | up |
| MELO3C029695.2 | 256 | 779 | 1.607 | 1.92557E-21 | up |
| MELO3C029717.2 | 0 | 5 | 4.546 | 0.020104157 | up |
| MELO3C029720.2 | 0 | 11 | 5.856 | 0.000278707 | up |
| MELO3C029737.2 | 6 | 0 | -5.237 | 0.005284499 | down |
| MELO3C029738.2 | 10 | 670 | 6.064 | 5.87615E-73 | up |
| MELO3C029746.2 | 0 | 119 | 9.234 | 4.53718E-14 | up |
| MELO3C029752.2 | 642 | 299 | -1.102 | 9.49331E-20 | down |
| MELO3C029753.2 | 780 | 368 | -1.088 | 6.97245E-07 | down |
| MELO3C029757.2 | 19 | 51 | 1.458 | 0.000335307 | up |
| MELO3C029765.2 | 16 | 0 | -6.499 | 1.13628E-05 | down |
| MELO3C029775.2 | 26 | 148 | 2.501 | 1.0652E-12 | up |
| MELO3C029795.2 | 0 | 10 | 5.737 | 0.000251498 | up |
| MELO3C029796.2 | 0 | 2162 | 13.418 | 7.42483E-30 | up |
| MELO3C029803.2 | 53 | 13 | -2.034 | 3.72923E-05 | down |
| MELO3C029825.2 | 1548 | 639 | -1.278 | 1.94181E-14 | down |
| MELO3C029831.2 | 1099 | 313 | -1.811 | 8.59911E-45 | down |
| MELO3C029852.2 | 5 | 20 | 1.79 | 0.008758354 | up |
| MELO3C029875.2 | 508 | 143 | -1.829 | 5.99858E-30 | down |
| MELO3C029895.2 | 2 | 16 | 2.675 | 0.006154516 | up |
| MELO3C029900.2 | 115 | 42 | -1.465 | 8.87627E-06 | down |
| MELO3C029915.2 | 0 | 10 | 5.693 | 0.00043596 | up |
| MELO3C029917.2 | 0 | 12 | 5.901 | 0.000150535 | up |
| MELO3C029922.2 | 24 | 0 | -7.171 | 1.00999E-06 | down |
| MELO3C029930.2 | 0 | 1284 | 12.665 | 1.87355E-26 | up |
| MELO3C029934.2 | 26 | 51 | 1.002 | 0.0232415 | up |
| MELO3C029936.2 | 185 | 2 | -6.423 | 5.247E-24 | down |
| MELO3C029940.2 | 1 | 17 | 4.563 | 0.0004604 | up |
| MELO3C029951.2 | 6 | 130 | 4.496 | 9.22377E-19 | up |
| MELO3C029968.2 | 87 | 2 | -5.822 | 5.90054E-15 | down |
| MELO3C029975.2 | 5 | 0 | -4.872 | 0.014761363 | down |
| MELO3C029991.2 | 0 | 1383 | 12.773 | 3.99662E-27 | up |
| MELO3C029997.2 | 28 | 1 | -4.49 | 1.48842E-06 | down |
| MELO3C030014.2 | 15 | 0 | -6.495 | 8.72787E-06 | down |
| MELO3C030023.2 | 2 | 185 | 6.692 | 1.17006E-21 | up |
| MELO3C030026.2 | 0 | 269 | 9.447 | 3.03145E-15 | up |
| MELO3C030037.2 | 0 | 35 | 7.481 | 1.49254E-08 | up |
| MELO3C030048.2 | 5 | 0 | -4.97 | 0.014769496 | down |
| MELO3C030060.2 | 284 | 970 | 1.769 | 2.03566E-32 | up |
| MELO3C030084.2 | 35 | 356 | 3.324 | 2.14602E-36 | up |
| MELO3C030102.2 | 131 | 38 | -1.779 | 2.19344E-07 | down |
| MELO3C030103.2 | 42 | 155 | 1.901 | 4.07952E-07 | up |
| MELO3C030106.2 | 32 | 132 | 2.063 | 1.07041E-11 | up |
| MELO3C030110.2 | 0 | 752 | 11.894 | 1.36166E-23 | up |
| MELO3C030113.2 | 1 | 491 | 9.421 | 7.66569E-20 | up |
| MELO3C030114.2 | 82 | 177 | 1.108 | 2.51861E-07 | up |
| MELO3C030119.2 | 7628 | 22659 | 1.571 | 2.65535E-118 | up |
| MELO3C030123.2 | 2 | 64 | 4.669 | 9.99366E-12 | up |
| MELO3C030125.2 | 35 | 0 | -7.701 | 4.52257E-09 | down |
| MELO3C030129.2 | 30 | 100 | 1.775 | 2.77203E-08 | up |
| MELO3C030131.2 | 0 | 980 | 12.277 | 3.84421E-25 | up |
| MELO3C030135.2 | 0 | 52 | 8.046 | 1.94904E-10 | up |
| MELO3C030145.2 | 32 | 1 | -5.115 | 3.58576E-07 | down |
| MELO3C030151.2 | 273 | 0 | -10.646 | 7.51915E-19 | down |
| MELO3C030163.2 | 0 | 33 | 7.394 | 1.68601E-08 | up |
| MELO3C030167.2 | 102 | 245 | 1.271 | 0.006279298 | up |
| MELO3C030175.2 | 23 | 5 | -2.321 | 0.001290649 | down |
| MELO3C030178.2 | 83 | 190 | 1.189 | 4.31291E-07 | up |
| MELO3C030184.2 | 0 | 70 | 8.464 | 3.16442E-11 | up |
| MELO3C030203.2 | 0 | 7 | 5.228 | 0.002163783 | up |
| MELO3C030204.2 | 32 | 1 | -5.689 | 1.53435E-06 | down |
| MELO3C030219.2 | 25 | 54 | 1.079 | 0.00955133 | up |
| MELO3C030221.2 | 19 | 96 | 2.31 | 4.40541E-11 | up |
| MELO3C030226.2 | 31 | 0 | -7.479 | 1.85466E-08 | down |
| MELO3C030228.2 | 0 | 978 | 12.275 | 4.98364E-25 | up |
| MELO3C030262.2 | 5 | 0 | -4.974 | 0.008660429 | down |
| MELO3C030271.2 | 9 | 32 | 1.815 | 0.002463593 | up |
| MELO3C030276.2 | 1 | 115 | 7.331 | 6.18013E-12 | up |
| MELO3C030280.2 | 9 | 29 | 1.63 | 0.01098068 | up |
| MELO3C030318.2 | 4 | 17 | 1.899 | 0.016311102 | up |
| MELO3C030319.2 | 256 | 715 | 1.48 | 1.6036E-18 | up |
| MELO3C030326.2 | 24 | 6 | -1.968 | 0.00314269 | down |
| MELO3C030333.2 | 0 | 7 | 4.132 | 0.013114135 | up |
| MELO3C030339.2 | 4777 | 979 | -2.286 | 4.38701E-25 | down |
| MELO3C030348.2 | 17 | 0 | -5.661 | 7.58038E-05 | down |
| MELO3C030349.2 | 69 | 0 | -7.697 | 1.72005E-09 | down |
| MELO3C030350.2 | 1845 | 1 | -11.546 | 7.48165E-29 | down |
| MELO3C030351.2 | 96 | 0 | -9.133 | 2.64293E-13 | down |
| MELO3C030359.2 | 151 | 0 | -8.83 | 3.52225E-13 | down |
| MELO3C030360.2 | 205 | 0 | -10.23 | 1.71261E-17 | down |
| MELO3C030365.2 | 8 | 0 | -4.631 | 0.004101651 | down |
| MELO3C030368.2 | 160 | 415 | 1.376 | 1.73844E-11 | up |
| MELO3C030370.2 | 0 | 30 | 7.241 | 8.34855E-08 | up |
| MELO3C030375.2 | 10 | 86 | 3.052 | 1.23361E-10 | up |
| MELO3C030383.2 | 4 | 0 | -4.531 | 0.029352181 | down |
| MELO3C030392.2 | 3 | 17 | 2.295 | 0.003033917 | up |
| MELO3C030396.2 | 496 | 5 | -6.494 | 1.64136E-55 | down |
| MELO3C030414.2 | 115 | 293 | 1.353 | 4.27695E-12 | up |
| MELO3C030429.2 | 195 | 27 | -2.848 | 1.60352E-23 | down |
| MELO3C030434.2 | 157 | 72 | -1.133 | 1.56274E-06 | down |
| MELO3C030436.2 | 72 | 1 | -6.871 | 5.88884E-10 | down |
| MELO3C030437.2 | 9 | 0 | -5.719 | 0.000756595 | down |
| MELO3C030440.2 | 0 | 108 | 9.093 | 1.2483E-13 | up |
| MELO3C030464.2 | 209 | 595 | 1.508 | 1.5732E-23 | up |
| MELO3C030470.2 | 84 | 30 | -1.504 | 3.32263E-05 | down |
| MELO3C030479.2 | 12 | 1 | -4.216 | 0.002538121 | down |
| MELO3C030491.2 | 1 | 756 | 10.044 | 2.48714E-22 | up |
| MELO3C030492.2 | 0 | 62 | 8.304 | 3.8608E-11 | up |
| MELO3C030496.2 | 189 | 6 | -5.095 | 1.2621E-33 | down |
| MELO3C030512.2 | 49 | 0 | -8.178 | 1.4665E-10 | down |
| MELO3C030522.2 | 2616 | 460 | -2.508 | 5.96853E-159 | down |
| MELO3C030532.2 | 24 | 1 | -4.323 | 3.34401E-05 | down |
| MELO3C030568.2 | 1 | 20 | 4.232 | 0.000175371 | up |
| MELO3C030576.2 | 1063 | 1 | -10.169 | 1.42668E-33 | down |
| MELO3C030600.2 | 0 | 24 | 6.89 | 4.08284E-07 | up |
| MELO3C030602.2 | 0 | 24 | 6.924 | 8.50859E-07 | up |
| MELO3C030606.2 | 274 | 19 | -3.821 | 1.15587E-45 | down |
| MELO3C030635.2 | 20 | 113 | 2.525 | 8.12813E-10 | up |
| MELO3C030650.2 | 0 | 5 | 4.731 | 0.012341966 | up |
| MELO3C030651.2 | 0 | 2333 | 13.528 | 2.66158E-30 | up |
| MELO3C030665.2 | 3 | 33 | 3.205 | 1.40372E-06 | up |
| MELO3C030668.2 | 249 | 23 | -3.456 | 1.61302E-14 | down |
| MELO3C030672.2 | 1 | 100 | 6.536 | 1.38996E-12 | up |
| MELO3C030675.2 | 0 | 737 | 11.866 | 1.55542E-23 | up |
| MELO3C030676.2 | 525 | 10 | -5.739 | 6.64083E-79 | down |
| MELO3C030688.2 | 32 | 85 | 1.424 | 5.98565E-05 | up |
| MELO3C030695.2 | 0 | 1495 | 12.886 | 1.41446E-27 | up |
| MELO3C030704.2 | 479 | 194 | -1.303 | 5.27886E-16 | down |
| MELO3C030711.2 | 69 | 16 | -2.081 | 1.20058E-05 | down |
| MELO3C030719.2 | 821 | 239 | -1.783 | 8.59461E-20 | down |
| MELO3C030723.2 | 0 | 933 | 12.206 | 8.14809E-25 | up |
| MELO3C030724.2 | 8 | 0 | -5.66 | 0.001944757 | down |
| MELO3C030730.2 | 3 | 20 | 2.835 | 0.001590419 | up |
| MELO3C030737.2 | 1041 | 3002 | 1.528 | 1.21679E-41 | up |
| MELO3C030740.2 | 9 | 47 | 2.455 | 3.08451E-06 | up |
| MELO3C030745.2 | 0 | 17 | 6.46 | 1.44035E-05 | up |
| MELO3C030747.2 | 12 | 119 | 3.324 | 2.43922E-15 | up |
| MELO3C030748.2 | 5 | 102 | 4.34 | 2.07316E-18 | up |
| MELO3C030751.2 | 1 | 9 | 3.122 | 0.023853877 | up |
| MELO3C030758.2 | 0 | 41 | 7.684 | 3.89919E-09 | up |
| MELO3C030768.2 | 0 | 90 | 8.825 | 6.76315E-13 | up |
| MELO3C030769.2 | 0 | 41 | 7.7 | 3.22842E-09 | up |
| MELO3C030770.2 | 164 | 18 | -3.142 | 1.81017E-16 | down |
| MELO3C030784.2 | 96 | 0 | -9.138 | 1.9272E-13 | down |
| MELO3C030787.2 | 11 | 40 | 1.906 | 0.000656759 | up |
| MELO3C030789.2 | 0 | 34 | 7.424 | 1.30023E-08 | up |
| MELO3C030795.2 | 0 | 572 | 11.5 | 3.90662E-22 | up |
| MELO3C030799.2 | 13 | 137 | 3.412 | 5.62217E-15 | up |
| MELO3C030800.2 | 136 | 30 | -2.193 | 1.13906E-07 | down |
| MELO3C030828.2 | 704 | 0 | -12.013 | 4.67878E-24 | down |
| MELO3C030836.2 | 17 | 2 | -3.258 | 0.000490945 | down |
| MELO3C030845.2 | 0 | 24 | 6.947 | 3.94959E-07 | up |
| MELO3C030854.2 | 4 | 101 | 4.683 | 4.56831E-14 | up |
| MELO3C030856.2 | 0 | 469 | 11.213 | 5.55058E-21 | up |
| MELO3C030859.2 | 299 | 131 | -1.185 | 1.73684E-10 | down |
| MELO3C030860.2 | 22 | 3 | -3.153 | 0.000152096 | down |
| MELO3C030862.2 | 0 | 12 | 5.911 | 0.00013053 | up |
| MELO3C030865.2 | 93 | 282 | 1.606 | 1.81099E-16 | up |
| MELO3C030868.2 | 1 | 12 | 3.065 | 0.029204293 | up |
| MELO3C030869.2 | 6 | 398 | 6.031 | 7.62203E-52 | up |
| MELO3C030870.2 | 0 | 6 | 4.988 | 0.005629698 | up |
| MELO3C030882.2 | 14 | 3 | -2.404 | 0.020560803 | down |
| MELO3C030893.2 | 256 | 75 | -1.78 | 3.832E-19 | down |
| MELO3C030897.2 | 14 | 513 | 5.199 | 9.35869E-74 | up |
| MELO3C030898.2 | 1 | 13 | 3.194 | 0.011185383 | up |
| MELO3C030900.2 | 31 | 0 | -7.496 | 2.73368E-08 | down |
| MELO3C030902.2 | 234 | 0 | -10.421 | 3.59456E-18 | down |
| MELO3C030927.2 | 2 | 16 | 2.692 | 0.004503444 | up |
| MELO3C030936.2 | 208 | 48 | -2.102 | 2.42885E-11 | down |
| MELO3C030939.2 | 0 | 8 | 5.365 | 0.001426773 | up |
| MELO3C030950.2 | 144 | 29 | -2.307 | 9.21162E-16 | down |
| MELO3C030959.2 | 536 | 216 | -1.307 | 7.51379E-16 | down |
| MELO3C030960.2 | 19 | 45 | 1.284 | 0.005195882 | up |
| MELO3C030966.2 | 153 | 306 | 1.001 | 3.27464E-08 | up |
| MELO3C030968.2 | 8 | 166 | 4.393 | 4.67947E-29 | up |
| MELO3C030971.2 | 1 | 10 | 3.799 | 0.008332147 | up |
| MELO3C030979.2 | 31 | 13 | -1.186 | 0.015929331 | down |
| MELO3C030980.2 | 4 | 0 | -4.769 | 0.015590779 | down |
| MELO3C030992.2 | 8 | 54 | 2.846 | 1.15656E-08 | up |
| MELO3C031014.2 | 1657 | 239 | -2.787 | 6.52319E-64 | down |
| MELO3C031024.2 | 134 | 428 | 1.674 | 5.78256E-22 | up |
| MELO3C031032.2 | 24 | 55 | 1.196 | 0.005393582 | up |
| MELO3C031036.2 | 17 | 40 | 1.21 | 0.007061656 | up |
| MELO3C031050.2 | 85 | 183 | 1.097 | 2.48756E-05 | up |
| MELO3C031052.2 | 0 | 172 | 9.766 | 1.07646E-15 | up |
| MELO3C031054.2 | 4 | 19 | 2.238 | 0.012626277 | up |
| MELO3C031055.2 | 4 | 25 | 2.459 | 0.001128154 | up |
| MELO3C031059.2 | 0 | 5 | 4.766 | 0.012169983 | up |
| MELO3C031065.2 | 2 | 12 | 2.702 | 0.02281288 | up |
| MELO3C031071.2 | 1156 | 201 | -2.524 | 2.03787E-36 | down |
| MELO3C031072.2 | 1050 | 2386 | 1.185 | 8.33501E-26 | up |
| MELO3C031083.2 | 171 | 418 | 1.29 | 2.87408E-13 | up |
| MELO3C031091.2 | 6 | 45 | 2.888 | 2.70451E-06 | up |
| MELO3C031122.2 | 128 | 442 | 1.789 | 2.06512E-20 | up |
| MELO3C031125.2 | 529 | 0 | -11.601 | 1.73E-22 | down |
| MELO3C031130.2 | 49 | 0 | -8.157 | 3.64998E-10 | down |
| MELO3C031131.2 | 37 | 0 | -7.744 | 2.74653E-09 | down |
| MELO3C031140.2 | 0 | 9 | 4.59 | 0.004572932 | up |
| MELO3C031169.2 | 83 | 231 | 1.469 | 1.3765E-06 | up |
| MELO3C031170.2 | 19 | 44 | 1.255 | 0.011693583 | up |
| MELO3C031184.2 | 40 | 3 | -3.555 | 3.59938E-08 | down |
| MELO3C031189.2 | 1 | 11 | 3.285 | 0.009176035 | up |
| MELO3C031210.2 | 5 | 0 | -4.872 | 0.013952132 | down |
| MELO3C031214.2 | 566 | 2 | -8.262 | 8.49383E-42 | down |
| MELO3C031216.2 | 0 | 34 | 7.428 | 1.30757E-08 | up |
| MELO3C031219.2 | 278 | 53 | -2.397 | 9.43937E-24 | down |
| MELO3C031220.2 | 16 | 4 | -1.979 | 0.024630039 | down |
| MELO3C031235.2 | 8 | 0 | -5.52 | 0.004008832 | down |
| MELO3C031239.2 | 205 | 656 | 1.678 | 9.274E-09 | up |
| MELO3C031246.2 | 20 | 2 | -3.172 | 0.001708926 | down |
| MELO3C031269.2 | 88 | 0 | -9.008 | 3.58335E-13 | down |
| MELO3C031290.2 | 1 | 15 | 3.374 | 0.003103133 | up |
| MELO3C031294.2 | 0 | 81 | 8.673 | 2.75645E-12 | up |
| MELO3C031304.2 | 10 | 0 | -4.915 | 0.00134732 | down |
| MELO3C031322.2 | 3 | 1394 | 8.606 | 1.93823E-72 | up |
| MELO3C031323.2 | 268 | 906 | 1.754 | 1.98797E-20 | up |
| MELO3C031324.2 | 16 | 47 | 1.554 | 0.000681353 | up |
| MELO3C031330.2 | 1229 | 365 | -1.752 | 3.82496E-14 | down |
| MELO3C031333.2 | 23 | 71 | 1.65 | 0.001224305 | up |
| MELO3C031334.2 | 1 | 19 | 3.715 | 0.001110389 | up |
| MELO3C031339.2 | 278 | 592 | 1.089 | 4.34679E-14 | up |
| MELO3C031340.2 | 18 | 0 | -5.78 | 3.68672E-05 | down |
| MELO3C031341.2 | 248 | 0 | -10.507 | 2.25962E-18 | down |
| MELO3C031344.2 | 21 | 3 | -2.993 | 0.000728337 | down |
| MELO3C031345.2 | 7 | 0 | -5.314 | 0.002878903 | down |
| MELO3C031355.2 | 7 | 32 | 2.1 | 0.000578315 | up |
| MELO3C031371.2 | 25 | 0 | -7.179 | 3.31455E-07 | down |
| MELO3C031372.2 | 147 | 0 | -9.752 | 8.89813E-16 | down |
| MELO3C031379.2 | 80 | 31 | -1.353 | 3.84375E-05 | down |
| MELO3C031382.2 | 44 | 10 | -2.071 | 4.56395E-05 | down |
| MELO3C031387.2 | 0 | 6 | 3.988 | 0.019473547 | up |
| MELO3C031395.2 | 0 | 6 | 4.846 | 0.010713516 | up |
| MELO3C031411.2 | 0 | 15 | 6.241 | 2.06829E-05 | up |
| MELO3C031416.2 | 0 | 29 | 7.188 | 6.76224E-08 | up |
| MELO3C031417.2 | 0 | 9 | 5.424 | 0.001306487 | up |
| MELO3C031425.2 | 22 | 8 | -1.44 | 0.021771888 | down |
| MELO3C031435.2 | 0 | 8 | 5.454 | 0.000938947 | up |
| MELO3C031437.2 | 50 | 0 | -8.186 | 2.27424E-10 | down |
| MELO3C031442.2 | 19 | 2 | -3.38 | 0.000148892 | down |
| MELO3C031443.2 | 54 | 7 | -2.925 | 8.97656E-08 | down |
| MELO3C031450.2 | 0 | 13 | 6.028 | 6.44111E-05 | up |
| MELO3C031452.2 | 400 | 192 | -1.067 | 7.84713E-07 | down |
| MELO3C031472.2 | 0 | 282 | 10.478 | 1.90569E-18 | up |
| MELO3C031476.2 | 10 | 26 | 1.373 | 0.017619204 | up |
| MELO3C031484.2 | 14 | 3 | -2.157 | 0.013562116 | down |
| MELO3C031486.2 | 0 | 28 | 6.199 | 2.50082E-05 | up |
| MELO3C031487.2 | 1145 | 1 | -10.859 | 4.21063E-26 | down |
| MELO3C031496.2 | 1 | 30 | 4.401 | 2.49444E-06 | up |
| MELO3C031498.2 | 23 | 54 | 1.246 | 0.00308866 | up |
| MELO3C031513.2 | 1 | 8 | 2.987 | 0.026318507 | up |
| MELO3C031541.2 | 1 | 8 | 2.962 | 0.022202763 | up |
| MELO3C031543.2 | 1 | 650 | 9.242 | 1.1118E-27 | up |
| MELO3C031548.2 | 30 | 84 | 1.48 | 4.71793E-05 | up |
| MELO3C031556.2 | 0 | 7 | 5.171 | 0.002734299 | up |
| MELO3C031557.2 | 2 | 23 | 3.651 | 0.000195916 | up |
| MELO3C031564.2 | 9 | 106 | 3.564 | 4.74955E-17 | up |
| MELO3C031600.2 | 76 | 215 | 1.501 | 5.37484E-11 | up |
| MELO3C031601.2 | 539 | 5608 | 3.378 | 1.91833E-98 | up |
| MELO3C031604.2 | 268 | 638 | 1.25 | 1.21659E-10 | up |
| MELO3C031605.2 | 8 | 45 | 2.429 | 0.000211874 | up |
| MELO3C031616.2 | 34 | 5 | -2.772 | 0.000101692 | down |
| MELO3C031618.2 | 25 | 75 | 1.587 | 3.2688E-05 | up |
| MELO3C031621.2 | 4 | 27 | 2.895 | 0.000502778 | up |
| MELO3C031622.2 | 0 | 4 | 4.464 | 0.025033946 | up |
| MELO3C031623.2 | 6 | 24 | 2.068 | 0.006989592 | up |
| MELO3C031644.2 | 7 | 25 | 1.887 | 0.00217509 | up |
| MELO3C031645.2 | 0 | 129 | 9.354 | 1.68672E-14 | up |
| MELO3C031662.2 | 24 | 144 | 2.563 | 8.56856E-19 | up |
| MELO3C031667.2 | 4 | 88 | 4.346 | 1.61581E-10 | up |
| MELO3C031670.2 | 561 | 136 | -2.048 | 8.89986E-13 | down |
| MELO3C031675.2 | 1 | 20 | 4.221 | 0.00011688 | up |
| MELO3C031717.2 | 909 | 0 | -12.38 | 1.55499E-25 | down |
| MELO3C031722.2 | 4 | 20 | 2.212 | 0.002139689 | up |
| MELO3C031755.2 | 269 | 20 | -3.712 | 2.08959E-44 | down |
| MELO3C031769.2 | 27 | 7 | -2.004 | 0.001083247 | down |
| MELO3C031779.2 | 2 | 218 | 6.666 | 3.70451E-24 | up |
| MELO3C031780.2 | 47 | 530 | 3.516 | 8.27734E-18 | up |
| MELO3C031782.2 | 4 | 0 | -4.653 | 0.021136749 | down |
| MELO3C031785.2 | 1 | 13 | 3.164 | 0.004098601 | up |
| MELO3C031801.2 | 36 | 156 | 2.108 | 8.71289E-16 | up |
| MELO3C031806.2 | 0 | 188 | 9.89 | 3.13104E-16 | up |
| MELO3C031809.2 | 0 | 3791 | 14.229 | 3.21197E-33 | up |
| MELO3C031812.2 | 2 | 245 | 6.609 | 7.34256E-27 | up |
| MELO3C031820.2 | 17 | 68 | 2.061 | 9.04822E-07 | up |
| MELO3C031824.2 | 217 | 100 | -1.109 | 2.44326E-07 | down |
| MELO3C031839.2 | 0 | 49 | 7.965 | 5.44881E-10 | up |
| MELO3C031840.2 | 1 | 31 | 4.838 | 2.62622E-06 | up |
| MELO3C031857.2 | 3656 | 254 | -3.842 | 4.60733E-61 | down |
| MELO3C031858.2 | 59 | 191 | 1.69 | 3.06232E-10 | up |
| MELO3C031859.2 | 1075 | 148 | -2.86 | 1.80868E-52 | down |
| MELO3C031860.2 | 54 | 114 | 1.093 | 2.16488E-05 | up |
| MELO3C031876.2 | 544 | 1 | -8.788 | 1.37036E-32 | down |
| MELO3C031893.2 | 26 | 1 | -4.809 | 9.82353E-06 | down |
| MELO3C031894.2 | 2520 | 0 | -13.852 | 1.04284E-31 | down |
| MELO3C031895.2 | 1415 | 0 | -13.019 | 4.81142E-28 | down |
| MELO3C031896.2 | 981 | 0 | -12.49 | 5.44447E-26 | down |
| MELO3C031906.2 | 0 | 30 | 7.255 | 6.12325E-08 | up |
| MELO3C031924.2 | 2827 | 1232 | -1.199 | 1.0078E-27 | down |
| MELO3C031926.2 | 0 | 69 | 8.454 | 2.36962E-11 | up |
| MELO3C031927.2 | 1 | 9 | 3.069 | 0.02065993 | up |
| MELO3C031934.2 | 0 | 5 | 4.519 | 0.027611386 | up |
| MELO3C031942.2 | 0 | 86 | 8.768 | 6.01203E-11 | up |
| MELO3C031943.2 | 0 | 31 | 7.284 | 8.78581E-08 | up |
| MELO3C031945.2 | 20 | 2 | -3.204 | 0.000144993 | down |
| MELO3C031947.2 | 0 | 113 | 9.168 | 5.46556E-14 | up |
| MELO3C031963.2 | 0 | 6 | 4.942 | 0.008700863 | up |
| MELO3C031967.2 | 5 | 0 | -4.978 | 0.008598989 | down |
| MELO3C031972.2 | 0 | 366 | 10.857 | 1.00926E-19 | up |
| MELO3C032000.2 | 42 | 96 | 1.208 | 4.31296E-05 | up |
| MELO3C032024.2 | 3 | 13 | 2.23 | 0.024169766 | up |
| MELO3C032026.2 | 7 | 0 | -5.396 | 0.002123705 | down |
| MELO3C032043.2 | 4 | 16 | 1.774 | 0.018120372 | up |
| MELO3C032048.2 | 8 | 23 | 1.529 | 0.014404067 | up |
| MELO3C032050.2 | 6 | 20 | 1.718 | 0.028635844 | up |
| MELO3C032061.2 | 313 | 774 | 1.308 | 3.4082E-17 | up |
| MELO3C032067.2 | 0 | 19 | 6.601 | 3.69105E-06 | up |
| MELO3C032074.2 | 18 | 55 | 1.63 | 5.29157E-05 | up |
| MELO3C032083.2 | 5 | 0 | -4.872 | 0.013952132 | down |
| MELO3C032089.2 | 246 | 29 | -3.107 | 7.40418E-38 | down |
| MELO3C032097.2 | 71 | 11 | -2.751 | 6.39241E-06 | down |
| MELO3C032108.2 | 0 | 45 | 7.85 | 9.19527E-10 | up |
| MELO3C032112.2 | 109 | 3 | -5.142 | 2.01083E-20 | down |
| MELO3C032130.2 | 20 | 0 | -6.876 | 8.87466E-07 | down |
| MELO3C032143.2 | 144 | 55 | -1.392 | 6.31984E-06 | down |
| MELO3C032145.2 | 3 | 23 | 2.867 | 0.00075391 | up |
| MELO3C032146.2 | 65 | 154 | 1.262 | 5.60186E-07 | up |
| MELO3C032149.2 | 0 | 54 | 8.103 | 1.30884E-10 | up |
| MELO3C032169.2 | 15 | 0 | -5.502 | 0.000126133 | down |
| MELO3C032178.2 | 0 | 38 | 7.592 | 4.25807E-09 | up |
| MELO3C032187.2 | 549 | 228 | -1.269 | 6.12353E-06 | down |
| MELO3C032192.2 | 7 | 50 | 2.753 | 3.0999E-08 | up |
| MELO3C032203.2 | 14 | 0 | -6.318 | 2.5567E-05 | down |
| MELO3C032207.2 | 2 | 27 | 3.663 | 0.000871796 | up |
| MELO3C032208.2 | 0 | 16 | 6.313 | 1.85191E-05 | up |
| MELO3C032211.2 | 0 | 5 | 4.62 | 0.017451245 | up |
| MELO3C032227.2 | 0 | 16 | 6.3 | 1.48506E-05 | up |
| MELO3C032269.2 | 148 | 10 | -3.897 | 4.23181E-20 | down |
| MELO3C032271.2 | 0 | 22 | 6.795 | 1.17991E-06 | up |
| MELO3C032281.2 | 0 | 384 | 10.925 | 7.22942E-20 | up |
| MELO3C032285.2 | 15 | 93 | 2.586 | 9.27828E-11 | up |
| MELO3C032290.2 | 0 | 4 | 4.478 | 0.029433373 | up |
| MELO3C032295.2 | 3 | 34 | 3.391 | 2.11046E-06 | up |
| MELO3C032302.2 | 178 | 51 | -1.81 | 8.9714E-11 | down |
| MELO3C032307.2 | 85 | 0 | -8.959 | 3.55281E-13 | down |
| MELO3C032309.2 | 90 | 184 | 1.036 | 9.34092E-06 | up |
| MELO3C032312.2 | 8 | 177 | 4.558 | 2.26721E-29 | up |
| MELO3C032315.2 | 748 | 262 | -1.515 | 3.89555E-21 | down |
| MELO3C032319.2 | 12 | 0 | -6.07 | 9.34879E-05 | down |
| MELO3C032320.2 | 25 | 84 | 1.758 | 3.54318E-06 | up |
| MELO3C032323.2 | 32 | 82 | 1.338 | 0.000335725 | up |
| MELO3C032330.2 | 272 | 101 | -1.425 | 0.000103195 | down |
| MELO3C032345.2 | 10 | 3302 | 8.411 | 3.46162E-37 | up |
| MELO3C032348.2 | 284 | 56 | -2.352 | 3.65076E-23 | down |
| MELO3C032352.2 | 5 | 19 | 1.832 | 0.012131052 | up |
| MELO3C032353.2 | 91 | 9 | -3.317 | 1.00229E-12 | down |
| MELO3C032356.2 | 3 | 17 | 2.428 | 0.002957402 | up |
| MELO3C032359.2 | 7 | 68 | 3.26 | 4.03034E-08 | up |
| MELO3C032387.2 | 132 | 0 | -9.59 | 2.32507E-14 | down |
| MELO3C032396.2 | 13 | 59 | 2.16 | 0.0002634 | up |
| MELO3C032401.2 | 28 | 72 | 1.391 | 6.22523E-05 | up |
| MELO3C032410.2 | 0 | 28 | 6.199 | 4.18322E-06 | up |
| MELO3C032437.2 | 0 | 128 | 9.341 | 1.79408E-14 | up |
| MELO3C032441.2 | 6 | 29 | 2.315 | 0.000344551 | up |
| MELO3C032442.2 | 2 | 560 | 8.026 | 2.65695E-38 | up |
| MELO3C032454.2 | 1590 | 291 | -2.448 | 1.85968E-86 | down |
| MELO3C032461.2 | 413 | 1379 | 1.741 | 2.11925E-46 | up |
| MELO3C032467.2 | 8 | 136 | 4.181 | 2.53976E-21 | up |
| MELO3C032468.2 | 0 | 11 | 5.838 | 0.000201331 | up |
| MELO3C032469.2 | 0 | 10 | 5.631 | 0.000551887 | up |
| MELO3C032471.2 | 129 | 264 | 1.036 | 3.56995E-09 | up |
| MELO3C032497.2 | 12 | 436 | 5.208 | 6.82732E-64 | up |
| MELO3C032499.2 | 124 | 61 | -1.02 | 0.000567665 | down |
| MELO3C032507.2 | 156 | 75 | -1.054 | 3.21187E-06 | down |
| MELO3C032516.2 | 664 | 247 | -1.426 | 2.2282E-28 | down |
| MELO3C032523.2 | 22 | 1 | -4.548 | 0.000154182 | down |
| MELO3C032530.2 | 25 | 4 | -2.576 | 0.000587581 | down |
| MELO3C032537.2 | 68 | 1 | -6.787 | 6.86822E-10 | down |
| MELO3C032579.2 | 2 | 96 | 5.489 | 5.32666E-13 | up |
| MELO3C032593.2 | 55 | 243 | 2.132 | 9.5213E-13 | up |
| MELO3C032602.2 | 202 | 1054 | 2.387 | 1.37875E-33 | up |
| MELO3C032603.2 | 238 | 834 | 1.811 | 3.17469E-27 | up |
| MELO3C032607.2 | 248 | 101 | -1.296 | 2.25029E-10 | down |
| MELO3C032612.2 | 5 | 0 | -4.985 | 0.015316583 | down |
| MELO3C032621.2 | 26 | 1 | -5.35 | 2.63014E-05 | down |
| MELO3C032630.2 | 270 | 69 | -1.96 | 7.86705E-09 | down |
| MELO3C032635.2 | 92 | 2 | -5.632 | 4.22855E-16 | down |
| MELO3C032637.2 | 9 | 0 | -5.774 | 0.000567839 | down |
| MELO3C032639.2 | 180 | 529 | 1.559 | 4.92257E-21 | up |
| MELO3C032661.2 | 0 | 2197 | 13.441 | 8.53406E-30 | up |
| MELO3C032662.2 | 0 | 55 | 8.138 | 1.33846E-10 | up |
| MELO3C032663.2 | 36396 | 14526 | -1.325 | 6.93004E-31 | down |
| MELO3C032665.2 | 0 | 53 | 7.11 | 2.01501E-08 | up |
| MELO3C032674.2 | 9 | 0 | -5.77 | 0.000646805 | down |
| MELO3C032689.2 | 0 | 63 | 7.364 | 4.22337E-09 | up |
| MELO3C032690.2 | 0 | 50 | 7.987 | 3.27415E-10 | up |
| MELO3C032692.2 | 18 | 179 | 3.263 | 6.30071E-20 | up |
| MELO3C032707.2 | 49 | 0 | -8.173 | 7.43068E-10 | down |
| MELO3C032717.2 | 0 | 7 | 4.221 | 0.009885851 | up |
| MELO3C032718.2 | 0 | 137 | 9.432 | 1.0542E-14 | up |
| MELO3C032731.2 | 92 | 0 | -9.07 | 2.63871E-13 | down |
| MELO3C032733.2 | 36 | 0 | -7.74 | 3.2812E-09 | down |
| MELO3C032739.2 | 712 | 3 | -7.875 | 3.51413E-19 | down |
| MELO3C032740.2 | 0 | 72 | 8.499 | 7.66658E-12 | up |
| MELO3C032750.2 | 282 | 132 | -1.1 | 3.08098E-10 | down |
| MELO3C032760.2 | 7 | 0 | -4.354 | 0.008879485 | down |
| MELO3C032766.2 | 15 | 670 | 5.445 | 3.18654E-93 | up |
| MELO3C032776.2 | 0 | 13 | 6.059 | 5.73991E-05 | up |
| MELO3C032778.2 | 16 | 63 | 1.981 | 8.99268E-06 | up |
| MELO3C032779.2 | 11 | 60 | 2.536 | 2.75249E-08 | up |
| MELO3C032784.2 | 158 | 4 | -5.421 | 3.7045E-27 | down |
| MELO3C032787.2 | 48 | 6 | -2.896 | 6.48955E-06 | down |
| MELO3C032807.2 | 14 | 39 | 1.446 | 0.001452795 | up |
| MELO3C032813.2 | 8 | 0 | -4.506 | 0.007463347 | down |
| MELO3C032833.2 | 4 | 24 | 2.366 | 0.000539565 | up |
| MELO3C032841.2 | 1575 | 8533 | 2.438 | 6.47974E-60 | up |
| MELO3C032847.2 | 229 | 57 | -2.001 | 1.11847E-16 | down |
| MELO3C032850.2 | 28 | 10 | -1.472 | 0.012854855 | down |
| MELO3C032851.2 | 25 | 6 | -2.094 | 0.000725902 | down |
| MELO3C032853.2 | 5 | 0 | -4.015 | 0.028520029 | down |
| MELO3C032857.2 | 1010 | 459 | -1.138 | 1.43669E-29 | down |
| MELO3C032862.2 | 80 | 244 | 1.607 | 6.14665E-12 | up |
| MELO3C032869.2 | 2 | 12 | 2.443 | 0.014546116 | up |
| MELO3C032879.2 | 43 | 111 | 1.376 | 4.41462E-05 | up |
| MELO3C032881.2 | 299 | 0 | -10.777 | 7.70642E-18 | down |
| MELO3C032882.2 | 66 | 6 | -3.514 | 1.15331E-09 | down |
| MELO3C032883.2 | 4 | 37 | 2.989 | 3.20727E-06 | up |
| MELO3C032888.2 | 11 | 44 | 1.975 | 0.000223567 | up |
| MELO3C032889.2 | 230 | 513 | 1.163 | 4.73396E-15 | up |
| MELO3C032896.2 | 0 | 4 | 4.478 | 0.029429592 | up |
| MELO3C032903.2 | 46 | 17 | -1.461 | 0.000874747 | down |
| MELO3C032910.2 | 291 | 76 | -1.919 | 3.21237E-10 | down |
| MELO3C032917.2 | 139 | 2 | -6.487 | 5.65349E-17 | down |
| MELO3C032939.2 | 10 | 1 | -4.073 | 0.007457628 | down |
| MELO3C032940.2 | 0 | 7 | 5.152 | 0.002969781 | up |
| MELO3C032943.2 | 1 | 29 | 5.353 | 1.05889E-05 | up |
| MELO3C032950.2 | 1 | 53 | 5.615 | 3.3716E-08 | up |
| MELO3C032955.2 | 603 | 244 | -1.309 | 5.52016E-21 | down |
| MELO3C032956.2 | 0 | 6 | 5.01 | 0.005331757 | up |
| MELO3C032967.2 | 1 | 348 | 8.341 | 8.8128E-21 | up |
| MELO3C032975.2 | 1121 | 2682 | 1.258 | 1.22205E-18 | up |
| MELO3C032980.2 | 0 | 8 | 5.282 | 0.00174027 | up |
| MELO3C032996.2 | 0 | 56 | 8.165 | 1.76383E-10 | up |
| MELO3C033009.2 | 5 | 0 | -5.069 | 0.00585854 | down |
| MELO3C033019.2 | 10 | 52 | 2.264 | 1.31943E-05 | up |
| MELO3C033024.2 | 72 | 13 | -2.448 | 2.22446E-09 | down |
| MELO3C033047.2 | 0 | 5 | 4.69 | 0.019669045 | up |
| MELO3C033048.2 | 0 | 411 | 11.02 | 3.3164E-20 | up |
| MELO3C033051.2 | 68 | 0 | -8.649 | 5.33334E-12 | down |
| MELO3C033070.2 | 0 | 72 | 8.505 | 7.08078E-12 | up |
| MELO3C033071.2 | 0 | 12 | 5.985 | 9.00485E-05 | up |
| MELO3C033082.2 | 51 | 118 | 1.203 | 2.33388E-05 | up |
| MELO3C033086.2 | 6 | 27 | 2.143 | 0.001704421 | up |
| MELO3C033094.2 | 8 | 1950 | 7.958 | 1.6118E-28 | up |
| MELO3C033095.2 | 39 | 8403 | 7.757 | 5.78166E-34 | up |
| MELO3C033110.2 | 8 | 1 | -3.735 | 0.017179666 | down |
| MELO3C033117.2 | 7 | 0 | -5.4 | 0.005130837 | down |
| MELO3C033123.2 | 0 | 72 | 8.502 | 7.50322E-12 | up |
| MELO3C033124.2 | 0 | 710 | 11.811 | 2.97237E-23 | up |
| MELO3C033125.2 | 17 | 1 | -4.238 | 0.00014044 | down |
| MELO3C033129.2 | 16 | 35 | 1.101 | 0.024154461 | up |
| MELO3C033155.2 | 0 | 49 | 7.955 | 3.81938E-10 | up |
| MELO3C033165.2 | 0 | 18 | 6.538 | 5.0184E-06 | up |
| MELO3C033192.2 | 330 | 113 | -1.545 | 6.48124E-15 | down |
| MELO3C033195.2 | 4 | 18 | 2.306 | 0.018769636 | up |
| MELO3C033197.2 | 402 | 2312 | 2.525 | 7.54803E-08 | up |
| MELO3C033219.2 | 0 | 404 | 10.036 | 4.63603E-17 | up |
| MELO3C033220.2 | 0 | 122 | 9.267 | 2.56407E-14 | up |
| MELO3C033228.2 | 0 | 28 | 7.134 | 4.3556E-07 | up |
| MELO3C033230.2 | 829 | 137 | -2.593 | 5.64816E-72 | down |
| MELO3C033241.2 | 0 | 320 | 9.7 | 8.02857E-16 | up |
| MELO3C033242.2 | 31 | 0 | -7.498 | 1.76987E-08 | down |
| MELO3C033262.2 | 64 | 138 | 1.107 | 0.000582147 | up |
| MELO3C033272.2 | 7 | 1 | -3.556 | 0.023429673 | down |
| MELO3C033291.2 | 0 | 194 | 9.942 | 4.90075E-16 | up |
| MELO3C033295.2 | 142 | 12 | -3.559 | 2.95227E-15 | down |
| MELO3C033300.2 | 386 | 77 | -2.317 | 5.33618E-15 | down |
| MELO3C033313.2 | 0 | 25 | 6.95 | 1.13273E-06 | up |
| MELO3C033314.2 | 48 | 0 | -8.133 | 3.11855E-10 | down |
| MELO3C033316.2 | 189 | 0 | -10.114 | 3.64269E-16 | down |
| MELO3C033317.2 | 278 | 1 | -8.812 | 1.57459E-16 | down |
| MELO3C033318.2 | 8 | 0 | -5.589 | 0.001087653 | down |
| MELO3C033320.2 | 45 | 0 | -8.043 | 2.45754E-09 | down |
| MELO3C033331.2 | 0 | 8 | 4.337 | 0.009095468 | up |
| MELO3C033346.2 | 0 | 171 | 9.761 | 5.5726E-16 | up |
| MELO3C033349.2 | 0 | 6 | 4.835 | 0.01094688 | up |
| MELO3C033351.2 | 0 | 33 | 7.358 | 3.42056E-08 | up |
| MELO3C033356.2 | 124 | 0 | -9.501 | 7.4076E-15 | down |
| MELO3C033365.2 | 0 | 6 | 4.991 | 0.008101538 | up |
| MELO3C033367.2 | 33 | 12 | -1.501 | 0.004573445 | down |
| MELO3C033370.2 | 0 | 334 | 10.724 | 2.98828E-19 | up |
| MELO3C033375.2 | 25 | 83 | 1.714 | 0.000496557 | up |
| MELO3C033377.2 | 0 | 6 | 4.976 | 0.00545292 | up |
| MELO3C033382.2 | 44 | 101 | 1.216 | 0.000129307 | up |
| MELO3C033388.2 | 35 | 87 | 1.29 | 2.31043E-05 | up |
| MELO3C033392.2 | 13 | 3 | -1.968 | 0.015978963 | down |
| MELO3C033394.2 | 814 | 1 | -9.049 | 4.62001E-42 | down |
| MELO3C033416.2 | 1040 | 1 | -10.137 | 3.71958E-33 | down |
| MELO3C033427.2 | 16 | 5 | -1.636 | 0.028796059 | down |
| MELO3C033455.2 | 0 | 139 | 9.461 | 1.01384E-14 | up |
| MELO3C033461.2 | 0 | 233 | 10.207 | 2.16367E-17 | up |
| MELO3C033464.2 | 0 | 425 | 11.071 | 1.97227E-20 | up |
| MELO3C033482.2 | 316 | 107 | -1.563 | 4.49344E-14 | down |
| MELO3C033491.2 | 0 | 12 | 5.897 | 0.000138573 | up |
| MELO3C033516.2 | 6 | 0 | -5.069 | 0.012456256 | down |
| MELO3C033520.2 | 3194 | 1454 | -1.136 | 1.74513E-27 | down |
| MELO3C033530.2 | 0 | 21 | 5.767 | 2.3394E-05 | up |
| MELO3C033536.2 | 44 | 297 | 2.76 | 3.09032E-10 | up |
| MELO3C033539.2 | 86 | 39 | -1.138 | 0.000273141 | down |
| MELO3C033542.2 | 3 | 22 | 2.765 | 0.005506504 | up |
| MELO3C033561.2 | 6 | 0 | -5.161 | 0.005331937 | down |
| MELO3C033564.2 | 0 | 15 | 6.253 | 1.73341E-05 | up |
| MELO3C033567.2 | 14 | 71 | 2.392 | 3.80198E-07 | up |
| MELO3C033578.2 | 39 | 1 | -5.019 | 1.9164E-07 | down |
| MELO3C033596.2 | 7 | 23 | 1.754 | 0.006464929 | up |
| MELO3C033603.2 | 2 | 273 | 7.253 | 1.07094E-26 | up |
| MELO3C033627.2 | 137 | 20 | -2.767 | 1.31339E-14 | down |
| MELO3C033665.2 | 77 | 24 | -1.681 | 6.13036E-06 | down |
| MELO3C033689.2 | 0 | 54 | 8.078 | 3.43751E-10 | up |
| MELO3C033692.2 | 8 | 44 | 2.42 | 0.000256618 | up |
| MELO3C033697.2 | 6 | 39 | 2.681 | 1.76218E-05 | up |
| MELO3C033732.2 | 33 | 10 | -1.72 | 0.004155366 | down |
| MELO3C033764.2 | 26 | 0 | -7.253 | 4.07102E-07 | down |
| MELO3C033790.2 | 271 | 56 | -2.294 | 2.87788E-08 | down |
| MELO3C033802.2 | 4 | 39 | 3.295 | 1.34511E-06 | up |
| MELO3C033835.2 | 0 | 8 | 5.356 | 0.003255261 | up |
| MELO3C033871.2 | 23 | 54 | 1.198 | 0.021465149 | up |
| MELO3C033885.2 | 92 | 10 | -3.215 | 7.52249E-15 | down |
| MELO3C033886.2 | 0 | 45 | 7.827 | 1.14338E-09 | up |
| MELO3C033896.2 | 0 | 69 | 8.453 | 1.04876E-11 | up |
| MELO3C033898.2 | 493 | 212 | -1.217 | 6.50148E-17 | down |
| MELO3C033902.2 | 146 | 53 | -1.461 | 1.90446E-09 | down |
| MELO3C033904.2 | 33 | 780 | 4.568 | 1.02498E-81 | up |
| MELO3C033910.2 | 4 | 0 | -4.773 | 0.015904137 | down |
| MELO3C033914.2 | 194 | 409 | 1.073 | 5.15348E-13 | up |
| MELO3C033933.2 | 10 | 29 | 1.495 | 0.019491259 | up |
| MELO3C033937.2 | 120 | 32 | -1.928 | 1.5761E-11 | down |
| MELO3C033939.2 | 28 | 176 | 2.633 | 2.52291E-08 | up |
| MELO3C033946.2 | 0 | 128 | 9.346 | 1.91868E-14 | up |
| MELO3C033947.2 | 0 | 50 | 8 | 2.91854E-10 | up |
| MELO3C033952.2 | 92 | 0 | -9.081 | 1.45624E-13 | down |
| MELO3C033954.2 | 3 | 408 | 6.984 | 4.38023E-42 | up |
| MELO3C033955.2 | 8 | 25 | 1.726 | 0.004900355 | up |
| MELO3C033957.2 | 39 | 108 | 1.474 | 9.83044E-06 | up |
| MELO3C033959.2 | 0 | 5 | 4.591 | 0.021426906 | up |
| MELO3C033960.2 | 0 | 77 | 8.598 | 3.56445E-12 | up |
| MELO3C033966.2 | 56 | 4 | -3.907 | 4.22106E-11 | down |
| MELO3C033967.2 | 244 | 588 | 1.271 | 3.53559E-11 | up |
| MELO3C033971.2 | 12 | 171 | 3.768 | 3.87447E-24 | up |
| MELO3C033988.2 | 1755 | 39 | -5.497 | 9.6557E-86 | down |
| MELO3C033989.2 | 31 | 3 | -3.373 | 5.34005E-05 | down |
| MELO3C033992.2 | 56 | 222 | 1.98 | 1.1415E-14 | up |
| MELO3C034004.2 | 26 | 9 | -1.532 | 0.010119486 | down |
| MELO3C034006.2 | 133 | 58 | -1.206 | 3.66066E-05 | down |
| MELO3C034009.2 | 271 | 660 | 1.281 | 1.25239E-14 | up |
| MELO3C034013.2 | 443 | 2 | -7.497 | 1.95729E-40 | down |
| MELO3C034020.2 | 0 | 8 | 4.334 | 0.007104956 | up |
| MELO3C034022.2 | 9 | 0 | -4.753 | 0.002380776 | down |
| MELO3C034023.2 | 9143 | 2402 | -1.929 | 1.22751E-54 | down |
| MELO3C034027.2 | 155 | 45 | -1.796 | 4.88898E-06 | down |
| MELO3C034038.2 | 68 | 26 | -1.344 | 0.011517916 | down |
| MELO3C034040.2 | 0 | 6 | 5.013 | 0.006985604 | up |
| MELO3C034045.2 | 154 | 14 | -3.386 | 1.50452E-20 | down |
| MELO3C034084.2 | 0 | 153 | 9.604 | 2.3375E-15 | up |
| MELO3C034093.2 | 5 | 0 | -4.971 | 0.011053116 | down |
| MELO3C034100.2 | 111 | 18 | -2.62 | 8.11853E-14 | down |
| MELO3C034110.2 | 75 | 198 | 1.394 | 6.14716E-09 | up |
| MELO3C034116.2 | 446 | 148 | -1.585 | 3.72532E-12 | down |
| MELO3C034119.2 | 5503 | 1062 | -2.374 | 3.15966E-52 | down |
| MELO3C034128.2 | 256 | 78 | -1.715 | 3.81265E-17 | down |
| MELO3C034129.2 | 272 | 77 | -1.826 | 2.63839E-18 | down |
| MELO3C034131.2 | 26 | 4 | -2.672 | 0.001623361 | down |
| MELO3C034152.2 | 50 | 6 | -3.073 | 5.20264E-07 | down |
| MELO3C034167.2 | 32 | 105 | 1.721 | 3.91233E-06 | up |
| MELO3C034181.2 | 131 | 63 | -1.057 | 8.51789E-06 | down |
| MELO3C034190.2 | 0 | 15 | 6.308 | 2.97011E-05 | up |
| MELO3C034211.2 | 13 | 0 | -6.199 | 4.14118E-05 | down |
| MELO3C034234.2 | 9 | 1 | -3.337 | 0.022962813 | down |
| MELO3C034251.2 | 297 | 1298 | 2.124 | 2.81781E-37 | up |
| MELO3C034276.2 | 0 | 132 | 9.385 | 1.03029E-14 | up |
| MELO3C034286.2 | 25 | 88 | 1.835 | 6.86784E-08 | up |
| MELO3C034288.2 | 76 | 1 | -6.95 | 2.31165E-10 | down |
| MELO3C034300.2 | 2 | 12 | 2.51 | 0.010802441 | up |
| MELO3C034301.2 | 29 | 71 | 1.287 | 0.002481191 | up |
| MELO3C034302.2 | 0 | 18 | 6.54 | 3.64376E-06 | up |
| MELO3C034306.2 | 11 | 150 | 3.753 | 2.07988E-20 | up |
| MELO3C034317.2 | 29 | 5 | -2.434 | 0.01181588 | down |
| MELO3C034330.2 | 0 | 179 | 9.824 | 4.97109E-16 | up |
| MELO3C034342.2 | 0 | 523 | 11.37 | 1.61364E-21 | up |
| MELO3C034352.2 | 235 | 0 | -10.428 | 5.75395E-12 | down |
| MELO3C034353.2 | 150 | 0 | -9.78 | 1.09243E-13 | down |
| MELO3C034361.2 | 0 | 22 | 6.761 | 1.05758E-06 | up |
| MELO3C034362.2 | 0 | 312 | 10.625 | 5.7013E-19 | up |
| MELO3C034378.2 | 1378 | 0 | -12.98 | 7.56287E-28 | down |
| MELO3C034380.2 | 0 | 46 | 7.855 | 2.73133E-09 | up |
| MELO3C034384.2 | 0 | 146 | 9.531 | 3.43113E-15 | up |
| MELO3C034386.2 | 0 | 171 | 9.758 | 6.15712E-16 | up |
| MELO3C034392.2 | 0 | 82 | 8.694 | 1.86417E-12 | up |
| MELO3C034398.2 | 363 | 0 | -11.056 | 2.24575E-20 | down |
| MELO3C034399.2 | 7 | 0 | -5.32 | 0.002472154 | down |
| MELO3C034400.2 | 34 | 0 | -7.623 | 7.73103E-09 | down |
| MELO3C034410.2 | 31 | 7 | -2.229 | 0.000907205 | down |
| MELO3C034413.2 | 5 | 0 | -4.97 | 0.014769496 | down |
| MELO3C034414.2 | 0 | 170 | 9.745 | 1.5171E-15 | up |
| MELO3C034420.2 | 336 | 0 | -10.943 | 7.98087E-20 | down |
| MELO3C034437.2 | 3 | 27 | 3.086 | 0.000806082 | up |
| MELO3C034439.2 | 0 | 65 | 8.357 | 2.05886E-11 | up |
| MELO3C034442.2 | 5 | 26 | 2.364 | 0.00266996 | up |
| MELO3C034448.2 | 1 | 22 | 4.95 | 4.21544E-05 | up |
| MELO3C034459.2 | 2 | 14 | 2.68 | 0.011051138 | up |
| MELO3C034461.2 | 126 | 12 | -3.358 | 6.49433E-21 | down |
| MELO3C034462.2 | 17 | 1 | -4.827 | 0.000292592 | down |
| MELO3C034465.2 | 46 | 12 | -1.864 | 0.000100348 | down |
| MELO3C034478.2 | 15 | 63 | 2.069 | 8.36023E-07 | up |
| MELO3C034486.2 | 243 | 96 | -1.351 | 2.20961E-07 | down |
| MELO3C034499.2 | 9 | 0 | -5.659 | 0.000725508 | down |
| MELO3C034500.2 | 3 | 17 | 2.611 | 0.004195202 | up |
| MELO3C034520.2 | 45 | 310 | 2.8 | 2.11784E-24 | up |
| MELO3C034523.2 | 7 | 26 | 1.769 | 0.002823713 | up |
| MELO3C034525.2 | 1 | 12 | 3.025 | 0.006369311 | up |
| MELO3C034527.2 | 0 | 9 | 5.522 | 0.00105976 | up |
| MELO3C034541.2 | 111 | 1 | -7.503 | 2.33473E-12 | down |
| MELO3C034544.2 | 7 | 1 | -3.54 | 0.022817258 | down |
| MELO3C034547.2 | 876 | 1 | -9.473 | 5.20549E-38 | down |
| MELO3C034553.2 | 4 | 18 | 2.187 | 0.005655898 | up |
| MELO3C034560.2 | 1252 | 0 | -12.842 | 2.29801E-27 | down |
| MELO3C034565.2 | 26 | 1 | -5.428 | 2.7154E-05 | down |
| MELO3C034581.2 | 2753 | 17 | -7.349 | 1.71983E-193 | down |
| MELO3C034589.2 | 0 | 14 | 6.165 | 2.94358E-05 | up |
| MELO3C034590.2 | 279 | 52 | -2.415 | 4.91971E-28 | down |
| MELO3C034613.2 | 28 | 422 | 3.936 | 3.6888E-59 | up |
| MELO3C034622.2 | 22 | 4 | -2.504 | 0.000389354 | down |
| MELO3C034646.2 | 44 | 103 | 1.238 | 4.37712E-05 | up |
| MELO3C034651.2 | 88 | 0 | -9.001 | 2.58432E-13 | down |
| MELO3C034657.2 | 41 | 1 | -6.087 | 1.55465E-07 | down |
| MELO3C034663.2 | 3 | 29 | 3.331 | 1.42107E-05 | up |
| MELO3C034678.2 | 46 | 392 | 3.105 | 1.54472E-20 | up |
| MELO3C034695.2 | 122 | 7 | -4.102 | 1.23497E-22 | down |
| MELO3C034701.2 | 69 | 29 | -1.245 | 0.004685861 | down |
| MELO3C034702.2 | 20 | 99 | 2.297 | 1.85342E-10 | up |
| MELO3C034703.2 | 18 | 4 | -2.321 | 0.014824741 | down |
| MELO3C034734.2 | 7 | 0 | -5.391 | 0.002179073 | down |
| MELO3C034744.2 | 0 | 41 | 7.699 | 2.12694E-09 | up |
| MELO3C034763.2 | 707 | 1529 | 1.113 | 2.33091E-19 | up |
| MELO3C034767.2 | 60 | 0 | -8.444 | 2.1175E-11 | down |
| MELO3C034780.2 | 51 | 0 | -7.259 | 9.13055E-09 | down |
| MELO3C034781.2 | 191 | 2 | -6.689 | 1.09698E-25 | down |
| MELO3C034810.2 | 76 | 0 | -8.791 | 1.48064E-12 | down |
| MELO3C034811.2 | 110 | 0 | -9.334 | 5.92785E-14 | down |
| MELO3C034813.2 | 253 | 604 | 1.256 | 5.27744E-07 | up |
| MELO3C034814.2 | 569 | 1421 | 1.32 | 4.63972E-14 | up |
| MELO3C034824.2 | 0 | 386 | 10.933 | 4.67773E-20 | up |
| MELO3C034853.2 | 4 | 0 | -4.529 | 0.028513558 | down |
| MELO3C034862.2 | 16 | 55 | 1.777 | 0.000802243 | up |
| MELO3C034872.2 | 182 | 2 | -6.883 | 2.88758E-23 | down |
| MELO3C034877.2 | 107 | 0 | -9.3 | 9.86832E-14 | down |
| MELO3C034903.2 | 0 | 309 | 10.614 | 6.1289E-19 | up |
| MELO3C034906.2 | 440 | 2 | -8.157 | 1.61063E-33 | down |
| MELO3C034915.2 | 95 | 15 | -2.648 | 8.45939E-11 | down |
| MELO3C034917.2 | 0 | 64 | 8.33 | 3.10744E-11 | up |
| MELO3C034926.2 | 560 | 159 | -1.82 | 1.17465E-22 | down |
| MELO3C034933.2 | 17 | 0 | -6.593 | 5.69514E-06 | down |
| MELO3C034941.2 | 7 | 34 | 2.3 | 0.00964051 | up |
| MELO3C034954.2 | 254 | 11 | -4.451 | 1.03006E-36 | down |
| MELO3C034955.2 | 0 | 318 | 10.653 | 9.06169E-19 | up |
| MELO3C034962.2 | 0 | 261 | 10.369 | 7.43914E-18 | up |
| MELO3C034964.2 | 0 | 19 | 6.592 | 8.38202E-06 | up |
| MELO3C034965.2 | 28 | 0 | -7.371 | 9.19888E-08 | down |
| MELO3C034966.2 | 7 | 0 | -5.313 | 0.019214555 | down |
| MELO3C034973.2 | 1551 | 594 | -1.386 | 1.50662E-41 | down |
| MELO3C034985.2 | 0 | 15 | 6.293 | 1.77278E-05 | up |
| MELO3C034995.2 | 455 | 74 | -2.631 | 5.85762E-35 | down |
| MELO3C035018.2 | 0 | 56 | 8.138 | 5.17509E-10 | up |
| MELO3C035023.2 | 123 | 416 | 1.752 | 2.93596E-24 | up |
| MELO3C035026.2 | 28 | 0 | -7.374 | 8.1894E-08 | down |
| MELO3C035031.2 | 5 | 129 | 4.69 | 2.92055E-19 | up |
| MELO3C035034.2 | 4 | 0 | -4.531 | 0.028394142 | down |
| MELO3C035044.2 | 411 | 171 | -1.265 | 1.11697E-10 | down |
| MELO3C035058.2 | 341 | 857 | 1.331 | 1.34304E-10 | up |
| MELO3C035066.2 | 125 | 512 | 2.03 | 1.71943E-27 | up |
| MELO3C035088.2 | 8 | 67 | 3.096 | 1.65835E-07 | up |
| MELO3C035091.2 | 0 | 162 | 9.679 | 1.06417E-15 | up |
| MELO3C035093.2 | 43 | 18 | -1.282 | 0.006073822 | down |
| MELO3C035114.2 | 0 | 6 | 5.067 | 0.005245004 | up |
| MELO3C035115.2 | 6 | 23 | 1.903 | 0.012513872 | up |
| MELO3C035119.2 | 0 | 107 | 9.085 | 1.10123E-13 | up |
| MELO3C035131.2 | 0 | 20 | 6.688 | 1.42147E-06 | up |
| MELO3C035133.2 | 83 | 34 | -1.298 | 0.000306964 | down |
| MELO3C035136.2 | 0 | 37 | 7.544 | 7.90186E-09 | up |
| MELO3C035139.2 | 246 | 99 | -1.305 | 5.83257E-10 | down |
| MELO3C035159.2 | 8 | 0 | -5.529 | 0.00180457 | down |
| MELO3C035177.2 | 587 | 7469 | 3.67 | 0.000329155 | up |
| MELO3C035191.2 | 9 | 34 | 2.003 | 0.000726169 | up |
| MELO3C035192.2 | 26 | 117 | 2.164 | 1.38649E-10 | up |
| MELO3C035199.2 | 46 | 635 | 3.792 | 1.41521E-35 | up |
| MELO3C035200.2 | 173 | 1 | -8.143 | 7.1778E-14 | down |
| MELO3C035201.2 | 11701 | 4 | -11.517 | 1.39743E-161 | down |
| MELO3C035229.2 | 6140 | 1180 | -2.38 | 0.00091169 | down |
| MELO3C035241.2 | 14 | 1 | -3.962 | 0.003323872 | down |
| MELO3C035242.2 | 14 | 39 | 1.504 | 0.019878094 | up |
| MELO3C035246.2 | 1774 | 767 | -1.207 | 6.94172E-11 | down |
| MELO3C035250.2 | 107 | 37 | -1.541 | 5.42865E-07 | down |
| MELO3C035256.2 | 103 | 32 | -1.697 | 1.56271E-07 | down |
| MELO3C035274.2 | 12783 | 3046 | -2.069 | 4.57494E-80 | down |
| MELO3C035281.2 | 32 | 4 | -3.096 | 2.40058E-06 | down |
| MELO3C035293.2 | 15269 | 3799 | -2.007 | 1.59973E-38 | down |
| MELO3C035299.2 | 2 | 21 | 3.07 | 0.002030644 | up |
| MELO3C035307.2 | 0 | 455 | 11.168 | 1.0555E-20 | up |
| MELO3C035318.2 | 154 | 23 | -2.733 | 3.39691E-18 | down |
| MELO3C035319.2 | 21 | 104 | 2.319 | 9.50196E-13 | up |
| MELO3C035321.2 | 64 | 23 | -1.485 | 0.000102096 | down |
| MELO3C035361.2 | 17360 | 35659 | 1.038 | 7.64115E-29 | up |
| MELO3C035367.2 | 70 | 0 | -8.683 | 8.49266E-12 | down |
| MELO3C035396.2 | 18 | 0 | -6.714 | 2.38688E-06 | down |
| MELO3C035414.2 | 0 | 1725 | 13.092 | 2.06321E-28 | up |
| MELO3C035435.2 | 7 | 204 | 4.763 | 2.81734E-23 | up |
| MELO3C035440.2 | 875 | 243 | -1.852 | 1.03407E-41 | down |
| MELO3C035452.2 | 15 | 0 | -6.464 | 1.09199E-05 | down |
| MELO3C035503.2 | 2 | 11 | 2.698 | 0.017525423 | up |
| MELO3C035515.2 | 295 | 36 | -3.027 | 4.32361E-27 | down |
| MELO3C035516.2 | 16 | 1 | -3.714 | 0.002221613 | down |
| MELO3C035517.2 | 41 | 4 | -3.467 | 5.36178E-06 | down |
| MELO3C035524.2 | 0 | 1070 | 12.404 | 1.34781E-25 | up |
| MELO3C035534.2 | 484 | 0 | -11.472 | 5.22641E-22 | down |
| MELO3C035535.2 | 752 | 4020 | 2.418 | 2.21312E-64 | up |
| MELO3C035538.2 | 5833 | 1030 | -2.5 | 6.97569E-120 | down |
| MELO3C035544.2 | 130 | 357 | 1.452 | 1.72703E-09 | up |
| MELO3C035548.2 | 0 | 21 | 6.755 | 2.81833E-06 | up |
| MELO3C035553.2 | 928 | 384 | -1.271 | 8.21798E-11 | down |
| MELO3C035555.2 | 1 | 111 | 6.277 | 2.67135E-15 | up |
| MELO3C035567.2 | 85 | 2 | -5.519 | 6.53352E-16 | down |
| MELO3C035568.2 | 137 | 2 | -6.472 | 4.18782E-20 | down |
| MELO3C035574.2 | 79 | 162 | 1.036 | 1.91872E-06 | up |
| MELO3C035575.2 | 0 | 54 | 7.13 | 1.4169E-08 | up |
| MELO3C035576.2 | 0 | 6 | 4.993 | 0.006672628 | up |
| MELO3C035583.2 | 19 | 67 | 1.807 | 1.96894E-05 | up |
| MELO3C035594.2 | 697 | 333 | -1.064 | 9.54806E-14 | down |
| MELO3C035597.2 | 0 | 16 | 6.328 | 5.85974E-05 | up |
| MELO3C035600.2 | 0 | 15 | 6.241 | 2.51228E-05 | up |
| MELO3C035604.2 | 8 | 31 | 1.92 | 0.000553586 | up |
| MELO3C035606.2 | 0 | 72 | 8.507 | 9.22676E-12 | up |
| MELO3C035611.2 | 14 | 52 | 1.857 | 7.4929E-05 | up |
| MELO3C035622.2 | 32 | 0 | -7.56 | 1.29326E-08 | down |
| MELO3C035627.2 | 1073 | 25 | -5.441 | 1.44471E-148 | down |
| MELO3C035646.2 | 28 | 0 | -7.357 | 6.66831E-08 | down |
| MELO3C035649.2 | 1 | 485 | 9.402 | 1.1152E-19 | up |
| MELO3C035650.2 | 11 | 0 | -6.027 | 0.000127742 | down |
| MELO3C035652.2 | 0 | 5 | 4.668 | 0.024260107 | up |
| MELO3C035654.2 | 0 | 32 | 7.358 | 3.30634E-08 | up |
| MELO3C035662.2 | 20 | 94 | 2.199 | 4.44567E-09 | up |
| MELO3C035676.2 | 62 | 131 | 1.073 | 0.000140388 | up |
| MELO3C035682.2 | 5 | 91 | 4.287 | 2.84744E-11 | up |
| MELO3C035683.2 | 0 | 125 | 9.303 | 2.19054E-14 | up |
| MELO3C035685.2 | 0 | 18 | 6.499 | 1.64455E-05 | up |
| MELO3C035692.2 | 3 | 20 | 2.802 | 0.000548234 | up |
| MELO3C035696.2 | 2 | 33 | 3.927 | 2.8239E-06 | up |
| MELO3C035704.2 | 214 | 0 | -10.295 | 1.41659E-17 | down |
| MELO3C035729.2 | 46 | 260 | 2.505 | 4.08316E-12 | up |
| MELO3C035730.2 | 16 | 43 | 1.46 | 0.001908164 | up |
| MELO3C035742.2 | 0 | 6 | 4.954 | 0.006814591 | up |
| MELO3C035750.2 | 21 | 1 | -4.098 | 3.27882E-05 | down |
| MELO3C035754.2 | 68 | 152 | 1.156 | 2.88019E-06 | up |
| MELO3C035757.2 | 58 | 25 | -1.226 | 0.002280669 | down |
| MELO3C035759.2 | 53 | 15 | -1.846 | 0.000489255 | down |
| MELO3C035767.2 | 34 | 93 | 1.436 | 0.001396117 | up |
| MELO3C035769.2 | 18 | 5 | -1.779 | 0.018999077 | down |
| MELO3C035770.2 | 172 | 0 | -9.976 | 2.94902E-10 | down |
| MELO3C035771.2 | 1 | 10657 | 13.862 | 7.94772E-42 | up |
| MELO3C035776.2 | 0 | 7 | 5.187 | 0.004861291 | up |
