# Supplementary material for: Comprehensive Analysis of Transcriptome and Metabolome Reveals the Flavonoid Metabolic Pathway Is Associated with Fruit Peel Coloration of Melon
Source: Molecules. 2021 May 10;26(9):2830. doi: 10.3390/molecules26092830 (PMC8126211; doi:10.3390/molecules26092830)
Supplement: Supplementary file 1 [file molecules-26-02830-s001.zip › molecules-1183709-supplementary/Table S4 FPKMs and function annotation of genes in B vs H .docx]

| **Table S4 FPKMs and function annotation of genes in B vs H** | | | | | |
| --- | --- | --- | --- | --- | --- |
| **ID** | **B** | **H** | **log2FoldChange** | **pvalue** | **regulated** |
| MELO3C000019.2 | 38 | 10 | -1.929 | 0.001368953 | down |
| MELO3C000026.2 | 13 | 1 | -4.446 | 0.001173038 | down |
| MELO3C000027.2 | 41 | 12 | -1.775 | 0.000849887 | down |
| MELO3C000030.2 | 132 | 44 | -1.563 | 9.77985E-07 | down |
| MELO3C000061.2 | 269 | 539 | 1.004 | 1.84588E-14 | up |
| MELO3C000062.2 | 428 | 1449 | 1.761 | 1.12558E-19 | up |
| MELO3C000064.2 | 92 | 0 | -9.133 | 2.39291E-13 | down |
| MELO3C000067.2 | 305 | 11 | -4.804 | 2.36221E-54 | down |
| MELO3C000076.2 | 2 | 70 | 5.232 | 9.49616E-12 | up |
| MELO3C000093.2 | 181 | 0 | -10.114 | 5.29631E-17 | down |
| MELO3C000100.2 | 29 | 5 | -2.412 | 7.08482E-05 | down |
| MELO3C000101.2 | 26 | 8 | -1.738 | 0.004482576 | down |
| MELO3C000105.2 | 13 | 1 | -3.837 | 0.001971108 | down |
| MELO3C000109.2 | 8 | 0 | -5.661 | 0.000584395 | down |
| MELO3C000111.2 | 199 | 38 | -2.38 | 6.37159E-23 | down |
| MELO3C000117.2 | 9505 | 3728 | -1.35 | 2.79027E-51 | down |
| MELO3C000123.2 | 1 | 2951 | 11.963 | 7.38503E-21 | up |
| MELO3C000130.2 | 66 | 30 | -1.151 | 0.000909212 | down |
| MELO3C000131.2 | 30 | 7 | -2.12 | 0.000828328 | down |
| MELO3C000150.2 | 23 | 0 | -7.092 | 4.21543E-07 | down |
| MELO3C000157.2 | 88 | 215 | 1.301 | 5.2343E-06 | up |
| MELO3C000161.2 | 11 | 76 | 2.76 | 8.42542E-11 | up |
| MELO3C000173.2 | 63 | 11 | -2.544 | 5.15803E-08 | down |
| MELO3C000196.2 | 185 | 56 | -1.738 | 1.53976E-11 | down |
| MELO3C000200.2 | 13 | 0 | -6.305 | 5.9204E-05 | down |
| MELO3C000201.2 | 39 | 19 | -1.069 | 0.02505844 | down |
| MELO3C000204.2 | 92 | 467 | 2.345 | 1.24771E-35 | up |
| MELO3C000213.2 | 74 | 432 | 2.545 | 7.97818E-38 | up |
| MELO3C000220.2 | 67 | 19 | -1.857 | 2.5586E-06 | down |
| MELO3C000225.2 | 0 | 14 | 6.073 | 4.58201E-05 | up |
| MELO3C000228.2 | 1987 | 161 | -3.633 | 7.13605E-195 | down |
| MELO3C000237.2 | 417 | 96 | -2.121 | 4.08622E-37 | down |
| MELO3C000241.2 | 25 | 10 | -1.307 | 0.023547544 | down |
| MELO3C000256.2 | 187 | 0 | -10.164 | 4.10273E-17 | down |
| MELO3C000277.2 | 55 | 17 | -1.696 | 0.000184599 | down |
| MELO3C000296.2 | 1 | 29 | 4.715 | 2.76753E-06 | up |
| MELO3C000311.2 | 3 | 131 | 5.639 | 1.11795E-17 | up |
| MELO3C000321.2 | 300 | 107 | -1.485 | 3.40082E-14 | down |
| MELO3C000329.2 | 1 | 8 | 3.387 | 0.025590257 | up |
| MELO3C000333.2 | 369 | 105 | -1.816 | 6.59673E-23 | down |
| MELO3C000334.2 | 45 | 12 | -1.924 | 0.000140706 | down |
| MELO3C000335.2 | 0 | 37 | 7.496 | 1.16969E-08 | up |
| MELO3C000336.2 | 31 | 3 | -3.306 | 0.001029953 | down |
| MELO3C000366.2 | 0 | 87 | 8.729 | 3.57494E-12 | up |
| MELO3C000374.2 | 0 | 6 | 4.915 | 0.009442214 | up |
| MELO3C000376.2 | 636 | 13 | -5.616 | 3.50672E-98 | down |
| MELO3C000378.2 | 102 | 636 | 2.635 | 1.18649E-40 | up |
| MELO3C000391.2 | 29 | 8 | -1.884 | 0.007068171 | down |
| MELO3C000392.2 | 94 | 1 | -6.322 | 2.93217E-15 | down |
| MELO3C000399.2 | 39 | 11 | -1.861 | 0.000120766 | down |
| MELO3C000449.2 | 65 | 18 | -1.864 | 1.39113E-06 | down |
| MELO3C000450.2 | 0 | 105 | 8.988 | 2.45945E-13 | up |
| MELO3C000483.2 | 92 | 36 | -1.337 | 9.14455E-05 | down |
| MELO3C000487.2 | 100 | 0 | -9.255 | 6.32684E-14 | down |
| MELO3C000500.2 | 15 | 3 | -2.354 | 0.013863064 | down |
| MELO3C000503.2 | 428 | 202 | -1.079 | 1.2607E-07 | down |
| MELO3C000515.2 | 9 | 1574 | 7.397 | 2.63863E-110 | up |
| MELO3C000526.2 | 35 | 109 | 1.66 | 4.99492E-08 | up |
| MELO3C000527.2 | 6 | 417 | 6.038 | 6.76992E-51 | up |
| MELO3C000583.2 | 47 | 272 | 2.535 | 4.62018E-14 | up |
| MELO3C000592.2 | 4345 | 1297 | -1.743 | 6.95886E-25 | down |
| MELO3C000601.2 | 12 | 2 | -2.558 | 0.023133197 | down |
| MELO3C000606.2 | 520 | 88 | -2.57 | 1.49503E-57 | down |
| MELO3C000609.2 | 32 | 99 | 1.635 | 0.000765505 | up |
| MELO3C000631.2 | 53 | 18 | -1.565 | 0.000983476 | down |
| MELO3C000655.2 | 283 | 612 | 1.111 | 2.47188E-14 | up |
| MELO3C000668.2 | 188 | 0 | -10.175 | 4.73933E-17 | down |
| MELO3C000776.2 | 369 | 7 | -5.724 | 6.99846E-11 | down |
| MELO3C000787.2 | 1 | 28 | 5.214 | 1.20781E-05 | up |
| MELO3C000794.2 | 73 | 0 | -8.802 | 1.41078E-12 | down |
| MELO3C000795.2 | 1 | 8 | 3.38 | 0.024833087 | up |
| MELO3C000806.2 | 165 | 0 | -9.98 | 0.010643207 | down |
| MELO3C000818.2 | 0 | 8 | 5.336 | 0.001343246 | up |
| MELO3C000822.2 | 12 | 34 | 1.495 | 0.010212095 | up |
| MELO3C000830.2 | 0 | 26 | 6.986 | 2.28459E-07 | up |
| MELO3C000849.2 | 170 | 52 | -1.714 | 7.08767E-05 | down |
| MELO3C000881.2 | 461 | 5 | -6.445 | 3.80683E-65 | down |
| MELO3C000920.2 | 5 | 0 | -4.882 | 0.013637206 | down |
| MELO3C000923.2 | 0 | 5 | 4.644 | 0.016312426 | up |
| MELO3C000966.2 | 75 | 243 | 1.686 | 2.30942E-09 | up |
| MELO3C000979.2 | 8 | 1 | -3.704 | 0.020660169 | down |
| MELO3C000994.2 | 25 | 7 | -1.816 | 0.002520973 | down |
| MELO3C001068.2 | 169 | 29 | -2.558 | 2.03432E-16 | down |
| MELO3C001088.2 | 13 | 2 | -2.726 | 0.009335662 | down |
| MELO3C001150.2 | 76 | 260 | 1.758 | 1.74812E-13 | up |
| MELO3C001160.2 | 80 | 0 | -8.95 | 3.8658E-13 | down |
| MELO3C001165.2 | 201 | 0 | -10.27 | 1.5702E-17 | down |
| MELO3C001175.2 | 262 | 701 | 1.416 | 2.46105E-13 | up |
| MELO3C001234.2 | 81 | 32 | -1.358 | 0.00127157 | down |
| MELO3C001235.2 | 70 | 15 | -2.258 | 4.62099E-08 | down |
| MELO3C001238.2 | 126 | 47 | -1.414 | 2.79558E-08 | down |
| MELO3C001253.2 | 54 | 0 | -8.367 | 3.05491E-11 | down |
| MELO3C001266.2 | 2 | 43 | 4.273 | 5.57732E-08 | up |
| MELO3C001303.2 | 132 | 53 | -1.323 | 1.33427E-05 | down |
| MELO3C001323.2 | 62 | 15 | -2.081 | 4.49571E-05 | down |
| MELO3C001354.2 | 14 | 193 | 3.768 | 1.04122E-26 | up |
| MELO3C001368.2 | 153 | 31 | -2.276 | 8.27889E-14 | down |
| MELO3C001387.2 | 0 | 5 | 4.609 | 0.013944936 | up |
| MELO3C001388.2 | 153 | 55 | -1.477 | 1.592E-08 | down |
| MELO3C001405.2 | 42 | 102 | 1.264 | 7.78199E-06 | up |
| MELO3C001461.2 | 67 | 0 | -8.693 | 0.026138018 | down |
| MELO3C001462.2 | 473 | 165 | -1.519 | 8.62908E-15 | down |
| MELO3C001489.2 | 0 | 5 | 4.583 | 0.019191883 | up |
| MELO3C001500.2 | 1 | 27 | 4.166 | 1.51924E-05 | up |
| MELO3C001553.2 | 18 | 6 | -1.648 | 0.01613998 | down |
| MELO3C001561.2 | 22 | 3 | -2.772 | 0.001391422 | down |
| MELO3C001593.2 | 10 | 0 | -4.934 | 0.001349511 | down |
| MELO3C001595.2 | 13 | 2 | -2.467 | 0.016779872 | down |
| MELO3C001609.2 | 6 | 37 | 2.643 | 9.54923E-06 | up |
| MELO3C001614.2 | 151 | 16 | -3.243 | 1.14012E-20 | down |
| MELO3C001621.2 | 0 | 25 | 6.91 | 5.89574E-07 | up |
| MELO3C001651.2 | 0 | 33 | 7.325 | 2.48098E-08 | up |
| MELO3C001656.2 | 1906 | 312 | -2.611 | 9.60943E-98 | down |
| MELO3C001657.2 | 138 | 1 | -6.55 | 5.86396E-20 | down |
| MELO3C001806.2 | 12 | 0 | -6.151 | 5.372E-05 | down |
| MELO3C001821.2 | 28 | 9 | -1.696 | 0.003123658 | down |
| MELO3C001844.2 | 40 | 1 | -5.512 | 1.82722E-07 | down |
| MELO3C001880.2 | 5 | 0 | -4.904 | 0.01542615 | down |
| MELO3C001902.2 | 0 | 156 | 9.565 | 3.39998E-15 | up |
| MELO3C001915.2 | 0 | 56 | 8.089 | 1.1215E-10 | up |
| MELO3C001924.2 | 547 | 1899 | 1.795 | 3.24962E-71 | up |
| MELO3C001938.2 | 38011 | 17129 | -1.15 | 2.48819E-49 | down |
| MELO3C001940.2 | 5 | 20 | 2.022 | 0.010872109 | up |
| MELO3C001942.2 | 3988 | 1939 | -1.04 | 4.40546E-35 | down |
| MELO3C001944.2 | 1134 | 170 | -2.735 | 4.84285E-33 | down |
| MELO3C001954.2 | 140 | 343 | 1.296 | 3.77432E-12 | up |
| MELO3C001956.2 | 10 | 83 | 2.989 | 6.11992E-12 | up |
| MELO3C001958.2 | 6 | 1 | -3.484 | 0.029225917 | down |
| MELO3C001960.2 | 354 | 172 | -1.04 | 3.66227E-09 | down |
| MELO3C001964.2 | 49 | 159 | 1.689 | 6.99323E-09 | up |
| MELO3C001970.2 | 17 | 239 | 3.808 | 1.90578E-32 | up |
| MELO3C001971.2 | 55 | 12 | -2.195 | 4.26134E-07 | down |
| MELO3C001980.2 | 2243 | 4757 | 1.085 | 4.81895E-49 | up |
| MELO3C001981.2 | 1106 | 2258 | 1.029 | 6.04263E-10 | up |
| MELO3C001983.2 | 10 | 147 | 3.917 | 2.22319E-23 | up |
| MELO3C001986.2 | 18 | 3 | -2.572 | 0.001385591 | down |
| MELO3C001992.2 | 279 | 1 | -7.896 | 9.34287E-24 | down |
| MELO3C001994.2 | 1241 | 442 | -1.49 | 3.11123E-18 | down |
| MELO3C001995.2 | 418 | 98 | -2.093 | 4.05617E-25 | down |
| MELO3C001996.2 | 786 | 235 | -1.741 | 1.43603E-42 | down |
| MELO3C001997.2 | 73 | 232 | 1.664 | 1.52894E-14 | up |
| MELO3C001998.2 | 8960 | 2727 | -1.716 | 2.3752E-61 | down |
| MELO3C002003.2 | 37 | 88 | 1.254 | 0.000286293 | up |
| MELO3C002004.2 | 18 | 56 | 1.625 | 8.01938E-05 | up |
| MELO3C002009.2 | 0 | 8 | 5.159 | 0.002428958 | up |
| MELO3C002013.2 | 39 | 13 | -1.538 | 0.005963687 | down |
| MELO3C002020.2 | 17320 | 6962 | -1.315 | 2.29E-49 | down |
| MELO3C002024.2 | 87 | 37 | -1.2 | 0.000654923 | down |
| MELO3C002025.2 | 0 | 6 | 4.931 | 0.006486083 | up |
| MELO3C002028.2 | 106 | 13 | -3.089 | 1.53738E-13 | down |
| MELO3C002029.2 | 219 | 61 | -1.854 | 1.05413E-15 | down |
| MELO3C002030.2 | 2499 | 6625 | 1.407 | 7.07905E-58 | up |
| MELO3C002036.2 | 1084 | 350 | -1.634 | 1.39124E-20 | down |
| MELO3C002043.2 | 4 | 17 | 1.879 | 0.018674934 | up |
| MELO3C002044.2 | 69 | 291 | 2.088 | 3.77036E-19 | up |
| MELO3C002049.2 | 15 | 1 | -3.365 | 0.001012697 | down |
| MELO3C002056.2 | 613 | 1949 | 1.668 | 4.74011E-52 | up |
| MELO3C002059.2 | 81 | 4 | -4.2 | 6.79164E-15 | down |
| MELO3C002063.2 | 3830 | 758 | -2.338 | 2.32078E-74 | down |
| MELO3C002066.2 | 35 | 114 | 1.695 | 1.60492E-06 | up |
| MELO3C002070.2 | 241 | 15 | -4.034 | 1.59834E-34 | down |
| MELO3C002072.2 | 44 | 22 | -1.023 | 0.014038083 | down |
| MELO3C002073.2 | 1210 | 84 | -3.837 | 1.63327E-116 | down |
| MELO3C002079.2 | 247 | 813 | 1.721 | 3.29696E-15 | up |
| MELO3C002083.2 | 154 | 49 | -1.643 | 7.10946E-05 | down |
| MELO3C002084.2 | 584 | 183 | -1.673 | 0.018023243 | down |
| MELO3C002090.2 | 71 | 163 | 1.198 | 2.86276E-05 | up |
| MELO3C002100.2 | 7 | 24 | 1.852 | 0.006510626 | up |
| MELO3C002105.2 | 236 | 809 | 1.778 | 1.4476E-22 | up |
| MELO3C002106.2 | 9 | 63 | 2.698 | 5.33668E-07 | up |
| MELO3C002107.2 | 8 | 0 | -5.569 | 0.002112265 | down |
| MELO3C002112.2 | 54 | 372 | 2.797 | 3.50466E-38 | up |
| MELO3C002114.2 | 2 | 46 | 4.36 | 3.83188E-07 | up |
| MELO3C002122.2 | 164 | 736 | 2.168 | 1.1635E-17 | up |
| MELO3C002126.2 | 1403 | 247 | -2.512 | 1.00319E-81 | down |
| MELO3C002128.2 | 8 | 201 | 4.752 | 8.03761E-05 | up |
| MELO3C002131.2 | 96 | 536 | 2.484 | 2.54412E-41 | up |
| MELO3C002138.2 | 212 | 642 | 1.6 | 5.95503E-27 | up |
| MELO3C002140.2 | 375 | 7 | -5.73 | 5.81837E-58 | down |
| MELO3C002143.2 | 82 | 335 | 2.03 | 1.92559E-21 | up |
| MELO3C002144.2 | 437 | 2843 | 2.702 | 7.3613E-113 | up |
| MELO3C002145.2 | 8 | 0 | -5.542 | 0.00125501 | down |
| MELO3C002147.2 | 842 | 354 | -1.251 | 1.90591E-16 | down |
| MELO3C002152.2 | 5297 | 2407 | -1.138 | 9.70563E-28 | down |
| MELO3C002154.2 | 751 | 74 | -3.339 | 5.85225E-78 | down |
| MELO3C002161.2 | 109 | 2 | -5.936 | 3.36356E-18 | down |
| MELO3C002187.2 | 4625 | 1529 | -1.596 | 8.44749E-45 | down |
| MELO3C002202.2 | 1 | 17 | 4.503 | 0.000447928 | up |
| MELO3C002206.2 | 956 | 397 | -1.267 | 1.50653E-22 | down |
| MELO3C002207.2 | 176 | 7 | -4.712 | 3.12379E-30 | down |
| MELO3C002208.2 | 8653 | 502 | -4.109 | 0 | down |
| MELO3C002209.2 | 25270 | 5355 | -2.238 | 1.82533E-180 | down |
| MELO3C002210.2 | 103 | 1 | -6.121 | 5.05113E-15 | down |
| MELO3C002216.2 | 42 | 16 | -1.38 | 0.006476377 | down |
| MELO3C002220.2 | 860 | 188 | -2.196 | 2.97591E-33 | down |
| MELO3C002224.2 | 182 | 53 | -1.771 | 2.56328E-10 | down |
| MELO3C002227.2 | 913 | 356 | -1.355 | 4.9921E-20 | down |
| MELO3C002228.2 | 9055 | 4241 | -1.094 | 4.01394E-62 | down |
| MELO3C002247.2 | 69 | 30 | -1.223 | 0.000811105 | down |
| MELO3C002248.2 | 13 | 209 | 3.999 | 2.0235E-32 | up |
| MELO3C002250.2 | 195 | 80 | -1.276 | 5.42962E-05 | down |
| MELO3C002253.2 | 31 | 9 | -1.79 | 0.001380096 | down |
| MELO3C002262.2 | 319 | 96 | -1.723 | 1.71433E-17 | down |
| MELO3C002269.2 | 5658 | 27448 | 2.278 | 1.41192E-159 | up |
| MELO3C002271.2 | 742 | 289 | -1.362 | 4.24321E-21 | down |
| MELO3C002276.2 | 1524 | 735 | -1.054 | 6.26348E-16 | down |
| MELO3C002277.2 | 1127 | 139 | -3.019 | 2.53015E-64 | down |
| MELO3C002280.2 | 72 | 471 | 2.704 | 1.25886E-30 | up |
| MELO3C002284.2 | 1028 | 174 | -2.557 | 2.86198E-38 | down |
| MELO3C002292.2 | 12 | 146 | 3.65 | 5.39512E-15 | up |
| MELO3C002305.2 | 71 | 12 | -2.615 | 1.86901E-09 | down |
| MELO3C002315.2 | 14 | 0 | -6.424 | 0.000134255 | down |
| MELO3C002316.2 | 1376 | 147 | -3.225 | 3.47241E-98 | down |
| MELO3C002318.2 | 292 | 1272 | 2.126 | 1.1605E-43 | up |
| MELO3C002319.2 | 221 | 11347 | 5.682 | 2.19141E-09 | up |
| MELO3C002337.2 | 6 | 40 | 2.733 | 3.6827E-06 | up |
| MELO3C002340.2 | 42 | 92 | 1.122 | 0.004150181 | up |
| MELO3C002347.2 | 969 | 1950 | 1.009 | 1.32306E-29 | up |
| MELO3C002350.2 | 1627 | 460 | -1.824 | 8.16439E-67 | down |
| MELO3C002360.2 | 6072 | 1118 | -2.442 | 3.69623E-111 | down |
| MELO3C002363.2 | 729 | 1683 | 1.205 | 2.11359E-31 | up |
| MELO3C002365.2 | 224 | 103 | -1.117 | 4.93952E-07 | down |
| MELO3C002369.2 | 279 | 14 | -4.391 | 1.15283E-50 | down |
| MELO3C002373.2 | 34 | 4 | -3.016 | 0.002947689 | down |
| MELO3C002374.2 | 27 | 0 | -7.397 | 3.05865E-08 | down |
| MELO3C002381.2 | 104 | 3 | -5.152 | 1.14702E-19 | down |
| MELO3C002382.2 | 436 | 1516 | 1.796 | 3.15337E-26 | up |
| MELO3C002383.2 | 1 | 36 | 5.004 | 6.00833E-06 | up |
| MELO3C002391.2 | 139 | 0 | -9.736 | 1.25868E-15 | down |
| MELO3C002392.2 | 312 | 1 | -9.049 | 3.14448E-18 | down |
| MELO3C002393.2 | 85 | 507 | 2.579 | 2.57764E-24 | up |
| MELO3C002394.2 | 43 | 355 | 3.031 | 1.04895E-23 | up |
| MELO3C002396.2 | 1583 | 486 | -1.705 | 7.25584E-56 | down |
| MELO3C002400.2 | 843 | 326 | -1.373 | 5.70378E-15 | down |
| MELO3C002414.2 | 63 | 164 | 1.393 | 8.5403E-07 | up |
| MELO3C002416.2 | 649 | 2582 | 1.994 | 1.71017E-82 | up |
| MELO3C002426.2 | 80 | 27 | -1.601 | 0.000121829 | down |
| MELO3C002435.2 | 14 | 203 | 3.913 | 4.1414E-31 | up |
| MELO3C002436.2 | 0 | 78 | 8.566 | 4.19788E-12 | up |
| MELO3C002437.2 | 84 | 4436 | 5.728 | 0 | up |
| MELO3C002441.2 | 3 | 196 | 6.004 | 1.36488E-09 | up |
| MELO3C002454.2 | 9 | 38 | 2.099 | 0.000260399 | up |
| MELO3C002456.2 | 10611 | 1788 | -2.569 | 8.96133E-137 | down |
| MELO3C002457.2 | 2527 | 10307 | 2.028 | 1.00252E-41 | up |
| MELO3C002459.2 | 1221 | 374 | -1.709 | 1.29024E-17 | down |
| MELO3C002463.2 | 10740 | 5135 | -1.065 | 3.40865E-36 | down |
| MELO3C002468.2 | 7686 | 3581 | -1.102 | 8.67065E-47 | down |
| MELO3C002479.2 | 213 | 59 | -1.838 | 1.16967E-08 | down |
| MELO3C002480.2 | 5 | 21 | 1.899 | 0.013079557 | up |
| MELO3C002483.2 | 82 | 12 | -2.857 | 3.33645E-11 | down |
| MELO3C002485.2 | 768 | 71 | -3.428 | 1.36124E-100 | down |
| MELO3C002489.2 | 1042 | 450 | -1.212 | 1.97046E-15 | down |
| MELO3C002493.2 | 49 | 7 | -2.859 | 2.32836E-06 | down |
| MELO3C002500.2 | 13 | 1 | -4.482 | 0.001690161 | down |
| MELO3C002501.2 | 41 | 1 | -5.524 | 4.00962E-07 | down |
| MELO3C002504.2 | 17440 | 6851 | -1.348 | 6.03871E-25 | down |
| MELO3C002508.2 | 9595 | 1867 | -2.361 | 2.35398E-86 | down |
| MELO3C002510.2 | 31390 | 2814 | -3.48 | 0 | down |
| MELO3C002511.2 | 413 | 9 | -5.586 | 2.31488E-52 | down |
| MELO3C002520.2 | 6 | 0 | -5.203 | 0.005248847 | down |
| MELO3C002521.2 | 850 | 219 | -1.957 | 2.48249E-30 | down |
| MELO3C002529.2 | 0 | 10 | 5.63 | 0.000422173 | up |
| MELO3C002542.2 | 486 | 237 | -1.034 | 4.93393E-11 | down |
| MELO3C002543.2 | 20 | 4 | -2.507 | 0.008693586 | down |
| MELO3C002546.2 | 16 | 181 | 3.556 | 1.60396E-26 | up |
| MELO3C002551.2 | 57 | 3 | -4.302 | 8.51275E-10 | down |
| MELO3C002553.2 | 118 | 421 | 1.828 | 4.82543E-21 | up |
| MELO3C002555.2 | 18 | 261 | 3.862 | 1.07325E-40 | up |
| MELO3C002560.2 | 151 | 22 | -2.796 | 4.19585E-15 | down |
| MELO3C002563.2 | 2012 | 859 | -1.228 | 2.35032E-44 | down |
| MELO3C002564.2 | 2352 | 850 | -1.468 | 1.4677E-46 | down |
| MELO3C002567.2 | 21 | 79 | 1.935 | 2.87072E-07 | up |
| MELO3C002572.2 | 354 | 4 | -6.305 | 5.22908E-07 | down |
| MELO3C002576.2 | 94 | 0 | -9.165 | 0.019007477 | down |
| MELO3C002579.2 | 23 | 164 | 2.834 | 0.005268955 | up |
| MELO3C002582.2 | 2 | 14 | 2.616 | 0.009358696 | up |
| MELO3C002590.2 | 24 | 8 | -1.65 | 0.006965449 | down |
| MELO3C002594.2 | 782 | 277 | -1.498 | 3.80101E-22 | down |
| MELO3C002599.2 | 8 | 31 | 2.001 | 0.001700748 | up |
| MELO3C002607.2 | 52 | 7 | -3.059 | 3.52937E-08 | down |
| MELO3C002609.2 | 78 | 235 | 1.584 | 2.25853E-15 | up |
| MELO3C002614.2 | 115 | 279 | 1.278 | 3.87991E-11 | up |
| MELO3C002615.2 | 22 | 2 | -3.729 | 0.000429424 | down |
| MELO3C002616.2 | 139 | 42 | -1.709 | 1.58083E-08 | down |
| MELO3C002624.2 | 84 | 29 | -1.507 | 0.00418442 | down |
| MELO3C002628.2 | 1190 | 324 | -1.877 | 5.44809E-50 | down |
| MELO3C002631.2 | 333 | 724 | 1.121 | 1.00835E-15 | up |
| MELO3C002637.2 | 81 | 4 | -4.209 | 2.80837E-16 | down |
| MELO3C002647.2 | 17 | 1 | -3.905 | 0.000262349 | down |
| MELO3C002651.2 | 1755 | 821 | -1.094 | 5.3923E-18 | down |
| MELO3C002655.2 | 5 | 17 | 1.815 | 0.015951897 | up |
| MELO3C002661.2 | 198 | 2 | -6.806 | 1.05545E-25 | down |
| MELO3C002664.2 | 455 | 1879 | 2.046 | 2.23774E-85 | up |
| MELO3C002665.2 | 114 | 250 | 1.129 | 7.9099E-07 | up |
| MELO3C002669.2 | 25 | 4 | -2.801 | 7.35385E-05 | down |
| MELO3C002674.2 | 307 | 19 | -4.002 | 1.07915E-49 | down |
| MELO3C002676.2 | 299 | 105 | -1.501 | 2.46959E-09 | down |
| MELO3C002677.2 | 4972 | 1831 | -1.441 | 4.28166E-39 | down |
| MELO3C002678.2 | 1696 | 638 | -1.41 | 6.39281E-41 | down |
| MELO3C002679.2 | 1499 | 471 | -1.669 | 2.66182E-52 | down |
| MELO3C002681.2 | 326 | 132 | -1.308 | 2.43029E-11 | down |
| MELO3C002685.2 | 199 | 720 | 1.853 | 1.47123E-44 | up |
| MELO3C002689.2 | 578 | 156 | -1.885 | 2.23055E-20 | down |
| MELO3C002691.2 | 7420 | 176 | -5.386 | 1.23123E-246 | down |
| MELO3C002695.2 | 149 | 24 | -2.626 | 8.98379E-17 | down |
| MELO3C002697.2 | 3 | 38 | 3.354 | 3.98466E-07 | up |
| MELO3C002700.2 | 183 | 1765 | 3.265 | 1.54291E-141 | up |
| MELO3C002702.2 | 14 | 3 | -2.412 | 0.00834199 | down |
| MELO3C002708.2 | 1652 | 684 | -1.272 | 3.04907E-38 | down |
| MELO3C002709.2 | 6 | 33 | 2.356 | 7.72897E-05 | up |
| MELO3C002716.2 | 2108 | 4882 | 1.211 | 3.19795E-37 | up |
| MELO3C002718.2 | 13 | 2 | -2.914 | 0.0022659 | down |
| MELO3C002719.2 | 1081 | 2233 | 1.046 | 4.59687E-19 | up |
| MELO3C002723.2 | 1 | 20 | 4.741 | 0.000197683 | up |
| MELO3C002726.2 | 1918 | 126 | -3.924 | 2.81113E-195 | down |
| MELO3C002727.2 | 45733 | 19 | -11.28 | 3.6273E-111 | down |
| MELO3C002730.2 | 62 | 245 | 1.982 | 2.59828E-21 | up |
| MELO3C002731.2 | 296 | 129 | -1.201 | 7.67187E-09 | down |
| MELO3C002732.2 | 498 | 1097 | 1.14 | 5.75278E-18 | up |
| MELO3C002736.2 | 1047 | 475 | -1.14 | 1.50038E-21 | down |
| MELO3C002747.2 | 63 | 167 | 1.413 | 7.62787E-06 | up |
| MELO3C002749.2 | 8 | 1 | -3.692 | 0.020460887 | down |
| MELO3C002750.2 | 931 | 144 | -2.693 | 3.18494E-57 | down |
| MELO3C002755.2 | 800 | 75 | -3.401 | 1.22355E-54 | down |
| MELO3C002756.2 | 262 | 59 | -2.141 | 1.18385E-23 | down |
| MELO3C002763.2 | 718 | 31 | -4.547 | 2.37526E-118 | down |
| MELO3C002768.2 | 3870 | 12 | -8.355 | 7.88181E-231 | down |
| MELO3C002770.2 | 231 | 471 | 1.029 | 2.60811E-14 | up |
| MELO3C002777.2 | 58 | 0 | -8.488 | 1.82273E-11 | down |
| MELO3C002778.2 | 4 | 277 | 6.222 | 6.12869E-28 | up |
| MELO3C002779.2 | 0 | 46 | 7.817 | 1.06651E-09 | up |
| MELO3C002781.2 | 93 | 321 | 1.788 | 3.03915E-21 | up |
| MELO3C002807.2 | 14 | 51 | 1.841 | 2.44083E-05 | up |
| MELO3C002809.2 | 15 | 91 | 2.537 | 1.06661E-08 | up |
| MELO3C002811.2 | 127 | 290 | 1.192 | 1.32782E-08 | up |
| MELO3C002812.2 | 61 | 183 | 1.574 | 7.59359E-11 | up |
| MELO3C002821.2 | 439 | 902 | 1.036 | 1.3667E-11 | up |
| MELO3C002829.2 | 65 | 146 | 1.163 | 6.22292E-06 | up |
| MELO3C002832.2 | 207 | 770 | 1.894 | 7.91073E-52 | up |
| MELO3C002839.2 | 1975 | 43 | -5.522 | 2.61021E-203 | down |
| MELO3C002853.2 | 108 | 40739 | 8.556 | 6.15743E-31 | up |
| MELO3C002855.2 | 28 | 84 | 1.566 | 1.02026E-05 | up |
| MELO3C002874.2 | 27250 | 2203 | -3.628 | 4.14699E-121 | down |
| MELO3C002875.2 | 1 | 138 | 6.952 | 4.5638E-14 | up |
| MELO3C002877.2 | 257 | 807 | 1.65 | 5.65882E-42 | up |
| MELO3C002892.2 | 5 | 17 | 1.762 | 0.025440153 | up |
| MELO3C002912.2 | 1677 | 619 | -1.439 | 6.25047E-26 | down |
| MELO3C002921.2 | 572 | 81 | -2.819 | 7.78238E-30 | down |
| MELO3C002925.2 | 52 | 0 | -8.304 | 8.74578E-11 | down |
| MELO3C002934.2 | 2163 | 667 | -1.698 | 2.22206E-37 | down |
| MELO3C002941.2 | 38 | 1334 | 5.136 | 1.25761E-109 | up |
| MELO3C002943.2 | 25 | 4 | -2.75 | 0.002454778 | down |
| MELO3C002961.2 | 5 | 0 | -5.053 | 0.010749957 | down |
| MELO3C002962.2 | 356 | 12 | -4.886 | 9.46112E-52 | down |
| MELO3C002975.2 | 65 | 30 | -1.108 | 0.002030074 | down |
| MELO3C002976.2 | 21 | 71 | 1.735 | 3.89291E-05 | up |
| MELO3C002978.2 | 293 | 37 | -2.973 | 1.30709E-16 | down |
| MELO3C002980.2 | 750 | 1626 | 1.116 | 5.61256E-28 | up |
| MELO3C002988.2 | 1686 | 724 | -1.22 | 1.89435E-28 | down |
| MELO3C003002.2 | 115 | 2 | -5.615 | 2.98602E-18 | down |
| MELO3C003032.2 | 299 | 11 | -4.748 | 1.14182E-54 | down |
| MELO3C003044.2 | 238 | 80 | -1.565 | 6.99066E-11 | down |
| MELO3C003061.2 | 3493 | 973 | -1.845 | 1.6526E-88 | down |
| MELO3C003066.2 | 11 | 79 | 2.821 | 3.43567E-08 | up |
| MELO3C003075.2 | 18825 | 9294 | -1.018 | 4.41001E-49 | down |
| MELO3C003080.2 | 393 | 961 | 1.289 | 1.68979E-30 | up |
| MELO3C003082.2 | 5 | 0 | -5.061 | 0.021412758 | down |
| MELO3C003088.2 | 1515 | 566 | -1.42 | 3.9554E-40 | down |
| MELO3C003090.2 | 86 | 10 | -3.203 | 9.87863E-13 | down |
| MELO3C003096.2 | 1067 | 351 | -1.603 | 7.08698E-35 | down |
| MELO3C003097.2 | 4048 | 1545 | -1.39 | 7.1387E-73 | down |
| MELO3C003098.2 | 10426 | 4578 | -1.187 | 8.73397E-21 | down |
| MELO3C003107.2 | 765 | 1878 | 1.296 | 6.23023E-18 | up |
| MELO3C003111.2 | 1151 | 484 | -1.251 | 1.44277E-21 | down |
| MELO3C003112.2 | 245 | 49 | -2.318 | 2.72402E-17 | down |
| MELO3C003126.2 | 44 | 21 | -1.052 | 0.024315015 | down |
| MELO3C003131.2 | 173 | 27 | -2.644 | 9.96007E-20 | down |
| MELO3C003132.2 | 12937 | 6179 | -1.066 | 1.96021E-12 | down |
| MELO3C003133.2 | 1335 | 662 | -1.012 | 1.10384E-24 | down |
| MELO3C003134.2 | 280 | 1015 | 1.856 | 1.50996E-19 | up |
| MELO3C003137.2 | 3155 | 1222 | -1.369 | 1.14309E-40 | down |
| MELO3C003138.2 | 8 | 1 | -3.905 | 0.018130006 | down |
| MELO3C003143.2 | 3408 | 1494 | -1.19 | 5.65563E-56 | down |
| MELO3C003146.2 | 112 | 1 | -6.576 | 1.93343E-16 | down |
| MELO3C003147.2 | 3352 | 669 | -2.324 | 2.17201E-86 | down |
| MELO3C003148.2 | 84 | 1 | -6.126 | 5.25985E-14 | down |
| MELO3C003155.2 | 46 | 13 | -1.833 | 0.0013576 | down |
| MELO3C003157.2 | 386 | 159 | -1.281 | 3.49451E-08 | down |
| MELO3C003167.2 | 0 | 5 | 4.495 | 0.023271607 | up |
| MELO3C003175.2 | 12 | 2 | -2.585 | 0.00571372 | down |
| MELO3C003177.2 | 16 | 1 | -4.833 | 0.000200618 | down |
| MELO3C003181.2 | 1678 | 728 | -1.205 | 2.05507E-30 | down |
| MELO3C003183.2 | 31 | 0 | -7.558 | 1.38705E-08 | down |
| MELO3C003187.2 | 91 | 478 | 2.387 | 7.77139E-37 | up |
| MELO3C003195.2 | 47 | 112 | 1.27 | 1.03145E-05 | up |
| MELO3C003197.2 | 508 | 166 | -1.611 | 8.63343E-16 | down |
| MELO3C003199.2 | 866 | 1787 | 1.044 | 4.16561E-10 | up |
| MELO3C003204.2 | 44 | 2 | -4.271 | 3.91878E-08 | down |
| MELO3C003205.2 | 4661 | 855 | -2.448 | 6.13255E-178 | down |
| MELO3C003206.2 | 149 | 329 | 1.145 | 3.02342E-08 | up |
| MELO3C003214.2 | 6604 | 1490 | -2.149 | 2.79907E-70 | down |
| MELO3C003215.2 | 2865 | 1392 | -1.041 | 1.21243E-24 | down |
| MELO3C003219.2 | 977 | 1972 | 1.013 | 6.77404E-21 | up |
| MELO3C003224.2 | 37 | 124 | 1.731 | 1.46732E-07 | up |
| MELO3C003227.2 | 914 | 272 | -1.753 | 9.07616E-38 | down |
| MELO3C003238.2 | 43807 | 8604 | -2.348 | 1.25784E-168 | down |
| MELO3C003239.2 | 74 | 184 | 1.313 | 1.99399E-09 | up |
| MELO3C003241.2 | 39 | 84 | 1.131 | 0.000359438 | up |
| MELO3C003245.2 | 3222 | 955 | -1.755 | 1.63603E-63 | down |
| MELO3C003254.2 | 81 | 0 | -8.95 | 7.45749E-13 | down |
| MELO3C003255.2 | 283 | 0 | -10.76 | 2.38884E-19 | down |
| MELO3C003271.2 | 4 | 19 | 2.293 | 0.004410243 | up |
| MELO3C003275.2 | 21 | 3 | -2.997 | 0.000133409 | down |
| MELO3C003284.2 | 187 | 60 | -1.62 | 2.21551E-08 | down |
| MELO3C003295.2 | 417 | 123 | -1.77 | 1.0007E-17 | down |
| MELO3C003299.2 | 25 | 444 | 4.134 | 6.81285E-54 | up |
| MELO3C003305.2 | 146 | 689 | 2.243 | 3.33985E-28 | up |
| MELO3C003308.2 | 2905 | 728 | -1.995 | 5.32842E-75 | down |
| MELO3C003318.2 | 12 | 0 | -6.249 | 4.18177E-05 | down |
| MELO3C003321.2 | 110 | 53 | -1.055 | 0.000113935 | down |
| MELO3C003324.2 | 11715 | 4675 | -1.326 | 5.43469E-24 | down |
| MELO3C003325.2 | 13 | 0 | -6.289 | 2.81086E-05 | down |
| MELO3C003328.2 | 480 | 138 | -1.799 | 4.60078E-17 | down |
| MELO3C003331.2 | 8093 | 855 | -3.245 | 6.22024E-105 | down |
| MELO3C003337.2 | 11 | 0 | -6.094 | 7.80675E-05 | down |
| MELO3C003339.2 | 567 | 1139 | 1.006 | 1.01659E-15 | up |
| MELO3C003344.2 | 8857 | 4005 | -1.145 | 1.04773E-44 | down |
| MELO3C003361.2 | 32 | 2 | -3.839 | 2.92607E-05 | down |
| MELO3C003366.2 | 0 | 26 | 6.016 | 6.29562E-06 | up |
| MELO3C003371.2 | 358 | 47 | -2.928 | 0.000224056 | down |
| MELO3C003372.2 | 1455 | 297 | -2.291 | 5.44621E-74 | down |
| MELO3C003373.2 | 1339 | 581 | -1.206 | 5.48518E-23 | down |
| MELO3C003374.2 | 1144 | 301 | -1.927 | 2.46113E-63 | down |
| MELO3C003375.2 | 119984 | 18105 | -2.728 | 3.7398E-162 | down |
| MELO3C003379.2 | 411 | 164 | -1.324 | 5.31711E-15 | down |
| MELO3C003380.2 | 9 | 38 | 2.001 | 0.000700085 | up |
| MELO3C003384.2 | 377 | 0 | -11.172 | 1.45423E-20 | down |
| MELO3C003385.2 | 14 | 0 | -6.399 | 1.43467E-05 | down |
| MELO3C003386.2 | 2421 | 474 | -2.353 | 1.38268E-39 | down |
| MELO3C003388.2 | 43 | 117 | 1.434 | 3.83209E-06 | up |
| MELO3C003393.2 | 2383 | 185 | -3.691 | 7.97755E-258 | down |
| MELO3C003394.2 | 2006 | 180 | -3.479 | 8.70791E-23 | down |
| MELO3C003395.2 | 2488 | 534 | -2.22 | 2.87156E-133 | down |
| MELO3C003403.2 | 67 | 228 | 1.775 | 2.69945E-13 | up |
| MELO3C003413.2 | 134 | 375 | 1.483 | 2.51994E-12 | up |
| MELO3C003422.2 | 4 | 0 | -4.524 | 0.030150149 | down |
| MELO3C003425.2 | 610 | 293 | -1.059 | 2.97963E-11 | down |
| MELO3C003426.2 | 508 | 126 | -2.01 | 2.49565E-38 | down |
| MELO3C003431.2 | 143 | 379 | 1.406 | 3.10209E-17 | up |
| MELO3C003433.2 | 330 | 116 | -1.509 | 2.02668E-13 | down |
| MELO3C003439.2 | 3819 | 1823 | -1.068 | 1.77422E-39 | down |
| MELO3C003442.2 | 893 | 376 | -1.249 | 2.48017E-27 | down |
| MELO3C003447.2 | 771 | 374 | -1.042 | 4.92833E-16 | down |
| MELO3C003450.2 | 1352 | 3275 | 1.276 | 3.98603E-13 | up |
| MELO3C003451.2 | 8 | 54 | 2.788 | 2.71143E-08 | up |
| MELO3C003452.2 | 102 | 374 | 1.871 | 4.53102E-22 | up |
| MELO3C003466.2 | 0 | 7 | 5.031 | 0.00455122 | up |
| MELO3C003468.2 | 97 | 360 | 1.893 | 1.3135E-24 | up |
| MELO3C003469.2 | 372 | 28 | -3.756 | 2.93632E-07 | down |
| MELO3C003473.2 | 55 | 16 | -1.813 | 5.12671E-05 | down |
| MELO3C003479.2 | 1189 | 480 | -1.31 | 3.76607E-33 | down |
| MELO3C003480.2 | 251 | 877 | 1.804 | 2.68365E-22 | up |
| MELO3C003483.2 | 895 | 232 | -1.944 | 7.93949E-52 | down |
| MELO3C003485.2 | 51 | 20 | -1.327 | 0.002359321 | down |
| MELO3C003491.2 | 1794 | 657 | -1.448 | 2.35869E-17 | down |
| MELO3C003494.2 | 3 | 83 | 4.799 | 1.39033E-13 | up |
| MELO3C003497.2 | 96 | 240 | 1.322 | 9.18015E-11 | up |
| MELO3C003506.2 | 492 | 122 | -2.017 | 1.13152E-33 | down |
| MELO3C003507.2 | 205 | 69 | -1.564 | 1.70696E-11 | down |
| MELO3C003508.2 | 602 | 255 | -1.238 | 5.76913E-17 | down |
| MELO3C003519.2 | 18 | 314 | 4.123 | 4.27983E-44 | up |
| MELO3C003520.2 | 5665 | 867 | -2.709 | 7.06531E-54 | down |
| MELO3C003522.2 | 38 | 86 | 1.19 | 0.000768563 | up |
| MELO3C003526.2 | 75 | 2 | -5.192 | 3.65294E-15 | down |
| MELO3C003532.2 | 459 | 1425 | 1.636 | 2.84306E-50 | up |
| MELO3C003540.2 | 840 | 4077 | 2.279 | 1.41629E-89 | up |
| MELO3C003544.2 | 16 | 1 | -4.724 | 0.000437893 | down |
| MELO3C003546.2 | 2755 | 743 | -1.892 | 1.24236E-84 | down |
| MELO3C003554.2 | 282 | 2574 | 3.193 | 2.20642E-107 | up |
| MELO3C003558.2 | 4 | 22 | 2.424 | 0.001938761 | up |
| MELO3C003559.2 | 1156 | 128 | -3.173 | 6.42685E-54 | down |
| MELO3C003561.2 | 1440 | 627 | -1.2 | 3.80913E-36 | down |
| MELO3C003565.2 | 5 | 52 | 3.509 | 2.45296E-06 | up |
| MELO3C003569.2 | 6143 | 2845 | -1.11 | 7.52138E-38 | down |
| MELO3C003576.2 | 24 | 1 | -4.083 | 4.40353E-05 | down |
| MELO3C003585.2 | 250 | 28 | -3.14 | 6.0428E-28 | down |
| MELO3C003598.2 | 43 | 6 | -2.708 | 1.70083E-05 | down |
| MELO3C003604.2 | 14 | 46 | 1.738 | 0.000157775 | up |
| MELO3C003616.2 | 58 | 419 | 2.855 | 1.08461E-30 | up |
| MELO3C003617.2 | 6 | 111 | 4.225 | 1.55334E-18 | up |
| MELO3C003620.2 | 322 | 40 | -3.026 | 3.91854E-44 | down |
| MELO3C003622.2 | 2806 | 828 | -1.761 | 1.97732E-94 | down |
| MELO3C003623.2 | 1341 | 346 | -1.955 | 1.09375E-43 | down |
| MELO3C003624.2 | 1147 | 507 | -1.176 | 1.3861E-26 | down |
| MELO3C003628.2 | 84 | 350 | 2.061 | 4.21425E-24 | up |
| MELO3C003629.2 | 5 | 197 | 5.135 | 2.93017E-30 | up |
| MELO3C003633.2 | 98 | 0 | -9.235 | 8.88255E-14 | down |
| MELO3C003634.2 | 43 | 1 | -4.879 | 1.92571E-09 | down |
| MELO3C003638.2 | 11 | 163 | 3.901 | 1.51943E-19 | up |
| MELO3C003640.2 | 8 | 1 | -3.809 | 0.010950682 | down |
| MELO3C003642.2 | 288 | 104 | -1.471 | 3.23922E-12 | down |
| MELO3C003646.2 | 426 | 896 | 1.072 | 6.42533E-15 | up |
| MELO3C003649.2 | 1408 | 632 | -1.154 | 3.70612E-24 | down |
| MELO3C003652.2 | 2 | 57 | 4.664 | 7.03241E-10 | up |
| MELO3C003656.2 | 239 | 46 | -2.382 | 1.29637E-27 | down |
| MELO3C003657.2 | 152 | 54 | -1.492 | 4.69057E-09 | down |
| MELO3C003658.2 | 584 | 1183 | 1.017 | 1.30604E-19 | up |
| MELO3C003662.2 | 273 | 89 | -1.619 | 2.00797E-14 | down |
| MELO3C003669.2 | 1589 | 3623 | 1.189 | 4.67537E-38 | up |
| MELO3C003672.2 | 10 | 41 | 1.981 | 0.000187444 | up |
| MELO3C003674.2 | 180 | 376 | 1.063 | 5.75806E-10 | up |
| MELO3C003676.2 | 233 | 14 | -3.977 | 1.27207E-31 | down |
| MELO3C003678.2 | 1743 | 839 | -1.056 | 1.88867E-24 | down |
| MELO3C003680.2 | 144 | 8 | -4.147 | 1.07726E-21 | down |
| MELO3C003686.2 | 575 | 138 | -2.052 | 3.12206E-13 | down |
| MELO3C003689.2 | 3190 | 11932 | 1.904 | 2.68347E-60 | up |
| MELO3C003692.2 | 250 | 627 | 1.325 | 7.64101E-23 | up |
| MELO3C003697.2 | 4433 | 15316 | 1.789 | 1.39534E-100 | up |
| MELO3C003698.2 | 45 | 669 | 3.898 | 2.3132E-66 | up |
| MELO3C003716.2 | 4 | 44 | 3.28 | 4.58258E-06 | up |
| MELO3C003720.2 | 77 | 0 | -7.919 | 2.79615E-10 | down |
| MELO3C003721.2 | 2384 | 633 | -1.914 | 1.16049E-67 | down |
| MELO3C003725.2 | 18884 | 254 | -6.217 | 0 | down |
| MELO3C003726.2 | 119 | 55 | -1.096 | 0.005283106 | down |
| MELO3C003727.2 | 8 | 1 | -3.732 | 0.015384322 | down |
| MELO3C003729.2 | 455 | 1074 | 1.237 | 1.38789E-22 | up |
| MELO3C003731.2 | 1625 | 5240 | 1.689 | 2.94304E-58 | up |
| MELO3C003735.2 | 14 | 34 | 1.322 | 0.013514753 | up |
| MELO3C003737.2 | 148 | 24 | -2.634 | 2.66748E-06 | down |
| MELO3C003742.2 | 65 | 14 | -2.24 | 0.000138017 | down |
| MELO3C003743.2 | 588 | 159 | -1.88 | 4.70371E-25 | down |
| MELO3C003745.2 | 66 | 144 | 1.141 | 0.02747411 | up |
| MELO3C003757.2 | 3 | 32 | 3.393 | 0.000168565 | up |
| MELO3C003759.2 | 885 | 336 | -1.398 | 6.14152E-21 | down |
| MELO3C003760.2 | 84 | 539 | 2.679 | 1.61628E-56 | up |
| MELO3C003761.2 | 126 | 5 | -4.657 | 8.52295E-23 | down |
| MELO3C003762.2 | 616 | 250 | -1.299 | 1.3089E-19 | down |
| MELO3C003764.2 | 536 | 2100 | 1.971 | 2.04037E-85 | up |
| MELO3C003783.2 | 0 | 10 | 5.62 | 0.00097411 | up |
| MELO3C003788.2 | 4399 | 1270 | -1.792 | 1.82403E-62 | down |
| MELO3C003790.2 | 0 | 11 | 5.707 | 0.000450774 | up |
| MELO3C003793.2 | 4390 | 1477 | -1.572 | 2.17239E-84 | down |
| MELO3C003797.2 | 94 | 26 | -1.832 | 0.000979796 | down |
| MELO3C003803.2 | 150 | 37 | -2.022 | 4.13422E-14 | down |
| MELO3C003811.2 | 2831 | 860 | -1.72 | 1.64006E-73 | down |
| MELO3C003813.2 | 953 | 348 | -1.452 | 4.71795E-21 | down |
| MELO3C003814.2 | 1435 | 708 | -1.018 | 4.20101E-20 | down |
| MELO3C003820.2 | 180 | 955 | 2.408 | 1.29518E-79 | up |
| MELO3C003821.2 | 35 | 299 | 3.104 | 5.16719E-34 | up |
| MELO3C003823.2 | 1793 | 90 | -4.325 | 6.29316E-28 | down |
| MELO3C003827.2 | 110 | 4 | -4.852 | 1.86791E-21 | down |
| MELO3C003838.2 | 33 | 12 | -1.526 | 0.005706482 | down |
| MELO3C003847.2 | 293 | 128 | -1.191 | 1.6043E-07 | down |
| MELO3C003852.2 | 37 | 267 | 2.867 | 1.35232E-15 | up |
| MELO3C003861.2 | 928 | 2268 | 1.291 | 3.46499E-38 | up |
| MELO3C003874.2 | 1181 | 235 | -2.326 | 1.43633E-83 | down |
| MELO3C003878.2 | 1404 | 311 | -2.172 | 1.15901E-27 | down |
| MELO3C003881.2 | 93 | 11 | -3.062 | 2.58304E-13 | down |
| MELO3C003886.2 | 14 | 145 | 3.406 | 8.32652E-14 | up |
| MELO3C003889.2 | 1 | 31 | 4.381 | 2.39913E-06 | up |
| MELO3C003890.2 | 3647 | 820 | -2.153 | 2.72801E-100 | down |
| MELO3C003902.2 | 3 | 42 | 3.66 | 3.19515E-06 | up |
| MELO3C003911.2 | 59 | 440 | 2.89 | 5.85876E-34 | up |
| MELO3C003916.2 | 46 | 222 | 2.263 | 2.66762E-21 | up |
| MELO3C003917.2 | 137 | 50 | -1.445 | 1.79488E-06 | down |
| MELO3C003918.2 | 4 | 53 | 3.546 | 3.43809E-09 | up |
| MELO3C003919.2 | 20 | 4 | -2.244 | 0.001749233 | down |
| MELO3C003929.2 | 603 | 1318 | 1.129 | 1.29218E-21 | up |
| MELO3C003930.2 | 0 | 14 | 6.069 | 4.7387E-05 | up |
| MELO3C003933.2 | 2 | 33 | 4.147 | 1.83319E-06 | up |
| MELO3C003958.2 | 8 | 1 | -3.797 | 0.014067464 | down |
| MELO3C003972.2 | 666 | 1529 | 1.201 | 2.13707E-32 | up |
| MELO3C003975.2 | 3508 | 651 | -2.431 | 5.0412E-137 | down |
| MELO3C003980.2 | 431 | 3757 | 3.126 | 3.66034E-131 | up |
| MELO3C003990.2 | 8414 | 631 | -3.738 | 1.00435E-152 | down |
| MELO3C004003.2 | 3134 | 322 | -3.287 | 4.31635E-89 | down |
| MELO3C004009.2 | 1034 | 496 | -1.058 | 1.49648E-15 | down |
| MELO3C004020.2 | 5591 | 11231 | 1.006 | 3.81688E-37 | up |
| MELO3C004033.2 | 1104 | 491 | -1.17 | 5.34418E-25 | down |
| MELO3C004039.2 | 248 | 37 | -2.73 | 2.97511E-27 | down |
| MELO3C004040.2 | 4519 | 1251 | -1.853 | 3.85126E-75 | down |
| MELO3C004050.2 | 5160 | 2552 | -1.015 | 5.6628E-12 | down |
| MELO3C004053.2 | 13 | 2 | -2.89 | 0.002863796 | down |
| MELO3C004054.2 | 65 | 10 | -2.755 | 0.000203629 | down |
| MELO3C004059.2 | 16 | 85 | 2.358 | 7.9159E-05 | up |
| MELO3C004065.2 | 883 | 2384 | 1.432 | 0.000182123 | up |
| MELO3C004078.2 | 17 | 156 | 3.196 | 1.94728E-10 | up |
| MELO3C004090.2 | 1025 | 288 | -1.833 | 4.38556E-26 | down |
| MELO3C004092.2 | 11 | 1 | -3.113 | 0.023701139 | down |
| MELO3C004096.2 | 3883 | 591 | -2.714 | 3.50862E-88 | down |
| MELO3C004110.2 | 30246 | 13025 | -1.215 | 6.2138E-21 | down |
| MELO3C004116.2 | 3555 | 1439 | -1.306 | 2.3535E-37 | down |
| MELO3C004125.2 | 2612 | 862 | -1.6 | 8.21367E-66 | down |
| MELO3C004135.2 | 2 | 81 | 5.106 | 0.00029907 | up |
| MELO3C004141.2 | 231 | 23 | -3.348 | 3.06152E-28 | down |
| MELO3C004142.2 | 164 | 19 | -3.145 | 6.38507E-26 | down |
| MELO3C004143.2 | 467 | 1013 | 1.117 | 6.70734E-20 | up |
| MELO3C004145.2 | 12 | 165 | 3.835 | 1.21611E-23 | up |
| MELO3C004155.2 | 16 | 911 | 5.859 | 2.96018E-79 | up |
| MELO3C004161.2 | 7 | 66 | 3.291 | 5.44938E-08 | up |
| MELO3C004172.2 | 719 | 1484 | 1.045 | 2.58711E-27 | up |
| MELO3C004175.2 | 30 | 102 | 1.755 | 4.05296E-08 | up |
| MELO3C004176.2 | 90 | 218 | 1.273 | 5.8991E-10 | up |
| MELO3C004178.2 | 989 | 2187 | 1.145 | 2.31882E-30 | up |
| MELO3C004183.2 | 116 | 9 | -3.653 | 1.56893E-15 | down |
| MELO3C004189.2 | 332 | 871 | 1.392 | 2.69586E-16 | up |
| MELO3C004193.2 | 4 | 0 | -4.552 | 0.031645611 | down |
| MELO3C004195.2 | 69 | 196 | 1.507 | 3.94277E-09 | up |
| MELO3C004199.2 | 20 | 74 | 1.922 | 4.51405E-05 | up |
| MELO3C004207.2 | 2 | 45 | 4.351 | 9.98503E-08 | up |
| MELO3C004212.2 | 247 | 94 | -1.391 | 2.80253E-08 | down |
| MELO3C004214.2 | 52508 | 53 | -9.943 | 0 | down |
| MELO3C004216.2 | 443 | 1616 | 1.867 | 1.08551E-73 | up |
| MELO3C004223.2 | 7762 | 3389 | -1.196 | 3.72236E-52 | down |
| MELO3C004227.2 | 221 | 1026 | 2.218 | 3.61217E-23 | up |
| MELO3C004234.2 | 24 | 60 | 1.356 | 0.000921798 | up |
| MELO3C004235.2 | 1 | 10 | 3.718 | 0.01055548 | up |
| MELO3C004242.2 | 352 | 40 | -3.136 | 0.002461904 | down |
| MELO3C004244.2 | 2187 | 17 | -7.065 | 1.15381E-68 | down |
| MELO3C004247.2 | 67 | 0 | -8.676 | 5.28675E-12 | down |
| MELO3C004249.2 | 36 | 0 | -7.797 | 0.001079651 | down |
| MELO3C004252.2 | 12 | 1 | -4.269 | 0.002846286 | down |
| MELO3C004257.2 | 626 | 286 | -1.132 | 4.88026E-10 | down |
| MELO3C004261.2 | 1 | 243 | 8.347 | 2.03973E-15 | up |
| MELO3C004262.2 | 83 | 24 | -1.787 | 1.48273E-06 | down |
| MELO3C004265.2 | 0 | 66 | 8.309 | 4.4003E-11 | up |
| MELO3C004269.2 | 10 | 38 | 1.966 | 0.000171256 | up |
| MELO3C004272.2 | 37 | 14 | -1.471 | 0.001797356 | down |
| MELO3C004281.2 | 95 | 810 | 3.087 | 2.38493E-84 | up |
| MELO3C004289.2 | 1903 | 0 | -13.51 | 3.22561E-30 | down |
| MELO3C004291.2 | 36 | 0 | -7.786 | 2.11533E-09 | down |
| MELO3C004296.2 | 3153 | 84 | -5.227 | 1.19544E-183 | down |
| MELO3C004301.2 | 716 | 2260 | 1.661 | 8.66038E-22 | up |
| MELO3C004303.2 | 406 | 1056 | 1.381 | 1.66913E-31 | up |
| MELO3C004307.2 | 6453 | 1596 | -2.016 | 5.76202E-83 | down |
| MELO3C004309.2 | 38 | 124 | 1.729 | 2.04068E-06 | up |
| MELO3C004313.2 | 199 | 16 | -3.62 | 4.57323E-24 | down |
| MELO3C004315.2 | 17 | 5 | -1.841 | 0.019551792 | down |
| MELO3C004316.2 | 2594 | 249 | -3.379 | 1.27575E-198 | down |
| MELO3C004317.2 | 159 | 799 | 2.331 | 8.58129E-36 | up |
| MELO3C004318.2 | 307 | 642 | 1.065 | 1.98287E-12 | up |
| MELO3C004320.2 | 0 | 23 | 5.832 | 2.26514E-05 | up |
| MELO3C004321.2 | 444 | 1340 | 1.593 | 3.54775E-19 | up |
| MELO3C004339.2 | 16 | 42 | 1.427 | 0.002348863 | up |
| MELO3C004346.2 | 426 | 130 | -1.715 | 1.82054E-21 | down |
| MELO3C004351.2 | 729 | 15 | -5.586 | 9.72791E-96 | down |
| MELO3C004355.2 | 2126 | 980 | -1.117 | 8.1235E-31 | down |
| MELO3C004358.2 | 2284 | 376 | -2.604 | 3.61078E-127 | down |
| MELO3C004362.2 | 1 | 58 | 5.274 | 1.47862E-08 | up |
| MELO3C004364.2 | 3 | 14 | 2.026 | 0.021358921 | up |
| MELO3C004365.2 | 29 | 118 | 2.033 | 3.24028E-09 | up |
| MELO3C004367.2 | 107 | 379 | 1.822 | 4.47189E-26 | up |
| MELO3C004378.2 | 498 | 144 | -1.797 | 4.13822E-30 | down |
| MELO3C004379.2 | 71 | 170 | 1.26 | 1.63538E-06 | up |
| MELO3C004383.2 | 3988 | 1266 | -1.654 | 2.09676E-55 | down |
| MELO3C004385.2 | 1445 | 269 | -2.425 | 0.003030705 | down |
| MELO3C004388.2 | 3169 | 128 | -4.631 | 8.77419E-239 | down |
| MELO3C004396.2 | 566 | 1138 | 1.008 | 2.74539E-06 | up |
| MELO3C004404.2 | 105 | 20 | -2.422 | 1.65715E-13 | down |
| MELO3C004407.2 | 53 | 197 | 1.889 | 5.29412E-09 | up |
| MELO3C004425.2 | 97 | 267 | 1.467 | 1.01817E-14 | up |
| MELO3C004426.2 | 76 | 213 | 1.487 | 6.54993E-08 | up |
| MELO3C004428.2 | 7227 | 2386 | -1.598 | 4.48684E-09 | down |
| MELO3C004434.2 | 655 | 218 | -1.582 | 9.98696E-22 | down |
| MELO3C004435.2 | 10035 | 1586 | -2.661 | 3.98721E-92 | down |
| MELO3C004442.2 | 1 | 8 | 3.417 | 0.021450323 | up |
| MELO3C004445.2 | 543 | 1105 | 1.027 | 2.77264E-14 | up |
| MELO3C004448.2 | 87 | 471 | 2.441 | 2.19171E-16 | up |
| MELO3C004449.2 | 241 | 30 | -2.989 | 3.2787E-28 | down |
| MELO3C004452.2 | 2485 | 1091 | -1.188 | 4.78994E-27 | down |
| MELO3C004457.2 | 371 | 169 | -1.134 | 5.55159E-13 | down |
| MELO3C004466.2 | 456 | 1671 | 1.875 | 1.03153E-74 | up |
| MELO3C004467.2 | 387 | 1260 | 1.705 | 4.08217E-51 | up |
| MELO3C004474.2 | 216 | 646 | 1.58 | 1.96049E-21 | up |
| MELO3C004478.2 | 92 | 641 | 2.8 | 2.36377E-62 | up |
| MELO3C004488.2 | 933 | 1886 | 1.015 | 1.17042E-19 | up |
| MELO3C004489.2 | 68 | 28 | -1.26 | 0.000400243 | down |
| MELO3C004494.2 | 4 | 0 | -4.649 | 0.02072054 | down |
| MELO3C004495.2 | 125 | 3 | -5.399 | 8.13768E-23 | down |
| MELO3C004497.2 | 27 | 55 | 1.013 | 0.013769943 | up |
| MELO3C004502.2 | 18 | 4 | -2.373 | 0.003090547 | down |
| MELO3C004504.2 | 1749 | 481 | -1.863 | 3.57941E-64 | down |
| MELO3C004506.2 | 3441 | 544 | -2.662 | 1.08665E-135 | down |
| MELO3C004507.2 | 1947 | 919 | -1.084 | 2.04344E-28 | down |
| MELO3C004509.2 | 6 | 26 | 2.083 | 0.004953283 | up |
| MELO3C004511.2 | 12 | 40 | 1.752 | 0.002683265 | up |
| MELO3C004513.2 | 11 | 56 | 2.41 | 1.32E-06 | up |
| MELO3C004515.2 | 18 | 68 | 1.94 | 4.91509E-06 | up |
| MELO3C004516.2 | 390 | 818 | 1.066 | 5.72068E-15 | up |
| MELO3C004520.2 | 115 | 54 | -1.115 | 0.000304118 | down |
| MELO3C004522.2 | 14 | 126 | 3.117 | 9.34812E-16 | up |
| MELO3C004524.2 | 119 | 514 | 2.116 | 3.37237E-34 | up |
| MELO3C004527.2 | 29 | 321 | 3.496 | 8.53413E-37 | up |
| MELO3C004529.2 | 508 | 106 | -2.267 | 1.36722E-23 | down |
| MELO3C004533.2 | 390 | 801 | 1.037 | 1.75578E-11 | up |
| MELO3C004535.2 | 307 | 71 | -2.109 | 5.78224E-20 | down |
| MELO3C004536.2 | 199 | 571 | 1.524 | 1.4209E-25 | up |
| MELO3C004538.2 | 1220 | 2573 | 1.076 | 2.11751E-18 | up |
| MELO3C004539.2 | 246 | 899 | 1.871 | 1.36643E-43 | up |
| MELO3C004543.2 | 188 | 77 | -1.275 | 2.33861E-08 | down |
| MELO3C004544.2 | 549 | 101 | -2.449 | 8.95789E-44 | down |
| MELO3C004548.2 | 32 | 13 | -1.314 | 0.014648881 | down |
| MELO3C004549.2 | 138 | 653 | 2.243 | 2.29218E-46 | up |
| MELO3C004550.2 | 737 | 2927 | 1.99 | 1.0028E-49 | up |
| MELO3C004551.2 | 3567 | 285 | -3.647 | 3.69555E-234 | down |
| MELO3C004553.2 | 1313 | 24 | -5.787 | 8.4174E-154 | down |
| MELO3C004554.2 | 34 | 10 | -1.713 | 0.001446019 | down |
| MELO3C004555.2 | 1564 | 647 | -1.271 | 1.73902E-13 | down |
| MELO3C004556.2 | 2530 | 515 | -2.295 | 2.39911E-116 | down |
| MELO3C004563.2 | 72 | 176 | 1.283 | 1.689E-05 | up |
| MELO3C004574.2 | 4019 | 1380 | -1.542 | 1.23309E-56 | down |
| MELO3C004577.2 | 1615 | 703 | -1.2 | 4.97879E-42 | down |
| MELO3C004578.2 | 1188 | 444 | -1.417 | 2.40436E-34 | down |
| MELO3C004579.2 | 21 | 439 | 4.39 | 5.69284E-56 | up |
| MELO3C004582.2 | 5 | 39 | 2.846 | 0.000493101 | up |
| MELO3C004586.2 | 24 | 1 | -5.353 | 1.26606E-05 | down |
| MELO3C004593.2 | 28 | 3 | -3.465 | 3.24437E-05 | down |
| MELO3C004594.2 | 4 | 15 | 2.016 | 0.017522922 | up |
| MELO3C004597.2 | 126 | 554 | 2.139 | 3.28921E-30 | up |
| MELO3C004604.2 | 55 | 18 | -1.577 | 0.000212148 | down |
| MELO3C004609.2 | 205 | 87 | -1.23 | 0.000260718 | down |
| MELO3C004620.2 | 1 | 14 | 3.597 | 0.002043336 | up |
| MELO3C004623.2 | 1099 | 515 | -1.094 | 4.45405E-14 | down |
| MELO3C004624.2 | 314 | 1136 | 1.857 | 1.66915E-35 | up |
| MELO3C004633.2 | 921 | 4 | -7.808 | 6.231E-72 | down |
| MELO3C004640.2 | 2269 | 582 | -1.964 | 1.74931E-49 | down |
| MELO3C004641.2 | 36 | 275 | 2.95 | 2.04675E-28 | up |
| MELO3C004642.2 | 5857 | 124 | -5.559 | 8.01049E-286 | down |
| MELO3C004657.2 | 23 | 134 | 2.563 | 7.17849E-16 | up |
| MELO3C004658.2 | 675 | 264 | -1.354 | 2.44702E-23 | down |
| MELO3C004659.2 | 1573 | 3680 | 1.226 | 1.56874E-49 | up |
| MELO3C004685.2 | 173 | 24 | -2.882 | 5.67412E-22 | down |
| MELO3C004704.2 | 674 | 1866 | 1.471 | 3.58251E-25 | up |
| MELO3C004728.2 | 83 | 19 | -2.162 | 1.60678E-08 | down |
| MELO3C004732.2 | 1309 | 215 | -2.606 | 1.47976E-73 | down |
| MELO3C004740.2 | 151 | 513 | 1.764 | 3.1311E-19 | up |
| MELO3C004742.2 | 176 | 19 | -3.21 | 5.13495E-25 | down |
| MELO3C004761.2 | 3651 | 1299 | -1.491 | 2.59651E-78 | down |
| MELO3C004787.2 | 266 | 775 | 1.543 | 9.44086E-36 | up |
| MELO3C004797.2 | 23 | 6 | -1.901 | 0.006828845 | down |
| MELO3C004801.2 | 1871 | 44 | -5.4 | 9.97515E-165 | down |
| MELO3C004809.2 | 1001 | 2065 | 1.044 | 2.69519E-18 | up |
| MELO3C004818.2 | 249 | 617 | 1.314 | 1.20201E-19 | up |
| MELO3C004850.2 | 0 | 747 | 11.824 | 9.14926E-17 | up |
| MELO3C004859.2 | 0 | 5 | 4.73 | 0.01081877 | up |
| MELO3C004861.2 | 11 | 0 | -6.021 | 0.000557278 | down |
| MELO3C004866.2 | 1282 | 2729 | 1.091 | 8.35545E-17 | up |
| MELO3C004867.2 | 8160 | 3206 | -1.348 | 1.17764E-32 | down |
| MELO3C004871.2 | 2829 | 343 | -3.045 | 5.59043E-184 | down |
| MELO3C004903.2 | 899 | 370 | -1.279 | 1.22231E-22 | down |
| MELO3C004914.2 | 63 | 0 | -8.594 | 8.39173E-12 | down |
| MELO3C004931.2 | 9 | 0 | -5.77 | 0.000369336 | down |
| MELO3C004936.2 | 99 | 327 | 1.722 | 2.0325E-15 | up |
| MELO3C004941.2 | 1 | 8 | 3.312 | 0.027907286 | up |
| MELO3C004946.2 | 159 | 62 | -1.352 | 9.50124E-07 | down |
| MELO3C004974.2 | 34 | 0 | -7.72 | 1.01391E-08 | down |
| MELO3C004975.2 | 8 | 99 | 3.655 | 4.84718E-14 | up |
| MELO3C004981.2 | 1 | 40 | 4.755 | 4.35548E-07 | up |
| MELO3C004982.2 | 16 | 3 | -2.419 | 0.007871869 | down |
| MELO3C004995.2 | 2167 | 658 | -1.721 | 1.19846E-63 | down |
| MELO3C004998.2 | 2443 | 476 | -2.362 | 2.77618E-34 | down |
| MELO3C004999.2 | 0 | 2484 | 13.556 | 2.19257E-30 | up |
| MELO3C005002.2 | 319 | 122 | -1.394 | 1.38685E-14 | down |
| MELO3C005006.2 | 1582 | 324 | -2.291 | 3.61856E-77 | down |
| MELO3C005009.2 | 18 | 56 | 1.602 | 0.000118893 | up |
| MELO3C005013.2 | 95 | 11 | -3.17 | 3.41396E-15 | down |
| MELO3C005038.2 | 495 | 1120 | 1.177 | 1.59802E-12 | up |
| MELO3C005044.2 | 16 | 1 | -4.82 | 0.00035146 | down |
| MELO3C005058.2 | 1924 | 933 | -1.045 | 2.26441E-32 | down |
| MELO3C005061.2 | 12362 | 5925 | -1.061 | 2.60545E-30 | down |
| MELO3C005070.2 | 24 | 81 | 1.737 | 1.04521E-05 | up |
| MELO3C005079.2 | 533 | 1239 | 1.216 | 6.77195E-31 | up |
| MELO3C005084.2 | 6625 | 3029 | -1.129 | 1.24899E-61 | down |
| MELO3C005085.2 | 45 | 112 | 1.319 | 0.000163622 | up |
| MELO3C005088.2 | 8 | 0 | -5.72 | 0.000481965 | down |
| MELO3C005095.2 | 81 | 547 | 2.762 | 4.73524E-49 | up |
| MELO3C005101.2 | 1828 | 520 | -1.815 | 5.4074E-64 | down |
| MELO3C005129.2 | 1168 | 328 | -1.828 | 1.01369E-29 | down |
| MELO3C005130.2 | 1784 | 635 | -1.49 | 5.82778E-49 | down |
| MELO3C005131.2 | 9048 | 3020 | -1.583 | 9.42559E-73 | down |
| MELO3C005132.2 | 0 | 8 | 5.208 | 0.002822133 | up |
| MELO3C005137.2 | 11 | 104 | 3.252 | 6.56204E-13 | up |
| MELO3C005145.2 | 0 | 38 | 7.52 | 6.74085E-09 | up |
| MELO3C005147.2 | 34 | 248 | 2.873 | 2.53786E-12 | up |
| MELO3C005148.2 | 6 | 129 | 4.352 | 2.93336E-21 | up |
| MELO3C005149.2 | 926 | 439 | -1.075 | 3.08564E-15 | down |
| MELO3C005156.2 | 65 | 1087 | 4.072 | 3.20976E-79 | up |
| MELO3C005169.2 | 76 | 16 | -2.258 | 4.3609E-09 | down |
| MELO3C005179.2 | 18349 | 8545 | -1.103 | 2.57129E-30 | down |
| MELO3C005189.2 | 4859 | 1890 | -1.362 | 5.07005E-69 | down |
| MELO3C005190.2 | 20 | 1 | -4.054 | 9.00012E-05 | down |
| MELO3C005204.2 | 65 | 2 | -4.79 | 1.98258E-13 | down |
| MELO3C005206.2 | 682 | 1406 | 1.042 | 1.06681E-09 | up |
| MELO3C005212.2 | 0 | 53 | 8.016 | 1.87799E-10 | up |
| MELO3C005214.2 | 5091 | 422 | -3.592 | 1.28306E-16 | down |
| MELO3C005215.2 | 81 | 2766 | 5.099 | 7.41863E-29 | up |
| MELO3C005226.2 | 460 | 4472 | 3.279 | 6.67312E-285 | up |
| MELO3C005227.2 | 21 | 6 | -1.779 | 0.005928927 | down |
| MELO3C005229.2 | 379 | 117 | -1.691 | 6.51286E-18 | down |
| MELO3C005231.2 | 2484 | 486 | -2.351 | 9.03431E-36 | down |
| MELO3C005241.2 | 3442 | 8176 | 1.249 | 2.53263E-71 | up |
| MELO3C005250.2 | 248 | 3576 | 3.85 | 6.34741E-184 | up |
| MELO3C005256.2 | 8 | 29 | 1.735 | 0.006308832 | up |
| MELO3C005258.2 | 259 | 11 | -4.503 | 3.46273E-35 | down |
| MELO3C005261.2 | 663 | 261 | -1.346 | 2.56523E-08 | down |
| MELO3C005262.2 | 96 | 239 | 1.317 | 5.95687E-10 | up |
| MELO3C005271.2 | 9 | 27 | 1.622 | 0.01023429 | up |
| MELO3C005278.2 | 0 | 7 | 5.078 | 0.003223071 | up |
| MELO3C005284.2 | 7 | 41 | 2.476 | 0.004209422 | up |
| MELO3C005291.2 | 67 | 14 | -2.207 | 2.36411E-08 | down |
| MELO3C005297.2 | 1065 | 173 | -2.629 | 2.7537E-44 | down |
| MELO3C005310.2 | 2733 | 140 | -4.29 | 7.74091E-284 | down |
| MELO3C005312.2 | 34 | 13 | -1.419 | 0.005355876 | down |
| MELO3C005318.2 | 497 | 172 | -1.534 | 1.54991E-21 | down |
| MELO3C005321.2 | 161 | 55 | -1.545 | 2.1026E-10 | down |
| MELO3C005326.2 | 5 | 0 | -4.049 | 0.024658069 | down |
| MELO3C005330.2 | 12 | 80 | 2.711 | 1.28441E-08 | up |
| MELO3C005332.2 | 2268 | 181 | -3.643 | 1.13082E-193 | down |
| MELO3C005350.2 | 49 | 8 | -2.695 | 0.00230126 | down |
| MELO3C005355.2 | 242 | 4 | -6.093 | 1.31237E-32 | down |
| MELO3C005356.2 | 171 | 434 | 1.338 | 8.23178E-06 | up |
| MELO3C005359.2 | 184 | 718 | 1.963 | 1.45336E-30 | up |
| MELO3C005361.2 | 40 | 113 | 1.502 | 4.55296E-06 | up |
| MELO3C005363.2 | 696 | 93 | -2.896 | 1.6255E-58 | down |
| MELO3C005368.2 | 5364 | 18270 | 1.768 | 4.88384E-102 | up |
| MELO3C005369.2 | 0 | 5 | 4.531 | 0.017290997 | up |
| MELO3C005375.2 | 16 | 56 | 1.85 | 0.000150965 | up |
| MELO3C005381.2 | 29 | 109 | 1.912 | 1.76728E-08 | up |
| MELO3C005388.2 | 2224 | 901 | -1.304 | 3.05673E-35 | down |
| MELO3C005395.2 | 514 | 1139 | 1.147 | 8.43699E-28 | up |
| MELO3C005397.2 | 90 | 19 | -2.215 | 8.29592E-07 | down |
| MELO3C005401.2 | 1340 | 3369 | 1.33 | 9.93712E-55 | up |
| MELO3C005403.2 | 107 | 3 | -5.179 | 5.47226E-21 | down |
| MELO3C005404.2 | 194 | 1496 | 2.952 | 6.23362E-88 | up |
| MELO3C005406.2 | 15 | 47 | 1.709 | 0.002992671 | up |
| MELO3C005408.2 | 10 | 31 | 1.686 | 0.001857864 | up |
| MELO3C005410.2 | 2754 | 1176 | -1.229 | 2.05346E-22 | down |
| MELO3C005414.2 | 46 | 340 | 2.899 | 7.28651E-38 | up |
| MELO3C005415.2 | 991 | 3437 | 1.795 | 1.90254E-42 | up |
| MELO3C005426.2 | 381 | 1446 | 1.921 | 9.17944E-46 | up |
| MELO3C005435.2 | 217 | 0 | -10.377 | 9.04183E-18 | down |
| MELO3C005436.2 | 788 | 6 | -7.06 | 1.42384E-88 | down |
| MELO3C005437.2 | 2914 | 3 | -10.094 | 1.19634E-58 | down |
| MELO3C005439.2 | 2037 | 925 | -1.139 | 1.12909E-34 | down |
| MELO3C005442.2 | 1001 | 184 | -2.441 | 4.86414E-28 | down |
| MELO3C005443.2 | 1548 | 616 | -1.328 | 9.89336E-32 | down |
| MELO3C005448.2 | 3115 | 1470 | -1.084 | 3.47092E-28 | down |
| MELO3C005449.2 | 4213 | 1825 | -1.207 | 4.31621E-43 | down |
| MELO3C005451.2 | 0 | 5 | 4.478 | 0.028742341 | up |
| MELO3C005455.2 | 62 | 143 | 1.202 | 0.00035901 | up |
| MELO3C005458.2 | 266 | 1055 | 1.989 | 3.42866E-32 | up |
| MELO3C005460.2 | 173 | 383 | 1.142 | 1.20141E-12 | up |
| MELO3C005462.2 | 5240 | 348 | -3.915 | 3.54463E-175 | down |
| MELO3C005463.2 | 35 | 2 | -4.034 | 4.77313E-05 | down |
| MELO3C005464.2 | 307 | 1723 | 2.487 | 8.50183E-50 | up |
| MELO3C005465.2 | 2426 | 7261 | 1.582 | 2.65614E-60 | up |
| MELO3C005466.2 | 1877 | 4177 | 1.154 | 7.21816E-25 | up |
| MELO3C005476.2 | 0 | 14 | 6.137 | 3.25861E-05 | up |
| MELO3C005483.2 | 517 | 244 | -1.078 | 3.77516E-11 | down |
| MELO3C005484.2 | 170 | 390 | 1.201 | 4.22786E-12 | up |
| MELO3C005485.2 | 733 | 2389 | 1.704 | 6.40668E-48 | up |
| MELO3C005487.2 | 11 | 60 | 2.408 | 2.21642E-07 | up |
| MELO3C005488.2 | 1625 | 606 | -1.424 | 6.54745E-34 | down |
| MELO3C005492.2 | 159 | 23 | -2.809 | 3.6689E-21 | down |
| MELO3C005495.2 | 268 | 78 | -1.788 | 3.87523E-16 | down |
| MELO3C005504.2 | 1131 | 530 | -1.095 | 7.30195E-23 | down |
| MELO3C005506.2 | 313 | 70 | -2.153 | 9.22495E-26 | down |
| MELO3C005507.2 | 534 | 201 | -1.406 | 1.35921E-17 | down |
| MELO3C005510.2 | 26 | 204 | 2.947 | 2.35912E-23 | up |
| MELO3C005518.2 | 5770 | 2820 | -1.033 | 1.80049E-44 | down |
| MELO3C005524.2 | 125 | 370 | 1.568 | 1.82708E-21 | up |
| MELO3C005526.2 | 1218 | 371 | -1.714 | 0.025991449 | down |
| MELO3C005527.2 | 116 | 297 | 1.364 | 1.5837E-07 | up |
| MELO3C005528.2 | 128 | 58 | -1.163 | 0.000111264 | down |
| MELO3C005529.2 | 25 | 9 | -1.45 | 0.012383387 | down |
| MELO3C005530.2 | 221 | 101 | -1.131 | 6.53192E-08 | down |
| MELO3C005539.2 | 153 | 705 | 2.205 | 1.49437E-48 | up |
| MELO3C005540.2 | 156 | 3670 | 4.555 | 3.9152E-132 | up |
| MELO3C005545.2 | 5 | 0 | -4.888 | 0.010995037 | down |
| MELO3C005556.2 | 1216 | 12 | -6.621 | 6.01517E-138 | down |
| MELO3C005558.2 | 12758 | 2862 | -2.157 | 2.60878E-143 | down |
| MELO3C005559.2 | 4859 | 1338 | -1.862 | 1.43489E-116 | down |
| MELO3C005561.2 | 324 | 763 | 1.236 | 5.19665E-17 | up |
| MELO3C005562.2 | 2376 | 494 | -2.267 | 7.65643E-101 | down |
| MELO3C005564.2 | 4007 | 265 | -3.916 | 1.86305E-106 | down |
| MELO3C005565.2 | 10119 | 2741 | -1.884 | 1.20263E-33 | down |
| MELO3C005566.2 | 11 | 66 | 2.508 | 1.54765E-08 | up |
| MELO3C005567.2 | 8 | 1 | -3.753 | 0.018027426 | down |
| MELO3C005568.2 | 2982 | 634 | -2.237 | 1.92208E-78 | down |
| MELO3C005571.2 | 856 | 4736 | 2.469 | 2.90624E-117 | up |
| MELO3C005574.2 | 74 | 11 | -2.816 | 8.00922E-09 | down |
| MELO3C005576.2 | 0 | 22 | 6.717 | 2.12853E-06 | up |
| MELO3C005577.2 | 15066 | 3966 | -1.925 | 2.14064E-45 | down |
| MELO3C005581.2 | 713 | 168 | -2.084 | 7.61319E-21 | down |
| MELO3C005592.2 | 229 | 42 | -2.446 | 1.24292E-10 | down |
| MELO3C005600.2 | 819 | 1892 | 1.208 | 4.99385E-29 | up |
| MELO3C005601.2 | 209 | 37 | -2.503 | 9.59259E-12 | down |
| MELO3C005602.2 | 97 | 1562 | 4.014 | 2.85243E-12 | up |
| MELO3C005604.2 | 73 | 24 | -1.612 | 0.001532737 | down |
| MELO3C005607.2 | 0 | 431 | 10.066 | 4.22697E-17 | up |
| MELO3C005608.2 | 136 | 477 | 1.815 | 4.07811E-29 | up |
| MELO3C005609.2 | 50 | 623 | 3.657 | 7.17234E-88 | up |
| MELO3C005611.2 | 0 | 6 | 4.875 | 0.00865957 | up |
| MELO3C005616.2 | 13603 | 3372 | -2.012 | 5.08598E-48 | down |
| MELO3C005617.2 | 34 | 12 | -1.48 | 0.003638481 | down |
| MELO3C005621.2 | 993 | 2025 | 1.028 | 6.11097E-33 | up |
| MELO3C005623.2 | 7 | 31 | 2.092 | 0.000913662 | up |
| MELO3C005636.2 | 1 | 10 | 3.116 | 0.017951587 | up |
| MELO3C005639.2 | 3136 | 1352 | -1.215 | 2.97633E-31 | down |
| MELO3C005640.2 | 2233 | 807 | -1.467 | 1.28774E-35 | down |
| MELO3C005651.2 | 5013 | 2208 | -1.183 | 9.0734E-37 | down |
| MELO3C005652.2 | 804 | 7 | -6.836 | 1.55945E-81 | down |
| MELO3C005653.2 | 21 | 84 | 1.991 | 1.11157E-06 | up |
| MELO3C005654.2 | 0 | 10 | 5.57 | 0.000649711 | up |
| MELO3C005656.2 | 18 | 37 | 1.017 | 0.021523198 | up |
| MELO3C005657.2 | 78 | 11 | -2.771 | 1.32006E-10 | down |
| MELO3C005658.2 | 13 | 1 | -4.479 | 0.001015009 | down |
| MELO3C005660.2 | 787 | 162 | -2.282 | 7.5743E-51 | down |
| MELO3C005663.2 | 6066 | 1958 | -1.631 | 5.98932E-98 | down |
| MELO3C005665.2 | 7 | 59 | 3.069 | 4.33537E-07 | up |
| MELO3C005667.2 | 594 | 1424 | 1.259 | 1.9118E-27 | up |
| MELO3C005672.2 | 1173 | 2734 | 1.221 | 2.04385E-48 | up |
| MELO3C005683.2 | 1556 | 581 | -1.419 | 4.92042E-28 | down |
| MELO3C005684.2 | 1277 | 525 | -1.282 | 2.52024E-24 | down |
| MELO3C005685.2 | 14568 | 3615 | -2.011 | 9.14609E-159 | down |
| MELO3C005688.2 | 24564 | 9209 | -1.415 | 3.55139E-57 | down |
| MELO3C005695.2 | 11 | 41 | 1.881 | 0.001455623 | up |
| MELO3C005701.2 | 12 | 796 | 6.116 | 8.44694E-18 | up |
| MELO3C005711.2 | 997 | 2097 | 1.072 | 3.04696E-17 | up |
| MELO3C005714.2 | 85 | 341 | 2.012 | 1.32559E-25 | up |
| MELO3C005716.2 | 204 | 0 | -10.288 | 3.91245E-17 | down |
| MELO3C005717.2 | 1123 | 526 | -1.097 | 1.34084E-15 | down |
| MELO3C005719.2 | 2136 | 880 | -1.28 | 2.49963E-10 | down |
| MELO3C005720.2 | 218 | 626 | 1.525 | 2.99642E-13 | up |
| MELO3C005721.2 | 9 | 24 | 1.51 | 0.015688399 | up |
| MELO3C005729.2 | 769 | 232 | -1.727 | 1.87817E-36 | down |
| MELO3C005732.2 | 78 | 378 | 2.281 | 1.06208E-28 | up |
| MELO3C005734.2 | 15 | 3 | -2.513 | 0.015620152 | down |
| MELO3C005736.2 | 7307 | 3220 | -1.183 | 2.30435E-13 | down |
| MELO3C005737.2 | 223 | 98 | -1.185 | 7.58506E-06 | down |
| MELO3C005739.2 | 477 | 1098 | 1.203 | 8.77892E-26 | up |
| MELO3C005746.2 | 206 | 779 | 1.92 | 1.41466E-33 | up |
| MELO3C005747.2 | 232 | 581 | 1.326 | 2.00011E-16 | up |
| MELO3C005748.2 | 129 | 782 | 2.602 | 9.27661E-30 | up |
| MELO3C005749.2 | 1172 | 578 | -1.021 | 1.30688E-15 | down |
| MELO3C005751.2 | 976 | 193 | -2.336 | 4.14051E-44 | down |
| MELO3C005752.2 | 14 | 2 | -2.949 | 0.006067296 | down |
| MELO3C005759.2 | 3170 | 764 | -2.052 | 6.19396E-137 | down |
| MELO3C005761.2 | 1047 | 2688 | 1.36 | 1.43588E-47 | up |
| MELO3C005763.2 | 6491 | 1094 | -2.57 | 2.15506E-73 | down |
| MELO3C005764.2 | 550 | 125 | -2.147 | 5.4707E-26 | down |
| MELO3C005767.2 | 177 | 1020 | 2.525 | 8.78174E-70 | up |
| MELO3C005769.2 | 278 | 1337 | 2.269 | 2.34374E-55 | up |
| MELO3C005783.2 | 100 | 25 | -1.99 | 4.67459E-07 | down |
| MELO3C005788.2 | 0 | 21 | 6.628 | 1.66323E-06 | up |
| MELO3C005789.2 | 7 | 203 | 4.838 | 1.19235E-30 | up |
| MELO3C005792.2 | 319 | 653 | 1.033 | 5.08957E-08 | up |
| MELO3C005796.2 | 850 | 279 | -1.606 | 9.6598E-19 | down |
| MELO3C005799.2 | 473 | 1170 | 1.306 | 1.54106E-31 | up |
| MELO3C005800.2 | 3204 | 673 | -2.252 | 6.19754E-68 | down |
| MELO3C005801.2 | 922 | 192 | -2.265 | 1.15801E-07 | down |
| MELO3C005805.2 | 88 | 21 | -2.041 | 2.06619E-06 | down |
| MELO3C005806.2 | 4 | 64 | 4.071 | 2.5791E-09 | up |
| MELO3C005811.2 | 2694 | 423 | -2.675 | 7.88596E-71 | down |
| MELO3C005813.2 | 1046 | 4668 | 2.159 | 3.35918E-84 | up |
| MELO3C005816.2 | 123 | 589 | 2.256 | 1.23832E-46 | up |
| MELO3C005819.2 | 102 | 407 | 1.997 | 1.05511E-24 | up |
| MELO3C005821.2 | 452 | 177 | -1.36 | 2.68327E-18 | down |
| MELO3C005822.2 | 12 | 30 | 1.398 | 0.008611791 | up |
| MELO3C005825.2 | 163 | 338 | 1.052 | 7.93065E-09 | up |
| MELO3C005829.2 | 2177 | 654 | -1.737 | 5.40091E-24 | down |
| MELO3C005831.2 | 0 | 10 | 5.568 | 0.000838017 | up |
| MELO3C005832.2 | 160 | 21 | -2.916 | 1.95379E-14 | down |
| MELO3C005834.2 | 6668 | 2313 | -1.528 | 1.13477E-34 | down |
| MELO3C005835.2 | 195 | 97 | -1.003 | 0.000117791 | down |
| MELO3C005847.2 | 333 | 65 | -2.354 | 3.34569E-18 | down |
| MELO3C005853.2 | 28 | 94 | 1.741 | 4.2206E-07 | up |
| MELO3C005855.2 | 72 | 377 | 2.391 | 1.72301E-27 | up |
| MELO3C005858.2 | 39 | 195 | 2.334 | 5.07991E-10 | up |
| MELO3C005869.2 | 6 | 32 | 2.432 | 0.003485525 | up |
| MELO3C005873.2 | 5198 | 2232 | -1.22 | 1.75271E-33 | down |
| MELO3C005874.2 | 851 | 4278 | 2.33 | 8.36458E-132 | up |
| MELO3C005885.2 | 80 | 450 | 2.491 | 9.70701E-14 | up |
| MELO3C005888.2 | 20 | 106 | 2.448 | 3.89967E-07 | up |
| MELO3C005889.2 | 651 | 1397 | 1.101 | 1.76339E-08 | up |
| MELO3C005898.2 | 637 | 2965 | 2.22 | 2.68381E-92 | up |
| MELO3C005899.2 | 358 | 69 | -2.381 | 1.3982E-27 | down |
| MELO3C005902.2 | 244 | 45 | -2.44 | 2.33459E-12 | down |
| MELO3C005903.2 | 21 | 61 | 1.528 | 0.00099625 | up |
| MELO3C005911.2 | 1143 | 288 | -1.988 | 8.08302E-47 | down |
| MELO3C005912.2 | 198 | 539 | 1.438 | 4.88545E-16 | up |
| MELO3C005914.2 | 823 | 95 | -3.116 | 3.7301E-64 | down |
| MELO3C005915.2 | 8652 | 2594 | -1.738 | 8.95209E-106 | down |
| MELO3C005921.2 | 1779 | 5988 | 1.752 | 9.6328E-62 | up |
| MELO3C005928.2 | 206 | 12 | -4.183 | 5.42573E-24 | down |
| MELO3C005937.2 | 54 | 108 | 1.004 | 0.000221477 | up |
| MELO3C005947.2 | 17 | 0 | -6.674 | 3.43026E-06 | down |
| MELO3C005949.2 | 603 | 232 | -1.377 | 5.13891E-21 | down |
| MELO3C005951.2 | 14 | 32 | 1.184 | 0.018527086 | up |
| MELO3C005960.2 | 123 | 360 | 1.552 | 8.29633E-11 | up |
| MELO3C005968.2 | 21 | 133 | 2.64 | 1.66801E-16 | up |
| MELO3C005972.2 | 10 | 99 | 3.286 | 5.45807E-11 | up |
| MELO3C005976.2 | 574 | 1269 | 1.144 | 3.80797E-27 | up |
| MELO3C005977.2 | 577 | 1172 | 1.022 | 2.70676E-12 | up |
| MELO3C005978.2 | 72 | 34 | -1.058 | 0.008923756 | down |
| MELO3C005979.2 | 1030 | 242 | -2.088 | 5.26214E-46 | down |
| MELO3C005983.2 | 423 | 860 | 1.024 | 1.45547E-15 | up |
| MELO3C005987.2 | 7512 | 2294 | -1.711 | 3.26807E-113 | down |
| MELO3C005988.2 | 35 | 105 | 1.579 | 1.03337E-05 | up |
| MELO3C005991.2 | 1175 | 506 | -1.217 | 8.83274E-20 | down |
| MELO3C005992.2 | 4878 | 1084 | -2.169 | 1.10736E-120 | down |
| MELO3C005994.2 | 185 | 477 | 1.368 | 7.94549E-13 | up |
| MELO3C005995.2 | 36 | 2 | -4.368 | 1.43541E-07 | down |
| MELO3C005999.2 | 3527 | 1138 | -1.632 | 5.94219E-39 | down |
| MELO3C006001.2 | 1115 | 136 | -3.04 | 1.56322E-72 | down |
| MELO3C006006.2 | 437 | 993 | 1.184 | 1.77762E-19 | up |
| MELO3C006008.2 | 73 | 24 | -1.599 | 0.000102253 | down |
| MELO3C006014.2 | 74 | 21 | -1.854 | 1.05734E-06 | down |
| MELO3C006019.2 | 1786 | 4121 | 1.206 | 3.46655E-41 | up |
| MELO3C006020.2 | 549 | 1364 | 1.315 | 1.67787E-11 | up |
| MELO3C006023.2 | 949 | 452 | -1.073 | 2.71232E-14 | down |
| MELO3C006025.2 | 494 | 1067 | 1.113 | 1.45788E-12 | up |
| MELO3C006028.2 | 13 | 43 | 1.747 | 0.002156737 | up |
| MELO3C006032.2 | 289 | 7 | -5.291 | 1.04058E-43 | down |
| MELO3C006035.2 | 12827 | 5528 | -1.215 | 6.28663E-29 | down |
| MELO3C006037.2 | 55 | 7 | -2.903 | 0.000102965 | down |
| MELO3C006042.2 | 3988 | 1051 | -1.923 | 5.65452E-40 | down |
| MELO3C006043.2 | 107 | 30 | -1.816 | 9.25178E-07 | down |
| MELO3C006046.2 | 4856 | 2114 | -1.2 | 2.07032E-55 | down |
| MELO3C006047.2 | 67 | 21 | -1.663 | 0.000329005 | down |
| MELO3C006053.2 | 10733 | 1254 | -3.097 | 6.22671E-280 | down |
| MELO3C006056.2 | 256 | 1367 | 2.418 | 4.58474E-79 | up |
| MELO3C006057.2 | 3 | 25 | 2.785 | 0.000219762 | up |
| MELO3C006062.2 | 9 | 53 | 2.576 | 7.34564E-08 | up |
| MELO3C006064.2 | 68 | 158 | 1.221 | 2.33869E-06 | up |
| MELO3C006066.2 | 14 | 35 | 1.326 | 0.008453766 | up |
| MELO3C006067.2 | 0 | 9 | 4.45 | 0.00444574 | up |
| MELO3C006072.2 | 14 | 49 | 1.801 | 0.000184874 | up |
| MELO3C006080.2 | 6 | 39 | 2.614 | 1.15417E-05 | up |
| MELO3C006081.2 | 56 | 176 | 1.655 | 2.39465E-08 | up |
| MELO3C006088.2 | 2955 | 988 | -1.582 | 1.15967E-58 | down |
| MELO3C006090.2 | 211 | 966 | 2.199 | 5.01281E-34 | up |
| MELO3C006094.2 | 343 | 1161 | 1.761 | 8.65943E-54 | up |
| MELO3C006098.2 | 115 | 350 | 1.603 | 2.92904E-14 | up |
| MELO3C006099.2 | 32 | 183 | 2.506 | 3.14736E-18 | up |
| MELO3C006100.2 | 1328 | 616 | -1.107 | 3.72378E-21 | down |
| MELO3C006117.2 | 170 | 506 | 1.569 | 6.62341E-22 | up |
| MELO3C006120.2 | 448 | 994 | 1.149 | 4.78967E-12 | up |
| MELO3C006123.2 | 10331 | 2634 | -1.972 | 2.73237E-175 | down |
| MELO3C006136.2 | 1757 | 820 | -1.099 | 1.10137E-27 | down |
| MELO3C006147.2 | 5972 | 1901 | -1.652 | 1.6033E-79 | down |
| MELO3C006151.2 | 807 | 287 | -1.495 | 1.83961E-26 | down |
| MELO3C006152.2 | 14 | 218 | 3.931 | 4.96032E-30 | up |
| MELO3C006155.2 | 1625 | 490 | -1.732 | 4.85799E-38 | down |
| MELO3C006159.2 | 158 | 40 | -2.004 | 1.97529E-14 | down |
| MELO3C006165.2 | 253 | 692 | 1.449 | 1.50727E-22 | up |
| MELO3C006166.2 | 577 | 1747 | 1.6 | 1.29778E-51 | up |
| MELO3C006172.2 | 4144 | 451 | -3.199 | 0 | down |
| MELO3C006174.2 | 65 | 230 | 1.833 | 1.80976E-15 | up |
| MELO3C006176.2 | 371 | 911 | 1.296 | 2.93653E-25 | up |
| MELO3C006180.2 | 239 | 1075 | 2.171 | 2.97702E-40 | up |
| MELO3C006182.2 | 455 | 140 | -1.701 | 1.85852E-29 | down |
| MELO3C006192.2 | 5312 | 10930 | 1.041 | 1.4771E-26 | up |
| MELO3C006193.2 | 172 | 12 | -3.899 | 9.99345E-23 | down |
| MELO3C006194.2 | 640 | 304 | -1.074 | 3.70954E-19 | down |
| MELO3C006195.2 | 815 | 351 | -1.212 | 2.55473E-12 | down |
| MELO3C006200.2 | 19853 | 3758 | -2.401 | 5.81096E-106 | down |
| MELO3C006202.2 | 926 | 460 | -1.009 | 2.15609E-20 | down |
| MELO3C006227.2 | 1953 | 536 | -1.865 | 3.63858E-69 | down |
| MELO3C006231.2 | 7 | 0 | -5.371 | 0.002155688 | down |
| MELO3C006235.2 | 293 | 99 | -1.571 | 1.7515E-14 | down |
| MELO3C006241.2 | 28 | 75 | 1.434 | 0.000628479 | up |
| MELO3C006248.2 | 99 | 342 | 1.784 | 4.55037E-19 | up |
| MELO3C006251.2 | 173 | 455 | 1.4 | 6.45708E-15 | up |
| MELO3C006252.2 | 62 | 327 | 2.401 | 5.17316E-14 | up |
| MELO3C006254.2 | 7312 | 2331 | -1.649 | 0.000280714 | down |
| MELO3C006256.2 | 150 | 446 | 1.576 | 5.82449E-24 | up |
| MELO3C006262.2 | 20 | 6 | -1.707 | 0.0276157 | down |
| MELO3C006289.2 | 73 | 555 | 2.933 | 3.502E-45 | up |
| MELO3C006291.2 | 8 | 0 | -5.585 | 0.007567391 | down |
| MELO3C006296.2 | 2372 | 1136 | -1.062 | 1.03707E-35 | down |
| MELO3C006299.2 | 236 | 476 | 1.014 | 1.33592E-10 | up |
| MELO3C006313.2 | 426 | 114 | -1.913 | 1.26003E-26 | down |
| MELO3C006315.2 | 34 | 15 | -1.138 | 0.031685226 | down |
| MELO3C006328.2 | 1873 | 4315 | 1.204 | 1.36894E-28 | up |
| MELO3C006334.2 | 9288 | 3456 | -1.426 | 1.54909E-74 | down |
| MELO3C006335.2 | 1018 | 7 | -7.228 | 6.01906E-110 | down |
| MELO3C006341.2 | 16 | 47 | 1.539 | 0.003443761 | up |
| MELO3C006351.2 | 344 | 1060 | 1.624 | 8.86633E-40 | up |
| MELO3C006353.2 | 14 | 54 | 1.924 | 1.677E-05 | up |
| MELO3C006358.2 | 4580 | 1379 | -1.732 | 7.87418E-57 | down |
| MELO3C006359.2 | 61 | 26 | -1.215 | 0.001126737 | down |
| MELO3C006360.2 | 70 | 177 | 1.341 | 8.02082E-06 | up |
| MELO3C006365.2 | 41 | 20 | -1.023 | 0.017127378 | down |
| MELO3C006366.2 | 0 | 10 | 5.557 | 0.000693806 | up |
| MELO3C006373.2 | 4473 | 1226 | -1.868 | 1.07805E-94 | down |
| MELO3C006379.2 | 14 | 37 | 1.459 | 0.005258197 | up |
| MELO3C006380.2 | 111 | 259 | 1.224 | 7.87495E-09 | up |
| MELO3C006392.2 | 165 | 361 | 1.135 | 1.44291E-08 | up |
| MELO3C006400.2 | 2585 | 751 | -1.785 | 2.03408E-49 | down |
| MELO3C006404.2 | 671 | 241 | -1.477 | 1.31693E-21 | down |
| MELO3C006405.2 | 1368 | 512 | -1.42 | 2.13951E-22 | down |
| MELO3C006406.2 | 74 | 183 | 1.316 | 3.83643E-05 | up |
| MELO3C006407.2 | 7377 | 1916 | -1.945 | 1.48078E-106 | down |
| MELO3C006410.2 | 337 | 113 | -1.564 | 7.05779E-16 | down |
| MELO3C006413.2 | 3 | 15 | 2.169 | 0.021486401 | up |
| MELO3C006426.2 | 227 | 103 | -1.147 | 3.27757E-07 | down |
| MELO3C006430.2 | 687 | 132 | -2.378 | 3.77928E-25 | down |
| MELO3C006433.2 | 52 | 26 | -1.016 | 0.019032369 | down |
| MELO3C006436.2 | 228 | 79 | -1.539 | 1.99954E-14 | down |
| MELO3C006439.2 | 364 | 30 | -3.612 | 1.10111E-57 | down |
| MELO3C006443.2 | 2025 | 639 | -1.663 | 1.71628E-44 | down |
| MELO3C006444.2 | 1036 | 271 | -1.934 | 4.11283E-32 | down |
| MELO3C006456.2 | 106 | 52 | -1.016 | 0.005412592 | down |
| MELO3C006464.2 | 124 | 14 | -3.108 | 6.52566E-07 | down |
| MELO3C006465.2 | 89 | 15 | -2.595 | 1.29802E-12 | down |
| MELO3C006467.2 | 126 | 7 | -4.217 | 1.97497E-23 | down |
| MELO3C006470.2 | 45 | 13 | -1.751 | 0.000257983 | down |
| MELO3C006472.2 | 2693 | 1170 | -1.202 | 3.87198E-35 | down |
| MELO3C006473.2 | 971 | 303 | -1.683 | 5.30978E-40 | down |
| MELO3C006475.2 | 8 | 21 | 1.468 | 0.02900257 | up |
| MELO3C006476.2 | 4107 | 10086 | 1.296 | 4.43033E-32 | up |
| MELO3C006482.2 | 855 | 69 | -3.626 | 1.76751E-66 | down |
| MELO3C006483.2 | 23 | 90 | 1.969 | 1.35611E-07 | up |
| MELO3C006489.2 | 455 | 128 | -1.836 | 7.12611E-23 | down |
| MELO3C006490.2 | 12 | 0 | -6.199 | 5.11903E-05 | down |
| MELO3C006492.2 | 11 | 27 | 1.274 | 0.030906118 | up |
| MELO3C006503.2 | 133 | 0 | -9.678 | 1.43803E-15 | down |
| MELO3C006506.2 | 110 | 0 | -9.395 | 1.56097E-14 | down |
| MELO3C006507.2 | 2486 | 5204 | 1.066 | 7.54524E-28 | up |
| MELO3C006510.2 | 895 | 2211 | 1.305 | 7.06801E-30 | up |
| MELO3C006511.2 | 59 | 19 | -1.609 | 0.001044164 | down |
| MELO3C006519.2 | 284 | 82 | -1.798 | 5.35093E-10 | down |
| MELO3C006527.2 | 1846 | 719 | -1.362 | 1.77782E-48 | down |
| MELO3C006532.2 | 301 | 110 | -1.458 | 2.50341E-14 | down |
| MELO3C006533.2 | 914 | 244 | -1.909 | 2.53125E-35 | down |
| MELO3C006539.2 | 36263 | 1237 | -4.874 | 0 | down |
| MELO3C006546.2 | 303 | 1080 | 1.835 | 2.23074E-34 | up |
| MELO3C006552.2 | 56 | 11 | -2.339 | 3.32291E-07 | down |
| MELO3C006553.2 | 244 | 698 | 1.515 | 6.53043E-32 | up |
| MELO3C006561.2 | 55 | 0 | -8.414 | 2.13732E-11 | down |
| MELO3C006562.2 | 3055 | 1197 | -1.351 | 7.2843E-06 | down |
| MELO3C006564.2 | 175 | 1541 | 3.142 | 7.16302E-69 | up |
| MELO3C006569.2 | 1654 | 3871 | 1.227 | 9.30198E-48 | up |
| MELO3C006572.2 | 3101 | 77 | -5.333 | 5.39099E-19 | down |
| MELO3C006574.2 | 322 | 709 | 1.139 | 9.5906E-16 | up |
| MELO3C006577.2 | 9889 | 2846 | -1.797 | 3.22207E-59 | down |
| MELO3C006578.2 | 12 | 2 | -2.997 | 0.00769155 | down |
| MELO3C006580.2 | 10 | 2 | -2.6 | 0.018271624 | down |
| MELO3C006581.2 | 18 | 38 | 1.063 | 0.03093451 | up |
| MELO3C006583.2 | 428 | 2089 | 2.287 | 3.8117E-10 | up |
| MELO3C006585.2 | 10245 | 3347 | -1.614 | 5.32148E-25 | down |
| MELO3C006593.2 | 71 | 30 | -1.242 | 0.000529605 | down |
| MELO3C006602.2 | 149 | 894 | 2.588 | 3.22721E-46 | up |
| MELO3C006605.2 | 493 | 1112 | 1.176 | 1.07577E-27 | up |
| MELO3C006610.2 | 28 | 155 | 2.465 | 4.63657E-14 | up |
| MELO3C006611.2 | 72 | 261 | 1.843 | 1.43507E-16 | up |
| MELO3C006612.2 | 51 | 18 | -1.478 | 0.000474552 | down |
| MELO3C006615.2 | 2293 | 1125 | -1.027 | 5.51948E-23 | down |
| MELO3C006620.2 | 17 | 0 | -6.737 | 2.45125E-06 | down |
| MELO3C006644.2 | 3 | 18 | 2.599 | 0.001679658 | up |
| MELO3C006654.2 | 14 | 34 | 1.252 | 0.026715943 | up |
| MELO3C006657.2 | 435 | 953 | 1.129 | 3.71993E-22 | up |
| MELO3C006662.2 | 791 | 1667 | 1.075 | 1.21844E-19 | up |
| MELO3C006671.2 | 37 | 131 | 1.823 | 6.42308E-10 | up |
| MELO3C006676.2 | 325 | 1142 | 1.815 | 2.72423E-32 | up |
| MELO3C006678.2 | 91 | 1 | -7.272 | 2.21669E-11 | down |
| MELO3C006679.2 | 1163 | 386 | -1.592 | 5.02785E-32 | down |
| MELO3C006682.2 | 176 | 77 | -1.183 | 1.97916E-05 | down |
| MELO3C006685.2 | 496 | 3 | -7.143 | 1.8991E-47 | down |
| MELO3C006688.2 | 15 | 1 | -3.706 | 0.001543678 | down |
| MELO3C006691.2 | 616 | 176 | -1.805 | 2.64332E-29 | down |
| MELO3C006695.2 | 29 | 273 | 3.274 | 4.80624E-16 | up |
| MELO3C006696.2 | 2658 | 1314 | -1.016 | 7.22944E-10 | down |
| MELO3C006698.2 | 70 | 8 | -3.126 | 2.32246E-09 | down |
| MELO3C006700.2 | 17 | 0 | -6.694 | 4.09683E-06 | down |
| MELO3C006702.2 | 10 | 31 | 1.606 | 0.003500971 | up |
| MELO3C006704.2 | 272 | 126 | -1.118 | 2.59161E-09 | down |
| MELO3C006715.2 | 5225 | 1837 | -1.508 | 2.2483E-62 | down |
| MELO3C006716.2 | 371 | 48 | -2.948 | 6.91591E-42 | down |
| MELO3C006717.2 | 8913 | 2297 | -1.956 | 3.74023E-124 | down |
| MELO3C006721.2 | 477 | 137 | -1.801 | 1.81547E-21 | down |
| MELO3C006722.2 | 144 | 369 | 1.361 | 2.03388E-12 | up |
| MELO3C006728.2 | 2 | 43 | 4.289 | 6.85677E-07 | up |
| MELO3C006733.2 | 6383 | 1044 | -2.612 | 8.04588E-200 | down |
| MELO3C006743.2 | 98 | 290 | 1.562 | 1.97068E-17 | up |
| MELO3C006744.2 | 51 | 140 | 1.451 | 3.01384E-06 | up |
| MELO3C006755.2 | 3334 | 1240 | -1.427 | 4.59569E-41 | down |
| MELO3C006757.2 | 41 | 1 | -5.106 | 1.24585E-08 | down |
| MELO3C006763.2 | 818 | 82 | -3.313 | 1.58691E-91 | down |
| MELO3C006765.2 | 2330 | 582 | -2.001 | 2.09031E-101 | down |
| MELO3C006766.2 | 1948 | 324 | -2.591 | 4.9579E-83 | down |
| MELO3C006773.2 | 20 | 55 | 1.472 | 0.000322481 | up |
| MELO3C006777.2 | 61 | 17 | -1.829 | 4.3073E-06 | down |
| MELO3C006783.2 | 11799 | 5234 | -1.173 | 3.51277E-30 | down |
| MELO3C006789.2 | 747 | 2372 | 1.667 | 2.94302E-28 | up |
| MELO3C006800.2 | 6 | 166 | 4.708 | 1.34995E-27 | up |
| MELO3C006801.2 | 395 | 2104 | 2.413 | 5.21383E-73 | up |
| MELO3C006802.2 | 1090 | 506 | -1.109 | 7.1737E-18 | down |
| MELO3C006805.2 | 116 | 1877 | 4.006 | 2.0386E-157 | up |
| MELO3C006807.2 | 60 | 172 | 1.513 | 3.98441E-07 | up |
| MELO3C006808.2 | 104 | 390 | 1.908 | 3.5857E-20 | up |
| MELO3C006810.2 | 323 | 44 | -2.864 | 5.92195E-39 | down |
| MELO3C006811.2 | 904 | 1988 | 1.137 | 2.06735E-27 | up |
| MELO3C006813.2 | 22 | 56 | 1.37 | 0.004259894 | up |
| MELO3C006821.2 | 4450 | 11228 | 1.335 | 1.47023E-65 | up |
| MELO3C006823.2 | 15 | 49 | 1.772 | 0.000603477 | up |
| MELO3C006826.2 | 1323 | 577 | -1.199 | 4.02367E-32 | down |
| MELO3C006827.2 | 98 | 1500 | 3.931 | 1.22772E-155 | up |
| MELO3C006833.2 | 37 | 163 | 2.133 | 1.74263E-12 | up |
| MELO3C006836.2 | 2 | 17 | 3.476 | 0.010746 | up |
| MELO3C006843.2 | 1500 | 4985 | 1.732 | 6.97532E-05 | up |
| MELO3C006847.2 | 53 | 6 | -3.088 | 9.74879E-06 | down |
| MELO3C006851.2 | 33 | 264 | 2.998 | 1.0151E-16 | up |
| MELO3C006852.2 | 61 | 10 | -2.646 | 1.81365E-06 | down |
| MELO3C006863.2 | 389 | 135 | -1.528 | 1.02054E-17 | down |
| MELO3C006865.2 | 36 | 292 | 3.043 | 3.95066E-37 | up |
| MELO3C006866.2 | 2246 | 583 | -1.947 | 1.10436E-78 | down |
| MELO3C006873.2 | 198 | 51 | -1.962 | 1.65495E-12 | down |
| MELO3C006885.2 | 797 | 1996 | 1.323 | 4.61494E-25 | up |
| MELO3C006887.2 | 82 | 214 | 1.378 | 2.14837E-09 | up |
| MELO3C006888.2 | 289 | 90 | -1.689 | 2.78481E-08 | down |
| MELO3C006891.2 | 1624 | 806 | -1.012 | 9.93571E-16 | down |
| MELO3C006895.2 | 2034 | 737 | -1.463 | 1.81959E-32 | down |
| MELO3C006909.2 | 41 | 1834 | 5.49 | 2.8049E-37 | up |
| MELO3C006917.2 | 93 | 715 | 2.942 | 1.14531E-78 | up |
| MELO3C006928.2 | 1384 | 597 | -1.213 | 3.26471E-28 | down |
| MELO3C006930.2 | 22 | 186 | 3.125 | 1.18736E-23 | up |
| MELO3C006933.2 | 13 | 111 | 3.062 | 1.72873E-15 | up |
| MELO3C006942.2 | 119 | 349 | 1.558 | 3.36078E-13 | up |
| MELO3C006944.2 | 275 | 7 | -5.276 | 1.00564E-45 | down |
| MELO3C006945.2 | 1022 | 2113 | 1.048 | 5.41089E-27 | up |
| MELO3C006946.2 | 0 | 13 | 5.959 | 7.73853E-05 | up |
| MELO3C006956.2 | 9 | 30 | 1.795 | 0.002311926 | up |
| MELO3C006958.2 | 718 | 27 | -4.749 | 2.84884E-96 | down |
| MELO3C006961.2 | 1627 | 551 | -1.562 | 3.0592E-51 | down |
| MELO3C006971.2 | 130 | 534 | 2.033 | 1.70979E-29 | up |
| MELO3C006973.2 | 27 | 191 | 2.86 | 2.25206E-17 | up |
| MELO3C006981.2 | 51 | 566 | 3.466 | 9.85205E-61 | up |
| MELO3C006995.2 | 123 | 785 | 2.683 | 5.27334E-33 | up |
| MELO3C006997.2 | 644 | 3458 | 2.426 | 2.02271E-54 | up |
| MELO3C007012.2 | 1200 | 400 | -1.585 | 3.08924E-43 | down |
| MELO3C007014.2 | 1143 | 301 | -1.925 | 8.50726E-54 | down |
| MELO3C007037.2 | 13 | 1213 | 6.491 | 6.05204E-104 | up |
| MELO3C007038.2 | 27 | 354 | 3.685 | 2.63236E-45 | up |
| MELO3C007044.2 | 31 | 9 | -1.867 | 0.00202744 | down |
| MELO3C007045.2 | 13 | 1 | -3.386 | 0.002844044 | down |
| MELO3C007046.2 | 59 | 7 | -3.054 | 8.67629E-09 | down |
| MELO3C007047.2 | 2465 | 916 | -1.426 | 1.16054E-32 | down |
| MELO3C007058.2 | 11 | 75 | 2.741 | 8.2906E-10 | up |
| MELO3C007060.2 | 21 | 1 | -3.882 | 0.000165268 | down |
| MELO3C007069.2 | 14 | 34 | 1.252 | 0.019845247 | up |
| MELO3C007070.2 | 394 | 934 | 1.245 | 1.00592E-16 | up |
| MELO3C007072.2 | 18 | 1 | -3.902 | 0.000227824 | down |
| MELO3C007083.2 | 391 | 1169 | 1.583 | 1.47716E-16 | up |
| MELO3C007091.2 | 17492 | 1119 | -3.968 | 0 | down |
| MELO3C007093.2 | 16 | 118 | 2.894 | 5.5804E-11 | up |
| MELO3C007098.2 | 56 | 113 | 1.009 | 0.000171501 | up |
| MELO3C007100.2 | 5364 | 1963 | -1.45 | 1.16948E-30 | down |
| MELO3C007103.2 | 4247 | 1101 | -1.947 | 4.85629E-137 | down |
| MELO3C007120.2 | 843 | 1995 | 1.243 | 4.79872E-33 | up |
| MELO3C007124.2 | 19039 | 4748 | -2.003 | 8.08293E-96 | down |
| MELO3C007125.2 | 10 | 0 | -5.871 | 0.000587726 | down |
| MELO3C007126.2 | 1160 | 538 | -1.11 | 1.38486E-21 | down |
| MELO3C007127.2 | 964 | 11 | -6.435 | 8.07634E-119 | down |
| MELO3C007132.2 | 15 | 0 | -6.515 | 7.15963E-06 | down |
| MELO3C007138.2 | 445 | 2794 | 2.649 | 8.87472E-53 | up |
| MELO3C007140.2 | 206 | 48 | -2.092 | 1.02873E-12 | down |
| MELO3C007144.2 | 126 | 628 | 2.318 | 7.62649E-25 | up |
| MELO3C007146.2 | 467 | 81 | -2.537 | 3.08087E-35 | down |
| MELO3C007147.2 | 266 | 121 | -1.132 | 2.11287E-07 | down |
| MELO3C007148.2 | 17 | 46 | 1.418 | 0.003152527 | up |
| MELO3C007152.2 | 31 | 3 | -3.24 | 9.38221E-06 | down |
| MELO3C007154.2 | 108658 | 349 | -8.286 | 0 | down |
| MELO3C007157.2 | 14 | 2 | -3.264 | 0.000906428 | down |
| MELO3C007158.2 | 906 | 419 | -1.11 | 2.52606E-20 | down |
| MELO3C007161.2 | 712 | 1754 | 1.301 | 7.46931E-46 | up |
| MELO3C007167.2 | 883 | 1989 | 1.171 | 1.66561E-26 | up |
| MELO3C007174.2 | 290 | 748 | 1.37 | 7.61763E-23 | up |
| MELO3C007185.2 | 168 | 64 | -1.384 | 1.22776E-09 | down |
| MELO3C007197.2 | 6421 | 3206 | -1.002 | 1.18385E-46 | down |
| MELO3C007199.2 | 18 | 40 | 1.163 | 0.020076474 | up |
| MELO3C007203.2 | 1 | 25 | 5.107 | 2.97961E-05 | up |
| MELO3C007204.2 | 3200 | 6739 | 1.074 | 5.55186E-45 | up |
| MELO3C007207.2 | 1453 | 4072 | 1.486 | 1.76714E-39 | up |
| MELO3C007210.2 | 6 | 0 | -5.299 | 0.003091289 | down |
| MELO3C007215.2 | 48 | 129 | 1.426 | 2.04209E-05 | up |
| MELO3C007216.2 | 1 | 10 | 3.189 | 0.01583694 | up |
| MELO3C007217.2 | 1 | 13 | 4.143 | 0.003030189 | up |
| MELO3C007218.2 | 778 | 201 | -1.95 | 1.81302E-37 | down |
| MELO3C007219.2 | 63 | 340 | 2.44 | 1.92638E-31 | up |
| MELO3C007230.2 | 12 | 39 | 1.667 | 0.000615192 | up |
| MELO3C007233.2 | 13507 | 1560 | -3.115 | 1.00602E-305 | down |
| MELO3C007234.2 | 404 | 834 | 1.045 | 5.14693E-20 | up |
| MELO3C007236.2 | 208 | 719 | 1.79 | 3.43983E-37 | up |
| MELO3C007252.2 | 13 | 84 | 2.681 | 1.18344E-10 | up |
| MELO3C007253.2 | 22 | 47 | 1.135 | 0.01281349 | up |
| MELO3C007257.2 | 35 | 1 | -5.889 | 5.44978E-07 | down |
| MELO3C007258.2 | 5 | 0 | -4.779 | 0.017400934 | down |
| MELO3C007259.2 | 20802 | 4160 | -2.322 | 1.12956E-95 | down |
| MELO3C007262.2 | 63 | 29 | -1.144 | 0.002215056 | down |
| MELO3C007263.2 | 216 | 62 | -1.799 | 0.03026313 | down |
| MELO3C007269.2 | 46920 | 14045 | -1.74 | 4.88401E-126 | down |
| MELO3C007271.2 | 22 | 3 | -2.804 | 0.001277686 | down |
| MELO3C007272.2 | 1464 | 692 | -1.08 | 4.41968E-17 | down |
| MELO3C007275.2 | 193 | 16 | -3.566 | 1.31697E-29 | down |
| MELO3C007277.2 | 2930 | 1042 | -1.492 | 4.08693E-54 | down |
| MELO3C007280.2 | 7022 | 1618 | -2.118 | 3.27733E-187 | down |
| MELO3C007281.2 | 1674 | 3793 | 1.18 | 3.32525E-35 | up |
| MELO3C007296.2 | 37 | 98 | 1.383 | 0.00011595 | up |
| MELO3C007297.2 | 885 | 39 | -4.482 | 6.59862E-71 | down |
| MELO3C007307.2 | 23 | 63 | 1.456 | 0.001280152 | up |
| MELO3C007314.2 | 521 | 102 | -2.358 | 1.20178E-44 | down |
| MELO3C007323.2 | 1631 | 376 | -2.118 | 2.26002E-101 | down |
| MELO3C007324.2 | 28 | 4 | -2.96 | 0.000226551 | down |
| MELO3C007325.2 | 304 | 52 | -2.542 | 0.000139836 | down |
| MELO3C007327.2 | 440 | 130 | -1.758 | 1.07495E-22 | down |
| MELO3C007328.2 | 197 | 1 | -7.806 | 2.08364E-19 | down |
| MELO3C007330.2 | 27 | 4 | -2.711 | 0.004224119 | down |
| MELO3C007337.2 | 2 | 21 | 3.785 | 0.000154839 | up |
| MELO3C007340.2 | 357 | 2415 | 2.758 | 9.38878E-68 | up |
| MELO3C007341.2 | 58 | 8 | -2.816 | 9.6638E-09 | down |
| MELO3C007345.2 | 7 | 1 | -3.027 | 0.030455652 | down |
| MELO3C007358.2 | 247 | 32 | -2.94 | 3.30875E-26 | down |
| MELO3C007367.2 | 632 | 1322 | 1.067 | 4.94499E-14 | up |
| MELO3C007375.2 | 95 | 34 | -1.473 | 0.008657294 | down |
| MELO3C007378.2 | 1 | 12 | 2.976 | 0.008477192 | up |
| MELO3C007386.2 | 121 | 25 | -2.271 | 2.51834E-08 | down |
| MELO3C007387.2 | 255 | 69 | -1.879 | 5.08028E-13 | down |
| MELO3C007391.2 | 41 | 19 | -1.123 | 0.028365101 | down |
| MELO3C007396.2 | 268 | 670 | 1.32 | 1.4405E-20 | up |
| MELO3C007403.2 | 10 | 0 | -5.952 | 0.000146969 | down |
| MELO3C007406.2 | 430 | 885 | 1.039 | 3.71743E-18 | up |
| MELO3C007409.2 | 83 | 37 | -1.185 | 0.004585157 | down |
| MELO3C007412.2 | 1800 | 4193 | 1.219 | 1.65038E-42 | up |
| MELO3C007414.2 | 2231 | 1038 | -1.104 | 2.95856E-29 | down |
| MELO3C007418.2 | 1556 | 401 | -1.956 | 1.24462E-48 | down |
| MELO3C007423.2 | 26852 | 7414 | -1.857 | 2.82693E-83 | down |
| MELO3C007428.2 | 313 | 88 | -1.822 | 1.4366E-13 | down |
| MELO3C007430.2 | 32 | 9 | -1.88 | 0.001324192 | down |
| MELO3C007431.2 | 122 | 330 | 1.433 | 1.32995E-17 | up |
| MELO3C007438.2 | 7784 | 1884 | -2.046 | 7.75793E-67 | down |
| MELO3C007440.2 | 405 | 1000 | 1.303 | 4.62395E-25 | up |
| MELO3C007442.2 | 52 | 9 | -2.61 | 5.64481E-07 | down |
| MELO3C007443.2 | 27 | 184 | 2.778 | 4.2868E-22 | up |
| MELO3C007446.2 | 728 | 221 | -1.718 | 3.16232E-41 | down |
| MELO3C007455.2 | 12 | 109 | 3.256 | 1.60832E-10 | up |
| MELO3C007469.2 | 2972 | 1181 | -1.333 | 2.93975E-40 | down |
| MELO3C007472.2 | 801 | 352 | -1.185 | 4.04097E-15 | down |
| MELO3C007481.2 | 11935 | 3461 | -1.786 | 1.28617E-07 | down |
| MELO3C007482.2 | 801 | 36 | -4.466 | 6.17483E-115 | down |
| MELO3C007483.2 | 31539 | 14483 | -1.123 | 1.76966E-38 | down |
| MELO3C007484.2 | 141 | 63 | -1.165 | 5.50592E-06 | down |
| MELO3C007486.2 | 81 | 31 | -1.377 | 5.82489E-05 | down |
| MELO3C007487.2 | 188 | 62 | -1.61 | 4.33803E-09 | down |
| MELO3C007491.2 | 212 | 66 | -1.684 | 2.02526E-11 | down |
| MELO3C007494.2 | 24 | 340 | 3.857 | 1.39107E-08 | up |
| MELO3C007502.2 | 17 | 3 | -2.435 | 0.005754499 | down |
| MELO3C007506.2 | 271 | 630 | 1.218 | 1.87279E-16 | up |
| MELO3C007507.2 | 64385 | 3855 | -4.062 | 0 | down |
| MELO3C007509.2 | 228 | 2756 | 3.6 | 8.05998E-219 | up |
| MELO3C007510.2 | 1069 | 450 | -1.249 | 8.03915E-33 | down |
| MELO3C007512.2 | 8 | 36 | 2.144 | 0.000112436 | up |
| MELO3C007524.2 | 4 | 0 | -4.764 | 0.016877956 | down |
| MELO3C007530.2 | 3 | 52 | 3.956 | 9.33849E-10 | up |
| MELO3C007531.2 | 76 | 33 | -1.223 | 0.000190108 | down |
| MELO3C007533.2 | 1225 | 4880 | 1.995 | 3.16664E-48 | up |
| MELO3C007535.2 | 29 | 0 | -7.499 | 4.77187E-08 | down |
| MELO3C007536.2 | 5 | 30 | 2.522 | 0.000533129 | up |
| MELO3C007537.2 | 1 | 69 | 5.52 | 7.07775E-11 | up |
| MELO3C007538.2 | 1 | 12 | 3.962 | 0.003043458 | up |
| MELO3C007540.2 | 10282 | 5050 | -1.026 | 9.70561E-22 | down |
| MELO3C007545.2 | 124 | 14 | -3.186 | 8.64365E-15 | down |
| MELO3C007547.2 | 9 | 45 | 2.315 | 9.09214E-06 | up |
| MELO3C007549.2 | 29 | 414 | 3.811 | 4.15703E-51 | up |
| MELO3C007554.2 | 36 | 116 | 1.668 | 8.73757E-08 | up |
| MELO3C007562.2 | 911 | 3049 | 1.742 | 1.00323E-35 | up |
| MELO3C007566.2 | 2 | 149 | 6.319 | 1.3387E-16 | up |
| MELO3C007570.2 | 160 | 78 | -1.02 | 2.05305E-05 | down |
| MELO3C007574.2 | 369 | 19 | -4.266 | 1.66494E-57 | down |
| MELO3C007575.2 | 208 | 479 | 1.202 | 2.56958E-15 | up |
| MELO3C007578.2 | 11 | 1 | -3.226 | 0.005895083 | down |
| MELO3C007579.2 | 24 | 2 | -3.743 | 2.35974E-05 | down |
| MELO3C007583.2 | 837 | 234 | -1.836 | 1.01171E-17 | down |
| MELO3C007586.2 | 1420 | 259 | -2.45 | 2.74806E-72 | down |
| MELO3C007589.2 | 21 | 1 | -5.163 | 3.6215E-05 | down |
| MELO3C007590.2 | 4 | 0 | -4.782 | 0.014207142 | down |
| MELO3C007592.2 | 61 | 7 | -3.204 | 2.48489E-09 | down |
| MELO3C007597.2 | 733 | 3322 | 2.18 | 6.14857E-47 | up |
| MELO3C007601.2 | 242 | 2938 | 3.601 | 3.02425E-15 | up |
| MELO3C007602.2 | 113 | 6 | -4.134 | 4.48466E-18 | down |
| MELO3C007609.2 | 2592 | 15960 | 2.623 | 2.71966E-54 | up |
| MELO3C007612.2 | 595 | 1194 | 1.004 | 4.30627E-22 | up |
| MELO3C007613.2 | 4038 | 9079 | 1.169 | 1.47831E-44 | up |
| MELO3C007616.2 | 1558 | 404 | -1.948 | 6.54037E-41 | down |
| MELO3C007617.2 | 94 | 394 | 2.068 | 8.14263E-17 | up |
| MELO3C007618.2 | 91 | 31 | -1.572 | 0.001174527 | down |
| MELO3C007627.2 | 3078 | 204 | -3.91 | 3.55043E-289 | down |
| MELO3C007634.2 | 1 | 68 | 6.492 | 3.09925E-09 | up |
| MELO3C007636.2 | 703 | 336 | -1.064 | 2.54902E-17 | down |
| MELO3C007644.2 | 1376 | 544 | -1.338 | 4.57642E-33 | down |
| MELO3C007649.2 | 8 | 2 | -2.475 | 0.027328665 | down |
| MELO3C007652.2 | 176 | 18 | -3.284 | 1.55189E-14 | down |
| MELO3C007653.2 | 55 | 19 | -1.512 | 0.000526526 | down |
| MELO3C007655.2 | 17 | 76 | 2.195 | 1.97642E-06 | up |
| MELO3C007656.2 | 10 | 26 | 1.336 | 0.025700097 | up |
| MELO3C007658.2 | 76 | 37 | -1.045 | 0.001251972 | down |
| MELO3C007660.2 | 245 | 64 | -1.937 | 3.51614E-12 | down |
| MELO3C007661.2 | 793 | 2284 | 1.526 | 1.71218E-49 | up |
| MELO3C007667.2 | 1102 | 81 | -3.776 | 2.05726E-92 | down |
| MELO3C007670.2 | 2 | 36 | 4.024 | 8.93455E-07 | up |
| MELO3C007672.2 | 11 | 1 | -2.964 | 0.009501232 | down |
| MELO3C007673.2 | 769 | 4497 | 2.549 | 2.12498E-78 | up |
| MELO3C007674.2 | 8700 | 3033 | -1.52 | 5.55354E-46 | down |
| MELO3C007675.2 | 279 | 103 | -1.439 | 2.63818E-08 | down |
| MELO3C007678.2 | 11 | 0 | -6.116 | 8.66449E-05 | down |
| MELO3C007683.2 | 764 | 1767 | 1.21 | 2.49747E-38 | up |
| MELO3C007687.2 | 10 | 78 | 3.016 | 7.259E-08 | up |
| MELO3C007694.2 | 224 | 1150 | 2.359 | 2.16556E-55 | up |
| MELO3C007696.2 | 5 | 22 | 2.308 | 0.001397422 | up |
| MELO3C007698.2 | 12888 | 703 | -4.197 | 4.87892E-172 | down |
| MELO3C007699.2 | 165 | 595 | 1.847 | 8.15382E-22 | up |
| MELO3C007702.2 | 278 | 3463 | 3.64 | 6.96202E-73 | up |
| MELO3C007707.2 | 5 | 114 | 4.46 | 6.41297E-17 | up |
| MELO3C007708.2 | 14 | 2 | -3.025 | 0.002905719 | down |
| MELO3C007715.2 | 1221 | 4029 | 1.722 | 5.22775E-90 | up |
| MELO3C007731.2 | 43 | 5 | -3.191 | 7.1485E-07 | down |
| MELO3C007742.2 | 3214 | 7132 | 1.15 | 7.2527E-40 | up |
| MELO3C007747.2 | 1156 | 377 | -1.619 | 8.83327E-32 | down |
| MELO3C007753.2 | 8 | 0 | -5.614 | 0.001380466 | down |
| MELO3C007760.2 | 24 | 130 | 2.415 | 1.58817E-11 | up |
| MELO3C007762.2 | 770 | 2271 | 1.56 | 9.69607E-38 | up |
| MELO3C007766.2 | 404 | 869 | 1.107 | 6.21253E-21 | up |
| MELO3C007767.2 | 1763 | 5304 | 1.589 | 1.07823E-27 | up |
| MELO3C007769.2 | 3583 | 1547 | -1.212 | 5.99238E-24 | down |
| MELO3C007773.2 | 1187 | 270 | -2.133 | 5.61975E-38 | down |
| MELO3C007774.2 | 84 | 24 | -1.807 | 0.002788114 | down |
| MELO3C007779.2 | 36 | 91 | 1.364 | 0.000404151 | up |
| MELO3C007781.2 | 3969 | 13371 | 1.753 | 3.27954E-57 | up |
| MELO3C007785.2 | 2570 | 1233 | -1.06 | 7.82393E-25 | down |
| MELO3C007794.2 | 144 | 32 | -2.16 | 1.53633E-14 | down |
| MELO3C007797.2 | 7 | 1 | -3.635 | 0.024103018 | down |
| MELO3C007798.2 | 332 | 77 | -2.105 | 9.82314E-14 | down |
| MELO3C007799.2 | 4149 | 989 | -2.069 | 1.14192E-51 | down |
| MELO3C007809.2 | 24 | 4 | -2.72 | 0.005302892 | down |
| MELO3C007817.2 | 4 | 15 | 2.01 | 0.031431193 | up |
| MELO3C007820.2 | 6600 | 1153 | -2.518 | 1.21516E-96 | down |
| MELO3C007827.2 | 15 | 97 | 2.696 | 3.10546E-09 | up |
| MELO3C007837.2 | 80 | 431 | 2.429 | 2.66454E-40 | up |
| MELO3C007838.2 | 1831 | 384 | -2.25 | 1.55661E-77 | down |
| MELO3C007839.2 | 835 | 373 | -1.163 | 1.17874E-26 | down |
| MELO3C007840.2 | 92 | 39 | -1.252 | 0.000314235 | down |
| MELO3C007842.2 | 406 | 892 | 1.137 | 7.00405E-14 | up |
| MELO3C007844.2 | 264 | 58 | -2.18 | 5.9219E-20 | down |
| MELO3C007845.2 | 94 | 323 | 1.793 | 6.19864E-15 | up |
| MELO3C007846.2 | 508 | 1883 | 1.89 | 8.71513E-39 | up |
| MELO3C007849.2 | 80 | 0 | -8.937 | 7.83936E-13 | down |
| MELO3C007850.2 | 10 | 74 | 2.9 | 6.38887E-07 | up |
| MELO3C007853.2 | 5 | 0 | -4.91 | 0.011554211 | down |
| MELO3C007859.2 | 650 | 1819 | 1.487 | 1.08055E-23 | up |
| MELO3C007863.2 | 5 | 195 | 5.32 | 8.78089E-28 | up |
| MELO3C007868.2 | 102 | 11 | -3.318 | 0.014228746 | down |
| MELO3C007871.2 | 419 | 1351 | 1.688 | 8.39607E-32 | up |
| MELO3C007872.2 | 182 | 9227 | 5.661 | 1.50208E-48 | up |
| MELO3C007873.2 | 65 | 132 | 1.039 | 0.003355669 | up |
| MELO3C007874.2 | 279 | 59 | -2.232 | 4.51789E-15 | down |
| MELO3C007877.2 | 64 | 302 | 2.233 | 5.96245E-24 | up |
| MELO3C007884.2 | 822 | 119 | -2.792 | 2.21101E-23 | down |
| MELO3C007889.2 | 36259 | 17639 | -1.04 | 2.64459E-37 | down |
| MELO3C007893.2 | 234 | 99 | -1.24 | 1.18997E-06 | down |
| MELO3C007896.2 | 1302 | 637 | -1.032 | 3.21306E-19 | down |
| MELO3C007901.2 | 24 | 67 | 1.463 | 0.00012093 | up |
| MELO3C007907.2 | 111 | 226 | 1.022 | 3.758E-06 | up |
| MELO3C007908.2 | 8823 | 3274 | -1.43 | 1.55559E-90 | down |
| MELO3C007917.2 | 120 | 24 | -2.325 | 5.57288E-12 | down |
| MELO3C007919.2 | 193 | 541 | 1.49 | 8.12751E-17 | up |
| MELO3C007920.2 | 1006 | 2490 | 1.309 | 1.68692E-50 | up |
| MELO3C007926.2 | 0 | 5 | 4.636 | 0.016160387 | up |
| MELO3C007927.2 | 1 | 29 | 4.697 | 4.92247E-06 | up |
| MELO3C007934.2 | 399 | 137 | -1.546 | 2.55508E-21 | down |
| MELO3C007935.2 | 174 | 73 | -1.246 | 8.45364E-09 | down |
| MELO3C007936.2 | 1 | 9 | 3.61 | 0.013024511 | up |
| MELO3C007938.2 | 26 | 185 | 2.805 | 3.6577E-22 | up |
| MELO3C007948.2 | 368 | 758 | 1.043 | 4.20714E-12 | up |
| MELO3C007949.2 | 5262 | 127 | -5.378 | 2.89742E-243 | down |
| MELO3C007950.2 | 1085 | 437 | -1.313 | 5.32031E-28 | down |
| MELO3C007956.2 | 45 | 112 | 1.342 | 1.65662E-05 | up |
| MELO3C007962.2 | 29 | 1 | -5.635 | 4.64804E-06 | down |
| MELO3C007967.2 | 181 | 5 | -5.106 | 1.11734E-31 | down |
| MELO3C007969.2 | 555 | 126 | -2.135 | 4.99039E-47 | down |
| MELO3C007979.2 | 562 | 124 | -2.175 | 4.10957E-18 | down |
| MELO3C007986.2 | 1023 | 404 | -1.34 | 2.63569E-31 | down |
| MELO3C007989.2 | 1152 | 2692 | 1.224 | 9.49504E-43 | up |
| MELO3C007990.2 | 63 | 297 | 2.238 | 8.40793E-22 | up |
| MELO3C007995.2 | 143 | 49 | -1.553 | 3.84118E-09 | down |
| MELO3C007997.2 | 439 | 914 | 1.059 | 1.58806E-17 | up |
| MELO3C008001.2 | 639 | 274 | -1.22 | 7.46419E-20 | down |
| MELO3C008004.2 | 42 | 19 | -1.18 | 0.008242451 | down |
| MELO3C008005.2 | 334 | 1348 | 2.013 | 1.51232E-70 | up |
| MELO3C008006.2 | 32 | 173 | 2.452 | 2.17007E-19 | up |
| MELO3C008008.2 | 1085 | 377 | -1.529 | 7.4687E-31 | down |
| MELO3C008025.2 | 15 | 61 | 1.977 | 2.56835E-05 | up |
| MELO3C008026.2 | 65 | 13 | -2.371 | 8.01217E-08 | down |
| MELO3C008028.2 | 37 | 297 | 3.018 | 2.69488E-31 | up |
| MELO3C008029.2 | 0 | 7 | 4.989 | 0.005184313 | up |
| MELO3C008039.2 | 176 | 27 | -2.693 | 9.03901E-19 | down |
| MELO3C008045.2 | 68 | 155 | 1.186 | 2.74585E-05 | up |
| MELO3C008050.2 | 3 | 31 | 3.222 | 7.25488E-06 | up |
| MELO3C008051.2 | 2312 | 864 | -1.419 | 3.34691E-33 | down |
| MELO3C008059.2 | 1249 | 452 | -1.467 | 3.1877E-38 | down |
| MELO3C008060.2 | 18140 | 321 | -5.82 | 0 | down |
| MELO3C008063.2 | 4434 | 1088 | -2.027 | 1.68718E-101 | down |
| MELO3C008077.2 | 18 | 165 | 3.207 | 8.30234E-18 | up |
| MELO3C008080.2 | 693 | 304 | -1.192 | 5.03215E-14 | down |
| MELO3C008084.2 | 13 | 0 | -6.339 | 4.53308E-05 | down |
| MELO3C008085.2 | 509 | 1263 | 1.31 | 1.64311E-26 | up |
| MELO3C008086.2 | 5636 | 2192 | -1.362 | 4.45535E-63 | down |
| MELO3C008091.2 | 126 | 11 | -3.47 | 1.67261E-18 | down |
| MELO3C008097.2 | 0 | 420 | 10.995 | 2.99384E-20 | up |
| MELO3C008100.2 | 471 | 26 | -4.152 | 2.07351E-51 | down |
| MELO3C008114.2 | 4674 | 610 | -2.938 | 5.36141E-262 | down |
| MELO3C008115.2 | 1547 | 485 | -1.673 | 6.15354E-60 | down |
| MELO3C008116.2 | 468 | 164 | -1.507 | 5.06629E-15 | down |
| MELO3C008124.2 | 40 | 105 | 1.384 | 1.83001E-06 | up |
| MELO3C008134.2 | 477 | 1479 | 1.634 | 2.05857E-37 | up |
| MELO3C008137.2 | 28 | 71 | 1.337 | 0.00142493 | up |
| MELO3C008140.2 | 573 | 170 | -1.759 | 1.91764E-17 | down |
| MELO3C008143.2 | 19 | 7 | -1.533 | 0.028158606 | down |
| MELO3C008145.2 | 45 | 128 | 1.509 | 1.09184E-05 | up |
| MELO3C008149.2 | 380 | 37 | -3.325 | 3.42893E-35 | down |
| MELO3C008160.2 | 192 | 87 | -1.151 | 2.54713E-06 | down |
| MELO3C008161.2 | 20 | 120 | 2.612 | 2.83618E-13 | up |
| MELO3C008163.2 | 2631 | 7844 | 1.576 | 4.7659E-64 | up |
| MELO3C008164.2 | 282 | 15 | -4.228 | 1.57426E-51 | down |
| MELO3C008171.2 | 648 | 1759 | 1.441 | 2.95393E-50 | up |
| MELO3C008173.2 | 823 | 359 | -1.197 | 5.43854E-11 | down |
| MELO3C008175.2 | 606 | 160 | -1.926 | 1.41216E-26 | down |
| MELO3C008183.2 | 947 | 3 | -8.092 | 1.01395E-21 | down |
| MELO3C008191.2 | 2414 | 896 | -1.43 | 7.02352E-39 | down |
| MELO3C008195.2 | 66 | 395 | 2.585 | 2.78365E-30 | up |
| MELO3C008196.2 | 0 | 9 | 5.473 | 0.000887872 | up |
| MELO3C008197.2 | 2216 | 895 | -1.307 | 2.84233E-18 | down |
| MELO3C008201.2 | 459 | 209 | -1.136 | 2.4024E-11 | down |
| MELO3C008203.2 | 42 | 1177 | 4.82 | 3.79465E-74 | up |
| MELO3C008205.2 | 9 | 0 | -5.706 | 0.00059845 | down |
| MELO3C008211.2 | 128 | 443 | 1.79 | 1.35219E-19 | up |
| MELO3C008214.2 | 7939 | 3824 | -1.054 | 3.76462E-27 | down |
| MELO3C008216.2 | 942 | 354 | -1.412 | 6.21996E-30 | down |
| MELO3C008226.2 | 110 | 236 | 1.099 | 3.35617E-07 | up |
| MELO3C008231.2 | 19075 | 6694 | -1.511 | 1.17085E-38 | down |
| MELO3C008233.2 | 1555 | 207 | -2.916 | 8.21458E-152 | down |
| MELO3C008236.2 | 267 | 703 | 1.397 | 6.66816E-19 | up |
| MELO3C008241.2 | 4684 | 9372 | 1.001 | 2.71404E-34 | up |
| MELO3C008244.2 | 1547 | 4603 | 1.573 | 8.19422E-91 | up |
| MELO3C008250.2 | 0 | 74 | 8.493 | 8.86982E-12 | up |
| MELO3C008252.2 | 0 | 14 | 6.035 | 0.00023455 | up |
| MELO3C008255.2 | 632 | 207 | -1.615 | 1.50357E-28 | down |
| MELO3C008261.2 | 5 | 80 | 3.829 | 6.24405E-13 | up |
| MELO3C008262.2 | 129 | 27 | -2.256 | 1.1815E-11 | down |
| MELO3C008263.2 | 9 | 0 | -5.808 | 0.000657435 | down |
| MELO3C008269.2 | 41 | 214 | 2.397 | 6.34138E-15 | up |
| MELO3C008272.2 | 8 | 32 | 2.072 | 0.002546004 | up |
| MELO3C008281.2 | 53 | 1 | -5.513 | 6.47469E-09 | down |
| MELO3C008286.2 | 1769 | 550 | -1.688 | 8.51855E-51 | down |
| MELO3C008287.2 | 6 | 230 | 5.176 | 1.61567E-28 | up |
| MELO3C008288.2 | 3284 | 889 | -1.886 | 5.27777E-94 | down |
| MELO3C008294.2 | 21 | 6 | -1.843 | 0.006178051 | down |
| MELO3C008313.2 | 2298 | 835 | -1.462 | 1.66092E-42 | down |
| MELO3C008314.2 | 1046 | 120 | -3.12 | 4.57001E-112 | down |
| MELO3C008318.2 | 1685 | 574 | -1.556 | 2.00297E-48 | down |
| MELO3C008319.2 | 113 | 4 | -4.809 | 0.000153929 | down |
| MELO3C008332.2 | 500 | 122 | -2.039 | 5.04924E-42 | down |
| MELO3C008337.2 | 0 | 80 | 8.606 | 3.52024E-12 | up |
| MELO3C008342.2 | 41 | 1 | -5.128 | 5.22061E-09 | down |
| MELO3C008350.2 | 219 | 22 | -3.298 | 3.51852E-17 | down |
| MELO3C008366.2 | 1853 | 15917 | 3.102 | 0.000604593 | up |
| MELO3C008375.2 | 9 | 328 | 5.185 | 1.44443E-40 | up |
| MELO3C008381.2 | 0 | 137 | 9.373 | 4.754E-14 | up |
| MELO3C008386.2 | 52 | 19 | -1.392 | 0.003753026 | down |
| MELO3C008394.2 | 0 | 13 | 5.972 | 9.39193E-05 | up |
| MELO3C008404.2 | 41 | 88 | 1.105 | 0.005326788 | up |
| MELO3C008410.2 | 30 | 6 | -2.439 | 0.000531438 | down |
| MELO3C008415.2 | 6938 | 2278 | -1.607 | 9.25531E-50 | down |
| MELO3C008424.2 | 33 | 1092 | 5.06 | 1.10643E-87 | up |
| MELO3C008429.2 | 8015 | 3378 | -1.246 | 1.58696E-58 | down |
| MELO3C008433.2 | 3042 | 839 | -1.859 | 3.51621E-51 | down |
| MELO3C008435.2 | 709 | 258 | -1.459 | 9.02007E-20 | down |
| MELO3C008436.2 | 408 | 0 | -11.289 | 3.59816E-21 | down |
| MELO3C008440.2 | 2 | 39 | 4.124 | 1.24813E-06 | up |
| MELO3C008456.2 | 175 | 27 | -2.708 | 1.23937E-22 | down |
| MELO3C008469.2 | 1952 | 13673 | 2.809 | 1.61017E-184 | up |
| MELO3C008477.2 | 2582 | 1030 | -1.326 | 1.61207E-12 | down |
| MELO3C008491.2 | 135 | 54 | -1.321 | 8.62316E-06 | down |
| MELO3C008492.2 | 143 | 1416 | 3.313 | 7.005E-153 | up |
| MELO3C008498.2 | 12 | 102 | 3.065 | 6.89E-15 | up |
| MELO3C008499.2 | 416 | 198 | -1.08 | 1.20517E-06 | down |
| MELO3C008513.2 | 2438 | 1197 | -1.026 | 5.04997E-27 | down |
| MELO3C008524.2 | 2193 | 364 | -2.59 | 7.64117E-94 | down |
| MELO3C008529.2 | 61 | 323 | 2.419 | 2.55317E-30 | up |
| MELO3C008530.2 | 4 | 58 | 3.837 | 1.4366E-09 | up |
| MELO3C008553.2 | 126 | 445 | 1.827 | 1.80908E-30 | up |
| MELO3C008570.2 | 209 | 5 | -5.291 | 8.99873E-38 | down |
| MELO3C008578.2 | 192 | 1160 | 2.594 | 2.09606E-76 | up |
| MELO3C008581.2 | 82 | 34 | -1.265 | 0.00053855 | down |
| MELO3C008596.2 | 635 | 1614 | 1.345 | 4.50022E-38 | up |
| MELO3C008598.2 | 3321 | 1252 | -1.409 | 1.21003E-43 | down |
| MELO3C008645.2 | 9 | 36 | 1.933 | 0.000236176 | up |
| MELO3C008647.2 | 2 | 117 | 5.717 | 3.42724E-17 | up |
| MELO3C008648.2 | 5 | 99 | 4.238 | 1.03942E-14 | up |
| MELO3C008657.2 | 447 | 4 | -6.856 | 8.8436E-49 | down |
| MELO3C008659.2 | 347 | 2 | -7.62 | 5.90657E-35 | down |
| MELO3C008663.2 | 16512 | 99 | -7.393 | 0 | down |
| MELO3C008669.2 | 34 | 158 | 2.202 | 1.01646E-16 | up |
| MELO3C008672.2 | 450 | 170 | -1.401 | 1.15726E-10 | down |
| MELO3C008677.2 | 1396 | 425 | -1.716 | 8.16045E-36 | down |
| MELO3C008679.2 | 52 | 5 | -3.369 | 1.88959E-09 | down |
| MELO3C008688.2 | 0 | 31 | 7.241 | 6.34984E-08 | up |
| MELO3C008693.2 | 2 | 47 | 4.967 | 2.95619E-08 | up |
| MELO3C008696.2 | 46 | 0 | -8.131 | 1.8416E-10 | down |
| MELO3C008699.2 | 2475 | 253 | -3.293 | 2.88321E-216 | down |
| MELO3C008731.2 | 37859 | 241 | -7.297 | 0 | down |
| MELO3C008738.2 | 0 | 10 | 5.554 | 0.000449768 | up |
| MELO3C008739.2 | 140 | 517 | 1.878 | 4.75182E-19 | up |
| MELO3C008742.2 | 1 | 66 | 5.878 | 7.14107E-10 | up |
| MELO3C008751.2 | 18 | 100 | 2.503 | 3.97729E-11 | up |
| MELO3C008767.2 | 214 | 499 | 1.226 | 3.72283E-15 | up |
| MELO3C008798.2 | 205 | 7 | -4.928 | 3.92679E-34 | down |
| MELO3C008799.2 | 384 | 95 | -2.002 | 1.48572E-19 | down |
| MELO3C008801.2 | 948 | 2598 | 1.455 | 7.7096E-41 | up |
| MELO3C008802.2 | 644 | 212 | -1.602 | 0.002580201 | down |
| MELO3C008835.2 | 681 | 269 | -1.337 | 2.55445E-27 | down |
| MELO3C008839.2 | 38 | 79 | 1.039 | 0.002557531 | up |
| MELO3C008846.2 | 412 | 159 | -1.369 | 1.00745E-16 | down |
| MELO3C008855.2 | 5 | 36 | 2.907 | 9.55931E-06 | up |
| MELO3C008856.2 | 658 | 250 | -1.397 | 7.35206E-24 | down |
| MELO3C008866.2 | 67 | 21 | -1.675 | 4.53137E-05 | down |
| MELO3C008882.2 | 8 | 0 | -4.626 | 0.006135934 | down |
| MELO3C008885.2 | 53 | 591 | 3.478 | 1.32958E-77 | up |
| MELO3C008894.2 | 72 | 2 | -4.954 | 5.44101E-09 | down |
| MELO3C008895.2 | 328 | 26 | -3.638 | 7.84575E-40 | down |
| MELO3C008898.2 | 1027 | 387 | -1.407 | 6.30908E-26 | down |
| MELO3C008899.2 | 535 | 1931 | 1.852 | 1.55476E-35 | up |
| MELO3C008910.2 | 229 | 585 | 1.352 | 3.18201E-22 | up |
| MELO3C008920.2 | 5025 | 908 | -2.469 | 1.63558E-67 | down |
| MELO3C008923.2 | 50 | 670 | 3.746 | 9.97871E-79 | up |
| MELO3C008949.2 | 752 | 347 | -1.116 | 6.54732E-23 | down |
| MELO3C008963.2 | 121 | 14 | -3.189 | 1.59106E-19 | down |
| MELO3C009001.2 | 50 | 133 | 1.425 | 2.68094E-07 | up |
| MELO3C009006.2 | 19 | 1 | -4.991 | 0.000373564 | down |
| MELO3C009007.2 | 108 | 503 | 2.223 | 3.99587E-44 | up |
| MELO3C009010.2 | 195 | 89 | -1.12 | 5.9002E-06 | down |
| MELO3C009016.2 | 50 | 216 | 2.117 | 1.00728E-15 | up |
| MELO3C009019.2 | 2054 | 475 | -2.114 | 1.87817E-100 | down |
| MELO3C009025.2 | 26 | 54 | 1.083 | 0.003876874 | up |
| MELO3C009030.2 | 46 | 0 | -8.126 | 1.9349E-10 | down |
| MELO3C009032.2 | 3504 | 1680 | -1.06 | 1.42447E-44 | down |
| MELO3C009053.2 | 569 | 1531 | 1.43 | 5.16891E-24 | up |
| MELO3C009076.2 | 612 | 5258 | 3.105 | 1.40832E-125 | up |
| MELO3C009078.2 | 169 | 36 | -2.227 | 1.86023E-14 | down |
| MELO3C009089.2 | 2577 | 6390 | 1.31 | 3.97835E-35 | up |
| MELO3C009091.2 | 2825 | 1143 | -1.305 | 3.21642E-44 | down |
| MELO3C009092.2 | 47 | 6 | -2.99 | 1.71733E-07 | down |
| MELO3C009097.2 | 3238 | 463 | -2.806 | 1.24719E-05 | down |
| MELO3C009098.2 | 10 | 1 | -3.556 | 0.013127157 | down |
| MELO3C009104.2 | 1951 | 663 | -1.559 | 1.9752E-58 | down |
| MELO3C009106.2 | 7 | 29 | 2.104 | 0.000769416 | up |
| MELO3C009107.2 | 12 | 105 | 3.165 | 1.46092E-12 | up |
| MELO3C009116.2 | 27 | 68 | 1.326 | 0.001472897 | up |
| MELO3C009121.2 | 11 | 60 | 2.521 | 8.94364E-08 | up |
| MELO3C009122.2 | 22 | 381 | 4.145 | 5.6659E-10 | up |
| MELO3C009133.2 | 180 | 765 | 2.091 | 2.42357E-39 | up |
| MELO3C009135.2 | 127 | 315 | 1.31 | 3.36988E-12 | up |
| MELO3C009137.2 | 5840 | 2575 | -1.181 | 7.43164E-67 | down |
| MELO3C009143.2 | 123 | 47 | -1.375 | 7.37515E-06 | down |
| MELO3C009146.2 | 1256 | 482 | -1.382 | 3.00353E-39 | down |
| MELO3C009147.2 | 840 | 290 | -1.535 | 8.71399E-25 | down |
| MELO3C009150.2 | 1435 | 683 | -1.071 | 1.06306E-25 | down |
| MELO3C009154.2 | 9 | 0 | -5.734 | 0.003786604 | down |
| MELO3C009155.2 | 8 | 0 | -5.564 | 0.001387023 | down |
| MELO3C009156.2 | 8 | 0 | -5.662 | 0.000597436 | down |
| MELO3C009164.2 | 297 | 774 | 1.383 | 1.19633E-26 | up |
| MELO3C009166.2 | 352 | 1121 | 1.673 | 2.31592E-46 | up |
| MELO3C009167.2 | 760 | 250 | -1.604 | 1.68156E-32 | down |
| MELO3C009177.2 | 1367 | 2831 | 1.05 | 2.9079E-19 | up |
| MELO3C009185.2 | 91 | 250 | 1.453 | 6.43148E-10 | up |
| MELO3C009186.2 | 3482 | 1510 | -1.206 | 7.90829E-39 | down |
| MELO3C009187.2 | 6227 | 1393 | -2.159 | 1.43766E-62 | down |
| MELO3C009190.2 | 432 | 84 | -2.375 | 1.43611E-20 | down |
| MELO3C009192.2 | 105 | 1969 | 4.237 | 4.82454E-186 | up |
| MELO3C009194.2 | 119 | 242 | 1.026 | 3.6536E-07 | up |
| MELO3C009197.2 | 635 | 1333 | 1.07 | 1.15525E-21 | up |
| MELO3C009199.2 | 162 | 337 | 1.06 | 3.96569E-09 | up |
| MELO3C009200.2 | 792 | 254 | -1.641 | 3.73834E-43 | down |
| MELO3C009203.2 | 760 | 12601 | 4.051 | 1.90559E-45 | up |
| MELO3C009204.2 | 2 | 16 | 2.824 | 0.004501216 | up |
| MELO3C009205.2 | 51 | 2 | -4.84 | 3.06078E-09 | down |
| MELO3C009210.2 | 1728 | 450 | -1.939 | 2.54524E-19 | down |
| MELO3C009213.2 | 406 | 1261 | 1.632 | 8.75163E-33 | up |
| MELO3C009219.2 | 7 | 189 | 4.805 | 1.55699E-28 | up |
| MELO3C009221.2 | 292 | 97 | -1.584 | 1.61432E-15 | down |
| MELO3C009222.2 | 4382 | 1585 | -1.467 | 9.59529E-60 | down |
| MELO3C009223.2 | 411 | 171 | -1.276 | 4.30235E-12 | down |
| MELO3C009226.2 | 2930 | 1338 | -1.132 | 7.82216E-34 | down |
| MELO3C009229.2 | 25 | 7 | -1.902 | 0.003648477 | down |
| MELO3C009230.2 | 260 | 735 | 1.5 | 3.34689E-34 | up |
| MELO3C009232.2 | 14 | 3 | -2.275 | 0.007700098 | down |
| MELO3C009233.2 | 658 | 76 | -3.12 | 5.52708E-70 | down |
| MELO3C009236.2 | 17 | 0 | -6.729 | 2.64201E-06 | down |
| MELO3C009238.2 | 44 | 7 | -2.724 | 7.66258E-06 | down |
| MELO3C009241.2 | 664 | 294 | -1.176 | 7.01245E-16 | down |
| MELO3C009245.2 | 100 | 0 | -8.29 | 1.24899E-11 | down |
| MELO3C009246.2 | 16 | 368 | 4.524 | 1.68944E-50 | up |
| MELO3C009249.2 | 160 | 52 | -1.616 | 3.0351E-10 | down |
| MELO3C009250.2 | 97 | 48 | -1.001 | 0.000315523 | down |
| MELO3C009252.2 | 2673 | 381 | -2.809 | 5.24924E-93 | down |
| MELO3C009254.2 | 869 | 377 | -1.203 | 1.06346E-18 | down |
| MELO3C009262.2 | 21 | 109 | 2.392 | 6.87835E-11 | up |
| MELO3C009263.2 | 2 | 247 | 7.047 | 2.56668E-24 | up |
| MELO3C009266.2 | 7 | 72 | 3.421 | 2.11872E-10 | up |
| MELO3C009267.2 | 47 | 9 | -2.517 | 4.38378E-06 | down |
| MELO3C009271.2 | 0 | 5 | 4.535 | 0.019135187 | up |
| MELO3C009282.2 | 2649 | 71 | -5.237 | 7.55991E-251 | down |
| MELO3C009288.2 | 480 | 2383 | 2.31 | 1.55426E-29 | up |
| MELO3C009291.2 | 24 | 88 | 1.88 | 2.72211E-07 | up |
| MELO3C009292.2 | 43 | 142 | 1.708 | 0.000352101 | up |
| MELO3C009294.2 | 33 | 9 | -1.875 | 0.001045553 | down |
| MELO3C009296.2 | 60 | 147 | 1.286 | 1.26968E-05 | up |
| MELO3C009297.2 | 1706 | 3781 | 1.148 | 5.06257E-39 | up |
| MELO3C009299.2 | 98 | 360 | 1.883 | 2.28559E-26 | up |
| MELO3C009302.2 | 29 | 69 | 1.229 | 0.000321345 | up |
| MELO3C009307.2 | 26 | 4 | -2.917 | 4.76122E-05 | down |
| MELO3C009308.2 | 138 | 408 | 1.571 | 1.39896E-14 | up |
| MELO3C009313.2 | 19 | 188 | 3.382 | 4.74356E-23 | up |
| MELO3C009321.2 | 18 | 41 | 1.251 | 0.006131536 | up |
| MELO3C009325.2 | 60 | 123 | 1.052 | 0.002792157 | up |
| MELO3C009330.2 | 853 | 137 | -2.636 | 9.35467E-41 | down |
| MELO3C009333.2 | 149 | 1 | -7.973 | 6.3877E-14 | down |
| MELO3C009334.2 | 8345 | 24473 | 1.552 | 6.32731E-123 | up |
| MELO3C009342.2 | 35 | 164 | 2.243 | 2.10135E-15 | up |
| MELO3C009343.2 | 10151 | 4733 | -1.101 | 4.87545E-55 | down |
| MELO3C009355.2 | 45377 | 13074 | -1.795 | 5.64046E-46 | down |
| MELO3C009356.2 | 18 | 49 | 1.422 | 0.001617874 | up |
| MELO3C009361.2 | 820 | 365 | -1.168 | 4.69672E-17 | down |
| MELO3C009364.2 | 218 | 41 | -2.425 | 3.58877E-16 | down |
| MELO3C009369.2 | 3 | 22 | 2.557 | 0.00089266 | up |
| MELO3C009372.2 | 3162 | 198 | -4.004 | 2.71498E-183 | down |
| MELO3C009385.2 | 140 | 312 | 1.155 | 5.48315E-11 | up |
| MELO3C009386.2 | 336 | 106 | -1.66 | 8.45984E-16 | down |
| MELO3C009387.2 | 460 | 35 | -3.723 | 1.41628E-40 | down |
| MELO3C009388.2 | 11 | 29 | 1.438 | 0.022389492 | up |
| MELO3C009391.2 | 2388 | 397 | -2.587 | 2.39614E-67 | down |
| MELO3C009406.2 | 1 | 13 | 4.103 | 0.002388262 | up |
| MELO3C009412.2 | 1 | 21 | 4.803 | 0.000103136 | up |
| MELO3C009415.2 | 834 | 127 | -2.716 | 2.24519E-62 | down |
| MELO3C009429.2 | 2718 | 1288 | -1.078 | 7.01229E-23 | down |
| MELO3C009438.2 | 7577 | 20782 | 1.456 | 3.78121E-56 | up |
| MELO3C009440.2 | 1643 | 4089 | 1.315 | 3.70449E-34 | up |
| MELO3C009445.2 | 12174 | 4777 | -1.349 | 1.10903E-57 | down |
| MELO3C009452.2 | 1131 | 3378 | 1.579 | 2.11018E-07 | up |
| MELO3C009454.2 | 2359 | 558 | -2.077 | 5.51753E-59 | down |
| MELO3C009459.2 | 721 | 252 | -1.516 | 5.631E-32 | down |
| MELO3C009461.2 | 47 | 1 | -5.045 | 5.39805E-09 | down |
| MELO3C009470.2 | 33 | 4 | -3.234 | 1.04478E-05 | down |
| MELO3C009475.2 | 1446 | 478 | -1.596 | 7.06768E-47 | down |
| MELO3C009477.2 | 194 | 1026 | 2.404 | 4.18091E-71 | up |
| MELO3C009486.2 | 11 | 142 | 3.671 | 9.43366E-18 | up |
| MELO3C009495.2 | 23 | 0 | -7.16 | 3.48209E-07 | down |
| MELO3C009497.2 | 697 | 3249 | 2.22 | 2.41428E-64 | up |
| MELO3C009501.2 | 303 | 65 | -2.2 | 3.99967E-19 | down |
| MELO3C009503.2 | 592 | 140 | -2.089 | 2.18934E-32 | down |
| MELO3C009504.2 | 0 | 205 | 9.953 | 1.26297E-16 | up |
| MELO3C009505.2 | 1 | 134 | 6.495 | 4.12104E-16 | up |
| MELO3C009506.2 | 164 | 5 | -5.037 | 3.27352E-30 | down |
| MELO3C009507.2 | 83 | 4 | -4.431 | 1.03606E-15 | down |
| MELO3C009512.2 | 1201 | 206 | -2.544 | 1.00311E-34 | down |
| MELO3C009519.2 | 12888 | 4741 | -1.443 | 1.6133E-45 | down |
| MELO3C009523.2 | 190 | 52 | -1.861 | 0.000117683 | down |
| MELO3C009524.2 | 987 | 91 | -3.428 | 1.43058E-68 | down |
| MELO3C009530.2 | 82 | 2 | -4.98 | 5.07829E-10 | down |
| MELO3C009531.2 | 39 | 2 | -4.462 | 1.79161E-05 | down |
| MELO3C009533.2 | 8 | 30 | 1.796 | 0.006272544 | up |
| MELO3C009541.2 | 11 | 0 | -6.017 | 0.000122718 | down |
| MELO3C009543.2 | 16 | 3 | -2.563 | 0.015723295 | down |
| MELO3C009550.2 | 1935 | 180 | -3.429 | 2.70655E-163 | down |
| MELO3C009554.2 | 70 | 6 | -3.701 | 5.31837E-12 | down |
| MELO3C009555.2 | 141 | 34 | -2.064 | 2.54206E-12 | down |
| MELO3C009563.2 | 244 | 119 | -1.036 | 9.61386E-08 | down |
| MELO3C009564.2 | 87 | 430 | 2.309 | 9.01257E-33 | up |
| MELO3C009567.2 | 1034 | 7570 | 2.873 | 3.60996E-110 | up |
| MELO3C009569.2 | 691 | 72 | -3.254 | 1.00584E-57 | down |
| MELO3C009572.2 | 16581 | 7445 | -1.155 | 2.73895E-21 | down |
| MELO3C009574.2 | 302 | 866 | 1.52 | 3.03933E-29 | up |
| MELO3C009581.2 | 2917 | 789 | -1.886 | 1.85679E-125 | down |
| MELO3C009585.2 | 8 | 31 | 1.921 | 0.008743823 | up |
| MELO3C009589.2 | 1444 | 704 | -1.038 | 1.17279E-14 | down |
| MELO3C009595.2 | 4661 | 1800 | -1.373 | 1.95044E-50 | down |
| MELO3C009596.2 | 90 | 18 | -2.287 | 1.00061E-06 | down |
| MELO3C009597.2 | 780 | 2213 | 1.504 | 1.97301E-47 | up |
| MELO3C009602.2 | 156 | 455 | 1.542 | 1.02573E-20 | up |
| MELO3C009608.2 | 429 | 210 | -1.028 | 6.41149E-10 | down |
| MELO3C009610.2 | 26 | 7 | -1.977 | 0.001882747 | down |
| MELO3C009618.2 | 4 | 47 | 3.401 | 3.44112E-08 | up |
| MELO3C009628.2 | 2171 | 667 | -1.705 | 1.02165E-45 | down |
| MELO3C009630.2 | 12757 | 2230 | -2.515 | 5.40078E-56 | down |
| MELO3C009637.2 | 131 | 1 | -6.791 | 3.67662E-17 | down |
| MELO3C009641.2 | 41 | 8 | -2.426 | 0.00107139 | down |
| MELO3C009649.2 | 1 | 45 | 4.919 | 1.00504E-08 | up |
| MELO3C009664.2 | 109 | 275 | 1.334 | 2.1247E-11 | up |
| MELO3C009665.2 | 111 | 571 | 2.359 | 1.02201E-31 | up |
| MELO3C009674.2 | 7153 | 964 | -2.893 | 8.25358E-87 | down |
| MELO3C009678.2 | 1157 | 3063 | 1.404 | 1.47923E-52 | up |
| MELO3C009679.2 | 404 | 1015 | 1.327 | 9.13529E-10 | up |
| MELO3C009682.2 | 304 | 833 | 1.454 | 1.04323E-27 | up |
| MELO3C009683.2 | 2201 | 10014 | 2.185 | 2.86586E-06 | up |
| MELO3C009686.2 | 208 | 72 | -1.514 | 1.31561E-05 | down |
| MELO3C009706.2 | 414 | 70 | -2.566 | 3.01101E-34 | down |
| MELO3C009713.2 | 7331 | 3383 | -1.116 | 3.25222E-24 | down |
| MELO3C009719.2 | 463 | 81 | -2.514 | 1.47844E-39 | down |
| MELO3C009728.2 | 338 | 93 | -1.86 | 8.48002E-17 | down |
| MELO3C009733.2 | 110 | 15 | -2.884 | 4.41852E-15 | down |
| MELO3C009739.2 | 49 | 1 | -5.353 | 1.23344E-08 | down |
| MELO3C009741.2 | 2142 | 401 | -2.422 | 6.8627E-54 | down |
| MELO3C009755.2 | 1212 | 230 | -2.394 | 1.40303E-09 | down |
| MELO3C009758.2 | 255 | 79 | -1.699 | 5.21755E-09 | down |
| MELO3C009759.2 | 23615 | 6042 | -1.967 | 1.54451E-82 | down |
| MELO3C009768.2 | 302 | 59 | -2.364 | 7.46981E-24 | down |
| MELO3C009773.2 | 15 | 39 | 1.402 | 0.018269132 | up |
| MELO3C009782.2 | 4039 | 27732 | 2.78 | 2.08027E-108 | up |
| MELO3C009784.2 | 37 | 144 | 1.943 | 3.16098E-10 | up |
| MELO3C009788.2 | 41 | 20 | -1.011 | 0.021226661 | down |
| MELO3C009789.2 | 764 | 100 | -2.942 | 1.23679E-64 | down |
| MELO3C009790.2 | 1278 | 51 | -4.657 | 8.42239E-192 | down |
| MELO3C009794.2 | 2864 | 1164 | -1.299 | 3.91617E-36 | down |
| MELO3C009798.2 | 203 | 27 | -2.921 | 2.27369E-28 | down |
| MELO3C009803.2 | 885 | 427 | -1.053 | 8.33785E-17 | down |
| MELO3C009806.2 | 101 | 34 | -1.566 | 3.74723E-07 | down |
| MELO3C009810.2 | 172 | 619 | 1.851 | 1.6321E-22 | up |
| MELO3C009821.2 | 750 | 351 | -1.097 | 4.09099E-18 | down |
| MELO3C009833.2 | 16 | 119 | 2.879 | 1.27718E-14 | up |
| MELO3C009840.2 | 2 | 12 | 2.22 | 0.019833029 | up |
| MELO3C009846.2 | 3097 | 1158 | -1.42 | 1.12827E-45 | down |
| MELO3C009849.2 | 87 | 18 | -2.307 | 6.92218E-07 | down |
| MELO3C009855.2 | 74 | 24 | -1.599 | 4.01796E-05 | down |
| MELO3C009856.2 | 7 | 0 | -5.431 | 0.001857393 | down |
| MELO3C009858.2 | 0 | 5 | 4.579 | 0.016138771 | up |
| MELO3C009863.2 | 550 | 235 | -1.23 | 2.58626E-17 | down |
| MELO3C009864.2 | 8099 | 148 | -5.774 | 0 | down |
| MELO3C009869.2 | 118 | 402 | 1.776 | 1.41085E-16 | up |
| MELO3C009871.2 | 333 | 1121 | 1.751 | 3.69902E-25 | up |
| MELO3C009872.2 | 86 | 580 | 2.763 | 7.27967E-25 | up |
| MELO3C009873.2 | 453 | 66 | -2.773 | 7.9799E-36 | down |
| MELO3C009877.2 | 2823 | 639 | -2.144 | 2.31309E-75 | down |
| MELO3C009881.2 | 530 | 1 | -9.811 | 2.41388E-21 | down |
| MELO3C009882.2 | 561 | 194 | -1.535 | 1.41244E-29 | down |
| MELO3C009884.2 | 123 | 37 | -1.709 | 2.30279E-09 | down |
| MELO3C009886.2 | 2042 | 4150 | 1.023 | 3.94202E-29 | up |
| MELO3C009892.2 | 46 | 19 | -1.259 | 0.022154916 | down |
| MELO3C009900.2 | 29 | 7 | -1.98 | 0.027212812 | down |
| MELO3C009902.2 | 3 | 17 | 2.307 | 0.004958181 | up |
| MELO3C009911.2 | 1860 | 680 | -1.453 | 3.18453E-55 | down |
| MELO3C009927.2 | 3002 | 729 | -2.044 | 5.30613E-72 | down |
| MELO3C009930.2 | 886 | 425 | -1.061 | 8.56679E-09 | down |
| MELO3C009937.2 | 245 | 89 | -1.469 | 3.11654E-13 | down |
| MELO3C009940.2 | 350 | 93 | -1.92 | 8.3608E-20 | down |
| MELO3C009943.2 | 256 | 833 | 1.707 | 1.29418E-34 | up |
| MELO3C009944.2 | 591 | 1316 | 1.155 | 3.4068E-19 | up |
| MELO3C009952.2 | 1850 | 336 | -2.461 | 1.25918E-125 | down |
| MELO3C009955.2 | 2316 | 992 | -1.224 | 5.96199E-37 | down |
| MELO3C009961.2 | 8980 | 3666 | -1.293 | 3.83594E-41 | down |
| MELO3C009963.2 | 980 | 2305 | 1.234 | 3.21963E-30 | up |
| MELO3C009965.2 | 4408 | 1202 | -1.876 | 7.23076E-57 | down |
| MELO3C009966.2 | 542 | 1547 | 1.511 | 2.40362E-35 | up |
| MELO3C009968.2 | 1279 | 512 | -1.322 | 2.84431E-27 | down |
| MELO3C009970.2 | 13 | 57 | 2.144 | 1.7692E-05 | up |
| MELO3C009990.2 | 18 | 1 | -3.945 | 0.000576159 | down |
| MELO3C009996.2 | 10621 | 3472 | -1.613 | 7.46381E-81 | down |
| MELO3C010002.2 | 31 | 164 | 2.428 | 4.09143E-17 | up |
| MELO3C010008.2 | 2878 | 1011 | -1.511 | 2.60617E-53 | down |
| MELO3C010015.2 | 70 | 400 | 2.511 | 9.35186E-17 | up |
| MELO3C010045.2 | 2439 | 437 | -2.483 | 3.31013E-55 | down |
| MELO3C010050.2 | 1711 | 678 | -1.336 | 8.61016E-41 | down |
| MELO3C010053.2 | 1953 | 4409 | 1.175 | 2.60444E-43 | up |
| MELO3C010056.2 | 13 | 38 | 1.547 | 0.001740948 | up |
| MELO3C010086.2 | 2984 | 1374 | -1.12 | 7.71522E-31 | down |
| MELO3C010097.2 | 2731 | 1184 | -1.206 | 1.33957E-39 | down |
| MELO3C010108.2 | 2300 | 1101 | -1.063 | 5.04981E-23 | down |
| MELO3C010125.2 | 59 | 836 | 3.832 | 3.67861E-76 | up |
| MELO3C010132.2 | 2 | 128 | 5.827 | 6.86302E-17 | up |
| MELO3C010152.2 | 519 | 122 | -2.08 | 3.70939E-38 | down |
| MELO3C010155.2 | 0 | 115 | 9.129 | 6.19756E-14 | up |
| MELO3C010162.2 | 325 | 43 | -2.917 | 2.41538E-36 | down |
| MELO3C010164.2 | 6830 | 1476 | -2.21 | 2.08038E-141 | down |
| MELO3C010170.2 | 430 | 136 | -1.663 | 5.16189E-09 | down |
| MELO3C010172.2 | 2847 | 65 | -5.461 | 1.11752E-214 | down |
| MELO3C010177.2 | 31 | 0 | -7.573 | 1.86129E-08 | down |
| MELO3C010180.2 | 15688 | 7562 | -1.053 | 2.39352E-42 | down |
| MELO3C010181.2 | 574 | 218 | -1.399 | 3.54469E-11 | down |
| MELO3C010182.2 | 11 | 1 | -4.301 | 0.002150193 | down |
| MELO3C010183.2 | 7765 | 634 | -3.613 | 4.18716E-12 | down |
| MELO3C010184.2 | 1334 | 25 | -5.724 | 4.59997E-12 | down |
| MELO3C010185.2 | 94 | 38 | -1.292 | 0.027169584 | down |
| MELO3C010190.2 | 486 | 36 | -3.716 | 1.60147E-71 | down |
| MELO3C010193.2 | 8 | 64 | 3.024 | 5.71115E-10 | up |
| MELO3C010196.2 | 703 | 241 | -1.549 | 2.17819E-11 | down |
| MELO3C010197.2 | 7422 | 1486 | -2.32 | 1.57107E-44 | down |
| MELO3C010200.2 | 222 | 103 | -1.106 | 1.12717E-05 | down |
| MELO3C010202.2 | 1363 | 653 | -1.061 | 3.89581E-21 | down |
| MELO3C010210.2 | 1120 | 121 | -3.205 | 3.87861E-51 | down |
| MELO3C010212.2 | 6 | 0 | -5.121 | 0.008536556 | down |
| MELO3C010214.2 | 2286 | 1097 | -1.058 | 8.74022E-28 | down |
| MELO3C010216.2 | 1304 | 543 | -1.263 | 5.84916E-27 | down |
| MELO3C010233.2 | 54 | 846 | 3.977 | 1.48762E-66 | up |
| MELO3C010234.2 | 855 | 2111 | 1.304 | 4.75329E-17 | up |
| MELO3C010236.2 | 30 | 97 | 1.731 | 5.25416E-08 | up |
| MELO3C010244.2 | 4086 | 70 | -5.866 | 1.00818E-61 | down |
| MELO3C010250.2 | 85 | 34 | -1.296 | 0.000178852 | down |
| MELO3C010259.2 | 0 | 5 | 4.57 | 0.018642019 | up |
| MELO3C010261.2 | 567 | 2213 | 1.964 | 1.1196E-76 | up |
| MELO3C010264.2 | 109 | 246 | 1.18 | 4.58822E-09 | up |
| MELO3C010270.2 | 454 | 57 | -3 | 7.78718E-43 | down |
| MELO3C010272.2 | 2330 | 14 | -7.341 | 2.32908E-52 | down |
| MELO3C010275.2 | 19281 | 3074 | -2.649 | 8.26465E-282 | down |
| MELO3C010276.2 | 148 | 3 | -5.662 | 9.61965E-26 | down |
| MELO3C010277.2 | 2136 | 874 | -1.29 | 2.3389E-42 | down |
| MELO3C010280.2 | 17 | 37 | 1.099 | 0.020581532 | up |
| MELO3C010293.2 | 42 | 112 | 1.401 | 1.5031E-06 | up |
| MELO3C010301.2 | 1014 | 2618 | 1.367 | 1.47848E-18 | up |
| MELO3C010308.2 | 2 | 21 | 3.797 | 0.000310583 | up |
| MELO3C010314.2 | 5511 | 155 | -5.142 | 3.68073E-206 | down |
| MELO3C010316.2 | 757 | 1853 | 1.293 | 1.10826E-20 | up |
| MELO3C010317.2 | 1165 | 141 | -3.04 | 2.48774E-44 | down |
| MELO3C010323.2 | 682 | 1367 | 1.001 | 7.62674E-09 | up |
| MELO3C010332.2 | 60 | 4 | -4.077 | 1.2379E-10 | down |
| MELO3C010338.2 | 762 | 367 | -1.054 | 1.26985E-05 | down |
| MELO3C010341.2 | 85 | 13 | -2.74 | 6.3288E-11 | down |
| MELO3C010353.2 | 5916 | 457 | -3.693 | 1.4321E-11 | down |
| MELO3C010358.2 | 1501 | 3011 | 1.004 | 2.92488E-19 | up |
| MELO3C010370.2 | 537 | 1343 | 1.323 | 9.39535E-32 | up |
| MELO3C010388.2 | 20 | 535 | 4.727 | 2.18339E-74 | up |
| MELO3C010391.2 | 4 | 99 | 4.457 | 2.65769E-15 | up |
| MELO3C010403.2 | 403 | 1857 | 2.206 | 1.37033E-39 | up |
| MELO3C010405.2 | 21 | 0 | -7.016 | 4.58785E-07 | down |
| MELO3C010409.2 | 359 | 81 | -2.155 | 2.37319E-11 | down |
| MELO3C010417.2 | 24 | 396 | 4.016 | 1.31005E-28 | up |
| MELO3C010419.2 | 222 | 738 | 1.734 | 9.21081E-22 | up |
| MELO3C010420.2 | 697 | 1926 | 1.465 | 9.91964E-44 | up |
| MELO3C010423.2 | 190 | 416 | 1.128 | 9.53614E-08 | up |
| MELO3C010425.2 | 285 | 137 | -1.054 | 1.16901E-06 | down |
| MELO3C010434.2 | 893 | 227 | -1.974 | 1.20169E-25 | down |
| MELO3C010452.2 | 192 | 36 | -2.416 | 3.32837E-19 | down |
| MELO3C010457.2 | 2 | 26 | 4.054 | 0.000108482 | up |
| MELO3C010458.2 | 67 | 5 | -3.654 | 3.94626E-13 | down |
| MELO3C010463.2 | 18 | 47 | 1.369 | 0.002196194 | up |
| MELO3C010470.2 | 1129 | 485 | -1.218 | 1.66373E-20 | down |
| MELO3C010471.2 | 528 | 153 | -1.783 | 0.010829 | down |
| MELO3C010473.2 | 24 | 161 | 2.746 | 1.6491E-14 | up |
| MELO3C010474.2 | 878 | 2273 | 1.373 | 2.53356E-40 | up |
| MELO3C010476.2 | 409 | 1102 | 1.429 | 5.50568E-30 | up |
| MELO3C010491.2 | 44 | 19 | -1.224 | 0.006064891 | down |
| MELO3C010492.2 | 292 | 1434 | 2.295 | 3.11494E-61 | up |
| MELO3C010497.2 | 1 | 9 | 2.639 | 0.02796417 | up |
| MELO3C010500.2 | 1580 | 276 | -2.514 | 1.31092E-104 | down |
| MELO3C010506.2 | 848 | 37 | -4.5 | 2.46938E-101 | down |
| MELO3C010512.2 | 1538 | 341 | -2.172 | 8.65587E-103 | down |
| MELO3C010520.2 | 4 | 33 | 2.888 | 4.12414E-05 | up |
| MELO3C010528.2 | 1 | 69 | 5.527 | 1.14504E-11 | up |
| MELO3C010532.2 | 1833 | 827 | -1.149 | 1.21892E-14 | down |
| MELO3C010540.2 | 81 | 242 | 1.578 | 2.30281E-08 | up |
| MELO3C010548.2 | 782 | 3262 | 2.061 | 2.02095E-127 | up |
| MELO3C010552.2 | 8739 | 4017 | -1.121 | 2.13604E-28 | down |
| MELO3C010561.2 | 144 | 47 | -1.634 | 4.50398E-10 | down |
| MELO3C010588.2 | 62 | 13 | -2.301 | 3.73141E-05 | down |
| MELO3C010598.2 | 605 | 133 | -2.183 | 2.82053E-26 | down |
| MELO3C010604.2 | 24 | 277 | 3.559 | 7.66818E-28 | up |
| MELO3C010605.2 | 81 | 23 | -1.839 | 2.3678E-06 | down |
| MELO3C010608.2 | 16663 | 6359 | -1.39 | 2.14797E-19 | down |
| MELO3C010611.2 | 1046 | 477 | -1.133 | 4.06712E-17 | down |
| MELO3C010614.2 | 9831 | 987 | -3.316 | 3.55938E-171 | down |
| MELO3C010617.2 | 19 | 2 | -3.434 | 0.000280458 | down |
| MELO3C010621.2 | 22 | 152 | 2.785 | 1.15934E-20 | up |
| MELO3C010624.2 | 341 | 3842 | 3.497 | 2.56302E-175 | up |
| MELO3C010632.2 | 831 | 153 | -2.438 | 3.70255E-49 | down |
| MELO3C010636.2 | 135 | 658 | 2.281 | 3.81942E-55 | up |
| MELO3C010639.2 | 60 | 124 | 1.055 | 0.010412969 | up |
| MELO3C010646.2 | 15 | 36 | 1.28 | 0.007576633 | up |
| MELO3C010660.2 | 395 | 1096 | 1.471 | 8.71544E-38 | up |
| MELO3C010668.2 | 294 | 144 | -1.026 | 0.000579098 | down |
| MELO3C010675.2 | 7435 | 19213 | 1.37 | 1.59037E-37 | up |
| MELO3C010677.2 | 35 | 76 | 1.132 | 0.001129201 | up |
| MELO3C010682.2 | 1 | 10 | 3.695 | 0.011001148 | up |
| MELO3C010689.2 | 535 | 249 | -1.102 | 1.75316E-15 | down |
| MELO3C010691.2 | 168 | 24 | -2.797 | 2.93971E-19 | down |
| MELO3C010697.2 | 696 | 306 | -1.184 | 2.94797E-19 | down |
| MELO3C010698.2 | 3860 | 1576 | -1.292 | 1.97233E-28 | down |
| MELO3C010700.2 | 20 | 2 | -3.598 | 0.000467032 | down |
| MELO3C010703.2 | 46 | 12 | -1.949 | 8.04775E-05 | down |
| MELO3C010706.2 | 2 | 19 | 3.634 | 0.001680943 | up |
| MELO3C010707.2 | 5252 | 2540 | -1.049 | 5.29829E-35 | down |
| MELO3C010708.2 | 4511 | 20 | -7.853 | 8.67051E-278 | down |
| MELO3C010711.2 | 1086 | 450 | -1.272 | 3.00725E-17 | down |
| MELO3C010716.2 | 7227 | 1207 | -2.584 | 1.73888E-135 | down |
| MELO3C010725.2 | 1 | 49 | 6.031 | 8.60579E-08 | up |
| MELO3C010726.2 | 105 | 297 | 1.509 | 6.93364E-11 | up |
| MELO3C010737.2 | 806 | 149 | -2.432 | 9.21129E-44 | down |
| MELO3C010738.2 | 4903 | 923 | -2.41 | 3.69508E-100 | down |
| MELO3C010743.2 | 19 | 71 | 1.924 | 2.12871E-06 | up |
| MELO3C010748.2 | 1029 | 338 | -1.603 | 7.06187E-35 | down |
| MELO3C010752.2 | 29 | 10 | -1.478 | 0.009304906 | down |
| MELO3C010755.2 | 233 | 94 | -1.307 | 1.47964E-11 | down |
| MELO3C010759.2 | 0 | 11 | 4.816 | 0.001375764 | up |
| MELO3C010760.2 | 129 | 2460 | 4.248 | 1.05918E-269 | up |
| MELO3C010763.2 | 55049 | 16704 | -1.721 | 1.46377E-66 | down |
| MELO3C010773.2 | 29 | 4 | -2.821 | 2.53139E-05 | down |
| MELO3C010774.2 | 1132 | 154 | -2.87 | 5.94041E-63 | down |
| MELO3C010776.2 | 16664 | 228 | -6.193 | 0 | down |
| MELO3C010777.2 | 26 | 3 | -3.374 | 0.001174708 | down |
| MELO3C010779.2 | 1120 | 68 | -4.045 | 7.34055E-70 | down |
| MELO3C010782.2 | 41 | 670 | 4.062 | 1.02171E-74 | up |
| MELO3C010784.2 | 8 | 41 | 2.291 | 0.001263723 | up |
| MELO3C010785.2 | 3389 | 1635 | -1.052 | 5.3375E-21 | down |
| MELO3C010786.2 | 168 | 360 | 1.098 | 2.93941E-08 | up |
| MELO3C010787.2 | 28 | 1 | -4.569 | 1.32245E-06 | down |
| MELO3C010788.2 | 14 | 3 | -1.95 | 0.027532389 | down |
| MELO3C010796.2 | 4 | 628 | 7.392 | 1.67555E-48 | up |
| MELO3C010797.2 | 579 | 1207 | 1.061 | 2.17198E-17 | up |
| MELO3C010809.2 | 771 | 382 | -1.014 | 2.94078E-14 | down |
| MELO3C010810.2 | 6945 | 2472 | -1.49 | 2.24019E-70 | down |
| MELO3C010812.2 | 3062 | 6473 | 1.08 | 3.58827E-29 | up |
| MELO3C010814.2 | 122 | 59 | -1.048 | 2.15655E-05 | down |
| MELO3C010822.2 | 1 | 23 | 3.895 | 7.07922E-05 | up |
| MELO3C010825.2 | 70 | 3 | -4.582 | 2.50421E-14 | down |
| MELO3C010826.2 | 23 | 1 | -4.275 | 2.64755E-05 | down |
| MELO3C010829.2 | 13 | 2 | -2.871 | 0.008830441 | down |
| MELO3C010830.2 | 37 | 13 | -1.497 | 0.004378924 | down |
| MELO3C010833.2 | 3806 | 1333 | -1.514 | 7.09345E-54 | down |
| MELO3C010840.2 | 829 | 27 | -4.964 | 1.73949E-134 | down |
| MELO3C010843.2 | 41 | 20 | -1.033 | 0.015658942 | down |
| MELO3C010846.2 | 160 | 743 | 2.219 | 1.84931E-33 | up |
| MELO3C010858.2 | 312 | 24 | -3.701 | 8.65815E-45 | down |
| MELO3C010859.2 | 165 | 70 | -1.238 | 9.90956E-08 | down |
| MELO3C010861.2 | 706 | 49 | -3.855 | 7.85291E-83 | down |
| MELO3C010863.2 | 731 | 233 | -1.649 | 5.33581E-26 | down |
| MELO3C010865.2 | 12 | 2 | -2.378 | 0.013808157 | down |
| MELO3C010867.2 | 15 | 36 | 1.319 | 0.007507241 | up |
| MELO3C010868.2 | 11 | 83 | 2.891 | 3.12951E-12 | up |
| MELO3C010869.2 | 1750 | 25 | -6.126 | 8.27584E-139 | down |
| MELO3C010870.2 | 33 | 121 | 1.856 | 9.73044E-07 | up |
| MELO3C010872.2 | 5508 | 2225 | -1.307 | 7.51253E-05 | down |
| MELO3C010875.2 | 3179 | 1012 | -1.651 | 4.78107E-18 | down |
| MELO3C010882.2 | 16 | 309 | 4.261 | 1.05569E-39 | up |
| MELO3C010893.2 | 16 | 57 | 1.902 | 0.000198436 | up |
| MELO3C010894.2 | 142 | 357 | 1.327 | 7.09471E-12 | up |
| MELO3C010900.2 | 1 | 9 | 3.613 | 0.014870912 | up |
| MELO3C010902.2 | 15 | 49 | 1.694 | 0.000360661 | up |
| MELO3C010909.2 | 14 | 128 | 3.175 | 3.60294E-18 | up |
| MELO3C010912.2 | 532 | 1125 | 1.078 | 1.60189E-23 | up |
| MELO3C010918.2 | 584 | 251 | -1.22 | 2.00639E-16 | down |
| MELO3C010922.2 | 332 | 1212 | 1.87 | 2.48533E-64 | up |
| MELO3C010925.2 | 216 | 525 | 1.288 | 3.94944E-11 | up |
| MELO3C010926.2 | 221 | 64 | -1.795 | 1.25244E-10 | down |
| MELO3C010933.2 | 510 | 1778 | 1.802 | 1.42239E-53 | up |
| MELO3C010935.2 | 157 | 424 | 1.431 | 2.63002E-14 | up |
| MELO3C010938.2 | 9349 | 4623 | -1.016 | 1.32757E-27 | down |
| MELO3C010951.2 | 37 | 339 | 3.217 | 9.05209E-30 | up |
| MELO3C010954.2 | 35 | 3 | -3.425 | 5.38799E-06 | down |
| MELO3C010963.2 | 300 | 61 | -2.314 | 1.19045E-29 | down |
| MELO3C010965.2 | 22 | 92 | 2.061 | 3.47144E-07 | up |
| MELO3C010967.2 | 1376 | 3098 | 1.171 | 3.34564E-17 | up |
| MELO3C010969.2 | 662 | 1623 | 1.292 | 3.33749E-14 | up |
| MELO3C010973.2 | 2815 | 1022 | -1.461 | 1.64614E-71 | down |
| MELO3C010974.2 | 114 | 699 | 2.61 | 6.98618E-39 | up |
| MELO3C010975.2 | 4136 | 8621 | 1.059 | 8.07151E-32 | up |
| MELO3C010976.2 | 4368 | 1796 | -1.282 | 4.7205E-72 | down |
| MELO3C010978.2 | 4 | 35 | 3.361 | 4.09898E-05 | up |
| MELO3C010988.2 | 70 | 277 | 1.986 | 2.74262E-14 | up |
| MELO3C010989.2 | 1144 | 556 | -1.04 | 1.11182E-12 | down |
| MELO3C011001.2 | 259 | 660 | 1.347 | 2.33414E-25 | up |
| MELO3C011008.2 | 1412 | 54 | -4.708 | 4.66053E-99 | down |
| MELO3C011016.2 | 133 | 1443 | 3.44 | 1.05393E-58 | up |
| MELO3C011017.2 | 1 | 14 | 4.19 | 0.002754137 | up |
| MELO3C011020.2 | 102 | 427 | 2.066 | 1.04179E-32 | up |
| MELO3C011029.2 | 179 | 558 | 1.644 | 4.04288E-21 | up |
| MELO3C011033.2 | 44 | 1 | -6.237 | 5.23976E-08 | down |
| MELO3C011034.2 | 13 | 312 | 4.618 | 8.54815E-42 | up |
| MELO3C011037.2 | 1720 | 12652 | 2.879 | 2.43172E-26 | up |
| MELO3C011041.2 | 866 | 2527 | 1.547 | 7.99249E-34 | up |
| MELO3C011046.2 | 1963 | 4152 | 1.081 | 8.22018E-19 | up |
| MELO3C011051.2 | 57 | 279 | 2.288 | 1.32461E-17 | up |
| MELO3C011053.2 | 56 | 22 | -1.381 | 0.000518952 | down |
| MELO3C011055.2 | 69 | 7 | -3.296 | 7.95937E-11 | down |
| MELO3C011056.2 | 178 | 83 | -1.099 | 0.000844408 | down |
| MELO3C011062.2 | 457 | 1216 | 1.412 | 2.13192E-27 | up |
| MELO3C011064.2 | 21 | 1 | -5.205 | 4.32092E-05 | down |
| MELO3C011066.2 | 69 | 18 | -1.968 | 3.43076E-07 | down |
| MELO3C011078.2 | 559 | 270 | -1.053 | 3.88334E-13 | down |
| MELO3C011080.2 | 4 | 23 | 2.327 | 0.000873068 | up |
| MELO3C011089.2 | 988 | 2023 | 1.032 | 2.63653E-27 | up |
| MELO3C011093.2 | 6 | 0 | -5.178 | 0.005954948 | down |
| MELO3C011100.2 | 3 | 24 | 3.004 | 0.000342273 | up |
| MELO3C011106.2 | 868 | 346 | -1.328 | 5.25423E-27 | down |
| MELO3C011113.2 | 9501 | 897 | -3.405 | 1.44952E-230 | down |
| MELO3C011114.2 | 2962 | 381 | -2.954 | 3.93478E-49 | down |
| MELO3C011117.2 | 302 | 992 | 1.716 | 3.16602E-19 | up |
| MELO3C011120.2 | 1 | 10 | 3.76 | 0.007863812 | up |
| MELO3C011123.2 | 21 | 8 | -1.407 | 0.024646402 | down |
| MELO3C011125.2 | 42 | 6 | -2.713 | 8.56103E-07 | down |
| MELO3C011129.2 | 7972 | 513 | -3.957 | 3.04952E-281 | down |
| MELO3C011146.2 | 61 | 162 | 1.419 | 1.51432E-07 | up |
| MELO3C011148.2 | 17 | 0 | -6.722 | 2.16748E-06 | down |
| MELO3C011159.2 | 203 | 628 | 1.626 | 1.80601E-24 | up |
| MELO3C011160.2 | 312 | 986 | 1.66 | 2.86657E-44 | up |
| MELO3C011169.2 | 305 | 790 | 1.373 | 1.1032E-12 | up |
| MELO3C011174.2 | 16 | 4 | -2.253 | 0.008898394 | down |
| MELO3C011177.2 | 2433 | 1009 | -1.27 | 1.11315E-13 | down |
| MELO3C011178.2 | 414 | 128 | -1.705 | 7.9504E-17 | down |
| MELO3C011179.2 | 594 | 2126 | 1.842 | 5.22136E-62 | up |
| MELO3C011180.2 | 30 | 191 | 2.671 | 4.05298E-22 | up |
| MELO3C011181.2 | 541 | 1386 | 1.36 | 1.88182E-30 | up |
| MELO3C011187.2 | 496 | 108 | -2.203 | 4.15885E-44 | down |
| MELO3C011192.2 | 1741 | 327 | -2.41 | 5.4576E-43 | down |
| MELO3C011196.2 | 17177 | 7060 | -1.283 | 5.3572E-52 | down |
| MELO3C011201.2 | 3158 | 1357 | -1.22 | 3.01126E-30 | down |
| MELO3C011207.2 | 84 | 173 | 1.035 | 7.01835E-06 | up |
| MELO3C011209.2 | 198 | 443 | 1.165 | 2.28915E-14 | up |
| MELO3C011210.2 | 27 | 8 | -1.832 | 0.003580966 | down |
| MELO3C011214.2 | 1587 | 724 | -1.133 | 9.84425E-29 | down |
| MELO3C011215.2 | 23 | 4 | -2.375 | 0.002663093 | down |
| MELO3C011217.2 | 936 | 1914 | 1.032 | 7.7237E-17 | up |
| MELO3C011218.2 | 0 | 7 | 5.062 | 0.005087803 | up |
| MELO3C011221.2 | 1 | 45 | 4.942 | 4.0292E-08 | up |
| MELO3C011224.2 | 6 | 37 | 2.534 | 3.83633E-05 | up |
| MELO3C011227.2 | 0 | 76 | 8.533 | 5.6004E-12 | up |
| MELO3C011237.2 | 390 | 983 | 1.333 | 1.08074E-24 | up |
| MELO3C011238.2 | 59 | 20 | -1.558 | 0.000702933 | down |
| MELO3C011240.2 | 88 | 1 | -6.213 | 1.89402E-14 | down |
| MELO3C011241.2 | 15 | 3 | -2.314 | 0.010914173 | down |
| MELO3C011242.2 | 677 | 40 | -4.082 | 1.12386E-62 | down |
| MELO3C011254.2 | 43 | 1 | -5.218 | 1.61579E-08 | down |
| MELO3C011257.2 | 800 | 1881 | 1.234 | 1.12807E-46 | up |
| MELO3C011269.2 | 9849 | 2721 | -1.856 | 2.17455E-59 | down |
| MELO3C011270.2 | 165 | 76 | -1.113 | 0.000655191 | down |
| MELO3C011271.2 | 2926 | 6481 | 1.148 | 1.14595E-45 | up |
| MELO3C011275.2 | 84 | 37 | -1.198 | 0.000128379 | down |
| MELO3C011276.2 | 2202 | 4904 | 1.155 | 1.18021E-28 | up |
| MELO3C011283.2 | 152 | 1868 | 3.624 | 3.81287E-121 | up |
| MELO3C011289.2 | 108 | 35 | -1.621 | 1.71024E-08 | down |
| MELO3C011290.2 | 38 | 14 | -1.481 | 0.007378435 | down |
| MELO3C011293.2 | 21 | 142 | 2.78 | 2.51273E-08 | up |
| MELO3C011295.2 | 14 | 1 | -4.62 | 0.000816733 | down |
| MELO3C011296.2 | 61 | 27 | -1.142 | 0.008412249 | down |
| MELO3C011298.2 | 6 | 28 | 2.217 | 0.000878646 | up |
| MELO3C011309.2 | 291 | 71 | -2.029 | 5.45874E-18 | down |
| MELO3C011313.2 | 327 | 670 | 1.035 | 6.26143E-17 | up |
| MELO3C011317.2 | 9885 | 2600 | -1.927 | 1.09491E-168 | down |
| MELO3C011329.2 | 9 | 435 | 5.594 | 1.81616E-05 | up |
| MELO3C011338.2 | 123 | 267 | 1.116 | 9.68166E-09 | up |
| MELO3C011339.2 | 380 | 774 | 1.028 | 1.74846E-19 | up |
| MELO3C011340.2 | 15431 | 6821 | -1.178 | 9.30279E-49 | down |
| MELO3C011357.2 | 1349 | 2832 | 1.069 | 1.2257E-40 | up |
| MELO3C011359.2 | 74 | 200 | 1.435 | 3.68756E-08 | up |
| MELO3C011366.2 | 503 | 5777 | 3.521 | 1.06689E-09 | up |
| MELO3C011368.2 | 1600 | 8885 | 2.474 | 4.79647E-171 | up |
| MELO3C011373.2 | 1327 | 373 | -1.833 | 1.53047E-49 | down |
| MELO3C011387.2 | 76 | 208 | 1.456 | 4.131E-10 | up |
| MELO3C011389.2 | 1673 | 281 | -2.574 | 4.69185E-12 | down |
| MELO3C011392.2 | 1587 | 495 | -1.68 | 2.6756E-29 | down |
| MELO3C011393.2 | 285 | 117 | -1.276 | 7.19117E-13 | down |
| MELO3C011402.2 | 1356 | 3187 | 1.233 | 1.64318E-37 | up |
| MELO3C011405.2 | 14 | 132 | 3.151 | 6.08843E-16 | up |
| MELO3C011413.2 | 645 | 1730 | 1.422 | 2.68813E-11 | up |
| MELO3C011414.2 | 719 | 1441 | 1.003 | 5.21551E-18 | up |
| MELO3C011424.2 | 2454 | 4927 | 1.005 | 1.78397E-09 | up |
| MELO3C011430.2 | 69 | 175 | 1.334 | 1.24421E-09 | up |
| MELO3C011435.2 | 24653 | 3835 | -2.685 | 3.56616E-18 | down |
| MELO3C011439.2 | 2273 | 5866 | 1.367 | 7.049E-25 | up |
| MELO3C011441.2 | 121 | 411 | 1.764 | 9.43945E-22 | up |
| MELO3C011443.2 | 33 | 271 | 3.022 | 2.3596E-26 | up |
| MELO3C011450.2 | 852 | 254 | -1.745 | 5.51448E-51 | down |
| MELO3C011460.2 | 169 | 7 | -4.701 | 1.29129E-29 | down |
| MELO3C011461.2 | 101 | 14 | -2.873 | 2.9144E-12 | down |
| MELO3C011466.2 | 18 | 0 | -6.811 | 1.36784E-06 | down |
| MELO3C011467.2 | 2 | 11 | 2.245 | 0.028830129 | up |
| MELO3C011474.2 | 40 | 607 | 3.915 | 2.72033E-11 | up |
| MELO3C011475.2 | 2 | 254 | 7.08 | 1.13628E-21 | up |
| MELO3C011478.2 | 927 | 124 | -2.91 | 1.69687E-71 | down |
| MELO3C011483.2 | 1486 | 664 | -1.163 | 6.35667E-29 | down |
| MELO3C011484.2 | 407 | 157 | -1.372 | 2.11617E-08 | down |
| MELO3C011491.2 | 428 | 15 | -4.831 | 3.97709E-55 | down |
| MELO3C011493.2 | 37 | 106 | 1.528 | 3.7085E-05 | up |
| MELO3C011504.2 | 468 | 166 | -1.497 | 5.30002E-17 | down |
| MELO3C011512.2 | 2264 | 6430 | 1.507 | 5.4607E-74 | up |
| MELO3C011535.2 | 587 | 107 | -2.45 | 3.50591E-50 | down |
| MELO3C011536.2 | 7485 | 2890 | -1.373 | 1.41218E-88 | down |
| MELO3C011541.2 | 20 | 258 | 3.705 | 1.57475E-30 | up |
| MELO3C011546.2 | 39 | 216 | 2.47 | 5.29103E-16 | up |
| MELO3C011550.2 | 237 | 46 | -2.382 | 2.33358E-25 | down |
| MELO3C011553.2 | 387 | 138 | -1.49 | 7.92537E-19 | down |
| MELO3C011565.2 | 1981 | 972 | -1.028 | 5.46312E-12 | down |
| MELO3C011570.2 | 287 | 1154 | 2.006 | 1.99877E-42 | up |
| MELO3C011572.2 | 801 | 1722 | 1.104 | 4.76711E-28 | up |
| MELO3C011576.2 | 8587 | 22 | -8.564 | 0 | down |
| MELO3C011588.2 | 54 | 15 | -1.818 | 0.000198579 | down |
| MELO3C011605.2 | 1022 | 465 | -1.134 | 1.26973E-22 | down |
| MELO3C011610.2 | 19 | 295 | 3.933 | 7.73261E-07 | up |
| MELO3C011632.2 | 1660 | 682 | -1.282 | 3.46053E-41 | down |
| MELO3C011657.2 | 2834 | 58 | -5.631 | 1.39899E-238 | down |
| MELO3C011674.2 | 1 | 15 | 4.39 | 0.001106107 | up |
| MELO3C011698.2 | 0 | 6 | 4.879 | 0.00901702 | up |
| MELO3C011705.2 | 4005 | 485 | -3.046 | 8.78374E-21 | down |
| MELO3C011706.2 | 6 | 108 | 4.257 | 2.34955E-16 | up |
| MELO3C011714.2 | 1565 | 680 | -1.204 | 8.08276E-32 | down |
| MELO3C011720.2 | 3106 | 633 | -2.295 | 1.05276E-149 | down |
| MELO3C011724.2 | 4 | 0 | -4.506 | 0.0318744 | down |
| MELO3C011726.2 | 1846 | 844 | -1.129 | 1.49873E-39 | down |
| MELO3C011730.2 | 7 | 1 | -3.641 | 0.029700332 | down |
| MELO3C011734.2 | 9 | 2 | -2.544 | 0.02522607 | down |
| MELO3C011749.2 | 840 | 345 | -1.286 | 4.76222E-18 | down |
| MELO3C011761.2 | 36 | 896 | 4.658 | 1.82318E-103 | up |
| MELO3C011770.2 | 347 | 151 | -1.197 | 6.62354E-06 | down |
| MELO3C011771.2 | 23 | 742 | 5.049 | 5.75046E-97 | up |
| MELO3C011786.2 | 421 | 202 | -1.062 | 3.723E-10 | down |
| MELO3C011800.2 | 48 | 141 | 1.54 | 1.87734E-09 | up |
| MELO3C011807.2 | 34 | 8 | -2.107 | 0.000120487 | down |
| MELO3C011809.2 | 495 | 2171 | 2.135 | 7.01608E-45 | up |
| MELO3C011810.2 | 23 | 100 | 2.121 | 6.47774E-07 | up |
| MELO3C011812.2 | 6435 | 2892 | -1.154 | 4.14222E-30 | down |
| MELO3C011815.2 | 2 | 19 | 3.08 | 0.001111381 | up |
| MELO3C011825.2 | 2951 | 1384 | -1.093 | 4.70544E-21 | down |
| MELO3C011828.2 | 46 | 21 | -1.127 | 0.007642174 | down |
| MELO3C011846.2 | 340 | 142 | -1.257 | 6.53823E-15 | down |
| MELO3C011848.2 | 1129 | 9 | -6.999 | 3.13888E-126 | down |
| MELO3C011849.2 | 1111 | 141 | -2.974 | 1.44577E-45 | down |
| MELO3C011850.2 | 9 | 2 | -2.639 | 0.021014818 | down |
| MELO3C011852.2 | 2940 | 7249 | 1.302 | 4.02856E-39 | up |
| MELO3C011855.2 | 90 | 269 | 1.583 | 1.76459E-16 | up |
| MELO3C011861.2 | 256 | 2033 | 2.988 | 1.64083E-133 | up |
| MELO3C011862.2 | 1212 | 498 | -1.283 | 3.18978E-31 | down |
| MELO3C011864.2 | 2861 | 751 | -1.932 | 5.46628E-70 | down |
| MELO3C011865.2 | 39316 | 9 | -12.132 | 1.62086E-20 | down |
| MELO3C011868.2 | 12003 | 4235 | -1.503 | 5.36343E-32 | down |
| MELO3C011870.2 | 4 | 24 | 2.4 | 0.001541969 | up |
| MELO3C011872.2 | 9658 | 2174 | -2.152 | 7.05113E-78 | down |
| MELO3C011879.2 | 203 | 77 | -1.386 | 2.46988E-08 | down |
| MELO3C011883.2 | 586 | 92 | -2.663 | 1.24107E-50 | down |
| MELO3C011885.2 | 1979 | 579 | -1.774 | 3.33322E-42 | down |
| MELO3C011889.2 | 28 | 344 | 3.654 | 5.42286E-26 | up |
| MELO3C011894.2 | 57 | 9 | -2.73 | 6.55271E-08 | down |
| MELO3C011905.2 | 2741 | 1165 | -1.235 | 2.75719E-45 | down |
| MELO3C011907.2 | 69 | 142 | 1.045 | 0.001709161 | up |
| MELO3C011908.2 | 4644 | 919 | -2.337 | 1.28773E-72 | down |
| MELO3C011909.2 | 107 | 443 | 2.05 | 2.46091E-33 | up |
| MELO3C011911.2 | 18821 | 856 | -4.46 | 0 | down |
| MELO3C011912.2 | 15090 | 5509 | -1.454 | 2.84823E-43 | down |
| MELO3C011916.2 | 378 | 875 | 1.209 | 1.25315E-25 | up |
| MELO3C011921.2 | 303 | 133 | -1.193 | 0.000685593 | down |
| MELO3C011925.2 | 233 | 623 | 1.423 | 2.79431E-10 | up |
| MELO3C011928.2 | 9309 | 2448 | -1.927 | 1.17547E-37 | down |
| MELO3C011929.2 | 2475 | 27 | -6.533 | 3.33572E-246 | down |
| MELO3C011930.2 | 169 | 1365 | 3.019 | 8.66535E-41 | up |
| MELO3C011931.2 | 227 | 0 | -10.445 | 3.26233E-18 | down |
| MELO3C011939.2 | 674 | 202 | -1.738 | 2.51995E-20 | down |
| MELO3C011942.2 | 129 | 46 | -1.466 | 5.17992E-06 | down |
| MELO3C011943.2 | 72 | 5 | -3.946 | 6.34314E-12 | down |
| MELO3C011944.2 | 810 | 396 | -1.032 | 1.34728E-18 | down |
| MELO3C011945.2 | 748 | 305 | -1.295 | 2.31656E-14 | down |
| MELO3C011948.2 | 20 | 119 | 2.585 | 6.89548E-11 | up |
| MELO3C011949.2 | 5637 | 2235 | -1.335 | 1.63318E-53 | down |
| MELO3C011950.2 | 3351 | 889 | -1.914 | 1.35276E-38 | down |
| MELO3C011957.2 | 1167 | 2570 | 1.139 | 2.77309E-15 | up |
| MELO3C011959.2 | 328 | 138 | -1.254 | 9.87129E-12 | down |
| MELO3C011965.2 | 2 | 27 | 3.6 | 9.32291E-06 | up |
| MELO3C011968.2 | 1342 | 141 | -3.251 | 1.34228E-101 | down |
| MELO3C011970.2 | 388 | 1319 | 1.765 | 2.7216E-21 | up |
| MELO3C011978.2 | 613 | 147 | -2.058 | 6.35775E-29 | down |
| MELO3C011979.2 | 1684 | 261 | -2.686 | 1.24134E-58 | down |
| MELO3C011980.2 | 2906 | 0 | -14.121 | 7.15485E-33 | down |
| MELO3C011982.2 | 2830 | 917 | -1.626 | 2.48105E-66 | down |
| MELO3C011984.2 | 1557 | 402 | -1.952 | 3.39624E-77 | down |
| MELO3C011985.2 | 24 | 4 | -2.506 | 0.000312154 | down |
| MELO3C011986.2 | 2717 | 439 | -2.629 | 2.849E-109 | down |
| MELO3C011987.2 | 14 | 69 | 2.317 | 2.41082E-06 | up |
| MELO3C011988.2 | 1136 | 15 | -6.26 | 5.33256E-118 | down |
| MELO3C011993.2 | 45 | 10 | -2.089 | 0.000549896 | down |
| MELO3C011995.2 | 94 | 22 | -2.084 | 9.82371E-09 | down |
| MELO3C011998.2 | 3311 | 1332 | -1.313 | 1.45081E-36 | down |
| MELO3C012002.2 | 387 | 74 | -2.382 | 1.28512E-27 | down |
| MELO3C012004.2 | 138 | 394 | 1.514 | 3.00313E-19 | up |
| MELO3C012009.2 | 220 | 470 | 1.096 | 2.97868E-10 | up |
| MELO3C012010.2 | 4364 | 1550 | -1.494 | 1.19901E-61 | down |
| MELO3C012013.2 | 95 | 16 | -2.526 | 4.15853E-11 | down |
| MELO3C012014.2 | 40 | 20 | -1.028 | 0.0168593 | down |
| MELO3C012015.2 | 58 | 10 | -2.471 | 5.64976E-06 | down |
| MELO3C012020.2 | 558 | 111 | -2.335 | 2.35609E-39 | down |
| MELO3C012023.2 | 2910 | 10754 | 1.886 | 1.06037E-109 | up |
| MELO3C012024.2 | 19 | 84 | 2.168 | 4.25381E-08 | up |
| MELO3C012027.2 | 35 | 516 | 3.885 | 2.05183E-48 | up |
| MELO3C012034.2 | 80 | 36 | -1.146 | 0.00048894 | down |
| MELO3C012039.2 | 77 | 21 | -1.842 | 1.20371E-05 | down |
| MELO3C012049.2 | 253 | 965 | 1.937 | 5.33815E-36 | up |
| MELO3C012052.2 | 6157 | 2471 | -1.317 | 3.74402E-20 | down |
| MELO3C012053.2 | 87 | 315 | 1.851 | 0.003479855 | up |
| MELO3C012055.2 | 6220 | 596 | -3.382 | 2.7802E-30 | down |
| MELO3C012061.2 | 5 | 0 | -4.991 | 0.009637982 | down |
| MELO3C012062.2 | 233 | 96 | -1.282 | 5.66952E-09 | down |
| MELO3C012064.2 | 76 | 18 | -2.043 | 0.000671352 | down |
| MELO3C012073.2 | 1903 | 841 | -1.178 | 4.24972E-43 | down |
| MELO3C012075.2 | 360 | 1475 | 2.037 | 1.43352E-36 | up |
| MELO3C012078.2 | 2776 | 364 | -2.935 | 1.3415E-152 | down |
| MELO3C012079.2 | 1912 | 6398 | 1.743 | 2.83941E-84 | up |
| MELO3C012082.2 | 1020 | 133 | -2.933 | 2.13038E-106 | down |
| MELO3C012090.2 | 874 | 333 | -1.391 | 2.06481E-32 | down |
| MELO3C012092.2 | 6 | 1 | -3.507 | 0.031551963 | down |
| MELO3C012093.2 | 25 | 7 | -1.968 | 0.001249427 | down |
| MELO3C012094.2 | 114 | 13 | -3.182 | 2.84033E-14 | down |
| MELO3C012099.2 | 11 | 1 | -4.116 | 0.01857872 | down |
| MELO3C012101.2 | 63 | 27 | -1.244 | 0.001685096 | down |
| MELO3C012103.2 | 2244 | 785 | -1.516 | 1.33638E-62 | down |
| MELO3C012107.2 | 651 | 288 | -1.177 | 1.50266E-11 | down |
| MELO3C012108.2 | 184 | 19 | -3.297 | 0.003889183 | down |
| MELO3C012111.2 | 426 | 903 | 1.082 | 1.05095E-18 | up |
| MELO3C012114.2 | 16 | 2 | -3.161 | 0.001683073 | down |
| MELO3C012124.2 | 411 | 953 | 1.216 | 4.85352E-22 | up |
| MELO3C012127.2 | 49 | 4 | -3.57 | 2.93262E-09 | down |
| MELO3C012128.2 | 62 | 140 | 1.177 | 3.72691E-06 | up |
| MELO3C012133.2 | 32 | 138 | 2.12 | 8.44672E-10 | up |
| MELO3C012134.2 | 4325 | 2085 | -1.053 | 1.48574E-48 | down |
| MELO3C012137.2 | 47 | 9 | -2.359 | 4.79056E-06 | down |
| MELO3C012139.2 | 330 | 1755 | 2.416 | 3.31485E-98 | up |
| MELO3C012142.2 | 146 | 329 | 1.176 | 1.97271E-09 | up |
| MELO3C012148.2 | 268 | 40 | -2.751 | 3.16198E-22 | down |
| MELO3C012149.2 | 449 | 1169 | 1.38 | 1.41811E-29 | up |
| MELO3C012151.2 | 39 | 5 | -2.882 | 2.65863E-06 | down |
| MELO3C012155.2 | 407 | 200 | -1.031 | 5.44205E-11 | down |
| MELO3C012158.2 | 83 | 319 | 1.935 | 1.72192E-20 | up |
| MELO3C012160.2 | 85 | 364 | 2.103 | 2.18323E-23 | up |
| MELO3C012162.2 | 77 | 392 | 2.355 | 0.001045926 | up |
| MELO3C012166.2 | 78 | 27 | -1.524 | 0.000289411 | down |
| MELO3C012167.2 | 248 | 945 | 1.925 | 1.18311E-24 | up |
| MELO3C012168.2 | 13 | 1 | -4.476 | 0.000753161 | down |
| MELO3C012169.2 | 167 | 478 | 1.518 | 2.03962E-22 | up |
| MELO3C012171.2 | 33356 | 14773 | -1.175 | 2.17241E-75 | down |
| MELO3C012175.2 | 1647 | 332 | -2.31 | 3.15821E-86 | down |
| MELO3C012178.2 | 1543 | 633 | -1.285 | 3.91707E-29 | down |
| MELO3C012180.2 | 1854 | 539 | -1.783 | 1.22994E-65 | down |
| MELO3C012181.2 | 232 | 22 | -3.381 | 5.67654E-34 | down |
| MELO3C012190.2 | 4 | 0 | -4.8 | 0.015738729 | down |
| MELO3C012191.2 | 7 | 1 | -3.635 | 0.01925728 | down |
| MELO3C012196.2 | 3289 | 8661 | 1.397 | 8.65964E-78 | up |
| MELO3C012200.2 | 519 | 48 | -3.405 | 2.06033E-44 | down |
| MELO3C012203.2 | 177 | 12 | -3.935 | 1.34297E-29 | down |
| MELO3C012204.2 | 67 | 10 | -2.821 | 1.41802E-08 | down |
| MELO3C012209.2 | 5150 | 1765 | -1.545 | 2.37429E-47 | down |
| MELO3C012222.2 | 4 | 510 | 6.83 | 6.03561E-49 | up |
| MELO3C012231.2 | 45 | 106 | 1.229 | 0.000131281 | up |
| MELO3C012241.2 | 10 | 28 | 1.398 | 0.024919404 | up |
| MELO3C012242.2 | 63 | 239 | 1.932 | 8.72684E-15 | up |
| MELO3C012243.2 | 116 | 47 | -1.285 | 2.52904E-06 | down |
| MELO3C012247.2 | 73 | 405 | 2.463 | 3.80852E-36 | up |
| MELO3C012248.2 | 13 | 62 | 2.235 | 1.0349E-06 | up |
| MELO3C012252.2 | 46665 | 13019 | -1.842 | 1.46609E-64 | down |
| MELO3C012253.2 | 1944 | 906 | -1.104 | 1.6273E-24 | down |
| MELO3C012254.2 | 5548 | 1394 | -1.993 | 4.06521E-123 | down |
| MELO3C012261.2 | 269 | 1236 | 2.199 | 1.95572E-47 | up |
| MELO3C012270.2 | 75 | 26 | -1.544 | 0.000120042 | down |
| MELO3C012273.2 | 21 | 988 | 5.561 | 1.11436E-128 | up |
| MELO3C012279.2 | 33 | 78 | 1.245 | 0.000264243 | up |
| MELO3C012284.2 | 1242 | 563 | -1.142 | 1.11926E-25 | down |
| MELO3C012286.2 | 251 | 733 | 1.549 | 3.13033E-24 | up |
| MELO3C012288.2 | 1972 | 664 | -1.57 | 2.82834E-34 | down |
| MELO3C012290.2 | 51 | 10 | -2.422 | 1.7533E-05 | down |
| MELO3C012302.2 | 5909 | 2175 | -1.442 | 4.89239E-51 | down |
| MELO3C012307.2 | 121 | 14 | -3.072 | 3.87202E-16 | down |
| MELO3C012316.2 | 1 | 9 | 3.521 | 0.016850545 | up |
| MELO3C012318.2 | 10 | 0 | -5.934 | 0.000201206 | down |
| MELO3C012322.2 | 278 | 685 | 1.298 | 6.99035E-13 | up |
| MELO3C012334.2 | 488 | 1239 | 1.343 | 2.5565E-16 | up |
| MELO3C012336.2 | 34 | 120 | 1.821 | 9.84318E-08 | up |
| MELO3C012344.2 | 1 | 14 | 4.2 | 0.001178723 | up |
| MELO3C012345.2 | 222 | 71 | -1.644 | 2.17776E-13 | down |
| MELO3C012346.2 | 73 | 9 | -3.053 | 6.63566E-12 | down |
| MELO3C012348.2 | 878 | 358 | -1.295 | 7.7522E-17 | down |
| MELO3C012349.2 | 32 | 157 | 2.283 | 2.78099E-12 | up |
| MELO3C012356.2 | 521 | 195 | -1.418 | 9.19593E-22 | down |
| MELO3C012372.2 | 6 | 364 | 5.838 | 3.19909E-50 | up |
| MELO3C012373.2 | 2100 | 901 | -1.221 | 5.50678E-31 | down |
| MELO3C012382.2 | 4596 | 2171 | -1.082 | 3.71255E-18 | down |
| MELO3C012388.2 | 66 | 27 | -1.304 | 0.003961397 | down |
| MELO3C012389.2 | 15694 | 6280 | -1.321 | 1.60566E-43 | down |
| MELO3C012403.2 | 10 | 41 | 2.017 | 5.63148E-05 | up |
| MELO3C012404.2 | 590 | 124 | -2.25 | 1.64249E-48 | down |
| MELO3C012408.2 | 21 | 1 | -4.192 | 1.92973E-05 | down |
| MELO3C012409.2 | 4791 | 1361 | -1.815 | 1.07958E-32 | down |
| MELO3C012412.2 | 182 | 74 | -1.31 | 1.01342E-06 | down |
| MELO3C012418.2 | 10 | 1 | -4.09 | 0.004728449 | down |
| MELO3C012424.2 | 2 | 16 | 3.098 | 0.001123311 | up |
| MELO3C012426.2 | 586 | 2092 | 1.837 | 1.86278E-54 | up |
| MELO3C012429.2 | 55 | 4 | -3.739 | 1.35347E-06 | down |
| MELO3C012433.2 | 84 | 863 | 3.36 | 1.22214E-41 | up |
| MELO3C012436.2 | 2865 | 1220 | -1.232 | 6.84417E-36 | down |
| MELO3C012437.2 | 1220 | 197 | -2.637 | 1.76748E-73 | down |
| MELO3C012438.2 | 951 | 23 | -5.352 | 2.51688E-148 | down |
| MELO3C012439.2 | 480 | 170 | -1.496 | 1.49877E-06 | down |
| MELO3C012440.2 | 160 | 341 | 1.086 | 7.87667E-11 | up |
| MELO3C012442.2 | 754 | 215 | -1.809 | 1.01173E-34 | down |
| MELO3C012452.2 | 105 | 218 | 1.054 | 3.6422E-05 | up |
| MELO3C012454.2 | 294 | 2681 | 3.189 | 3.87969E-60 | up |
| MELO3C012455.2 | 222 | 42 | -2.422 | 6.74116E-23 | down |
| MELO3C012456.2 | 745 | 167 | -2.159 | 1.98675E-23 | down |
| MELO3C012470.2 | 568 | 21 | -4.725 | 1.41316E-16 | down |
| MELO3C012471.2 | 814 | 1 | -10.431 | 4.94003E-24 | down |
| MELO3C012472.2 | 7427 | 3587 | -1.05 | 1.9103E-22 | down |
| MELO3C012476.2 | 85 | 22 | -1.959 | 8.72863E-06 | down |
| MELO3C012477.2 | 86 | 229 | 1.411 | 7.10337E-09 | up |
| MELO3C012484.2 | 181 | 81 | -1.165 | 0.000143833 | down |
| MELO3C012494.2 | 3262 | 1616 | -1.014 | 1.06844E-17 | down |
| MELO3C012498.2 | 26 | 6 | -2.198 | 0.001583543 | down |
| MELO3C012502.2 | 0 | 440 | 11.056 | 2.01785E-20 | up |
| MELO3C012508.2 | 3 | 53 | 4.34 | 1.25735E-08 | up |
| MELO3C012525.2 | 292 | 1836 | 2.658 | 2.10589E-80 | up |
| MELO3C012547.2 | 1378 | 20 | -6.097 | 3.6101E-162 | down |
| MELO3C012550.2 | 398 | 1207 | 1.602 | 0.014827272 | up |
| MELO3C012556.2 | 395 | 188 | -1.071 | 3.31307E-08 | down |
| MELO3C012559.2 | 2508 | 165 | -3.931 | 4.1856E-130 | down |
| MELO3C012570.2 | 1 | 18 | 4.562 | 0.000372325 | up |
| MELO3C012573.2 | 18 | 5 | -1.785 | 0.016289029 | down |
| MELO3C012575.2 | 306 | 137 | -1.158 | 4.74291E-11 | down |
| MELO3C012590.2 | 1262 | 276 | -2.191 | 1.26464E-39 | down |
| MELO3C012594.2 | 3 | 20 | 2.721 | 0.006364919 | up |
| MELO3C012595.2 | 43 | 14 | -1.635 | 0.002214083 | down |
| MELO3C012601.2 | 101 | 302 | 1.573 | 4.51085E-12 | up |
| MELO3C012606.2 | 58 | 127 | 1.135 | 2.80261E-05 | up |
| MELO3C012619.2 | 2 | 16 | 3.1 | 0.002497979 | up |
| MELO3C012626.2 | 20 | 3 | -2.727 | 0.000830036 | down |
| MELO3C012631.2 | 662 | 1463 | 1.145 | 2.91248E-22 | up |
| MELO3C012644.2 | 38 | 77 | 1.024 | 0.001784359 | up |
| MELO3C012645.2 | 1607 | 353 | -2.185 | 6.54932E-67 | down |
| MELO3C012656.2 | 5 | 0 | -4.986 | 0.009739763 | down |
| MELO3C012661.2 | 26 | 1 | -5.476 | 1.44201E-05 | down |
| MELO3C012686.2 | 2 | 17 | 2.96 | 0.001388861 | up |
| MELO3C012699.2 | 558 | 223 | -1.325 | 1.8599E-18 | down |
| MELO3C012701.2 | 15926 | 1792 | -3.151 | 3.9018E-23 | down |
| MELO3C012702.2 | 8189 | 466 | -4.134 | 9.50742E-41 | down |
| MELO3C012712.2 | 741 | 9 | -6.44 | 4.14972E-87 | down |
| MELO3C012714.2 | 151 | 570 | 1.918 | 7.9245E-30 | up |
| MELO3C012716.2 | 1803 | 4068 | 1.174 | 1.30343E-37 | up |
| MELO3C012717.2 | 391 | 1142 | 1.546 | 1.57929E-34 | up |
| MELO3C012727.2 | 328 | 108 | -1.601 | 5.32925E-09 | down |
| MELO3C012728.2 | 2278 | 1078 | -1.081 | 4.01373E-26 | down |
| MELO3C012740.2 | 463 | 224 | -1.048 | 2.31112E-11 | down |
| MELO3C012746.2 | 114 | 336 | 1.559 | 3.38876E-16 | up |
| MELO3C012753.2 | 74 | 207 | 1.485 | 1.28159E-07 | up |
| MELO3C012755.2 | 5 | 0 | -4.773 | 0.014936337 | down |
| MELO3C012802.2 | 9 | 0 | -5.812 | 0.000481035 | down |
| MELO3C012806.2 | 0 | 3168 | 13.907 | 1.31867E-21 | up |
| MELO3C012814.2 | 194 | 398 | 1.034 | 1.32594E-08 | up |
| MELO3C012819.2 | 711 | 288 | -1.302 | 2.0189E-22 | down |
| MELO3C012822.2 | 202 | 851 | 2.07 | 4.04072E-51 | up |
| MELO3C012837.2 | 75 | 32 | -1.216 | 0.002776737 | down |
| MELO3C012838.2 | 47 | 233 | 2.311 | 1.15986E-16 | up |
| MELO3C012852.2 | 28 | 113 | 2.045 | 2.31243E-09 | up |
| MELO3C012854.2 | 1698 | 80 | -4.402 | 1.42524E-124 | down |
| MELO3C012870.2 | 6 | 209 | 5.125 | 8.20862E-31 | up |
| MELO3C012874.2 | 132 | 1055 | 2.997 | 3.88743E-83 | up |
| MELO3C012885.2 | 2604 | 920 | -1.502 | 1.80018E-49 | down |
| MELO3C012902.2 | 1776 | 712 | -1.321 | 1.14523E-37 | down |
| MELO3C012906.2 | 9 | 174 | 4.276 | 1.23589E-28 | up |
| MELO3C012911.2 | 7550 | 23106 | 1.614 | 2.88289E-87 | up |
| MELO3C012912.2 | 101927 | 62 | -10.705 | 0 | down |
| MELO3C012916.2 | 7 | 0 | -5.405 | 0.010170791 | down |
| MELO3C012917.2 | 15 | 157 | 3.351 | 1.17453E-16 | up |
| MELO3C012920.2 | 54 | 1294 | 4.59 | 6.32753E-82 | up |
| MELO3C012921.2 | 151 | 713 | 2.233 | 5.53313E-44 | up |
| MELO3C012925.2 | 2792 | 6473 | 1.213 | 3.11968E-33 | up |
| MELO3C012944.2 | 79 | 318 | 2.018 | 8.34092E-26 | up |
| MELO3C012945.2 | 409 | 26 | -3.956 | 2.70778E-36 | down |
| MELO3C012956.2 | 666 | 211 | -1.653 | 1.7592E-21 | down |
| MELO3C012958.2 | 266 | 114 | -1.213 | 2.25181E-08 | down |
| MELO3C012960.2 | 7901 | 861 | -3.199 | 2.67767E-196 | down |
| MELO3C012962.2 | 208 | 630 | 1.597 | 2.16073E-19 | up |
| MELO3C012968.2 | 395 | 171 | -1.212 | 2.10694E-10 | down |
| MELO3C012970.2 | 168 | 2 | -6.161 | 5.14507E-27 | down |
| MELO3C012971.2 | 29 | 2 | -3.589 | 1.31977E-05 | down |
| MELO3C012982.2 | 125 | 322 | 1.368 | 2.13132E-08 | up |
| MELO3C012987.2 | 36 | 11 | -1.672 | 0.011492233 | down |
| MELO3C012992.2 | 97 | 836 | 3.114 | 1.12198E-84 | up |
| MELO3C012994.2 | 1229 | 44 | -4.785 | 8.25853E-133 | down |
| MELO3C013000.2 | 65255 | 26005 | -1.327 | 4.48504E-55 | down |
| MELO3C013002.2 | 1172 | 527 | -1.153 | 3.98165E-25 | down |
| MELO3C013003.2 | 143 | 43 | -1.725 | 2.2842E-11 | down |
| MELO3C013005.2 | 33 | 112 | 1.786 | 5.94816E-08 | up |
| MELO3C013006.2 | 130 | 18 | -2.837 | 6.2306E-16 | down |
| MELO3C013009.2 | 271 | 114 | -1.257 | 1.17584E-08 | down |
| MELO3C013013.2 | 1167 | 5171 | 2.147 | 1.545E-13 | up |
| MELO3C013014.2 | 271 | 21 | -3.712 | 1.09445E-43 | down |
| MELO3C013018.2 | 7 | 0 | -5.419 | 0.003144688 | down |
| MELO3C013034.2 | 4508 | 1357 | -1.733 | 4.60894E-80 | down |
| MELO3C013043.2 | 1 | 9 | 3.592 | 0.013768052 | up |
| MELO3C013047.2 | 307 | 10 | -4.95 | 1.19847E-42 | down |
| MELO3C013057.2 | 100 | 3 | -4.839 | 6.02547E-15 | down |
| MELO3C013082.2 | 4677 | 1968 | -1.249 | 9.87354E-54 | down |
| MELO3C013090.2 | 0 | 64 | 8.274 | 3.37914E-11 | up |
| MELO3C013099.2 | 23 | 0 | -7.144 | 3.90102E-07 | down |
| MELO3C013103.2 | 0 | 36 | 7.443 | 4.88071E-07 | up |
| MELO3C013111.2 | 8 | 589 | 6.301 | 3.85838E-64 | up |
| MELO3C013114.2 | 415 | 7 | -5.867 | 1.10431E-59 | down |
| MELO3C013122.2 | 1321 | 2689 | 1.025 | 3.70554E-21 | up |
| MELO3C013132.2 | 69 | 170 | 1.303 | 8.94995E-07 | up |
| MELO3C013152.2 | 383 | 822 | 1.103 | 3.85461E-19 | up |
| MELO3C013157.2 | 1100 | 15 | -6.223 | 2.53741E-148 | down |
| MELO3C013158.2 | 1 | 318 | 8.735 | 3.94824E-17 | up |
| MELO3C013171.2 | 2893 | 1283 | -1.173 | 1.08827E-30 | down |
| MELO3C013175.2 | 1202 | 2589 | 1.107 | 4.2807E-30 | up |
| MELO3C013186.2 | 228 | 615 | 1.434 | 4.95882E-28 | up |
| MELO3C013188.2 | 481 | 1318 | 1.454 | 1.02559E-39 | up |
| MELO3C013195.2 | 61 | 154 | 1.324 | 8.28979E-07 | up |
| MELO3C013203.2 | 933 | 278 | -1.748 | 5.06263E-16 | down |
| MELO3C013225.2 | 1126 | 2310 | 1.036 | 1.45841E-29 | up |
| MELO3C013232.2 | 171 | 671 | 1.969 | 1.02771E-31 | up |
| MELO3C013246.2 | 34 | 2 | -4.27 | 2.07011E-07 | down |
| MELO3C013250.2 | 7434 | 2291 | -1.698 | 3.38091E-91 | down |
| MELO3C013264.2 | 293 | 623 | 1.089 | 5.23733E-07 | up |
| MELO3C013273.2 | 18 | 89 | 2.325 | 4.90224E-08 | up |
| MELO3C013274.2 | 36 | 75 | 1.075 | 0.002622055 | up |
| MELO3C013276.2 | 219 | 504 | 1.203 | 4.27675E-18 | up |
| MELO3C013289.2 | 47 | 537 | 3.496 | 7.74141E-26 | up |
| MELO3C013302.2 | 24 | 6 | -2.143 | 0.000964803 | down |
| MELO3C013306.2 | 5358 | 244 | -4.459 | 0 | down |
| MELO3C013308.2 | 54 | 432 | 2.982 | 3.0944E-39 | up |
| MELO3C013310.2 | 12 | 0 | -6.119 | 9.06012E-05 | down |
| MELO3C013320.2 | 7315 | 2489 | -1.556 | 3.40038E-90 | down |
| MELO3C013322.2 | 502 | 209 | -1.262 | 5.96154E-17 | down |
| MELO3C013347.2 | 11441 | 259 | -5.466 | 1.72057E-294 | down |
| MELO3C013348.2 | 360 | 123 | -1.551 | 1.77504E-18 | down |
| MELO3C013350.2 | 18473 | 5418 | -1.77 | 3.91398E-75 | down |
| MELO3C013352.2 | 36 | 17 | -1.104 | 0.029600045 | down |
| MELO3C013353.2 | 1922 | 3963 | 1.044 | 3.63575E-30 | up |
| MELO3C013360.2 | 34 | 6 | -2.6 | 2.58044E-05 | down |
| MELO3C013361.2 | 173 | 348 | 1.007 | 3.93576E-08 | up |
| MELO3C013366.2 | 26532 | 2000 | -3.729 | 9.84606E-113 | down |
| MELO3C013376.2 | 54 | 249 | 2.205 | 1.86993E-11 | up |
| MELO3C013387.2 | 11 | 97 | 3.114 | 3.99825E-15 | up |
| MELO3C013403.2 | 73 | 12 | -2.589 | 8.28932E-11 | down |
| MELO3C013427.2 | 23 | 2 | -3.709 | 0.000610119 | down |
| MELO3C013428.2 | 529 | 255 | -1.055 | 2.8827E-14 | down |
| MELO3C013429.2 | 441 | 999 | 1.18 | 1.69364E-10 | up |
| MELO3C013436.2 | 48 | 403 | 3.063 | 1.12523E-05 | up |
| MELO3C013439.2 | 5 | 84 | 4.107 | 1.48922E-12 | up |
| MELO3C013446.2 | 121 | 47 | -1.346 | 0.000779078 | down |
| MELO3C013449.2 | 377 | 36 | -3.371 | 3.49259E-28 | down |
| MELO3C013451.2 | 0 | 8371 | 15.309 | 4.95408E-38 | up |
| MELO3C013455.2 | 1403 | 2903 | 1.05 | 1.75475E-32 | up |
| MELO3C013470.2 | 12308 | 6066 | -1.021 | 1.03959E-42 | down |
| MELO3C013473.2 | 38 | 82 | 1.124 | 0.000221659 | up |
| MELO3C013478.2 | 48 | 7 | -2.768 | 7.5307E-08 | down |
| MELO3C013480.2 | 824 | 269 | -1.616 | 1.01623E-22 | down |
| MELO3C013487.2 | 34 | 593 | 4.113 | 3.33602E-82 | up |
| MELO3C013495.2 | 325 | 875 | 1.431 | 1.24653E-17 | up |
| MELO3C013512.2 | 2031 | 609 | -1.737 | 1.87745E-26 | down |
| MELO3C013519.2 | 690 | 289 | -1.253 | 7.8532E-23 | down |
| MELO3C013524.2 | 752 | 213 | -1.819 | 1.52884E-31 | down |
| MELO3C013526.2 | 24 | 8 | -1.512 | 0.011726434 | down |
| MELO3C013538.2 | 15 | 3 | -2.368 | 0.004779753 | down |
| MELO3C013544.2 | 111 | 10 | -3.487 | 4.66168E-17 | down |
| MELO3C013552.2 | 49 | 197 | 2.014 | 9.93499E-13 | up |
| MELO3C013553.2 | 411 | 108 | -1.928 | 3.95592E-18 | down |
| MELO3C013561.2 | 12 | 55 | 2.116 | 5.10154E-06 | up |
| MELO3C013570.2 | 96 | 686 | 2.84 | 3.33743E-52 | up |
| MELO3C013577.2 | 167 | 44 | -1.914 | 6.16163E-13 | down |
| MELO3C013583.2 | 2684 | 7645 | 1.511 | 1.54652E-50 | up |
| MELO3C013586.2 | 23 | 88 | 1.946 | 6.30767E-06 | up |
| MELO3C013592.2 | 97 | 33 | -1.567 | 1.42089E-06 | down |
| MELO3C013594.2 | 7 | 1 | -3.593 | 0.020909735 | down |
| MELO3C013603.2 | 425 | 1082 | 1.348 | 8.20259E-21 | up |
| MELO3C013608.2 | 186 | 437 | 1.232 | 2.26511E-10 | up |
| MELO3C013612.2 | 32 | 81 | 1.345 | 0.000137586 | up |
| MELO3C013615.2 | 2204 | 332 | -2.733 | 2.1999E-116 | down |
| MELO3C013618.2 | 258 | 27 | -3.289 | 2.94105E-21 | down |
| MELO3C013622.2 | 157 | 911 | 2.535 | 7.45788E-71 | up |
| MELO3C013623.2 | 522 | 137 | -1.919 | 2.71153E-25 | down |
| MELO3C013625.2 | 223 | 33 | -2.736 | 3.51092E-25 | down |
| MELO3C013626.2 | 170 | 372 | 1.131 | 4.55499E-07 | up |
| MELO3C013632.2 | 2940 | 523 | -2.49 | 2.70979E-48 | down |
| MELO3C013634.2 | 3473 | 1452 | -1.258 | 1.53738E-44 | down |
| MELO3C013641.2 | 126 | 765 | 2.603 | 4.90082E-26 | up |
| MELO3C013645.2 | 3866 | 1714 | -1.173 | 4.035E-27 | down |
| MELO3C013647.2 | 837 | 0 | -12.326 | 2.59398E-25 | down |
| MELO3C013665.2 | 154 | 1583 | 3.359 | 6.88781E-55 | up |
| MELO3C013667.2 | 418 | 1084 | 1.375 | 4.5991E-28 | up |
| MELO3C013669.2 | 436 | 24 | -4.174 | 5.99738E-53 | down |
| MELO3C013672.2 | 395 | 1104 | 1.483 | 7.24607E-40 | up |
| MELO3C013677.2 | 549 | 45 | -3.589 | 4.66867E-43 | down |
| MELO3C013681.2 | 254 | 534 | 1.068 | 8.68948E-13 | up |
| MELO3C013682.2 | 11 | 305 | 4.753 | 1.13463E-10 | up |
| MELO3C013686.2 | 64 | 828 | 3.707 | 1.17219E-45 | up |
| MELO3C013687.2 | 459 | 1168 | 1.349 | 3.92236E-15 | up |
| MELO3C013692.2 | 713 | 357 | -1 | 2.76003E-15 | down |
| MELO3C013693.2 | 61 | 5 | -3.535 | 2.45209E-10 | down |
| MELO3C013699.2 | 291 | 2330 | 3.004 | 5.40371E-80 | up |
| MELO3C013708.2 | 433 | 122 | -1.829 | 1.61795E-20 | down |
| MELO3C013710.2 | 2596 | 309 | -3.069 | 2.25419E-62 | down |
| MELO3C013715.2 | 884 | 1961 | 1.15 | 1.56432E-23 | up |
| MELO3C013726.2 | 1075 | 2184 | 1.024 | 2.03218E-27 | up |
| MELO3C013727.2 | 1007 | 4266 | 2.083 | 8.26382E-56 | up |
| MELO3C013734.2 | 10 | 2 | -2.528 | 0.026117639 | down |
| MELO3C013737.2 | 17 | 71 | 2.073 | 5.87522E-07 | up |
| MELO3C013738.2 | 2931 | 1451 | -1.014 | 1.01062E-23 | down |
| MELO3C013747.2 | 1423 | 678 | -1.069 | 4.58348E-28 | down |
| MELO3C013751.2 | 212 | 679 | 1.682 | 1.93032E-17 | up |
| MELO3C013752.2 | 55 | 286 | 2.378 | 2.02279E-14 | up |
| MELO3C013758.2 | 288 | 592 | 1.039 | 1.11716E-10 | up |
| MELO3C013763.2 | 6 | 0 | -5.229 | 0.014895941 | down |
| MELO3C013765.2 | 14875 | 5286 | -1.493 | 6.48049E-41 | down |
| MELO3C013771.2 | 992 | 131 | -2.926 | 6.68209E-101 | down |
| MELO3C013774.2 | 4029 | 899 | -2.163 | 1.21664E-64 | down |
| MELO3C013778.2 | 446 | 212 | -1.073 | 1.07748E-09 | down |
| MELO3C013780.2 | 129 | 285 | 1.147 | 1.0185E-09 | up |
| MELO3C013782.2 | 0 | 94 | 8.835 | 7.12441E-13 | up |
| MELO3C013784.2 | 103 | 276 | 1.421 | 1.05037E-10 | up |
| MELO3C013790.2 | 188 | 889 | 2.243 | 4.01548E-33 | up |
| MELO3C013793.2 | 4751 | 2165 | -1.135 | 4.37494E-28 | down |
| MELO3C013801.2 | 4048 | 676 | -2.584 | 3.2993E-156 | down |
| MELO3C013805.2 | 131 | 362 | 1.471 | 7.92173E-18 | up |
| MELO3C013807.2 | 6 | 37 | 2.62 | 8.2865E-06 | up |
| MELO3C013817.2 | 1261 | 193 | -2.706 | 1.0333E-83 | down |
| MELO3C013821.2 | 1809 | 3801 | 1.071 | 1.77768E-26 | up |
| MELO3C013828.2 | 4042 | 1722 | -1.231 | 1.60649E-30 | down |
| MELO3C013829.2 | 16 | 35 | 1.103 | 0.024769751 | up |
| MELO3C013843.2 | 778 | 221 | -1.821 | 3.0911E-42 | down |
| MELO3C013845.2 | 69 | 0 | -8.721 | 1.1547E-11 | down |
| MELO3C013852.2 | 828 | 342 | -1.274 | 1.01223E-23 | down |
| MELO3C013859.2 | 8887 | 24 | -8.538 | 0 | down |
| MELO3C013862.2 | 911 | 214 | -2.091 | 4.31215E-48 | down |
| MELO3C013867.2 | 100 | 552 | 2.465 | 6.05763E-28 | up |
| MELO3C013868.2 | 0 | 3073 | 13.863 | 9.16405E-30 | up |
| MELO3C013870.2 | 2350 | 1128 | -1.059 | 4.37072E-31 | down |
| MELO3C013871.2 | 1027 | 503 | -1.028 | 5.40529E-24 | down |
| MELO3C013872.2 | 894 | 376 | -1.245 | 3.37427E-15 | down |
| MELO3C013875.2 | 5992 | 15843 | 1.403 | 2.8914E-39 | up |
| MELO3C013880.2 | 892 | 106 | -3.068 | 1.67204E-41 | down |
| MELO3C013885.2 | 4732 | 2243 | -1.078 | 1.19138E-28 | down |
| MELO3C013889.2 | 72 | 389 | 2.445 | 1.44405E-35 | up |
| MELO3C013893.2 | 498 | 93 | -2.414 | 2.22965E-37 | down |
| MELO3C013899.2 | 9771 | 4263 | -1.197 | 8.6419E-35 | down |
| MELO3C013905.2 | 295 | 117 | -1.335 | 3.88161E-13 | down |
| MELO3C013906.2 | 3584 | 336 | -3.414 | 2.13411E-234 | down |
| MELO3C013907.2 | 288 | 88 | -1.699 | 1.66275E-13 | down |
| MELO3C013919.2 | 202 | 2 | -6.839 | 1.13142E-26 | down |
| MELO3C013922.2 | 399 | 99 | -1.998 | 1.76666E-12 | down |
| MELO3C013923.2 | 2929 | 810 | -1.853 | 2.48549E-32 | down |
| MELO3C013924.2 | 301 | 139 | -1.121 | 2.65269E-09 | down |
| MELO3C013925.2 | 17691 | 3597 | -2.298 | 1.13282E-219 | down |
| MELO3C013926.2 | 99 | 354 | 1.835 | 4.74841E-11 | up |
| MELO3C013927.2 | 1174 | 581 | -1.016 | 6.51589E-15 | down |
| MELO3C013934.2 | 17 | 3 | -2.526 | 0.003159724 | down |
| MELO3C013935.2 | 273 | 1 | -7.534 | 1.94055E-28 | down |
| MELO3C013936.2 | 157 | 75 | -1.061 | 8.66968E-05 | down |
| MELO3C013941.2 | 575 | 213 | -1.432 | 7.31166E-22 | down |
| MELO3C013943.2 | 897 | 2395 | 1.418 | 3.6192E-23 | up |
| MELO3C013945.2 | 7198 | 2615 | -1.461 | 3.88756E-32 | down |
| MELO3C013946.2 | 171 | 868 | 2.34 | 3.59359E-71 | up |
| MELO3C013961.2 | 470 | 3175 | 2.755 | 7.81213E-133 | up |
| MELO3C013962.2 | 0 | 17 | 5.35 | 0.000184508 | up |
| MELO3C013964.2 | 2968 | 1131 | -1.393 | 1.12774E-34 | down |
| MELO3C013968.2 | 659 | 1689 | 1.359 | 1.5733E-28 | up |
| MELO3C013969.2 | 1532 | 437 | -1.811 | 2.81429E-46 | down |
| MELO3C013972.2 | 43 | 188 | 2.118 | 2.01986E-15 | up |
| MELO3C013973.2 | 180 | 525 | 1.543 | 5.05117E-27 | up |
| MELO3C013989.2 | 42 | 157 | 1.879 | 8.16204E-10 | up |
| MELO3C014002.2 | 10 | 73 | 2.875 | 1.80779E-07 | up |
| MELO3C014005.2 | 110 | 1480 | 3.754 | 3.55583E-187 | up |
| MELO3C014007.2 | 714 | 2555 | 1.84 | 9.36491E-93 | up |
| MELO3C014009.2 | 11074 | 2641 | -2.068 | 2.97317E-130 | down |
| MELO3C014015.2 | 3 | 41 | 3.766 | 7.4106E-06 | up |
| MELO3C014023.2 | 12 | 29 | 1.262 | 0.016580635 | up |
| MELO3C014025.2 | 1155 | 465 | -1.312 | 5.20874E-27 | down |
| MELO3C014026.2 | 61 | 0 | -8.549 | 8.00833E-12 | down |
| MELO3C014027.2 | 179 | 376 | 1.069 | 1.52348E-11 | up |
| MELO3C014033.2 | 0 | 6 | 4.938 | 0.007202662 | up |
| MELO3C014040.2 | 140 | 0 | -9.748 | 2.20236E-15 | down |
| MELO3C014042.2 | 0 | 310 | 10.552 | 1.17499E-18 | up |
| MELO3C014045.2 | 112 | 19722 | 7.46 | 2.25149E-93 | up |
| MELO3C014047.2 | 51 | 6 | -3.296 | 9.34066E-08 | down |
| MELO3C014056.2 | 14 | 1569 | 6.866 | 6.76034E-125 | up |
| MELO3C014062.2 | 13 | 3 | -2.226 | 0.028725118 | down |
| MELO3C014076.2 | 0 | 1075 | 12.348 | 2.0268E-25 | up |
| MELO3C014089.2 | 1946 | 214 | -3.188 | 5.15545E-153 | down |
| MELO3C014099.2 | 1353 | 4362 | 1.688 | 7.69485E-31 | up |
| MELO3C014105.2 | 244 | 44 | -2.475 | 1.19467E-24 | down |
| MELO3C014107.2 | 26 | 149 | 2.524 | 3.51507E-13 | up |
| MELO3C014121.2 | 5 | 85 | 4.122 | 1.56799E-14 | up |
| MELO3C014124.2 | 12858 | 2023 | -2.668 | 3.18012E-187 | down |
| MELO3C014128.2 | 284 | 19 | -3.943 | 9.33332E-42 | down |
| MELO3C014131.2 | 0 | 79 | 8.585 | 3.83523E-12 | up |
| MELO3C014132.2 | 368 | 173 | -1.091 | 5.57196E-12 | down |
| MELO3C014140.2 | 211 | 581 | 1.462 | 1.22485E-14 | up |
| MELO3C014154.2 | 38 | 100 | 1.404 | 4.81685E-05 | up |
| MELO3C014156.2 | 2415 | 1101 | -1.133 | 9.50053E-39 | down |
| MELO3C014159.2 | 58 | 23 | -1.322 | 0.001055015 | down |
| MELO3C014161.2 | 23597 | 11139 | -1.083 | 2.86E-39 | down |
| MELO3C014168.2 | 865 | 401 | -1.111 | 6.39765E-15 | down |
| MELO3C014175.2 | 2058 | 1003 | -1.035 | 5.01323E-16 | down |
| MELO3C014178.2 | 1970 | 158 | -3.642 | 1.75506E-140 | down |
| MELO3C014190.2 | 114 | 54 | -1.078 | 0.000361268 | down |
| MELO3C014199.2 | 0 | 180 | 9.775 | 4.71999E-16 | up |
| MELO3C014208.2 | 65 | 668 | 3.35 | 2.95006E-65 | up |
| MELO3C014209.2 | 0 | 9 | 5.356 | 0.002318565 | up |
| MELO3C014224.2 | 253 | 26 | -3.265 | 2.44901E-15 | down |
| MELO3C014227.2 | 55 | 5 | -3.432 | 3.59455E-08 | down |
| MELO3C014228.2 | 481 | 2849 | 2.565 | 6.88323E-06 | up |
| MELO3C014232.2 | 1908 | 4066 | 1.091 | 5.1984E-30 | up |
| MELO3C014236.2 | 52 | 20 | -1.364 | 0.001230035 | down |
| MELO3C014238.2 | 623 | 3869 | 2.635 | 2.81674E-57 | up |
| MELO3C014240.2 | 261 | 23 | -3.486 | 8.71694E-06 | down |
| MELO3C014241.2 | 8165 | 16890 | 1.049 | 1.47468E-15 | up |
| MELO3C014247.2 | 42 | 2 | -4.157 | 3.67083E-08 | down |
| MELO3C014256.2 | 664 | 2948 | 2.15 | 6.20656E-40 | up |
| MELO3C014257.2 | 45 | 606 | 3.754 | 2.04153E-43 | up |
| MELO3C014260.2 | 545 | 2044 | 1.908 | 1.54482E-62 | up |
| MELO3C014268.2 | 4329 | 2035 | -1.089 | 2.30403E-28 | down |
| MELO3C014279.2 | 4 | 31 | 3.049 | 8.82892E-05 | up |
| MELO3C014288.2 | 41 | 201 | 2.288 | 1.43333E-15 | up |
| MELO3C014289.2 | 2265 | 454 | -2.321 | 6.9029E-34 | down |
| MELO3C014293.2 | 773 | 2115 | 1.453 | 2.20541E-38 | up |
| MELO3C014294.2 | 2266 | 166 | -3.771 | 5.8602E-09 | down |
| MELO3C014297.2 | 272 | 574 | 1.078 | 1.67869E-13 | up |
| MELO3C014299.2 | 922 | 455 | -1.019 | 0.016733677 | down |
| MELO3C014304.2 | 59 | 0 | -8.489 | 3.35061E-05 | down |
| MELO3C014305.2 | 413 | 10 | -5.411 | 1.35596E-53 | down |
| MELO3C014309.2 | 827 | 374 | -1.149 | 1.64676E-21 | down |
| MELO3C014314.2 | 0 | 89 | 8.752 | 3.7688E-12 | up |
| MELO3C014317.2 | 56 | 640 | 3.523 | 2.11384E-06 | up |
| MELO3C014321.2 | 16108 | 7283 | -1.145 | 2.55871E-27 | down |
| MELO3C014324.2 | 159 | 514 | 1.692 | 1.61739E-13 | up |
| MELO3C014337.2 | 2154 | 37 | -5.884 | 7.08536E-53 | down |
| MELO3C014347.2 | 51 | 136 | 1.416 | 1.05884E-07 | up |
| MELO3C014353.2 | 136 | 635 | 2.219 | 8.34777E-37 | up |
| MELO3C014379.2 | 15731 | 6885 | -1.192 | 1.5951E-74 | down |
| MELO3C014391.2 | 3355 | 601 | -2.483 | 3.22261E-168 | down |
| MELO3C014392.2 | 60 | 150 | 1.33 | 1.59816E-08 | up |
| MELO3C014393.2 | 1129 | 206 | -2.456 | 8.11562E-80 | down |
| MELO3C014398.2 | 17 | 41 | 1.287 | 0.006853852 | up |
| MELO3C014400.2 | 2291 | 393 | -2.542 | 6.62149E-85 | down |
| MELO3C014401.2 | 2794 | 612 | -2.192 | 3.86032E-131 | down |
| MELO3C014402.2 | 2 | 24 | 3.168 | 0.000682771 | up |
| MELO3C014409.2 | 66 | 162 | 1.291 | 3.11648E-05 | up |
| MELO3C014412.2 | 137 | 19 | -2.864 | 6.56279E-15 | down |
| MELO3C014419.2 | 4 | 17 | 2.163 | 0.016815372 | up |
| MELO3C014420.2 | 6157 | 2558 | -1.267 | 4.69748E-35 | down |
| MELO3C014425.2 | 1 | 18 | 3.973 | 0.001688162 | up |
| MELO3C014430.2 | 235 | 4100 | 4.126 | 2.70681E-138 | up |
| MELO3C014432.2 | 154 | 913 | 2.563 | 3.32462E-32 | up |
| MELO3C014433.2 | 88 | 27 | -1.704 | 1.73827E-06 | down |
| MELO3C014436.2 | 2521 | 732 | -1.785 | 3.99341E-49 | down |
| MELO3C014437.2 | 7972 | 142 | -5.813 | 3.25842E-17 | down |
| MELO3C014438.2 | 16 | 1 | -3.462 | 0.003542143 | down |
| MELO3C014443.2 | 418 | 947 | 1.179 | 1.00295E-07 | up |
| MELO3C014444.2 | 1 | 14 | 3.194 | 0.0028853 | up |
| MELO3C014445.2 | 14 | 94 | 2.692 | 4.8851E-10 | up |
| MELO3C014448.2 | 147 | 480 | 1.705 | 1.49471E-20 | up |
| MELO3C014463.2 | 0 | 9 | 5.356 | 0.001201682 | up |
| MELO3C014475.2 | 131 | 797 | 2.606 | 1.02299E-06 | up |
| MELO3C014476.2 | 376 | 857 | 1.189 | 7.25863E-21 | up |
| MELO3C014477.2 | 242 | 492 | 1.025 | 2.89152E-13 | up |
| MELO3C014478.2 | 24 | 202 | 3.064 | 1.03265E-15 | up |
| MELO3C014481.2 | 3242 | 238 | -3.765 | 7.56432E-220 | down |
| MELO3C014486.2 | 2 | 18 | 3.545 | 0.00155007 | up |
| MELO3C014489.2 | 140 | 387 | 1.465 | 3.20097E-15 | up |
| MELO3C014495.2 | 19 | 67 | 1.8 | 0.000209192 | up |
| MELO3C014498.2 | 67 | 447 | 2.746 | 1.28718E-34 | up |
| MELO3C014507.2 | 461 | 225 | -1.033 | 7.67661E-12 | down |
| MELO3C014511.2 | 13 | 2478 | 7.531 | 1.70549E-143 | up |
| MELO3C014512.2 | 8 | 30 | 1.899 | 0.002078892 | up |
| MELO3C014513.2 | 3140 | 1451 | -1.114 | 3.1875E-26 | down |
| MELO3C014514.2 | 402 | 819 | 1.026 | 1.60529E-17 | up |
| MELO3C014523.2 | 20 | 60 | 1.544 | 0.000566914 | up |
| MELO3C014526.2 | 1362 | 2848 | 1.064 | 4.40039E-30 | up |
| MELO3C014527.2 | 4373 | 11849 | 1.438 | 8.38869E-63 | up |
| MELO3C014530.2 | 251 | 109 | -1.205 | 5.09786E-07 | down |
| MELO3C014535.2 | 83 | 270 | 1.708 | 1.53463E-18 | up |
| MELO3C014564.2 | 379 | 964 | 1.35 | 1.2744E-19 | up |
| MELO3C014567.2 | 407 | 858 | 1.075 | 9.04173E-15 | up |
| MELO3C014568.2 | 3848 | 9 | -8.736 | 3.2116E-102 | down |
| MELO3C014571.2 | 5240 | 966 | -2.441 | 8.5301E-140 | down |
| MELO3C014578.2 | 1510 | 3308 | 1.131 | 9.57855E-36 | up |
| MELO3C014579.2 | 27 | 361 | 3.71 | 3.55498E-45 | up |
| MELO3C014584.2 | 3434 | 1694 | -1.019 | 3.80757E-30 | down |
| MELO3C014587.2 | 8555 | 442 | -4.275 | 0 | down |
| MELO3C014588.2 | 188 | 803 | 2.095 | 2.33083E-48 | up |
| MELO3C014589.2 | 575 | 37 | -3.954 | 1.52011E-94 | down |
| MELO3C014595.2 | 705 | 1496 | 1.085 | 1.38713E-23 | up |
| MELO3C014596.2 | 5967 | 1942 | -1.619 | 1.84306E-50 | down |
| MELO3C014599.2 | 1726 | 444 | -1.96 | 4.45219E-47 | down |
| MELO3C014602.2 | 9 | 51 | 2.563 | 1.28617E-06 | up |
| MELO3C014622.2 | 677 | 159 | -2.092 | 8.14654E-53 | down |
| MELO3C014624.2 | 71 | 29 | -1.315 | 0.0002309 | down |
| MELO3C014625.2 | 235 | 3 | -6.183 | 1.74468E-36 | down |
| MELO3C014630.2 | 368 | 51 | -2.864 | 0.000128638 | down |
| MELO3C014632.2 | 2 | 302 | 7.073 | 4.16251E-30 | up |
| MELO3C014638.2 | 46 | 111 | 1.275 | 0.00053864 | up |
| MELO3C014648.2 | 1496 | 268 | -2.482 | 4.87803E-76 | down |
| MELO3C014652.2 | 609 | 121 | -2.33 | 1.32823E-13 | down |
| MELO3C014653.2 | 22 | 2 | -3.275 | 0.008076258 | down |
| MELO3C014657.2 | 3 | 27 | 2.999 | 6.27094E-05 | up |
| MELO3C014658.2 | 142 | 428 | 1.596 | 0.007542505 | up |
| MELO3C014661.2 | 1895 | 633 | -1.583 | 2.47827E-56 | down |
| MELO3C014666.2 | 1698 | 185 | -3.192 | 1.55223E-56 | down |
| MELO3C014677.2 | 3 | 15 | 2.13 | 0.019376298 | up |
| MELO3C014680.2 | 9 | 1 | -4.042 | 0.025795516 | down |
| MELO3C014683.2 | 227 | 867 | 1.934 | 4.70973E-16 | up |
| MELO3C014688.2 | 390 | 133 | -1.555 | 1.9569E-17 | down |
| MELO3C014692.2 | 455 | 116 | -1.963 | 2.5561E-26 | down |
| MELO3C014696.2 | 90 | 9 | -3.284 | 5.95152E-16 | down |
| MELO3C014697.2 | 357 | 149 | -1.266 | 4.79281E-13 | down |
| MELO3C014698.2 | 442 | 1286 | 1.54 | 7.98611E-35 | up |
| MELO3C014701.2 | 194 | 45 | -2.101 | 7.53339E-15 | down |
| MELO3C014709.2 | 304 | 51 | -2.574 | 5.93563E-28 | down |
| MELO3C014714.2 | 975 | 365 | -1.418 | 4.74728E-29 | down |
| MELO3C014717.2 | 152 | 330 | 1.119 | 7.48579E-09 | up |
| MELO3C014723.2 | 3265 | 1509 | -1.114 | 1.9203E-45 | down |
| MELO3C014724.2 | 350 | 77 | -2.195 | 1.72005E-17 | down |
| MELO3C014728.2 | 3420 | 1029 | -1.734 | 4.24483E-40 | down |
| MELO3C014752.2 | 26363 | 9028 | -1.546 | 2.5185E-68 | down |
| MELO3C014757.2 | 15 | 3 | -2.4 | 0.010452126 | down |
| MELO3C014759.2 | 28 | 10 | -1.457 | 0.015557383 | down |
| MELO3C014762.2 | 65 | 9 | -2.853 | 3.5269E-06 | down |
| MELO3C014774.2 | 1059 | 3165 | 1.58 | 1.3233E-44 | up |
| MELO3C014795.2 | 617 | 1709 | 1.471 | 5.99575E-22 | up |
| MELO3C014803.2 | 84 | 235 | 1.479 | 3.88965E-12 | up |
| MELO3C014807.2 | 13 | 45 | 1.777 | 0.000128999 | up |
| MELO3C014810.2 | 7 | 30 | 2.069 | 0.002072665 | up |
| MELO3C014812.2 | 77 | 201 | 1.374 | 0.000164821 | up |
| MELO3C014815.2 | 62 | 16 | -1.958 | 1.75144E-06 | down |
| MELO3C014821.2 | 78 | 403 | 2.381 | 6.69068E-29 | up |
| MELO3C014824.2 | 7493 | 3363 | -1.156 | 4.56419E-54 | down |
| MELO3C014826.2 | 1802 | 597 | -1.595 | 1.26827E-39 | down |
| MELO3C014827.2 | 15 | 1 | -4.69 | 0.000370796 | down |
| MELO3C014853.2 | 52 | 3 | -4.167 | 4.54415E-11 | down |
| MELO3C014874.2 | 2116 | 1001 | -1.081 | 1.40642E-32 | down |
| MELO3C014875.2 | 3110 | 606 | -2.358 | 2.61463E-103 | down |
| MELO3C014881.2 | 1 | 9 | 2.962 | 0.030705125 | up |
| MELO3C014888.2 | 4138 | 1295 | -1.677 | 2.22703E-61 | down |
| MELO3C014896.2 | 469 | 79 | -2.558 | 6.58404E-45 | down |
| MELO3C014897.2 | 39158 | 14838 | -1.4 | 7.4385E-25 | down |
| MELO3C014898.2 | 14 | 32 | 1.227 | 0.02477427 | up |
| MELO3C014905.2 | 4 | 26 | 2.515 | 0.000226695 | up |
| MELO3C014907.2 | 57 | 170 | 1.572 | 6.21478E-09 | up |
| MELO3C014909.2 | 99 | 6 | -4.132 | 2.142E-15 | down |
| MELO3C014912.2 | 1 | 9 | 3.538 | 0.014530406 | up |
| MELO3C014926.2 | 6707 | 3150 | -1.091 | 7.32733E-23 | down |
| MELO3C014935.2 | 71 | 235 | 1.712 | 9.41396E-15 | up |
| MELO3C014936.2 | 72 | 248 | 1.793 | 1.62483E-14 | up |
| MELO3C014944.2 | 47 | 1 | -5.328 | 1.00472E-09 | down |
| MELO3C014949.2 | 0 | 171 | 9.695 | 8.45579E-16 | up |
| MELO3C014959.2 | 24 | 73 | 1.597 | 2.62535E-05 | up |
| MELO3C014984.2 | 69 | 455 | 2.73 | 2.64161E-33 | up |
| MELO3C014986.2 | 5 | 320 | 6.043 | 5.2129E-41 | up |
| MELO3C014990.2 | 0 | 31 | 6.276 | 2.04777E-06 | up |
| MELO3C014991.2 | 18426 | 861 | -4.419 | 1.12171E-21 | down |
| MELO3C015002.2 | 25 | 182 | 2.884 | 8.14642E-12 | up |
| MELO3C015005.2 | 134 | 0 | -9.685 | 4.97747E-15 | down |
| MELO3C015011.2 | 2449 | 101 | -4.594 | 1.31992E-104 | down |
| MELO3C015021.2 | 79 | 8 | -3.311 | 3.5695E-12 | down |
| MELO3C015024.2 | 272 | 102 | -1.411 | 8.8413E-10 | down |
| MELO3C015029.2 | 0 | 7 | 4.095 | 0.012752036 | up |
| MELO3C015055.2 | 107 | 303 | 1.503 | 3.1644E-07 | up |
| MELO3C015067.2 | 1101 | 429 | -1.359 | 4.42437E-12 | down |
| MELO3C015071.2 | 7 | 1 | -3.639 | 0.019723568 | down |
| MELO3C015076.2 | 41042 | 1865 | -4.46 | 0 | down |
| MELO3C015079.2 | 131 | 277 | 1.087 | 1.13439E-05 | up |
| MELO3C015093.2 | 123 | 4357 | 5.142 | 4.18237E-36 | up |
| MELO3C015101.2 | 247 | 31 | -2.991 | 4.2575E-34 | down |
| MELO3C015109.2 | 3543 | 1426 | -1.313 | 7.10278E-16 | down |
| MELO3C015118.2 | 386 | 64 | -2.587 | 1.26088E-34 | down |
| MELO3C015119.2 | 131 | 53 | -1.313 | 3.24658E-07 | down |
| MELO3C015123.2 | 9 | 38 | 1.988 | 0.001253978 | up |
| MELO3C015128.2 | 68 | 2 | -5.269 | 3.91897E-13 | down |
| MELO3C015129.2 | 32 | 5 | -2.836 | 4.98033E-05 | down |
| MELO3C015140.2 | 265 | 2 | -7.093 | 1.07745E-07 | down |
| MELO3C015149.2 | 42 | 151 | 1.861 | 6.82373E-12 | up |
| MELO3C015151.2 | 12222 | 3206 | -1.931 | 4.41815E-76 | down |
| MELO3C015152.2 | 1144 | 312 | -1.876 | 9.1746E-43 | down |
| MELO3C015155.2 | 494 | 2005 | 2.023 | 4.66857E-75 | up |
| MELO3C015183.2 | 35 | 3 | -3.878 | 6.93679E-08 | down |
| MELO3C015185.2 | 317 | 2846 | 3.169 | 0.00013726 | up |
| MELO3C015189.2 | 4427 | 1 | -12.876 | 3.34009E-36 | down |
| MELO3C015193.2 | 1824 | 674 | -1.439 | 3.53484E-30 | down |
| MELO3C015208.2 | 30 | 95 | 1.69 | 5.77796E-07 | up |
| MELO3C015209.2 | 9 | 82 | 3.211 | 4.88561E-13 | up |
| MELO3C015216.2 | 497 | 139 | -1.838 | 1.21771E-18 | down |
| MELO3C015221.2 | 61 | 13 | -2.264 | 0.00052386 | down |
| MELO3C015228.2 | 131 | 525 | 2.008 | 3.20245E-30 | up |
| MELO3C015230.2 | 186 | 1911 | 3.362 | 7.9289E-112 | up |
| MELO3C015231.2 | 17525 | 5962 | -1.556 | 6.78838E-48 | down |
| MELO3C015243.2 | 102 | 479 | 2.225 | 1.04815E-17 | up |
| MELO3C015253.2 | 164 | 41 | -2.019 | 1.57396E-11 | down |
| MELO3C015255.2 | 7343 | 2459 | -1.579 | 4.26067E-45 | down |
| MELO3C015257.2 | 122 | 28 | -2.143 | 6.80099E-07 | down |
| MELO3C015259.2 | 0 | 77 | 8.54 | 6.86754E-12 | up |
| MELO3C015260.2 | 346 | 1144 | 1.724 | 1.86519E-45 | up |
| MELO3C015261.2 | 0 | 143 | 9.441 | 7.32802E-15 | up |
| MELO3C015276.2 | 210 | 435 | 1.047 | 1.06411E-12 | up |
| MELO3C015277.2 | 331 | 137 | -1.276 | 2.76276E-07 | down |
| MELO3C015291.2 | 212 | 677 | 1.673 | 2.49931E-30 | up |
| MELO3C015294.2 | 4929 | 1654 | -1.576 | 3.92511E-61 | down |
| MELO3C015297.2 | 155 | 72 | -1.097 | 0.00012162 | down |
| MELO3C015303.2 | 299 | 639 | 1.095 | 2.41916E-15 | up |
| MELO3C015306.2 | 5016 | 53 | -6.562 | 6.41608E-53 | down |
| MELO3C015312.2 | 16 | 41 | 1.378 | 0.008113701 | up |
| MELO3C015313.2 | 12 | 184 | 3.933 | 9.1696E-27 | up |
| MELO3C015329.2 | 79 | 20 | -1.949 | 5.48245E-07 | down |
| MELO3C015331.2 | 106 | 48 | -1.125 | 3.67865E-05 | down |
| MELO3C015337.2 | 7772 | 41122 | 2.404 | 9.09378E-116 | up |
| MELO3C015338.2 | 3235 | 1340 | -1.271 | 1.43137E-44 | down |
| MELO3C015340.2 | 69 | 32 | -1.118 | 0.00212968 | down |
| MELO3C015351.2 | 939 | 321 | -1.549 | 1.01648E-34 | down |
| MELO3C015353.2 | 359 | 69 | -2.376 | 3.62462E-37 | down |
| MELO3C015354.2 | 44 | 121 | 1.456 | 0.000133586 | up |
| MELO3C015360.2 | 3069 | 1399 | -1.134 | 3.01018E-43 | down |
| MELO3C015374.2 | 3139 | 9315 | 1.569 | 5.6374E-73 | up |
| MELO3C015378.2 | 2308 | 761 | -1.601 | 2.45436E-67 | down |
| MELO3C015387.2 | 39 | 734 | 4.257 | 2.16385E-91 | up |
| MELO3C015396.2 | 1877 | 774 | -1.279 | 8.96618E-28 | down |
| MELO3C015398.2 | 1515 | 226 | -2.744 | 1.27202E-123 | down |
| MELO3C015399.2 | 6338 | 2896 | -1.13 | 3.83812E-29 | down |
| MELO3C015407.2 | 26740 | 10540 | -1.343 | 2.6388E-28 | down |
| MELO3C015408.2 | 1658 | 587 | -1.498 | 4.40353E-42 | down |
| MELO3C015418.2 | 10 | 164 | 4.017 | 1.37284E-24 | up |
| MELO3C015421.2 | 9359 | 1712 | -2.45 | 3.04466E-187 | down |
| MELO3C015423.2 | 401 | 24 | -4.038 | 3.08505E-52 | down |
| MELO3C015427.2 | 34 | 3 | -3.272 | 2.54625E-06 | down |
| MELO3C015430.2 | 27 | 4 | -2.592 | 0.000126645 | down |
| MELO3C015431.2 | 2239 | 4638 | 1.05 | 2.62808E-35 | up |
| MELO3C015434.2 | 11 | 29 | 1.439 | 0.013801333 | up |
| MELO3C015440.2 | 1806 | 899 | -1.006 | 5.41932E-20 | down |
| MELO3C015443.2 | 812 | 1722 | 1.085 | 1.73893E-28 | up |
| MELO3C015446.2 | 4153 | 1494 | -1.476 | 1.53458E-35 | down |
| MELO3C015450.2 | 773 | 1900 | 1.298 | 4.33406E-35 | up |
| MELO3C015451.2 | 261 | 539 | 1.045 | 7.30712E-12 | up |
| MELO3C015457.2 | 217 | 521 | 1.264 | 1.21832E-19 | up |
| MELO3C015467.2 | 56 | 121 | 1.107 | 0.000407279 | up |
| MELO3C015469.2 | 2019 | 455 | -2.147 | 4.26473E-79 | down |
| MELO3C015470.2 | 11202 | 185 | -5.92 | 0 | down |
| MELO3C015475.2 | 165 | 592 | 1.849 | 6.76453E-18 | up |
| MELO3C015480.2 | 161 | 968 | 2.593 | 3.54553E-52 | up |
| MELO3C015483.2 | 1225 | 2667 | 1.121 | 6.08884E-22 | up |
| MELO3C015494.2 | 5369 | 1712 | -1.649 | 2.63519E-45 | down |
| MELO3C015495.2 | 11 | 40 | 1.979 | 0.000243937 | up |
| MELO3C015506.2 | 245 | 45 | -2.459 | 2.84953E-25 | down |
| MELO3C015507.2 | 0 | 14 | 6.036 | 6.50492E-05 | up |
| MELO3C015509.2 | 610 | 17 | -5.182 | 2.0582E-106 | down |
| MELO3C015513.2 | 75 | 305 | 2.024 | 4.01448E-17 | up |
| MELO3C015515.2 | 5 | 19 | 2.081 | 0.009666705 | up |
| MELO3C015526.2 | 126 | 2833 | 4.496 | 6.66582E-28 | up |
| MELO3C015527.2 | 138 | 639 | 2.214 | 1.9419E-36 | up |
| MELO3C015533.2 | 30 | 3 | -3.367 | 1.48429E-06 | down |
| MELO3C015536.2 | 3553 | 230 | -3.953 | 6.62058E-170 | down |
| MELO3C015538.2 | 1478 | 102 | -3.846 | 8.30284E-100 | down |
| MELO3C015544.2 | 17593 | 6330 | -1.475 | 8.57048E-92 | down |
| MELO3C015549.2 | 22 | 1 | -4.618 | 6.22904E-05 | down |
| MELO3C015550.2 | 15 | 1 | -4.592 | 0.001875103 | down |
| MELO3C015551.2 | 0 | 9 | 5.396 | 0.000944977 | up |
| MELO3C015552.2 | 3241 | 995 | -1.703 | 1.19996E-38 | down |
| MELO3C015556.2 | 4130 | 1339 | -1.626 | 7.58225E-51 | down |
| MELO3C015564.2 | 2196 | 1080 | -1.023 | 1.7942E-26 | down |
| MELO3C015567.2 | 1587 | 382 | -2.054 | 1.62958E-74 | down |
| MELO3C015568.2 | 33997 | 11097 | -1.615 | 3.9158E-101 | down |
| MELO3C015570.2 | 23 | 338 | 3.893 | 2.57374E-45 | up |
| MELO3C015571.2 | 22 | 2 | -3.638 | 6.20208E-05 | down |
| MELO3C015575.2 | 467 | 1299 | 1.475 | 2.7656E-26 | up |
| MELO3C015579.2 | 47 | 97 | 1.05 | 0.004441563 | up |
| MELO3C015587.2 | 15 | 2 | -2.857 | 0.004210217 | down |
| MELO3C015590.2 | 91 | 238 | 1.388 | 5.47825E-12 | up |
| MELO3C015593.2 | 889 | 5162 | 2.539 | 1.69038E-107 | up |
| MELO3C015595.2 | 2569 | 938 | -1.454 | 2.7716E-47 | down |
| MELO3C015598.2 | 170 | 353 | 1.06 | 1.80079E-09 | up |
| MELO3C015606.2 | 1101 | 360 | -1.613 | 9.01361E-48 | down |
| MELO3C015618.2 | 87 | 188 | 1.103 | 3.30006E-06 | up |
| MELO3C015622.2 | 81 | 366 | 2.189 | 2.1786E-27 | up |
| MELO3C015640.2 | 17 | 53 | 1.66 | 0.00260806 | up |
| MELO3C015648.2 | 1911 | 739 | -1.373 | 5.23653E-40 | down |
| MELO3C015651.2 | 30 | 3 | -3.5 | 1.97948E-06 | down |
| MELO3C015653.2 | 8415 | 3302 | -1.349 | 6.93789E-47 | down |
| MELO3C015661.2 | 3601 | 7331 | 1.026 | 3.131E-15 | up |
| MELO3C015675.2 | 5982 | 2823 | -1.083 | 2.69807E-50 | down |
| MELO3C015677.2 | 277 | 128 | -1.115 | 1.87542E-08 | down |
| MELO3C015678.2 | 485 | 203 | -1.255 | 1.46965E-13 | down |
| MELO3C015685.2 | 514 | 222 | -1.214 | 2.91848E-17 | down |
| MELO3C015687.2 | 6 | 0 | -5.176 | 0.004605545 | down |
| MELO3C015691.2 | 34 | 1 | -5.751 | 0.009085027 | down |
| MELO3C015693.2 | 4049 | 1500 | -1.432 | 2.66209E-82 | down |
| MELO3C015705.2 | 237 | 552 | 1.22 | 1.12349E-17 | up |
| MELO3C015706.2 | 4795 | 1805 | -1.409 | 1.1825E-52 | down |
| MELO3C015715.2 | 4 | 15 | 1.781 | 0.030825526 | up |
| MELO3C015718.2 | 2564 | 1085 | -1.241 | 3.96561E-36 | down |
| MELO3C015722.2 | 2355 | 6969 | 1.565 | 9.29686E-88 | up |
| MELO3C015726.2 | 25 | 148 | 2.589 | 1.69653E-09 | up |
| MELO3C015727.2 | 123 | 371 | 1.595 | 4.39439E-13 | up |
| MELO3C015738.2 | 76 | 14 | -2.459 | 5.66843E-08 | down |
| MELO3C015740.2 | 224 | 473 | 1.081 | 3.71883E-13 | up |
| MELO3C015743.2 | 119 | 3622 | 4.926 | 6.61024E-30 | up |
| MELO3C015744.2 | 24998 | 3 | -13.045 | 4.60947E-135 | down |
| MELO3C015746.2 | 0 | 11 | 5.712 | 0.000494903 | up |
| MELO3C015762.2 | 49 | 5 | -3.252 | 3.80553E-06 | down |
| MELO3C015764.2 | 24 | 172 | 2.847 | 1.66053E-23 | up |
| MELO3C015772.2 | 20279 | 1157 | -4.131 | 0 | down |
| MELO3C015789.2 | 3872 | 549 | -2.816 | 5.6583E-264 | down |
| MELO3C015791.2 | 163 | 75 | -1.104 | 1.12083E-05 | down |
| MELO3C015792.2 | 708 | 54 | -3.739 | 5.41395E-71 | down |
| MELO3C015795.2 | 1274 | 253 | -2.329 | 1.71765E-64 | down |
| MELO3C015796.2 | 2549 | 494 | -2.365 | 3.59429E-116 | down |
| MELO3C015799.2 | 221 | 887 | 2.007 | 2.46458E-48 | up |
| MELO3C015801.2 | 481 | 1308 | 1.441 | 9.09835E-34 | up |
| MELO3C015811.2 | 1658 | 746 | -1.153 | 1.14222E-27 | down |
| MELO3C015813.2 | 4 | 68 | 4.055 | 7.372E-12 | up |
| MELO3C015815.2 | 4 | 17 | 2.214 | 0.009508215 | up |
| MELO3C015818.2 | 0 | 8 | 5.362 | 0.00113018 | up |
| MELO3C015828.2 | 699 | 1499 | 1.1 | 1.76658E-27 | up |
| MELO3C015831.2 | 22 | 54 | 1.303 | 0.006240514 | up |
| MELO3C015842.2 | 7 | 0 | -5.363 | 0.003690788 | down |
| MELO3C015848.2 | 32 | 3 | -3.304 | 3.89772E-05 | down |
| MELO3C015850.2 | 706 | 174 | -2.023 | 2.55377E-51 | down |
| MELO3C015852.2 | 443 | 2485 | 2.487 | 2.78325E-81 | up |
| MELO3C015865.2 | 0 | 19 | 6.537 | 4.92159E-06 | up |
| MELO3C015868.2 | 1325 | 300 | -2.139 | 1.77296E-23 | down |
| MELO3C015872.2 | 1903 | 663 | -1.523 | 1.4228E-53 | down |
| MELO3C015875.2 | 159 | 360 | 1.181 | 7.45625E-11 | up |
| MELO3C015881.2 | 1 | 16 | 3.442 | 0.002173925 | up |
| MELO3C015882.2 | 19 | 343 | 4.176 | 1.68627E-05 | up |
| MELO3C015894.2 | 69 | 11 | -2.635 | 1.20445E-08 | down |
| MELO3C015895.2 | 2100 | 687 | -1.611 | 6.36868E-37 | down |
| MELO3C015901.2 | 263 | 653 | 1.316 | 3.87027E-14 | up |
| MELO3C015905.2 | 975 | 403 | -1.275 | 1.44226E-25 | down |
| MELO3C015908.2 | 23 | 1 | -5.26 | 8.51286E-05 | down |
| MELO3C015909.2 | 553 | 2424 | 2.13 | 3.47806E-70 | up |
| MELO3C015910.2 | 133 | 37 | -1.84 | 9.28917E-11 | down |
| MELO3C015913.2 | 11244 | 4900 | -1.198 | 1.11435E-27 | down |
| MELO3C015922.2 | 1053 | 2197 | 1.06 | 6.38527E-18 | up |
| MELO3C015924.2 | 12847 | 6268 | -1.036 | 2.69105E-35 | down |
| MELO3C015926.2 | 37 | 396 | 3.425 | 7.50584E-24 | up |
| MELO3C015930.2 | 97 | 17 | -2.495 | 5.41562E-07 | down |
| MELO3C015931.2 | 7 | 0 | -5.411 | 0.003338739 | down |
| MELO3C015932.2 | 1 | 7 | 3.275 | 0.032340456 | up |
| MELO3C015934.2 | 23 | 113 | 2.327 | 2.9256E-11 | up |
| MELO3C015942.2 | 1 | 15 | 3.292 | 0.004573205 | up |
| MELO3C015949.2 | 253 | 592 | 1.225 | 1.14968E-15 | up |
| MELO3C015965.2 | 83 | 28 | -1.58 | 1.54592E-05 | down |
| MELO3C015984.2 | 225 | 74 | -1.611 | 9.5775E-15 | down |
| MELO3C015985.2 | 297 | 124 | -1.258 | 1.73425E-13 | down |
| MELO3C015995.2 | 23974 | 6010 | -1.996 | 5.62354E-57 | down |
| MELO3C015998.2 | 3650 | 1359 | -1.425 | 1.50021E-51 | down |
| MELO3C016004.2 | 4 | 19 | 2.194 | 0.011668117 | up |
| MELO3C016006.2 | 57 | 183 | 1.678 | 2.17105E-11 | up |
| MELO3C016007.2 | 6131 | 853 | -2.845 | 1.61971E-193 | down |
| MELO3C016008.2 | 107 | 318 | 1.575 | 3.85005E-14 | up |
| MELO3C016014.2 | 272 | 549 | 1.013 | 5.919E-13 | up |
| MELO3C016017.2 | 278 | 35 | -3.003 | 4.24633E-31 | down |
| MELO3C016019.2 | 177 | 366 | 1.051 | 2.66738E-09 | up |
| MELO3C016022.2 | 0 | 22 | 6.788 | 6.37991E-07 | up |
| MELO3C016031.2 | 3532 | 285 | -3.63 | 2.92351E-163 | down |
| MELO3C016033.2 | 1797 | 433 | -2.05 | 2.60713E-33 | down |
| MELO3C016049.2 | 1837 | 460 | -1.999 | 1.25702E-70 | down |
| MELO3C016055.2 | 95 | 2 | -5.529 | 3.48337E-17 | down |
| MELO3C016056.2 | 90 | 190 | 1.071 | 0.000188609 | up |
| MELO3C016080.2 | 29 | 94 | 1.709 | 3.60043E-07 | up |
| MELO3C016082.2 | 358 | 1262 | 1.815 | 2.4821E-05 | up |
| MELO3C016089.2 | 3133 | 1408 | -1.153 | 1.34625E-31 | down |
| MELO3C016092.2 | 229 | 459 | 1.006 | 3.08382E-05 | up |
| MELO3C016110.2 | 11 | 2 | -2.594 | 0.02034558 | down |
| MELO3C016114.2 | 82 | 327 | 1.985 | 1.38275E-23 | up |
| MELO3C016116.2 | 15 | 61 | 1.996 | 1.96806E-06 | up |
| MELO3C016119.2 | 832 | 1730 | 1.057 | 5.35822E-28 | up |
| MELO3C016124.2 | 1501 | 709 | -1.084 | 1.34649E-29 | down |
| MELO3C016126.2 | 1088 | 2269 | 1.06 | 7.03111E-20 | up |
| MELO3C016131.2 | 4159 | 1299 | -1.679 | 1.55515E-91 | down |
| MELO3C016140.2 | 4630 | 2131 | -1.12 | 5.53239E-16 | down |
| MELO3C016141.2 | 4 | 53 | 3.565 | 9.86263E-09 | up |
| MELO3C016152.2 | 54 | 7 | -3.036 | 1.37863E-06 | down |
| MELO3C016164.2 | 462 | 1326 | 1.525 | 1.0358E-34 | up |
| MELO3C016167.2 | 27443 | 804 | -5.094 | 0 | down |
| MELO3C016168.2 | 81 | 212 | 1.386 | 9.98119E-07 | up |
| MELO3C016172.2 | 61 | 201 | 1.718 | 8.26801E-11 | up |
| MELO3C016176.2 | 147 | 14 | -3.415 | 3.0028E-26 | down |
| MELO3C016177.2 | 1913 | 520 | -1.879 | 7.74137E-80 | down |
| MELO3C016181.2 | 27201 | 4469 | -2.606 | 4.64632E-244 | down |
| MELO3C016190.2 | 35300 | 3813 | -3.211 | 2.40949E-270 | down |
| MELO3C016193.2 | 6289 | 13956 | 1.15 | 1.01153E-17 | up |
| MELO3C016197.2 | 31 | 165 | 2.393 | 1.28926E-12 | up |
| MELO3C016208.2 | 143 | 17 | -3.082 | 3.91606E-12 | down |
| MELO3C016210.2 | 809 | 214 | -1.917 | 4.66458E-26 | down |
| MELO3C016214.2 | 0 | 8 | 5.207 | 0.001899694 | up |
| MELO3C016218.2 | 107 | 378 | 1.829 | 1.21586E-21 | up |
| MELO3C016221.2 | 806 | 302 | -1.416 | 0.005219535 | down |
| MELO3C016224.2 | 67390 | 15861 | -2.087 | 6.02352E-119 | down |
| MELO3C016226.2 | 9247 | 2274 | -2.024 | 9.58274E-107 | down |
| MELO3C016233.2 | 3087 | 1263 | -1.29 | 6.62724E-52 | down |
| MELO3C016236.2 | 546 | 134 | -2.026 | 8.93233E-42 | down |
| MELO3C016247.2 | 77 | 184 | 1.251 | 4.4838E-07 | up |
| MELO3C016252.2 | 1014 | 2067 | 1.028 | 9.16059E-30 | up |
| MELO3C016259.2 | 3784 | 180 | -4.389 | 4.7164E-143 | down |
| MELO3C016260.2 | 6279 | 2675 | -1.231 | 1.96081E-59 | down |
| MELO3C016263.2 | 44 | 9 | -2.374 | 4.27036E-06 | down |
| MELO3C016267.2 | 3 | 61 | 4.194 | 4.86146E-11 | up |
| MELO3C016268.2 | 35 | 6 | -2.469 | 1.42865E-05 | down |
| MELO3C016270.2 | 383 | 2 | -7.279 | 2.91973E-06 | down |
| MELO3C016271.2 | 131 | 8 | -4.053 | 3.43887E-21 | down |
| MELO3C016287.2 | 167 | 2305 | 3.787 | 2.12155E-09 | up |
| MELO3C016288.2 | 475 | 1108 | 1.224 | 9.58398E-25 | up |
| MELO3C016294.2 | 29 | 152 | 2.389 | 1.4296E-12 | up |
| MELO3C016296.2 | 7312 | 949 | -2.945 | 3.62249E-230 | down |
| MELO3C016297.2 | 920 | 401 | -1.198 | 2.869E-20 | down |
| MELO3C016300.2 | 5990 | 2649 | -1.177 | 2.47019E-27 | down |
| MELO3C016304.2 | 27 | 6 | -2.254 | 0.001328836 | down |
| MELO3C016308.2 | 7721 | 2684 | -1.525 | 8.72702E-86 | down |
| MELO3C016310.2 | 221 | 474 | 1.101 | 1.78134E-12 | up |
| MELO3C016322.2 | 10 | 38 | 1.899 | 0.012253395 | up |
| MELO3C016325.2 | 92 | 0 | -9.137 | 2.26375E-06 | down |
| MELO3C016331.2 | 3 | 122 | 5.542 | 1.74199E-18 | up |
| MELO3C016332.2 | 3 | 320 | 6.749 | 2.77071E-33 | up |
| MELO3C016337.2 | 8 | 0 | -5.678 | 0.004672178 | down |
| MELO3C016340.2 | 353 | 5 | -6.318 | 4.47604E-46 | down |
| MELO3C016342.2 | 280 | 1143 | 2.031 | 6.99567E-53 | up |
| MELO3C016346.2 | 96 | 1763 | 4.201 | 4.55183E-221 | up |
| MELO3C016347.2 | 82 | 261 | 1.677 | 1.69037E-17 | up |
| MELO3C016348.2 | 2235 | 5559 | 1.315 | 1.48451E-47 | up |
| MELO3C016349.2 | 4057 | 1790 | -1.179 | 1.14065E-49 | down |
| MELO3C016351.2 | 4386 | 1064 | -2.044 | 2.67812E-118 | down |
| MELO3C016354.2 | 3127 | 368 | -3.086 | 2.82848E-196 | down |
| MELO3C016358.2 | 18 | 1 | -4.948 | 0.000158701 | down |
| MELO3C016360.2 | 6 | 21 | 1.806 | 0.011655347 | up |
| MELO3C016366.2 | 33168 | 7421 | -2.16 | 1.19182E-252 | down |
| MELO3C016368.2 | 2644 | 1250 | -1.08 | 3.14673E-35 | down |
| MELO3C016374.2 | 1966 | 912 | -1.108 | 3.86218E-23 | down |
| MELO3C016383.2 | 1529 | 524 | -1.546 | 2.81875E-58 | down |
| MELO3C016384.2 | 181 | 13 | -3.775 | 1.99374E-32 | down |
| MELO3C016385.2 | 49 | 0 | -8.231 | 1.09965E-10 | down |
| MELO3C016389.2 | 46 | 94 | 1.055 | 0.000382441 | up |
| MELO3C016390.2 | 134 | 275 | 1.034 | 3.3585E-07 | up |
| MELO3C016396.2 | 52 | 16 | -1.722 | 0.00028178 | down |
| MELO3C016397.2 | 1505 | 703 | -1.099 | 6.02053E-20 | down |
| MELO3C016398.2 | 369 | 827 | 1.165 | 2.48E-06 | up |
| MELO3C016402.2 | 77 | 1 | -7.002 | 6.56423E-09 | down |
| MELO3C016410.2 | 324 | 124 | -1.388 | 2.77445E-12 | down |
| MELO3C016419.2 | 608 | 1551 | 1.351 | 3.31106E-21 | up |
| MELO3C016420.2 | 925 | 437 | -1.08 | 5.64676E-21 | down |
| MELO3C016434.2 | 147 | 504 | 1.78 | 8.55436E-20 | up |
| MELO3C016444.2 | 831 | 372 | -1.161 | 3.03745E-09 | down |
| MELO3C016445.2 | 0 | 66 | 8.323 | 2.334E-11 | up |
| MELO3C016451.2 | 27 | 92 | 1.779 | 1.77901E-07 | up |
| MELO3C016459.2 | 103 | 883 | 3.097 | 1.01168E-88 | up |
| MELO3C016465.2 | 99 | 1 | -7.378 | 8.61273E-12 | down |
| MELO3C016476.2 | 12 | 59 | 2.346 | 1.51957E-07 | up |
| MELO3C016482.2 | 122 | 303 | 1.309 | 6.77479E-11 | up |
| MELO3C016483.2 | 3543 | 1624 | -1.126 | 2.45657E-48 | down |
| MELO3C016491.2 | 515 | 1278 | 1.311 | 3.168E-21 | up |
| MELO3C016494.2 | 1579 | 3 | -9.387 | 3.48783E-72 | down |
| MELO3C016495.2 | 2120 | 998 | -1.087 | 4.67325E-26 | down |
| MELO3C016502.2 | 385 | 905 | 1.232 | 1.50567E-15 | up |
| MELO3C016505.2 | 3103 | 1228 | -1.338 | 2.09874E-46 | down |
| MELO3C016522.2 | 67 | 3 | -4.391 | 1.37503E-13 | down |
| MELO3C016525.2 | 870 | 3286 | 1.917 | 8.96449E-76 | up |
| MELO3C016527.2 | 462 | 133 | -1.796 | 3.90316E-14 | down |
| MELO3C016536.2 | 3746 | 35 | -6.717 | 0 | down |
| MELO3C016539.2 | 21 | 0 | -7.022 | 1.38129E-06 | down |
| MELO3C016542.2 | 916 | 394 | -1.221 | 6.99263E-18 | down |
| MELO3C016551.2 | 9 | 0 | -5.708 | 0.000531727 | down |
| MELO3C016552.2 | 37 | 15 | -1.327 | 0.005211897 | down |
| MELO3C016554.2 | 437 | 1001 | 1.194 | 8.01655E-24 | up |
| MELO3C016556.2 | 65373 | 651 | -6.652 | 0 | down |
| MELO3C016579.2 | 201 | 494 | 1.299 | 2.44386E-18 | up |
| MELO3C016584.2 | 40 | 89 | 1.136 | 0.000697901 | up |
| MELO3C016586.2 | 6 | 0 | -5.196 | 0.005099958 | down |
| MELO3C016588.2 | 10 | 1 | -3.562 | 0.008452295 | down |
| MELO3C016590.2 | 13 | 1 | -4.522 | 0.000850283 | down |
| MELO3C016593.2 | 438 | 17 | -4.658 | 6.50134E-72 | down |
| MELO3C016595.2 | 5261 | 1 | -13.125 | 3.16853E-37 | down |
| MELO3C016602.2 | 288 | 142 | -1.022 | 9.40975E-08 | down |
| MELO3C016608.2 | 727 | 154 | -2.239 | 1.04805E-35 | down |
| MELO3C016619.2 | 188 | 33 | -2.479 | 4.56256E-08 | down |
| MELO3C016623.2 | 463 | 1266 | 1.451 | 2.35924E-26 | up |
| MELO3C016627.2 | 281 | 74 | -1.922 | 1.06003E-16 | down |
| MELO3C016628.2 | 249 | 9 | -4.703 | 8.09424E-38 | down |
| MELO3C016636.2 | 5 | 0 | -4.934 | 0.012301299 | down |
| MELO3C016650.2 | 34 | 370 | 3.474 | 1.79011E-45 | up |
| MELO3C016656.2 | 749 | 336 | -1.158 | 1.28685E-15 | down |
| MELO3C016659.2 | 13 | 38 | 1.628 | 0.000877692 | up |
| MELO3C016660.2 | 1739 | 4795 | 1.463 | 9.4958E-58 | up |
| MELO3C016661.2 | 177 | 24 | -2.903 | 9.31172E-23 | down |
| MELO3C016668.2 | 252 | 121 | -1.054 | 4.31763E-06 | down |
| MELO3C016674.2 | 335 | 81 | -2.051 | 8.04041E-21 | down |
| MELO3C016680.2 | 42 | 17 | -1.32 | 0.003661367 | down |
| MELO3C016683.2 | 54 | 11 | -2.259 | 1.36284E-05 | down |
| MELO3C016684.2 | 36 | 5 | -2.931 | 1.0707E-05 | down |
| MELO3C016685.2 | 1547 | 463 | -1.741 | 8.45587E-30 | down |
| MELO3C016686.2 | 323 | 1006 | 1.64 | 7.81921E-26 | up |
| MELO3C016695.2 | 222 | 60 | -1.869 | 1.47986E-10 | down |
| MELO3C016703.2 | 214 | 585 | 1.452 | 7.91877E-26 | up |
| MELO3C016704.2 | 69 | 12 | -2.572 | 1.21971E-09 | down |
| MELO3C016712.2 | 5774 | 450 | -3.683 | 9.80999E-173 | down |
| MELO3C016714.2 | 53135 | 383 | -7.12 | 0 | down |
| MELO3C016717.2 | 1849 | 5747 | 1.637 | 3.22659E-31 | up |
| MELO3C016719.2 | 3396 | 229 | -3.891 | 1.67564E-102 | down |
| MELO3C016729.2 | 521 | 1134 | 1.123 | 1.08578E-20 | up |
| MELO3C016732.2 | 278 | 938 | 1.756 | 1.35105E-42 | up |
| MELO3C016733.2 | 1646 | 10250 | 2.638 | 7.97182E-189 | up |
| MELO3C016739.2 | 18 | 0 | -6.804 | 1.362E-06 | down |
| MELO3C016742.2 | 31704 | 15015 | -1.078 | 2.67154E-52 | down |
| MELO3C016745.2 | 1387 | 93 | -3.894 | 2.48574E-131 | down |
| MELO3C016746.2 | 3090 | 787 | -1.972 | 2.15401E-26 | down |
| MELO3C016754.2 | 234 | 590 | 1.33 | 1.04761E-15 | up |
| MELO3C016759.2 | 20 | 170 | 3.069 | 2.41396E-23 | up |
| MELO3C016762.2 | 15 | 2 | -3.086 | 0.006912539 | down |
| MELO3C016765.2 | 15 | 49 | 1.741 | 0.001022448 | up |
| MELO3C016768.2 | 4 | 24 | 2.708 | 0.000224137 | up |
| MELO3C016769.2 | 109 | 3 | -4.961 | 2.28363E-18 | down |
| MELO3C016770.2 | 20 | 2 | -3.54 | 0.000134581 | down |
| MELO3C016771.2 | 10675 | 1991 | -2.423 | 4.43152E-06 | down |
| MELO3C016772.2 | 26 | 8 | -1.774 | 0.002196065 | down |
| MELO3C016773.2 | 169 | 60 | -1.491 | 1.39645E-08 | down |
| MELO3C016777.2 | 2198 | 7776 | 1.823 | 2.7611E-52 | up |
| MELO3C016779.2 | 1271 | 337 | -1.914 | 3.0622E-50 | down |
| MELO3C016780.2 | 537 | 218 | -1.305 | 6.54376E-17 | down |
| MELO3C016782.2 | 216 | 465 | 1.107 | 1.13624E-15 | up |
| MELO3C016783.2 | 7 | 28 | 2.13 | 0.002111551 | up |
| MELO3C016787.2 | 42 | 236 | 2.473 | 5.51077E-18 | up |
| MELO3C016794.2 | 1083 | 164 | -2.723 | 2.83422E-05 | down |
| MELO3C016806.2 | 1 | 12 | 3.008 | 0.008240041 | up |
| MELO3C016807.2 | 78 | 21 | -1.865 | 4.07625E-05 | down |
| MELO3C016808.2 | 278 | 54 | -2.354 | 1.58364E-15 | down |
| MELO3C016809.2 | 1255 | 410 | -1.614 | 2.31338E-18 | down |
| MELO3C016824.2 | 1 | 30 | 5.341 | 5.73748E-06 | up |
| MELO3C016826.2 | 3 | 19 | 2.664 | 0.0037209 | up |
| MELO3C016828.2 | 1675 | 382 | -2.134 | 3.89692E-25 | down |
| MELO3C016829.2 | 2422 | 583 | -2.055 | 0.001743665 | down |
| MELO3C016830.2 | 268 | 1018 | 1.923 | 3.67551E-23 | up |
| MELO3C016835.2 | 17 | 1 | -4.894 | 0.000144528 | down |
| MELO3C016836.2 | 36 | 1 | -5.945 | 3.32881E-07 | down |
| MELO3C016841.2 | 161 | 26 | -2.638 | 1.17756E-12 | down |
| MELO3C016842.2 | 476 | 1824 | 1.94 | 1.80461E-33 | up |
| MELO3C016847.2 | 51 | 16 | -1.715 | 9.39704E-05 | down |
| MELO3C016848.2 | 30 | 799 | 4.742 | 1.13471E-87 | up |
| MELO3C016849.2 | 21 | 482 | 4.55 | 5.48526E-67 | up |
| MELO3C016850.2 | 214 | 2 | -7.181 | 8.63053E-26 | down |
| MELO3C016857.2 | 41 | 1 | -5.139 | 1.08865E-08 | down |
| MELO3C016860.2 | 70 | 392 | 2.473 | 1.73886E-39 | up |
| MELO3C016864.2 | 48 | 13 | -1.96 | 3.63986E-05 | down |
| MELO3C016867.2 | 913 | 339 | -1.435 | 3.02484E-30 | down |
| MELO3C016871.2 | 808 | 275 | -1.552 | 5.40486E-25 | down |
| MELO3C016872.2 | 6 | 404 | 6.172 | 1.08122E-49 | up |
| MELO3C016873.2 | 5495 | 2221 | -1.307 | 4.88815E-41 | down |
| MELO3C016878.2 | 1870 | 4131 | 1.143 | 1.84973E-28 | up |
| MELO3C016879.2 | 5 | 35 | 2.852 | 1.30986E-05 | up |
| MELO3C016882.2 | 414 | 1352 | 1.708 | 2.2648E-49 | up |
| MELO3C016883.2 | 152 | 723 | 2.249 | 6.44814E-37 | up |
| MELO3C016897.2 | 5315 | 1445 | -1.879 | 1.97005E-57 | down |
| MELO3C016901.2 | 155 | 31 | -2.327 | 2.01703E-15 | down |
| MELO3C016905.2 | 108 | 14 | -2.941 | 7.64212E-16 | down |
| MELO3C016910.2 | 1176 | 305 | -1.948 | 1.8243E-34 | down |
| MELO3C016922.2 | 28 | 4 | -2.86 | 2.70156E-05 | down |
| MELO3C016926.2 | 20 | 107 | 2.424 | 3.73665E-10 | up |
| MELO3C016932.2 | 1713 | 201 | -3.092 | 6.23111E-51 | down |
| MELO3C016935.2 | 1178 | 512 | -1.204 | 1.41579E-23 | down |
| MELO3C016938.2 | 110 | 422 | 1.949 | 2.27868E-21 | up |
| MELO3C016940.2 | 100 | 260 | 1.373 | 3.01964E-11 | up |
| MELO3C016941.2 | 11 | 2 | -2.949 | 0.008195875 | down |
| MELO3C016942.2 | 4295 | 9867 | 1.2 | 2.48611E-70 | up |
| MELO3C016951.2 | 9424 | 3302 | -1.513 | 1.26926E-49 | down |
| MELO3C016962.2 | 243 | 18 | -3.694 | 1.79126E-40 | down |
| MELO3C016964.2 | 29 | 5 | -2.631 | 5.26565E-05 | down |
| MELO3C016976.2 | 10 | 0 | -5.885 | 0.000221118 | down |
| MELO3C016977.2 | 58 | 180 | 1.626 | 1.29451E-10 | up |
| MELO3C016978.2 | 756 | 340 | -1.157 | 5.85089E-16 | down |
| MELO3C016999.2 | 1 | 9 | 3.613 | 0.014251266 | up |
| MELO3C017005.2 | 41 | 160 | 1.966 | 3.3188E-10 | up |
| MELO3C017011.2 | 24423 | 7985 | -1.613 | 6.46208E-30 | down |
| MELO3C017012.2 | 19 | 42 | 1.142 | 0.009913008 | up |
| MELO3C017017.2 | 2298 | 726 | -1.661 | 2.48102E-48 | down |
| MELO3C017023.2 | 2015 | 248 | -3.019 | 2.56876E-98 | down |
| MELO3C017027.2 | 1185 | 3502 | 1.563 | 2.00592E-23 | up |
| MELO3C017032.2 | 68 | 138 | 1.009 | 8.50014E-05 | up |
| MELO3C017034.2 | 15 | 163 | 3.449 | 1.61467E-25 | up |
| MELO3C017041.2 | 188 | 478 | 1.344 | 4.72336E-20 | up |
| MELO3C017044.2 | 33460 | 14303 | -1.226 | 4.6521E-33 | down |
| MELO3C017047.2 | 2561 | 845 | -1.601 | 8.72108E-65 | down |
| MELO3C017048.2 | 1125 | 555 | -1.018 | 1.25965E-18 | down |
| MELO3C017052.2 | 20 | 57 | 1.533 | 0.000931695 | up |
| MELO3C017053.2 | 1 | 8 | 3.371 | 0.024594314 | up |
| MELO3C017055.2 | 56 | 26 | -1.097 | 0.008372708 | down |
| MELO3C017057.2 | 355 | 741 | 1.061 | 1.02464E-11 | up |
| MELO3C017059.2 | 14 | 66 | 2.266 | 7.75524E-08 | up |
| MELO3C017060.2 | 513 | 1111 | 1.114 | 7.10631E-13 | up |
| MELO3C017068.2 | 789 | 6957 | 3.142 | 3.3302E-166 | up |
| MELO3C017072.2 | 18 | 3 | -2.663 | 0.016469383 | down |
| MELO3C017087.2 | 3719 | 1804 | -1.044 | 1.42198E-28 | down |
| MELO3C017090.2 | 58 | 172 | 1.549 | 5.92073E-09 | up |
| MELO3C017099.2 | 1052 | 522 | -1.014 | 2.72098E-10 | down |
| MELO3C017100.2 | 4447 | 11732 | 1.4 | 1.148E-28 | up |
| MELO3C017104.2 | 3998 | 1090 | -1.876 | 7.25859E-46 | down |
| MELO3C017109.2 | 2748 | 1334 | -1.043 | 1.21117E-12 | down |
| MELO3C017113.2 | 760 | 280 | -1.437 | 4.71292E-15 | down |
| MELO3C017121.2 | 885 | 426 | -1.054 | 3.02661E-14 | down |
| MELO3C017124.2 | 60 | 11 | -2.444 | 2.30501E-06 | down |
| MELO3C017126.2 | 1445 | 3621 | 1.325 | 2.83774E-54 | up |
| MELO3C017130.2 | 490 | 222 | -1.141 | 9.20675E-15 | down |
| MELO3C017131.2 | 3302 | 1405 | -1.233 | 1.02151E-49 | down |
| MELO3C017141.2 | 142 | 37 | -1.941 | 1.223E-11 | down |
| MELO3C017147.2 | 36 | 158 | 2.117 | 2.84239E-13 | up |
| MELO3C017150.2 | 4776 | 2169 | -1.139 | 8.79492E-19 | down |
| MELO3C017154.2 | 482 | 35 | -3.779 | 6.34216E-11 | down |
| MELO3C017156.2 | 1968 | 500 | -1.977 | 7.85119E-42 | down |
| MELO3C017158.2 | 15 | 167 | 3.481 | 8.67595E-18 | up |
| MELO3C017161.2 | 0 | 19 | 6.547 | 5.64923E-05 | up |
| MELO3C017166.2 | 1698 | 797 | -1.091 | 3.3939E-19 | down |
| MELO3C017168.2 | 78 | 1 | -7.052 | 1.87827E-10 | down |
| MELO3C017170.2 | 0 | 4 | 4.349 | 0.028719577 | up |
| MELO3C017175.2 | 487 | 2091 | 2.101 | 2.32335E-112 | up |
| MELO3C017176.2 | 19182 | 5420 | -1.823 | 6.87363E-86 | down |
| MELO3C017177.2 | 63 | 7 | -3.214 | 0.000279531 | down |
| MELO3C017180.2 | 50692 | 175 | -8.177 | 0 | down |
| MELO3C017182.2 | 1022 | 214 | -2.262 | 1.97639E-42 | down |
| MELO3C017197.2 | 271 | 1188 | 2.134 | 7.16033E-74 | up |
| MELO3C017198.2 | 38 | 132 | 1.8 | 1.84593E-10 | up |
| MELO3C017201.2 | 9 | 39 | 2.181 | 0.000142956 | up |
| MELO3C017206.2 | 903 | 2174 | 1.268 | 3.31445E-22 | up |
| MELO3C017218.2 | 9 | 53 | 2.533 | 0.000202325 | up |
| MELO3C017219.2 | 2241 | 12643 | 2.496 | 2.76036E-07 | up |
| MELO3C017220.2 | 126 | 344 | 1.447 | 1.09143E-10 | up |
| MELO3C017228.2 | 18 | 271 | 3.896 | 1.15137E-21 | up |
| MELO3C017231.2 | 798 | 2237 | 1.488 | 3.16468E-62 | up |
| MELO3C017232.2 | 37 | 143 | 1.931 | 6.67261E-10 | up |
| MELO3C017242.2 | 1460 | 9126 | 2.644 | 3.05265E-102 | up |
| MELO3C017243.2 | 8 | 23 | 1.438 | 0.030980212 | up |
| MELO3C017244.2 | 6 | 49 | 3.036 | 3.41347E-08 | up |
| MELO3C017245.2 | 241 | 1124 | 2.221 | 5.65695E-31 | up |
| MELO3C017246.2 | 49 | 133 | 1.439 | 2.55938E-07 | up |
| MELO3C017253.2 | 2589 | 5632 | 1.122 | 2.51467E-25 | up |
| MELO3C017254.2 | 1429 | 711 | -1.007 | 6.28951E-22 | down |
| MELO3C017256.2 | 336 | 1625 | 2.276 | 4.16106E-66 | up |
| MELO3C017258.2 | 40 | 177 | 2.17 | 2.23581E-12 | up |
| MELO3C017261.2 | 1274 | 511 | -1.319 | 2.94196E-16 | down |
| MELO3C017263.2 | 307 | 760 | 1.31 | 1.1103E-20 | up |
| MELO3C017267.2 | 1825 | 555 | -1.717 | 3.47258E-73 | down |
| MELO3C017270.2 | 4905 | 2405 | -1.029 | 2.39262E-32 | down |
| MELO3C017272.2 | 1 | 114 | 6.255 | 1.32325E-15 | up |
| MELO3C017291.2 | 1282 | 3375 | 1.396 | 2.29182E-36 | up |
| MELO3C017293.2 | 463 | 1024 | 1.144 | 6.4101E-21 | up |
| MELO3C017294.2 | 1070 | 1 | -9.506 | 3.3382E-47 | down |
| MELO3C017298.2 | 461 | 1402 | 1.604 | 1.35293E-27 | up |
| MELO3C017299.2 | 18 | 94 | 2.415 | 2.00056E-10 | up |
| MELO3C017302.2 | 939 | 5101 | 2.441 | 5.32847E-58 | up |
| MELO3C017314.2 | 45 | 3 | -3.716 | 2.73836E-08 | down |
| MELO3C017315.2 | 38 | 14 | -1.502 | 0.010728661 | down |
| MELO3C017317.2 | 383 | 838 | 1.13 | 1.75561E-16 | up |
| MELO3C017318.2 | 306 | 867 | 1.501 | 3.5418E-27 | up |
| MELO3C017320.2 | 4795 | 2115 | -1.181 | 1.17962E-26 | down |
| MELO3C017322.2 | 336 | 62 | -2.443 | 4.53682E-26 | down |
| MELO3C017323.2 | 1865 | 703 | -1.406 | 2.93959E-31 | down |
| MELO3C017328.2 | 0 | 18 | 5.448 | 0.000118851 | up |
| MELO3C017332.2 | 2163 | 11135 | 2.364 | 9.34798E-108 | up |
| MELO3C017333.2 | 178 | 78 | -1.184 | 1.83726E-06 | down |
| MELO3C017341.2 | 363 | 1063 | 1.551 | 8.00952E-17 | up |
| MELO3C017345.2 | 8 | 130 | 3.986 | 3.12666E-18 | up |
| MELO3C017348.2 | 223 | 87 | -1.351 | 1.25358E-09 | down |
| MELO3C017352.2 | 42 | 14 | -1.586 | 0.00720747 | down |
| MELO3C017357.2 | 30 | 260 | 3.113 | 3.78164E-14 | up |
| MELO3C017358.2 | 13941 | 4864 | -1.519 | 3.18933E-08 | down |
| MELO3C017374.2 | 1539 | 655 | -1.233 | 2.34225E-23 | down |
| MELO3C017385.2 | 473 | 78 | -2.61 | 7.93664E-44 | down |
| MELO3C017390.2 | 3110 | 1411 | -1.14 | 5.3267E-38 | down |
| MELO3C017391.2 | 162 | 505 | 1.637 | 2.61208E-28 | up |
| MELO3C017398.2 | 134 | 326 | 1.287 | 1.55344E-14 | up |
| MELO3C017410.2 | 183 | 392 | 1.097 | 1.62002E-10 | up |
| MELO3C017415.2 | 366 | 111 | -1.717 | 2.43234E-21 | down |
| MELO3C017420.2 | 17 | 36 | 1.139 | 0.020560081 | up |
| MELO3C017424.2 | 357 | 107 | -1.735 | 2.53201E-19 | down |
| MELO3C017426.2 | 2467 | 876 | -1.493 | 1.01156E-52 | down |
| MELO3C017437.2 | 435 | 79 | -2.465 | 6.79632E-40 | down |
| MELO3C017439.2 | 1035 | 2620 | 1.339 | 2.17694E-43 | up |
| MELO3C017450.2 | 42 | 6 | -2.889 | 1.14616E-06 | down |
| MELO3C017453.2 | 29 | 121 | 2.065 | 1.57549E-07 | up |
| MELO3C017459.2 | 6 | 0 | -5.171 | 0.005298123 | down |
| MELO3C017478.2 | 133 | 403 | 1.609 | 1.34909E-07 | up |
| MELO3C017481.2 | 28 | 1135 | 5.373 | 4.30245E-17 | up |
| MELO3C017482.2 | 3 | 35 | 3.548 | 6.59947E-06 | up |
| MELO3C017485.2 | 1237 | 423 | -1.549 | 1.0702E-27 | down |
| MELO3C017490.2 | 307 | 757 | 1.303 | 9.16926E-19 | up |
| MELO3C017499.2 | 5 | 28 | 2.394 | 0.006425954 | up |
| MELO3C017500.2 | 10615 | 3449 | -1.622 | 3.75281E-76 | down |
| MELO3C017503.2 | 2942 | 193 | -3.926 | 2.1825E-87 | down |
| MELO3C017504.2 | 201 | 533 | 1.402 | 4.69738E-23 | up |
| MELO3C017507.2 | 20 | 5 | -2.046 | 0.005651359 | down |
| MELO3C017511.2 | 801 | 2361 | 1.559 | 6.19109E-26 | up |
| MELO3C017520.2 | 1298 | 30 | -5.44 | 9.98698E-116 | down |
| MELO3C017540.2 | 17 | 5 | -1.758 | 0.022478124 | down |
| MELO3C017541.2 | 15 | 3 | -2.257 | 0.029238559 | down |
| MELO3C017546.2 | 2857 | 1292 | -1.145 | 2.40544E-46 | down |
| MELO3C017548.2 | 622 | 274 | -1.18 | 2.89499E-06 | down |
| MELO3C017554.2 | 109 | 10 | -3.496 | 1.0076E-15 | down |
| MELO3C017557.2 | 15 | 0 | -5.553 | 0.000101538 | down |
| MELO3C017558.2 | 342 | 1028 | 1.589 | 1.99553E-32 | up |
| MELO3C017566.2 | 2460 | 7040 | 1.517 | 1.09695E-43 | up |
| MELO3C017568.2 | 157 | 36 | -2.109 | 1.17247E-13 | down |
| MELO3C017569.2 | 15 | 32 | 1.106 | 0.031743414 | up |
| MELO3C017571.2 | 3476 | 1275 | -1.446 | 1.87683E-27 | down |
| MELO3C017574.2 | 248 | 26 | -3.293 | 1.72347E-26 | down |
| MELO3C017575.2 | 8 | 0 | -5.508 | 0.001147887 | down |
| MELO3C017579.2 | 341 | 884 | 1.377 | 1.21541E-34 | up |
| MELO3C017580.2 | 15 | 105 | 2.853 | 1.91474E-13 | up |
| MELO3C017581.2 | 9393 | 1241 | -2.919 | 9.56648E-16 | down |
| MELO3C017582.2 | 10 | 114 | 3.505 | 8.99582E-14 | up |
| MELO3C017588.2 | 150 | 30 | -2.282 | 3.19315E-09 | down |
| MELO3C017590.2 | 2288 | 688 | -1.735 | 1.11789E-62 | down |
| MELO3C017597.2 | 746 | 1532 | 1.037 | 2.41019E-27 | up |
| MELO3C017603.2 | 836 | 5021 | 2.586 | 3.87079E-64 | up |
| MELO3C017606.2 | 328 | 767 | 1.223 | 2.03835E-16 | up |
| MELO3C017618.2 | 168 | 61 | -1.472 | 2.11251E-07 | down |
| MELO3C017620.2 | 23903 | 3858 | -2.631 | 9.21546E-179 | down |
| MELO3C017623.2 | 32 | 11 | -1.532 | 0.008095456 | down |
| MELO3C017624.2 | 4526 | 1332 | -1.764 | 1.53213E-109 | down |
| MELO3C017630.2 | 142 | 841 | 2.568 | 7.50634E-50 | up |
| MELO3C017632.2 | 7048 | 2376 | -1.569 | 1.60472E-88 | down |
| MELO3C017656.2 | 27 | 267 | 3.317 | 1.03473E-28 | up |
| MELO3C017662.2 | 14 | 38 | 1.397 | 0.005254687 | up |
| MELO3C017672.2 | 414 | 905 | 1.129 | 8.94068E-14 | up |
| MELO3C017674.2 | 3 | 101 | 5.07 | 5.19126E-15 | up |
| MELO3C017675.2 | 302 | 872 | 1.528 | 2.14559E-33 | up |
| MELO3C017677.2 | 4031 | 16296 | 2.015 | 1.52407E-152 | up |
| MELO3C017681.2 | 20 | 4 | -2.501 | 0.006755709 | down |
| MELO3C017687.2 | 18 | 84 | 2.191 | 8.1925E-07 | up |
| MELO3C017691.2 | 402 | 99 | -2.013 | 7.9745E-18 | down |
| MELO3C017701.2 | 146 | 304 | 1.063 | 1.2593E-08 | up |
| MELO3C017708.2 | 83 | 289 | 1.79 | 9.13947E-18 | up |
| MELO3C017716.2 | 196 | 397 | 1.022 | 1.06815E-09 | up |
| MELO3C017721.2 | 1200 | 545 | -1.14 | 1.59034E-27 | down |
| MELO3C017723.2 | 141 | 57 | -1.304 | 2.14741E-07 | down |
| MELO3C017729.2 | 206 | 513 | 1.315 | 3.66897E-17 | up |
| MELO3C017731.2 | 2937 | 1361 | -1.11 | 2.49971E-39 | down |
| MELO3C017733.2 | 8 | 1 | -2.75 | 0.029705643 | down |
| MELO3C017743.2 | 226 | 48 | -2.208 | 1.29819E-10 | down |
| MELO3C017750.2 | 592 | 1324 | 1.16 | 7.85346E-20 | up |
| MELO3C017752.2 | 1145 | 462 | -1.306 | 9.52752E-19 | down |
| MELO3C017753.2 | 3969 | 1786 | -1.153 | 8.83082E-27 | down |
| MELO3C017754.2 | 10006 | 2529 | -1.984 | 3.24928E-52 | down |
| MELO3C017756.2 | 106 | 46 | -1.199 | 2.9512E-05 | down |
| MELO3C017762.2 | 467 | 2282 | 2.288 | 4.37267E-97 | up |
| MELO3C017772.2 | 13295 | 2762 | -2.267 | 4.93428E-140 | down |
| MELO3C017774.2 | 2686 | 1034 | -1.379 | 1.52271E-54 | down |
| MELO3C017776.2 | 411 | 33 | -3.645 | 2.18119E-62 | down |
| MELO3C017782.2 | 334 | 3 | -6.832 | 2.97386E-42 | down |
| MELO3C017784.2 | 459 | 1002 | 1.125 | 6.83058E-24 | up |
| MELO3C017795.2 | 432 | 183 | -1.241 | 1.18428E-13 | down |
| MELO3C017796.2 | 212 | 972 | 2.197 | 5.68108E-30 | up |
| MELO3C017797.2 | 34 | 4 | -3.064 | 4.24912E-06 | down |
| MELO3C017803.2 | 10 | 1 | -3.163 | 0.007110842 | down |
| MELO3C017807.2 | 24 | 70 | 1.578 | 4.84405E-05 | up |
| MELO3C017808.2 | 801 | 106 | -2.917 | 1.74367E-52 | down |
| MELO3C017809.2 | 14 | 44 | 1.668 | 0.010517683 | up |
| MELO3C017811.2 | 9 | 82 | 3.226 | 2.04867E-06 | up |
| MELO3C017812.2 | 385 | 927 | 1.27 | 1.70834E-16 | up |
| MELO3C017816.2 | 3067 | 679 | -2.178 | 1.86168E-80 | down |
| MELO3C017823.2 | 3276 | 8158 | 1.316 | 1.27336E-35 | up |
| MELO3C017831.2 | 705 | 108 | -2.7 | 9.78483E-45 | down |
| MELO3C017834.2 | 794 | 209 | -1.921 | 2.48254E-37 | down |
| MELO3C017836.2 | 72 | 305 | 2.088 | 1.1952E-21 | up |
| MELO3C017840.2 | 276 | 108 | -1.356 | 1.04436E-12 | down |
| MELO3C017844.2 | 402 | 804 | 1 | 4.82641E-13 | up |
| MELO3C017846.2 | 324 | 102 | -1.674 | 2.70606E-18 | down |
| MELO3C017847.2 | 4921 | 1068 | -2.205 | 9.45572E-73 | down |
| MELO3C017855.2 | 1745 | 827 | -1.08 | 6.69934E-21 | down |
| MELO3C017856.2 | 283 | 83 | -1.776 | 3.021E-12 | down |
| MELO3C017857.2 | 21 | 0 | -6.07 | 1.42825E-05 | down |
| MELO3C017858.2 | 688 | 269 | -1.355 | 7.01762E-19 | down |
| MELO3C017861.2 | 1410 | 584 | -1.272 | 2.0402E-24 | down |
| MELO3C017864.2 | 2190 | 6995 | 1.675 | 2.27815E-36 | up |
| MELO3C017876.2 | 969 | 34 | -4.856 | 4.44425E-101 | down |
| MELO3C017878.2 | 398 | 1331 | 1.741 | 1.49159E-30 | up |
| MELO3C017880.2 | 1840 | 570 | -1.692 | 3.10915E-56 | down |
| MELO3C017883.2 | 151 | 51 | -1.57 | 6.15184E-10 | down |
| MELO3C017889.2 | 1196 | 2529 | 1.081 | 1.01503E-27 | up |
| MELO3C017906.2 | 190 | 489 | 1.367 | 2.47355E-06 | up |
| MELO3C017910.2 | 25 | 66 | 1.397 | 0.000144423 | up |
| MELO3C017911.2 | 5427 | 2341 | -1.213 | 2.95711E-43 | down |
| MELO3C017914.2 | 643 | 319 | -1.008 | 4.28791E-15 | down |
| MELO3C017915.2 | 4219 | 82 | -5.698 | 0 | down |
| MELO3C017917.2 | 7195 | 685 | -3.392 | 3.98399E-162 | down |
| MELO3C017920.2 | 268 | 108 | -1.315 | 1.98322E-09 | down |
| MELO3C017924.2 | 26 | 62 | 1.239 | 0.014101885 | up |
| MELO3C017930.2 | 636 | 1752 | 1.461 | 2.03795E-54 | up |
| MELO3C017931.2 | 902 | 129 | -2.803 | 1.14598E-76 | down |
| MELO3C017933.2 | 421 | 7 | -5.957 | 4.49147E-62 | down |
| MELO3C017935.2 | 52 | 5 | -3.44 | 1.78401E-07 | down |
| MELO3C017940.2 | 2133 | 444 | -2.264 | 2.21892E-64 | down |
| MELO3C017946.2 | 69 | 407 | 2.555 | 1.64135E-18 | up |
| MELO3C017947.2 | 8 | 0 | -5.579 | 0.003065358 | down |
| MELO3C017948.2 | 244 | 50 | -2.271 | 1.55417E-19 | down |
| MELO3C017953.2 | 1561 | 221 | -2.819 | 1.72881E-102 | down |
| MELO3C017954.2 | 11 | 0 | -5.118 | 0.001260132 | down |
| MELO3C017968.2 | 21541 | 10497 | -1.037 | 2.27046E-37 | down |
| MELO3C017971.2 | 44 | 18 | -1.316 | 0.001952731 | down |
| MELO3C017981.2 | 53 | 13 | -2.041 | 0.000270548 | down |
| MELO3C017989.2 | 350 | 1106 | 1.662 | 3.7802E-45 | up |
| MELO3C018003.2 | 1235 | 2 | -9.034 | 3.40349E-63 | down |
| MELO3C018004.2 | 32 | 2 | -4.16 | 3.70908E-06 | down |
| MELO3C018005.2 | 402 | 5241 | 3.703 | 2.38942E-09 | up |
| MELO3C018007.2 | 8 | 0 | -5.52 | 0.001841005 | down |
| MELO3C018008.2 | 339 | 0 | -11.021 | 5.1916E-20 | down |
| MELO3C018009.2 | 5377 | 11 | -8.968 | 5.09573E-38 | down |
| MELO3C018018.2 | 8 | 22 | 1.415 | 0.026365538 | up |
| MELO3C018019.2 | 47 | 102 | 1.119 | 0.000636991 | up |
| MELO3C018020.2 | 55 | 144 | 1.403 | 3.35108E-07 | up |
| MELO3C018023.2 | 2477 | 850 | -1.544 | 5.72867E-49 | down |
| MELO3C018025.2 | 13624 | 46971 | 1.786 | 2.12398E-102 | up |
| MELO3C018048.2 | 54 | 309 | 2.54 | 5.81774E-15 | up |
| MELO3C018049.2 | 16 | 94 | 2.517 | 3.23487E-12 | up |
| MELO3C018051.2 | 791 | 1611 | 1.027 | 6.35735E-13 | up |
| MELO3C018058.2 | 126 | 486 | 1.946 | 1.5999E-25 | up |
| MELO3C018060.2 | 1686 | 4170 | 1.306 | 3.93224E-27 | up |
| MELO3C018066.2 | 2189 | 1079 | -1.02 | 2.62863E-27 | down |
| MELO3C018082.2 | 1710 | 4095 | 1.26 | 8.55621E-51 | up |
| MELO3C018088.2 | 29 | 12 | -1.325 | 0.022922805 | down |
| MELO3C018090.2 | 38 | 5 | -2.908 | 2.52117E-06 | down |
| MELO3C018094.2 | 0 | 4 | 4.349 | 0.028719577 | up |
| MELO3C018128.2 | 1559 | 3256 | 1.062 | 5.14095E-38 | up |
| MELO3C018131.2 | 437 | 145 | -1.593 | 1.01557E-15 | down |
| MELO3C018132.2 | 54 | 417 | 2.949 | 2.07616E-35 | up |
| MELO3C018149.2 | 2524 | 418 | -2.595 | 2.18065E-172 | down |
| MELO3C018158.2 | 0 | 11 | 5.71 | 0.000321829 | up |
| MELO3C018184.2 | 84 | 32 | -1.394 | 0.000913941 | down |
| MELO3C018185.2 | 572 | 4 | -7.119 | 1.62645E-59 | down |
| MELO3C018201.2 | 721 | 293 | -1.3 | 5.91474E-26 | down |
| MELO3C018202.2 | 15 | 2 | -2.743 | 0.00295478 | down |
| MELO3C018223.2 | 558 | 1200 | 1.106 | 8.84843E-19 | up |
| MELO3C018228.2 | 19 | 3 | -2.788 | 0.006524466 | down |
| MELO3C018242.2 | 962 | 11 | -6.464 | 3.87446E-25 | down |
| MELO3C018247.2 | 107 | 18 | -2.56 | 2.08918E-12 | down |
| MELO3C018248.2 | 6 | 139 | 4.554 | 7.88969E-20 | up |
| MELO3C018292.2 | 473 | 143 | -1.719 | 5.54293E-30 | down |
| MELO3C018309.2 | 599 | 228 | -1.388 | 5.94582E-23 | down |
| MELO3C018311.2 | 25 | 0 | -7.243 | 1.11917E-07 | down |
| MELO3C018312.2 | 85 | 6 | -3.898 | 7.19548E-11 | down |
| MELO3C018317.2 | 104 | 19 | -2.435 | 7.33142E-07 | down |
| MELO3C018347.2 | 101 | 21 | -2.23 | 1.58881E-05 | down |
| MELO3C018349.2 | 81 | 30 | -1.419 | 0.001438168 | down |
| MELO3C018353.2 | 1815 | 835 | -1.12 | 3.05215E-17 | down |
| MELO3C018362.2 | 57 | 17 | -1.796 | 3.22304E-05 | down |
| MELO3C018364.2 | 4421 | 1138 | -1.958 | 1.75439E-100 | down |
| MELO3C018366.2 | 36 | 15 | -1.238 | 0.007071182 | down |
| MELO3C018372.2 | 1940 | 492 | -1.981 | 5.83796E-54 | down |
| MELO3C018382.2 | 5705 | 2749 | -1.053 | 1.2149E-24 | down |
| MELO3C018385.2 | 61 | 236 | 1.963 | 1.22351E-10 | up |
| MELO3C018386.2 | 118 | 445 | 1.916 | 1.4438E-30 | up |
| MELO3C018394.2 | 23 | 69 | 1.581 | 7.17149E-05 | up |
| MELO3C018407.2 | 20522 | 9116 | -1.171 | 8.98585E-35 | down |
| MELO3C018409.2 | 5304 | 1164 | -2.188 | 5.94291E-138 | down |
| MELO3C018411.2 | 3 | 26 | 2.904 | 0.000206383 | up |
| MELO3C018412.2 | 3302 | 3 | -10.284 | 2.5929E-96 | down |
| MELO3C018413.2 | 18205 | 2 | -12.915 | 1.78161E-138 | down |
| MELO3C018414.2 | 245 | 1049 | 2.096 | 3.42383E-52 | up |
| MELO3C018415.2 | 64 | 16 | -2.047 | 0.002669797 | down |
| MELO3C018417.2 | 1003 | 379 | -1.406 | 1.8688E-40 | down |
| MELO3C018419.2 | 4607 | 814 | -2.502 | 8.06205E-118 | down |
| MELO3C018421.2 | 715 | 1861 | 1.38 | 1.33247E-36 | up |
| MELO3C018422.2 | 70 | 294 | 2.071 | 4.61115E-21 | up |
| MELO3C018424.2 | 59 | 389 | 2.729 | 8.88148E-26 | up |
| MELO3C018439.2 | 669 | 189 | -1.824 | 1.21153E-40 | down |
| MELO3C018441.2 | 7312 | 3267 | -1.162 | 1.21302E-24 | down |
| MELO3C018442.2 | 186 | 40 | -2.226 | 4.90282E-13 | down |
| MELO3C018445.2 | 1555 | 431 | -1.85 | 9.10748E-22 | down |
| MELO3C018448.2 | 828 | 282 | -1.552 | 4.77552E-32 | down |
| MELO3C018450.2 | 1104 | 340 | -1.699 | 1.72766E-27 | down |
| MELO3C018453.2 | 4532 | 1022 | -2.148 | 6.96234E-96 | down |
| MELO3C018456.2 | 4704 | 1216 | -1.952 | 4.34465E-106 | down |
| MELO3C018463.2 | 21 | 3 | -2.721 | 0.009282442 | down |
| MELO3C018467.2 | 285 | 22 | -3.713 | 1.98562E-33 | down |
| MELO3C018468.2 | 38 | 6 | -2.734 | 6.82811E-06 | down |
| MELO3C018469.2 | 21 | 48 | 1.212 | 0.005874116 | up |
| MELO3C018470.2 | 20 | 4126 | 7.711 | 9.07197E-24 | up |
| MELO3C018476.2 | 6 | 33 | 2.429 | 0.000578776 | up |
| MELO3C018482.2 | 1989 | 597 | -1.737 | 2.09316E-69 | down |
| MELO3C018484.2 | 169 | 48 | -1.812 | 7.27524E-12 | down |
| MELO3C018487.2 | 754 | 363 | -1.054 | 4.44333E-17 | down |
| MELO3C018489.2 | 5085 | 855 | -2.572 | 5.06698E-111 | down |
| MELO3C018490.2 | 7164 | 2143 | -1.742 | 0.000189328 | down |
| MELO3C018492.2 | 1685 | 129 | -3.704 | 1.3804E-67 | down |
| MELO3C018495.2 | 99 | 739 | 2.901 | 6.97198E-62 | up |
| MELO3C018496.2 | 29 | 3 | -3.557 | 1.87643E-05 | down |
| MELO3C018503.2 | 19 | 1141 | 5.931 | 2.80127E-99 | up |
| MELO3C018505.2 | 2910 | 951 | -1.615 | 1.45369E-69 | down |
| MELO3C018509.2 | 429 | 105 | -2.019 | 5.60509E-14 | down |
| MELO3C018514.2 | 16435 | 7471 | -1.137 | 1.38937E-39 | down |
| MELO3C018522.2 | 0 | 60 | 8.179 | 9.80431E-11 | up |
| MELO3C018528.2 | 841 | 6 | -7.147 | 2.32159E-81 | down |
| MELO3C018530.2 | 159 | 391 | 1.294 | 9.48645E-13 | up |
| MELO3C018532.2 | 29 | 664 | 4.541 | 1.98504E-64 | up |
| MELO3C018539.2 | 3 | 47 | 4.137 | 3.32824E-06 | up |
| MELO3C018540.2 | 10 | 0 | -5.977 | 0.000209114 | down |
| MELO3C018551.2 | 19 | 89 | 2.224 | 3.97603E-08 | up |
| MELO3C018552.2 | 1826 | 4169 | 1.191 | 9.22583E-59 | up |
| MELO3C018553.2 | 205 | 83 | -1.304 | 2.40572E-06 | down |
| MELO3C018556.2 | 368 | 757 | 1.04 | 1.13693E-13 | up |
| MELO3C018569.2 | 4110 | 1386 | -1.569 | 1.24387E-61 | down |
| MELO3C018571.2 | 445 | 68 | -2.711 | 4.629E-49 | down |
| MELO3C018572.2 | 9943 | 3079 | -1.691 | 9.53166E-104 | down |
| MELO3C018573.2 | 2560 | 1168 | -1.132 | 3.52973E-29 | down |
| MELO3C018578.2 | 235 | 117 | -1.006 | 8.41383E-08 | down |
| MELO3C018579.2 | 11613 | 918 | -3.661 | 5.52759E-21 | down |
| MELO3C018580.2 | 8756 | 0 | -15.712 | 6.15505E-34 | down |
| MELO3C018582.2 | 111 | 421 | 1.925 | 3.5325E-25 | up |
| MELO3C018593.2 | 365 | 2 | -7.687 | 1.36991E-35 | down |
| MELO3C018594.2 | 218 | 2 | -6.536 | 7.60028E-30 | down |
| MELO3C018598.2 | 77 | 2 | -5.371 | 0.000314416 | down |
| MELO3C018599.2 | 68 | 3 | -4.282 | 8.99613E-07 | down |
| MELO3C018600.2 | 245 | 14 | -4.165 | 5.15395E-05 | down |
| MELO3C018601.2 | 195 | 680 | 1.795 | 1.26391E-23 | up |
| MELO3C018603.2 | 23 | 0 | -7.139 | 1.83861E-07 | down |
| MELO3C018607.2 | 3348 | 926 | -1.854 | 2.39507E-129 | down |
| MELO3C018618.2 | 132 | 37 | -1.859 | 2.49348E-06 | down |
| MELO3C018621.2 | 1971 | 912 | -1.113 | 5.66127E-29 | down |
| MELO3C018625.2 | 19 | 45 | 1.222 | 0.01109583 | up |
| MELO3C018627.2 | 285 | 75 | -1.92 | 1.22127E-13 | down |
| MELO3C018631.2 | 412 | 89 | -2.214 | 1.58481E-14 | down |
| MELO3C018632.2 | 267 | 9 | -4.939 | 8.19359E-28 | down |
| MELO3C018634.2 | 16 | 3 | -2.642 | 0.001987439 | down |
| MELO3C018636.2 | 376 | 101 | -1.886 | 9.24954E-09 | down |
| MELO3C018643.2 | 1294 | 498 | -1.378 | 1.24048E-36 | down |
| MELO3C018645.2 | 8 | 23 | 1.53 | 0.03250266 | up |
| MELO3C018657.2 | 64 | 127 | 1 | 0.001514746 | up |
| MELO3C018658.2 | 761 | 2907 | 1.934 | 2.14776E-53 | up |
| MELO3C018663.2 | 69 | 245 | 1.842 | 2.3867E-14 | up |
| MELO3C018664.2 | 145 | 14 | -3.428 | 1.90982E-18 | down |
| MELO3C018667.2 | 140 | 501 | 1.846 | 2.75898E-16 | up |
| MELO3C018669.2 | 1107 | 234 | -2.242 | 3.72153E-85 | down |
| MELO3C018677.2 | 390 | 195 | -1 | 3.66726E-07 | down |
| MELO3C018681.2 | 7575 | 709 | -3.416 | 7.99976E-117 | down |
| MELO3C018692.2 | 674 | 0 | -12.013 | 8.31411E-24 | down |
| MELO3C018693.2 | 12 | 0 | -6.229 | 4.43654E-05 | down |
| MELO3C018698.2 | 696 | 230 | -1.592 | 4.63629E-23 | down |
| MELO3C018700.2 | 680 | 237 | -1.517 | 0.000853179 | down |
| MELO3C018707.2 | 36 | 15 | -1.326 | 0.004998926 | down |
| MELO3C018713.2 | 26640 | 7719 | -1.787 | 1.09625E-58 | down |
| MELO3C018715.2 | 6947 | 2727 | -1.349 | 1.96997E-73 | down |
| MELO3C018718.2 | 2487 | 295 | -3.072 | 1.74922E-181 | down |
| MELO3C018720.2 | 13792 | 2840 | -2.28 | 1.40489E-150 | down |
| MELO3C018722.2 | 500 | 249 | -1.004 | 1.58505E-12 | down |
| MELO3C018724.2 | 12193 | 5908 | -1.045 | 8.58572E-22 | down |
| MELO3C018729.2 | 2157 | 82 | -4.717 | 2.29044E-136 | down |
| MELO3C018730.2 | 243 | 112 | -1.12 | 1.17515E-08 | down |
| MELO3C018732.2 | 68 | 272 | 2.01 | 4.3132E-20 | up |
| MELO3C018733.2 | 714 | 210 | -1.761 | 6.0772E-16 | down |
| MELO3C018738.2 | 950 | 46 | -4.367 | 3.96831E-139 | down |
| MELO3C018739.2 | 54 | 1 | -6.534 | 5.63588E-09 | down |
| MELO3C018740.2 | 148 | 346 | 1.231 | 1.57283E-08 | up |
| MELO3C018742.2 | 254 | 102 | -1.318 | 4.04046E-12 | down |
| MELO3C018744.2 | 39 | 4 | -3.233 | 2.70659E-05 | down |
| MELO3C018747.2 | 32 | 153 | 2.26 | 5.16568E-11 | up |
| MELO3C018748.2 | 3284 | 154 | -4.413 | 6.5392E-273 | down |
| MELO3C018749.2 | 934 | 243 | -1.942 | 5.88537E-59 | down |
| MELO3C018758.2 | 77 | 268 | 1.801 | 7.06862E-15 | up |
| MELO3C018762.2 | 2412 | 1014 | -1.251 | 1.90342E-26 | down |
| MELO3C018772.2 | 5204 | 1772 | -1.554 | 1.59259E-96 | down |
| MELO3C018774.2 | 1 | 98 | 6.03 | 4.06796E-14 | up |
| MELO3C018775.2 | 8200 | 2153 | -1.93 | 4.07537E-72 | down |
| MELO3C018782.2 | 312 | 822 | 1.397 | 3.35507E-20 | up |
| MELO3C018784.2 | 2 | 17 | 2.73 | 0.001850791 | up |
| MELO3C018785.2 | 26 | 1 | -4.16 | 1.70569E-05 | down |
| MELO3C018798.2 | 84 | 4 | -4.448 | 3.13194E-16 | down |
| MELO3C018799.2 | 65 | 2 | -5.199 | 4.61504E-12 | down |
| MELO3C018808.2 | 1 | 11 | 3.844 | 0.006594202 | up |
| MELO3C018813.2 | 3882 | 1139 | -1.77 | 6.84335E-67 | down |
| MELO3C018817.2 | 48 | 182 | 1.928 | 9.46563E-14 | up |
| MELO3C018818.2 | 781 | 357 | -1.127 | 7.31775E-20 | down |
| MELO3C018819.2 | 953 | 139 | -2.773 | 3.91026E-61 | down |
| MELO3C018823.2 | 0 | 16 | 6.232 | 1.63886E-05 | up |
| MELO3C018825.2 | 13 | 0 | -6.331 | 2.5544E-05 | down |
| MELO3C018828.2 | 114 | 261 | 1.204 | 5.26863E-08 | up |
| MELO3C018830.2 | 142 | 406 | 1.516 | 7.37356E-19 | up |
| MELO3C018831.2 | 85 | 235 | 1.461 | 3.98455E-09 | up |
| MELO3C018832.2 | 531 | 1225 | 1.204 | 1.36461E-29 | up |
| MELO3C018839.2 | 930 | 324 | -1.525 | 2.4237E-15 | down |
| MELO3C018844.2 | 230 | 84 | -1.453 | 2.82835E-09 | down |
| MELO3C018850.2 | 39 | 0 | -7.906 | 1.09851E-09 | down |
| MELO3C018851.2 | 109 | 3 | -5.361 | 2.87325E-20 | down |
| MELO3C018853.2 | 26 | 76 | 1.564 | 5.86865E-06 | up |
| MELO3C018859.2 | 259 | 117 | -1.146 | 3.0764E-05 | down |
| MELO3C018862.2 | 25 | 51 | 1.015 | 0.012506742 | up |
| MELO3C018873.2 | 39 | 170 | 2.107 | 3.21279E-13 | up |
| MELO3C018878.2 | 281 | 9069 | 5.016 | 7.87663E-240 | up |
| MELO3C018879.2 | 6 | 0 | -5.1 | 0.005595329 | down |
| MELO3C018880.2 | 54239 | 18148 | -1.58 | 4.70574E-85 | down |
| MELO3C018881.2 | 0 | 344 | 10.703 | 2.798E-19 | up |
| MELO3C018888.2 | 19 | 1500 | 6.353 | 2.87521E-147 | up |
| MELO3C018892.2 | 5513 | 946 | -2.543 | 2.41191E-155 | down |
| MELO3C018897.2 | 185 | 452 | 1.288 | 3.1833E-18 | up |
| MELO3C018899.2 | 5 | 0 | -4.138 | 0.018403022 | down |
| MELO3C018910.2 | 45 | 93 | 1.043 | 0.001541573 | up |
| MELO3C018925.2 | 4 | 0 | -4.517 | 0.029263239 | down |
| MELO3C018937.2 | 28652 | 30 | -9.886 | 0 | down |
| MELO3C018942.2 | 70 | 258 | 1.898 | 1.24667E-12 | up |
| MELO3C018950.2 | 7323 | 3135 | -1.224 | 2.42631E-56 | down |
| MELO3C018954.2 | 5816 | 2258 | -1.365 | 3.87616E-43 | down |
| MELO3C018959.2 | 22 | 6 | -1.788 | 0.006288644 | down |
| MELO3C018967.2 | 612 | 111 | -2.464 | 2.5249E-16 | down |
| MELO3C018969.2 | 2950 | 1459 | -1.016 | 6.08415E-24 | down |
| MELO3C018985.2 | 831 | 1953 | 1.233 | 1.52618E-26 | up |
| MELO3C018994.2 | 6796 | 2451 | -1.471 | 1.98136E-35 | down |
| MELO3C018998.2 | 2723 | 1345 | -1.018 | 2.21376E-22 | down |
| MELO3C019002.2 | 2 | 164 | 6.771 | 4.52672E-17 | up |
| MELO3C019017.2 | 107 | 0 | -9.36 | 7.48118E-14 | down |
| MELO3C019025.2 | 0 | 37 | 7.489 | 2.86215E-08 | up |
| MELO3C019056.2 | 1194 | 2856 | 1.259 | 6.51133E-38 | up |
| MELO3C019065.2 | 9 | 0 | -5.755 | 0.00059089 | down |
| MELO3C019082.2 | 184 | 407 | 1.143 | 3.06584E-11 | up |
| MELO3C019099.2 | 15 | 34 | 1.214 | 0.023002331 | up |
| MELO3C019110.2 | 185 | 30 | -2.652 | 6.50666E-19 | down |
| MELO3C019112.2 | 352 | 1303 | 1.888 | 4.43362E-47 | up |
| MELO3C019125.2 | 236 | 54 | -2.123 | 1.93677E-12 | down |
| MELO3C019148.2 | 3026 | 349 | -3.121 | 5.37297E-186 | down |
| MELO3C019149.2 | 294 | 120 | -1.298 | 3.76744E-12 | down |
| MELO3C019150.2 | 0 | 206 | 9.961 | 4.30882E-16 | up |
| MELO3C019156.2 | 4414 | 1809 | -1.287 | 1.69262E-51 | down |
| MELO3C019161.2 | 29 | 240 | 3.047 | 1.76004E-18 | up |
| MELO3C019171.2 | 2785 | 6171 | 1.148 | 1.67708E-11 | up |
| MELO3C019177.2 | 102 | 24 | -2.088 | 1.79349E-07 | down |
| MELO3C019186.2 | 9 | 0 | -5.856 | 0.000279945 | down |
| MELO3C019188.2 | 40 | 10 | -2.005 | 0.000111221 | down |
| MELO3C019192.2 | 8 | 40 | 2.346 | 1.95257E-05 | up |
| MELO3C019201.2 | 8 | 29 | 1.824 | 0.0039779 | up |
| MELO3C019203.2 | 497 | 26 | -4.215 | 8.87185E-79 | down |
| MELO3C019205.2 | 72 | 740 | 3.347 | 1.2358E-58 | up |
| MELO3C019209.2 | 4 | 0 | -4.752 | 0.02050639 | down |
| MELO3C019210.2 | 30 | 0 | -7.493 | 2.94825E-08 | down |
| MELO3C019215.2 | 13805 | 3693 | -1.902 | 5.60003E-68 | down |
| MELO3C019221.2 | 1336 | 0 | -13 | 9.46168E-28 | down |
| MELO3C019224.2 | 7 | 0 | -5.375 | 0.002925021 | down |
| MELO3C019225.2 | 752 | 317 | -1.247 | 2.6851E-22 | down |
| MELO3C019227.2 | 2 | 17 | 2.908 | 0.002390953 | up |
| MELO3C019231.2 | 906 | 212 | -2.094 | 4.16289E-40 | down |
| MELO3C019232.2 | 69 | 6 | -3.528 | 2.2169E-12 | down |
| MELO3C019254.2 | 169 | 810 | 2.265 | 5.05253E-39 | up |
| MELO3C019257.2 | 18 | 61 | 1.749 | 1.27936E-05 | up |
| MELO3C019265.2 | 14 | 58 | 2.073 | 9.22605E-06 | up |
| MELO3C019266.2 | 63 | 1040 | 4.054 | 2.44175E-118 | up |
| MELO3C019273.2 | 1283 | 378 | -1.762 | 1.09626E-42 | down |
| MELO3C019287.2 | 348 | 738 | 1.081 | 1.73122E-15 | up |
| MELO3C019291.2 | 4 | 19 | 2.293 | 0.008330142 | up |
| MELO3C019297.2 | 220 | 3 | -6.22 | 1.03363E-28 | down |
| MELO3C019303.2 | 77 | 365 | 2.246 | 2.92412E-27 | up |
| MELO3C019310.2 | 2133 | 420 | -2.345 | 2.66744E-66 | down |
| MELO3C019320.2 | 1624 | 463 | -1.811 | 2.54753E-39 | down |
| MELO3C019335.2 | 172 | 23 | -2.917 | 4.97521E-20 | down |
| MELO3C019337.2 | 43 | 6 | -2.922 | 2.23061E-06 | down |
| MELO3C019348.2 | 1188 | 500 | -1.247 | 1.87461E-31 | down |
| MELO3C019361.2 | 3 | 62 | 4.366 | 5.21461E-11 | up |
| MELO3C019366.2 | 992 | 315 | -1.654 | 3.01846E-37 | down |
| MELO3C019373.2 | 4119 | 920 | -2.164 | 2.53817E-118 | down |
| MELO3C019377.2 | 1552 | 64 | -4.602 | 1.00327E-164 | down |
| MELO3C019379.2 | 28 | 124 | 2.142 | 1.08699E-10 | up |
| MELO3C019382.2 | 2687 | 419 | -2.682 | 2.15193E-141 | down |
| MELO3C019393.2 | 275 | 65 | -2.086 | 7.69888E-21 | down |
| MELO3C019394.2 | 13 | 59 | 2.218 | 1.81523E-06 | up |
| MELO3C019397.2 | 10 | 28 | 1.426 | 0.028562197 | up |
| MELO3C019407.2 | 854 | 352 | -1.278 | 4.20873E-27 | down |
| MELO3C019410.2 | 11265 | 866 | -3.702 | 0 | down |
| MELO3C019416.2 | 15 | 4 | -1.932 | 0.027819259 | down |
| MELO3C019418.2 | 410 | 101 | -2.021 | 1.55084E-21 | down |
| MELO3C019435.2 | 11 | 889 | 6.409 | 1.21454E-96 | up |
| MELO3C019437.2 | 1 | 9 | 3.621 | 0.012115457 | up |
| MELO3C019440.2 | 12849 | 1378 | -3.222 | 1.35644E-166 | down |
| MELO3C019441.2 | 529 | 198 | -1.419 | 3.2225E-06 | down |
| MELO3C019445.2 | 33 | 259 | 2.992 | 4.36608E-32 | up |
| MELO3C019447.2 | 150 | 1069 | 2.832 | 1.57876E-107 | up |
| MELO3C019451.2 | 38 | 148 | 1.976 | 5.25401E-12 | up |
| MELO3C019453.2 | 184 | 611 | 1.735 | 5.10914E-31 | up |
| MELO3C019469.2 | 25 | 4 | -2.682 | 0.009386087 | down |
| MELO3C019470.2 | 4905 | 1 | -12.026 | 4.84113E-60 | down |
| MELO3C019474.2 | 8 | 0 | -5.694 | 0.004457912 | down |
| MELO3C019475.2 | 1603 | 623 | -1.365 | 2.39521E-37 | down |
| MELO3C019497.2 | 5290 | 2275 | -1.218 | 3.00032E-42 | down |
| MELO3C019506.2 | 28 | 1 | -4.607 | 6.64847E-05 | down |
| MELO3C019527.2 | 2730 | 1354 | -1.012 | 9.36112E-41 | down |
| MELO3C019529.2 | 237 | 63 | -1.898 | 1.62551E-09 | down |
| MELO3C019538.2 | 7 | 0 | -5.499 | 0.001545986 | down |
| MELO3C019540.2 | 37 | 10 | -1.932 | 0.000603284 | down |
| MELO3C019544.2 | 37 | 136 | 1.855 | 5.25093E-09 | up |
| MELO3C019548.2 | 389 | 179 | -1.117 | 1.61593E-07 | down |
| MELO3C019552.2 | 529 | 31 | -4.109 | 7.57003E-70 | down |
| MELO3C019561.2 | 402 | 128 | -1.649 | 1.64814E-17 | down |
| MELO3C019563.2 | 121 | 397 | 1.718 | 2.85487E-22 | up |
| MELO3C019567.2 | 7 | 1 | -3.538 | 0.031481646 | down |
| MELO3C019572.2 | 1982 | 313 | -2.664 | 3.38972E-106 | down |
| MELO3C019573.2 | 4087 | 12155 | 1.572 | 5.09696E-52 | up |
| MELO3C019588.2 | 246 | 741 | 1.591 | 7.17671E-29 | up |
| MELO3C019595.2 | 1584 | 596 | -1.412 | 3.74969E-26 | down |
| MELO3C019598.2 | 3815 | 702 | -2.442 | 1.13896E-175 | down |
| MELO3C019599.2 | 674 | 294 | -1.198 | 2.26824E-11 | down |
| MELO3C019603.2 | 1425 | 602 | -1.243 | 4.55308E-20 | down |
| MELO3C019616.2 | 1452 | 652 | -1.153 | 2.86985E-27 | down |
| MELO3C019619.2 | 5021 | 2373 | -1.081 | 3.46529E-37 | down |
| MELO3C019621.2 | 694 | 224 | -1.631 | 3.08031E-14 | down |
| MELO3C019634.2 | 17858 | 4818 | -1.89 | 1.27491E-57 | down |
| MELO3C019639.2 | 491 | 82 | -2.591 | 2.48458E-24 | down |
| MELO3C019649.2 | 205 | 42 | -2.281 | 5.75938E-15 | down |
| MELO3C019654.2 | 34 | 2 | -4.224 | 2.71834E-07 | down |
| MELO3C019677.2 | 111046 | 638 | -7.444 | 0 | down |
| MELO3C019679.2 | 37 | 10 | -1.94 | 0.006692318 | down |
| MELO3C019680.2 | 13 | 1 | -4.424 | 0.001386094 | down |
| MELO3C019691.2 | 255 | 1392 | 2.449 | 1.57738E-86 | up |
| MELO3C019696.2 | 20 | 6 | -1.642 | 0.028840967 | down |
| MELO3C019717.2 | 3144 | 1369 | -1.199 | 5.9079E-34 | down |
| MELO3C019719.2 | 253 | 514 | 1.023 | 5.81571E-15 | up |
| MELO3C019723.2 | 379 | 916 | 1.276 | 1.83081E-22 | up |
| MELO3C019735.2 | 3700 | 897 | -2.043 | 1.12679E-32 | down |
| MELO3C019745.2 | 409 | 0 | -11.292 | 2.4364E-21 | down |
| MELO3C019759.2 | 3547 | 228 | -3.96 | 3.66565E-161 | down |
| MELO3C019781.2 | 1910 | 858 | -1.153 | 2.5897E-20 | down |
| MELO3C019783.2 | 134 | 296 | 1.149 | 3.3028E-09 | up |
| MELO3C019785.2 | 2485 | 1232 | -1.013 | 1.13782E-19 | down |
| MELO3C019791.2 | 402 | 1395 | 1.797 | 6.09032E-28 | up |
| MELO3C019796.2 | 8 | 1 | -3.203 | 0.024811581 | down |
| MELO3C019808.2 | 88 | 5 | -4.128 | 2.03175E-15 | down |
| MELO3C019809.2 | 25 | 109 | 2.107 | 3.69874E-06 | up |
| MELO3C019810.2 | 41 | 268 | 2.734 | 1.55078E-20 | up |
| MELO3C019816.2 | 37 | 10 | -1.864 | 0.000215303 | down |
| MELO3C019818.2 | 43 | 400 | 3.209 | 6.15207E-55 | up |
| MELO3C019833.2 | 0 | 790 | 11.902 | 1.17581E-23 | up |
| MELO3C019840.2 | 305 | 2937 | 3.271 | 5.36999E-218 | up |
| MELO3C019842.2 | 17 | 48 | 1.482 | 0.000846626 | up |
| MELO3C019843.2 | 2 | 19 | 3.635 | 0.001027046 | up |
| MELO3C019846.2 | 673 | 1532 | 1.188 | 1.73064E-21 | up |
| MELO3C019849.2 | 3 | 120 | 5.342 | 2.65179E-19 | up |
| MELO3C019853.2 | 411 | 1023 | 1.316 | 3.76066E-30 | up |
| MELO3C019871.2 | 933 | 3348 | 1.843 | 5.81302E-43 | up |
| MELO3C019879.2 | 1072 | 2255 | 1.074 | 6.72909E-25 | up |
| MELO3C019884.2 | 2881 | 6067 | 1.074 | 4.34039E-10 | up |
| MELO3C019906.2 | 1106 | 197 | -2.486 | 4.28377E-08 | down |
| MELO3C019912.2 | 1784 | 598 | -1.575 | 9.01387E-34 | down |
| MELO3C019917.2 | 23 | 1 | -5.321 | 1.41733E-05 | down |
| MELO3C019930.2 | 109 | 421 | 1.948 | 1.60529E-21 | up |
| MELO3C019939.2 | 1019 | 2283 | 1.165 | 1.11313E-15 | up |
| MELO3C019941.2 | 8349 | 2756 | -1.599 | 1.79248E-95 | down |
| MELO3C019942.2 | 1897 | 679 | -1.483 | 1.95746E-43 | down |
| MELO3C019948.2 | 756 | 73 | -3.377 | 1.90433E-98 | down |
| MELO3C019962.2 | 149 | 401 | 1.43 | 5.47127E-19 | up |
| MELO3C019965.2 | 6 | 34 | 2.48 | 0.000150852 | up |
| MELO3C019970.2 | 17 | 66 | 1.925 | 0.000244855 | up |
| MELO3C019974.2 | 421 | 187 | -1.167 | 5.77476E-13 | down |
| MELO3C019981.2 | 3954 | 1524 | -1.375 | 3.87407E-46 | down |
| MELO3C019982.2 | 243 | 94 | -1.368 | 5.58531E-10 | down |
| MELO3C019983.2 | 8767 | 3050 | -1.523 | 2.02741E-71 | down |
| MELO3C019994.2 | 150 | 9 | -4.055 | 0.001191639 | down |
| MELO3C019997.2 | 340 | 1385 | 2.026 | 1.91133E-37 | up |
| MELO3C019998.2 | 30 | 165 | 2.475 | 7.72779E-15 | up |
| MELO3C020000.2 | 105 | 21 | -2.332 | 3.61672E-12 | down |
| MELO3C020001.2 | 263 | 710 | 1.434 | 8.11607E-22 | up |
| MELO3C020005.2 | 1021 | 9428 | 3.207 | 1.14652E-31 | up |
| MELO3C020007.2 | 5020 | 80 | -5.959 | 5.54094E-241 | down |
| MELO3C020010.2 | 229 | 685 | 1.581 | 5.0482E-20 | up |
| MELO3C020011.2 | 52 | 267 | 2.356 | 2.02652E-17 | up |
| MELO3C020023.2 | 1038 | 2452 | 1.238 | 8.90209E-38 | up |
| MELO3C020032.2 | 29 | 160 | 2.44 | 1.46678E-17 | up |
| MELO3C020052.2 | 50 | 13 | -1.921 | 0.000232489 | down |
| MELO3C020055.2 | 2393 | 5498 | 1.2 | 2.53687E-61 | up |
| MELO3C020056.2 | 20 | 5 | -1.985 | 0.005208071 | down |
| MELO3C020061.2 | 30 | 0 | -7.527 | 1.59699E-08 | down |
| MELO3C020063.2 | 0 | 24 | 6.89 | 1.58152E-06 | up |
| MELO3C020091.2 | 23 | 5 | -2.393 | 0.000433803 | down |
| MELO3C020093.2 | 1226 | 3055 | 1.318 | 2.85688E-50 | up |
| MELO3C020095.2 | 169 | 44 | -1.95 | 6.31166E-15 | down |
| MELO3C020096.2 | 137 | 38 | -1.869 | 3.32375E-10 | down |
| MELO3C020099.2 | 243 | 59 | -2.047 | 1.57528E-14 | down |
| MELO3C020101.2 | 476 | 1118 | 1.231 | 3.98215E-22 | up |
| MELO3C020115.2 | 4869 | 1115 | -2.126 | 5.27031E-85 | down |
| MELO3C020120.2 | 493 | 0 | -11.562 | 4.46863E-22 | down |
| MELO3C020121.2 | 0 | 87 | 8.723 | 1.61152E-12 | up |
| MELO3C020131.2 | 1956 | 114 | -4.099 | 1.61622E-28 | down |
| MELO3C020132.2 | 2484 | 429 | -2.532 | 1.86583E-44 | down |
| MELO3C020138.2 | 5455 | 1406 | -1.955 | 4.62609E-83 | down |
| MELO3C020146.2 | 3379 | 15762 | 2.221 | 3.22401E-07 | up |
| MELO3C020156.2 | 0 | 283 | 10.422 | 3.76275E-18 | up |
| MELO3C020160.2 | 8 | 0 | -5.576 | 0.001924392 | down |
| MELO3C020166.2 | 24 | 3 | -3.036 | 0.000341172 | down |
| MELO3C020171.2 | 3873 | 1607 | -1.27 | 4.18597E-32 | down |
| MELO3C020181.2 | 1163 | 2862 | 1.3 | 6.69985E-41 | up |
| MELO3C020204.2 | 2038 | 926 | -1.139 | 2.73926E-30 | down |
| MELO3C020218.2 | 11 | 32 | 1.549 | 0.019042083 | up |
| MELO3C020224.2 | 55 | 5 | -3.451 | 6.70135E-06 | down |
| MELO3C020257.2 | 0 | 26 | 6.968 | 2.07011E-07 | up |
| MELO3C020268.2 | 140 | 878 | 2.653 | 6.32027E-24 | up |
| MELO3C020270.2 | 1093 | 2361 | 1.11 | 0.000652675 | up |
| MELO3C020278.2 | 725 | 275 | -1.398 | 8.7123E-28 | down |
| MELO3C020292.2 | 7 | 23 | 1.649 | 0.019311002 | up |
| MELO3C020296.2 | 2872 | 1198 | -1.262 | 2.22302E-44 | down |
| MELO3C020303.2 | 192 | 429 | 1.159 | 5.75435E-13 | up |
| MELO3C020311.2 | 1811 | 611 | -1.569 | 5.37733E-24 | down |
| MELO3C020312.2 | 5656 | 2010 | -1.493 | 5.49544E-56 | down |
| MELO3C020316.2 | 767 | 1572 | 1.034 | 5.70448E-16 | up |
| MELO3C020317.2 | 173 | 1398 | 3.011 | 2.21503E-60 | up |
| MELO3C020318.2 | 370 | 164 | -1.173 | 1.88979E-11 | down |
| MELO3C020321.2 | 928 | 2477 | 1.416 | 3.98043E-49 | up |
| MELO3C020328.2 | 134 | 59 | -1.177 | 0.000122871 | down |
| MELO3C020341.2 | 27801 | 11855 | -1.23 | 2.7781E-35 | down |
| MELO3C020370.2 | 399 | 2 | -8.081 | 1.87413E-31 | down |
| MELO3C020373.2 | 4 | 96 | 4.676 | 1.19552E-14 | up |
| MELO3C020375.2 | 167 | 482 | 1.533 | 4.92292E-17 | up |
| MELO3C020377.2 | 28 | 191 | 2.798 | 2.21446E-19 | up |
| MELO3C020384.2 | 4 | 0 | -4.629 | 0.027948478 | down |
| MELO3C020392.2 | 968 | 483 | -1.004 | 8.86626E-15 | down |
| MELO3C020393.2 | 75 | 21 | -1.884 | 1.98491E-07 | down |
| MELO3C020398.2 | 2052 | 541 | -1.922 | 3.9281E-05 | down |
| MELO3C020400.2 | 86 | 25 | -1.782 | 3.13513E-06 | down |
| MELO3C020408.2 | 981 | 2237 | 1.189 | 1.31571E-15 | up |
| MELO3C020409.2 | 0 | 7 | 5.112 | 0.003534871 | up |
| MELO3C020418.2 | 49 | 0 | -8.229 | 9.80048E-11 | down |
| MELO3C020419.2 | 50 | 1 | -6.405 | 1.19731E-08 | down |
| MELO3C020421.2 | 0 | 578 | 11.453 | 5.93215E-22 | up |
| MELO3C020426.2 | 6542 | 16813 | 1.362 | 5.93585E-18 | up |
| MELO3C020428.2 | 669 | 6136 | 3.199 | 2.97134E-93 | up |
| MELO3C020432.2 | 26868 | 67523 | 1.329 | 8.37656E-105 | up |
| MELO3C020434.2 | 3 | 110 | 5.026 | 2.81606E-17 | up |
| MELO3C020446.2 | 117 | 48 | -1.285 | 2.92965E-05 | down |
| MELO3C020457.2 | 0 | 268 | 10.348 | 7.99199E-18 | up |
| MELO3C020464.2 | 26 | 6 | -2.047 | 0.00081903 | down |
| MELO3C020486.2 | 2028 | 4171 | 1.04 | 2.14631E-43 | up |
| MELO3C020496.2 | 1787 | 53 | -5.092 | 3.46336E-29 | down |
| MELO3C020508.2 | 493 | 1238 | 1.33 | 4.1975E-19 | up |
| MELO3C020510.2 | 2357 | 739 | -1.672 | 3.67462E-60 | down |
| MELO3C020515.2 | 2787 | 781 | -1.836 | 1.64346E-51 | down |
| MELO3C020517.2 | 85 | 4 | -4.305 | 0.007142799 | down |
| MELO3C020521.2 | 1864 | 5924 | 1.668 | 2.3214E-34 | up |
| MELO3C020532.2 | 918 | 440 | -1.062 | 9.3776E-09 | down |
| MELO3C020535.2 | 494 | 12 | -5.434 | 2.35261E-14 | down |
| MELO3C020537.2 | 50 | 277 | 2.461 | 6.77948E-17 | up |
| MELO3C020541.2 | 66 | 778 | 3.575 | 2.82414E-35 | up |
| MELO3C020542.2 | 11 | 46 | 2.114 | 9.65949E-05 | up |
| MELO3C020543.2 | 5713 | 2693 | -1.085 | 6.39353E-33 | down |
| MELO3C020554.2 | 2233 | 898 | -1.316 | 1.57468E-40 | down |
| MELO3C020561.2 | 130 | 11 | -3.598 | 3.10799E-09 | down |
| MELO3C020566.2 | 2447 | 1011 | -1.275 | 2.27361E-52 | down |
| MELO3C020570.2 | 683 | 9411 | 3.785 | 1.87648E-163 | up |
| MELO3C020577.2 | 416 | 923 | 1.147 | 3.39536E-16 | up |
| MELO3C020588.2 | 1691 | 288 | -2.554 | 1.26238E-80 | down |
| MELO3C020589.2 | 6766 | 2306 | -1.553 | 2.39781E-63 | down |
| MELO3C020591.2 | 32 | 4559 | 7.15 | 0 | up |
| MELO3C020592.2 | 750 | 150 | -2.323 | 2.26354E-05 | down |
| MELO3C020594.2 | 3910 | 7 | -9.169 | 2.3825E-161 | down |
| MELO3C020596.2 | 17 | 62 | 1.845 | 1.65727E-05 | up |
| MELO3C020597.2 | 177 | 518 | 1.547 | 1.23379E-20 | up |
| MELO3C020600.2 | 135 | 563 | 2.055 | 2.68429E-18 | up |
| MELO3C020601.2 | 2952 | 1354 | -1.124 | 5.26391E-20 | down |
| MELO3C020605.2 | 177 | 447 | 1.334 | 1.09785E-14 | up |
| MELO3C020609.2 | 216 | 758 | 1.813 | 1.31884E-34 | up |
| MELO3C020616.2 | 923 | 2003 | 1.116 | 2.36214E-16 | up |
| MELO3C020617.2 | 4067 | 8188 | 1.009 | 6.06307E-26 | up |
| MELO3C020630.2 | 315 | 1396 | 2.152 | 6.76325E-24 | up |
| MELO3C020637.2 | 613 | 2200 | 1.842 | 2.51732E-51 | up |
| MELO3C020644.2 | 820 | 227 | -1.851 | 9.08636E-31 | down |
| MELO3C020652.2 | 5233 | 1628 | -1.685 | 9.12886E-102 | down |
| MELO3C020653.2 | 17 | 3 | -2.502 | 0.008417053 | down |
| MELO3C020666.2 | 13781 | 5154 | -1.419 | 1.61998E-44 | down |
| MELO3C020670.2 | 0 | 6 | 4.878 | 0.024976704 | up |
| MELO3C020675.2 | 6125 | 1613 | -1.926 | 2.37394E-68 | down |
| MELO3C020681.2 | 734 | 28 | -4.735 | 1.09874E-84 | down |
| MELO3C020688.2 | 875 | 46 | -4.244 | 2.00555E-115 | down |
| MELO3C020695.2 | 46 | 103 | 1.149 | 3.37042E-05 | up |
| MELO3C020703.2 | 6 | 0 | -4.324 | 0.010217475 | down |
| MELO3C020704.2 | 2717 | 5672 | 1.061 | 9.7158E-25 | up |
| MELO3C020742.2 | 9 | 119 | 3.669 | 3.82459E-19 | up |
| MELO3C020744.2 | 4895 | 2329 | -1.072 | 4.89657E-30 | down |
| MELO3C020749.2 | 2743 | 992 | -1.469 | 4.24509E-59 | down |
| MELO3C020752.2 | 26 | 290 | 3.493 | 8.18804E-43 | up |
| MELO3C020761.2 | 38 | 8 | -2.322 | 0.002634966 | down |
| MELO3C020763.2 | 26 | 8 | -1.655 | 0.008874882 | down |
| MELO3C020765.2 | 69 | 5 | -3.863 | 1.41067E-12 | down |
| MELO3C020771.2 | 42 | 14 | -1.619 | 0.000917341 | down |
| MELO3C020772.2 | 407 | 49 | -3.073 | 8.46129E-56 | down |
| MELO3C020773.2 | 1894 | 546 | -1.796 | 1.76214E-32 | down |
| MELO3C020782.2 | 36 | 0 | -6.806 | 1.4949E-07 | down |
| MELO3C020791.2 | 60 | 124 | 1.047 | 0.0027996 | up |
| MELO3C020792.2 | 9 | 87 | 3.272 | 2.42542E-11 | up |
| MELO3C020793.2 | 27 | 204 | 2.922 | 3.52708E-15 | up |
| MELO3C020796.2 | 35 | 1 | -5.922 | 2.67658E-05 | down |
| MELO3C020798.2 | 1985 | 234 | -3.082 | 2.15636E-44 | down |
| MELO3C020799.2 | 92 | 3 | -5.001 | 3.6284E-15 | down |
| MELO3C020802.2 | 389 | 810 | 1.059 | 1.92158E-18 | up |
| MELO3C020803.2 | 4 | 28 | 2.866 | 0.000625909 | up |
| MELO3C020805.2 | 13 | 0 | -6.284 | 3.41828E-05 | down |
| MELO3C020806.2 | 180 | 487 | 1.438 | 3.21765E-17 | up |
| MELO3C020809.2 | 32 | 85 | 1.426 | 6.86395E-05 | up |
| MELO3C020810.2 | 8133 | 19284 | 1.246 | 2.68991E-32 | up |
| MELO3C020812.2 | 413 | 132 | -1.651 | 1.04517E-26 | down |
| MELO3C020817.2 | 115 | 1740 | 3.919 | 3.46571E-122 | up |
| MELO3C020822.2 | 2585 | 800 | -1.693 | 3.70576E-71 | down |
| MELO3C020823.2 | 1154 | 494 | -1.225 | 9.71418E-32 | down |
| MELO3C020829.2 | 2253 | 4838 | 1.102 | 1.16246E-23 | up |
| MELO3C020830.2 | 20 | 148 | 2.906 | 2.14437E-18 | up |
| MELO3C020834.2 | 185 | 389 | 1.073 | 2.65004E-09 | up |
| MELO3C020835.2 | 22 | 90 | 2.003 | 5.65095E-08 | up |
| MELO3C020844.2 | 717 | 105 | -2.761 | 2.21191E-71 | down |
| MELO3C020845.2 | 739 | 265 | -1.481 | 1.42643E-25 | down |
| MELO3C020848.2 | 266 | 803 | 1.596 | 2.10629E-39 | up |
| MELO3C020851.2 | 291 | 103 | -1.507 | 6.90659E-13 | down |
| MELO3C020855.2 | 775 | 1625 | 1.068 | 4.2236E-23 | up |
| MELO3C020857.2 | 1 | 32 | 4.425 | 5.90042E-06 | up |
| MELO3C020860.2 | 134 | 627 | 2.227 | 3.58366E-22 | up |
| MELO3C020861.2 | 919 | 201 | -2.19 | 5.65341E-37 | down |
| MELO3C020864.2 | 8 | 1 | -3.819 | 0.016754573 | down |
| MELO3C020867.2 | 62 | 874 | 3.818 | 6.12409E-102 | up |
| MELO3C020872.2 | 13130 | 4306 | -1.608 | 3.15244E-43 | down |
| MELO3C020877.2 | 1232 | 2640 | 1.1 | 6.18464E-13 | up |
| MELO3C020879.2 | 47 | 20 | -1.288 | 0.002496721 | down |
| MELO3C020886.2 | 5 | 25 | 2.469 | 0.000312829 | up |
| MELO3C020889.2 | 418 | 187 | -1.16 | 1.89385E-14 | down |
| MELO3C020900.2 | 359 | 62 | -2.544 | 3.87801E-30 | down |
| MELO3C020901.2 | 13 | 58 | 2.151 | 1.01006E-05 | up |
| MELO3C020910.2 | 225 | 465 | 1.047 | 5.16528E-14 | up |
| MELO3C020912.2 | 8171 | 3481 | -1.231 | 3.12074E-76 | down |
| MELO3C020913.2 | 15214 | 4430 | -1.78 | 3.96982E-127 | down |
| MELO3C020915.2 | 421 | 90 | -2.234 | 2.30985E-41 | down |
| MELO3C020917.2 | 1964 | 354 | -2.474 | 8.08034E-58 | down |
| MELO3C020919.2 | 1378 | 577 | -1.256 | 7.41338E-32 | down |
| MELO3C020928.2 | 52 | 2 | -4.45 | 8.54516E-07 | down |
| MELO3C020931.2 | 395 | 198 | -1 | 2.31635E-09 | down |
| MELO3C020932.2 | 220 | 754 | 1.775 | 1.55098E-27 | up |
| MELO3C020934.2 | 119 | 0 | -8.546 | 8.05165E-11 | down |
| MELO3C020941.2 | 3550 | 1580 | -1.168 | 1.71516E-32 | down |
| MELO3C020942.2 | 3813 | 1093 | -1.802 | 2.37054E-51 | down |
| MELO3C020948.2 | 400 | 139 | -1.534 | 2.88911E-18 | down |
| MELO3C020952.2 | 8512 | 3171 | -1.424 | 1.05606E-65 | down |
| MELO3C020956.2 | 0 | 13 | 5.935 | 0.000144382 | up |
| MELO3C020963.2 | 154 | 19 | -3.05 | 5.4897E-10 | down |
| MELO3C020965.2 | 306 | 83 | -1.872 | 1.72925E-20 | down |
| MELO3C020967.2 | 50 | 10 | -2.368 | 6.21109E-07 | down |
| MELO3C020975.2 | 108 | 26 | -2.06 | 1.54612E-06 | down |
| MELO3C020979.2 | 925 | 172 | -2.423 | 2.67003E-56 | down |
| MELO3C020980.2 | 1308 | 3109 | 1.249 | 9.34196E-38 | up |
| MELO3C020982.2 | 130 | 4 | -4.895 | 8.73301E-23 | down |
| MELO3C020984.2 | 473 | 10 | -5.499 | 1.08488E-62 | down |
| MELO3C020997.2 | 108 | 512 | 2.246 | 2.56006E-24 | up |
| MELO3C020998.2 | 509 | 131 | -1.954 | 3.8587E-30 | down |
| MELO3C021000.2 | 10 | 53 | 2.314 | 0.000158144 | up |
| MELO3C021005.2 | 0 | 1542 | 12.869 | 1.57607E-27 | up |
| MELO3C021012.2 | 305 | 46 | -2.743 | 1.93042E-36 | down |
| MELO3C021014.2 | 6 | 60 | 3.254 | 3.97139E-08 | up |
| MELO3C021015.2 | 0 | 19 | 6.508 | 9.01106E-06 | up |
| MELO3C021031.2 | 205 | 1634 | 2.99 | 9.96178E-69 | up |
| MELO3C021033.2 | 237 | 604 | 1.347 | 3.84256E-23 | up |
| MELO3C021047.2 | 783 | 233 | -1.746 | 2.98016E-26 | down |
| MELO3C021050.2 | 0 | 125 | 9.243 | 2.8259E-14 | up |
| MELO3C021058.2 | 10 | 135 | 3.743 | 7.0337E-18 | up |
| MELO3C021060.2 | 276 | 27 | -3.379 | 8.76229E-44 | down |
| MELO3C021064.2 | 4184 | 787 | -2.409 | 5.29024E-61 | down |
| MELO3C021069.2 | 0 | 84 | 8.676 | 4.84086E-12 | up |
| MELO3C021074.2 | 12996 | 3311 | -1.973 | 1.56679E-140 | down |
| MELO3C021075.2 | 8657 | 2780 | -1.639 | 2.6947E-116 | down |
| MELO3C021077.2 | 827 | 2671 | 1.692 | 2.50574E-42 | up |
| MELO3C021079.2 | 149 | 1945 | 3.709 | 2.37756E-139 | up |
| MELO3C021083.2 | 232 | 760 | 1.708 | 2.6208E-41 | up |
| MELO3C021084.2 | 2133 | 4441 | 1.058 | 7.63472E-23 | up |
| MELO3C021091.2 | 1160 | 347 | -1.742 | 1.15519E-15 | down |
| MELO3C021100.2 | 2 | 1458 | 9.609 | 1.141E-48 | up |
| MELO3C021106.2 | 137 | 362 | 1.407 | 9.2964E-12 | up |
| MELO3C021111.2 | 26 | 11 | -1.339 | 0.016224657 | down |
| MELO3C021118.2 | 543 | 2260 | 2.057 | 3.25119E-65 | up |
| MELO3C021125.2 | 878 | 3068 | 1.803 | 1.82441E-79 | up |
| MELO3C021137.2 | 29 | 862 | 4.907 | 1.11544E-111 | up |
| MELO3C021142.2 | 12 | 0 | -6.221 | 6.24637E-05 | down |
| MELO3C021143.2 | 1731 | 23 | -6.257 | 1.45677E-24 | down |
| MELO3C021144.2 | 123 | 391 | 1.669 | 9.91413E-17 | up |
| MELO3C021148.2 | 2369 | 973 | -1.284 | 3.03833E-55 | down |
| MELO3C021151.2 | 102 | 243 | 1.258 | 1.18116E-07 | up |
| MELO3C021153.2 | 5138 | 1345 | -1.933 | 7.07572E-144 | down |
| MELO3C021155.2 | 1162 | 183 | -2.669 | 9.91044E-42 | down |
| MELO3C021156.2 | 8 | 114 | 3.804 | 1.49609E-19 | up |
| MELO3C021167.2 | 465 | 149 | -1.639 | 2.16032E-14 | down |
| MELO3C021170.2 | 1744 | 669 | -1.382 | 8.76913E-43 | down |
| MELO3C021171.2 | 64945 | 28626 | -1.182 | 1.23557E-51 | down |
| MELO3C021176.2 | 11360 | 1639 | -2.793 | 9.44519E-138 | down |
| MELO3C021178.2 | 2 | 33 | 4.153 | 1.23363E-06 | up |
| MELO3C021180.2 | 18 | 76 | 2.069 | 2.98673E-06 | up |
| MELO3C021182.2 | 316 | 3 | -7.053 | 2.04698E-34 | down |
| MELO3C021185.2 | 1083 | 337 | -1.685 | 5.32115E-48 | down |
| MELO3C021190.2 | 81 | 194 | 1.254 | 4.276E-07 | up |
| MELO3C021191.2 | 0 | 8 | 5.215 | 0.002041102 | up |
| MELO3C021193.2 | 8 | 153 | 4.207 | 3.70566E-21 | up |
| MELO3C021194.2 | 227 | 490 | 1.109 | 9.88539E-13 | up |
| MELO3C021195.2 | 0 | 5 | 4.562 | 0.018235455 | up |
| MELO3C021196.2 | 9 | 29 | 1.684 | 0.020941869 | up |
| MELO3C021206.2 | 509 | 5797 | 3.509 | 5.93937E-26 | up |
| MELO3C021211.2 | 11 | 82 | 2.896 | 2.79016E-07 | up |
| MELO3C021216.2 | 768 | 1769 | 1.205 | 1.12667E-38 | up |
| MELO3C021226.2 | 2644 | 1126 | -1.231 | 1.80479E-31 | down |
| MELO3C021231.2 | 10817 | 4713 | -1.199 | 3.02488E-27 | down |
| MELO3C021235.2 | 274 | 133 | -1.04 | 5.0238E-06 | down |
| MELO3C021238.2 | 10 | 32 | 1.778 | 0.00208287 | up |
| MELO3C021240.2 | 760 | 1826 | 1.264 | 1.16316E-20 | up |
| MELO3C021245.2 | 226 | 57 | -1.989 | 1.20528E-17 | down |
| MELO3C021249.2 | 17236 | 5895 | -1.548 | 2.73581E-07 | down |
| MELO3C021250.2 | 2791 | 987 | -1.499 | 5.52996E-36 | down |
| MELO3C021253.2 | 11506 | 3877 | -1.569 | 2.1004E-94 | down |
| MELO3C021254.2 | 4469 | 2210 | -1.016 | 5.7345E-29 | down |
| MELO3C021255.2 | 1112 | 463 | -1.264 | 2.28675E-36 | down |
| MELO3C021273.2 | 210 | 427 | 1.025 | 1.48391E-10 | up |
| MELO3C021279.2 | 55 | 116 | 1.065 | 0.000164376 | up |
| MELO3C021281.2 | 9019 | 2394 | -1.914 | 5.10867E-41 | down |
| MELO3C021282.2 | 22 | 1383 | 5.947 | 6.89696E-161 | up |
| MELO3C021285.2 | 262 | 605 | 1.209 | 2.30203E-11 | up |
| MELO3C021300.2 | 6307 | 17546 | 1.476 | 7.94702E-47 | up |
| MELO3C021303.2 | 289 | 626 | 1.116 | 1.30824E-17 | up |
| MELO3C021309.2 | 15 | 0 | -6.482 | 1.20958E-05 | down |
| MELO3C021310.2 | 243 | 31 | -2.959 | 9.65806E-22 | down |
| MELO3C021322.2 | 31 | 564 | 4.188 | 1.39124E-63 | up |
| MELO3C021326.2 | 1143 | 173 | -2.722 | 1.9233E-71 | down |
| MELO3C021333.2 | 1315 | 3677 | 1.482 | 5.3521E-49 | up |
| MELO3C021334.2 | 110 | 0 | -9.39 | 1.89153E-14 | down |
| MELO3C021350.2 | 184 | 8 | -4.499 | 2.56372E-29 | down |
| MELO3C021351.2 | 61 | 2 | -5.344 | 6.75422E-12 | down |
| MELO3C021358.2 | 37 | 10 | -1.936 | 8.95103E-05 | down |
| MELO3C021370.2 | 356 | 101 | -1.812 | 2.45621E-24 | down |
| MELO3C021375.2 | 37125 | 7915 | -2.23 | 4.34515E-68 | down |
| MELO3C021379.2 | 724 | 226 | -1.675 | 1.46403E-15 | down |
| MELO3C021404.2 | 5232 | 11239 | 1.103 | 1.89205E-31 | up |
| MELO3C021406.2 | 3693 | 1024 | -1.852 | 2.81275E-53 | down |
| MELO3C021407.2 | 28037 | 6682 | -2.069 | 1.93582E-145 | down |
| MELO3C021412.2 | 3951 | 18 | -7.849 | 6.94305E-278 | down |
| MELO3C021418.2 | 47 | 21 | -1.127 | 0.00523002 | down |
| MELO3C021427.2 | 141 | 25 | -2.483 | 3.3566E-13 | down |
| MELO3C021428.2 | 134 | 35 | -1.94 | 2.57092E-06 | down |
| MELO3C021431.2 | 44 | 7 | -2.621 | 0.004394459 | down |
| MELO3C021433.2 | 5649 | 2566 | -1.139 | 2.75334E-18 | down |
| MELO3C021434.2 | 229 | 1075 | 2.235 | 6.74037E-38 | up |
| MELO3C021436.2 | 1670 | 3638 | 1.123 | 1.8798E-37 | up |
| MELO3C021437.2 | 140 | 750 | 2.424 | 8.88606E-21 | up |
| MELO3C021447.2 | 30 | 412 | 3.771 | 3.04837E-31 | up |
| MELO3C021449.2 | 1084 | 499 | -1.119 | 5.60372E-18 | down |
| MELO3C021451.2 | 180 | 31 | -2.555 | 3.73464E-20 | down |
| MELO3C021453.2 | 4542 | 1846 | -1.3 | 6.07397E-43 | down |
| MELO3C021455.2 | 19244 | 6354 | -1.599 | 6.03153E-60 | down |
| MELO3C021457.2 | 594 | 1394 | 1.231 | 2.01959E-19 | up |
| MELO3C021458.2 | 395 | 16722 | 5.406 | 6.93474E-235 | up |
| MELO3C021465.2 | 1594 | 5088 | 1.675 | 6.14887E-113 | up |
| MELO3C021466.2 | 4 | 17 | 1.914 | 0.016922584 | up |
| MELO3C021473.2 | 185 | 42 | -2.148 | 8.16805E-06 | down |
| MELO3C021483.2 | 282 | 865 | 1.617 | 4.84311E-33 | up |
| MELO3C021486.2 | 11 | 44 | 1.978 | 0.000103443 | up |
| MELO3C021488.2 | 317 | 107 | -1.57 | 3.26216E-17 | down |
| MELO3C021491.2 | 447 | 25 | -4.134 | 1.73982E-59 | down |
| MELO3C021499.2 | 52 | 13 | -1.975 | 4.87792E-05 | down |
| MELO3C021510.2 | 4477 | 2154 | -1.056 | 2.50951E-24 | down |
| MELO3C021528.2 | 1053 | 146 | -2.848 | 2.41348E-84 | down |
| MELO3C021532.2 | 370 | 770 | 1.06 | 1.25305E-11 | up |
| MELO3C021535.2 | 1267 | 596 | -1.088 | 4.17201E-23 | down |
| MELO3C021542.2 | 136 | 687 | 2.337 | 6.87847E-38 | up |
| MELO3C021545.2 | 84 | 190 | 1.18 | 1.26181E-07 | up |
| MELO3C021547.2 | 4579 | 1758 | -1.382 | 9.87443E-46 | down |
| MELO3C021552.2 | 899 | 2204 | 1.294 | 3.37824E-33 | up |
| MELO3C021553.2 | 2657 | 104 | -4.684 | 1.30546E-143 | down |
| MELO3C021554.2 | 3099 | 1249 | -1.311 | 1.52784E-49 | down |
| MELO3C021559.2 | 4 | 0 | -4.705 | 0.027910731 | down |
| MELO3C021569.2 | 1311 | 425 | -1.626 | 4.55188E-58 | down |
| MELO3C021570.2 | 82 | 33 | -1.339 | 0.000125381 | down |
| MELO3C021571.2 | 51 | 115 | 1.161 | 1.74618E-05 | up |
| MELO3C021579.2 | 8 | 91 | 3.604 | 2.82282E-15 | up |
| MELO3C021597.2 | 119 | 431 | 1.852 | 5.38474E-22 | up |
| MELO3C021598.2 | 805 | 1790 | 1.152 | 3.86297E-28 | up |
| MELO3C021603.2 | 1178 | 536 | -1.135 | 4.68561E-25 | down |
| MELO3C021604.2 | 242 | 489 | 1.014 | 2.86485E-05 | up |
| MELO3C021608.2 | 248 | 84 | -1.555 | 3.17248E-10 | down |
| MELO3C021609.2 | 6 | 177 | 4.981 | 4.73832E-29 | up |
| MELO3C021616.2 | 4964 | 1394 | -1.832 | 2.12913E-68 | down |
| MELO3C021623.2 | 5 | 0 | -5.025 | 0.010931185 | down |
| MELO3C021648.2 | 22542 | 6216 | -1.859 | 2.28077E-105 | down |
| MELO3C021658.2 | 22557 | 6007 | -1.909 | 5.26754E-94 | down |
| MELO3C021663.2 | 141 | 399 | 1.495 | 4.04168E-15 | up |
| MELO3C021682.2 | 369 | 1169 | 1.663 | 4.13746E-27 | up |
| MELO3C021688.2 | 6593 | 1713 | -1.944 | 2.07278E-31 | down |
| MELO3C021694.2 | 117 | 338 | 1.536 | 2.73333E-14 | up |
| MELO3C021703.2 | 74 | 417 | 2.486 | 7.39351E-22 | up |
| MELO3C021708.2 | 9883 | 482 | -4.357 | 1.26462E-291 | down |
| MELO3C021720.2 | 1537 | 3159 | 1.038 | 2.77803E-21 | up |
| MELO3C021727.2 | 746 | 4099 | 2.459 | 7.23435E-72 | up |
| MELO3C021744.2 | 557 | 161 | -1.795 | 1.80952E-21 | down |
| MELO3C021760.2 | 2880 | 67 | -5.419 | 2.38826E-45 | down |
| MELO3C021765.2 | 230 | 519 | 1.172 | 9.6269E-13 | up |
| MELO3C021766.2 | 6051 | 526 | -3.523 | 2.27793E-21 | down |
| MELO3C021767.2 | 2904 | 674 | -2.105 | 1.70798E-25 | down |
| MELO3C021768.2 | 166 | 30 | -2.469 | 1.68099E-17 | down |
| MELO3C021780.2 | 14 | 1 | -3.477 | 0.004025592 | down |
| MELO3C021781.2 | 76 | 6 | -3.727 | 1.0116E-05 | down |
| MELO3C021788.2 | 27632 | 1059 | -4.707 | 0 | down |
| MELO3C021789.2 | 22 | 6 | -1.786 | 0.008475681 | down |
| MELO3C021793.2 | 310 | 113 | -1.46 | 3.15357E-16 | down |
| MELO3C021797.2 | 19 | 45 | 1.305 | 0.003110933 | up |
| MELO3C021807.2 | 725 | 206 | -1.811 | 7.67932E-21 | down |
| MELO3C021811.2 | 1097 | 252 | -2.12 | 8.63903E-49 | down |
| MELO3C021820.2 | 28 | 847 | 4.939 | 5.80956E-103 | up |
| MELO3C021823.2 | 163 | 7451 | 5.517 | 9.06732E-44 | up |
| MELO3C021843.2 | 362 | 53 | -2.779 | 1.02029E-26 | down |
| MELO3C021846.2 | 143 | 781 | 2.443 | 9.49709E-44 | up |
| MELO3C021851.2 | 1278 | 2 | -9.083 | 1.05839E-66 | down |
| MELO3C021853.2 | 328 | 72 | -2.189 | 1.85298E-24 | down |
| MELO3C021858.2 | 5679 | 2551 | -1.155 | 1.20303E-59 | down |
| MELO3C021871.2 | 41 | 1 | -5.106 | 4.85421E-08 | down |
| MELO3C021877.2 | 169 | 0 | -10.015 | 1.02008E-16 | down |
| MELO3C021880.2 | 1771 | 878 | -1.012 | 4.08504E-31 | down |
| MELO3C021884.2 | 820 | 2865 | 1.805 | 1.29884E-69 | up |
| MELO3C021893.2 | 1022 | 2115 | 1.048 | 1.73291E-21 | up |
| MELO3C021896.2 | 25 | 0 | -7.28 | 6.96598E-08 | down |
| MELO3C021904.2 | 12 | 48 | 1.996 | 0.000457956 | up |
| MELO3C021914.2 | 211 | 9 | -4.518 | 5.32555E-25 | down |
| MELO3C021918.2 | 55 | 18 | -1.649 | 4.99494E-05 | down |
| MELO3C021919.2 | 864 | 2688 | 1.638 | 9.57954E-70 | up |
| MELO3C021920.2 | 108 | 40 | -1.406 | 1.23868E-06 | down |
| MELO3C021923.2 | 3650 | 423 | -3.107 | 9.28259E-110 | down |
| MELO3C021934.2 | 765 | 2572 | 1.749 | 3.46776E-48 | up |
| MELO3C021940.2 | 4433 | 2036 | -1.123 | 2.0128E-58 | down |
| MELO3C021941.2 | 2018 | 13549 | 2.747 | 5.57384E-61 | up |
| MELO3C021943.2 | 1173 | 2726 | 1.216 | 2.64935E-30 | up |
| MELO3C021945.2 | 65 | 146 | 1.157 | 6.58539E-05 | up |
| MELO3C021948.2 | 2 | 21 | 3.488 | 0.000514192 | up |
| MELO3C021949.2 | 0 | 5 | 4.551 | 0.020312255 | up |
| MELO3C021951.2 | 106 | 0 | -9.344 | 3.87198E-14 | down |
| MELO3C021952.2 | 868 | 2470 | 1.509 | 5.30419E-28 | up |
| MELO3C021955.2 | 90 | 4 | -4.653 | 2.43848E-16 | down |
| MELO3C021971.2 | 1896 | 377 | -2.329 | 8.10837E-88 | down |
| MELO3C021972.2 | 4733 | 1864 | -1.344 | 4.50076E-36 | down |
| MELO3C021980.2 | 882 | 0 | -11.439 | 4.76955E-22 | down |
| MELO3C021982.2 | 327 | 697 | 1.092 | 3.47963E-13 | up |
| MELO3C021987.2 | 3514 | 9815 | 1.482 | 9.59138E-60 | up |
| MELO3C021988.2 | 5093 | 1340 | -1.927 | 4.12868E-48 | down |
| MELO3C021993.2 | 580 | 27 | -4.442 | 2.5293E-08 | down |
| MELO3C021998.2 | 334 | 165 | -1.014 | 0.00020516 | down |
| MELO3C021999.2 | 6879 | 33867 | 2.299 | 2.69371E-189 | up |
| MELO3C022002.2 | 30 | 3 | -3.486 | 3.37792E-06 | down |
| MELO3C022005.2 | 1086 | 299 | -1.861 | 2.8398E-34 | down |
| MELO3C022006.2 | 34 | 12 | -1.572 | 0.01747445 | down |
| MELO3C022007.2 | 35 | 96 | 1.448 | 2.54777E-05 | up |
| MELO3C022015.2 | 1528 | 419 | -1.866 | 9.82566E-67 | down |
| MELO3C022021.2 | 4168 | 1646 | -1.34 | 2.28953E-27 | down |
| MELO3C022027.2 | 1068 | 2720 | 1.349 | 1.19097E-27 | up |
| MELO3C022028.2 | 0 | 17 | 6.384 | 1.1134E-05 | up |
| MELO3C022029.2 | 53 | 122 | 1.221 | 5.64501E-05 | up |
| MELO3C022034.2 | 36 | 100 | 1.467 | 1.48238E-06 | up |
| MELO3C022037.2 | 175 | 1343 | 2.939 | 1.72911E-105 | up |
| MELO3C022039.2 | 2 | 46 | 4.353 | 1.04318E-07 | up |
| MELO3C022040.2 | 996 | 210 | -2.242 | 1.11255E-36 | down |
| MELO3C022041.2 | 337 | 9 | -5.209 | 5.37514E-60 | down |
| MELO3C022045.2 | 2571 | 7341 | 1.514 | 3.14205E-60 | up |
| MELO3C022064.2 | 40 | 9 | -2.221 | 1.05315E-05 | down |
| MELO3C022068.2 | 428 | 912 | 1.091 | 3.37752E-22 | up |
| MELO3C022070.2 | 69 | 23 | -1.585 | 3.57047E-05 | down |
| MELO3C022076.2 | 278 | 54 | -2.376 | 8.4211E-28 | down |
| MELO3C022077.2 | 1474 | 595 | -1.31 | 8.79563E-32 | down |
| MELO3C022087.2 | 0 | 5 | 4.384 | 0.031474944 | up |
| MELO3C022088.2 | 2323 | 964 | -1.269 | 1.46595E-18 | down |
| MELO3C022089.2 | 68 | 146 | 1.11 | 1.43226E-05 | up |
| MELO3C022090.2 | 2665 | 613 | -2.118 | 1.68002E-60 | down |
| MELO3C022093.2 | 331 | 673 | 1.023 | 2.75399E-13 | up |
| MELO3C022095.2 | 2321 | 5319 | 1.197 | 2.15232E-32 | up |
| MELO3C022099.2 | 1056 | 525 | -1.007 | 9.03743E-24 | down |
| MELO3C022108.2 | 13 | 132 | 3.381 | 1.91397E-16 | up |
| MELO3C022113.2 | 42939 | 255 | -7.398 | 0 | down |
| MELO3C022116.2 | 248 | 92 | -1.438 | 2.09339E-10 | down |
| MELO3C022120.2 | 124 | 805 | 2.697 | 5.29733E-34 | up |
| MELO3C022121.2 | 6 | 0 | -5.14 | 0.006759837 | down |
| MELO3C022123.2 | 22 | 373 | 4.061 | 1.94265E-46 | up |
| MELO3C022140.2 | 1493 | 507 | -1.56 | 1.3111E-26 | down |
| MELO3C022141.2 | 132 | 50 | -1.415 | 8.26173E-07 | down |
| MELO3C022146.2 | 224 | 20 | -3.489 | 2.51811E-15 | down |
| MELO3C022148.2 | 91 | 454 | 2.322 | 4.22577E-38 | up |
| MELO3C022150.2 | 0 | 69 | 8.389 | 1.48702E-11 | up |
| MELO3C022152.2 | 0 | 365 | 10.792 | 1.32296E-19 | up |
| MELO3C022157.2 | 0 | 5 | 4.406 | 0.024606389 | up |
| MELO3C022162.2 | 19916 | 414 | -5.586 | 1.7675E-58 | down |
| MELO3C022176.2 | 1526 | 25535 | 4.065 | 6.47714E-305 | up |
| MELO3C022180.2 | 1358 | 352 | -1.946 | 3.47732E-21 | down |
| MELO3C022193.2 | 2328 | 464 | -2.328 | 1.69562E-78 | down |
| MELO3C022202.2 | 1311 | 18 | -6.213 | 2.80025E-175 | down |
| MELO3C022206.2 | 28 | 237 | 3.1 | 3.38243E-15 | up |
| MELO3C022207.2 | 14 | 85 | 2.585 | 6.99587E-11 | up |
| MELO3C022212.2 | 2743 | 1272 | -1.108 | 9.14826E-32 | down |
| MELO3C022214.2 | 405 | 959 | 1.245 | 6.58438E-22 | up |
| MELO3C022226.2 | 20847 | 10141 | -1.04 | 3.78777E-44 | down |
| MELO3C022228.2 | 3375 | 712 | -2.246 | 5.42733E-92 | down |
| MELO3C022236.2 | 2495 | 845 | -1.562 | 4.15408E-74 | down |
| MELO3C022240.2 | 642 | 0 | -11.943 | 9.99334E-24 | down |
| MELO3C022242.2 | 4699 | 1733 | -1.439 | 4.01677E-88 | down |
| MELO3C022246.2 | 7399 | 144 | -5.675 | 5.26958E-67 | down |
| MELO3C022249.2 | 462 | 185 | -1.317 | 5.37956E-10 | down |
| MELO3C022250.2 | 1 | 8 | 3.347 | 0.027380703 | up |
| MELO3C022251.2 | 30 | 6 | -2.285 | 0.000974959 | down |
| MELO3C022252.2 | 480 | 164 | -1.554 | 1.75925E-14 | down |
| MELO3C022264.2 | 2 | 147 | 6.045 | 2.60697E-16 | up |
| MELO3C022268.2 | 26 | 87 | 1.737 | 5.0004E-06 | up |
| MELO3C022272.2 | 309 | 690 | 1.157 | 1.27745E-18 | up |
| MELO3C022273.2 | 4350 | 1939 | -1.165 | 6.09062E-45 | down |
| MELO3C022278.2 | 2067 | 5319 | 1.364 | 1.66577E-59 | up |
| MELO3C022279.2 | 295 | 652 | 1.143 | 8.9385E-19 | up |
| MELO3C022281.2 | 0 | 8 | 5.316 | 0.001671276 | up |
| MELO3C022287.2 | 6 | 83 | 3.778 | 2.4058E-10 | up |
| MELO3C022291.2 | 1253 | 0 | -12.907 | 1.15681E-27 | down |
| MELO3C022295.2 | 271 | 1018 | 1.912 | 1.9504E-33 | up |
| MELO3C022299.2 | 747 | 253 | -1.566 | 3.06895E-23 | down |
| MELO3C022300.2 | 182 | 14 | -3.755 | 1.9009E-20 | down |
| MELO3C022303.2 | 7115 | 1972 | -1.851 | 8.06217E-74 | down |
| MELO3C022310.2 | 6072 | 2313 | -1.393 | 2.64393E-54 | down |
| MELO3C022315.2 | 132 | 814 | 2.62 | 4.93657E-21 | up |
| MELO3C022316.2 | 82 | 3 | -4.924 | 4.94169E-15 | down |
| MELO3C022317.2 | 5 | 0 | -4.865 | 0.016015479 | down |
| MELO3C022319.2 | 26 | 292 | 3.513 | 4.51628E-27 | up |
| MELO3C022321.2 | 3172 | 1523 | -1.058 | 1.31762E-35 | down |
| MELO3C022324.2 | 60 | 126 | 1.059 | 0.000572792 | up |
| MELO3C022334.2 | 1162 | 505 | -1.203 | 6.30492E-27 | down |
| MELO3C022335.2 | 5138 | 1677 | -1.615 | 1.1025E-98 | down |
| MELO3C022338.2 | 15 | 63 | 2.027 | 2.74652E-06 | up |
| MELO3C022342.2 | 4773 | 2229 | -1.099 | 5.71599E-21 | down |
| MELO3C022345.2 | 752 | 167 | -2.167 | 3.20623E-42 | down |
| MELO3C022353.2 | 68 | 166 | 1.275 | 1.72103E-08 | up |
| MELO3C022354.2 | 4112 | 204 | -4.332 | 0 | down |
| MELO3C022355.2 | 708 | 274 | -1.369 | 3.64901E-29 | down |
| MELO3C022356.2 | 5351 | 2302 | -1.217 | 5.89901E-28 | down |
| MELO3C022358.2 | 96 | 192 | 1.004 | 0.00056751 | up |
| MELO3C022359.2 | 1781 | 747 | -1.253 | 2.04277E-29 | down |
| MELO3C022362.2 | 5 | 37 | 2.855 | 2.83094E-05 | up |
| MELO3C022371.2 | 43 | 98 | 1.18 | 4.64445E-05 | up |
| MELO3C022375.2 | 33 | 12 | -1.512 | 0.004345101 | down |
| MELO3C022377.2 | 11 | 0 | -6.125 | 0.000758789 | down |
| MELO3C022391.2 | 102 | 306 | 1.586 | 1.85418E-15 | up |
| MELO3C022397.2 | 11 | 1207 | 6.755 | 6.26874E-114 | up |
| MELO3C022404.2 | 33 | 74 | 1.209 | 0.000681103 | up |
| MELO3C022409.2 | 169 | 341 | 1.017 | 3.46005E-10 | up |
| MELO3C022410.2 | 1482 | 142 | -3.391 | 5.44298E-46 | down |
| MELO3C022412.2 | 131 | 415 | 1.66 | 5.35746E-15 | up |
| MELO3C022417.2 | 525 | 63 | -3.069 | 1.01893E-63 | down |
| MELO3C022429.2 | 26 | 329 | 3.678 | 5.12834E-40 | up |
| MELO3C022430.2 | 219 | 2651 | 3.596 | 0.006037976 | up |
| MELO3C022433.2 | 138 | 568 | 2.04 | 0.00445646 | up |
| MELO3C022436.2 | 104 | 505 | 2.282 | 9.00755E-21 | up |
| MELO3C022437.2 | 1 | 23 | 3.956 | 5.65719E-05 | up |
| MELO3C022447.2 | 64 | 257 | 2.004 | 9.74515E-17 | up |
| MELO3C022457.2 | 1241 | 3433 | 1.468 | 6.99353E-24 | up |
| MELO3C022458.2 | 0 | 7 | 5.006 | 0.004246336 | up |
| MELO3C022459.2 | 35 | 153 | 2.121 | 5.36656E-11 | up |
| MELO3C022464.2 | 135 | 11 | -3.703 | 1.34628E-14 | down |
| MELO3C022468.2 | 5 | 26 | 2.338 | 0.004375148 | up |
| MELO3C022470.2 | 1 | 64 | 6.43 | 1.54061E-08 | up |
| MELO3C022473.2 | 6 | 18 | 1.72 | 0.016818474 | up |
| MELO3C022483.2 | 1395 | 240 | -2.537 | 5.23763E-79 | down |
| MELO3C022485.2 | 4931 | 1220 | -2.015 | 1.25633E-106 | down |
| MELO3C022487.2 | 27 | 12 | -1.203 | 0.022016141 | down |
| MELO3C022499.2 | 1955 | 679 | -1.527 | 4.28519E-26 | down |
| MELO3C022501.2 | 0 | 115 | 9.129 | 8.51197E-14 | up |
| MELO3C022506.2 | 189 | 77 | -1.298 | 3.5169E-07 | down |
| MELO3C022507.2 | 128 | 1234 | 3.271 | 7.00816E-79 | up |
| MELO3C022512.2 | 413 | 206 | -1.005 | 5.5537E-11 | down |
| MELO3C022513.2 | 61 | 30 | -1 | 0.005938434 | down |
| MELO3C022516.2 | 26 | 3 | -2.905 | 6.42374E-05 | down |
| MELO3C022517.2 | 1611 | 4454 | 1.467 | 4.21289E-84 | up |
| MELO3C022518.2 | 347 | 35 | -3.303 | 1.25876E-24 | down |
| MELO3C022520.2 | 398 | 157 | -1.345 | 4.16013E-17 | down |
| MELO3C022542.2 | 159 | 10 | -4.003 | 1.1735E-26 | down |
| MELO3C022568.2 | 944 | 54 | -4.122 | 3.9117E-111 | down |
| MELO3C022574.2 | 126 | 49 | -1.373 | 0.002431859 | down |
| MELO3C022577.2 | 5 | 0 | -4.874 | 0.012417496 | down |
| MELO3C022590.2 | 12 | 58 | 2.218 | 4.25886E-05 | up |
| MELO3C022596.2 | 29 | 82 | 1.483 | 0.000251731 | up |
| MELO3C022601.2 | 60 | 7 | -3.053 | 1.52306E-09 | down |
| MELO3C022602.2 | 288 | 96 | -1.581 | 1.30473E-16 | down |
| MELO3C022620.2 | 76 | 27 | -1.49 | 5.79376E-05 | down |
| MELO3C022631.2 | 699 | 0 | -12.065 | 3.78E-24 | down |
| MELO3C022641.2 | 60 | 23 | -1.378 | 0.001526779 | down |
| MELO3C022646.2 | 132 | 0 | -9.666 | 2.15286E-15 | down |
| MELO3C022653.2 | 2 | 11 | 2.236 | 0.029226268 | up |
| MELO3C022654.2 | 6487 | 2808 | -1.208 | 1.2328E-59 | down |
| MELO3C022665.2 | 27 | 119 | 2.139 | 2.5781E-07 | up |
| MELO3C022669.2 | 41 | 7 | -2.462 | 3.28298E-06 | down |
| MELO3C022671.2 | 7 | 146 | 4.309 | 3.86057E-17 | up |
| MELO3C022678.2 | 505 | 238 | -1.086 | 2.00725E-14 | down |
| MELO3C022688.2 | 72 | 30 | -1.237 | 0.000228066 | down |
| MELO3C022697.2 | 24 | 64 | 1.394 | 0.000592812 | up |
| MELO3C022698.2 | 0 | 7 | 5.117 | 0.014797734 | up |
| MELO3C022722.2 | 4170 | 1654 | -1.333 | 4.88231E-58 | down |
| MELO3C022727.2 | 26 | 163 | 2.677 | 2.3391E-16 | up |
| MELO3C022731.2 | 1 | 52 | 6.119 | 7.33835E-08 | up |
| MELO3C022734.2 | 1344 | 87 | -3.948 | 2.63936E-89 | down |
| MELO3C022736.2 | 72 | 239 | 1.741 | 5.13245E-16 | up |
| MELO3C022738.2 | 360 | 1985 | 2.465 | 1.85004E-50 | up |
| MELO3C022746.2 | 5895 | 1214 | -2.279 | 2.16592E-123 | down |
| MELO3C022748.2 | 32 | 6 | -2.349 | 0.000184063 | down |
| MELO3C022753.2 | 3498 | 8929 | 1.352 | 1.38507E-43 | up |
| MELO3C022773.2 | 197 | 28 | -2.81 | 1.5519E-23 | down |
| MELO3C022791.2 | 159 | 67 | -1.268 | 0.000173884 | down |
| MELO3C022802.2 | 227 | 53 | -2.084 | 3.97618E-10 | down |
| MELO3C022819.2 | 795 | 1771 | 1.157 | 7.77641E-22 | up |
| MELO3C022820.2 | 246 | 65 | -1.924 | 1.46372E-15 | down |
| MELO3C022828.2 | 4493 | 173 | -4.691 | 2.94552E-179 | down |
| MELO3C022834.2 | 1180 | 258 | -2.193 | 7.69319E-87 | down |
| MELO3C022835.2 | 0 | 5 | 4.679 | 0.01110219 | up |
| MELO3C022843.2 | 0 | 17 | 6.302 | 1.4019E-05 | up |
| MELO3C022844.2 | 0 | 88 | 8.737 | 1.34317E-12 | up |
| MELO3C022852.2 | 137 | 369 | 1.431 | 1.25587E-15 | up |
| MELO3C022910.2 | 136 | 272 | 1.004 | 1.16194E-06 | up |
| MELO3C022917.2 | 270 | 1317 | 2.287 | 5.01653E-40 | up |
| MELO3C022935.2 | 55 | 3 | -4.405 | 2.7695E-10 | down |
| MELO3C022936.2 | 1463 | 595 | -1.298 | 5.58891E-28 | down |
| MELO3C022941.2 | 3051 | 1137 | -1.425 | 6.84065E-68 | down |
| MELO3C022951.2 | 5 | 0 | -4.916 | 0.030512151 | down |
| MELO3C022954.2 | 10 | 59 | 2.528 | 3.56669E-06 | up |
| MELO3C022955.2 | 16 | 1 | -4.72 | 0.000428908 | down |
| MELO3C022961.2 | 3250 | 1374 | -1.243 | 6.22134E-44 | down |
| MELO3C022970.2 | 2722 | 1210 | -1.17 | 1.28449E-20 | down |
| MELO3C022979.2 | 108 | 280 | 1.385 | 2.51399E-12 | up |
| MELO3C022987.2 | 343 | 23 | -3.907 | 0.000413682 | down |
| MELO3C022989.2 | 0 | 6 | 4.778 | 0.032031905 | up |
| MELO3C022991.2 | 3 | 2018 | 9.592 | 1.10497E-64 | up |
| MELO3C022997.2 | 63 | 131 | 1.067 | 5.01164E-05 | up |
| MELO3C023001.2 | 3403 | 8758 | 1.364 | 1.38206E-51 | up |
| MELO3C023002.2 | 0 | 6 | 4.832 | 0.010067996 | up |
| MELO3C023008.2 | 27 | 6 | -2.266 | 0.001510922 | down |
| MELO3C023011.2 | 44 | 228 | 2.363 | 2.00771E-11 | up |
| MELO3C023013.2 | 4 | 0 | -4.806 | 0.015558572 | down |
| MELO3C023017.2 | 136 | 550 | 2.019 | 2.6402E-25 | up |
| MELO3C023027.2 | 16 | 342 | 4.391 | 4.59716E-29 | up |
| MELO3C023031.2 | 0 | 5 | 4.573 | 0.016499837 | up |
| MELO3C023032.2 | 477 | 46 | -3.388 | 1.4891E-25 | down |
| MELO3C023036.2 | 141 | 295 | 1.063 | 3.39711E-05 | up |
| MELO3C023041.2 | 560 | 30 | -4.206 | 1.78398E-69 | down |
| MELO3C023043.2 | 2 | 28 | 3.904 | 8.41447E-05 | up |
| MELO3C023045.2 | 97 | 35 | -1.475 | 7.18647E-06 | down |
| MELO3C023046.2 | 8 | 29 | 1.789 | 0.006397834 | up |
| MELO3C023047.2 | 360 | 922 | 1.354 | 3.16859E-21 | up |
| MELO3C023065.2 | 31 | 469 | 3.92 | 3.37978E-67 | up |
| MELO3C023067.2 | 2542 | 273 | -3.215 | 4.93129E-120 | down |
| MELO3C023069.2 | 1455 | 627 | -1.214 | 3.11642E-19 | down |
| MELO3C023086.2 | 5442 | 1037 | -2.392 | 9.77245E-70 | down |
| MELO3C023087.2 | 30 | 6 | -2.303 | 0.000350999 | down |
| MELO3C023093.2 | 0 | 27 | 7.015 | 4.20073E-07 | up |
| MELO3C023105.2 | 1554 | 707 | -1.136 | 9.44889E-25 | down |
| MELO3C023114.2 | 3317 | 7158 | 1.11 | 0.000164477 | up |
| MELO3C023118.2 | 0 | 18 | 6.413 | 5.7664E-06 | up |
| MELO3C023128.2 | 66 | 248 | 1.9 | 1.11046E-14 | up |
| MELO3C023131.2 | 58950 | 5839 | -3.336 | 6.46115E-116 | down |
| MELO3C023133.2 | 89 | 202 | 1.188 | 5.73677E-05 | up |
| MELO3C023150.2 | 1753 | 801 | -1.13 | 4.27492E-31 | down |
| MELO3C023156.2 | 0 | 11 | 5.735 | 0.000188718 | up |
| MELO3C023166.2 | 317 | 55 | -2.511 | 1.52353E-17 | down |
| MELO3C023168.2 | 536 | 1177 | 1.134 | 1.87838E-21 | up |
| MELO3C023173.2 | 16 | 85 | 2.39 | 1.17188E-09 | up |
| MELO3C023180.2 | 7 | 168 | 4.561 | 2.09204E-22 | up |
| MELO3C023188.2 | 636 | 2495 | 1.973 | 2.43119E-74 | up |
| MELO3C023190.2 | 7714 | 2389 | -1.692 | 1.91419E-80 | down |
| MELO3C023192.2 | 19 | 62 | 1.721 | 3.77568E-05 | up |
| MELO3C023197.2 | 1607 | 565 | -1.51 | 3.34871E-31 | down |
| MELO3C023200.2 | 650 | 182 | -1.833 | 4.74353E-26 | down |
| MELO3C023203.2 | 94 | 435 | 2.204 | 0.000310412 | up |
| MELO3C023220.2 | 535 | 3105 | 2.536 | 7.19458E-10 | up |
| MELO3C023230.2 | 41 | 2 | -4.515 | 9.27603E-09 | down |
| MELO3C023231.2 | 13 | 1 | -3.366 | 0.002607092 | down |
| MELO3C023232.2 | 479 | 215 | -1.158 | 2.10697E-10 | down |
| MELO3C023234.2 | 200 | 58 | -1.801 | 6.1222E-09 | down |
| MELO3C023240.2 | 17 | 42 | 1.26 | 0.006119806 | up |
| MELO3C023241.2 | 10 | 159 | 4.036 | 8.75204E-26 | up |
| MELO3C023245.2 | 36 | 2 | -4.343 | 3.0984E-07 | down |
| MELO3C023252.2 | 50 | 315 | 2.663 | 2.26546E-28 | up |
| MELO3C023253.2 | 82 | 644 | 2.969 | 3.32251E-56 | up |
| MELO3C023255.2 | 1875 | 135 | -3.792 | 6.87551E-79 | down |
| MELO3C023263.2 | 46 | 12 | -1.985 | 0.000838054 | down |
| MELO3C023268.2 | 338 | 159 | -1.093 | 8.83036E-09 | down |
| MELO3C023270.2 | 246 | 48 | -2.35 | 3.20978E-16 | down |
| MELO3C023272.2 | 3053 | 625 | -2.289 | 7.63481E-05 | down |
| MELO3C023285.2 | 0 | 7 | 4.982 | 0.004131872 | up |
| MELO3C023286.2 | 0 | 24 | 6.858 | 5.83328E-07 | up |
| MELO3C023299.2 | 6 | 0 | -5.2 | 0.003822913 | down |
| MELO3C023304.2 | 281 | 684 | 1.287 | 2.96839E-12 | up |
| MELO3C023313.2 | 234 | 852 | 1.866 | 0.000198474 | up |
| MELO3C023316.2 | 0 | 30 | 7.166 | 6.65003E-08 | up |
| MELO3C023320.2 | 0 | 86 | 8.698 | 1.51637E-11 | up |
| MELO3C023335.2 | 7 | 48 | 2.923 | 1.10322E-07 | up |
| MELO3C023342.2 | 196 | 1559 | 2.995 | 3.04091E-46 | up |
| MELO3C023349.2 | 15 | 41 | 1.453 | 0.002138783 | up |
| MELO3C023351.2 | 872 | 1900 | 1.125 | 9.4829E-31 | up |
| MELO3C023354.2 | 69172 | 18040 | -1.939 | 1.69912E-95 | down |
| MELO3C023361.2 | 11 | 69 | 2.672 | 1.01534E-07 | up |
| MELO3C023377.2 | 0 | 16 | 6.251 | 1.49996E-05 | up |
| MELO3C023385.2 | 675 | 1577 | 1.224 | 5.3181E-24 | up |
| MELO3C023405.2 | 521 | 218 | -1.259 | 7.69458E-16 | down |
| MELO3C023407.2 | 3 | 120 | 5.517 | 2.17511E-18 | up |
| MELO3C023408.2 | 2381 | 357 | -2.739 | 2.89433E-131 | down |
| MELO3C023420.2 | 574 | 100 | -2.515 | 3.13648E-15 | down |
| MELO3C023425.2 | 851 | 4358 | 2.357 | 2.23527E-100 | up |
| MELO3C023426.2 | 973 | 2135 | 1.134 | 2.29312E-24 | up |
| MELO3C023430.2 | 34 | 72 | 1.113 | 0.001449461 | up |
| MELO3C023431.2 | 3910 | 310 | -3.659 | 9.9821E-194 | down |
| MELO3C023439.2 | 6 | 0 | -5.191 | 0.003981256 | down |
| MELO3C023440.2 | 72 | 0 | -8.777 | 1.80609E-12 | down |
| MELO3C023441.2 | 168 | 2 | -6.349 | 1.65766E-24 | down |
| MELO3C023442.2 | 1915 | 618 | -1.632 | 1.60365E-53 | down |
| MELO3C023447.2 | 14 | 2 | -2.562 | 0.005426852 | down |
| MELO3C023457.2 | 135 | 273 | 1.01 | 4.06437E-07 | up |
| MELO3C023460.2 | 19 | 60 | 1.655 | 0.001309681 | up |
| MELO3C023461.2 | 461 | 127 | -1.853 | 1.6267E-27 | down |
| MELO3C023465.2 | 15521 | 2862 | -2.439 | 1.92338E-10 | down |
| MELO3C023468.2 | 4267 | 1100 | -1.957 | 1.21006E-83 | down |
| MELO3C023470.2 | 242 | 39 | -2.615 | 1.38204E-28 | down |
| MELO3C023473.2 | 4140 | 1589 | -1.381 | 1.82738E-64 | down |
| MELO3C023478.2 | 84 | 228 | 1.44 | 6.96263E-12 | up |
| MELO3C023479.2 | 921 | 327 | -1.493 | 1.57846E-27 | down |
| MELO3C023483.2 | 4 | 0 | -4.782 | 0.014207142 | down |
| MELO3C023484.2 | 474 | 1421 | 1.584 | 4.8856E-20 | up |
| MELO3C023485.2 | 18 | 65 | 1.863 | 1.06368E-05 | up |
| MELO3C023490.2 | 501 | 3127 | 2.641 | 2.61217E-141 | up |
| MELO3C023491.2 | 586 | 250 | -1.232 | 1.21035E-16 | down |
| MELO3C023493.2 | 35 | 3 | -3.608 | 2.40274E-05 | down |
| MELO3C023496.2 | 101 | 584 | 2.537 | 8.8484E-50 | up |
| MELO3C023500.2 | 0 | 7 | 5.056 | 0.019496613 | up |
| MELO3C023503.2 | 70 | 7 | -3.243 | 2.95897E-11 | down |
| MELO3C023522.2 | 48 | 4 | -3.517 | 1.37871E-08 | down |
| MELO3C023523.2 | 1871 | 554 | -1.757 | 4.05189E-50 | down |
| MELO3C023527.2 | 157 | 1278 | 3.024 | 1.1476E-35 | up |
| MELO3C023532.2 | 1098 | 549 | -1.001 | 1.2314E-14 | down |
| MELO3C023535.2 | 0 | 8 | 4.197 | 0.010059046 | up |
| MELO3C023539.2 | 145 | 18 | -3.023 | 1.95352E-16 | down |
| MELO3C023540.2 | 70 | 5 | -3.878 | 8.2294E-11 | down |
| MELO3C023542.2 | 990 | 109 | -3.181 | 6.08127E-76 | down |
| MELO3C023545.2 | 174 | 550 | 1.654 | 5.57844E-28 | up |
| MELO3C023546.2 | 5 | 17 | 1.839 | 0.01595412 | up |
| MELO3C023550.2 | 105 | 275 | 1.393 | 7.60911E-11 | up |
| MELO3C023560.2 | 4350 | 2153 | -1.015 | 9.82382E-25 | down |
| MELO3C023563.2 | 314 | 59 | -2.429 | 7.13596E-28 | down |
| MELO3C023566.2 | 1 | 255 | 8.417 | 8.24685E-16 | up |
| MELO3C023567.2 | 2 | 23 | 3.596 | 6.44451E-05 | up |
| MELO3C023568.2 | 386 | 80 | -2.264 | 9.68333E-30 | down |
| MELO3C023570.2 | 304 | 1224 | 2.01 | 8.58761E-41 | up |
| MELO3C023571.2 | 255 | 713 | 1.485 | 2.12156E-15 | up |
| MELO3C023573.2 | 84 | 246 | 1.555 | 2.34337E-09 | up |
| MELO3C023578.2 | 104 | 262 | 1.325 | 2.0578E-07 | up |
| MELO3C023579.2 | 42 | 0 | -7.996 | 1.0496E-09 | down |
| MELO3C023580.2 | 951 | 449 | -1.082 | 3.68303E-21 | down |
| MELO3C023581.2 | 6690 | 14989 | 1.164 | 3.65224E-50 | up |
| MELO3C023582.2 | 1813 | 693 | -1.388 | 4.23942E-40 | down |
| MELO3C023586.2 | 713 | 3 | -8.094 | 2.50768E-30 | down |
| MELO3C023587.2 | 95 | 19 | -2.34 | 2.58913E-11 | down |
| MELO3C023589.2 | 7 | 25 | 1.733 | 0.005635773 | up |
| MELO3C023590.2 | 21841 | 10444 | -1.064 | 1.68566E-31 | down |
| MELO3C023591.2 | 1 | 67 | 5.505 | 6.05918E-11 | up |
| MELO3C023593.2 | 15 | 66 | 2.182 | 5.07557E-07 | up |
| MELO3C023594.2 | 1985 | 4212 | 1.085 | 9.58602E-29 | up |
| MELO3C023596.2 | 4307 | 766 | -2.492 | 2.3341E-91 | down |
| MELO3C023605.2 | 2148 | 384 | -2.484 | 1.35067E-98 | down |
| MELO3C023606.2 | 12394 | 33186 | 1.421 | 1.31944E-48 | up |
| MELO3C023609.2 | 2649 | 707 | -1.904 | 2.90272E-60 | down |
| MELO3C023610.2 | 500 | 1011 | 1.013 | 1.97809E-16 | up |
| MELO3C023618.2 | 14 | 38 | 1.458 | 0.004336631 | up |
| MELO3C023620.2 | 1 | 97 | 6.023 | 1.97857E-13 | up |
| MELO3C023621.2 | 62 | 0 | -8.582 | 6.52868E-12 | down |
| MELO3C023630.2 | 4819 | 10005 | 1.054 | 6.63629E-33 | up |
| MELO3C023633.2 | 441 | 6 | -6.089 | 4.34989E-66 | down |
| MELO3C023654.2 | 19 | 157 | 3.07 | 7.13254E-20 | up |
| MELO3C023667.2 | 1340 | 660 | -1.021 | 1.22702E-18 | down |
| MELO3C023673.2 | 257 | 627 | 1.286 | 1.59099E-18 | up |
| MELO3C023678.2 | 409 | 1901 | 2.217 | 1.0393E-09 | up |
| MELO3C023684.2 | 2629 | 1036 | -1.343 | 2.63318E-45 | down |
| MELO3C023714.2 | 317 | 52 | -2.602 | 3.15845E-17 | down |
| MELO3C023724.2 | 4108 | 1930 | -1.09 | 3.54498E-25 | down |
| MELO3C023727.2 | 35 | 120 | 1.783 | 9.90647E-09 | up |
| MELO3C023762.2 | 13 | 30 | 1.22 | 0.026301722 | up |
| MELO3C023770.2 | 108 | 8 | -3.726 | 7.37805E-19 | down |
| MELO3C023777.2 | 261 | 740 | 1.504 | 3.78037E-25 | up |
| MELO3C023781.2 | 2109 | 169 | -3.646 | 2.9217E-74 | down |
| MELO3C023788.2 | 3051 | 786 | -1.957 | 2.38899E-94 | down |
| MELO3C023790.2 | 967 | 135 | -2.844 | 6.82005E-87 | down |
| MELO3C023794.2 | 15 | 68 | 2.191 | 2.24064E-07 | up |
| MELO3C023799.2 | 0 | 28 | 7.061 | 1.12428E-07 | up |
| MELO3C023804.2 | 2000 | 4730 | 1.241 | 2.71736E-42 | up |
| MELO3C023809.2 | 6012 | 2962 | -1.021 | 1.6483E-40 | down |
| MELO3C023810.2 | 57 | 16 | -1.825 | 7.32565E-06 | down |
| MELO3C023814.2 | 1 | 44 | 4.895 | 3.0467E-08 | up |
| MELO3C023822.2 | 316 | 768 | 1.281 | 9.72808E-14 | up |
| MELO3C023827.2 | 263 | 0 | -10.658 | 8.89297E-19 | down |
| MELO3C023833.2 | 48 | 8 | -2.591 | 1.36179E-05 | down |
| MELO3C023842.2 | 247 | 581 | 1.234 | 5.25632E-06 | up |
| MELO3C023843.2 | 305 | 35 | -3.103 | 7.17234E-18 | down |
| MELO3C023848.2 | 59 | 206 | 1.809 | 1.21848E-09 | up |
| MELO3C023849.2 | 1 | 114 | 7.25 | 1.07369E-11 | up |
| MELO3C023852.2 | 427 | 13 | -4.959 | 8.04215E-16 | down |
| MELO3C023853.2 | 493 | 151 | -1.703 | 5.55253E-12 | down |
| MELO3C023855.2 | 55 | 150 | 1.449 | 1.06049E-07 | up |
| MELO3C023856.2 | 27 | 64 | 1.256 | 0.000726746 | up |
| MELO3C023866.2 | 39 | 15 | -1.404 | 0.028612477 | down |
| MELO3C023876.2 | 36 | 4 | -3.253 | 8.97044E-06 | down |
| MELO3C023879.2 | 64985 | 18841 | -1.786 | 1.39088E-60 | down |
| MELO3C023881.2 | 42 | 331 | 2.968 | 4.7943E-40 | up |
| MELO3C023896.2 | 380 | 107 | -1.824 | 1.32827E-13 | down |
| MELO3C023900.2 | 0 | 84 | 8.672 | 1.91996E-12 | up |
| MELO3C023909.2 | 17 | 37 | 1.119 | 0.013150478 | up |
| MELO3C023917.2 | 0 | 13 | 5.982 | 0.000115128 | up |
| MELO3C023918.2 | 0 | 25 | 6.954 | 2.63394E-07 | up |
| MELO3C023928.2 | 61 | 11 | -2.446 | 9.01118E-08 | down |
| MELO3C023930.2 | 472 | 20 | -4.543 | 1.2917E-70 | down |
| MELO3C023931.2 | 0 | 808 | 11.937 | 7.35821E-24 | up |
| MELO3C023938.2 | 13 | 4 | -1.867 | 0.026064081 | down |
| MELO3C023944.2 | 35 | 109 | 1.634 | 3.82314E-07 | up |
| MELO3C023946.2 | 81 | 30 | -1.448 | 2.5371E-05 | down |
| MELO3C023972.2 | 59 | 230 | 1.967 | 2.62225E-16 | up |
| MELO3C023976.2 | 16 | 3 | -2.71 | 0.00560838 | down |
| MELO3C023981.2 | 43 | 19 | -1.222 | 0.004583978 | down |
| MELO3C023982.2 | 1593 | 478 | -1.738 | 3.13636E-62 | down |
| MELO3C023986.2 | 468 | 2 | -8.047 | 1.05013E-38 | down |
| MELO3C023987.2 | 78 | 418 | 2.432 | 3.62613E-16 | up |
| MELO3C023997.2 | 10110 | 3984 | -1.344 | 5.0981E-51 | down |
| MELO3C023998.2 | 322 | 154 | -1.065 | 2.57046E-07 | down |
| MELO3C024010.2 | 4970 | 812 | -2.615 | 7.10698E-192 | down |
| MELO3C024015.2 | 44 | 95 | 1.117 | 0.000644991 | up |
| MELO3C024016.2 | 1485 | 714 | -1.057 | 1.60781E-22 | down |
| MELO3C024017.2 | 8 | 0 | -5.609 | 0.001395231 | down |
| MELO3C024030.2 | 2933 | 8073 | 1.461 | 1.49033E-27 | up |
| MELO3C024033.2 | 139 | 52 | -1.429 | 5.4785E-07 | down |
| MELO3C024035.2 | 6 | 25 | 2.058 | 0.001311701 | up |
| MELO3C024045.2 | 1 | 161 | 7.759 | 1.97887E-13 | up |
| MELO3C024058.2 | 2488 | 1162 | -1.099 | 1.07392E-34 | down |
| MELO3C024061.2 | 2958 | 1441 | -1.038 | 4.70506E-10 | down |
| MELO3C024063.2 | 258 | 42 | -2.607 | 5.8286E-26 | down |
| MELO3C024080.2 | 312 | 3224 | 3.371 | 1.06389E-154 | up |
| MELO3C024086.2 | 9885 | 1576 | -2.65 | 1.88073E-105 | down |
| MELO3C024088.2 | 699 | 284 | -1.3 | 1.63748E-24 | down |
| MELO3C024092.2 | 186 | 8 | -4.588 | 1.33405E-28 | down |
| MELO3C024096.2 | 324 | 6 | -5.897 | 8.83541E-07 | down |
| MELO3C024098.2 | 424 | 866 | 1.031 | 3.98088E-21 | up |
| MELO3C024100.2 | 12 | 0 | -6.179 | 9.6503E-05 | down |
| MELO3C024102.2 | 321 | 1097 | 1.774 | 8.3502E-18 | up |
| MELO3C024107.2 | 674 | 69 | -3.298 | 2.24949E-77 | down |
| MELO3C024108.2 | 1006 | 6394 | 2.668 | 5.82676E-204 | up |
| MELO3C024121.2 | 3077 | 538 | -2.517 | 7.06198E-108 | down |
| MELO3C024127.2 | 110 | 496 | 2.172 | 1.99033E-36 | up |
| MELO3C024143.2 | 11 | 42 | 1.91 | 0.002340713 | up |
| MELO3C024146.2 | 106 | 653 | 2.62 | 3.29459E-47 | up |
| MELO3C024155.2 | 368 | 119 | -1.629 | 2.48683E-20 | down |
| MELO3C024163.2 | 202 | 613 | 1.605 | 1.03654E-15 | up |
| MELO3C024167.2 | 595 | 1269 | 1.092 | 1.5913E-14 | up |
| MELO3C024175.2 | 5853 | 1880 | -1.638 | 1.74602E-56 | down |
| MELO3C024176.2 | 28 | 86 | 1.64 | 6.14995E-05 | up |
| MELO3C024178.2 | 362 | 161 | -1.169 | 2.2931E-08 | down |
| MELO3C024185.2 | 1671 | 773 | -1.112 | 6.42842E-28 | down |
| MELO3C024188.2 | 2382 | 824 | -1.531 | 9.62484E-38 | down |
| MELO3C024192.2 | 1582 | 11888 | 2.91 | 8.1595E-89 | up |
| MELO3C024198.2 | 32 | 79 | 1.332 | 6.65426E-05 | up |
| MELO3C024199.2 | 6 | 42 | 2.73 | 1.85481E-06 | up |
| MELO3C024206.2 | 71023 | 4939 | -3.846 | 0 | down |
| MELO3C024213.2 | 241 | 747 | 1.633 | 1.12625E-35 | up |
| MELO3C024225.2 | 1280 | 3420 | 1.419 | 1.5038E-38 | up |
| MELO3C024228.2 | 2015 | 618 | -1.706 | 4.22861E-71 | down |
| MELO3C024231.2 | 63 | 327 | 2.382 | 1.39655E-26 | up |
| MELO3C024234.2 | 426 | 158 | -1.436 | 1.12366E-19 | down |
| MELO3C024235.2 | 31 | 7 | -2.117 | 0.000286807 | down |
| MELO3C024236.2 | 123 | 589 | 2.268 | 1.82593E-22 | up |
| MELO3C024238.2 | 5 | 22 | 2.164 | 0.014065401 | up |
| MELO3C024239.2 | 141 | 6 | -4.567 | 9.67348E-27 | down |
| MELO3C024241.2 | 53 | 924 | 4.139 | 2.54897E-66 | up |
| MELO3C024247.2 | 25 | 1515 | 5.919 | 1.32421E-150 | up |
| MELO3C024252.2 | 99 | 200 | 1.019 | 1.92816E-05 | up |
| MELO3C024255.2 | 3088 | 693 | -2.157 | 1.38798E-112 | down |
| MELO3C024256.2 | 101 | 234 | 1.218 | 4.76991E-05 | up |
| MELO3C024263.2 | 73 | 2 | -4.951 | 1.07214E-13 | down |
| MELO3C024264.2 | 470 | 80 | -2.573 | 1.59747E-26 | down |
| MELO3C024268.2 | 12 | 34 | 1.482 | 0.003691147 | up |
| MELO3C024278.2 | 284 | 698 | 1.296 | 7.57114E-22 | up |
| MELO3C024292.2 | 2231 | 5923 | 1.409 | 3.11897E-43 | up |
| MELO3C024303.2 | 325 | 1093 | 1.751 | 2.50513E-47 | up |
| MELO3C024311.2 | 14 | 3 | -2.265 | 0.018994127 | down |
| MELO3C024312.2 | 320 | 58 | -2.464 | 8.57663E-36 | down |
| MELO3C024314.2 | 1288 | 2862 | 1.153 | 2.57523E-14 | up |
| MELO3C024317.2 | 4450 | 1235 | -1.848 | 5.45265E-38 | down |
| MELO3C024318.2 | 134 | 12 | -3.448 | 3.67467E-19 | down |
| MELO3C024323.2 | 149 | 50 | -1.561 | 6.42467E-07 | down |
| MELO3C024324.2 | 1982 | 329 | -2.591 | 8.2185E-114 | down |
| MELO3C024326.2 | 25154 | 9840 | -1.354 | 6.33122E-46 | down |
| MELO3C024337.2 | 1027 | 4032 | 1.974 | 8.05199E-42 | up |
| MELO3C024339.2 | 13 | 2 | -2.466 | 0.018182804 | down |
| MELO3C024344.2 | 5376 | 1545 | -1.799 | 5.87709E-60 | down |
| MELO3C024346.2 | 4208 | 1815 | -1.214 | 7.26014E-17 | down |
| MELO3C024371.2 | 43 | 137 | 1.671 | 2.57173E-08 | up |
| MELO3C024374.2 | 155 | 341 | 1.136 | 5.92391E-11 | up |
| MELO3C024377.2 | 20 | 192 | 3.283 | 5.64473E-14 | up |
| MELO3C024387.2 | 10 | 129 | 3.729 | 5.8373E-20 | up |
| MELO3C024388.2 | 433 | 1643 | 1.923 | 7.53184E-81 | up |
| MELO3C024390.2 | 2070 | 5429 | 1.392 | 5.81834E-75 | up |
| MELO3C024396.2 | 70 | 14 | -2.301 | 5.1301E-05 | down |
| MELO3C024398.2 | 1526 | 251 | -2.604 | 1.37501E-48 | down |
| MELO3C024400.2 | 80 | 4 | -4.603 | 2.36431E-13 | down |
| MELO3C024405.2 | 158 | 448 | 1.507 | 4.50909E-21 | up |
| MELO3C024412.2 | 135 | 283 | 1.073 | 0.003513824 | up |
| MELO3C024420.2 | 14 | 1210 | 6.492 | 9.972E-131 | up |
| MELO3C024422.2 | 7 | 42 | 2.612 | 0.000102767 | up |
| MELO3C024425.2 | 101 | 46 | -1.133 | 0.000162974 | down |
| MELO3C024427.2 | 0 | 15 | 6.171 | 3.00403E-05 | up |
| MELO3C024431.2 | 2246 | 698 | -1.688 | 7.5642E-75 | down |
| MELO3C024434.2 | 92 | 15 | -2.6 | 1.56571E-11 | down |
| MELO3C024436.2 | 1888 | 587 | -1.687 | 2.59606E-52 | down |
| MELO3C024437.2 | 1131 | 2349 | 1.054 | 1.15942E-19 | up |
| MELO3C024439.2 | 6 | 27 | 2.169 | 0.002638759 | up |
| MELO3C024445.2 | 23 | 8 | -1.549 | 0.014513845 | down |
| MELO3C024459.2 | 87 | 289 | 1.734 | 3.25127E-19 | up |
| MELO3C024463.2 | 758 | 123 | -2.632 | 2.92263E-58 | down |
| MELO3C024464.2 | 7 | 1 | -2.978 | 0.028403236 | down |
| MELO3C024465.2 | 1390 | 601 | -1.211 | 1.86897E-26 | down |
| MELO3C024466.2 | 221 | 1764 | 2.998 | 1.3214E-51 | up |
| MELO3C024482.2 | 713 | 284 | -1.328 | 2.00665E-24 | down |
| MELO3C024486.2 | 723 | 174 | -2.059 | 8.18295E-54 | down |
| MELO3C024490.2 | 58 | 301 | 2.377 | 9.27084E-16 | up |
| MELO3C024493.2 | 0 | 11 | 5.667 | 0.000276427 | up |
| MELO3C024495.2 | 1738 | 816 | -1.091 | 1.63511E-22 | down |
| MELO3C024498.2 | 575 | 85 | -2.757 | 3.42581E-25 | down |
| MELO3C024508.2 | 174 | 1384 | 3.001 | 5.28077E-58 | up |
| MELO3C024510.2 | 280 | 562 | 1.008 | 2.70223E-11 | up |
| MELO3C024513.2 | 14 | 1632 | 6.847 | 8.94909E-24 | up |
| MELO3C024520.2 | 24 | 0 | -7.201 | 1.33711E-07 | down |
| MELO3C024523.2 | 42 | 2 | -4.36 | 1.7924E-08 | down |
| MELO3C024529.2 | 17 | 121 | 2.793 | 1.17731E-12 | up |
| MELO3C024530.2 | 611 | 2664 | 2.123 | 4.25306E-36 | up |
| MELO3C024531.2 | 898 | 2147 | 1.258 | 7.90162E-05 | up |
| MELO3C024532.2 | 647 | 144 | -2.173 | 3.66878E-15 | down |
| MELO3C024535.2 | 2 | 13 | 2.264 | 0.024153835 | up |
| MELO3C024539.2 | 206 | 1 | -7.461 | 9.35368E-22 | down |
| MELO3C024541.2 | 0 | 5 | 4.582 | 0.015389123 | up |
| MELO3C024545.2 | 463 | 1943 | 2.067 | 1.51836E-21 | up |
| MELO3C024549.2 | 381 | 1372 | 1.847 | 8.95966E-54 | up |
| MELO3C024550.2 | 7820 | 661 | -3.565 | 0 | down |
| MELO3C024552.2 | 1078 | 2830 | 1.392 | 9.25753E-43 | up |
| MELO3C024554.2 | 5068 | 1895 | -1.42 | 1.23344E-55 | down |
| MELO3C024557.2 | 323 | 59 | -2.451 | 2.68039E-23 | down |
| MELO3C024560.2 | 0 | 19 | 6.554 | 3.29213E-06 | up |
| MELO3C024569.2 | 121 | 247 | 1.03 | 1.1976E-06 | up |
| MELO3C024570.2 | 1144 | 461 | -1.313 | 3.97516E-23 | down |
| MELO3C024571.2 | 28 | 158 | 2.49 | 1.67642E-10 | up |
| MELO3C024574.2 | 12 | 43 | 1.839 | 0.000105041 | up |
| MELO3C024575.2 | 252 | 539 | 1.094 | 1.97213E-16 | up |
| MELO3C024579.2 | 787 | 219 | -1.845 | 6.12573E-44 | down |
| MELO3C024582.2 | 928 | 109 | -3.091 | 5.51855E-104 | down |
| MELO3C024585.2 | 11 | 2 | -2.643 | 0.031056483 | down |
| MELO3C024587.2 | 8279 | 4095 | -1.016 | 1.45543E-60 | down |
| MELO3C024602.2 | 284 | 584 | 1.042 | 6.3875E-13 | up |
| MELO3C024603.2 | 2200 | 5739 | 1.384 | 4.4132E-28 | up |
| MELO3C024605.2 | 5168 | 494 | -3.385 | 1.68989E-76 | down |
| MELO3C024610.2 | 15284 | 1658 | -3.204 | 3.43777E-100 | down |
| MELO3C024616.2 | 927 | 229 | -2.013 | 1.29458E-17 | down |
| MELO3C024627.2 | 8 | 23 | 1.491 | 0.018493679 | up |
| MELO3C024628.2 | 6 | 83 | 3.87 | 1.37041E-11 | up |
| MELO3C024629.2 | 0 | 58 | 8.14 | 8.37342E-11 | up |
| MELO3C024638.2 | 1 | 31 | 5.39 | 4.45381E-06 | up |
| MELO3C024660.2 | 464 | 1223 | 1.403 | 5.55464E-11 | up |
| MELO3C024664.2 | 2730 | 577 | -2.24 | 4.9988E-58 | down |
| MELO3C024669.2 | 978 | 1966 | 1.008 | 6.77168E-29 | up |
| MELO3C024673.2 | 887 | 5153 | 2.54 | 3.77806E-87 | up |
| MELO3C024674.2 | 10217 | 2987 | -1.774 | 1.12405E-81 | down |
| MELO3C024678.2 | 175 | 364 | 1.054 | 7.0008E-12 | up |
| MELO3C024679.2 | 1750 | 527 | -1.732 | 6.53335E-56 | down |
| MELO3C024683.2 | 252 | 122 | -1.052 | 8.31794E-09 | down |
| MELO3C024684.2 | 3319 | 6851 | 1.046 | 3.47805E-45 | up |
| MELO3C024699.2 | 616 | 3300 | 2.42 | 1.35745E-82 | up |
| MELO3C024701.2 | 10418 | 2582 | -2.013 | 6.42536E-92 | down |
| MELO3C024704.2 | 138 | 463 | 1.747 | 1.39712E-25 | up |
| MELO3C024728.2 | 419 | 961 | 1.199 | 8.91384E-22 | up |
| MELO3C024731.2 | 0 | 76 | 8.52 | 2.15109E-11 | up |
| MELO3C024736.2 | 70 | 655 | 3.235 | 7.83915E-54 | up |
| MELO3C024745.2 | 45 | 13 | -1.812 | 0.002573392 | down |
| MELO3C024750.2 | 317 | 715 | 1.175 | 4.66463E-15 | up |
| MELO3C024760.2 | 8196 | 1036 | -2.985 | 5.11805E-163 | down |
| MELO3C024766.2 | 50746 | 18357 | -1.467 | 5.70179E-21 | down |
| MELO3C024769.2 | 35 | 5 | -2.84 | 0.000522867 | down |
| MELO3C024771.2 | 70843 | 3506 | -4.337 | 6.81217E-49 | down |
| MELO3C024777.2 | 526 | 1317 | 1.324 | 1.59758E-33 | up |
| MELO3C024783.2 | 1114 | 2928 | 1.393 | 1.15176E-45 | up |
| MELO3C024796.2 | 208 | 1001 | 2.27 | 2.60144E-52 | up |
| MELO3C024799.2 | 5001 | 2031 | -1.3 | 1.91756E-24 | down |
| MELO3C024800.2 | 1616 | 740 | -1.127 | 6.59575E-24 | down |
| MELO3C024809.2 | 58 | 20 | -1.581 | 0.000289164 | down |
| MELO3C024816.2 | 49 | 5 | -3.372 | 8.28901E-05 | down |
| MELO3C024820.2 | 2494 | 353 | -2.821 | 1.10178E-12 | down |
| MELO3C024832.2 | 2 | 13 | 2.5 | 0.017333072 | up |
| MELO3C024834.2 | 20 | 0 | -6.956 | 5.53964E-07 | down |
| MELO3C024841.2 | 160 | 9 | -4.169 | 0.010336697 | down |
| MELO3C024842.2 | 51 | 0 | -8.282 | 6.51094E-09 | down |
| MELO3C024849.2 | 667 | 1680 | 1.333 | 3.14934E-42 | up |
| MELO3C024857.2 | 197 | 38 | -2.35 | 3.24663E-10 | down |
| MELO3C024871.2 | 1101 | 2552 | 1.212 | 1.4874E-34 | up |
| MELO3C024872.2 | 39 | 430 | 3.492 | 2.70578E-53 | up |
| MELO3C024877.2 | 71 | 1 | -5.92 | 1.39658E-12 | down |
| MELO3C024882.2 | 16 | 36 | 1.117 | 0.017295043 | up |
| MELO3C024885.2 | 1332 | 309 | -2.111 | 1.33852E-67 | down |
| MELO3C024886.2 | 242 | 29 | -3.073 | 0.001062079 | down |
| MELO3C024887.2 | 3213 | 1457 | -1.142 | 1.91865E-18 | down |
| MELO3C024898.2 | 9 | 35 | 1.939 | 0.000514451 | up |
| MELO3C024899.2 | 2974 | 694 | -2.1 | 1.41001E-88 | down |
| MELO3C024900.2 | 480 | 120 | -1.992 | 5.79792E-25 | down |
| MELO3C024918.2 | 122 | 257 | 1.08 | 3.45663E-08 | up |
| MELO3C024920.2 | 5808 | 549 | -3.403 | 2.00015E-101 | down |
| MELO3C024924.2 | 22 | 54 | 1.293 | 0.000995635 | up |
| MELO3C024925.2 | 847 | 160 | -2.4 | 1.61863E-29 | down |
| MELO3C024938.2 | 196 | 813 | 2.06 | 5.28105E-27 | up |
| MELO3C024959.2 | 846 | 405 | -1.065 | 1.80652E-16 | down |
| MELO3C024964.2 | 4508 | 1086 | -2.054 | 1.92681E-106 | down |
| MELO3C024972.2 | 111 | 297 | 1.424 | 1.3639E-12 | up |
| MELO3C024975.2 | 387 | 2706 | 2.806 | 1.10107E-116 | up |
| MELO3C024982.2 | 306 | 899 | 1.554 | 2.40236E-20 | up |
| MELO3C024990.2 | 348 | 779 | 1.163 | 3.9227E-19 | up |
| MELO3C025005.2 | 6382 | 729 | -3.131 | 4.62733E-149 | down |
| MELO3C025012.2 | 58 | 27 | -1.109 | 0.002433538 | down |
| MELO3C025019.2 | 1 | 16 | 4.424 | 0.000598501 | up |
| MELO3C025023.2 | 2938 | 1089 | -1.433 | 1.75539E-35 | down |
| MELO3C025026.2 | 1214 | 28 | -5.443 | 4.85684E-36 | down |
| MELO3C025027.2 | 41083 | 17498 | -1.231 | 1.60101E-40 | down |
| MELO3C025029.2 | 3251 | 24802 | 2.932 | 7.05603E-91 | up |
| MELO3C025034.2 | 1329 | 480 | -1.472 | 1.78909E-25 | down |
| MELO3C025049.2 | 99 | 312 | 1.662 | 8.12667E-18 | up |
| MELO3C025056.2 | 501 | 2419 | 2.273 | 6.50041E-59 | up |
| MELO3C025078.2 | 5546 | 11744 | 1.083 | 1.37686E-31 | up |
| MELO3C025079.2 | 554 | 8 | -6.19 | 3.26671E-70 | down |
| MELO3C025087.2 | 258 | 531 | 1.039 | 9.15787E-12 | up |
| MELO3C025095.2 | 256 | 0 | -10.617 | 9.33445E-19 | down |
| MELO3C025101.2 | 17135 | 1065 | -4.006 | 3.58346E-152 | down |
| MELO3C025102.2 | 3752 | 532 | -2.817 | 3.47126E-186 | down |
| MELO3C025110.2 | 87 | 11 | -3.067 | 1.70704E-10 | down |
| MELO3C025111.2 | 9799 | 1299 | -2.916 | 9.37407E-172 | down |
| MELO3C025138.2 | 292 | 75 | -1.96 | 2.32593E-19 | down |
| MELO3C025140.2 | 1911 | 4475 | 1.228 | 1.41601E-56 | up |
| MELO3C025142.2 | 244 | 96 | -1.349 | 5.66345E-12 | down |
| MELO3C025143.2 | 32 | 94 | 1.543 | 8.19481E-06 | up |
| MELO3C025149.2 | 20377 | 2552 | -2.997 | 3.63886E-106 | down |
| MELO3C025165.2 | 176 | 0 | -10.075 | 3.84974E-08 | down |
| MELO3C025166.2 | 154 | 10 | -3.94 | 0.000122019 | down |
| MELO3C025174.2 | 125 | 62 | -1.004 | 0.007422782 | down |
| MELO3C025175.2 | 347 | 717 | 1.046 | 1.39427E-18 | up |
| MELO3C025183.2 | 1217 | 194 | -2.642 | 1.76756E-30 | down |
| MELO3C025191.2 | 14 | 2 | -2.589 | 0.019364079 | down |
| MELO3C025192.2 | 169 | 453 | 1.424 | 1.2218E-18 | up |
| MELO3C025196.2 | 3346 | 1092 | -1.617 | 2.82558E-56 | down |
| MELO3C025199.2 | 86 | 18 | -2.266 | 9.56403E-08 | down |
| MELO3C025205.2 | 289 | 57 | -2.335 | 4.92116E-30 | down |
| MELO3C025206.2 | 198 | 418 | 1.081 | 2.93431E-05 | up |
| MELO3C025210.2 | 14 | 48 | 1.761 | 0.002083679 | up |
| MELO3C025219.2 | 3000 | 28289 | 3.237 | 5.68488E-155 | up |
| MELO3C025231.2 | 160 | 333 | 1.056 | 4.86016E-08 | up |
| MELO3C025251.2 | 24 | 7 | -1.725 | 0.028364442 | down |
| MELO3C025262.2 | 116 | 253 | 1.121 | 2.4095E-08 | up |
| MELO3C025264.2 | 76587 | 931 | -6.362 | 2.12188E-74 | down |
| MELO3C025268.2 | 392 | 3 | -7.21 | 2.93563E-44 | down |
| MELO3C025282.2 | 466 | 1589 | 1.77 | 1.45175E-28 | up |
| MELO3C025291.2 | 8 | 24 | 1.634 | 0.017254293 | up |
| MELO3C025295.2 | 356 | 62 | -2.53 | 4.10568E-34 | down |
| MELO3C025297.2 | 35 | 202 | 2.532 | 1.26034E-22 | up |
| MELO3C025301.2 | 4 | 53 | 3.566 | 4.22317E-09 | up |
| MELO3C025307.2 | 1156 | 474 | -1.287 | 6.44892E-30 | down |
| MELO3C025308.2 | 49 | 13 | -1.964 | 0.000509325 | down |
| MELO3C025316.2 | 2822 | 493 | -2.52 | 9.30384E-85 | down |
| MELO3C025327.2 | 4 | 0 | -4.542 | 0.029879344 | down |
| MELO3C025328.2 | 1967 | 5546 | 1.495 | 1.83008E-19 | up |
| MELO3C025332.2 | 23 | 106 | 2.208 | 1.51609E-05 | up |
| MELO3C025334.2 | 61 | 28 | -1.103 | 0.002742125 | down |
| MELO3C025338.2 | 144 | 681 | 2.24 | 2.92003E-35 | up |
| MELO3C025345.2 | 120 | 843 | 2.81 | 1.34603E-37 | up |
| MELO3C025346.2 | 6104 | 2152 | -1.505 | 3.97874E-54 | down |
| MELO3C025347.2 | 34020 | 13523 | -1.331 | 1.11952E-43 | down |
| MELO3C025352.2 | 5503 | 2022 | -1.445 | 4.26066E-36 | down |
| MELO3C025354.2 | 942 | 3169 | 1.749 | 2.92977E-61 | up |
| MELO3C025360.2 | 8 | 1 | -3.617 | 0.017860991 | down |
| MELO3C025365.2 | 137 | 1120 | 3.041 | 7.96378E-67 | up |
| MELO3C025369.2 | 21 | 0 | -7.048 | 2.5443E-06 | down |
| MELO3C025383.2 | 32 | 3 | -3.387 | 2.28563E-06 | down |
| MELO3C025392.2 | 195 | 80 | -1.278 | 1.89971E-09 | down |
| MELO3C025397.2 | 2542 | 812 | -1.647 | 8.26492E-40 | down |
| MELO3C025401.2 | 307 | 120 | -1.358 | 3.02094E-10 | down |
| MELO3C025405.2 | 95 | 28 | -1.783 | 5.10028E-06 | down |
| MELO3C025408.2 | 2473 | 7602 | 1.621 | 8.59403E-42 | up |
| MELO3C025424.2 | 304 | 108 | -1.485 | 1.16E-08 | down |
| MELO3C025433.2 | 81 | 38 | -1.121 | 0.000718845 | down |
| MELO3C025448.2 | 261 | 56 | -2.235 | 3.21997E-21 | down |
| MELO3C025456.2 | 318 | 84 | -1.927 | 2.63414E-17 | down |
| MELO3C025462.2 | 49 | 9 | -2.43 | 1.44013E-06 | down |
| MELO3C025463.2 | 134 | 66 | -1.019 | 0.002341157 | down |
| MELO3C025464.2 | 1346 | 196 | -2.775 | 3.29832E-78 | down |
| MELO3C025467.2 | 328 | 100 | -1.716 | 1.40851E-10 | down |
| MELO3C025470.2 | 235 | 554 | 1.235 | 4.39173E-15 | up |
| MELO3C025475.2 | 798 | 272 | -1.553 | 8.08223E-22 | down |
| MELO3C025477.2 | 384 | 15 | -4.754 | 7.86316E-66 | down |
| MELO3C025484.2 | 15 | 2 | -3.112 | 0.000918806 | down |
| MELO3C025485.2 | 5 | 0 | -4.921 | 0.011713661 | down |
| MELO3C025486.2 | 63 | 26 | -1.263 | 0.028393026 | down |
| MELO3C025493.2 | 899 | 411 | -1.129 | 6.52344E-20 | down |
| MELO3C025505.2 | 65 | 218 | 1.756 | 2.72308E-11 | up |
| MELO3C025508.2 | 21 | 66 | 1.659 | 0.000595248 | up |
| MELO3C025521.2 | 42 | 6 | -2.707 | 3.82794E-06 | down |
| MELO3C025523.2 | 77 | 1 | -6.016 | 1.52536E-12 | down |
| MELO3C025525.2 | 55 | 298 | 2.446 | 4.57683E-32 | up |
| MELO3C025532.2 | 7 | 43 | 2.673 | 4.83828E-06 | up |
| MELO3C025563.2 | 759 | 141 | -2.427 | 2.20596E-34 | down |
| MELO3C025579.2 | 35 | 1010 | 4.864 | 5.37812E-117 | up |
| MELO3C025583.2 | 3059 | 2 | -10.759 | 2.8072E-70 | down |
| MELO3C025587.2 | 31568 | 458 | -6.108 | 0 | down |
| MELO3C025597.2 | 8 | 31 | 1.983 | 0.002339048 | up |
| MELO3C025601.2 | 357 | 2973 | 3.058 | 1.35864E-179 | up |
| MELO3C025609.2 | 2871 | 414 | -2.796 | 6.35617E-211 | down |
| MELO3C025615.2 | 831 | 1679 | 1.015 | 2.87417E-20 | up |
| MELO3C025623.2 | 8 | 1 | -3.785 | 0.018499711 | down |
| MELO3C025626.2 | 19 | 47 | 1.285 | 0.006076677 | up |
| MELO3C025629.2 | 256 | 93 | -1.462 | 4.10101E-11 | down |
| MELO3C025641.2 | 298 | 629 | 1.081 | 3.44219E-18 | up |
| MELO3C025645.2 | 2625 | 1008 | -1.382 | 1.27018E-40 | down |
| MELO3C025651.2 | 418 | 1371 | 1.712 | 8.66826E-47 | up |
| MELO3C025662.2 | 1572 | 780 | -1.013 | 9.78028E-21 | down |
| MELO3C025663.2 | 443 | 4417 | 3.321 | 1.33995E-115 | up |
| MELO3C025667.2 | 43 | 331 | 2.939 | 8.56561E-37 | up |
| MELO3C025669.2 | 4953 | 811 | -2.611 | 7.06442E-199 | down |
| MELO3C025673.2 | 1266 | 440 | -1.524 | 2.142E-27 | down |
| MELO3C025676.2 | 5 | 73 | 4.006 | 1.24866E-11 | up |
| MELO3C025677.2 | 271 | 635 | 1.228 | 2.74152E-14 | up |
| MELO3C025678.2 | 0 | 26 | 6.964 | 2.2145E-07 | up |
| MELO3C025688.2 | 0 | 25 | 6.921 | 4.2223E-07 | up |
| MELO3C025689.2 | 287 | 107 | -1.425 | 7.47702E-06 | down |
| MELO3C025691.2 | 1401 | 682 | -1.04 | 2.48138E-22 | down |
| MELO3C025702.2 | 1462 | 598 | -1.289 | 5.32256E-14 | down |
| MELO3C025706.2 | 9 | 0 | -5.821 | 0.000482523 | down |
| MELO3C025708.2 | 241 | 519 | 1.105 | 7.46673E-13 | up |
| MELO3C025712.2 | 337 | 42 | -2.98 | 5.01932E-22 | down |
| MELO3C025717.2 | 21 | 2 | -3.323 | 0.00025857 | down |
| MELO3C025720.2 | 5698 | 7 | -9.72 | 3.24502E-196 | down |
| MELO3C025735.2 | 41 | 126 | 1.615 | 1.11367E-08 | up |
| MELO3C025737.2 | 24 | 58 | 1.261 | 0.00790502 | up |
| MELO3C025741.2 | 1234 | 2871 | 1.218 | 2.25627E-23 | up |
| MELO3C025744.2 | 283 | 84 | -1.758 | 4.60661E-16 | down |
| MELO3C025746.2 | 42 | 89 | 1.08 | 0.002092526 | up |
| MELO3C025753.2 | 92 | 44 | -1.071 | 0.001721644 | down |
| MELO3C025754.2 | 38 | 18 | -1.07 | 0.023876704 | down |
| MELO3C025758.2 | 861 | 1909 | 1.149 | 2.9925E-31 | up |
| MELO3C025761.2 | 199 | 2656 | 3.74 | 8.73826E-80 | up |
| MELO3C025764.2 | 8 | 487 | 5.96 | 1.01188E-09 | up |
| MELO3C025770.2 | 67 | 28 | -1.253 | 0.000631639 | down |
| MELO3C025771.2 | 112 | 297 | 1.411 | 7.20575E-12 | up |
| MELO3C025772.2 | 14756 | 4220 | -1.806 | 4.17787E-47 | down |
| MELO3C025779.2 | 106 | 323 | 1.612 | 3.81E-18 | up |
| MELO3C025783.2 | 283 | 2575 | 3.186 | 2.84293E-130 | up |
| MELO3C025784.2 | 155 | 635 | 2.03 | 1.06022E-31 | up |
| MELO3C025791.2 | 676 | 297 | -1.187 | 1.03762E-13 | down |
| MELO3C025794.2 | 204 | 63 | -1.69 | 5.21431E-09 | down |
| MELO3C025795.2 | 749 | 341 | -1.135 | 1.03156E-20 | down |
| MELO3C025797.2 | 4 | 32 | 3.143 | 3.13756E-05 | up |
| MELO3C025798.2 | 15134 | 177 | -6.416 | 2.12251E-48 | down |
| MELO3C025807.2 | 6 | 489 | 6.359 | 8.41556E-55 | up |
| MELO3C025837.2 | 23 | 3 | -3.038 | 0.000217542 | down |
| MELO3C025853.2 | 1266 | 443 | -1.519 | 8.07491E-23 | down |
| MELO3C025855.2 | 4225 | 454 | -3.218 | 3.50832E-159 | down |
| MELO3C025857.2 | 382 | 1140 | 1.58 | 3.04351E-28 | up |
| MELO3C025861.2 | 594 | 240 | -1.312 | 4.6205E-16 | down |
| MELO3C025862.2 | 59 | 123 | 1.081 | 0.00050899 | up |
| MELO3C025863.2 | 662 | 146 | -2.18 | 4.37839E-50 | down |
| MELO3C025864.2 | 88 | 404 | 2.199 | 1.0566E-28 | up |
| MELO3C025865.2 | 994 | 202 | -2.3 | 2.37831E-78 | down |
| MELO3C025866.2 | 203 | 685 | 1.756 | 2.62691E-21 | up |
| MELO3C025867.2 | 162 | 343 | 1.08 | 2.40217E-10 | up |
| MELO3C025869.2 | 4960 | 1213 | -2.031 | 1.99141E-85 | down |
| MELO3C025872.2 | 574 | 248 | -1.214 | 2.64746E-19 | down |
| MELO3C025876.2 | 9004 | 3767 | -1.257 | 5.64754E-56 | down |
| MELO3C025879.2 | 256 | 606 | 1.243 | 9.11723E-12 | up |
| MELO3C025882.2 | 39 | 346 | 3.147 | 1.52039E-29 | up |
| MELO3C025883.2 | 74 | 391 | 2.41 | 1.57221E-24 | up |
| MELO3C025884.2 | 14 | 68 | 2.296 | 1.35321E-07 | up |
| MELO3C025885.2 | 89 | 222 | 1.317 | 7.04546E-09 | up |
| MELO3C025886.2 | 916 | 2010 | 1.134 | 1.31278E-26 | up |
| MELO3C025888.2 | 18448 | 839 | -4.457 | 0 | down |
| MELO3C025892.2 | 11199 | 3893 | -1.525 | 1.18136E-44 | down |
| MELO3C025896.2 | 263 | 123 | -1.104 | 2.61464E-09 | down |
| MELO3C025900.2 | 0 | 19 | 6.5 | 3.62524E-06 | up |
| MELO3C025902.2 | 79 | 22 | -1.848 | 7.8286E-08 | down |
| MELO3C025912.2 | 2797 | 106 | -4.728 | 1.06964E-206 | down |
| MELO3C025914.2 | 1451 | 468 | -1.633 | 2.42856E-56 | down |
| MELO3C025918.2 | 754 | 247 | -1.614 | 2.68439E-33 | down |
| MELO3C025924.2 | 3 | 28 | 3.239 | 4.35812E-05 | up |
| MELO3C025940.2 | 154 | 661 | 2.097 | 2.20288E-40 | up |
| MELO3C025950.2 | 3243 | 1470 | -1.141 | 2.1548E-12 | down |
| MELO3C025951.2 | 1109 | 486 | -1.191 | 3.39675E-25 | down |
| MELO3C025953.2 | 1826 | 709 | -1.364 | 1.34158E-47 | down |
| MELO3C025955.2 | 37 | 4 | -3.077 | 1.80349E-06 | down |
| MELO3C025974.2 | 44 | 0 | -8.083 | 3.24424E-10 | down |
| MELO3C026001.2 | 54 | 12 | -2.202 | 1.9848E-06 | down |
| MELO3C026013.2 | 25 | 91 | 1.853 | 2.13695E-07 | up |
| MELO3C026014.2 | 3 | 25 | 3.235 | 0.000109819 | up |
| MELO3C026018.2 | 652 | 3357 | 2.364 | 5.42105E-63 | up |
| MELO3C026019.2 | 246 | 1053 | 2.097 | 1.05246E-59 | up |
| MELO3C026021.2 | 638 | 1294 | 1.019 | 1.85576E-25 | up |
| MELO3C026029.2 | 115 | 927 | 3.01 | 3.68129E-41 | up |
| MELO3C026034.2 | 8 | 139 | 4.229 | 1.63732E-16 | up |
| MELO3C026043.2 | 654 | 268 | -1.285 | 1.87754E-11 | down |
| MELO3C026045.2 | 4264 | 904 | -2.238 | 7.28007E-14 | down |
| MELO3C026046.2 | 3364 | 703 | -2.259 | 5.78433E-105 | down |
| MELO3C026050.2 | 1655 | 593 | -1.481 | 1.35526E-35 | down |
| MELO3C026051.2 | 505 | 1629 | 1.688 | 2.03891E-52 | up |
| MELO3C026054.2 | 11009 | 5457 | -1.013 | 9.19297E-43 | down |
| MELO3C026058.2 | 1292 | 131 | -3.298 | 9.81974E-74 | down |
| MELO3C026066.2 | 643 | 1 | -10.092 | 1.73076E-22 | down |
| MELO3C026099.2 | 23059 | 3097 | -2.896 | 5.38832E-175 | down |
| MELO3C026109.2 | 1355 | 257 | -2.399 | 1.29896E-93 | down |
| MELO3C026115.2 | 29 | 0 | -7.49 | 1.83594E-08 | down |
| MELO3C026134.2 | 225 | 0 | -10.427 | 6.80507E-18 | down |
| MELO3C026137.2 | 382 | 67 | -2.498 | 6.69819E-27 | down |
| MELO3C026142.2 | 36 | 2 | -3.94 | 3.67745E-08 | down |
| MELO3C026143.2 | 3 | 102 | 4.919 | 9.24719E-18 | up |
| MELO3C026158.2 | 7 | 1 | -3.7 | 0.020213978 | down |
| MELO3C026160.2 | 9007 | 1731 | -2.38 | 9.85675E-172 | down |
| MELO3C026170.2 | 2249 | 40 | -5.813 | 1.07502E-154 | down |
| MELO3C026179.2 | 26 | 3 | -3.136 | 2.60951E-05 | down |
| MELO3C026183.2 | 29 | 0 | -7.471 | 1.24241E-07 | down |
| MELO3C026184.2 | 1358 | 159 | -3.092 | 6.80973E-05 | down |
| MELO3C026188.2 | 189 | 16 | -3.606 | 0.000533709 | down |
| MELO3C026194.2 | 24 | 159 | 2.775 | 2.42857E-13 | up |
| MELO3C026199.2 | 128 | 375 | 1.557 | 9.50564E-21 | up |
| MELO3C026210.2 | 8442 | 4154 | -1.023 | 6.41612E-33 | down |
| MELO3C026220.2 | 25 | 90 | 1.815 | 1.34012E-05 | up |
| MELO3C026221.2 | 3433 | 1450 | -1.244 | 4.14476E-30 | down |
| MELO3C026226.2 | 3 | 30 | 3.355 | 2.64659E-05 | up |
| MELO3C026227.2 | 75 | 330 | 2.137 | 9.81178E-17 | up |
| MELO3C026229.2 | 13940 | 4983 | -1.484 | 8.55819E-56 | down |
| MELO3C026230.2 | 3719 | 1809 | -1.04 | 7.28238E-32 | down |
| MELO3C026235.2 | 4086 | 228 | -4.166 | 1.73788E-216 | down |
| MELO3C026236.2 | 35 | 81 | 1.189 | 0.000739061 | up |
| MELO3C026238.2 | 16 | 106 | 2.728 | 3.12344E-07 | up |
| MELO3C026242.2 | 10 | 32 | 1.669 | 0.005502396 | up |
| MELO3C026246.2 | 10095 | 1881 | -2.424 | 6.21438E-145 | down |
| MELO3C026247.2 | 2121 | 656 | -1.692 | 1.23629E-60 | down |
| MELO3C026250.2 | 2593 | 474 | -2.454 | 2.88671E-93 | down |
| MELO3C026252.2 | 664 | 305 | -1.118 | 3.51266E-13 | down |
| MELO3C026259.2 | 22757 | 4443 | -2.357 | 6.63045E-96 | down |
| MELO3C026260.2 | 55 | 1 | -5.57 | 5.89404E-10 | down |
| MELO3C026261.2 | 367 | 99 | -1.884 | 4.09674E-28 | down |
| MELO3C026262.2 | 125 | 29 | -2.105 | 2.1222E-11 | down |
| MELO3C026265.2 | 1 | 55 | 6.195 | 1.31281E-07 | up |
| MELO3C026270.2 | 19 | 168 | 3.114 | 4.54974E-14 | up |
| MELO3C026272.2 | 99 | 421 | 2.09 | 5.79012E-27 | up |
| MELO3C026278.2 | 18 | 384 | 4.402 | 1.71226E-31 | up |
| MELO3C026288.2 | 3974 | 1423 | -1.483 | 8.08319E-40 | down |
| MELO3C026289.2 | 284 | 9 | -4.92 | 9.57202E-45 | down |
| MELO3C026292.2 | 11190 | 1673 | -2.742 | 4.45354E-222 | down |
| MELO3C026296.2 | 721 | 1739 | 1.271 | 9.31244E-26 | up |
| MELO3C026300.2 | 19614 | 9595 | -1.032 | 1.48782E-22 | down |
| MELO3C026307.2 | 70 | 33 | -1.068 | 0.001691502 | down |
| MELO3C026308.2 | 52 | 142 | 1.448 | 3.0725E-08 | up |
| MELO3C026310.2 | 352 | 1066 | 1.597 | 1.24308E-17 | up |
| MELO3C026323.2 | 4 | 0 | -4.752 | 0.02050639 | down |
| MELO3C026331.2 | 6322 | 2929 | -1.11 | 3.01454E-53 | down |
| MELO3C026338.2 | 519 | 1235 | 1.253 | 1.20658E-29 | up |
| MELO3C026340.2 | 5 | 177 | 5.2 | 4.44436E-26 | up |
| MELO3C026342.2 | 426 | 932 | 1.129 | 8.65358E-18 | up |
| MELO3C026366.2 | 163 | 44 | -1.884 | 6.74932E-14 | down |
| MELO3C026367.2 | 44 | 16 | -1.472 | 0.004081832 | down |
| MELO3C026368.2 | 2278 | 710 | -1.681 | 1.27821E-51 | down |
| MELO3C026372.2 | 2543 | 618 | -2.042 | 7.70363E-36 | down |
| MELO3C026374.2 | 938 | 395 | -1.251 | 1.50946E-22 | down |
| MELO3C026375.2 | 467 | 165 | -1.504 | 1.82162E-11 | down |
| MELO3C026378.2 | 2485 | 438 | -2.504 | 1.07888E-40 | down |
| MELO3C026380.2 | 480 | 188 | -1.351 | 9.54419E-18 | down |
| MELO3C026387.2 | 1568 | 390 | -2.008 | 6.70031E-60 | down |
| MELO3C026388.2 | 113 | 260 | 1.202 | 6.68359E-06 | up |
| MELO3C026395.2 | 4 | 66 | 3.904 | 1.5316E-10 | up |
| MELO3C026398.2 | 1308 | 587 | -1.156 | 1.69991E-33 | down |
| MELO3C026401.2 | 12 | 0 | -6.128 | 0.000115159 | down |
| MELO3C026402.2 | 2817 | 134 | -4.394 | 9.53842E-205 | down |
| MELO3C026403.2 | 142 | 300 | 1.074 | 7.2914E-11 | up |
| MELO3C026419.2 | 263 | 863 | 1.715 | 2.77491E-32 | up |
| MELO3C026424.2 | 1637 | 572 | -1.518 | 6.85308E-41 | down |
| MELO3C026431.2 | 158 | 44 | -1.826 | 9.79056E-07 | down |
| MELO3C026432.2 | 97 | 7 | -3.78 | 1.78477E-16 | down |
| MELO3C026436.2 | 24213 | 7260 | -1.738 | 7.19654E-96 | down |
| MELO3C026468.2 | 947 | 136 | -2.807 | 2.58164E-32 | down |
| MELO3C026484.2 | 19 | 95 | 2.293 | 2.7112E-08 | up |
| MELO3C026485.2 | 27 | 140 | 2.404 | 5.62736E-12 | up |
| MELO3C026486.2 | 1955 | 0 | -13.55 | 5.62462E-30 | down |
| MELO3C026489.2 | 360 | 3812 | 3.403 | 2.60992E-225 | up |
| MELO3C026493.2 | 99 | 984 | 3.322 | 3.5479E-43 | up |
| MELO3C026498.2 | 2510 | 99 | -4.657 | 7.79177E-276 | down |
| MELO3C026500.2 | 3706 | 122 | -4.92 | 6.80796E-286 | down |
| MELO3C026507.2 | 40 | 7 | -2.526 | 6.39694E-05 | down |
| MELO3C026509.2 | 12 | 111 | 3.298 | 5.65066E-11 | up |
| MELO3C026512.2 | 27746 | 6818 | -2.025 | 3.4157E-134 | down |
| MELO3C026517.2 | 31 | 88 | 1.53 | 1.19551E-05 | up |
| MELO3C026518.2 | 408 | 126 | -1.693 | 2.37864E-18 | down |
| MELO3C026519.2 | 321 | 916 | 1.512 | 5.57114E-22 | up |
| MELO3C026529.2 | 47 | 6 | -3.125 | 1.13493E-07 | down |
| MELO3C026532.2 | 177520 | 79745 | -1.154 | 1.3314E-48 | down |
| MELO3C026536.2 | 5075 | 11244 | 1.148 | 5.29048E-56 | up |
| MELO3C026537.2 | 179 | 431 | 1.271 | 2.7087E-17 | up |
| MELO3C026550.2 | 7607 | 29265 | 1.944 | 1.94966E-35 | up |
| MELO3C026552.2 | 26 | 68 | 1.393 | 0.000611011 | up |
| MELO3C026562.2 | 17 | 5 | -1.743 | 0.022133003 | down |
| MELO3C026563.2 | 55 | 118 | 1.089 | 3.33369E-05 | up |
| MELO3C026564.2 | 390 | 44 | -3.172 | 7.4192E-27 | down |
| MELO3C026565.2 | 3 | 236 | 6.141 | 5.16894E-27 | up |
| MELO3C026569.2 | 144 | 2 | -5.771 | 6.64995E-24 | down |
| MELO3C026570.2 | 3 | 30 | 3.366 | 0.000783643 | up |
| MELO3C026572.2 | 512 | 1145 | 1.161 | 4.91558E-14 | up |
| MELO3C026575.2 | 282 | 56 | -2.313 | 3.61974E-13 | down |
| MELO3C026578.2 | 3 | 60 | 4.318 | 1.69361E-10 | up |
| MELO3C026581.2 | 1487 | 574 | -1.374 | 9.01538E-36 | down |
| MELO3C026585.2 | 1823 | 4623 | 1.343 | 3.38468E-46 | up |
| MELO3C026587.2 | 302 | 692 | 1.197 | 1.07929E-17 | up |
| MELO3C026588.2 | 0 | 8 | 4.351 | 0.007189061 | up |
| MELO3C026590.2 | 467 | 3303 | 2.824 | 7.19472E-59 | up |
| MELO3C026594.2 | 2642 | 166 | -3.995 | 0 | down |
| MELO3C026599.2 | 15 | 0 | -6.549 | 1.0551E-05 | down |
| MELO3C026603.2 | 68 | 913 | 3.75 | 1.21562E-88 | up |
| MELO3C026609.2 | 17 | 81 | 2.25 | 6.41183E-08 | up |
| MELO3C026611.2 | 1674 | 368 | -2.186 | 8.63777E-84 | down |
| MELO3C026618.2 | 47 | 161 | 1.787 | 1.91248E-11 | up |
| MELO3C026629.2 | 1288 | 4496 | 1.804 | 1.92126E-94 | up |
| MELO3C026630.2 | 573 | 109 | -2.4 | 1.04854E-29 | down |
| MELO3C026634.2 | 10 | 0 | -5.935 | 0.000361184 | down |
| MELO3C026652.2 | 13 | 0 | -6.357 | 1.8841E-05 | down |
| MELO3C026654.2 | 140 | 0 | -9.742 | 1.09164E-15 | down |
| MELO3C026658.2 | 487 | 1509 | 1.632 | 2.36807E-44 | up |
| MELO3C026665.2 | 276 | 819 | 1.572 | 3.35867E-25 | up |
| MELO3C026677.2 | 3 | 449 | 7.24 | 1.24664E-36 | up |
| MELO3C026685.2 | 69 | 358 | 2.366 | 3.46821E-25 | up |
| MELO3C026686.2 | 408 | 1 | -8.846 | 3.20071E-25 | down |
| MELO3C026687.2 | 42 | 0 | -7.068 | 3.68357E-08 | down |
| MELO3C026689.2 | 3025 | 182 | -4.054 | 1.37691E-281 | down |
| MELO3C026715.2 | 1538 | 554 | -1.474 | 1.38502E-38 | down |
| MELO3C026718.2 | 0 | 3339 | 13.983 | 3.30706E-22 | up |
| MELO3C026722.2 | 14769 | 1247 | -3.567 | 2.33969E-153 | down |
| MELO3C026723.2 | 2230 | 720 | -1.631 | 2.87016E-20 | down |
| MELO3C026731.2 | 0 | 470 | 10.192 | 1.07621E-17 | up |
| MELO3C026732.2 | 9862 | 4125 | -1.257 | 4.19344E-45 | down |
| MELO3C026734.2 | 66 | 553 | 3.065 | 1.06009E-58 | up |
| MELO3C026737.2 | 494 | 1471 | 1.576 | 1.06376E-32 | up |
| MELO3C026738.2 | 952 | 36 | -4.702 | 9.3575E-114 | down |
| MELO3C026748.2 | 5 | 180 | 5.204 | 2.42714E-28 | up |
| MELO3C026749.2 | 145 | 994 | 2.778 | 1.05509E-05 | up |
| MELO3C026752.2 | 91 | 32 | -1.519 | 1.81754E-05 | down |
| MELO3C026754.2 | 1157 | 182 | -2.667 | 3.81506E-08 | down |
| MELO3C026755.2 | 96 | 696 | 2.857 | 0.007080695 | up |
| MELO3C026765.2 | 12 | 0 | -6.191 | 5.84593E-05 | down |
| MELO3C026769.2 | 10 | 0 | -5.901 | 0.000215587 | down |
| MELO3C026782.2 | 15 | 1037 | 6.112 | 1.90636E-90 | up |
| MELO3C026792.2 | 121 | 37 | -1.699 | 0.000727733 | down |
| MELO3C026796.2 | 114 | 38 | -1.578 | 1.47042E-07 | down |
| MELO3C026802.2 | 33872 | 1010 | -5.068 | 0 | down |
| MELO3C026803.2 | 487 | 1360 | 1.48 | 7.43022E-34 | up |
| MELO3C026805.2 | 13 | 508 | 5.324 | 3.26135E-69 | up |
| MELO3C026807.2 | 19 | 4 | -2.39 | 0.002440766 | down |
| MELO3C026818.2 | 0 | 14 | 6.098 | 4.28629E-05 | up |
| MELO3C026824.2 | 533 | 69 | -2.956 | 2.49859E-33 | down |
| MELO3C026828.2 | 207 | 22 | -3.211 | 9.77051E-16 | down |
| MELO3C026842.2 | 70 | 0 | -8.745 | 3.61339E-12 | down |
| MELO3C026843.2 | 190 | 13 | -3.888 | 2.22547E-18 | down |
| MELO3C026847.2 | 387 | 140 | -1.463 | 2.16245E-19 | down |
| MELO3C026848.2 | 92 | 26 | -1.812 | 5.81494E-08 | down |
| MELO3C026849.2 | 93 | 190 | 1.027 | 8.68057E-06 | up |
| MELO3C026868.2 | 41 | 8 | -2.384 | 1.09533E-05 | down |
| MELO3C026870.2 | 2128 | 12384 | 2.541 | 2.28437E-89 | up |
| MELO3C026873.2 | 1612 | 784 | -1.041 | 7.70031E-30 | down |
| MELO3C026875.2 | 10284 | 3559 | -1.531 | 4.63622E-94 | down |
| MELO3C026887.2 | 106 | 31 | -1.759 | 2.68587E-08 | down |
| MELO3C026889.2 | 1058 | 32 | -5.054 | 2.63217E-143 | down |
| MELO3C026892.2 | 11 | 60 | 2.403 | 8.75628E-08 | up |
| MELO3C026896.2 | 818 | 202 | -2.014 | 4.14595E-16 | down |
| MELO3C026897.2 | 5826 | 2119 | -1.459 | 9.81481E-62 | down |
| MELO3C026898.2 | 53016 | 24766 | -1.098 | 2.7231E-40 | down |
| MELO3C026900.2 | 20 | 5 | -2.001 | 0.006446771 | down |
| MELO3C026906.2 | 5 | 25 | 2.245 | 0.001710797 | up |
| MELO3C026907.2 | 577 | 162 | -1.84 | 4.98242E-23 | down |
| MELO3C026918.2 | 403 | 190 | -1.085 | 2.12673E-11 | down |
| MELO3C026919.2 | 36009 | 11328 | -1.668 | 5.32166E-104 | down |
| MELO3C026921.2 | 43 | 151 | 1.807 | 7.55773E-11 | up |
| MELO3C026941.2 | 2 | 31 | 3.553 | 2.23216E-06 | up |
| MELO3C026945.2 | 405 | 157 | -1.359 | 6.23798E-12 | down |
| MELO3C026947.2 | 57 | 1 | -6.574 | 3.03615E-09 | down |
| MELO3C026950.2 | 751 | 1686 | 1.166 | 1.67525E-29 | up |
| MELO3C026951.2 | 404 | 25 | -3.999 | 1.48239E-62 | down |
| MELO3C026961.2 | 60 | 5 | -3.661 | 1.4638E-11 | down |
| MELO3C026967.2 | 159 | 35 | -2.16 | 1.95869E-13 | down |
| MELO3C026968.2 | 1004 | 232 | -2.112 | 1.0117E-53 | down |
| MELO3C026973.2 | 750 | 1538 | 1.034 | 1.05763E-24 | up |
| MELO3C026974.2 | 66 | 18 | -1.906 | 4.89445E-06 | down |
| MELO3C026992.2 | 501 | 199 | -1.327 | 5.51908E-11 | down |
| MELO3C026998.2 | 86 | 231 | 1.421 | 8.09879E-10 | up |
| MELO3C027001.2 | 43 | 615 | 3.842 | 3.84946E-39 | up |
| MELO3C027004.2 | 179 | 56 | -1.668 | 2.25852E-09 | down |
| MELO3C027005.2 | 64 | 14 | -2.204 | 6.56417E-07 | down |
| MELO3C027009.2 | 250 | 17 | -3.834 | 7.14643E-39 | down |
| MELO3C027020.2 | 853 | 1818 | 1.091 | 1.19009E-21 | up |
| MELO3C027040.2 | 4 | 133 | 5.139 | 1.29508E-17 | up |
| MELO3C027042.2 | 28 | 1 | -5.56 | 3.91794E-06 | down |
| MELO3C027052.2 | 26 | 1 | -5.433 | 2.43763E-05 | down |
| MELO3C027054.2 | 95 | 47 | -1.005 | 0.000918821 | down |
| MELO3C027057.2 | 1037 | 6 | -7.327 | 8.61756E-95 | down |
| MELO3C027061.2 | 12820 | 4691 | -1.451 | 1.19932E-46 | down |
| MELO3C027064.2 | 1581 | 353 | -2.161 | 3.75085E-76 | down |
| MELO3C027083.2 | 476 | 15 | -4.961 | 4.74412E-75 | down |
| MELO3C027089.2 | 129 | 486 | 1.91 | 3.76681E-27 | up |
| MELO3C027100.2 | 7 | 0 | -5.517 | 0.001256121 | down |
| MELO3C027107.2 | 145 | 46 | -1.664 | 5.43881E-11 | down |
| MELO3C027112.2 | 2673 | 310 | -3.107 | 5.91885E-20 | down |
| MELO3C027119.2 | 5295 | 1086 | -2.285 | 2.66454E-196 | down |
| MELO3C027120.2 | 535 | 1596 | 1.576 | 4.82356E-24 | up |
| MELO3C027124.2 | 254 | 33 | -2.959 | 2.68885E-30 | down |
| MELO3C027138.2 | 750 | 353 | -1.084 | 1.79299E-13 | down |
| MELO3C027140.2 | 12 | 2 | -2.998 | 0.006101131 | down |
| MELO3C027143.2 | 1153 | 523 | -1.142 | 1.57698E-27 | down |
| MELO3C027158.2 | 1872 | 4726 | 1.336 | 6.05797E-51 | up |
| MELO3C027159.2 | 92 | 11 | -3.117 | 1.26985E-14 | down |
| MELO3C027169.2 | 67 | 0 | -8.673 | 5.31307E-12 | down |
| MELO3C027172.2 | 711 | 2389 | 1.748 | 3.06783E-23 | up |
| MELO3C027179.2 | 96 | 421 | 2.136 | 5.30275E-22 | up |
| MELO3C027184.2 | 32 | 108 | 1.738 | 1.40957E-08 | up |
| MELO3C027191.2 | 14 | 0 | -6.45 | 1.09067E-05 | down |
| MELO3C027203.2 | 173 | 375 | 1.114 | 6.09372E-09 | up |
| MELO3C027208.2 | 2 | 347 | 7.842 | 6.33161E-15 | up |
| MELO3C027216.2 | 65 | 11 | -2.609 | 4.34092E-07 | down |
| MELO3C027220.2 | 6 | 0 | -5.104 | 0.007271658 | down |
| MELO3C027227.2 | 16 | 3 | -2.553 | 0.003191018 | down |
| MELO3C027239.2 | 88 | 197 | 1.169 | 9.8115E-06 | up |
| MELO3C027244.2 | 613 | 170 | -1.843 | 7.0148E-11 | down |
| MELO3C027250.2 | 42 | 12 | -1.797 | 0.000448408 | down |
| MELO3C027254.2 | 25 | 81 | 1.675 | 8.6909E-06 | up |
| MELO3C027259.2 | 67 | 0 | -8.686 | 6.50725E-12 | down |
| MELO3C027276.2 | 0 | 7 | 5 | 0.005219053 | up |
| MELO3C027277.2 | 486 | 2 | -8.099 | 4.21582E-38 | down |
| MELO3C027278.2 | 7 | 38 | 2.5 | 1.70786E-05 | up |
| MELO3C027288.2 | 3388 | 448 | -2.92 | 0.000184163 | down |
| MELO3C027297.2 | 1541 | 252 | -2.618 | 4.91344E-54 | down |
| MELO3C027325.2 | 106 | 0 | -9.344 | 4.43069E-14 | down |
| MELO3C027338.2 | 9 | 0 | -5.789 | 0.001333861 | down |
| MELO3C027345.2 | 2954 | 1106 | -1.416 | 9.77292E-56 | down |
| MELO3C027346.2 | 32 | 271 | 3.081 | 3.54312E-14 | up |
| MELO3C027362.2 | 1875 | 674 | -1.475 | 1.41971E-53 | down |
| MELO3C027375.2 | 145 | 429 | 1.573 | 3.34774E-10 | up |
| MELO3C027376.2 | 69 | 0 | -8.734 | 2.8595E-12 | down |
| MELO3C027378.2 | 185 | 25 | -2.877 | 1.07024E-20 | down |
| MELO3C027379.2 | 783 | 2136 | 1.448 | 7.52166E-49 | up |
| MELO3C027402.2 | 116 | 48 | -1.27 | 0.000161698 | down |
| MELO3C027407.2 | 1577 | 659 | -1.258 | 4.91238E-42 | down |
| MELO3C027408.2 | 6 | 28 | 2.191 | 0.005793468 | up |
| MELO3C027413.2 | 53 | 12 | -2.132 | 9.04417E-07 | down |
| MELO3C027418.2 | 77 | 31 | -1.279 | 0.01344959 | down |
| MELO3C027420.2 | 236 | 1431 | 2.602 | 3.04992E-66 | up |
| MELO3C027425.2 | 735 | 239 | -1.618 | 4.77271E-23 | down |
| MELO3C027428.2 | 187 | 10 | -4.315 | 2.57799E-28 | down |
| MELO3C027441.2 | 145 | 707 | 2.281 | 3.94014E-51 | up |
| MELO3C027448.2 | 1567 | 107 | -3.873 | 1.87348E-202 | down |
| MELO3C027461.2 | 6669 | 3162 | -1.076 | 1.11066E-11 | down |
| MELO3C027462.2 | 79 | 33 | -1.264 | 0.000204395 | down |
| MELO3C027466.2 | 0 | 14 | 6.101 | 4.99412E-05 | up |
| MELO3C027479.2 | 340 | 0 | -11.027 | 2.58952E-20 | down |
| MELO3C027509.2 | 0 | 6 | 4.793 | 0.008898715 | up |
| MELO3C027538.2 | 83 | 0 | -8.984 | 3.7425E-13 | down |
| MELO3C027553.2 | 3 | 24 | 3.001 | 0.000422756 | up |
| MELO3C027595.2 | 87 | 32 | -1.429 | 2.13744E-05 | down |
| MELO3C027607.2 | 77 | 0 | -8.888 | 6.15373E-13 | down |
| MELO3C027615.2 | 105 | 602 | 2.523 | 3.60236E-41 | up |
| MELO3C027618.2 | 385 | 0 | -10.244 | 8.55358E-18 | down |
| MELO3C027632.2 | 0 | 8 | 5.236 | 0.00180668 | up |
| MELO3C027633.2 | 17 | 0 | -6.69 | 2.61163E-06 | down |
| MELO3C027646.2 | 1 | 700 | 9.289 | 5.79658E-28 | up |
| MELO3C027654.2 | 4 | 41 | 3.316 | 4.70867E-07 | up |
| MELO3C027671.2 | 27 | 0 | -7.357 | 5.09579E-08 | down |
| MELO3C027697.2 | 11 | 0 | -6.105 | 7.85953E-05 | down |
| MELO3C027699.2 | 0 | 11 | 5.753 | 0.000200692 | up |
| MELO3C027701.2 | 105 | 0 | -9.329 | 3.19198E-14 | down |
| MELO3C027702.2 | 0 | 7 | 5.09 | 0.005477162 | up |
| MELO3C027740.2 | 69 | 13 | -2.354 | 2.55729E-07 | down |
| MELO3C027746.2 | 117 | 5 | -4.583 | 1.05149E-16 | down |
| MELO3C027753.2 | 19 | 5 | -1.974 | 0.005406405 | down |
| MELO3C027756.2 | 0 | 62 | 8.229 | 5.08969E-11 | up |
| MELO3C027769.2 | 557 | 0 | -11.738 | 5.17667E-23 | down |
| MELO3C027773.2 | 100 | 388 | 1.958 | 2.3684E-21 | up |
| MELO3C027785.2 | 0 | 16 | 6.291 | 1.21185E-05 | up |
| MELO3C027796.2 | 16 | 73 | 2.188 | 0.005714046 | up |
| MELO3C027813.2 | 28 | 3 | -3.091 | 3.31505E-05 | down |
| MELO3C027823.2 | 182 | 50 | -1.858 | 2.77694E-15 | down |
| MELO3C027831.2 | 2 | 20 | 3.454 | 0.00072595 | up |
| MELO3C027836.2 | 51 | 10 | -2.389 | 5.82842E-06 | down |
| MELO3C027843.2 | 33 | 0 | -7.671 | 4.29158E-09 | down |
| MELO3C027847.2 | 0 | 19 | 5.59 | 6.27817E-05 | up |
| MELO3C027855.2 | 2004 | 441 | -2.183 | 1.25506E-53 | down |
| MELO3C027872.2 | 1214 | 0 | -12.861 | 2.13724E-27 | down |
| MELO3C027877.2 | 0 | 7 | 5.152 | 0.002213197 | up |
| MELO3C027885.2 | 12 | 2 | -2.771 | 0.008802076 | down |
| MELO3C027887.2 | 18 | 1 | -3.66 | 0.000368221 | down |
| MELO3C027893.2 | 2 | 28 | 3.608 | 1.39366E-05 | up |
| MELO3C027905.2 | 0 | 5 | 3.797 | 0.027041954 | up |
| MELO3C027907.2 | 127 | 262 | 1.049 | 0.000159424 | up |
| MELO3C027912.2 | 1 | 29 | 5.274 | 1.54962E-05 | up |
| MELO3C027913.2 | 58 | 8 | -2.791 | 1.10824E-06 | down |
| MELO3C027922.2 | 0 | 15 | 6.182 | 1.9818E-05 | up |
| MELO3C027930.2 | 2096 | 283 | -2.895 | 1.06841E-103 | down |
| MELO3C027934.2 | 0 | 49 | 7.901 | 4.35959E-10 | up |
| MELO3C027940.2 | 8 | 0 | -4.557 | 0.005173307 | down |
| MELO3C027946.2 | 4 | 97 | 4.711 | 1.33648E-15 | up |
| MELO3C027949.2 | 0 | 12 | 5.87 | 9.70626E-05 | up |
| MELO3C027962.2 | 124 | 628 | 2.348 | 7.94917E-43 | up |
| MELO3C027971.2 | 58 | 285 | 2.292 | 5.5633E-21 | up |
| MELO3C027975.2 | 740 | 0 | -12.147 | 1.31127E-24 | down |
| MELO3C027992.2 | 7 | 0 | -5.375 | 0.001971368 | down |
| MELO3C027995.2 | 43 | 14 | -1.569 | 0.002444627 | down |
| MELO3C027998.2 | 11 | 0 | -6.11 | 7.18507E-05 | down |
| MELO3C028009.2 | 7 | 22 | 1.715 | 0.013359088 | up |
| MELO3C028020.2 | 15 | 2 | -2.891 | 0.003940988 | down |
| MELO3C028032.2 | 0 | 13 | 5.931 | 8.46307E-05 | up |
| MELO3C028033.2 | 24 | 0 | -6.221 | 4.62641E-06 | down |
| MELO3C028034.2 | 0 | 4 | 4.399 | 0.026597153 | up |
| MELO3C028036.2 | 0 | 66 | 8.321 | 3.04355E-11 | up |
| MELO3C028037.2 | 40 | 13 | -1.645 | 0.00082506 | down |
| MELO3C028069.2 | 17 | 1 | -4.852 | 0.000184055 | down |
| MELO3C028091.2 | 5 | 0 | -4.993 | 0.015331509 | down |
| MELO3C028104.2 | 0 | 25 | 6.888 | 3.88824E-07 | up |
| MELO3C028107.2 | 3 | 28 | 3.228 | 0.000189846 | up |
| MELO3C028117.2 | 139 | 9 | -3.916 | 1.64297E-25 | down |
| MELO3C028118.2 | 9 | 0 | -5.791 | 0.000356572 | down |
| MELO3C028121.2 | 147 | 28 | -2.372 | 2.131E-08 | down |
| MELO3C028139.2 | 21 | 2 | -3.831 | 0.000364247 | down |
| MELO3C028143.2 | 54 | 15 | -1.8 | 0.003493625 | down |
| MELO3C028153.2 | 0 | 21 | 6.688 | 1.04729E-06 | up |
| MELO3C028155.2 | 9 | 0 | -4.695 | 0.003393203 | down |
| MELO3C028176.2 | 9 | 0 | -5.73 | 0.000456724 | down |
| MELO3C028190.2 | 1 | 12 | 3.983 | 0.002943415 | up |
| MELO3C028211.2 | 10 | 0 | -5.954 | 0.000149656 | down |
| MELO3C028251.2 | 13 | 1 | -4.425 | 0.001376645 | down |
| MELO3C028262.2 | 32 | 1 | -4.768 | 1.46697E-07 | down |
| MELO3C028265.2 | 222 | 73 | -1.594 | 1.37349E-12 | down |
| MELO3C028271.2 | 2 | 27 | 3.847 | 0.000176316 | up |
| MELO3C028274.2 | 0 | 10 | 4.682 | 0.001951442 | up |
| MELO3C028281.2 | 660 | 0 | -11.022 | 1.7776E-20 | down |
| MELO3C028301.2 | 12 | 50 | 2.068 | 1.41702E-05 | up |
| MELO3C028402.2 | 58 | 0 | -8.468 | 2.1877E-11 | down |
| MELO3C028422.2 | 13 | 0 | -6.254 | 3.65651E-05 | down |
| MELO3C028427.2 | 1 | 15 | 4.306 | 0.000869977 | up |
| MELO3C028430.2 | 0 | 4 | 4.437 | 0.022597461 | up |
| MELO3C028431.2 | 0 | 5 | 4.461 | 0.025898615 | up |
| MELO3C028442.2 | 1 | 42 | 5.814 | 4.40676E-07 | up |
| MELO3C028443.2 | 0 | 6 | 4.755 | 0.009553827 | up |
| MELO3C028449.2 | 672 | 310 | -1.116 | 1.03833E-13 | down |
| MELO3C028458.2 | 165 | 11 | -3.902 | 5.32774E-24 | down |
| MELO3C028461.2 | 29 | 12 | -1.301 | 0.023170428 | down |
| MELO3C028469.2 | 1 | 17 | 3.878 | 0.000495215 | up |
| MELO3C028471.2 | 7 | 100 | 3.887 | 2.08822E-17 | up |
| MELO3C028475.2 | 0 | 7 | 5.106 | 0.003939931 | up |
| MELO3C028484.2 | 0 | 47 | 7.826 | 8.95901E-10 | up |
| MELO3C028503.2 | 327 | 0 | -10.971 | 4.75199E-20 | down |
| MELO3C028506.2 | 34 | 0 | -6.737 | 2.23233E-07 | down |
| MELO3C028507.2 | 249 | 1 | -8.722 | 6.24678E-17 | down |
| MELO3C028511.2 | 40 | 18 | -1.199 | 0.008499571 | down |
| MELO3C028513.2 | 37 | 7 | -2.389 | 2.22139E-05 | down |
| MELO3C028515.2 | 0 | 49 | 7.894 | 6.32854E-10 | up |
| MELO3C028517.2 | 0 | 7 | 4.137 | 0.017182876 | up |
| MELO3C028519.2 | 31 | 0 | -7.545 | 1.48712E-08 | down |
| MELO3C028526.2 | 0 | 22 | 6.722 | 1.35217E-06 | up |
| MELO3C028530.2 | 94 | 0 | -9.172 | 1.34632E-13 | down |
| MELO3C028531.2 | 133 | 0 | -9.673 | 1.66632E-15 | down |
| MELO3C028535.2 | 0 | 598 | 11.502 | 5.34518E-22 | up |
| MELO3C028540.2 | 14 | 1 | -3.32 | 0.004893869 | down |
| MELO3C028543.2 | 0 | 53 | 8.01 | 1.92051E-10 | up |
| MELO3C028547.2 | 8 | 33 | 2.079 | 0.000674359 | up |
| MELO3C028550.2 | 1 | 19 | 4.085 | 0.000269759 | up |
| MELO3C028558.2 | 49 | 1 | -5.791 | 2.77198E-09 | down |
| MELO3C028562.2 | 40 | 0 | -7.92 | 3.16827E-09 | down |
| MELO3C028563.2 | 0 | 42 | 7.667 | 3.1428E-09 | up |
| MELO3C028566.2 | 6 | 0 | -5.225 | 0.00447796 | down |
| MELO3C028577.2 | 98 | 38 | -1.377 | 8.40499E-06 | down |
| MELO3C028584.2 | 48 | 11 | -2.216 | 8.18444E-06 | down |
| MELO3C028593.2 | 102 | 0 | -9.286 | 3.33667E-14 | down |
| MELO3C028594.2 | 16 | 54 | 1.766 | 0.000322054 | up |
| MELO3C028648.2 | 39 | 122 | 1.623 | 9.00391E-09 | up |
| MELO3C028652.2 | 28 | 0 | -7.417 | 2.61931E-08 | down |
| MELO3C028661.2 | 2 | 20 | 3.725 | 0.00046913 | up |
| MELO3C028666.2 | 43 | 4 | -3.58 | 2.50642E-06 | down |
| MELO3C028682.2 | 0 | 22 | 6.776 | 6.64703E-07 | up |
| MELO3C028687.2 | 6 | 0 | -4.254 | 0.014146975 | down |
| MELO3C028701.2 | 0 | 863 | 12.03 | 3.22484E-24 | up |
| MELO3C028713.2 | 0 | 23 | 6.818 | 5.00158E-06 | up |
| MELO3C028749.2 | 83 | 191 | 1.203 | 2.46342E-08 | up |
| MELO3C028763.2 | 0 | 10 | 5.557 | 0.000658047 | up |
| MELO3C028764.2 | 1 | 130 | 6.446 | 9.57443E-17 | up |
| MELO3C028773.2 | 1 | 28 | 5.239 | 9.52851E-06 | up |
| MELO3C028774.2 | 13 | 31 | 1.286 | 0.027697431 | up |
| MELO3C028779.2 | 183 | 0 | -10.127 | 4.74853E-17 | down |
| MELO3C028786.2 | 0 | 266 | 10.333 | 5.81764E-18 | up |
| MELO3C028788.2 | 0 | 48 | 6.903 | 1.35726E-07 | up |
| MELO3C028796.2 | 20 | 0 | -6.937 | 7.17886E-06 | down |
| MELO3C028797.2 | 1343 | 2730 | 1.024 | 6.2507E-30 | up |
| MELO3C028836.2 | 21 | 45 | 1.112 | 0.03249815 | up |
| MELO3C028846.2 | 13 | 3 | -2.248 | 0.018484681 | down |
| MELO3C028848.2 | 36 | 101 | 1.503 | 1.77683E-06 | up |
| MELO3C028852.2 | 238 | 61 | -1.969 | 4.79589E-19 | down |
| MELO3C028860.2 | 388 | 2690 | 2.792 | 5.8639E-80 | up |
| MELO3C028864.2 | 6 | 0 | -5.254 | 0.005484728 | down |
| MELO3C028865.2 | 0 | 18 | 6.441 | 1.34939E-05 | up |
| MELO3C028873.2 | 11 | 38 | 1.804 | 0.001008905 | up |
| MELO3C028881.2 | 30 | 0 | -7.519 | 1.58626E-08 | down |
| MELO3C028889.2 | 145 | 415 | 1.522 | 1.88224E-18 | up |
| MELO3C028898.2 | 8 | 40 | 2.36 | 9.18516E-05 | up |
| MELO3C028906.2 | 28 | 0 | -6.443 | 1.89095E-06 | down |
| MELO3C028921.2 | 0 | 28 | 6.109 | 4.10673E-06 | up |
| MELO3C028931.2 | 0 | 4 | 4.346 | 0.029610149 | up |
| MELO3C028959.2 | 294 | 12 | -4.606 | 8.25155E-53 | down |
| MELO3C028961.2 | 3 | 12 | 1.997 | 0.029759914 | up |
| MELO3C028967.2 | 0 | 63 | 8.256 | 6.46927E-10 | up |
| MELO3C028971.2 | 443 | 214 | -1.05 | 1.98758E-09 | down |
| MELO3C028978.2 | 36 | 73 | 1.039 | 0.012104578 | up |
| MELO3C028983.2 | 6 | 27 | 2.282 | 0.000852637 | up |
| MELO3C029003.2 | 43 | 164 | 1.946 | 2.22736E-14 | up |
| MELO3C029009.2 | 6 | 0 | -5.178 | 0.004421475 | down |
| MELO3C029038.2 | 11 | 30 | 1.493 | 0.005711191 | up |
| MELO3C029049.2 | 56 | 806 | 3.846 | 3.85099E-79 | up |
| MELO3C029054.2 | 11997 | 999 | -3.586 | 7.10084E-225 | down |
| MELO3C029067.2 | 16 | 0 | -5.624 | 7.33828E-05 | down |
| MELO3C029078.2 | 0 | 12 | 5.816 | 0.000132786 | up |
| MELO3C029083.2 | 539 | 200 | -1.432 | 1.61278E-22 | down |
| MELO3C029084.2 | 0 | 44 | 7.739 | 1.56241E-09 | up |
| MELO3C029093.2 | 32 | 77 | 1.234 | 0.000799068 | up |
| MELO3C029095.2 | 332 | 148 | -1.172 | 6.33316E-12 | down |
| MELO3C029112.2 | 0 | 339 | 10.682 | 4.00424E-19 | up |
| MELO3C029122.2 | 1 | 20 | 3.714 | 0.000229061 | up |
| MELO3C029124.2 | 0 | 210 | 9.996 | 9.05933E-17 | up |
| MELO3C029138.2 | 27 | 1 | -4.542 | 4.81237E-06 | down |
| MELO3C029141.2 | 7 | 0 | -5.33 | 0.004555002 | down |
| MELO3C029144.2 | 102 | 35 | -1.544 | 2.45146E-07 | down |
| MELO3C029147.2 | 0 | 58 | 8.134 | 1.08015E-10 | up |
| MELO3C029148.2 | 215 | 0 | -10.364 | 9.02947E-18 | down |
| MELO3C029161.2 | 287 | 0 | -10.779 | 2.19232E-19 | down |
| MELO3C029162.2 | 35 | 2 | -4.282 | 1.00008E-07 | down |
| MELO3C029163.2 | 0 | 348 | 10.72 | 2.4069E-19 | up |
| MELO3C029164.2 | 0 | 22 | 6.76 | 3.86679E-06 | up |
| MELO3C029165.2 | 8 | 0 | -5.595 | 0.001435589 | down |
| MELO3C029166.2 | 351 | 2813 | 3.003 | 2.81917E-175 | up |
| MELO3C029167.2 | 217 | 9 | -4.618 | 1.54939E-41 | down |
| MELO3C029171.2 | 2 | 70 | 5.224 | 2.21002E-11 | up |
| MELO3C029173.2 | 0 | 5 | 4.529 | 0.017986751 | up |
| MELO3C029177.2 | 1 | 10 | 3.777 | 0.007634469 | up |
| MELO3C029178.2 | 19 | 170 | 3.19 | 1.75471E-23 | up |
| MELO3C029185.2 | 24 | 228 | 3.275 | 8.38185E-33 | up |
| MELO3C029189.2 | 1 | 12 | 3.929 | 0.004747937 | up |
| MELO3C029196.2 | 0 | 37 | 7.516 | 6.16005E-09 | up |
| MELO3C029197.2 | 489 | 13 | -5.235 | 3.49046E-77 | down |
| MELO3C029198.2 | 82 | 0 | -8.014 | 1.13634E-10 | down |
| MELO3C029199.2 | 0 | 1220 | 12.53 | 4.38018E-26 | up |
| MELO3C029217.2 | 0 | 11 | 5.648 | 0.000270617 | up |
| MELO3C029218.2 | 10 | 1 | -3.477 | 0.005792626 | down |
| MELO3C029249.2 | 327 | 154 | -1.08 | 4.40528E-11 | down |
| MELO3C029252.2 | 68 | 8 | -3.17 | 1.14447E-11 | down |
| MELO3C029257.2 | 50 | 142 | 1.516 | 5.80614E-09 | up |
| MELO3C029266.2 | 13 | 138 | 3.453 | 1.45631E-20 | up |
| MELO3C029267.2 | 0 | 171 | 8.731 | 4.61442E-13 | up |
| MELO3C029269.2 | 12 | 34 | 1.486 | 0.008326853 | up |
| MELO3C029276.2 | 2 | 10 | 2.188 | 0.031480417 | up |
| MELO3C029279.2 | 26 | 281 | 3.447 | 1.43369E-34 | up |
| MELO3C029287.2 | 0 | 862 | 11.067 | 9.92533E-21 | up |
| MELO3C029292.2 | 38 | 0 | -7.87 | 1.08223E-09 | down |
| MELO3C029297.2 | 0 | 38 | 7.528 | 8.75632E-09 | up |
| MELO3C029301.2 | 90 | 15 | -2.559 | 1.47227E-11 | down |
| MELO3C029304.2 | 53 | 131 | 1.314 | 9.56756E-06 | up |
| MELO3C029317.2 | 912 | 7 | -6.979 | 2.75558E-15 | down |
| MELO3C029341.2 | 536 | 241 | -1.155 | 0.016657542 | down |
| MELO3C029344.2 | 38 | 236 | 2.627 | 1.15636E-21 | up |
| MELO3C029371.2 | 2 | 11 | 2.793 | 0.017303897 | up |
| MELO3C029378.2 | 12 | 57 | 2.23 | 3.83644E-06 | up |
| MELO3C029383.2 | 7 | 0 | -5.419 | 0.002516758 | down |
| MELO3C029386.2 | 45 | 4 | -3.464 | 5.50954E-09 | down |
| MELO3C029414.2 | 0 | 15 | 6.127 | 2.49961E-05 | up |
| MELO3C029430.2 | 185 | 42 | -2.131 | 6.10423E-17 | down |
| MELO3C029441.2 | 6 | 906 | 7.247 | 9.46561E-80 | up |
| MELO3C029447.2 | 8 | 0 | -5.596 | 0.000792467 | down |
| MELO3C029461.2 | 126 | 279 | 1.148 | 4.68218E-10 | up |
| MELO3C029472.2 | 1 | 20 | 3.764 | 0.000153848 | up |
| MELO3C029477.2 | 0 | 22 | 6.716 | 9.27362E-07 | up |
| MELO3C029487.2 | 1 | 32 | 5.426 | 3.92025E-06 | up |
| MELO3C029494.2 | 101 | 13 | -2.956 | 6.51984E-15 | down |
| MELO3C029503.2 | 0 | 11 | 4.829 | 0.001217079 | up |
| MELO3C029517.2 | 0 | 235 | 10.156 | 2.30307E-17 | up |
| MELO3C029522.2 | 15 | 2 | -3.137 | 0.001874806 | down |
| MELO3C029544.2 | 1 | 122 | 6.766 | 2.44555E-14 | up |
| MELO3C029545.2 | 1029 | 302 | -1.769 | 7.76332E-27 | down |
| MELO3C029549.2 | 0 | 519 | 11.296 | 1.97896E-21 | up |
| MELO3C029550.2 | 0 | 304 | 9.564 | 1.76101E-15 | up |
| MELO3C029551.2 | 0 | 17 | 6.339 | 8.79937E-06 | up |
| MELO3C029553.2 | 18 | 74 | 2.011 | 2.11531E-06 | up |
| MELO3C029555.2 | 52 | 20 | -1.41 | 0.000601265 | down |
| MELO3C029558.2 | 15 | 133 | 3.156 | 5.07042E-18 | up |
| MELO3C029559.2 | 0 | 239 | 10.175 | 2.13031E-17 | up |
| MELO3C029572.2 | 4 | 34 | 3.056 | 2.72075E-05 | up |
| MELO3C029575.2 | 485 | 67 | -2.845 | 2.27647E-49 | down |
| MELO3C029576.2 | 1 | 42 | 5.211 | 5.56087E-07 | up |
| MELO3C029580.2 | 12 | 1 | -3.794 | 0.002325516 | down |
| MELO3C029581.2 | 36 | 1 | -5.914 | 3.96022E-07 | down |
| MELO3C029585.2 | 0 | 5 | 4.677 | 0.018665214 | up |
| MELO3C029588.2 | 0 | 10 | 5.615 | 0.000402735 | up |
| MELO3C029590.2 | 466 | 0 | -11.479 | 4.87512E-22 | down |
| MELO3C029591.2 | 1095 | 3 | -8.402 | 1.08394E-76 | down |
| MELO3C029598.2 | 0 | 6 | 4.787 | 0.00972145 | up |
| MELO3C029610.2 | 10 | 26 | 1.431 | 0.011348233 | up |
| MELO3C029616.2 | 0 | 8 | 5.329 | 0.001379271 | up |
| MELO3C029624.2 | 18 | 1 | -3.884 | 0.000246439 | down |
| MELO3C029630.2 | 0 | 713 | 10.793 | 1.53763E-19 | up |
| MELO3C029631.2 | 107 | 413 | 1.946 | 1.16631E-19 | up |
| MELO3C029633.2 | 0 | 7 | 5.005 | 0.004639241 | up |
| MELO3C029634.2 | 59 | 27 | -1.167 | 0.003315406 | down |
| MELO3C029646.2 | 1 | 40 | 5.732 | 6.35538E-07 | up |
| MELO3C029647.2 | 0 | 31 | 7.219 | 3.84872E-08 | up |
| MELO3C029677.2 | 0 | 13 | 6.007 | 6.90057E-05 | up |
| MELO3C029692.2 | 0 | 1050 | 11.351 | 9.806E-22 | up |
| MELO3C029695.2 | 194 | 745 | 1.939 | 4.95663E-41 | up |
| MELO3C029714.2 | 0 | 5 | 4.506 | 0.018456468 | up |
| MELO3C029720.2 | 0 | 11 | 5.73 | 0.000312444 | up |
| MELO3C029737.2 | 7 | 0 | -5.442 | 0.001814323 | down |
| MELO3C029738.2 | 4 | 641 | 7.308 | 2.72198E-05 | up |
| MELO3C029746.2 | 0 | 114 | 9.108 | 9.15889E-14 | up |
| MELO3C029752.2 | 637 | 286 | -1.154 | 2.24102E-19 | down |
| MELO3C029757.2 | 25 | 49 | 1.009 | 0.010314409 | up |
| MELO3C029765.2 | 23 | 0 | -7.127 | 1.93495E-07 | down |
| MELO3C029773.2 | 7 | 22 | 1.524 | 0.019374449 | up |
| MELO3C029775.2 | 22 | 142 | 2.706 | 1.30462E-17 | up |
| MELO3C029795.2 | 0 | 10 | 5.611 | 0.000330047 | up |
| MELO3C029796.2 | 1 | 2069 | 11.436 | 6.46947E-29 | up |
| MELO3C029803.2 | 34 | 12 | -1.449 | 0.006752819 | down |
| MELO3C029831.2 | 1107 | 300 | -1.884 | 1.52528E-25 | down |
| MELO3C029842.2 | 37 | 85 | 1.198 | 0.007427657 | up |
| MELO3C029875.2 | 470 | 137 | -1.778 | 9.96154E-31 | down |
| MELO3C029895.2 | 2 | 15 | 2.804 | 0.012432669 | up |
| MELO3C029900.2 | 112 | 40 | -1.484 | 4.21434E-06 | down |
| MELO3C029915.2 | 0 | 10 | 5.567 | 0.00051903 | up |
| MELO3C029917.2 | 0 | 11 | 5.774 | 0.000188289 | up |
| MELO3C029922.2 | 9 | 0 | -5.821 | 0.000482523 | down |
| MELO3C029930.2 | 0 | 1228 | 12.54 | 5.04814E-26 | up |
| MELO3C029936.2 | 328 | 2 | -7.314 | 8.3201E-37 | down |
| MELO3C029940.2 | 2 | 16 | 3.099 | 0.001952099 | up |
| MELO3C029951.2 | 2 | 124 | 5.805 | 6.96748E-06 | up |
| MELO3C029968.2 | 87 | 2 | -5.887 | 1.05441E-15 | down |
| MELO3C029972.2 | 25 | 11 | -1.248 | 0.030682985 | down |
| MELO3C029991.2 | 0 | 1323 | 12.647 | 1.22962E-26 | up |
| MELO3C029997.2 | 18 | 1 | -3.913 | 8.42943E-05 | down |
| MELO3C030008.2 | 0 | 9 | 5.483 | 0.000617745 | up |
| MELO3C030014.2 | 13 | 0 | -6.343 | 2.53527E-05 | down |
| MELO3C030023.2 | 3 | 177 | 5.729 | 7.91713E-24 | up |
| MELO3C030026.2 | 1 | 257 | 8.426 | 6.13489E-16 | up |
| MELO3C030037.2 | 0 | 34 | 7.355 | 2.30317E-08 | up |
| MELO3C030060.2 | 247 | 928 | 1.911 | 7.47233E-59 | up |
| MELO3C030084.2 | 27 | 340 | 3.695 | 5.61743E-46 | up |
| MELO3C030102.2 | 137 | 36 | -1.91 | 6.4043E-09 | down |
| MELO3C030103.2 | 45 | 148 | 1.733 | 1.32043E-08 | up |
| MELO3C030106.2 | 26 | 126 | 2.264 | 4.24119E-10 | up |
| MELO3C030110.2 | 0 | 719 | 11.768 | 3.7831E-23 | up |
| MELO3C030113.2 | 0 | 469 | 11.152 | 6.71981E-21 | up |
| MELO3C030117.2 | 782 | 287 | -1.446 | 1.29188E-07 | down |
| MELO3C030119.2 | 6743 | 21682 | 1.685 | 1.49004E-129 | up |
| MELO3C030123.2 | 3 | 61 | 4.353 | 6.30831E-11 | up |
| MELO3C030125.2 | 49 | 0 | -8.222 | 9.84916E-11 | down |
| MELO3C030131.2 | 0 | 938 | 12.151 | 1.13555E-24 | up |
| MELO3C030135.2 | 0 | 50 | 7.92 | 3.69265E-10 | up |
| MELO3C030145.2 | 36 | 1 | -5.366 | 5.30787E-08 | down |
| MELO3C030151.2 | 229 | 0 | -10.453 | 3.34885E-18 | down |
| MELO3C030156.2 | 7 | 0 | -5.379 | 0.002369728 | down |
| MELO3C030163.2 | 0 | 32 | 7.268 | 2.90056E-08 | up |
| MELO3C030167.2 | 87 | 234 | 1.44 | 1.58969E-06 | up |
| MELO3C030175.2 | 18 | 4 | -1.97 | 0.023795352 | down |
| MELO3C030184.2 | 0 | 66 | 8.337 | 4.70098E-11 | up |
| MELO3C030198.2 | 4 | 0 | -4.77 | 0.015025123 | down |
| MELO3C030203.2 | 0 | 7 | 5.102 | 0.002673961 | up |
| MELO3C030204.2 | 35 | 1 | -5.872 | 8.47055E-07 | down |
| MELO3C030219.2 | 25 | 52 | 1.033 | 0.008054895 | up |
| MELO3C030221.2 | 34 | 92 | 1.43 | 3.32649E-06 | up |
| MELO3C030226.2 | 31 | 0 | -7.557 | 1.19447E-08 | down |
| MELO3C030228.2 | 0 | 936 | 12.149 | 1.38719E-24 | up |
| MELO3C030276.2 | 1 | 110 | 7.208 | 1.41135E-11 | up |
| MELO3C030280.2 | 3 | 28 | 3.039 | 4.78399E-05 | up |
| MELO3C030318.2 | 5 | 17 | 1.657 | 0.024311419 | up |
| MELO3C030319.2 | 207 | 684 | 1.728 | 1.02128E-29 | up |
| MELO3C030326.2 | 21 | 6 | -1.85 | 0.007144881 | down |
| MELO3C030333.2 | 0 | 7 | 4.007 | 0.016661734 | up |
| MELO3C030339.2 | 4182 | 938 | -2.157 | 2.07962E-11 | down |
| MELO3C030346.2 | 11 | 1 | -2.934 | 0.011035251 | down |
| MELO3C030348.2 | 14 | 0 | -5.452 | 0.000281041 | down |
| MELO3C030349.2 | 16 | 0 | -5.631 | 0.000171159 | down |
| MELO3C030350.2 | 894 | 1 | -10.565 | 2.36414E-24 | down |
| MELO3C030351.2 | 31 | 0 | -7.593 | 2.78003E-08 | down |
| MELO3C030358.2 | 293 | 44 | -2.722 | 7.816E-32 | down |
| MELO3C030359.2 | 126 | 0 | -8.628 | 1.19432E-12 | down |
| MELO3C030360.2 | 194 | 0 | -10.219 | 2.25264E-17 | down |
| MELO3C030365.2 | 11 | 0 | -5.076 | 0.001051499 | down |
| MELO3C030368.2 | 161 | 397 | 1.303 | 5.08306E-14 | up |
| MELO3C030370.2 | 0 | 29 | 7.116 | 1.19595E-07 | up |
| MELO3C030373.2 | 4 | 14 | 1.962 | 0.023427504 | up |
| MELO3C030375.2 | 3 | 83 | 4.472 | 3.40727E-14 | up |
| MELO3C030379.2 | 4 | 0 | -4.776 | 0.016486718 | down |
| MELO3C030383.2 | 7 | 0 | -5.373 | 0.001979321 | down |
| MELO3C030392.2 | 2 | 17 | 3.163 | 0.00071992 | up |
| MELO3C030396.2 | 792 | 5 | -7.228 | 4.6743E-84 | down |
| MELO3C030414.2 | 85 | 281 | 1.721 | 1.04273E-15 | up |
| MELO3C030429.2 | 162 | 26 | -2.651 | 1.79956E-19 | down |
| MELO3C030434.2 | 170 | 69 | -1.306 | 1.71542E-08 | down |
| MELO3C030436.2 | 54 | 1 | -6.515 | 6.48358E-09 | down |
| MELO3C030437.2 | 10 | 0 | -5.998 | 0.000137794 | down |
| MELO3C030440.2 | 0 | 103 | 8.967 | 2.5348E-13 | up |
| MELO3C030464.2 | 153 | 570 | 1.893 | 3.39504E-30 | up |
| MELO3C030468.2 | 8 | 23 | 1.464 | 0.018762813 | up |
| MELO3C030479.2 | 10 | 1 | -3.993 | 0.00572205 | down |
| MELO3C030491.2 | 0 | 723 | 11.776 | 3.83743E-23 | up |
| MELO3C030492.2 | 0 | 60 | 8.178 | 7.15426E-11 | up |
| MELO3C030496.2 | 122 | 5 | -4.522 | 3.74754E-24 | down |
| MELO3C030506.2 | 17 | 41 | 1.244 | 0.018660122 | up |
| MELO3C030512.2 | 33 | 0 | -7.669 | 5.54075E-09 | down |
| MELO3C030522.2 | 2230 | 440 | -2.342 | 2.53875E-149 | down |
| MELO3C030532.2 | 37 | 1 | -4.998 | 7.26154E-08 | down |
| MELO3C030540.2 | 60 | 153 | 1.362 | 0.00199954 | up |
| MELO3C030568.2 | 4 | 19 | 2.238 | 0.007297521 | up |
| MELO3C030576.2 | 945 | 1 | -10.062 | 8.03196E-33 | down |
| MELO3C030600.2 | 0 | 23 | 6.764 | 6.57259E-07 | up |
| MELO3C030602.2 | 0 | 23 | 6.798 | 1.07409E-06 | up |
| MELO3C030606.2 | 83 | 19 | -2.164 | 2.69591E-08 | down |
| MELO3C030623.2 | 4 | 0 | -4.552 | 0.031645611 | down |
| MELO3C030635.2 | 14 | 108 | 2.91 | 3.11934E-12 | up |
| MELO3C030650.2 | 0 | 5 | 4.605 | 0.014355575 | up |
| MELO3C030651.2 | 0 | 2232 | 13.402 | 8.83198E-30 | up |
| MELO3C030665.2 | 6 | 32 | 2.316 | 7.5714E-05 | up |
| MELO3C030668.2 | 240 | 22 | -3.463 | 1.44952E-17 | down |
| MELO3C030672.2 | 5 | 95 | 4.188 | 3.08747E-15 | up |
| MELO3C030675.2 | 0 | 706 | 11.74 | 4.4132E-23 | up |
| MELO3C030676.2 | 405 | 9 | -5.429 | 1.49201E-65 | down |
| MELO3C030695.2 | 0 | 1430 | 12.76 | 4.36117E-27 | up |
| MELO3C030699.2 | 105 | 259 | 1.304 | 2.25631E-09 | up |
| MELO3C030704.2 | 430 | 186 | -1.21 | 1.11398E-14 | down |
| MELO3C030711.2 | 45 | 16 | -1.513 | 0.002861275 | down |
| MELO3C030719.2 | 1100 | 229 | -2.269 | 1.01046E-33 | down |
| MELO3C030720.2 | 22 | 4 | -2.361 | 0.006966096 | down |
| MELO3C030721.2 | 1018 | 2508 | 1.301 | 0.000101624 | up |
| MELO3C030722.2 | 709 | 1814 | 1.356 | 0.000160643 | up |
| MELO3C030723.2 | 0 | 893 | 12.08 | 2.3414E-24 | up |
| MELO3C030724.2 | 10 | 0 | -6.006 | 0.000127737 | down |
| MELO3C030730.2 | 2 | 20 | 3.117 | 0.001878984 | up |
| MELO3C030737.2 | 1245 | 2873 | 1.206 | 2.4262E-35 | up |
| MELO3C030740.2 | 9 | 45 | 2.337 | 7.77488E-06 | up |
| MELO3C030745.2 | 0 | 17 | 6.333 | 1.67039E-05 | up |
| MELO3C030747.2 | 14 | 113 | 3.04 | 3.57669E-16 | up |
| MELO3C030748.2 | 9 | 97 | 3.499 | 7.27178E-16 | up |
| MELO3C030758.2 | 0 | 39 | 7.558 | 6.1516E-09 | up |
| MELO3C030768.2 | 0 | 86 | 8.699 | 1.4356E-12 | up |
| MELO3C030769.2 | 0 | 39 | 7.574 | 5.20667E-09 | up |
| MELO3C030770.2 | 102 | 18 | -2.519 | 1.13856E-09 | down |
| MELO3C030784.2 | 139 | 0 | -9.735 | 1.31603E-15 | down |
| MELO3C030787.2 | 10 | 38 | 1.915 | 0.001648062 | up |
| MELO3C030789.2 | 0 | 33 | 7.298 | 2.28279E-08 | up |
| MELO3C030795.2 | 0 | 547 | 11.374 | 1.06349E-21 | up |
| MELO3C030799.2 | 14 | 131 | 3.235 | 2.14122E-15 | up |
| MELO3C030800.2 | 114 | 29 | -1.997 | 3.81571E-08 | down |
| MELO3C030828.2 | 615 | 0 | -11.881 | 1.38906E-23 | down |
| MELO3C030845.2 | 0 | 23 | 6.821 | 5.86652E-07 | up |
| MELO3C030854.2 | 2 | 97 | 5.686 | 2.62142E-14 | up |
| MELO3C030856.2 | 0 | 449 | 11.088 | 1.41142E-20 | up |
| MELO3C030862.2 | 0 | 12 | 4.823 | 0.001304383 | up |
| MELO3C030865.2 | 88 | 270 | 1.63 | 6.3525E-15 | up |
| MELO3C030869.2 | 15 | 381 | 4.638 | 1.19815E-60 | up |
| MELO3C030870.2 | 0 | 6 | 4.863 | 0.006615499 | up |
| MELO3C030890.2 | 171 | 80 | -1.099 | 3.44346E-06 | down |
| MELO3C030893.2 | 289 | 71 | -2.018 | 3.74467E-25 | down |
| MELO3C030897.2 | 8 | 491 | 5.905 | 1.61835E-63 | up |
| MELO3C030898.2 | 0 | 13 | 4.968 | 0.00076889 | up |
| MELO3C030900.2 | 22 | 0 | -7.074 | 3.83797E-07 | down |
| MELO3C030902.2 | 190 | 0 | -10.185 | 2.67735E-17 | down |
| MELO3C030927.2 | 1 | 15 | 3.371 | 0.001946895 | up |
| MELO3C030936.2 | 136 | 46 | -1.554 | 1.08135E-05 | down |
| MELO3C030939.2 | 0 | 8 | 5.239 | 0.001731847 | up |
| MELO3C030950.2 | 196 | 28 | -2.81 | 7.59605E-26 | down |
| MELO3C030959.2 | 586 | 207 | -1.498 | 6.35801E-22 | down |
| MELO3C030960.2 | 17 | 43 | 1.339 | 0.002246779 | up |
| MELO3C030968.2 | 5 | 159 | 4.921 | 2.07371E-26 | up |
| MELO3C030969.2 | 2 | 15 | 3.022 | 0.004777657 | up |
| MELO3C030970.2 | 4 | 0 | -4.636 | 0.022926636 | down |
| MELO3C030992.2 | 6 | 52 | 3.026 | 1.07198E-08 | up |
| MELO3C031014.2 | 1581 | 229 | -2.782 | 2.19984E-73 | down |
| MELO3C031020.2 | 132 | 289 | 1.128 | 1.00488E-09 | up |
| MELO3C031023.2 | 275 | 701 | 1.347 | 1.17572E-13 | up |
| MELO3C031024.2 | 178 | 410 | 1.206 | 5.17377E-10 | up |
| MELO3C031032.2 | 16 | 53 | 1.724 | 0.000570683 | up |
| MELO3C031036.2 | 18 | 38 | 1.109 | 0.016609463 | up |
| MELO3C031041.2 | 321 | 803 | 1.323 | 1.34673E-28 | up |
| MELO3C031050.2 | 70 | 175 | 1.313 | 2.39913E-06 | up |
| MELO3C031052.2 | 0 | 165 | 9.64 | 2.13876E-15 | up |
| MELO3C031055.2 | 2 | 25 | 3.725 | 0.000104724 | up |
| MELO3C031059.2 | 0 | 5 | 4.64 | 0.013825291 | up |
| MELO3C031071.2 | 836 | 193 | -2.118 | 2.12974E-28 | down |
| MELO3C031072.2 | 779 | 2284 | 1.551 | 4.40503E-27 | up |
| MELO3C031073.2 | 11 | 0 | -6.083 | 0.000449887 | down |
| MELO3C031083.2 | 158 | 399 | 1.335 | 2.25469E-14 | up |
| MELO3C031091.2 | 8 | 43 | 2.378 | 5.57037E-05 | up |
| MELO3C031122.2 | 144 | 423 | 1.546 | 7.80425E-17 | up |
| MELO3C031125.2 | 593 | 0 | -11.828 | 2.4611E-23 | down |
| MELO3C031130.2 | 51 | 0 | -8.28 | 7.645E-11 | down |
| MELO3C031131.2 | 39 | 0 | -7.907 | 1.18279E-09 | down |
| MELO3C031140.2 | 1 | 9 | 3.577 | 0.018049233 | up |
| MELO3C031144.2 | 11 | 30 | 1.473 | 0.009878908 | up |
| MELO3C031147.2 | 5 | 0 | -4.156 | 0.017627627 | down |
| MELO3C031169.2 | 73 | 221 | 1.602 | 5.9132E-10 | up |
| MELO3C031184.2 | 28 | 3 | -3.106 | 1.84122E-05 | down |
| MELO3C031189.2 | 0 | 10 | 5.603 | 0.000424248 | up |
| MELO3C031210.2 | 3 | 0 | -4.503 | 0.03135504 | down |
| MELO3C031214.2 | 530 | 2 | -8.231 | 2.45961E-41 | down |
| MELO3C031216.2 | 1 | 33 | 4.863 | 9.95707E-07 | up |
| MELO3C031217.2 | 16 | 4 | -2.101 | 0.024953131 | down |
| MELO3C031219.2 | 238 | 50 | -2.238 | 1.46742E-17 | down |
| MELO3C031220.2 | 29 | 4 | -2.965 | 5.40783E-05 | down |
| MELO3C031229.2 | 16 | 2 | -3.024 | 0.002185398 | down |
| MELO3C031246.2 | 37 | 2 | -4.107 | 5.59005E-07 | down |
| MELO3C031269.2 | 117 | 0 | -9.491 | 6.40357E-15 | down |
| MELO3C031290.2 | 1 | 14 | 4.228 | 0.002073171 | up |
| MELO3C031294.2 | 0 | 77 | 8.547 | 5.33379E-12 | up |
| MELO3C031304.2 | 11 | 0 | -5.092 | 0.000735175 | down |
| MELO3C031322.2 | 4 | 1334 | 8.481 | 4.94218E-71 | up |
| MELO3C031323.2 | 165 | 867 | 2.388 | 5.095E-38 | up |
| MELO3C031324.2 | 17 | 45 | 1.463 | 0.000700497 | up |
| MELO3C031330.2 | 2001 | 349 | -2.52 | 3.22627E-43 | down |
| MELO3C031334.2 | 1 | 18 | 4.595 | 0.000334274 | up |
| MELO3C031335.2 | 0 | 6 | 4.838 | 0.006686954 | up |
| MELO3C031339.2 | 266 | 567 | 1.092 | 1.04314E-13 | up |
| MELO3C031340.2 | 19 | 0 | -5.914 | 2.61617E-05 | down |
| MELO3C031341.2 | 269 | 0 | -10.689 | 4.08483E-19 | down |
| MELO3C031344.2 | 15 | 3 | -2.565 | 0.007290829 | down |
| MELO3C031355.2 | 6 | 31 | 2.459 | 5.90887E-05 | up |
| MELO3C031365.2 | 617 | 1251 | 1.019 | 8.34315E-22 | up |
| MELO3C031367.2 | 5 | 0 | -5.007 | 0.009340421 | down |
| MELO3C031371.2 | 30 | 0 | -7.555 | 1.94016E-08 | down |
| MELO3C031372.2 | 134 | 0 | -9.681 | 1.8261E-15 | down |
| MELO3C031379.2 | 71 | 30 | -1.238 | 0.001236568 | down |
| MELO3C031382.2 | 36 | 10 | -1.86 | 0.000480809 | down |
| MELO3C031411.2 | 0 | 14 | 6.115 | 2.88029E-05 | up |
| MELO3C031416.2 | 0 | 27 | 7.063 | 1.11203E-07 | up |
| MELO3C031417.2 | 0 | 8 | 5.297 | 0.001540021 | up |
| MELO3C031424.2 | 39 | 11 | -1.784 | 0.001784996 | down |
| MELO3C031425.2 | 49 | 8 | -2.663 | 6.88139E-06 | down |
| MELO3C031435.2 | 0 | 8 | 5.327 | 0.001167873 | up |
| MELO3C031437.2 | 65 | 0 | -8.644 | 3.85943E-12 | down |
| MELO3C031442.2 | 12 | 2 | -2.721 | 0.006715412 | down |
| MELO3C031443.2 | 39 | 7 | -2.521 | 7.63441E-06 | down |
| MELO3C031450.2 | 0 | 13 | 5.902 | 8.66233E-05 | up |
| MELO3C031472.2 | 0 | 269 | 10.352 | 4.80923E-18 | up |
| MELO3C031484.2 | 17 | 3 | -2.542 | 0.002173834 | down |
| MELO3C031486.2 | 1 | 27 | 5.153 | 9.38843E-05 | up |
| MELO3C031487.2 | 993 | 1 | -10.718 | 1.81077E-25 | down |
| MELO3C031496.2 | 3 | 29 | 3.098 | 2.11465E-05 | up |
| MELO3C031513.2 | 0 | 8 | 5.3 | 0.001709379 | up |
| MELO3C031540.2 | 0 | 146 | 9.467 | 2.69797E-14 | up |
| MELO3C031543.2 | 0 | 622 | 10.597 | 4.66572E-19 | up |
| MELO3C031548.2 | 36 | 80 | 1.161 | 0.000524609 | up |
| MELO3C031556.2 | 0 | 7 | 5.045 | 0.003336572 | up |
| MELO3C031557.2 | 0 | 21 | 5.747 | 2.72512E-05 | up |
| MELO3C031564.2 | 14 | 101 | 2.846 | 2.45983E-10 | up |
| MELO3C031571.2 | 62 | 27 | -1.227 | 0.003272755 | down |
| MELO3C031573.2 | 10 | 27 | 1.413 | 0.021500219 | up |
| MELO3C031600.2 | 66 | 206 | 1.655 | 2.74398E-11 | up |
| MELO3C031601.2 | 427 | 5366 | 3.655 | 2.8024E-128 | up |
| MELO3C031602.2 | 455 | 975 | 1.098 | 1.66674E-17 | up |
| MELO3C031618.2 | 20 | 72 | 1.836 | 9.35627E-06 | up |
| MELO3C031621.2 | 6 | 26 | 2.111 | 0.001975229 | up |
| MELO3C031623.2 | 6 | 23 | 1.846 | 0.004095201 | up |
| MELO3C031644.2 | 4 | 24 | 2.567 | 0.000558971 | up |
| MELO3C031645.2 | 0 | 124 | 9.228 | 3.54134E-14 | up |
| MELO3C031662.2 | 14 | 138 | 3.319 | 6.91079E-22 | up |
| MELO3C031667.2 | 9 | 84 | 3.208 | 9.94074E-11 | up |
| MELO3C031670.2 | 321 | 130 | -1.307 | 8.51637E-07 | down |
| MELO3C031675.2 | 1 | 19 | 4.083 | 0.000191249 | up |
| MELO3C031717.2 | 905 | 0 | -12.438 | 9.31199E-26 | down |
| MELO3C031722.2 | 5 | 19 | 1.879 | 0.010432172 | up |
| MELO3C031755.2 | 277 | 20 | -3.813 | 1.3367E-48 | down |
| MELO3C031769.2 | 21 | 7 | -1.704 | 0.012056938 | down |
| MELO3C031779.2 | 3 | 209 | 5.951 | 7.01232E-27 | up |
| MELO3C031780.2 | 50 | 507 | 3.345 | 6.45535E-37 | up |
| MELO3C031785.2 | 1 | 12 | 4.01 | 0.003160666 | up |
| MELO3C031801.2 | 49 | 149 | 1.607 | 2.73475E-10 | up |
| MELO3C031806.2 | 0 | 179 | 9.765 | 6.72863E-16 | up |
| MELO3C031809.2 | 0 | 3627 | 13.141 | 1.25578E-28 | up |
| MELO3C031812.2 | 1 | 234 | 8.294 | 3.70412E-15 | up |
| MELO3C031820.2 | 18 | 66 | 1.867 | 3.34403E-06 | up |
| MELO3C031824.2 | 204 | 96 | -1.088 | 3.64096E-05 | down |
| MELO3C031839.2 | 0 | 47 | 7.84 | 9.04791E-10 | up |
| MELO3C031840.2 | 0 | 29 | 6.192 | 3.06127E-06 | up |
| MELO3C031857.2 | 5195 | 243 | -4.415 | 1.66463E-35 | down |
| MELO3C031858.2 | 87 | 182 | 1.068 | 2.96926E-05 | up |
| MELO3C031859.2 | 894 | 141 | -2.658 | 1.84556E-74 | down |
| MELO3C031870.2 | 3496 | 1325 | -1.399 | 8.35905E-45 | down |
| MELO3C031876.2 | 159 | 1 | -7.082 | 4.54272E-20 | down |
| MELO3C031893.2 | 21 | 1 | -4.61 | 2.23454E-05 | down |
| MELO3C031894.2 | 1993 | 0 | -13.577 | 1.88749E-30 | down |
| MELO3C031895.2 | 1536 | 0 | -13.201 | 6.42021E-29 | down |
| MELO3C031896.2 | 770 | 0 | -12.205 | 8.03775E-25 | down |
| MELO3C031906.2 | 0 | 29 | 7.129 | 9.30106E-08 | up |
| MELO3C031911.2 | 2258 | 4919 | 1.124 | 1.72463E-37 | up |
| MELO3C031924.2 | 2380 | 1179 | -1.014 | 2.038E-25 | down |
| MELO3C031926.2 | 0 | 66 | 8.328 | 3.86777E-11 | up |
| MELO3C031934.2 | 0 | 4 | 4.392 | 0.030070083 | up |
| MELO3C031942.2 | 0 | 82 | 8.641 | 5.19696E-11 | up |
| MELO3C031943.2 | 0 | 29 | 7.157 | 1.16377E-07 | up |
| MELO3C031945.2 | 23 | 2 | -3.515 | 2.48425E-05 | down |
| MELO3C031947.2 | 0 | 109 | 9.042 | 1.19146E-13 | up |
| MELO3C031963.2 | 0 | 6 | 4.817 | 0.009480926 | up |
| MELO3C031964.2 | 0 | 6 | 4.786 | 0.029333015 | up |
| MELO3C031972.2 | 0 | 351 | 10.731 | 2.53886E-19 | up |
| MELO3C032000.2 | 36 | 92 | 1.346 | 1.54096E-05 | up |
| MELO3C032006.2 | 1 | 21 | 4.221 | 0.000182875 | up |
| MELO3C032024.2 | 1 | 13 | 3.521 | 0.004726396 | up |
| MELO3C032026.2 | 7 | 0 | -5.498 | 0.002549991 | down |
| MELO3C032039.2 | 128 | 62 | -1.046 | 0.000136022 | down |
| MELO3C032043.2 | 3 | 15 | 2.33 | 0.00885165 | up |
| MELO3C032061.2 | 244 | 741 | 1.602 | 1.98295E-35 | up |
| MELO3C032067.2 | 0 | 18 | 6.474 | 5.00924E-06 | up |
| MELO3C032074.2 | 21 | 53 | 1.357 | 0.002755273 | up |
| MELO3C032089.2 | 175 | 27 | -2.676 | 2.02611E-20 | down |
| MELO3C032096.2 | 18 | 58 | 1.728 | 0.001520169 | up |
| MELO3C032097.2 | 39 | 10 | -1.92 | 0.013737788 | down |
| MELO3C032108.2 | 0 | 43 | 7.724 | 1.61212E-09 | up |
| MELO3C032111.2 | 14 | 47 | 1.743 | 0.003154143 | up |
| MELO3C032112.2 | 51 | 3 | -4.123 | 3.57033E-10 | down |
| MELO3C032130.2 | 8 | 0 | -5.586 | 0.002247497 | down |
| MELO3C032145.2 | 3 | 23 | 3.099 | 0.000261662 | up |
| MELO3C032146.2 | 58 | 148 | 1.35 | 1.10523E-06 | up |
| MELO3C032149.2 | 0 | 52 | 7.977 | 2.49259E-10 | up |
| MELO3C032169.2 | 13 | 0 | -5.286 | 0.000353609 | down |
| MELO3C032170.2 | 0 | 6 | 4.799 | 0.029861518 | up |
| MELO3C032178.2 | 0 | 36 | 7.466 | 7.64685E-09 | up |
| MELO3C032187.2 | 653 | 217 | -1.582 | 6.04609E-10 | down |
| MELO3C032192.2 | 7 | 48 | 2.772 | 6.62552E-07 | up |
| MELO3C032203.2 | 9 | 0 | -5.872 | 0.000499178 | down |
| MELO3C032207.2 | 1 | 26 | 5.116 | 4.18148E-05 | up |
| MELO3C032208.2 | 1 | 15 | 4.337 | 0.00106938 | up |
| MELO3C032227.2 | 0 | 15 | 6.174 | 2.09564E-05 | up |
| MELO3C032234.2 | 6 | 0 | -4.316 | 0.010537783 | down |
| MELO3C032255.2 | 5 | 16 | 1.614 | 0.028427312 | up |
| MELO3C032269.2 | 86 | 10 | -3.186 | 3.12194E-13 | down |
| MELO3C032271.2 | 0 | 21 | 6.669 | 1.63983E-06 | up |
| MELO3C032273.2 | 4 | 0 | -4.779 | 0.014761153 | down |
| MELO3C032281.2 | 1 | 368 | 7.944 | 3.31944E-26 | up |
| MELO3C032285.2 | 5 | 88 | 4.073 | 1.13397E-13 | up |
| MELO3C032290.2 | 0 | 4 | 4.354 | 0.032097458 | up |
| MELO3C032295.2 | 6 | 32 | 2.345 | 0.000134844 | up |
| MELO3C032296.2 | 158 | 71 | -1.143 | 4.94124E-07 | down |
| MELO3C032302.2 | 132 | 48 | -1.438 | 7.83566E-07 | down |
| MELO3C032307.2 | 70 | 0 | -8.762 | 4.25383E-12 | down |
| MELO3C032309.2 | 36 | 176 | 2.292 | 1.04088E-15 | up |
| MELO3C032312.2 | 4 | 169 | 5.13 | 4.94771E-27 | up |
| MELO3C032315.2 | 976 | 250 | -1.963 | 3.11488E-32 | down |
| MELO3C032319.2 | 11 | 0 | -5.973 | 0.000210554 | down |
| MELO3C032320.2 | 27 | 80 | 1.564 | 5.61895E-05 | up |
| MELO3C032323.2 | 39 | 79 | 1.026 | 0.008115368 | up |
| MELO3C032330.2 | 247 | 97 | -1.345 | 9.15093E-05 | down |
| MELO3C032345.2 | 8 | 3160 | 8.587 | 8.22471E-158 | up |
| MELO3C032348.2 | 157 | 54 | -1.556 | 2.60294E-10 | down |
| MELO3C032352.2 | 4 | 19 | 2.283 | 0.005720293 | up |
| MELO3C032353.2 | 53 | 9 | -2.585 | 7.02792E-07 | down |
| MELO3C032356.2 | 1 | 17 | 3.854 | 0.001029011 | up |
| MELO3C032359.2 | 8 | 65 | 2.975 | 1.08256E-07 | up |
| MELO3C032387.2 | 67 | 0 | -8.673 | 3.44775E-12 | down |
| MELO3C032396.2 | 23 | 57 | 1.33 | 0.003905314 | up |
| MELO3C032410.2 | 0 | 27 | 6.073 | 6.09196E-06 | up |
| MELO3C032429.2 | 40 | 118 | 1.566 | 8.18765E-07 | up |
| MELO3C032437.2 | 0 | 122 | 9.216 | 3.81131E-14 | up |
| MELO3C032441.2 | 9 | 27 | 1.675 | 0.005107941 | up |
| MELO3C032442.2 | 0 | 536 | 11.343 | 1.92695E-21 | up |
| MELO3C032454.2 | 1374 | 278 | -2.301 | 9.95472E-72 | down |
| MELO3C032461.2 | 369 | 1319 | 1.836 | 1.49145E-25 | up |
| MELO3C032462.2 | 7 | 0 | -5.417 | 0.002469269 | down |
| MELO3C032467.2 | 11 | 130 | 3.534 | 1.4331E-19 | up |
| MELO3C032468.2 | 0 | 11 | 5.712 | 0.000249927 | up |
| MELO3C032469.2 | 0 | 10 | 5.505 | 0.000658115 | up |
| MELO3C032497.2 | 12 | 417 | 5.172 | 7.51683E-53 | up |
| MELO3C032507.2 | 179 | 72 | -1.309 | 4.80032E-07 | down |
| MELO3C032516.2 | 1006 | 236 | -2.088 | 1.07698E-61 | down |
| MELO3C032523.2 | 72 | 1 | -6.338 | 1.96648E-11 | down |
| MELO3C032530.2 | 29 | 4 | -2.836 | 5.27031E-05 | down |
| MELO3C032537.2 | 68 | 1 | -6.862 | 5.67645E-10 | down |
| MELO3C032579.2 | 1 | 92 | 5.944 | 1.99949E-12 | up |
| MELO3C032602.2 | 300 | 1009 | 1.749 | 1.63418E-35 | up |
| MELO3C032603.2 | 221 | 798 | 1.856 | 2.041E-31 | up |
| MELO3C032612.2 | 8 | 0 | -5.71 | 0.000548143 | down |
| MELO3C032618.2 | 874 | 429 | -1.027 | 2.22179E-06 | down |
| MELO3C032621.2 | 34 | 1 | -5.835 | 5.30204E-07 | down |
| MELO3C032627.2 | 68 | 137 | 1.014 | 4.00752E-05 | up |
| MELO3C032630.2 | 206 | 66 | -1.635 | 7.22568E-07 | down |
| MELO3C032635.2 | 42 | 2 | -4.583 | 2.27296E-08 | down |
| MELO3C032637.2 | 11 | 0 | -6.101 | 9.34816E-05 | down |
| MELO3C032639.2 | 111 | 506 | 2.181 | 1.35403E-26 | up |
| MELO3C032659.2 | 9 | 0 | -4.869 | 0.001915348 | down |
| MELO3C032661.2 | 0 | 2103 | 13.315 | 2.60538E-29 | up |
| MELO3C032662.2 | 0 | 53 | 8.012 | 2.38676E-10 | up |
| MELO3C032663.2 | 32156 | 13903 | -1.21 | 2.539E-30 | down |
| MELO3C032665.2 | 0 | 51 | 6.984 | 3.43868E-08 | up |
| MELO3C032674.2 | 7 | 0 | -5.349 | 0.00260178 | down |
| MELO3C032689.2 | 1 | 61 | 5.347 | 1.83187E-10 | up |
| MELO3C032690.2 | 0 | 48 | 7.861 | 5.96754E-10 | up |
| MELO3C032692.2 | 20 | 171 | 3.093 | 1.55517E-17 | up |
| MELO3C032707.2 | 30 | 0 | -7.498 | 1.43461E-08 | down |
| MELO3C032711.2 | 16 | 5 | -1.713 | 0.022829187 | down |
| MELO3C032717.2 | 0 | 7 | 5.058 | 0.003266079 | up |
| MELO3C032718.2 | 0 | 131 | 9.307 | 2.1722E-14 | up |
| MELO3C032731.2 | 67 | 0 | -8.671 | 7.53641E-12 | down |
| MELO3C032733.2 | 30 | 0 | -7.529 | 1.55661E-08 | down |
| MELO3C032739.2 | 663 | 3 | -7.818 | 3.27898E-55 | down |
| MELO3C032740.2 | 0 | 69 | 8.373 | 1.53936E-11 | up |
| MELO3C032750.2 | 321 | 126 | -1.352 | 3.33349E-14 | down |
| MELO3C032760.2 | 20 | 0 | -5.944 | 2.14734E-05 | down |
| MELO3C032766.2 | 9 | 641 | 6.163 | 2.10401E-78 | up |
| MELO3C032776.2 | 1 | 13 | 3.486 | 0.003223826 | up |
| MELO3C032778.2 | 16 | 61 | 1.893 | 1.08688E-05 | up |
| MELO3C032779.2 | 14 | 58 | 1.998 | 1.59467E-05 | up |
| MELO3C032784.2 | 97 | 3 | -4.777 | 9.51444E-20 | down |
| MELO3C032787.2 | 44 | 6 | -2.823 | 2.06537E-05 | down |
| MELO3C032794.2 | 22 | 75 | 1.758 | 3.32483E-06 | up |
| MELO3C032807.2 | 15 | 37 | 1.333 | 0.006835932 | up |
| MELO3C032809.2 | 125 | 41 | -1.603 | 1.07137E-08 | down |
| MELO3C032813.2 | 6 | 0 | -4.202 | 0.021124182 | down |
| MELO3C032818.2 | 55 | 123 | 1.174 | 1.12988E-05 | up |
| MELO3C032824.2 | 0 | 8 | 4.303 | 0.028917283 | up |
| MELO3C032833.2 | 2 | 23 | 3.142 | 0.000106686 | up |
| MELO3C032841.2 | 2215 | 8163 | 1.882 | 4.59573E-47 | up |
| MELO3C032847.2 | 166 | 55 | -1.604 | 1.82243E-09 | down |
| MELO3C032851.2 | 40 | 6 | -2.841 | 3.62007E-07 | down |
| MELO3C032857.2 | 893 | 439 | -1.024 | 3.61767E-22 | down |
| MELO3C032861.2 | 0 | 5 | 4.537 | 0.017446887 | up |
| MELO3C032862.2 | 68 | 234 | 1.783 | 3.16167E-14 | up |
| MELO3C032868.2 | 162 | 328 | 1.014 | 4.39881E-07 | up |
| MELO3C032874.2 | 52 | 1 | -5.867 | 1.61038E-08 | down |
| MELO3C032879.2 | 51 | 106 | 1.056 | 0.000106787 | up |
| MELO3C032881.2 | 325 | 0 | -10.959 | 4.86935E-20 | down |
| MELO3C032882.2 | 41 | 6 | -2.891 | 1.13608E-06 | down |
| MELO3C032883.2 | 7 | 36 | 2.315 | 9.47615E-05 | up |
| MELO3C032888.2 | 9 | 41 | 2.226 | 0.000101245 | up |
| MELO3C032889.2 | 208 | 491 | 1.242 | 6.61968E-17 | up |
| MELO3C032903.2 | 48 | 16 | -1.566 | 0.000340492 | down |
| MELO3C032910.2 | 401 | 73 | -2.447 | 2.64588E-20 | down |
| MELO3C032917.2 | 42 | 2 | -4.83 | 7.51066E-09 | down |
| MELO3C032939.2 | 14 | 1 | -4.635 | 0.000558497 | down |
| MELO3C032940.2 | 0 | 7 | 5.025 | 0.003613009 | up |
| MELO3C032943.2 | 2 | 28 | 3.907 | 8.13253E-06 | up |
| MELO3C032950.2 | 0 | 50 | 7.934 | 9.29252E-10 | up |
| MELO3C032955.2 | 726 | 233 | -1.639 | 1.46754E-28 | down |
| MELO3C032956.2 | 0 | 6 | 4.884 | 0.00626421 | up |
| MELO3C032967.2 | 1 | 333 | 8.798 | 1.12861E-16 | up |
| MELO3C032980.2 | 0 | 7 | 5.156 | 0.002169357 | up |
| MELO3C032996.2 | 0 | 54 | 8.039 | 2.80084E-10 | up |
| MELO3C033019.2 | 15 | 49 | 1.769 | 0.000323833 | up |
| MELO3C033024.2 | 67 | 13 | -2.399 | 1.23789E-08 | down |
| MELO3C033047.2 | 0 | 5 | 4.562 | 0.021028697 | up |
| MELO3C033048.2 | 0 | 393 | 10.895 | 7.98166E-20 | up |
| MELO3C033051.2 | 78 | 0 | -8.905 | 7.00826E-13 | down |
| MELO3C033058.2 | 235 | 110 | -1.088 | 0.000192832 | down |
| MELO3C033070.2 | 0 | 69 | 8.379 | 1.44238E-11 | up |
| MELO3C033071.2 | 1 | 12 | 4.021 | 0.003537945 | up |
| MELO3C033082.2 | 39 | 113 | 1.521 | 1.12816E-07 | up |
| MELO3C033086.2 | 3 | 26 | 2.935 | 8.22348E-05 | up |
| MELO3C033094.2 | 0 | 1867 | 13.144 | 5.94756E-26 | up |
| MELO3C033095.2 | 0 | 8043 | 15.251 | 9.46522E-38 | up |
| MELO3C033117.2 | 7 | 0 | -5.423 | 0.001944078 | down |
| MELO3C033123.2 | 0 | 69 | 8.376 | 1.50905E-11 | up |
| MELO3C033124.2 | 0 | 679 | 11.685 | 8.06157E-23 | up |
| MELO3C033125.2 | 17 | 1 | -4.307 | 0.000281596 | down |
| MELO3C033155.2 | 0 | 47 | 7.829 | 7.05717E-10 | up |
| MELO3C033157.2 | 301 | 53 | -2.488 | 1.48384E-11 | down |
| MELO3C033158.2 | 8 | 22 | 1.579 | 0.0126344 | up |
| MELO3C033165.2 | 0 | 18 | 6.413 | 6.84702E-06 | up |
| MELO3C033192.2 | 265 | 108 | -1.292 | 7.46282E-12 | down |
| MELO3C033195.2 | 2 | 17 | 2.922 | 0.001729148 | up |
| MELO3C033197.2 | 649 | 2211 | 1.77 | 8.3308E-22 | up |
| MELO3C033209.2 | 17 | 55 | 1.741 | 8.58103E-05 | up |
| MELO3C033219.2 | 0 | 387 | 10.873 | 8.87461E-20 | up |
| MELO3C033220.2 | 0 | 117 | 9.141 | 5.68737E-14 | up |
| MELO3C033228.2 | 0 | 27 | 7.008 | 4.89853E-07 | up |
| MELO3C033230.2 | 790 | 131 | -2.589 | 5.33449E-72 | down |
| MELO3C033241.2 | 0 | 306 | 10.536 | 1.88863E-18 | up |
| MELO3C033242.2 | 32 | 0 | -7.609 | 6.99586E-09 | down |
| MELO3C033262.2 | 58 | 132 | 1.178 | 8.71754E-05 | up |
| MELO3C033291.2 | 0 | 186 | 9.816 | 8.67667E-16 | up |
| MELO3C033295.2 | 91 | 12 | -2.982 | 2.6022E-10 | down |
| MELO3C033300.2 | 338 | 74 | -2.194 | 6.0049E-13 | down |
| MELO3C033305.2 | 3333 | 148 | -4.491 | 2.04559E-14 | down |
| MELO3C033313.2 | 1 | 24 | 4.961 | 7.33803E-05 | up |
| MELO3C033314.2 | 12 | 0 | -6.139 | 9.23916E-05 | down |
| MELO3C033316.2 | 42 | 0 | -8.025 | 5.04583E-10 | down |
| MELO3C033317.2 | 60 | 1 | -6.656 | 1.68177E-09 | down |
| MELO3C033320.2 | 9 | 0 | -5.89 | 0.000281945 | down |
| MELO3C033346.2 | 0 | 164 | 9.635 | 1.31402E-15 | up |
| MELO3C033349.2 | 0 | 5 | 4.71 | 0.012159701 | up |
| MELO3C033351.2 | 0 | 31 | 7.232 | 5.14564E-08 | up |
| MELO3C033356.2 | 125 | 0 | -9.577 | 3.8085E-15 | down |
| MELO3C033363.2 | 73 | 35 | -1.059 | 0.007864693 | down |
| MELO3C033365.2 | 0 | 6 | 4.864 | 0.008709662 | up |
| MELO3C033370.2 | 1 | 320 | 8.154 | 1.72291E-21 | up |
| MELO3C033375.2 | 10 | 79 | 2.963 | 4.14948E-09 | up |
| MELO3C033377.2 | 0 | 6 | 4.851 | 0.006534529 | up |
| MELO3C033382.2 | 27 | 97 | 1.812 | 3.71332E-08 | up |
| MELO3C033388.2 | 32 | 83 | 1.38 | 6.88856E-05 | up |
| MELO3C033394.2 | 917 | 1 | -9.284 | 3.75927E-45 | down |
| MELO3C033396.2 | 0 | 6 | 4.74 | 0.010293733 | up |
| MELO3C033416.2 | 765 | 1 | -9.758 | 6.26729E-31 | down |
| MELO3C033427.2 | 17 | 5 | -1.769 | 0.018473434 | down |
| MELO3C033455.2 | 0 | 133 | 9.335 | 1.98787E-14 | up |
| MELO3C033461.2 | 0 | 223 | 10.081 | 4.95443E-17 | up |
| MELO3C033464.2 | 0 | 407 | 10.946 | 4.86769E-20 | up |
| MELO3C033472.2 | 0 | 7 | 4.091 | 0.013023344 | up |
| MELO3C033482.2 | 328 | 102 | -1.683 | 2.75054E-23 | down |
| MELO3C033491.2 | 0 | 11 | 5.772 | 0.000177397 | up |
| MELO3C033530.2 | 0 | 20 | 6.603 | 1.72028E-06 | up |
| MELO3C033536.2 | 66 | 284 | 2.115 | 1.87334E-18 | up |
| MELO3C033540.2 | 1 | 7 | 3.323 | 0.028568471 | up |
| MELO3C033542.2 | 7 | 21 | 1.654 | 0.011521464 | up |
| MELO3C033561.2 | 6 | 0 | -5.265 | 0.003316562 | down |
| MELO3C033564.2 | 0 | 15 | 6.127 | 2.49961E-05 | up |
| MELO3C033567.2 | 19 | 68 | 1.798 | 0.000115634 | up |
| MELO3C033578.2 | 70 | 1 | -5.906 | 1.86594E-12 | down |
| MELO3C033596.2 | 9 | 22 | 1.367 | 0.029188116 | up |
| MELO3C033603.2 | 1 | 261 | 7.449 | 3.20029E-23 | up |
| MELO3C033627.2 | 95 | 19 | -2.301 | 3.44123E-11 | down |
| MELO3C033665.2 | 70 | 23 | -1.599 | 8.35805E-06 | down |
| MELO3C033684.2 | 11 | 36 | 1.644 | 0.004052907 | up |
| MELO3C033689.2 | 0 | 51 | 7.952 | 5.34039E-10 | up |
| MELO3C033692.2 | 6 | 42 | 2.748 | 3.66557E-05 | up |
| MELO3C033697.2 | 4 | 37 | 3.336 | 6.11994E-07 | up |
| MELO3C033732.2 | 35 | 10 | -1.875 | 0.000395915 | down |
| MELO3C033764.2 | 93 | 0 | -9.159 | 1.06534E-13 | down |
| MELO3C033790.2 | 310 | 53 | -2.555 | 4.69316E-26 | down |
| MELO3C033802.2 | 10 | 37 | 1.859 | 0.001521267 | up |
| MELO3C033804.2 | 13 | 32 | 1.265 | 0.014939938 | up |
| MELO3C033830.2 | 116 | 314 | 1.433 | 4.32079E-14 | up |
| MELO3C033835.2 | 0 | 8 | 5.229 | 0.003283408 | up |
| MELO3C033882.2 | 37 | 1 | -4.981 | 2.94684E-07 | down |
| MELO3C033885.2 | 112 | 10 | -3.564 | 4.63321E-20 | down |
| MELO3C033886.2 | 0 | 43 | 7.701 | 1.9669E-09 | up |
| MELO3C033896.2 | 0 | 66 | 8.327 | 2.11109E-11 | up |
| MELO3C033902.2 | 124 | 51 | -1.289 | 3.61897E-07 | down |
| MELO3C033904.2 | 18 | 746 | 5.418 | 2.20543E-81 | up |
| MELO3C033914.2 | 173 | 391 | 1.181 | 1.09633E-13 | up |
| MELO3C033925.2 | 7 | 20 | 1.606 | 0.020683222 | up |
| MELO3C033933.2 | 9 | 27 | 1.591 | 0.00791104 | up |
| MELO3C033937.2 | 101 | 30 | -1.743 | 8.41157E-09 | down |
| MELO3C033939.2 | 48 | 168 | 1.815 | 6.94795E-12 | up |
| MELO3C033946.2 | 0 | 123 | 9.219 | 3.94029E-14 | up |
| MELO3C033947.2 | 0 | 48 | 7.875 | 5.37528E-10 | up |
| MELO3C033952.2 | 78 | 0 | -8.889 | 8.91312E-13 | down |
| MELO3C033954.2 | 5 | 390 | 6.175 | 1.78821E-09 | up |
| MELO3C033957.2 | 29 | 103 | 1.8 | 2.47533E-06 | up |
| MELO3C033959.2 | 0 | 5 | 4.465 | 0.023670182 | up |
| MELO3C033960.2 | 0 | 73 | 8.472 | 7.35106E-12 | up |
| MELO3C033966.2 | 46 | 4 | -3.704 | 2.38731E-09 | down |
| MELO3C033971.2 | 6 | 163 | 4.688 | 9.84715E-27 | up |
| MELO3C033988.2 | 1066 | 37 | -4.841 | 4.12787E-99 | down |
| MELO3C033989.2 | 24 | 3 | -3.053 | 0.000413188 | down |
| MELO3C033992.2 | 63 | 212 | 1.757 | 4.1397E-11 | up |
| MELO3C033999.2 | 7 | 0 | -5.359 | 0.002036327 | down |
| MELO3C034004.2 | 31 | 8 | -1.85 | 0.003850152 | down |
| MELO3C034009.2 | 196 | 631 | 1.682 | 6.27747E-29 | up |
| MELO3C034013.2 | 363 | 2 | -7.274 | 5.80416E-41 | down |
| MELO3C034022.2 | 12 | 0 | -5.252 | 0.000789802 | down |
| MELO3C034023.2 | 7970 | 2299 | -1.794 | 1.7035E-51 | down |
| MELO3C034027.2 | 112 | 43 | -1.387 | 0.000518696 | down |
| MELO3C034045.2 | 160 | 14 | -3.512 | 1.84283E-24 | down |
| MELO3C034046.2 | 32 | 93 | 1.565 | 5.1285E-06 | up |
| MELO3C034048.2 | 16 | 50 | 1.618 | 0.000560953 | up |
| MELO3C034084.2 | 0 | 147 | 9.478 | 5.11753E-15 | up |
| MELO3C034093.2 | 5 | 0 | -4.836 | 0.021510953 | down |
| MELO3C034100.2 | 111 | 17 | -2.677 | 1.74572E-13 | down |
| MELO3C034101.2 | 35 | 105 | 1.569 | 3.80973E-06 | up |
| MELO3C034116.2 | 387 | 142 | -1.446 | 2.37381E-12 | down |
| MELO3C034119.2 | 3667 | 1016 | -1.853 | 1.99554E-44 | down |
| MELO3C034128.2 | 240 | 74 | -1.688 | 1.32515E-13 | down |
| MELO3C034129.2 | 265 | 73 | -1.858 | 9.85039E-17 | down |
| MELO3C034152.2 | 43 | 6 | -2.92 | 1.32057E-06 | down |
| MELO3C034164.2 | 122 | 262 | 1.103 | 2.13248E-08 | up |
| MELO3C034167.2 | 42 | 101 | 1.255 | 8.41296E-06 | up |
| MELO3C034190.2 | 0 | 15 | 6.182 | 3.46597E-05 | up |
| MELO3C034211.2 | 24 | 0 | -7.188 | 2.27923E-07 | down |
| MELO3C034251.2 | 179 | 1242 | 2.793 | 2.046E-52 | up |
| MELO3C034276.2 | 0 | 126 | 9.259 | 2.32679E-14 | up |
| MELO3C034286.2 | 33 | 84 | 1.357 | 1.81949E-05 | up |
| MELO3C034288.2 | 84 | 1 | -7.158 | 3.62918E-11 | down |
| MELO3C034301.2 | 21 | 69 | 1.735 | 0.002958757 | up |
| MELO3C034302.2 | 1 | 18 | 4.538 | 0.000337969 | up |
| MELO3C034306.2 | 4 | 143 | 5.005 | 2.43954E-22 | up |
| MELO3C034317.2 | 23 | 5 | -2.151 | 0.014901904 | down |
| MELO3C034330.2 | 0 | 171 | 9.698 | 1.07564E-15 | up |
| MELO3C034342.2 | 0 | 500 | 11.244 | 4.0758E-21 | up |
| MELO3C034352.2 | 246 | 0 | -10.559 | 5.74363E-18 | down |
| MELO3C034353.2 | 174 | 0 | -10.062 | 2.4963E-16 | down |
| MELO3C034361.2 | 0 | 21 | 6.636 | 1.59001E-06 | up |
| MELO3C034362.2 | 0 | 298 | 10.499 | 1.46322E-18 | up |
| MELO3C034378.2 | 911 | 0 | -12.448 | 8.67496E-26 | down |
| MELO3C034380.2 | 0 | 44 | 7.729 | 3.62075E-09 | up |
| MELO3C034384.2 | 0 | 140 | 9.405 | 7.83126E-15 | up |
| MELO3C034386.2 | 0 | 164 | 9.632 | 1.42603E-15 | up |
| MELO3C034392.2 | 0 | 78 | 8.569 | 3.842E-12 | up |
| MELO3C034398.2 | 343 | 0 | -11.04 | 2.16054E-20 | down |
| MELO3C034399.2 | 14 | 0 | -6.41 | 1.53107E-05 | down |
| MELO3C034400.2 | 45 | 0 | -8.13 | 1.89231E-10 | down |
| MELO3C034410.2 | 33 | 6 | -2.39 | 0.000106642 | down |
| MELO3C034413.2 | 5 | 0 | -4.988 | 0.012629174 | down |
| MELO3C034414.2 | 0 | 162 | 9.619 | 2.8829E-15 | up |
| MELO3C034420.2 | 561 | 0 | -11.749 | 7.3864E-23 | down |
| MELO3C034437.2 | 1 | 26 | 4.131 | 2.33857E-05 | up |
| MELO3C034439.2 | 0 | 62 | 8.231 | 4.10728E-11 | up |
| MELO3C034442.2 | 4 | 25 | 2.748 | 0.000703912 | up |
| MELO3C034448.2 | 2 | 21 | 3.495 | 0.000160139 | up |
| MELO3C034452.2 | 290 | 593 | 1.034 | 2.85772E-12 | up |
| MELO3C034461.2 | 124 | 12 | -3.395 | 1.05284E-19 | down |
| MELO3C034465.2 | 37 | 12 | -1.623 | 0.002429219 | down |
| MELO3C034478.2 | 12 | 60 | 2.371 | 8.60559E-06 | up |
| MELO3C034484.2 | 6 | 0 | -5.264 | 0.016727215 | down |
| MELO3C034490.2 | 6 | 0 | -5.196 | 0.00373719 | down |
| MELO3C034500.2 | 5 | 17 | 1.676 | 0.021703043 | up |
| MELO3C034501.2 | 10 | 2 | -2.555 | 0.025736021 | down |
| MELO3C034520.2 | 44 | 296 | 2.756 | 2.83125E-24 | up |
| MELO3C034527.2 | 0 | 9 | 5.395 | 0.001199971 | up |
| MELO3C034541.2 | 194 | 1 | -8.363 | 2.45035E-15 | down |
| MELO3C034543.2 | 43 | 5 | -3.073 | 2.01364E-05 | down |
| MELO3C034544.2 | 11 | 1 | -4.185 | 0.003838679 | down |
| MELO3C034547.2 | 811 | 1 | -9.425 | 8.42447E-38 | down |
| MELO3C034559.2 | 133 | 44 | -1.582 | 9.22831E-08 | down |
| MELO3C034560.2 | 1296 | 0 | -12.956 | 1.17701E-27 | down |
| MELO3C034565.2 | 51 | 1 | -6.442 | 1.81922E-08 | down |
| MELO3C034581.2 | 3996 | 16 | -7.949 | 2.28983E-272 | down |
| MELO3C034589.2 | 0 | 14 | 6.039 | 4.11674E-05 | up |
| MELO3C034590.2 | 213 | 50 | -2.089 | 1.91337E-19 | down |
| MELO3C034613.2 | 31 | 403 | 3.701 | 1.41437E-57 | up |
| MELO3C034622.2 | 23 | 4 | -2.578 | 0.000235142 | down |
| MELO3C034646.2 | 45 | 99 | 1.112 | 0.000110131 | up |
| MELO3C034649.2 | 4 | 0 | -4.669 | 0.021552986 | down |
| MELO3C034651.2 | 30 | 0 | -7.51 | 4.17822E-08 | down |
| MELO3C034657.2 | 26 | 1 | -5.462 | 1.00301E-05 | down |
| MELO3C034663.2 | 6 | 27 | 2.299 | 0.000262033 | up |
| MELO3C034678.2 | 36 | 375 | 3.372 | 5.38088E-34 | up |
| MELO3C034695.2 | 108 | 7 | -4 | 3.66398E-21 | down |
| MELO3C034702.2 | 17 | 95 | 2.519 | 2.9205E-11 | up |
| MELO3C034703.2 | 37 | 3 | -3.474 | 1.55285E-06 | down |
| MELO3C034744.2 | 0 | 39 | 7.573 | 3.84346E-09 | up |
| MELO3C034763.2 | 557 | 1463 | 1.394 | 1.41435E-29 | up |
| MELO3C034767.2 | 61 | 0 | -8.555 | 1.29387E-11 | down |
| MELO3C034780.2 | 58 | 0 | -7.51 | 2.02905E-09 | down |
| MELO3C034781.2 | 157 | 2 | -6.47 | 1.05131E-23 | down |
| MELO3C034810.2 | 71 | 0 | -8.776 | 1.69971E-12 | down |
| MELO3C034811.2 | 123 | 0 | -9.561 | 3.57673E-15 | down |
| MELO3C034812.2 | 4 | 0 | -4.633 | 0.024070513 | down |
| MELO3C034813.2 | 239 | 578 | 1.275 | 1.50927E-08 | up |
| MELO3C034814.2 | 512 | 1360 | 1.409 | 9.79814E-18 | up |
| MELO3C034824.2 | 0 | 369 | 10.807 | 1.22577E-19 | up |
| MELO3C034862.2 | 5 | 53 | 3.346 | 2.55695E-07 | up |
| MELO3C034872.2 | 80 | 2 | -5.765 | 5.6684E-15 | down |
| MELO3C034877.2 | 57 | 0 | -8.435 | 2.12746E-11 | down |
| MELO3C034903.2 | 1 | 296 | 8.632 | 9.54017E-17 | up |
| MELO3C034906.2 | 365 | 2 | -7.951 | 4.29159E-32 | down |
| MELO3C034915.2 | 89 | 15 | -2.63 | 5.0524E-11 | down |
| MELO3C034917.2 | 0 | 61 | 8.204 | 5.84912E-11 | up |
| MELO3C034931.2 | 670 | 1339 | 1 | 1.28311E-21 | up |
| MELO3C034933.2 | 6 | 0 | -5.196 | 0.00373719 | down |
| MELO3C034941.2 | 6 | 32 | 2.41 | 0.000179167 | up |
| MELO3C034954.2 | 133 | 11 | -3.584 | 3.57023E-22 | down |
| MELO3C034955.2 | 0 | 305 | 10.527 | 1.97939E-18 | up |
| MELO3C034962.2 | 0 | 250 | 10.243 | 1.65487E-17 | up |
| MELO3C034964.2 | 0 | 18 | 6.466 | 9.41373E-06 | up |
| MELO3C034973.2 | 1501 | 568 | -1.403 | 1.3529E-42 | down |
| MELO3C034985.2 | 0 | 15 | 6.166 | 2.40799E-05 | up |
| MELO3C034995.2 | 368 | 71 | -2.387 | 6.36471E-31 | down |
| MELO3C035018.2 | 0 | 53 | 8.011 | 6.62744E-10 | up |
| MELO3C035023.2 | 107 | 398 | 1.894 | 2.8987E-23 | up |
| MELO3C035026.2 | 29 | 0 | -7.492 | 2.02265E-08 | down |
| MELO3C035031.2 | 8 | 124 | 3.916 | 5.11494E-20 | up |
| MELO3C035034.2 | 9 | 0 | -5.693 | 0.000748429 | down |
| MELO3C035044.2 | 336 | 164 | -1.038 | 2.14354E-07 | down |
| MELO3C035058.2 | 234 | 821 | 1.81 | 1.08334E-26 | up |
| MELO3C035066.2 | 92 | 490 | 2.411 | 3.95969E-34 | up |
| MELO3C035088.2 | 1 | 64 | 5.856 | 7.77604E-10 | up |
| MELO3C035091.2 | 0 | 155 | 9.553 | 2.48767E-15 | up |
| MELO3C035110.2 | 16 | 5 | -1.741 | 0.018941094 | down |
| MELO3C035114.2 | 0 | 6 | 3.979 | 0.019623342 | up |
| MELO3C035115.2 | 4 | 22 | 2.401 | 0.002798846 | up |
| MELO3C035119.2 | 0 | 102 | 8.958 | 2.33298E-13 | up |
| MELO3C035131.2 | 1 | 19 | 4.71 | 0.000157089 | up |
| MELO3C035133.2 | 107 | 33 | -1.72 | 1.48117E-07 | down |
| MELO3C035136.2 | 0 | 35 | 7.418 | 1.29999E-08 | up |
| MELO3C035139.2 | 267 | 95 | -1.49 | 1.14787E-13 | down |
| MELO3C035159.2 | 7 | 0 | -5.431 | 0.001667506 | down |
| MELO3C035177.2 | 1206 | 7148 | 2.568 | 4.4606E-51 | up |
| MELO3C035192.2 | 22 | 112 | 2.348 | 1.9963E-11 | up |
| MELO3C035199.2 | 124 | 607 | 2.289 | 4.06894E-31 | up |
| MELO3C035200.2 | 508 | 1 | -9.756 | 1.21162E-20 | down |
| MELO3C035201.2 | 25940 | 4 | -12.728 | 1.3028E-208 | down |
| MELO3C035224.2 | 1 | 8 | 2.843 | 0.03096262 | up |
| MELO3C035241.2 | 18 | 1 | -4.362 | 0.00027026 | down |
| MELO3C035246.2 | 2637 | 734 | -1.843 | 2.53968E-29 | down |
| MELO3C035250.2 | 117 | 35 | -1.725 | 2.37384E-08 | down |
| MELO3C035256.2 | 92 | 31 | -1.607 | 1.52181E-06 | down |
| MELO3C035261.2 | 3 | 14 | 2.215 | 0.020161342 | up |
| MELO3C035274.2 | 10943 | 2915 | -1.909 | 6.22211E-125 | down |
| MELO3C035281.2 | 35 | 4 | -3.323 | 2.23979E-07 | down |
| MELO3C035293.2 | 8624 | 3637 | -1.246 | 4.21082E-18 | down |
| MELO3C035299.2 | 1 | 20 | 4.743 | 0.000124301 | up |
| MELO3C035307.2 | 1 | 435 | 9.186 | 1.01345E-18 | up |
| MELO3C035318.2 | 125 | 22 | -2.493 | 1.61063E-14 | down |
| MELO3C035319.2 | 18 | 99 | 2.412 | 6.56748E-12 | up |
| MELO3C035321.2 | 54 | 22 | -1.304 | 0.000951597 | down |
| MELO3C035350.2 | 7 | 0 | -4.411 | 0.007953148 | down |
| MELO3C035367.2 | 68 | 0 | -8.697 | 3.47098E-12 | down |
| MELO3C035396.2 | 20 | 0 | -6.946 | 5.57391E-07 | down |
| MELO3C035414.2 | 0 | 1651 | 12.966 | 6.43166E-28 | up |
| MELO3C035435.2 | 2 | 195 | 6.225 | 2.79694E-25 | up |
| MELO3C035440.2 | 628 | 232 | -1.438 | 6.82349E-27 | down |
| MELO3C035452.2 | 16 | 0 | -6.608 | 6.51959E-06 | down |
| MELO3C035469.2 | 5 | 0 | -4.921 | 0.011713661 | down |
| MELO3C035515.2 | 287 | 34 | -3.057 | 4.3013E-33 | down |
| MELO3C035516.2 | 18 | 1 | -3.908 | 0.000647919 | down |
| MELO3C035517.2 | 34 | 3 | -3.298 | 4.47827E-07 | down |
| MELO3C035524.2 | 0 | 1024 | 12.278 | 3.88254E-25 | up |
| MELO3C035534.2 | 371 | 0 | -11.153 | 9.0658E-21 | down |
| MELO3C035535.2 | 810 | 3847 | 2.247 | 1.28764E-116 | up |
| MELO3C035538.2 | 7738 | 986 | -2.972 | 3.00456E-194 | down |
| MELO3C035544.2 | 166 | 342 | 1.045 | 1.87264E-06 | up |
| MELO3C035548.2 | 0 | 20 | 6.628 | 3.33956E-06 | up |
| MELO3C035553.2 | 902 | 367 | -1.296 | 3.69648E-12 | down |
| MELO3C035555.2 | 0 | 106 | 9.01 | 1.74351E-13 | up |
| MELO3C035567.2 | 84 | 2 | -5.574 | 3.48448E-16 | down |
| MELO3C035568.2 | 170 | 2 | -6.847 | 4.54725E-23 | down |
| MELO3C035574.2 | 60 | 154 | 1.377 | 1.45485E-08 | up |
| MELO3C035575.2 | 0 | 51 | 7.966 | 2.61993E-10 | up |
| MELO3C035576.2 | 0 | 6 | 4.867 | 0.007509754 | up |
| MELO3C035583.2 | 17 | 65 | 1.889 | 3.07882E-06 | up |
| MELO3C035595.2 | 8 | 1 | -3.149 | 0.0280224 | down |
| MELO3C035597.2 | 0 | 15 | 6.201 | 5.7583E-05 | up |
| MELO3C035600.2 | 0 | 14 | 6.115 | 3.32518E-05 | up |
| MELO3C035602.2 | 4 | 0 | -4.552 | 0.031645611 | down |
| MELO3C035604.2 | 4 | 30 | 2.76 | 0.00028155 | up |
| MELO3C035606.2 | 0 | 69 | 8.381 | 1.74694E-11 | up |
| MELO3C035611.2 | 15 | 50 | 1.7 | 0.000102142 | up |
| MELO3C035622.2 | 46 | 0 | -8.149 | 2.80519E-10 | down |
| MELO3C035627.2 | 889 | 24 | -5.233 | 4.02142E-133 | down |
| MELO3C035646.2 | 36 | 0 | -7.776 | 2.6929E-09 | down |
| MELO3C035647.2 | 7 | 0 | -5.423 | 0.002112508 | down |
| MELO3C035649.2 | 0 | 464 | 11.134 | 8.91456E-21 | up |
| MELO3C035650.2 | 27 | 0 | -7.362 | 7.39976E-08 | down |
| MELO3C035652.2 | 0 | 5 | 4.541 | 0.025010664 | up |
| MELO3C035654.2 | 0 | 31 | 7.232 | 5.03441E-08 | up |
| MELO3C035656.2 | 11 | 28 | 1.313 | 0.028016146 | up |
| MELO3C035662.2 | 35 | 90 | 1.338 | 3.49256E-05 | up |
| MELO3C035669.2 | 18 | 40 | 1.205 | 0.024329175 | up |
| MELO3C035676.2 | 52 | 125 | 1.262 | 3.46289E-06 | up |
| MELO3C035679.2 | 141 | 42 | -1.723 | 8.36688E-10 | down |
| MELO3C035682.2 | 10 | 87 | 3.096 | 1.02189E-08 | up |
| MELO3C035683.2 | 0 | 119 | 9.177 | 4.72183E-14 | up |
| MELO3C035685.2 | 0 | 17 | 6.371 | 1.77426E-05 | up |
| MELO3C035692.2 | 4 | 19 | 2.119 | 0.012796739 | up |
| MELO3C035696.2 | 2 | 31 | 3.586 | 1.23981E-05 | up |
| MELO3C035704.2 | 238 | 0 | -10.512 | 1.76809E-18 | down |
| MELO3C035729.2 | 19 | 249 | 3.734 | 1.42523E-21 | up |
| MELO3C035742.2 | 0 | 6 | 3.867 | 0.024697039 | up |
| MELO3C035750.2 | 17 | 1 | -3.884 | 0.000122022 | down |
| MELO3C035751.2 | 56 | 128 | 1.203 | 4.65383E-06 | up |
| MELO3C035753.2 | 54 | 150 | 1.47 | 1.34364E-08 | up |
| MELO3C035759.2 | 43 | 15 | -1.591 | 0.002415481 | down |
| MELO3C035765.2 | 28 | 58 | 1.056 | 0.009971445 | up |
| MELO3C035769.2 | 18 | 5 | -1.837 | 0.011094599 | down |
| MELO3C035770.2 | 158 | 0 | -9.924 | 1.82612E-15 | down |
| MELO3C035771.2 | 0 | 10197 | 15.594 | 7.61674E-40 | up |
| MELO3C035775.2 | 5 | 0 | -5.014 | 0.008747164 | down |
| MELO3C035776.2 | 0 | 7 | 5.062 | 0.005087803 | up |
