# Supplementary material for: Comprehensive Analysis of Transcriptome and Metabolome Reveals the Flavonoid Metabolic Pathway Is Associated with Fruit Peel Coloration of Melon
Source: Molecules. 2021 May 10;26(9):2830. doi: 10.3390/molecules26092830 (PMC8126211; doi:10.3390/molecules26092830)
Supplement: Supplementary file 1 [file molecules-26-02830-s001.zip › molecules-1183709-supplementary/Table S5 GO annotation of differentially expressed genes in W vs B.docx]

| **Table S5 GO annotation of differentially expressed genes in W vs B** | | | | | | | |
| --- | --- | --- | --- | --- | --- | --- | --- |
| **Ontology** | **ID** | **Description** | **pvalue** | **Count** | **up** | **down** |  |
| Molecular function | GO:0008168 | methyltransferase activity | 0.0003 | 7 | 3 | 4 |  |
| Molecular function | GO:0016741 | transferase activity, transferring one-carbon groups | 0.0003 | 7 | 3 | 4 |  |
| Molecular function | GO:0008171 | O-methyltransferase activity | 0.0007 | 4 | 2 | 2 |  |
| Molecular function | GO:0051537 | 2 iron, 2 sulfur cluster binding | 0.0025 | 4 | 1 | 3 |  |
| Molecular function | GO:0016491 | oxidoreductase activity | 0.0033 | 26 | 15 | 11 |  |
| Molecular function | GO:0016705 | oxidoreductase activity, acting on paired donors, with incorporation or reduction of molecular oxygen | 0.0041 | 11 | 6 | 5 |  |
| Molecular function | GO:0008757 | S-adenosylmethionine-dependent methyltransferase activity | 0.0051 | 4 | 1 | 3 |  |
| Molecular function | GO:0051213 | dioxygenase activity | 0.0090 | 4 | 2 | 2 |  |
| Molecular function | GO:0046983 | protein dimerization activity | 0.0108 | 11 | 7 | 4 |  |
| Molecular function | GO:0004497 | monooxygenase activity | 0.0112 | 10 | 7 | 3 |  |
| Molecular function | GO:0016701 | oxidoreductase activity, acting on single donors with incorporation of molecular oxygen | 0.0138 | 3 | 3 | 0 |  |
| Molecular function | GO:0016874 | ligase activity | 0.0193 | 5 | 3 | 2 |  |
| Molecular function | GO:0005506 | iron ion binding | 0.0211 | 9 | 6 | 3 |  |
| Molecular function | GO:0015035 | protein disulfide oxidoreductase activity | 0.0220 | 4 | 0 | 4 |  |
| Molecular function | GO:0020037 | heme binding | 0.0222 | 10 | 7 | 3 |  |
| Molecular function | GO:0048037 | cofactor binding | 0.0313 | 18 | 10 | 8 |  |
| Molecular function | GO:0015036 | disulfide oxidoreductase activity | 0.0348 | 4 | 0 | 4 |  |
| Molecular function | GO:0016667 | oxidoreductase activity, acting on a sulfur group of donors | 0.0559 | 4 | 0 | 4 |  |
| Molecular function | GO:0016702 | oxidoreductase activity, acting on single donors with incorporation of molecular oxygen, incorporation of two atoms of oxygen | 0.0594 | 2 | 2 | 0 |  |
| Molecular function | GO:0140101 | catalytic activity, acting on a tRNA | 0.0594 | 2 | 1 | 1 |  |
| Molecular function | GO:0051536 | iron-sulfur cluster binding | 0.0659 | 4 | 1 | 3 |  |
| Molecular function | GO:0051540 | metal cluster binding | 0.0659 | 4 | 1 | 3 |  |
| Biological process | GO:0065008 | regulation of biological quality | 0.0019 | 17 | 6 | 11 |  |
| Biological process | GO:0071407 | cellular response to organic cyclic compound | 0.0036 | 5 | 4 | 1 |  |
| Biological process | GO:0045934 | negative regulation of nucleobase-containing compound metabolic process | 0.0042 | 5 | 4 | 1 |  |
| Biological process | GO:0042446 | hormone biosynthetic process | 0.0046 | 6 | 1 | 5 |  |
| Biological process | GO:0045454 | cell redox homeostasis | 0.0081 | 5 | 1 | 4 |  |
| Biological process | GO:0009739 | response to gibberellin | 0.0091 | 5 | 5 | 0 |  |
| Biological process | GO:0014070 | response to organic cyclic compound | 0.0120 | 7 | 6 | 1 |  |
| Biological process | GO:2000113 | negative regulation of cellular macromolecule biosynthetic process | 0.0126 | 5 | 4 | 1 |  |
| Biological process | GO:0016053 | organic acid biosynthetic process | 0.0126 | 9 | 5 | 4 |  |
| Biological process | GO:0046394 | carboxylic acid biosynthetic process | 0.0126 | 9 | 5 | 4 |  |
| Biological process | GO:0008610 | lipid biosynthetic process | 0.0135 | 8 | 5 | 3 |  |
| Biological process | GO:0010558 | negative regulation of macromolecule biosynthetic process | 0.0140 | 5 | 4 | 1 |  |
| Biological process | GO:0009741 | response to brassinosteroid | 0.0141 | 4 | 4 | 0 |  |
| Biological process | GO:0042592 | homeostatic process | 0.0142 | 9 | 3 | 6 |  |
| Biological process | GO:0016125 | sterol metabolic process | 0.0145 | 3 | 2 | 1 |  |
| Biological process | GO:0006631 | fatty acid metabolic process | 0.0154 | 5 | 4 | 1 |  |
| Biological process | GO:0044283 | small molecule biosynthetic process | 0.0174 | 10 | 5 | 5 |  |
| Biological process | GO:0045892 | negative regulation of transcription, DNA-templated | 0.0181 | 4 | 3 | 1 |  |
| Biological process | GO:0031327 | negative regulation of cellular biosynthetic process | 0.0186 | 5 | 4 | 1 |  |
| Biological process | GO:0019752 | carboxylic acid metabolic process | 0.0198 | 12 | 7 | 5 |  |
| Biological process | GO:0051253 | negative regulation of RNA metabolic process | 0.0203 | 4 | 3 | 1 |  |
| Biological process | GO:1902679 | negative regulation of RNA biosynthetic process | 0.0203 | 4 | 3 | 1 |  |
| Biological process | GO:1903507 | negative regulation of nucleic acid-templated transcription | 0.0203 | 4 | 3 | 1 |  |
| Biological process | GO:0009890 | negative regulation of biosynthetic process | 0.0222 | 5 | 4 | 1 |  |
| Biological process | GO:0042445 | hormone metabolic process | 0.0247 | 6 | 1 | 5 |  |
| Biological process | GO:0044255 | cellular lipid metabolic process | 0.0251 | 9 | 6 | 3 |  |
| Biological process | GO:0032787 | monocarboxylic acid metabolic process | 0.0269 | 8 | 6 | 2 |  |
| Biological process | GO:0048609 | multicellular organismal reproductive process | 0.0279 | 4 | 3 | 1 |  |
| Biological process | GO:0009742 | brassinosteroid mediated signaling pathway | 0.0279 | 3 | 3 | 0 |  |
| Biological process | GO:0043401 | steroid hormone mediated signaling pathway | 0.0279 | 3 | 3 | 0 |  |
| Biological process | GO:0048545 | response to steroid hormone | 0.0279 | 3 | 3 | 0 |  |
| Biological process | GO:0071367 | cellular response to brassinosteroid stimulus | 0.0279 | 3 | 3 | 0 |  |
| Biological process | GO:0071383 | cellular response to steroid hormone stimulus | 0.0279 | 3 | 3 | 0 |  |
| Biological process | GO:0006082 | organic acid metabolic process | 0.0298 | 13 | 8 | 5 |  |
| Biological process | GO:0043436 | oxoacid metabolic process | 0.0298 | 13 | 8 | 5 |  |
| Biological process | GO:0008202 | steroid metabolic process | 0.0320 | 3 | 2 | 1 |  |
| Biological process | GO:0009835 | fruit ripening | 0.0320 | 3 | 1 | 2 |  |
