# Supplementary material for: Comprehensive Analysis of Transcriptome and Metabolome Reveals the Flavonoid Metabolic Pathway Is Associated with Fruit Peel Coloration of Melon
Source: Molecules. 2021 May 10;26(9):2830. doi: 10.3390/molecules26092830 (PMC8126211; doi:10.3390/molecules26092830)
Supplement: Supplementary file 1 [file molecules-26-02830-s001.zip › molecules-1183709-supplementary/Table S6 GO annotation of differentially expressed genes in W vs H.docx]

| **Table S6 GO annotation of differentially expressed genes in W vs H** | | | | | | | |
| --- | --- | --- | --- | --- | --- | --- | --- |
| **Ontology** | **ID** | **Description** | **pvalue** | **Count** | **up** | **down** |  |
| Cellular component | GO:0009523 | photosystem II | 0.0001 | 22 | 0 | 22 |  |
| Cellular component | GO:0009521 | photosystem | 0.0001 | 29 | 1 | 28 |  |
| Cellular component | GO:0009522 | photosystem I | 0.0003 | 18 | 1 | 17 |  |
| Cellular component | GO:0009579 | thylakoid | 0.0004 | 58 | 9 | 49 |  |
| Cellular component | GO:0034357 | photosynthetic membrane | 0.0010 | 51 | 4 | 47 |  |
| Cellular component | GO:0042651 | thylakoid membrane | 0.0010 | 51 | 4 | 47 |  |
| Cellular component | GO:0009534 | chloroplast thylakoid | 0.0010 | 54 | 5 | 49 |  |
| Cellular component | GO:0031976 | plastid thylakoid | 0.0010 | 54 | 5 | 49 |  |
| Cellular component | GO:0009535 | chloroplast thylakoid membrane | 0.0012 | 50 | 4 | 46 |  |
| Cellular component | GO:0055035 | plastid thylakoid membrane | 0.0012 | 50 | 4 | 46 |  |
| Cellular component | GO:0044436 | thylakoid part | 0.0014 | 52 | 5 | 47 |  |
| Cellular component | GO:0005777 | peroxisome | 0.0016 | 15 | 3 | 12 |  |
| Cellular component | GO:0042579 | microbody | 0.0016 | 15 | 3 | 12 |  |
| Cellular component | GO:0000786 | nucleosome | 0.0114 | 9 | 9 | 0 |  |
| Cellular component | GO:0032993 | protein-DNA complex | 0.0114 | 9 | 9 | 0 |  |
| Cellular component | GO:0044815 | DNA packaging complex | 0.0114 | 9 | 9 | 0 |  |
| Cellular component | GO:0044435 | plastid part | 0.0135 | 88 | 11 | 77 |  |
| Cellular component | GO:0044434 | chloroplast part | 0.0151 | 87 | 11 | 76 |  |
| Cellular component | GO:0031984 | organelle subcompartment | 0.0204 | 114 | 37 | 77 |  |
| Cellular component | GO:0000139 | Golgi membrane | 0.0578 | 35 | 26 | 9 |  |
| Cellular component | GO:0010287 | plastoglobule | 0.0585 | 6 | 3 | 3 |  |
| Molecular function | GO:0016705 | oxidoreductase activity, acting on paired donors, with incorporation or reduction of molecular oxygen | 0.0012 | 51 | 18 | 33 |  |
| Molecular function | GO:0016701 | oxidoreductase activity, acting on single donors with incorporation of molecular oxygen | 0.0013 | 10 | 2 | 8 |  |
| Molecular function | GO:0004497 | monooxygenase activity | 0.0018 | 50 | 17 | 33 |  |
| Molecular function | GO:0016491 | oxidoreductase activity | 0.0019 | 147 | 52 | 95 |  |
| Molecular function | GO:0008194 | UDP-glycosyltransferase activity | 0.0020 | 44 | 15 | 29 |  |
| Molecular function | GO:0035251 | UDP-glucosyltransferase activity | 0.0023 | 35 | 10 | 25 |  |
| Molecular function | GO:0048037 | cofactor binding | 0.0027 | 113 | 29 | 84 |  |
| Molecular function | GO:0016702 | oxidoreductase activity, acting on single donors with incorporation of molecular oxygen, incorporation of two atoms of oxygen | 0.0029 | 8 | 1 | 7 |  |
| Molecular function | GO:0016758 | transferase activity, transferring hexosyl groups | 0.0054 | 55 | 21 | 34 |  |
| Molecular function | GO:0051213 | dioxygenase activity | 0.0054 | 13 | 4 | 9 |  |
| Molecular function | GO:0046906 | tetrapyrrole binding | 0.0062 | 67 | 15 | 52 |  |
| Molecular function | GO:0051537 | 2 iron, 2 sulfur cluster binding | 0.0071 | 10 | 1 | 9 |  |
| Molecular function | GO:0046527 | glucosyltransferase activity | 0.0072 | 35 | 10 | 25 |  |
| Molecular function | GO:0004175 | endopeptidase activity | 0.0088 | 30 | 17 | 13 |  |
| Molecular function | GO:0070011 | peptidase activity, acting on L-amino acid peptides | 0.0107 | 34 | 20 | 14 |  |
| Molecular function | GO:0016757 | transferase activity, transferring glycosyl groups | 0.0131 | 69 | 29 | 40 |  |
| Molecular function | GO:0008233 | peptidase activity | 0.0136 | 34 | 20 | 14 |  |
| Molecular function | GO:0004672 | protein kinase activity | 0.0148 | 109 | 54 | 55 |  |
| Molecular function | GO:0016860 | intramolecular oxidoreductase activity | 0.0162 | 8 | 1 | 7 |  |
| Molecular function | GO:0016835 | carbon-oxygen lyase activity | 0.0172 | 15 | 7 | 8 |  |
| Molecular function | GO:0043531 | ADP binding | 0.0190 | 19 | 12 | 7 |  |
| Molecular function | GO:0005509 | calcium ion binding | 0.0220 | 29 | 11 | 18 |  |
| Molecular function | GO:0001134 | transcription regulator recruiting activity | 0.0231 | 10 | 4 | 6 |  |
| Molecular function | GO:0001135 | RNA polymerase II transcription regulator recruiting activity | 0.0231 | 10 | 4 | 6 |  |
| Molecular function | GO:0004674 | protein serine/threonine kinase activity | 0.0241 | 98 | 47 | 51 |  |
| Molecular function | GO:0016773 | phosphotransferase activity, alcohol group as acceptor | 0.0252 | 115 | 54 | 61 |  |
| Molecular function | GO:0005506 | iron ion binding | 0.0255 | 43 | 14 | 29 |  |
| Molecular function | GO:0030246 | carbohydrate binding | 0.0284 | 49 | 26 | 23 |  |
| Molecular function | GO:0016301 | kinase activity | 0.0286 | 117 | 55 | 62 |  |
| Molecular function | GO:0004190 | aspartic-type endopeptidase activity | 0.0294 | 14 | 8 | 6 |  |
| Molecular function | GO:0070001 | aspartic-type peptidase activity | 0.0294 | 14 | 8 | 6 |  |
| Molecular function | GO:0016836 | hydro-lyase activity | 0.0302 | 8 | 4 | 4 |  |
| Molecular function | GO:0008236 | serine-type peptidase activity | 0.0404 | 12 | 8 | 4 |  |
| Molecular function | GO:0017171 | serine hydrolase activity | 0.0404 | 12 | 8 | 4 |  |
| Molecular function | GO:0016168 | chlorophyll binding | 0.0405 | 18 | 1 | 17 |  |
| Molecular function | GO:0020037 | heme binding | 0.0504 | 48 | 14 | 34 |  |
| Molecular function | GO:0043621 | protein self-association | 0.0507 | 8 | 3 | 5 |  |
| Molecular function | GO:0016874 | ligase activity | 0.0530 | 18 | 7 | 11 |  |
| Molecular function | GO:0016709 | oxidoreductase activity, acting on paired donors, with incorporation or reduction of molecular oxygen, NAD(P)H as one donor, and incorporation of one atom of oxygen | 0.0558 | 24 | 8 | 16 |  |
| Molecular function | GO:0004252 | serine-type endopeptidase activity | 0.0560 | 10 | 7 | 3 |  |
| Molecular function | GO:0022838 | substrate-specific channel activity | 0.0560 | 10 | 5 | 5 |  |
| Molecular function | GO:0015267 | channel activity | 0.0575 | 12 | 7 | 5 |  |
| Molecular function | GO:0022803 | passive transmembrane transporter activity | 0.0575 | 12 | 7 | 5 |  |
| Molecular function | GO:0016747 | transferase activity, transferring acyl groups other than amino-acyl groups | 0.0630 | 33 | 12 | 21 |  |
| Molecular function | GO:0004869 | cysteine-type endopeptidase inhibitor activity | 0.0662 | 9 | 1 | 8 |  |
| Molecular function | GO:0015035 | protein disulfide oxidoreductase activity | 0.0670 | 13 | 2 | 11 |  |
| Molecular function | GO:0016746 | transferase activity, transferring acyl groups | 0.0683 | 35 | 12 | 23 |  |
| Molecular function | GO:0004713 | protein tyrosine kinase activity | 0.0693 | 6 | 5 | 1 |  |
| Molecular function | GO:0016838 | carbon-oxygen lyase activity, acting on phosphates | 0.0693 | 6 | 2 | 4 |  |
| Molecular function | GO:0019842 | vitamin binding | 0.0758 | 14 | 4 | 10 |  |
| Molecular function | GO:0004866 | endopeptidase inhibitor activity | 0.0889 | 13 | 4 | 9 |  |
| Molecular function | GO:0008134 | transcription factor binding | 0.0889 | 13 | 6 | 7 |  |
| Molecular function | GO:0030414 | peptidase inhibitor activity | 0.0889 | 13 | 4 | 9 |  |
| Molecular function | GO:0061134 | peptidase regulator activity | 0.0889 | 13 | 4 | 9 |  |
| Molecular function | GO:0061135 | endopeptidase regulator activity | 0.0889 | 13 | 4 | 9 |  |
| Molecular function | GO:0016667 | oxidoreductase activity, acting on a sulfur group of donors | 0.0918 | 16 | 3 | 13 |  |
| Biological process | GO:0009765 | photosynthesis, light harvesting | 0.0000 | 13 | 0 | 13 |  |
| Biological process | GO:0019752 | carboxylic acid metabolic process | 0.0000 | 81 | 24 | 57 |  |
| Biological process | GO:0015979 | photosynthesis | 0.0001 | 47 | 2 | 45 |  |
| Biological process | GO:0031407 | oxylipin metabolic process | 0.0001 | 10 | 4 | 6 |  |
| Biological process | GO:0031408 | oxylipin biosynthetic process | 0.0001 | 10 | 4 | 6 |  |
| Biological process | GO:0010035 | response to inorganic substance | 0.0001 | 79 | 31 | 48 |  |
| Biological process | GO:0006082 | organic acid metabolic process | 0.0001 | 90 | 26 | 64 |  |
| Biological process | GO:0043436 | oxoacid metabolic process | 0.0001 | 90 | 26 | 64 |  |
| Biological process | GO:0006629 | lipid metabolic process | 0.0001 | 69 | 23 | 46 |  |
| Biological process | GO:0016053 | organic acid biosynthetic process | 0.0001 | 52 | 15 | 37 |  |
| Biological process | GO:0046394 | carboxylic acid biosynthetic process | 0.0001 | 52 | 15 | 37 |  |
| Biological process | GO:0009835 | fruit ripening | 0.0005 | 14 | 5 | 9 |  |
| Biological process | GO:0006631 | fatty acid metabolic process | 0.0005 | 24 | 7 | 17 |  |
| Biological process | GO:0032787 | monocarboxylic acid metabolic process | 0.0006 | 48 | 15 | 33 |  |
| Biological process | GO:0044255 | cellular lipid metabolic process | 0.0011 | 54 | 19 | 35 |  |
| Biological process | GO:0065008 | regulation of biological quality | 0.0011 | 92 | 41 | 51 |  |
| Biological process | GO:0072330 | monocarboxylic acid biosynthetic process | 0.0014 | 34 | 10 | 24 |  |
| Biological process | GO:0044283 | small molecule biosynthetic process | 0.0015 | 58 | 17 | 41 |  |
| Biological process | GO:0009414 | response to water deprivation | 0.0018 | 43 | 19 | 24 |  |
| Biological process | GO:0009642 | response to light intensity | 0.0019 | 14 | 1 | 13 |  |
| Biological process | GO:0044281 | small molecule metabolic process | 0.0023 | 121 | 39 | 82 |  |
| Biological process | GO:0009415 | response to water | 0.0024 | 43 | 19 | 24 |  |
| Biological process | GO:0006778 | porphyrin-containing compound metabolic process | 0.0025 | 9 | 1 | 8 |  |
| Biological process | GO:0033013 | tetrapyrrole metabolic process | 0.0025 | 9 | 1 | 8 |  |
| Biological process | GO:0009741 | response to brassinosteroid | 0.0026 | 16 | 8 | 8 |  |
| Biological process | GO:0019684 | photosynthesis, light reaction | 0.0032 | 23 | 0 | 23 |  |
| Biological process | GO:0008610 | lipid biosynthetic process | 0.0033 | 41 | 14 | 27 |  |
| Biological process | GO:0040007 | growth | 0.0036 | 71 | 39 | 32 |  |
| Biological process | GO:0008299 | isoprenoid biosynthetic process | 0.0045 | 21 | 7 | 14 |  |
| Biological process | GO:0042446 | hormone biosynthetic process | 0.0047 | 23 | 9 | 14 |  |
| Biological process | GO:0046165 | alcohol biosynthetic process | 0.0049 | 11 | 4 | 7 |  |
| Biological process | GO:0006714 | sesquiterpenoid metabolic process | 0.0058 | 9 | 2 | 7 |  |
| Biological process | GO:0009644 | response to high light intensity | 0.0058 | 9 | 0 | 9 |  |
| Biological process | GO:0009687 | abscisic acid metabolic process | 0.0058 | 9 | 2 | 7 |  |
| Biological process | GO:0043288 | apocarotenoid metabolic process | 0.0058 | 9 | 2 | 7 |  |
| Biological process | GO:1902644 | tertiary alcohol metabolic process | 0.0058 | 9 | 2 | 7 |  |
| Biological process | GO:0006066 | alcohol metabolic process | 0.0059 | 14 | 4 | 10 |  |
| Biological process | GO:0006779 | porphyrin-containing compound biosynthetic process | 0.0060 | 8 | 1 | 7 |  |
| Biological process | GO:0015994 | chlorophyll metabolic process | 0.0060 | 8 | 1 | 7 |  |
| Biological process | GO:0015995 | chlorophyll biosynthetic process | 0.0060 | 8 | 1 | 7 |  |
| Biological process | GO:0033014 | tetrapyrrole biosynthetic process | 0.0060 | 8 | 1 | 7 |  |
| Biological process | GO:0046148 | pigment biosynthetic process | 0.0063 | 19 | 8 | 11 |  |
| Biological process | GO:1901615 | organic hydroxy compound metabolic process | 0.0063 | 27 | 9 | 18 |  |
| Biological process | GO:0042445 | hormone metabolic process | 0.0068 | 30 | 11 | 19 |  |
| Biological process | GO:0040008 | regulation of growth | 0.0073 | 39 | 21 | 18 |  |
| Biological process | GO:0016114 | terpenoid biosynthetic process | 0.0075 | 18 | 5 | 13 |  |
| Biological process | GO:0048868 | pollen tube development | 0.0075 | 18 | 12 | 6 |  |
| Biological process | GO:0006720 | isoprenoid metabolic process | 0.0078 | 22 | 7 | 15 |  |
| Biological process | GO:0051273 | beta-glucan metabolic process | 0.0091 | 11 | 6 | 5 |  |
| Biological process | GO:0042440 | pigment metabolic process | 0.0092 | 19 | 8 | 11 |  |
| Biological process | GO:0030243 | cellulose metabolic process | 0.0104 | 10 | 5 | 5 |  |
| Biological process | GO:0051274 | beta-glucan biosynthetic process | 0.0104 | 10 | 5 | 5 |  |
| Biological process | GO:0014070 | response to organic cyclic compound | 0.0108 | 32 | 14 | 18 |  |
| Biological process | GO:0008283 | cell proliferation | 0.0117 | 9 | 6 | 3 |  |
| Biological process | GO:0030244 | cellulose biosynthetic process | 0.0117 | 9 | 4 | 5 |  |
| Biological process | GO:0042542 | response to hydrogen peroxide | 0.0117 | 9 | 2 | 7 |  |
| Biological process | GO:0048509 | regulation of meristem development | 0.0130 | 8 | 4 | 4 |  |
| Biological process | GO:0006721 | terpenoid metabolic process | 0.0131 | 19 | 5 | 14 |  |
| Biological process | GO:0019216 | regulation of lipid metabolic process | 0.0141 | 7 | 4 | 3 |  |
| Biological process | GO:0043455 | regulation of secondary metabolic process | 0.0141 | 7 | 3 | 4 |  |
| Biological process | GO:1901617 | organic hydroxy compound biosynthetic process | 0.0156 | 18 | 7 | 11 |  |
| Biological process | GO:0048589 | developmental growth | 0.0179 | 40 | 22 | 18 |  |
| Biological process | GO:0050793 | regulation of developmental process | 0.0184 | 55 | 25 | 30 |  |
| Biological process | GO:0006633 | fatty acid biosynthetic process | 0.0184 | 15 | 5 | 10 |  |
| Biological process | GO:0006952 | defense response | 0.0211 | 124 | 48 | 76 |  |
| Biological process | GO:0044282 | small molecule catabolic process | 0.0216 | 18 | 3 | 15 |  |
| Biological process | GO:1901605 | alpha-amino acid metabolic process | 0.0221 | 26 | 5 | 21 |  |
| Biological process | GO:0016054 | organic acid catabolic process | 0.0257 | 17 | 3 | 14 |  |
| Biological process | GO:0046395 | carboxylic acid catabolic process | 0.0257 | 17 | 3 | 14 |  |
| Biological process | GO:0009860 | pollen tube growth | 0.0263 | 15 | 11 | 4 |  |
| Biological process | GO:0016051 | carbohydrate biosynthetic process | 0.0264 | 32 | 17 | 15 |  |
| Biological process | GO:0010817 | regulation of hormone levels | 0.0270 | 41 | 19 | 22 |  |
| Biological process | GO:0048869 | cellular developmental process | 0.0271 | 67 | 39 | 28 |  |
| Biological process | GO:0010038 | response to metal ion | 0.0274 | 29 | 10 | 19 |  |
| Biological process | GO:0005976 | polysaccharide metabolic process | 0.0276 | 37 | 24 | 13 |  |
| Biological process | GO:0033692 | cellular polysaccharide biosynthetic process | 0.0292 | 18 | 12 | 6 |  |
| Biological process | GO:0007568 | aging | 0.0304 | 21 | 6 | 15 |  |
| Biological process | GO:0034637 | cellular carbohydrate biosynthetic process | 0.0304 | 21 | 12 | 9 |  |
| Biological process | GO:0048638 | regulation of developmental growth | 0.0313 | 12 | 7 | 5 |  |
| Biological process | GO:0009813 | flavonoid biosynthetic process | 0.0314 | 14 | 9 | 5 |  |
| Biological process | GO:0030154 | cell differentiation | 0.0326 | 54 | 31 | 23 |  |
| Biological process | GO:0000271 | polysaccharide biosynthetic process | 0.0332 | 22 | 15 | 7 |  |
| Biological process | GO:0048588 | developmental cell growth | 0.0359 | 20 | 12 | 8 |  |
| Biological process | GO:0050832 | defense response to fungus | 0.0372 | 27 | 14 | 13 |  |
| Biological process | GO:0080167 | response to karrikin | 0.0376 | 13 | 7 | 6 |  |
| Biological process | GO:1901607 | alpha-amino acid biosynthetic process | 0.0376 | 13 | 3 | 10 |  |
| Biological process | GO:0016049 | cell growth | 0.0406 | 38 | 22 | 16 |  |
| Biological process | GO:0009856 | pollination | 0.0413 | 29 | 18 | 11 |  |
| Biological process | GO:0044706 | multi-multicellular organism process | 0.0413 | 29 | 18 | 11 |  |
| Biological process | GO:0009812 | flavonoid metabolic process | 0.0436 | 14 | 9 | 5 |  |
| Biological process | GO:0000902 | cell morphogenesis | 0.0450 | 31 | 21 | 10 |  |
| Biological process | GO:0009742 | brassinosteroid mediated signaling pathway | 0.0451 | 10 | 6 | 4 |  |
| Biological process | GO:0043401 | steroid hormone mediated signaling pathway | 0.0451 | 10 | 6 | 4 |  |
| Biological process | GO:0048545 | response to steroid hormone | 0.0451 | 10 | 6 | 4 |  |
| Biological process | GO:0071367 | cellular response to brassinosteroid stimulus | 0.0451 | 10 | 6 | 4 |  |
| Biological process | GO:0071383 | cellular response to steroid hormone stimulus | 0.0451 | 10 | 6 | 4 |  |
| Biological process | GO:0009250 | glucan biosynthetic process | 0.0451 | 12 | 7 | 5 |  |
| Biological process | GO:0051188 | cofactor biosynthetic process | 0.0459 | 17 | 6 | 11 |  |
| Biological process | GO:0009932 | cell tip growth | 0.0493 | 15 | 11 | 4 |  |
| Biological process | GO:0043086 | negative regulation of catalytic activity | 0.0499 | 7 | 0 | 7 |  |
| Biological process | GO:0000302 | response to reactive oxygen species | 0.0522 | 13 | 4 | 9 |  |
| Biological process | GO:0008652 | cellular amino acid biosynthetic process | 0.0522 | 13 | 3 | 10 |  |
