# Supplementary material for: Comprehensive Analysis of Transcriptome and Metabolome Reveals the Flavonoid Metabolic Pathway Is Associated with Fruit Peel Coloration of Melon
Source: Molecules. 2021 May 10;26(9):2830. doi: 10.3390/molecules26092830 (PMC8126211; doi:10.3390/molecules26092830)
Supplement: Supplementary file 1 [file molecules-26-02830-s001.zip › molecules-1183709-supplementary/Table S7 GO annotation of differentially expressed genes in B vs H.docx]

| **Table S7 GO annotation of differentially expressed genes in B vs H** | | | | | | | |
| --- | --- | --- | --- | --- | --- | --- | --- |
| **Ontology** | **ID** | **Description** | **pvalue** | **Count** | **up** | **down** |  |
| Cellular component | GO:0009579 | thylakoid | 0.0000 | 64 | 8 | 56 |  |
| Cellular component | GO:0009523 | photosystem II | 0.0002 | 22 | 0 | 22 |  |
| Cellular component | GO:0009534 | chloroplast thylakoid | 0.0002 | 59 | 4 | 55 |  |
| Cellular component | GO:0031976 | plastid thylakoid | 0.0002 | 59 | 4 | 55 |  |
| Cellular component | GO:0044436 | thylakoid part | 0.0003 | 57 | 4 | 53 |  |
| Cellular component | GO:0034357 | photosynthetic membrane | 0.0003 | 55 | 3 | 52 |  |
| Cellular component | GO:0042651 | thylakoid membrane | 0.0003 | 55 | 3 | 52 |  |
| Cellular component | GO:0044435 | plastid part | 0.0004 | 101 | 9 | 92 |  |
| Cellular component | GO:0009535 | chloroplast thylakoid membrane | 0.0004 | 54 | 3 | 51 |  |
| Cellular component | GO:0055035 | plastid thylakoid membrane | 0.0004 | 54 | 3 | 51 |  |
| Cellular component | GO:0044434 | chloroplast part | 0.0004 | 100 | 9 | 91 |  |
| Cellular component | GO:0009521 | photosystem | 0.0004 | 29 | 0 | 29 |  |
| Cellular component | GO:0009522 | photosystem I | 0.0008 | 18 | 0 | 18 |  |
| Cellular component | GO:0005777 | peroxisome | 0.0008 | 16 | 2 | 14 |  |
| Cellular component | GO:0042579 | microbody | 0.0008 | 16 | 2 | 14 |  |
| Cellular component | GO:0009570 | chloroplast stroma | 0.0038 | 41 | 5 | 36 |  |
| Cellular component | GO:0009532 | plastid stroma | 0.0049 | 41 | 5 | 36 |  |
| Cellular component | GO:0042995 | cell projection | 0.0160 | 10 | 3 | 7 |  |
| Cellular component | GO:0090406 | pollen tube | 0.0160 | 10 | 3 | 7 |  |
| Cellular component | GO:0120025 | plasma membrane bounded cell projection | 0.0160 | 10 | 3 | 7 |  |
| Cellular component | GO:0000786 | nucleosome | 0.0175 | 9 | 9 | 0 |  |
| Cellular component | GO:0032993 | protein-DNA complex | 0.0175 | 9 | 9 | 0 |  |
| Cellular component | GO:0044815 | DNA packaging complex | 0.0175 | 9 | 9 | 0 |  |
| Cellular component | GO:0031977 | thylakoid lumen | 0.0388 | 7 | 1 | 6 |  |
| Cellular component | GO:0031984 | organelle subcompartment | 0.0514 | 117 | 32 | 85 |  |
| Cellular component | GO:0009507 | chloroplast | 0.0594 | 182 | 27 | 155 |  |
| Cellular component | GO:0010287 | plastoglobule | 0.0770 | 6 | 3 | 3 |  |
| Molecular function | GO:0016705 | oxidoreductase activity, acting on paired donors, with incorporation or reduction of molecular oxygen | 0.0009 | 54 | 16 | 38 |  |
| Molecular function | GO:0016491 | oxidoreductase activity | 0.0010 | 157 | 50 | 107 |  |
| Molecular function | GO:0070011 | peptidase activity, acting on L-amino acid peptides | 0.0022 | 38 | 22 | 16 |  |
| Molecular function | GO:0051213 | dioxygenase activity | 0.0028 | 14 | 5 | 9 |  |
| Molecular function | GO:0008233 | peptidase activity | 0.0030 | 38 | 22 | 16 |  |
| Molecular function | GO:0048037 | cofactor binding | 0.0042 | 118 | 24 | 94 |  |
| Molecular function | GO:0004497 | monooxygenase activity | 0.0043 | 51 | 14 | 37 |  |
| Molecular function | GO:0046906 | tetrapyrrole binding | 0.0050 | 71 | 11 | 60 |  |
| Molecular function | GO:0004175 | endopeptidase activity | 0.0059 | 32 | 18 | 14 |  |
| Molecular function | GO:0035251 | UDP-glucosyltransferase activity | 0.0075 | 35 | 8 | 27 |  |
| Molecular function | GO:0008236 | serine-type peptidase activity | 0.0091 | 14 | 8 | 6 |  |
| Molecular function | GO:0017171 | serine hydrolase activity | 0.0091 | 14 | 8 | 6 |  |
| Molecular function | GO:0004252 | serine-type endopeptidase activity | 0.0105 | 12 | 7 | 5 |  |
| Molecular function | GO:0001134 | transcription regulator recruiting activity | 0.0111 | 11 | 5 | 6 |  |
| Molecular function | GO:0001135 | RNA polymerase II transcription regulator recruiting activity | 0.0111 | 11 | 5 | 6 |  |
| Molecular function | GO:0016701 | oxidoreductase activity, acting on single donors with incorporation of molecular oxygen | 0.0118 | 9 | 2 | 7 |  |
| Molecular function | GO:0016835 | carbon-oxygen lyase activity | 0.0123 | 16 | 5 | 11 |  |
| Molecular function | GO:0008194 | UDP-glycosyltransferase activity | 0.0132 | 43 | 13 | 30 |  |
| Molecular function | GO:0046527 | glucosyltransferase activity | 0.0205 | 35 | 8 | 27 |  |
| Molecular function | GO:0016758 | transferase activity, transferring hexosyl groups | 0.0214 | 55 | 18 | 37 |  |
| Molecular function | GO:0016860 | intramolecular oxidoreductase activity | 0.0241 | 8 | 1 | 7 |  |
| Molecular function | GO:0016702 | oxidoreductase activity, acting on single donors with incorporation of molecular oxygen, incorporation of two atoms of oxygen | 0.0247 | 7 | 1 | 6 |  |
| Molecular function | GO:0016829 | lyase activity | 0.0257 | 35 | 12 | 23 |  |
| Molecular function | GO:0005506 | iron ion binding | 0.0290 | 45 | 11 | 34 |  |
| Molecular function | GO:0016709 | oxidoreductase activity, acting on paired donors, with incorporation or reduction of molecular oxygen, NAD(P)H as one donor, and incorporation of one atom of oxygen | 0.0358 | 26 | 11 | 15 |  |
| Molecular function | GO:0004869 | cysteine-type endopeptidase inhibitor activity | 0.0360 | 10 | 1 | 9 |  |
| Molecular function | GO:0016168 | chlorophyll binding | 0.0369 | 19 | 0 | 19 |  |
| Molecular function | GO:0043531 | ADP binding | 0.0369 | 19 | 11 | 8 |  |
| Molecular function | GO:0016757 | transferase activity, transferring glycosyl groups | 0.0377 | 70 | 26 | 44 |  |
| Molecular function | GO:0051537 | 2 iron, 2 sulfur cluster binding | 0.0399 | 9 | 2 | 7 |  |
| Molecular function | GO:0016746 | transferase activity, transferring acyl groups | 0.0416 | 38 | 14 | 24 |  |
| Molecular function | GO:0020037 | heme binding | 0.0438 | 51 | 11 | 40 |  |
| Molecular function | GO:0016836 | hydro-lyase activity | 0.0439 | 8 | 3 | 5 |  |
| Molecular function | GO:0046915 | transition metal ion transmembrane transporter activity | 0.0439 | 8 | 1 | 7 |  |
| Molecular function | GO:0004190 | aspartic-type endopeptidase activity | 0.0497 | 14 | 9 | 5 |  |
| Molecular function | GO:0070001 | aspartic-type peptidase activity | 0.0497 | 14 | 9 | 5 |  |
| Molecular function | GO:0016853 | isomerase activity | 0.0508 | 23 | 9 | 14 |  |
| Molecular function | GO:0080043 | quercetin 3-O-glucosyltransferase activity | 0.0533 | 16 | 6 | 10 |  |
| Molecular function | GO:0080044 | quercetin 7-O-glucosyltransferase activity | 0.0533 | 16 | 6 | 10 |  |
| Molecular function | GO:0004672 | protein kinase activity | 0.0659 | 110 | 48 | 62 |  |
| Molecular function | GO:0008134 | transcription factor binding | 0.0686 | 14 | 8 | 6 |  |
| Molecular function | GO:0043621 | protein self-association | 0.0719 | 8 | 2 | 6 |  |
| Biological process | GO:0009765 | photosynthesis, light harvesting | 0.0000 | 13 | 0 | 13 |  |
| Biological process | GO:0015979 | photosynthesis | 0.0000 | 50 | 0 | 50 |  |
| Biological process | GO:0019752 | carboxylic acid metabolic process | 0.0000 | 84 | 22 | 62 |  |
| Biological process | GO:0008299 | isoprenoid biosynthetic process | 0.0000 | 26 | 8 | 18 |  |
| Biological process | GO:0016114 | terpenoid biosynthetic process | 0.0000 | 23 | 6 | 17 |  |
| Biological process | GO:0016053 | organic acid biosynthetic process | 0.0001 | 55 | 16 | 39 |  |
| Biological process | GO:0046394 | carboxylic acid biosynthetic process | 0.0001 | 55 | 16 | 39 |  |
| Biological process | GO:0044283 | small molecule biosynthetic process | 0.0001 | 65 | 19 | 46 |  |
| Biological process | GO:0006720 | isoprenoid metabolic process | 0.0001 | 27 | 8 | 19 |  |
| Biological process | GO:0006629 | lipid metabolic process | 0.0001 | 72 | 19 | 53 |  |
| Biological process | GO:0006721 | terpenoid metabolic process | 0.0001 | 24 | 6 | 18 |  |
| Biological process | GO:0008610 | lipid biosynthetic process | 0.0003 | 46 | 14 | 32 |  |
| Biological process | GO:0014070 | response to organic cyclic compound | 0.0003 | 38 | 18 | 20 |  |
| Biological process | GO:0006082 | organic acid metabolic process | 0.0004 | 92 | 24 | 68 |  |
| Biological process | GO:0043436 | oxoacid metabolic process | 0.0004 | 92 | 24 | 68 |  |
| Biological process | GO:0019684 | photosynthesis, light reaction | 0.0004 | 26 | 0 | 26 |  |
| Biological process | GO:0065008 | regulation of biological quality | 0.0005 | 98 | 43 | 55 |  |
| Biological process | GO:0055080 | cation homeostasis | 0.0005 | 23 | 11 | 12 |  |
| Biological process | GO:0046148 | pigment biosynthetic process | 0.0005 | 22 | 7 | 15 |  |
| Biological process | GO:0098771 | inorganic ion homeostasis | 0.0008 | 23 | 11 | 12 |  |
| Biological process | GO:0009642 | response to light intensity | 0.0008 | 15 | 2 | 13 |  |
| Biological process | GO:0042440 | pigment metabolic process | 0.0009 | 22 | 7 | 15 |  |
| Biological process | GO:1901605 | alpha-amino acid metabolic process | 0.0011 | 31 | 8 | 23 |  |
| Biological process | GO:0044255 | cellular lipid metabolic process | 0.0013 | 56 | 14 | 42 |  |
| Biological process | GO:0031407 | oxylipin metabolic process | 0.0013 | 9 | 3 | 6 |  |
| Biological process | GO:0031408 | oxylipin biosynthetic process | 0.0013 | 9 | 3 | 6 |  |
| Biological process | GO:0009741 | response to brassinosteroid | 0.0015 | 17 | 8 | 9 |  |
| Biological process | GO:0055076 | transition metal ion homeostasis | 0.0018 | 12 | 4 | 8 |  |
| Biological process | GO:0044281 | small molecule metabolic process | 0.0020 | 127 | 39 | 88 |  |
| Biological process | GO:0010035 | response to inorganic substance | 0.0023 | 77 | 32 | 45 |  |
| Biological process | GO:0046677 | response to antibiotic | 0.0028 | 27 | 12 | 15 |  |
| Biological process | GO:0055065 | metal ion homeostasis | 0.0028 | 16 | 6 | 10 |  |
| Biological process | GO:0009414 | response to water deprivation | 0.0031 | 44 | 21 | 23 |  |
| Biological process | GO:0042542 | response to hydrogen peroxide | 0.0039 | 10 | 3 | 7 |  |
| Biological process | GO:0009415 | response to water | 0.0040 | 44 | 21 | 23 |  |
| Biological process | GO:0032787 | monocarboxylic acid metabolic process | 0.0043 | 47 | 14 | 33 |  |
| Biological process | GO:0050801 | ion homeostasis | 0.0069 | 23 | 11 | 12 |  |
| Biological process | GO:0044282 | small molecule catabolic process | 0.0071 | 20 | 5 | 15 |  |
| Biological process | GO:0009742 | brassinosteroid mediated signaling pathway | 0.0072 | 12 | 6 | 6 |  |
| Biological process | GO:0043401 | steroid hormone mediated signaling pathway | 0.0072 | 12 | 6 | 6 |  |
| Biological process | GO:0048545 | response to steroid hormone | 0.0072 | 12 | 6 | 6 |  |
| Biological process | GO:0071367 | cellular response to brassinosteroid stimulus | 0.0072 | 12 | 6 | 6 |  |
| Biological process | GO:0071383 | cellular response to steroid hormone stimulus | 0.0072 | 12 | 6 | 6 |  |
| Biological process | GO:0006875 | cellular metal ion homeostasis | 0.0078 | 11 | 5 | 6 |  |
| Biological process | GO:0030003 | cellular cation homeostasis | 0.0078 | 11 | 5 | 6 |  |
| Biological process | GO:0006631 | fatty acid metabolic process | 0.0079 | 22 | 4 | 18 |  |
| Biological process | GO:0042445 | hormone metabolic process | 0.0080 | 31 | 11 | 20 |  |
| Biological process | GO:0072330 | monocarboxylic acid biosynthetic process | 0.0080 | 33 | 11 | 22 |  |
| Biological process | GO:0016054 | organic acid catabolic process | 0.0081 | 19 | 5 | 14 |  |
| Biological process | GO:0046395 | carboxylic acid catabolic process | 0.0081 | 19 | 5 | 14 |  |
| Biological process | GO:0006714 | sesquiterpenoid metabolic process | 0.0086 | 9 | 2 | 7 |  |
| Biological process | GO:0009644 | response to high light intensity | 0.0086 | 9 | 1 | 8 |  |
| Biological process | GO:0009687 | abscisic acid metabolic process | 0.0086 | 9 | 2 | 7 |  |
| Biological process | GO:0043288 | apocarotenoid metabolic process | 0.0086 | 9 | 2 | 7 |  |
| Biological process | GO:1902644 | tertiary alcohol metabolic process | 0.0086 | 9 | 2 | 7 |  |
| Biological process | GO:0046916 | cellular transition metal ion homeostasis | 0.0087 | 8 | 4 | 4 |  |
| Biological process | GO:0010817 | regulation of hormone levels | 0.0088 | 45 | 20 | 25 |  |
| Biological process | GO:0080167 | response to karrikin | 0.0088 | 15 | 9 | 6 |  |
| Biological process | GO:1901607 | alpha-amino acid biosynthetic process | 0.0088 | 15 | 3 | 12 |  |
| Biological process | GO:0019725 | cellular homeostasis | 0.0090 | 28 | 12 | 16 |  |
| Biological process | GO:0006520 | cellular amino acid metabolic process | 0.0092 | 32 | 8 | 24 |  |
| Biological process | GO:0042446 | hormone biosynthetic process | 0.0097 | 23 | 9 | 14 |  |
| Biological process | GO:0016051 | carbohydrate biosynthetic process | 0.0102 | 35 | 16 | 19 |  |
| Biological process | GO:0009835 | fruit ripening | 0.0126 | 12 | 3 | 9 |  |
| Biological process | GO:1901700 | response to oxygen-containing compound | 0.0130 | 141 | 57 | 84 |  |
| Biological process | GO:0008652 | cellular amino acid biosynthetic process | 0.0139 | 15 | 3 | 12 |  |
| Biological process | GO:0006873 | cellular ion homeostasis | 0.0141 | 11 | 5 | 6 |  |
| Biological process | GO:0044550 | secondary metabolite biosynthetic process | 0.0153 | 39 | 16 | 23 |  |
| Biological process | GO:0008283 | cell proliferation | 0.0171 | 9 | 6 | 3 |  |
| Biological process | GO:0009063 | cellular amino acid catabolic process | 0.0181 | 13 | 5 | 8 |  |
| Biological process | GO:1901606 | alpha-amino acid catabolic process | 0.0181 | 13 | 5 | 8 |  |
| Biological process | GO:0071407 | cellular response to organic cyclic compound | 0.0181 | 16 | 8 | 8 |  |
| Biological process | GO:0000041 | transition metal ion transport | 0.0184 | 8 | 1 | 7 |  |
| Biological process | GO:0006778 | porphyrin-containing compound metabolic process | 0.0184 | 8 | 0 | 8 |  |
| Biological process | GO:0033013 | tetrapyrrole metabolic process | 0.0184 | 8 | 0 | 8 |  |
| Biological process | GO:0042592 | homeostatic process | 0.0187 | 47 | 20 | 27 |  |
| Biological process | GO:0040008 | regulation of growth | 0.0189 | 39 | 17 | 22 |  |
| Biological process | GO:0010345 | suberin biosynthetic process | 0.0194 | 7 | 2 | 5 |  |
| Biological process | GO:0015977 | carbon fixation | 0.0194 | 7 | 0 | 7 |  |
| Biological process | GO:0019253 | reductive pentose-phosphate cycle | 0.0194 | 7 | 0 | 7 |  |
| Biological process | GO:0019685 | photosynthesis, dark reaction | 0.0194 | 7 | 0 | 7 |  |
| Biological process | GO:0006091 | generation of precursor metabolites and energy | 0.0194 | 32 | 1 | 31 |  |
| Biological process | GO:0009408 | response to heat | 0.0209 | 15 | 3 | 12 |  |
| Biological process | GO:0030001 | metal ion transport | 0.0209 | 22 | 10 | 12 |  |
| Biological process | GO:0046165 | alcohol biosynthetic process | 0.0267 | 10 | 3 | 7 |  |
| Biological process | GO:1901615 | organic hydroxy compound metabolic process | 0.0270 | 26 | 7 | 19 |  |
| Biological process | GO:1901617 | organic hydroxy compound biosynthetic process | 0.0273 | 18 | 6 | 12 |  |
| Biological process | GO:0006066 | alcohol metabolic process | 0.0277 | 13 | 3 | 10 |  |
| Biological process | GO:0018298 | protein-chromophore linkage | 0.0278 | 20 | 1 | 19 |  |
| Biological process | GO:0034637 | cellular carbohydrate biosynthetic process | 0.0278 | 22 | 10 | 12 |  |
| Biological process | GO:0009651 | response to salt stress | 0.0301 | 43 | 11 | 32 |  |
| Biological process | GO:0009072 | aromatic amino acid family metabolic process | 0.0320 | 12 | 5 | 7 |  |
| Biological process | GO:0040007 | growth | 0.0320 | 69 | 34 | 35 |  |
| Biological process | GO:0006558 | L-phenylalanine metabolic process | 0.0369 | 11 | 5 | 6 |  |
| Biological process | GO:1902221 | erythrose 4-phosphate/phosphoenolpyruvate family amino acid metabolic process | 0.0369 | 11 | 5 | 6 |  |
| Biological process | GO:0006970 | response to osmotic stress | 0.0381 | 51 | 14 | 37 |  |
| Biological process | GO:0006779 | porphyrin-containing compound biosynthetic process | 0.0381 | 7 | 0 | 7 |  |
| Biological process | GO:0009116 | nucleoside metabolic process | 0.0381 | 7 | 2 | 5 |  |
| Biological process | GO:0015994 | chlorophyll metabolic process | 0.0381 | 7 | 0 | 7 |  |
| Biological process | GO:0015995 | chlorophyll biosynthetic process | 0.0381 | 7 | 0 | 7 |  |
| Biological process | GO:0033014 | tetrapyrrole biosynthetic process | 0.0381 | 7 | 0 | 7 |  |
| Biological process | GO:0042127 | regulation of cell proliferation | 0.0381 | 7 | 5 | 2 |  |
| Biological process | GO:0044038 | cell wall macromolecule biosynthetic process | 0.0426 | 10 | 8 | 2 |  |
| Biological process | GO:0070589 | cellular component macromolecule biosynthetic process | 0.0426 | 10 | 8 | 2 |  |
| Biological process | GO:0009751 | response to salicylic acid | 0.0429 | 17 | 9 | 8 |  |
| Biological process | GO:0001101 | response to acid chemical | 0.0491 | 102 | 43 | 59 |  |
| Biological process | GO:0030243 | cellulose metabolic process | 0.0492 | 9 | 4 | 5 |  |
| Biological process | GO:0031324 | negative regulation of cellular metabolic process | 0.0534 | 26 | 7 | 19 |  |
| Biological process | GO:0006812 | cation transport | 0.0544 | 35 | 12 | 23 |  |
| Biological process | GO:0010928 | regulation of auxin mediated signaling pathway | 0.0547 | 11 | 7 | 4 |  |
| Biological process | GO:0055082 | cellular chemical homeostasis | 0.0547 | 11 | 5 | 6 |  |
| Biological process | GO:1901565 | organonitrogen compound catabolic process | 0.0565 | 67 | 28 | 39 |  |
| Biological process | GO:0009699 | phenylpropanoid biosynthetic process | 0.0568 | 17 | 7 | 10 |  |
| Biological process | GO:0045454 | cell redox homeostasis | 0.0568 | 17 | 7 | 10 |  |
| Biological process | GO:0030244 | cellulose biosynthetic process | 0.0569 | 8 | 3 | 5 |  |
| Biological process | GO:0048878 | chemical homeostasis | 0.0581 | 30 | 13 | 17 |  |
| Biological process | GO:0055067 | monovalent inorganic cation homeostasis | 0.0638 | 10 | 6 | 4 |  |
| Biological process | GO:0009628 | response to abiotic stimulus | 0.0639 | 144 | 49 | 95 |  |
| Biological process | GO:0019748 | secondary metabolic process | 0.0645 | 54 | 22 | 32 |  |
| Biological process | GO:0042493 | response to drug | 0.0649 | 50 | 17 | 33 |  |
| Biological process | GO:0006885 | regulation of pH | 0.0657 | 7 | 5 | 2 |  |
| Biological process | GO:0048509 | regulation of meristem development | 0.0657 | 7 | 3 | 4 |  |
| Biological process | GO:1901659 | glycosyl compound biosynthetic process | 0.0657 | 7 | 0 | 7 |  |
