# Supplementary material for: Comprehensive Analysis of Transcriptome and Metabolome Reveals the Flavonoid Metabolic Pathway Is Associated with Fruit Peel Coloration of Melon
Source: Molecules. 2021 May 10;26(9):2830. doi: 10.3390/molecules26092830 (PMC8126211; doi:10.3390/molecules26092830)
Supplement: Supplementary file 1 [file molecules-26-02830-s001.zip › molecules-1183709-supplementary/Table S8 differentially expressed metabolites in W vs B.docx]

| **Table S8 differentially expressed metabolites in W vs B** | | | | |
| --- | --- | --- | --- | --- |
| **Index** | **Compounds** | **Class** | **LogFC** | **type** |
| pma0760 | Selgin O-malonylhexoside | Flavone | -1.7996 | down |
| pma1108 | Apigenin C-glucoside | Flavone | -12.7195 | down |
| pma6229 | Eriodictyol C-hexoside | Flavone | 5.3734 | up |
| pma6373 | 3',4',5'-Dihydrotricetin O-hexosyl-O-hexoside | Flavone | -2.2446 | down |
| pma6496 | Luteolin 6-C-glucoside | Flavone | 5.7557 | up |
| pma6516 | C-hexosyl-apigenin O-hexosyl-O-hexoside | Flavone | -18.5215 | down |
| pma6518 | C-pentosyl-chrysoeriol 7-O-feruloylhexoside | Flavone | -12.9577 | down |
| pma6638 | O-methylChrysoeriol 7-O-hexoside | Flavone | 12.6480 | up |
| pmb0563 | Peonidin | Anthocyanins | -12.2476 | down |
| pmb0588 | Luteolin 3',7-di-O-glucoside | Flavone | 5.0934 | up |
| pmb0592 | Chrysoeriol O-hexosyl-O-rutinoside | Flavone | -18.9443 | down |
| pmb0603 | Chrysoeriol O-hexosyl-O-hexoside | Flavone | 4.3293 | up |
| pmb0604 | Kaempferol 3-O-glucoside (Astragalin) | Flavonol | -13.5436 | down |
| pmb0613 | Apigenin 6-C-hexosyl-8-C-hexosyl-O-hexoside | Flavone | 2.7774 | up |
| pmb0618 | 8-C-hexosyl-hesperetin O-hexoside | Flavone | 4.4416 | up |
| pmb0620 | Chrysoeriol 6-C-hexoside 8-C-hexoside-O-hexoside | Flavone | 3.9891 | up |
| pmb0623 | 6-C-hexosyl chrysoeriol O-hexoside | Flavone | 4.3098 | up |
| pmb0626 | 6-C-hexosyl-apigenin O-hexosyl-O-hexoside | Flavone | 11.6801 | up |
| pmb0629 | Chrysoeriol 6-C-hexoside | Flavone | 13.1842 | up |
| pmb0649 | C-hexosyl-luteolin O-hexosyl-O-salicylic acid | Flavone | -15.5362 | down |
| pmb0652 | C-hexosyl-apigenin O-pentoside | Flavone | 12.5946 | up |
| pmb0657 | C-rhamnosyl-acacetin O-p-coumaroylhexoside | Flavone | -6.8833 | down |
| pmb0660 | C-hexosyl-luteolin O-p-coumaroylhexoside | Flavone | -6.4221 | down |
| pmb0668 | Apigenin C-hexosyl-O-hexosyl-O-Salicylic acid | Flavone | -6.4145 | down |
| pmb0672 | 6-C-hexosyl-apigenin O-feruloylhexoside | Flavone | -8.2071 | down |
| pmb0675 | C-pentosyl-apigenin O-p-coumaroylhexoside | Flavone | 3.5451 | up |
| pmb0678 | 8-C-hexosyl-apigenin O-feruloylhexoside | Flavone | -12.7226 | down |
| pmb0680 | C-hexosyl-apigenin O-p-coumaroylhexoside | Flavone | -7.7638 | down |
| pmb0682 | 8-C-hexosyl-apigenin O-sinapoylhexoside | Flavone | -15.4636 | down |
| pmb0701 | Chrysoeriol 8-C-hexoside | Flavone | 4.7385 | up |
| pmb0703 | 6-C-hexosyl-chrysoeriol O-feruloylhexoside | Flavone | -15.4800 | down |
| pmb0712 | Tricin 5-O-hexosyl-O-hexoside | Flavone | -14.5838 | down |
| pmb0713 | Tricin 7-O-hexosyl-O-hexoside | Flavone | -14.6266 | down |
| pmb0725 | Tricin 7-O-feruloylhexoside | Flavone | 2.2694 | up |
| pmb2954 | Luteolin O-hexosyl-O-hexosyl-O-hexoside | Flavone | 5.4965 | up |
| pmb2970 | Hesperetin O-hexosyl-O-hexoside | Flavanone | -9.4821 | down |
| pmb2976 | Chrysoeriol C-pentosyl-O-hexosyl-O-hexoside | Flavone | -11.6884 | down |
| pmb2983 | Chrysoeriol C-pentosyl-O-rhamnosyl-rhamnoside | Flavone | -8.6780 | down |
| pmb2984 | Acetyl-eriodictyol O-hexoside | Flavone | 11.4184 | up |
| pmb2991 | Apigenin O-hexosyl-O-rutinoside | Flavone | -12.3729 | down |
| pmb3013 | Isorhamnetin O-acetyl-hexoside | Flavonol | -1.7080 | down |
| pme0205 | Catechin | Polyphenol | 2.0217 | up |
| pme0368 | Apigenin 7-rutinoside (Isorhoifolin) | Flavone | -11.0790 | down |
| pme0426 | 4-Methylcatechol | Polyphenol | 10.3156 | up |
| pme2459 | Luteolin 7-O-glucoside (Cynaroside) | Flavone | -16.6729 | down |
| pme3211 | Quercetin 3-O-glucoside (Isotrifoliin) | Flavonol | 10.5652 | up |
| pmf0369 | Persicoside | Flavonoid | 2.2700 | up |
| pmf0375 | Isorhamnetin 3-O-glucoside | Flavonoid | 13.4008 | up |
| pmf0582 | Glabridin | Flavonoid | 3.0505 | up |
