# Supplementary material for: Comprehensive Analysis of Transcriptome and Metabolome Reveals the Flavonoid Metabolic Pathway Is Associated with Fruit Peel Coloration of Melon
Source: Molecules. 2021 May 10;26(9):2830. doi: 10.3390/molecules26092830 (PMC8126211; doi:10.3390/molecules26092830)
Supplement: Supplementary file 1 [file molecules-26-02830-s001.zip › molecules-1183709-supplementary/Table S9 differentially expressed metabolites in W vs H.docx]

| **Table S9 differentially expressed metabolites in W vs H** | | | | |
| --- | --- | --- | --- | --- |
| **Index** | **Compounds** | **Class** | **LogFC** | **type** |
| pma0214 | methylQuercetin O-hexoside | Flavonol | 12.8182 | up |
| pma0249 | Selgin 5-O-hexoside | Flavone | 14.6118 | up |
| pma0253 | O-methylChrysoeriol 5-O-hexoside | Flavone | 16.8173 | up |
| pma0791 | Naringenin O-malonylhexoside | Flavanone | 18.4246 | up |
| pma0795 | Tricetin O-malonylhexoside | Flavone | 20.8991 | up |
| pma1116 | Kaempferide | Flavonol | 21.9607 | up |
| pma6218 | O-methylnaringenin C-pentoside | Flavone | 16.9208 | up |
| pma6353 | Chrysin C-hexoside | Flavone | 18.0045 | up |
| pma6373 | 3',4',5'-Dihydrotricetin O-hexosyl-O-hexoside | Flavone | -13.0469 | down |
| pma6638 | O-methylChrysoeriol 7-O-hexoside | Flavone | 16.8948 | up |
| pma6639 | Isorhamnetin O-hexoside | Flavonol | 16.5362 | up |
| pmb0541 | Cyanidin 3-O-glucosyl-malonylglucoside | Anthocyanins | -12.1405 | down |
| pmb0566 | Luteolin O-hexosyl-O-pentoside | Flavone | 14.3657 | up |
| pmb0576 | Apigenin O-malonylhexoside | Flavone | 13.7068 | up |
| pmb0578 | Luteolin O-sinapoylhexoside | Flavone | 17.8182 | up |
| pmb0595 | Isorhamnetin 5-O-hexoside | Flavonol | 16.4075 | up |
| pmb0607 | Chrysoeriol 7-O-hexoside | Flavone | 19.7813 | up |
| pmb0608 | Chrysoeriol O-malonylhexoside | Flavone | 5.1105 | up |
| pmb0623 | 6-C-hexosyl chrysoeriol O-hexoside | Flavone | 4.6853 | up |
| pmb0624 | 6-C-hexosyl-luteolin O-hexoside | Flavone | 18.1046 | up |
| pmb0626 | 6-C-hexosyl-apigenin O-hexosyl-O-hexoside | Flavone | 14.3298 | up |
| pmb0628 | Eriodictiol C-hexosyl-O-hexoside | Flavone | 2.9283 | up |
| pmb0629 | Chrysoeriol 6-C-hexoside | Flavone | 16.1475 | up |
| pmb0636 | 6-C-hexosyl luteolin O-pentoside | Flavone | 5.2615 | up |
| pmb0645 | 6-C-hexosyl-hesperetin O-hexoside | Flavone | -2.7467 | down |
| pmb0652 | C-hexosyl-apigenin O-pentoside | Flavone | 15.2729 | up |
| pmb0661 | Chrysoeriol C-hexosyl-O-rhamnoside | Flavone | 5.1974 | up |
| pmb0674 | C-pentosyl apigenin O-salicyloyl hexoside | Flavone | -13.8293 | down |
| pmb0679 | C-rhamnosyl-apigenin O-feruloylhexoside | Flavone | 12.8048 | up |
| pmb0684 | C-hexosyl-luteolin O-sinapic acid | Flavone | 17.5728 | up |
| pmb0686 | Eriodictyol O-malonylhexoside | Flavanone | 19.0485 | up |
| pmb0696 | 8-C-hexosyl chrysoeriol O-hexoside | Flavone | -14.6927 | down |
| pmb0711 | Quercetin 7-O-rutinoside | Flavonol | -13.3386 | down |
| pmb0717 | Tricin 5-O-β-guaiacylglycerol | Flavone | -12.8988 | down |
| pmb0732 | Tricin 5-O-feruloylhexoside | Flavone | 4.8230 | up |
| pmb0736 | Tricin 7-O-hexoside | Flavone | 4.4096 | up |
| pmb0739 | Tricin O-hexosyl-O-syringin alcohol | Flavone | -13.0664 | down |
| pmb0743 | Tricin 7-O-β-guaiacylglycerol | Flavone | 13.2199 | up |
| pmb0744 | Tricin O-phenylformic acid | Flavone | -12.1755 | down |
| pmb0835 | Gallocatechin-gallocatechin | Polyphenol | -13.6228 | down |
| pmb1108 | Luteolin 6-C-hexoside 8-C-hexosyl-O-hexoside | Flavone | -15.6032 | down |
| pmb2831 | Protocatechuic acid O-glucoside | Polyphenol | -16.0207 | down |
| pmb2954 | Luteolin O-hexosyl-O-hexosyl-O-hexoside | Flavone | 6.6409 | up |
| pmb2961 | Peonidin O-malonylhexoside | Anthocyanins | -15.7157 | down |
| pmb2975 | Hesperetin O-Glucuronic acid | Flavanone | -10.8974 | down |
| pmb2977 | Chrysoeriol 8-C-pentosyl-O-rutinoside | Flavone | -10.4266 | down |
| pmb2984 | Acetyl-eriodictyol O-hexoside | Flavone | 11.6049 | up |
| pmb2986 | Chrysoeriol O-hexosyl-O-malonylhexoside | Flavone | -9.8651 | down |
| pmb2997 | Chrysoeriol O-hexosyl-O-hexosyl-O-Glucuronic acid | Flavone | 15.7845 | up |
| pmb3006 | Apigenin 7-O-glucoside (Cosmosiin) | Flavone | 10.4065 | up |
| pmb3007 | Chrysoeriol O-glucuronic acid | Flavone | 10.7345 | up |
| pmb3019 | Chrysoeriol O-homovanillic acid | Flavone | 13.0027 | up |
| pmb3026 | Quercetin O-acetylhexoside | Flavonol | 12.8388 | up |
| pmb3041 | Tricin O-saccharic acid | Flavone | -13.7321 | down |
| pme0088 | Luteolin | Flavone | 16.9882 | up |
| pme0200 | Kaempferol | Flavonol | 14.6011 | up |
| pme0330 | Naringenin 7-O-neohesperidoside (Naringin) | Flavanone | 11.4777 | up |
| pme0332 | Apigenin 7-O-neohesperidoside (Rhoifolin) | Flavone | 12.1950 | up |
| pme0359 | Apigenin 5-O-glucoside | Flavone | 20.7586 | up |
| pme0371 | Naringenin 7-O-glucoside (Prunin) | Flavanone | 19.0014 | up |
| pme0374 | Isovitexin | Flavone | 7.5130 | up |
| pme0376 | Naringenin | Flavanone | 11.1780 | up |
| pme0379 | Apigenin | Flavone | 8.1009 | up |
| pme0426 | 4-Methylcatechol | Polyphenol | 10.8344 | up |
| pme1201 | Phloretin | Flavanone | 8.4472 | up |
| pme1510 | Baicalein (5,6,7-Trihydroxyflavone) | Flavone | 11.0514 | up |
| pme1521 | Dihydroquercetin (Taxifolin) | Flavonol | 13.5684 | up |
| pme1572 | Orobol (5,7,3',4'-tetrahydroxyisoflavone) | Isoflavone | 19.2607 | up |
| pme1578 | Genistein (4',5,7-Trihydroxyisoflavone) | Isoflavone | 17.5935 | up |
| pme1583 | Eriodictyol | Flavanone | 10.7363 | up |
| pme1588 | Isorhamnetin | Flavonol | 7.3224 | up |
| pme1598 | Hesperetin 5-O-glucoside | Flavanone | 15.1636 | up |
| pme1599 | 7-O-Methyleriodictyol | Flavanone | 16.8615 | up |
| pme1611 | Isohemiphloin | Flavone | 20.1622 | up |
| pme1665 | Isovitexin 7-O-glucoside (Saponarin) | Flavonoid | 20.0401 | up |
| pme1773 | Cyanidin 3-O-rutinoside (Keracyanin) | Anthocyanins | -3.8176 | down |
| pme2296 | Curcumin | Polyphenol | 10.3144 | up |
| pme2319 | Hesperetin | Flavanone | 13.5513 | up |
| pme2957 | Naringenin chalcone | Flavanone | 11.5688 | up |
| pme2963 | Aromadedrin (Dihydrokaempferol) | Flavonol | 20.1572 | up |
| pme2973 | Icariin (kaempferol 3,7-O-diglucoside 8-prenyl derivative) | Flavonol | -11.6505 | down |
| pme3129 | Quercetin 4'-O-glucoside (Spiraeoside) | Flavonol | 19.6216 | up |
| pme3209 | Genistein 7-O-Glucoside (Genistin) | Isoflavone | 16.5881 | up |
| pme3211 | Quercetin 3-O-glucoside (Isotrifoliin) | Flavonol | 15.2662 | up |
| pme3215 | Isoliquiritigenin | Flavanone | 13.6580 | up |
| pme3227 | Vitexin 2''-O-beta-L-rhamnoside | Flavone | 13.6959 | up |
| pme3230 | Calycosin | Isoflavone | 16.0525 | up |
| pme3256 | Delphinidin 3-O-rutinoside (Tulipanin) | Anthocyanins | -12.1639 | down |
| pme3261 | 6-Hydroxydaidzein | Isoflavone | 16.9337 | up |
| pme3267 | Kaempferol 3-O-galactoside (Trifolin) | Flavonol | 17.9695 | up |
| pme3276 | 2'-Hydroxygenistein | Isoflavone | 15.9053 | up |
| pme3300 | Tricetin | Flavone | 9.6216 | up |
| pme3369 | Rhamnetin (7-O-methxyl quercetin) | Flavonol | 9.7771 | up |
| pme3391 | Petunidin 3-O-glucoside | Anthocyanins | -15.7520 | down |
| pme3392 | Pelargonidin 3-O-beta-D-glucoside（Callistephin chloride) | Anthocyanins | 16.1178 | up |
| pme3401 | Syringetin | Flavonol | 5.3098 | up |
| pme3407 | Laricitrin | Flavonol | 11.4870 | up |
| pme3440 | Butein | Flavanone | 16.4313 | up |
| pme3461 | Homoeriodictyol | Flavanone | 14.3144 | up |
| pme3473 | Butin | Flavone | 11.5490 | up |
| pme3502 | Formononetin 7-O-glucoside (Ononin) | Isoflavone | -12.5851 | down |
| pmf0005 | Narirutin | Flavone | 11.5183 | up |
| pmf0057 | 4,2',4',6'-Tetrahydroxychalcone | Flavone | 10.8045 | up |
| pmf0058 | 4',5,7-Trihydroxyflavanone | Flavanone | 11.2998 | up |
| pmf0108 | Garbanzol | Flavanone | 11.5364 | up |
| pmf0279 | Gossypitrin | Flavonoid | -15.1752 | down |
| pmf0301 | Engeletin | Flavonoid | 14.1709 | up |
| pmf0345 | (-)-Epiafzelechin | Polyphenol | 14.5147 | up |
| pmf0361 | Astilbin | Flavonoid | -17.3641 | down |
| pmf0375 | Isorhamnetin 3-O-glucoside | Flavonoid | 16.5832 | up |
| pmf0393 | Persicogenin | Flavonoid | 19.4775 | up |
| pmf0568 | Tectorigenin | Flavonoid | 11.6304 | up |
| pmf0618 | Cyanidin 3-p-hydroxybenzoylsophoroside-5-glucoside | Anthocyanins | -11.2019 | down |
