# Supplementary figures and images for: Comprehensive Analysis of Transcriptome and Metabolome Reveals the Flavonoid Metabolic Pathway Is Associated with Fruit Peel Coloration of Melon
Source: Molecules. 2021 May 10;26(9):2830. doi: 10.3390/molecules26092830 (PMC8126211; doi:10.3390/molecules26092830)

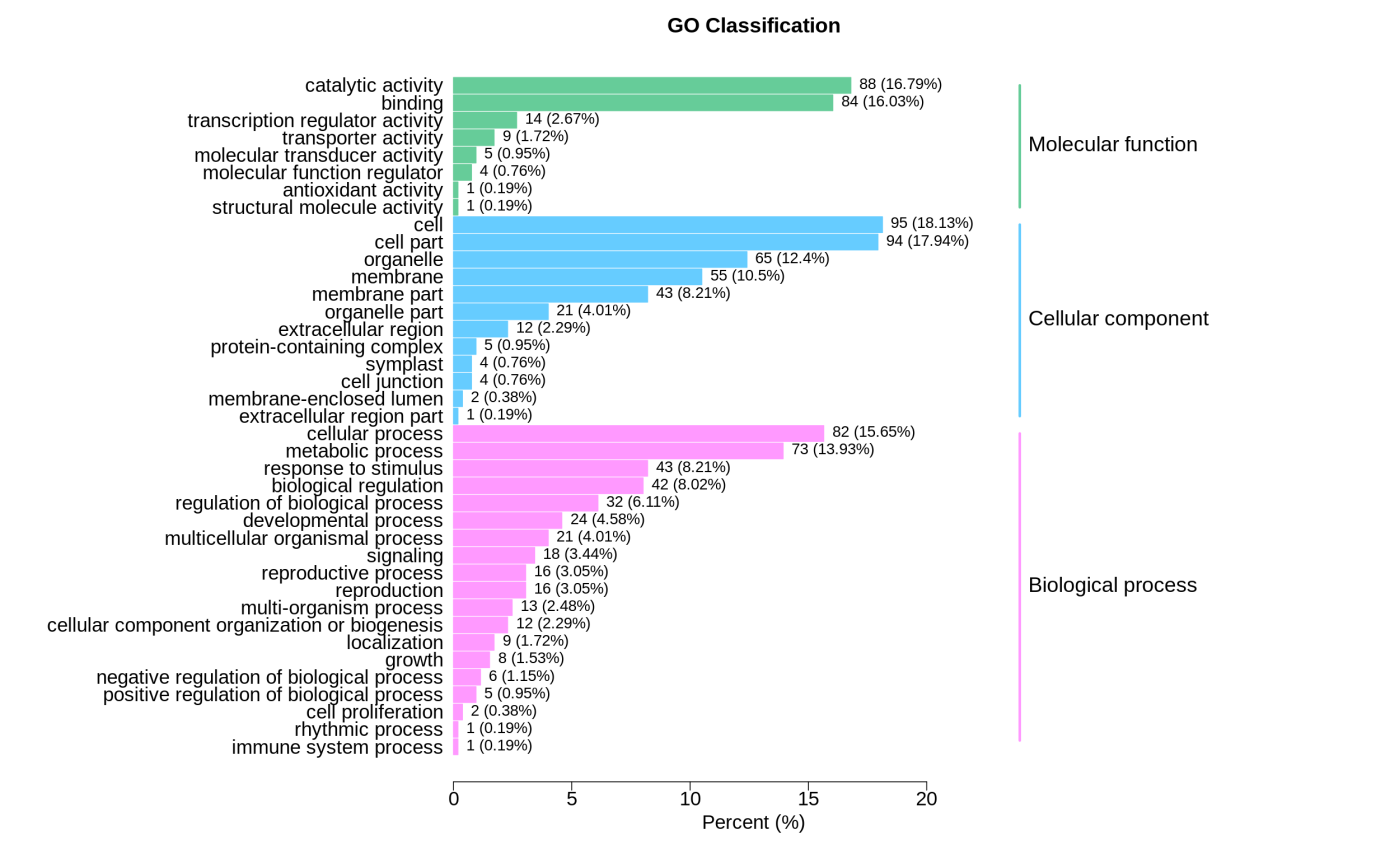


Figure S1 GO analysis of DEGs in W vs. B

Supplement: Supplementary file 1 [file molecules-26-02830-s001.zip › molecules-1183709-supplementary/Figure S1 GO analysis of DEGs in W vs. B.docx]
